# Enantioselective aza-Friedel–Crafts reaction of furan with α-ketimino esters induced by a conjugated double hydrogen bond network of chiral bis(phosphoric acid) catalysts†
†Electronic supplementary information (ESI) available: Experimental procedure, characterization data, additional control experiments, copies of ^1^H NMR and ^13^C NMR spectra of all new compounds. CCDC 1520624, 1520625, 1834631 and 1834632. For ESI and crystallographic data in CIF or other electronic format see DOI: 10.1039/c8sc02290a


**DOI:** 10.1039/c8sc02290a

**Published:** 2018-06-25

**Authors:** Manabu Hatano, Haruka Okamoto, Taro Kawakami, Kohei Toh, Hidefumi Nakatsuji, Akira Sakakura, Kazuaki Ishihara

**Affiliations:** a Graduate School of Engineering , Nagoya University , Furo-cho, Chikusa , Nagoya 464-8603 , Japan . Email: ishihara@cc.nagoya-u.ac.jp ; Fax: +81-52-789-3222 ; Tel: +81-52-789-3331; b Graduate School of Natural Science and Technology , Okayama University , 3-1-1 Tsushimanaka, Kita-ku , Okayama 700-8530 , Japan . Email: sakakura@okayama-u.ac.jp ; Fax: +81-86-251-8215 ; Tel: +81-86-251-8215

## Abstract

Chiral *C*_2_- and *C*_1_-symmetric BINOL-derived bis(phosphoric acid) catalysts, which have OP(=O)(OH)_2_/OP(=O)(OH)(OR) moieties at the 2,2′-positions, were developed and used for the enantioselective aza-Friedel–Crafts reaction of 2-methoxyfuran with α-ketimino esters for the first time. The intramolecular conjugated double hydrogen bond network is a key to increasing the Brønsted acidity and preventing deactivation of the catalysts. Highly functionalized α-amino acid derivatives with a chiral quaternary carbon center could be transformed into versatile optically active *N*- and *O*-heterocycles and an α-aryl-substituted serine.

## Abstract 2

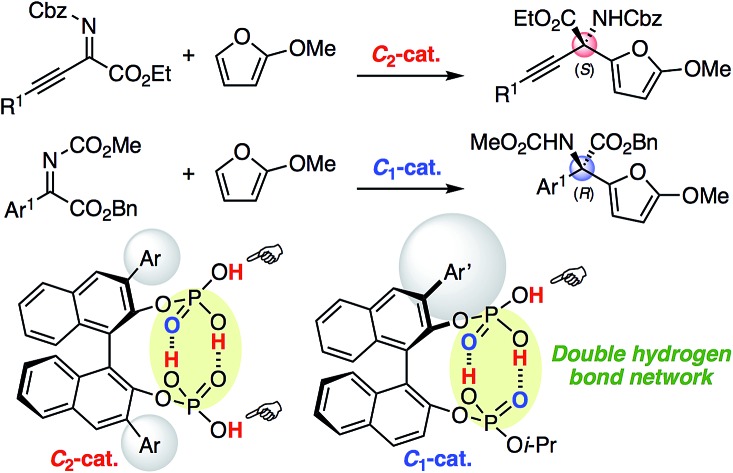
Chiral *C*_2_- and *C*_1_-symmetric BINOL-derived bis(phosphoric acid) catalysts facilitated the enantioselective aza-Friedel–Crafts reaction of 2-methoxyfuran with α-ketimino esters.

## Introduction

**Fig. 1:**
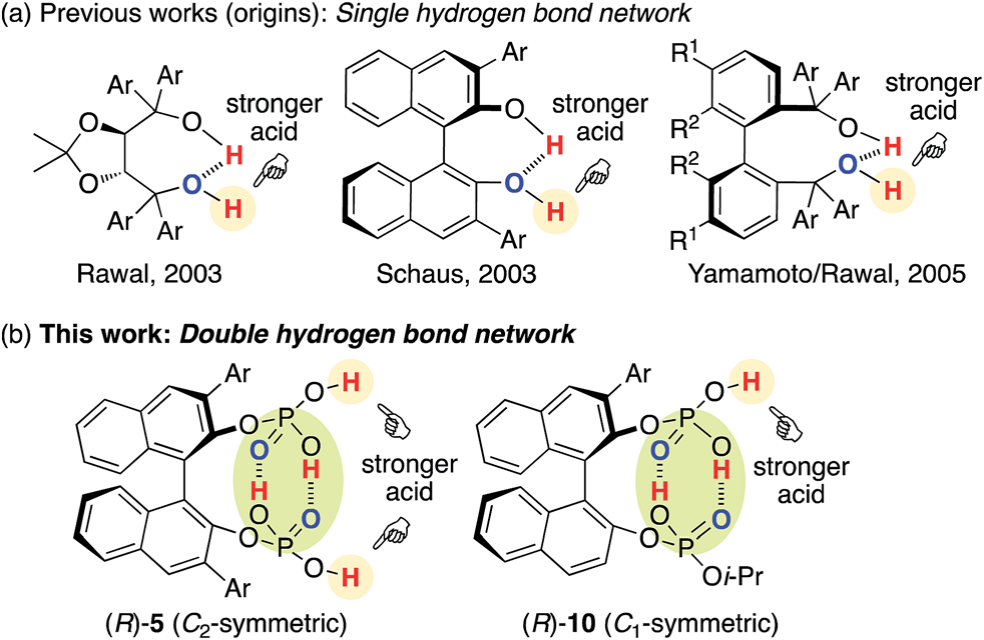
Design of chiral BINOL-derived bis(phosphoric acid) catalysts.

The hydrogen bond network of chiral multiprotic acid catalysts plays an important role in activating Brønsted acidity, controlling conformational flexibility, and producing high enantioselectivity.^1^,^2^ According to the general classification of combined acid catalysts described by Yamamoto,^3^ some chiral Brønsted acid catalysts R*(XH)_2_ with a hydrogen bond network could be considered part of a Brønsted acid-assisted Brønsted acid (BBA) catalyst system. In such a BBA system, one hydrogen atom of an XH group might participate in an intramolecular hydrogen bond with the other XH group, which might be activated and thus used for activation of the substrate. Since 2003, when Rawal reported the first example of an intramolecular single hydrogen bonding network in chiral TADDOLs (α,α,α′,α′-tetraaryl-1,3-dioxolan-4,5-dimethanols)4a,c for the enantioselective hetero-Diels–Alder reaction, and after Schaus developed chiral 3,3′-diaryl-BINOLs (1,1′-bi-2-naphthol)4*b* for the enantioselective Morita–Baylis–Hillman reaction, great effort has been devoted to this research area (Fig. 1a). Chiral 1,1′-biaryl-2,2′-dimethanol (Yamamoto/Rawal),4*d* chiral glycolic acid (Yamamoto),^5^ chiral 2-bis(triflyl)methyl-2′-hydroxy-1,1′-binaphthyl (Ishihara/Yamamoto),^6^ chiral binaphthyl di-carboxylic acids (Maruoka),^7^ 3,3-linked-bis(BINOL)-derived bis(phosphoric acid)s (Gong),^8^ chiral binaphthyl disulfonic acids (BINSA) (our group),^9^ and chiral 3,3′-di(2-hydroxy-3-arylphenyl)-BINOL-derived bis(phosphoric acid)s and chiral carboxylic acid–phosphoric acid combined catalysts (Momiyama/Terada)^10^,^11^ have been developed for a variety of asymmetric catalyses. These outstanding chiral BBA catalysts might have an intramolecular single hydrogen bond network with the use of bis(monoprotic acid)s R*(XH)_2_. In sharp contrast, we envisioned that an intramolecular double hydrogen bond network may represent a new strategy for the design of chiral Brønsted acid catalysts. However, a simple and closed double hydrogen bond network, as seen in a dimeric structure of two molecules of carboxylic acids, would lose both the Brønsted acid- and base-functions upon neutralization. Therefore, here we developed chiral *C*_2_-symmetric BINOL-derived bis(phosphoric acid) catalysts (*R*)-**5** as bis(diprotic acid)s R*(XH_2_)_2_, which have two OP(=O)(OH)_2_ moieties at the 2,2′-positions of the chiral binaphthyl backbone (Fig. 1b). Based on the essence of (*R*)-**5**, chiral *C*_1_-symmetric catalysts (*R*)-**10**, which have OP(=O)(OH)_2_/OP(=O)(OH)(Oi-Pr) moieties, were also developed. Remarkably, outside of the conjugated intramolecular double hydrogen bond network, the Brønsted acid moiety would still exist and work as an active center by the BBA methodology.

## Results and discussion

### Experimental investigation with chiral *C*_2_-symmetric catalysts

We initially examined the aza-Friedel–Crafts (FC) reaction of 2-methoxyfuran **2** (ref. 12 and 13) with β,γ-alkynyl-α-imino esters **1a**^14^ through the use of achiral Brønsted acid catalysts (5 mol%) in dichloromethane at –78 °C (Table 1 and also see the ESI†). As a result, suitable Brønsted acidity would be required for the reaction to proceed smoothly; catalysts that were too weakly acidic gave poor catalytic activity (entries 1 and 2) and catalysts that were too strongly acidic gave undesired byproducts due to the instability of **1a** and particularly **2** under acidic conditions (entries 4–6). Fortunately, phosphoric acids could be used without the serious generation of byproducts due to their suitable Brønsted acidity for this reaction, although the yields of product **3a** were moderate (entries 7 and 8). Next, we examined conventional chiral phosphoric acid (*R*)-**4a** (entry 9), which would be less aggregatable than the less bulky achiral phosphoric acids in entries 7 and 8. As a result, although the p*K*_a_ of (*R*)-**4a** would be similar to those of the achiral phosphoric acids in entries 7 and 8, the catalytic activity was greatly improved (see the ESI† for details). Particularly, when (*R*)-**4b** with electron-withdrawing CF_3_ groups in its 3,3′-diaryl moieties was used, the reaction was accelerated, although the enantioselectivity was still low (40% ee) (entry 10). Moreover, well-acknowledged bulky (*R*)-**4c** was much less active than (*R*)-**4a** and (*R*)-**4b** (entry 11). In contrast, chiral *C*_2_-symmetric bis(phosphoric acid) (*R*)-**5a**^15^ was much more effective than (*R*)-**4a–c**, and **3a** was obtained in 82% yield with 70% ee within 5 h (entry 12).^16^ Slightly modified catalyst (*R*)-**5b** derived from (*R*)-3,3′-(3,5-(*o*-Tol)_2_C_6_H_3_)_2_-BINOL improved the enantioselectivity (76% ee) of **3a** (entry 13). (*R*)-**5c** with a 5,5′,6,6′,7,7′,8,8′-H_8_-binaphthyl backbone showed lower catalytic activity than (*R*)-**5a** and (*R*)-**5b**, and a prolonged reaction time (24 h) was needed (entry 14). Moreover, we optimized β,γ-alkynyl-α-imino esters **1** with the use of (*R*)-**5b** (see the ESI† for details).^17^ To avoid the effect of adventitious water, which might react with **1** and **2** to give undesired products, powdered MS 5 Å was used as a drying agent. As a result, when we used **1b** with bulky i-Pr_3_Si protection for the alkyne moiety, (*S*)-**3b** was obtained in 83% yield with 91% ee (eqn (1)). Remarkably, we could perform a 1.89 g-scale synthesis of (*S*)-**3b** (77% yield with 91% ee), and 99% of (*R*)-**5b** could be recovered.
1

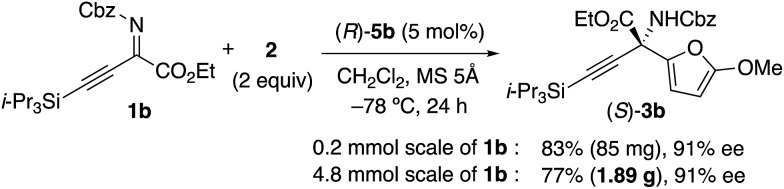




**Table 1:** Screening of Brønsted acid catalysts^*a*^

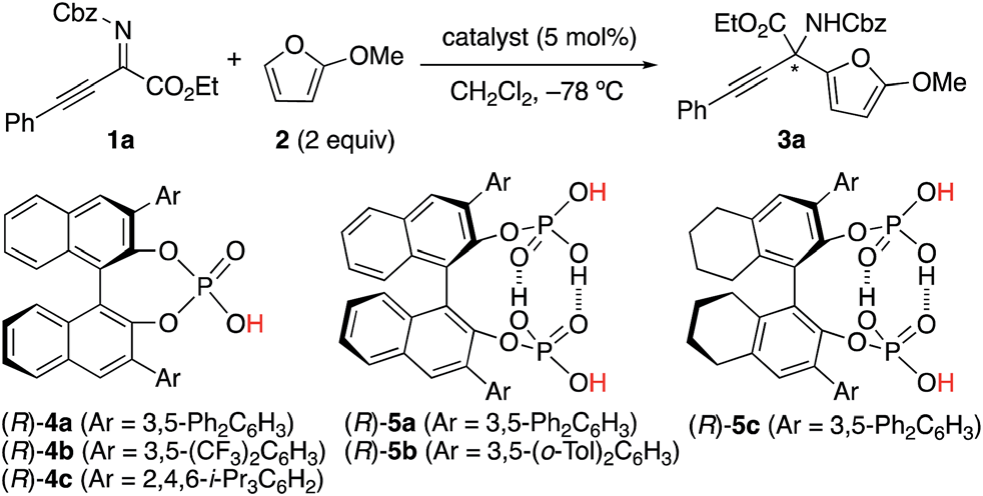							
Entry	Catalyst	p*K*_a_ in H_2_O^*b*^	p*K*_a_ in DMSO^*b*^	Reaction time (h)	Conversion (%) of **1a**	Yield (%) of **3a**	ee (%) of **3a**
1	CH_3_CO_2_H	4.76	12.3	24	0	0	—
2	CH_2_BrCO_2_H	2.86	—	24	13	13	—
3	CHF_2_CO_2_H	1.24	6.45	24	56	52	—
4	CCl_3_CO_2_H	0.65	2.5	24	>99	59	—
5	CF_3_CO_2_H	0.26	3.5	12	>99	53	—
6	*p*-CH_3_C_6_H_4_SO_3_H	–1.34	0.9	12	>99	34	—
7	PhOP(=O)(OH)_2_	1.42	—	24	51	49	—
8	(PhO)_2_P(=O)OH	0.26	3.7	24	60	60	—
9	(*R*)-**4a**	—	—	24	>99	87	26(*S*)
10	(*R*)-**4b**	—	2.63	12	>99	73	40(*S*)
11	(*R*)-**4c**	—	4.22	24	95	60	10(*R*)
12	(*R*)-**5a**	—	—	5	>99	82	70(*S*)
13	(*R*)-**5b**	—	—	8	>99	88	76(*S*)
14	(*R*)-**5c**	—	—	24	>99	85	75(*S*)

^*a*^The reaction was carried out with catalyst (5 mol%), **1a** (0.20 mmol, 1 equiv.), and **2** (2 equiv.) in dichloromethane (0.1 M based on **1a**) at –78 °C. Isolated yield of **3a** is shown. Cbz = CO_2_CH_2_Ph.

^*b*^The p*K*_a_ value in the references. See the ESI for details.

**Fig. 2:**
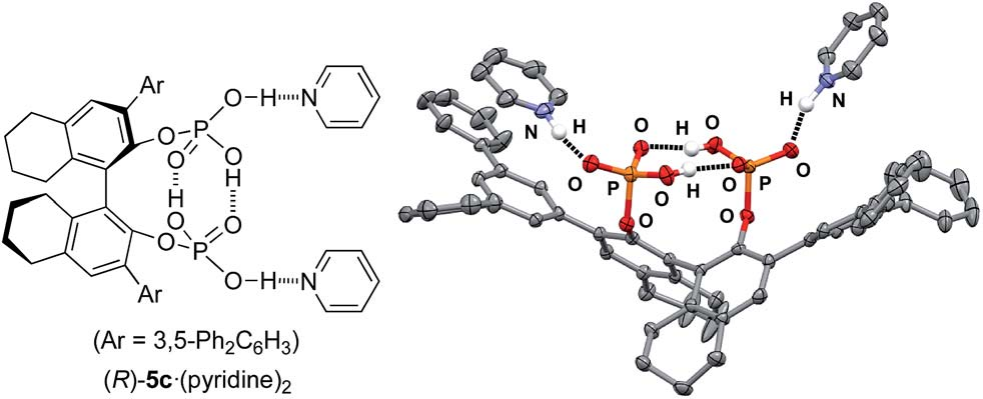
X-ray analysis of (*R*)-**5c**·(pyridine)_2_. Hydrogen atoms are partially omitted for clarity.

**Scheme 1:**
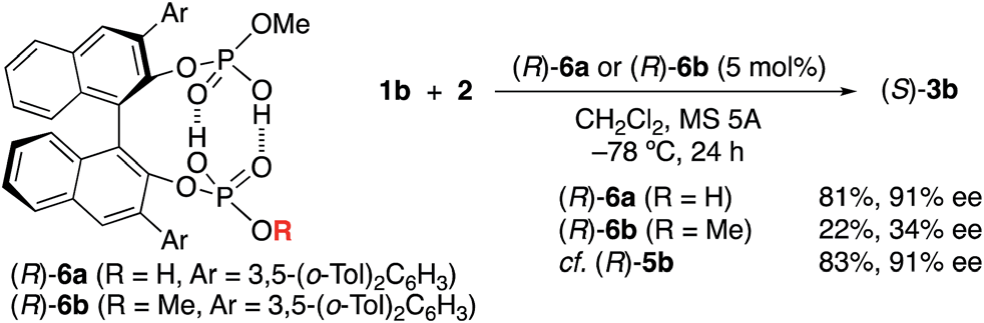
Role of active H^+^-centers in chiral *C*_2_-symmetric catalysts (*R*)-**5b**.

We now turn our attention to mechanistic aspects. It is important to identify the intramolecular double hydrogen bond network in the catalysts. Fortunately, (*R*)-**5c**·(pyridine)_2_ was crystallized, and the results of an X-ray analysis are shown in Fig. 2. As a result, two P(=O)(OH)_2_ moieties at the 2,2′-positions of the H_8_-binaphthyl backbone coordinate with each other, and the conjugated double hydrogen bond network is unambiguously formed at the center of the monomeric molecules. Pyridines are coordinated BBA-activated protons, which would be considered to activate the substrate in a similar way. In this regard, the role of the two outside protons was next examined with the use of monomethyl-protected (*R*)-**6a** and dimethyl-protected (*R*)-**6b** (Scheme 1). As a result, (*R*)-**6a** showed almost the same catalytic activity (81% yield and 91% ee of **3b**) as (*R*)-**5b**, whereas (*R*)-**6b** gave a poor result (22% yield and 34% ee of **3b**) in the reaction of **2** with **1b**. This result strongly suggests that two activated protons in (*R*)-**5b** should be independent of each other, although the absence of both protons results in a loss of catalytic activity. In contrast, the protons in the tight structure of a double hydrogen bond network might not be directly involved in promoting the reaction as possible Brønsted acids.

**Fig. 3:**
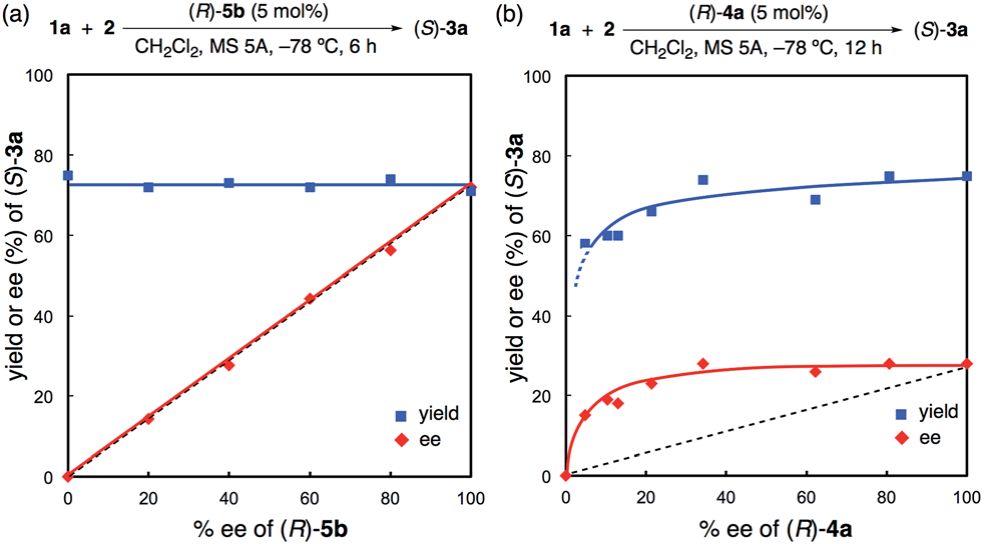
Non-linear effects of (*R*)-**5b** and (*R*)-**4a**.

Moreover, a non-linear effect was examined in the reaction of **2** with **1a** with the use of (*R*)-**5b** or (*R*)-**4a** (Fig. 3 and also see the ESI†). As expected from the X-ray structure of monomeric (*R*)-**5c**·(pyridine)_2_, a linear relationship was observed for (*R*)-**5b**, and the yields were almost constant (71–75%) (Fig. 3a). In contrast, a positive non-linear effect was observed for (*R*)-**4a** (Fig. 3b).^18^ This non-linear relationship strongly suggests that inactive dimeric species^19^ might be involved under the reaction conditions for (*R*)-**4a** unlike (*R*)-**5b**. Indeed, although both (*R*)-**5b** and (*R*)-**4a** have almost the same sterically hindered 3,3′-diaryl moieties, the strongly Brønsted basic P=O moiety is still free in (*R*)-**4a**. It is quite unlike the situation in (*R*)-**5b**, which has a core hydrogen bond network through the “intramolecular dimerization” of two P(=O)(OH)_2_ moieties. Thus, the much better catalytic activity of (*R*)-**5b** compared to (*R*)-**4a** might be attributed to not only the stronger acidity of (*R*)-**5b** due to a BBA system but also the monomeric active species of (*R*)-**5b** due to the closed P=O moieties.^20^

### Experimental investigation with chiral *C*_1_-symmetric catalysts

With the above understanding of the present catalyst system, we should expand the substrate scope of α-ketimino esters beyond the useful but specific substrates **1a** and **1b**. Due to their synthetic importance, we chose aryl α-ketimino esters, such as **7a** (Table 2). Unfortunately, however, (*R*)-**5b** was not effective for the reaction of less reactive **7a**, unlike more reactive **1a** and **1b**, and (*R*)-**8a** was barely obtained with 15% ee at slightly higher temperature (–60 °C) (entry 3). To improve the enantioselectivity, stereocontrol by more bulky substituents at the 3,3′-positions of the *C*_2_-symmetric catalysts (*R*)-**5** should be needed. However, we could not introduce phosphoric acid moieties at the 2,2′-positions of the bulky 3,3′-Ar_2_-BINOL-skeleton (*e.g.*, Ar = 2,4,6-i-Pr_3_C_6_H_2_). Instead, an extremely bulky *C*_1_-symmetric catalyst (*R*)-**9c** with 2,4,6-Cy_3_C_6_H_2_ at the 3-position could be readily prepared as well as (*R*)-**9a** (Ar = 3,5-Ph_2_C_6_H_3_) and (*R*)-**9b** (Ar = 2,4,6-i-Pr_3_C_6_H_2_). However, since two different active Brønsted acid centers would compete, the enantioselectivity was low (11–18% ee), as expected (entries 4–6). Based on the above consideration of *C*_2_-symmetric catalysts (*R*)-**5**, we designed the site-selectively mono-i-Pr-capped catalysts (*R*)-**10**.^21^ As a result, when we use (*R*)-**10c** with extremely bulky 2,4,6-Cy_3_C_6_H_2_ at the 3-position, the reaction proceeded smoothly, and (*R*)-**8a** was obtained in 93% yield with 95% ee (entry 9). The bulkiness of the aryl moiety at the 3-position is important, since slightly less hindered (*R*)-**10b** with 2,4,6-i-Pr_3_C_6_H_2_ was less effective (entry 8), and much less hindered (*R*)-**10a** with 3,5-Ph_2_C_6_H_3_ was entirely ineffective (entry 7). Conventional chiral phosphoric acids, such as (*R*)-**4b** and (*R*)-**4c**, were not effective (also see the ESI† for details): a prolonged reaction time (24 h) was needed and the enantioselectivity was low (entries 1 and 2).

**Table 2:** Optimization of catalysts for the reaction of α-ketimino ester **7a**^*a*^

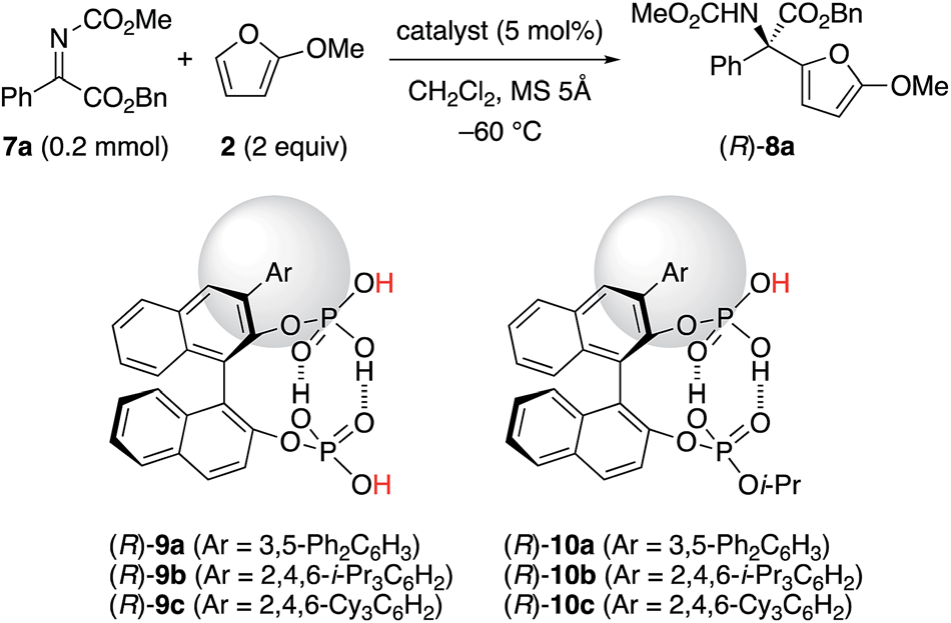				
Entry	Catalyst	Reaction time (h)	Yield (%)	ee (%)
1	(*R*)-**4b** (Ar = 3,5-(CF_3_)_2_C_6_H_3_)	24	83	14
2	(*R*)-**4c** (Ar = 2,4,6-i-Pr_3_C_6_H_2_)	24	79	0
3	(*R*)-**5b** (Ar = 3,5-(*o*-Tol)_2_C_6_H_3_)	12	89	15
4	(*R*)-**9a** (Ar = 3,5-Ph_2_C_6_H_3_)	3	51	11
5	(*R*)-**9b** (Ar = 2,4,6-i-Pr_3_C_6_H_2_)	3	74	13
6	(*R*)-**9c** (Ar = 2,4,6-Cy_3_C_6_H_2_)	3	82	18
7	(*R*)-**10a** (Ar = 3,5-Ph_2_C_6_H_3_)	3	86	36
8	(*R*)-**10b** (Ar = 2,4,6-i-Pr_3_C_6_H_2_)	3	96	91
9	(*R*)-**10c** (Ar = 2,4,6-Cy_3_C_6_H_2_)	3	93	95

^*a*^The reaction was carried out with catalyst (5 mol%), **7a** (0.20 mmol, 1 equiv.), **2** (2 equiv.), and MS 5 Å in dichloromethane (0.1 M based on **7a**) at –60 °C.

**Scheme 2:**
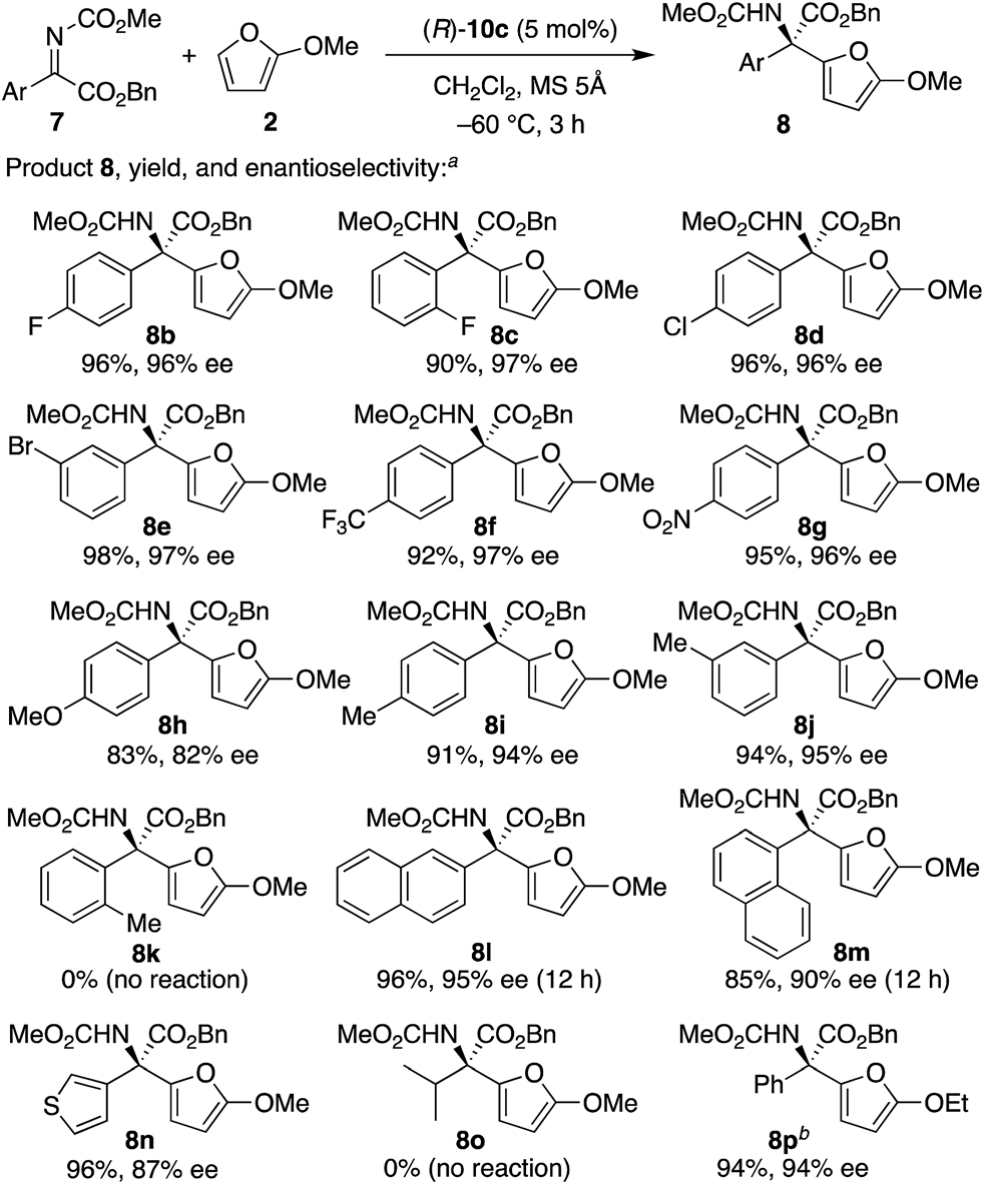
Substrate scope in the enantioselective aza-FC reaction of 2-methoxyfuran **2** with aryl α-ketimino esters **7**. (a) The reaction was carried out with (*R*)-**10c** (5 mol%), **7** (0.20 mmol, 1 equiv.), and **2** (2 equiv.) in dichloromethane (0.1 M based on **7**) at –60 °C for 3 h. (b) 2-Ethoxyfuran was used instead of **2**.

With the optimized catalyst in hand, we next examined the scope of aryl α-ketimino esters **7** (Scheme 2). As a result, not only electron-withdrawing group- but also electron-donating group-substituted aryl substrates could be generally used (see **8b–j**, 83–98% yield with 82–97% ee). When we investigated simple *o*-, *m*-, and *p*-tolyl-substituted substrates (**7i–k**), *p*-tolyl **8i** and *m*-tolyl **8j** were obtained in high yields with high enantioselectivities (94% and 95% ee, respectively), whereas *o*-tolyl **8k** could not be obtained probably due to steric hindrance. Indeed, less sterically hindered *o*-F-C_6_H_4_**8c** and 1-naphthyl **8m** were obtained with high enantioselectivities (97% and 90% ee, respectively). 2-Naphthyl **8l** and heteroaryl **8n** were also obtained successfully (95% and 87% ee, respectively). Unfortunately, however, a low-reactive aliphatic substrate **7o** could not be used, and no reaction proceeded. Moreover, instead of 2-methoxyfuran **2**, 2-ethoxyfuran^22^ could be used, and the corresponding product **8p** was obtained in 94% yield with 94% ee.

To date, it has been difficult to consider the difference between the optimized *C*_2_-symmetric (*R*)-**5b** and *C*_1_-symmetric (*R*)-**10c**. As seen above, (*R*)-**5b**-catalysis of **1b** and **2** provided (*S*)-**3b**, and (*R*)-**10c**-catalysis of **7a** and **2** provided (*R*)-**8a**. Therefore, the observed absolute stereochemistries in **5b** and **8a** were opposite each other. This changeover might be caused by the geometry of α-ketimino esters (*E*)-**1b**^14^ and (*Z*)-**7a**^23^ (see the ESI† for details). Moreover, (*S*)-**3b** was obtained in 14% yield with 6% ee by using (*R*)-**10c** (eqn (2)), whereas (*R*)-**8a** was obtained in 66% yield with 21% ee by using (*R*)-**5b** (eqn (3)). Overall, chiral *C*_2_- and *C*_1_-symmetric bis(phosphoric acid) catalysts were complementary, and either catalyst that was suitable for one reaction would not be suitable for the other reaction from the viewpoint of yield and enantioselectivity. Although preliminary possible transition states are considered as a working model (see the ESI† for details), further investigations of the catalysts are still needed.^16^,^20^

2

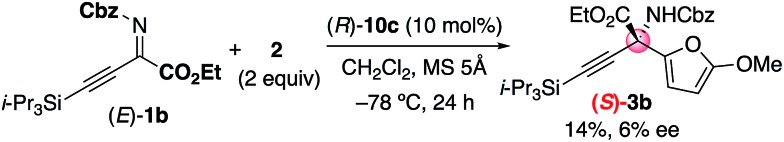



3

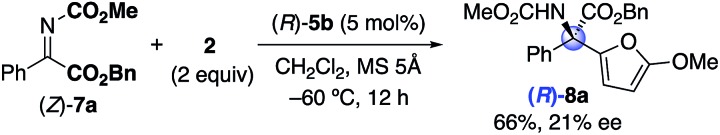




### Transformation of products

**Scheme 3:**
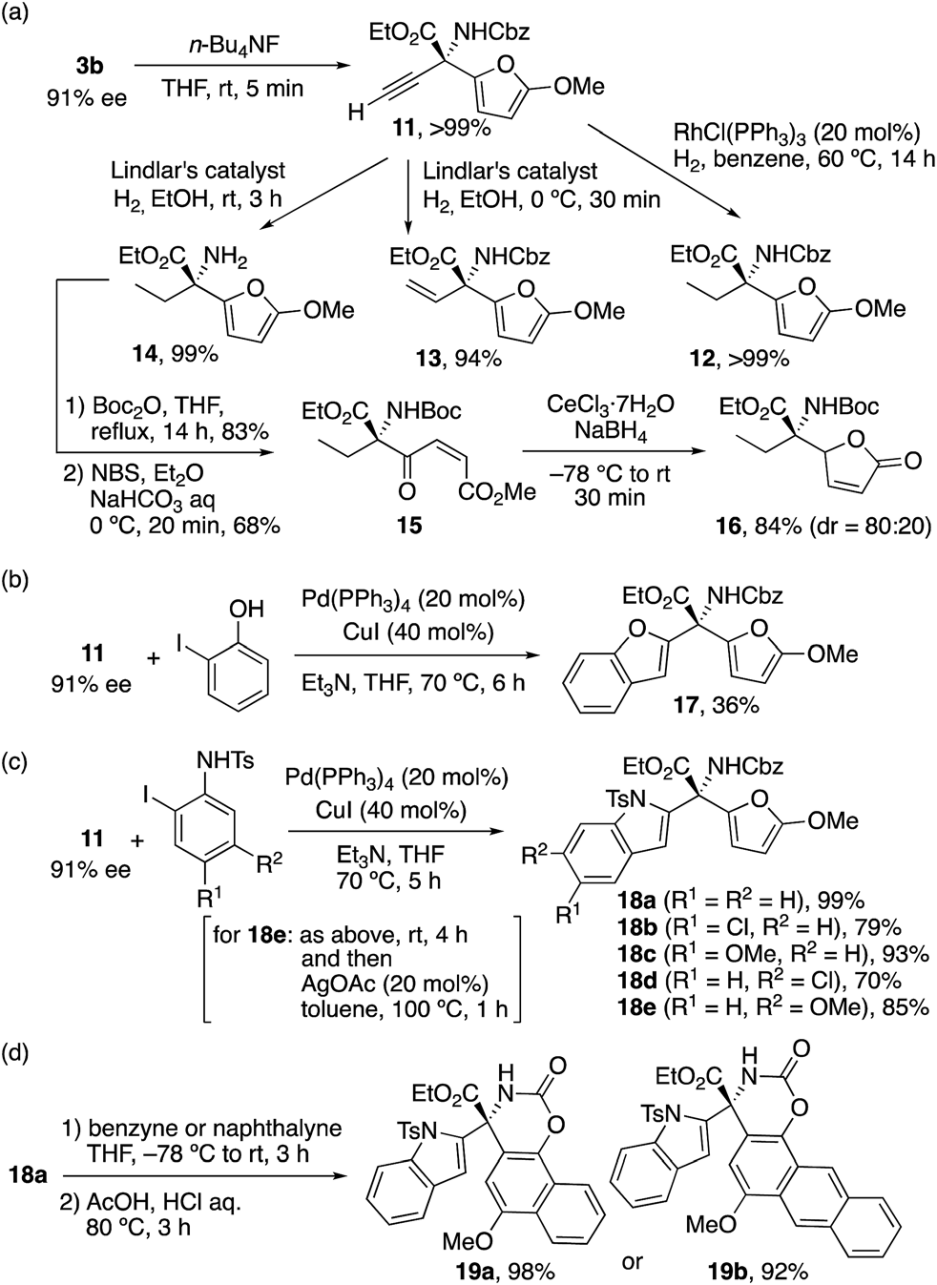
Transformation of alkyne and furan moieties.

Since optically active **3b** has four versatile and transferable functional groups, including acetylene, ester, carbamate, and furyl groups, at the chiral quaternary carbon, we chose to explore the transformations of these functional groups (Scheme 3a). First, the i-Pr_3_Si moiety of **3b** was removed by tetrabutylammonium fluoride (TBAF) to give **11** quantitatively. Next, **11** was reduced under typical reaction conditions. Wilkinson's catalyst completely reduced the acetylene moiety of **11**, and **12** was obtained quantitatively. Lindlar's catalyst facilitated the selective hydrogenation of acetylene at 0 °C to give vinyl compound **13** in 94% yield, whereas both acetylene and the Cbz moieties of **11** were reduced at room temperature and **14** was obtained quantitatively. Furthermore, the NH_2_ moiety of **14** was protected by di-*tert*-butyl dicarbonate (Boc_2_O) in 83% yield, and the corresponding product was consequently treated with *N*-bromosuccinimide (NBS) to give 1,4-dicarbonyl compound **15** in 68% yield *via* furan cleavage. Finally, chemoselective reduction of the keto moiety of **15** with the use of CeCl_3_/NaBH_4_ gave γ-butenolide **16** in 84% yield with a diastereomeric ratio of 80 : 20.^12^

We further explored the transformation of **11** to some optically active N- and O-heterocycles. Sonogashira coupling of **11** with 2-iodophenol proceeded in the presence of Pd(PPh_3_)_4_/CuI catalysts, and novel 2-substituted benzofuran **17**, which has a chiral quaternary carbon center with substitutions of furan, ester, and carbonate moieties, was obtained in 36% yield (Scheme 3b). A similar transformation of **11** with *N*-tosyl-2-iodoaniline proceeded, and novel 2-substituted indol **18a** with those functional groups was obtained quantitatively (Scheme 3c). Cl- or MeO-substituted *N*-tosyl-2-iodoanilines were also tolerable, and **18b–e** were obtained in high yields (70–93%). Moreover, a Diels–Alder reaction of the furan moiety of **18a** with benzyne gave the corresponding adduct, which, without purification, was treated with HCl/acetic acid to give **19a** in 98% yield (Scheme 3d).^24^ Naphthalyne in place of benzyne also gave the corresponding product **19b** in 92% yield. These indole-derived α-amino acid derivatives **18** and **19** with extraordinary structural diversities would facilitate the process of drug discovery.^25^

**Scheme 4:**
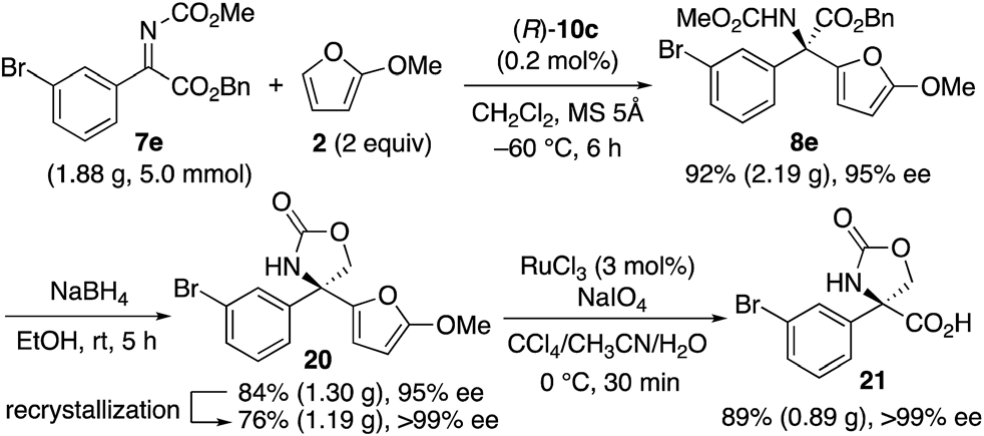
Transformation to optically active α-aryl-substituted serine **21**.

Since optically active α-aryl-substituted serines are synthetically useful,^26^ we finally transformed the obtained product **8e** (Scheme 4). Before the transformation, we performed a >2 g-scale synthesis of **8e** (95% ee) with a catalyst loading as low as 0.2 mol%. The obtained **8e** was then reduced by NaBH_4_, and compound **20** was obtained in 84% yield. Compound **20** was highly crystalline, and a single recrystallization increased the enantiopurity up to >99% ee. Finally, the 2-methoxyfuran moiety of **20** was oxidized by RuCl_3_-catalyzed NaIO_4_-oxidation,^27^ and the corresponding desired optically active α-aryl-substituted serine-derivative **21** (0.89 g) was obtained in 89% yield.

## Conclusions

In summary, we have developed chiral BINOL-derived *C*_2_- and *C*_1_-symmetric bis(phosphoric acid) catalysts. The conjugated double hydrogen bond network was key to increasing the Brønsted acidity and preventing dimerization/deactivation of the catalysts. In particular, we developed a highly enantioselective aza-Friedel–Crafts reaction of 2-methoxyfuran with α-ketimino esters for the first time. By taking advantage of the highly functionalized products, some transformations to versatile N- and O-heterocycles and α-aryl-substituted serine with a chiral quaternary carbon center could be achieved. The further application of these catalysts in other asymmetric catalyses is underway.

## Conflicts of interest

There are no conflicts to declare.

## Supplementary Material

Supplementary information

Crystal structure data
