# Supplementary material for: Enantioselective aza-Friedel–Crafts reaction of furan with α-ketimino esters induced by a conjugated double hydrogen bond network of chiral bis(phosphoric acid) catalysts
Source: Chem Sci. 2018 Jun 25;9(30):6361–7. doi: 10.1039/c8sc02290a (PMC6143995; doi:10.1039/c8sc02290a)
Supplement: Supplementary file 1 [file SC-009-C8SC02290A-s001.pdf]

Electronic Supplementary Material (ESI) for Chemical Science.
This journal is © The Royal Society of Chemistry 2018

Electronic Supplementary Information

Enantioselective Aza-Friedel–Crafts Reaction of Furan with α-Ketimino Esters Induced by a Conjugated Double Hydrogen Bond Network of Chiral Bis(phosphoric Acid) Catalysts

Manabu Hatano, Haruka Okamoto, Taro Kawakami, Kohei Toh, Hidefumi Nakatsuji, Akira Sakakura* and Kazuaki Ishihara*

Table of Contents:

1. General methods. S2
2. Preparation of chiral C_2-symmetric bis(phosphoric acid)s (*R*)-**5** (Method 1). S3
3. Preparation of chiral C_2-symmetric bis(phosphoric acid)s (*R*)-**5** (Method 2). S6
4. X-ray analysis of (*R*)-**5c**•(pyridine)$_2$ (Fig. 2). S9
5. Preparation of chiral C_1-symmetric bis(phosphoric acid)s (*R*)-**9**. S11
6. Preparation of chiral C_1-symmetric bis(phosphoric acid)s (*R*)-**10**. S15
7. Preparation of α-ketimino esters **1**. S19
8. Representative procedures for the enantioselective aza-Friedel–Crafts reaction of **2** with **1** (Table1 and Eq. 1). S20
9. Screening of achiral catalysts in the probe reaction of **2** with **1a**. S22
10. Screening of chiral catalysts in the probe reaction of **2** with **1a**. S23
11. Screening of chiral catalysts in the probe reaction of **2** with **1b**. S24
12. Calculation of the electrostatic potential of phosphoric acids. S25
13. Optimization of the concentration, drying agents, solvents, and substrates in the reaction of **2** with **1**. S31
14. Preparation of (*R*)-**6** (Scheme 1). S33
15. Non-linear effect in the reaction of **2** with **1a** (Fig. 3). S35
16. ESI-MS analysis of catalysts. S37
17. Preparation of α-ketimino esters **7**. S41
18. Representative procedures for the enantioselective aza-Friedel–Crafts reaction of **2** with **7** (Table 2 and Scheme 2). S46
19. Optimization of catalysts, protecting groups on substrates, and reaction temperature in the reaction of **2** with **7a**. S53
20. Preparation of (*R*)-**S32** and the control experiments. S57
21. Transformation of **3b** to **12–14** by selective reduction (Scheme 3a). S59
22. Transformation of **14** to γ-butenolide **16** (Scheme 3a). S61
23. Transformation of **11** to **17** (Scheme 3b). S62
24. Transformation of **11** to **18** (Scheme 3c). S63
25. Transformation of **18a** to **19** (Scheme 3d). S66
26. Gram-scale reaction and transformation to amino acid **21** (Scheme 4). S69
27. Theoretical study on the *E*/*Z*-geometry of substrates **1b** and **7a**. S71
28. Possible transition states for the reactions. S72
29. References. S73

Appendix A: ^{1}H and ^{13}C spectra. S-A1 to S-A67
Appendix B: HPLC spectra. S-B1 to S-B16

1. General methods.

^{1}H NMR spectra were measured on a JEOL ECS400 (400 MHz) spectrometer at ambient temperature. Data were recorded as follows: chemical shift in ppm from internal tetramethylsilane on the δ scale, multiplicity (s = singlet; d = doublet; t = triplet; q = quartet, m = multiplet, br = broad), coupling constant (Hz), integration, and assignment. ^{13}C NMR spectra were measured on a JEOL ECS400 (100 MHz) spectrometer. Chemical shifts were recorded in ppm from the solvent resonance employed as the internal standard (deuterochloroform at 77.10 ppm). ^{19}F NMR spectra were measured on a JEOL ECS-400 (376 MHz) spectrometer. Chemical shifts were recorded in ppm from the solvent resonance employed as the external standard ($CFCl_3$ at 0 ppm). ^{31}P NMR spectra were measured on a JEOL ECS-400 (161 MHz) spectrometer. Chemical shifts were recorded in ppm from the solvent resonance employed as the external standard (H_3PO_4 at 0 ppm). High resolution mass spectral analyses were performed at Chemical Instrument Center, Nagoya University (JEOL JMS-700 (FAB), Bruker Daltonics micrOTOF-QII (ESI)). Infrared (IR) spectra were recorded on a JASCO FT/IR 460 plus spectrometer. *In situ*-IR analysis was performed by Mettler-Toledo ReactIR 15. High performance liquid chromatography (HPLC) analysis was conducted using Shimadzu LC-10 AD coupled diode array-detector SPD-M20A and chiral column of Daicel CHIRALCEL, CHIRALPAK; AD-H, AS-H, OD-H, OD-3, IA-3, IC-3. Optical rotations were measured on Rudolph Autopol IV digital polarimeter. DFT calculation was performed by Spartan’10 for Macintosh from Wavefunction, Inc. X-ray analysis was performed by Rigaku PILATUS-200K. The products were purified by column chromatography on silica gel (E. Merck Art. 9385; Kanto Chemical Co., Inc. 37560). For thin-layer chromatography (TLC) analysis throughout this work, Merck precoated TLC plates (silica gel 60GF254 0.25 mm) were used. Visualization was accomplished by UV light (254 nm), anisaldehyde, $KMnO_4$, and phosphomolybdic acid. In experiments that required dry solvents such as chloroform, dichloromethane, methanol, acetonitrile, etc. were distilled in prior to use. 2-Methoxyfuran is commercially available, and 2-ethoxyfuran was prepared according to the literature procedure.[1]

2. Preparation of chiral C_2-symmetric bis(phosphoric acid)s (*R*)-5 (Method 1).

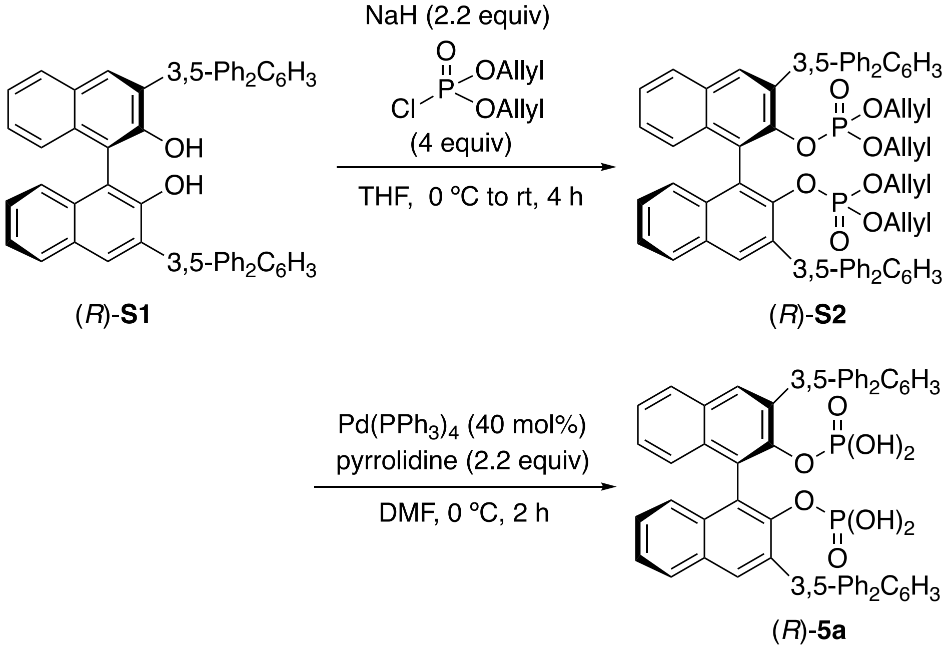

Preparation of (*R*)-S2: A solution of (*R*)-**S1** (4.05 g, 5.45 mmol) and sodium hydride (*ca.* 60%w/w oil dispersion, 480 mg, 12 mmol) in THF (55 mL) was stirred at 0 °C for 30 min under a nitrogen atmosphere. Diallyl chlorophosphate[2] (3.55 mL, 21.7 mmol) was slowly added at 0 °C, and the mixture was stirred at room temperature for 4 h. The resulting mixture was then cooled in ice bath, and diluted with ethyl acetate (40 mL) and water (20 mL). The product was extracted with ethyl acetate (20 mL × 2) and washed with brine (20 mL). The combined extracts were dried over Na_2SO_4. The organic phase was concentrated under reduced pressure, and the crude product was purified by silica gel column chromatography (eluent: *n*-hexane:EtOAc = 5:1 to 3:1) to give the product (*R*)-**S2** as colorless solid (4.05 g, 70% yield). ^{1}H NMR (400 MHz, $CDCl_3$) δ 3.17-3.24 (m, 2H), 3.49-3.60 (m, 4H), 3.73-3.81 (m, 2H), 4.75-4.84 (m, 8H), 5.15-5.25 (m, 2H), 5.26-5.38 (m, 2H), 7.32-7.42 (m, 8H), 7.44-7.50 (m, 10H), 7.73 (d, *J* = 7.3 Hz, 8H), 7.84 (t, *J* = 1.4 Hz, 2H), 7.90 (d, *J* = 8.2 Hz, 2H), 7.92 (d, *J* = 1.4 Hz, 4H), 8.06 (s, 2H). ^{13}C NMR (100 MHz, $CDCl_3$) Many peaks overlapped. δ 67.4, 67.7, 124.7, 125.3, 126.1, 126.9, 127.5, 127.6, 127.7, 128.9, 131.3, 131.6, 132.0, 132.3, 133.7, 134.7, 139.3, 141.1, 145.4. ^{31}P NMR (160 MHz, $CDCl_3$) δ –5.8. HRMS (FAB+) calcd for $C_{68}H_{57}O_8P_2$ $[M+H]^+$ 1063.3529, found 1063.3527.

Preparation of (*R*)-3,3'-di(3,5-terphenyl)-1,1'-binaphthyl-2,2'-bis(phosphoric acid) ((*R*)-5a): Pyrrolidine (690 μL, 8.4 mmol) and tetrakis(triphenylphosphine)palladium (0) (1.76 g, 1.52 mmol) was added to a solution of (*R*)-**S2** (4.05 g, 3.80 mmol) in *N*,*N*-dimethylformamide (38 mL) at 0 °C. The reaction mixture was stirred at room temperature for 4 h. The solution was put onto a column with the anion exchange resin (DOWEX Cl^- form), which was prepared in advance. The sample-mounted resin was washed with THF (300 mL). Then THF and 12 *M* HCl aqueous solution (v/v = 10/1, 500 mL) was passed through the resin, and the filtrate was collected. The

acidic layer was concentrated under reducued pressure, and extracted with dichloromethane (20 mL × 2) and washed with 1 *M* HCl aqueous solution (10 mL). The resulting organic layer was concentrated under reduced pressure. The obtained product was dissolved in toluene (20 mL), and the volatiles were thoroughly removed under reduce pressure. The product was dissolved in dichloromethane (10 mL), and the excess amount of *n*-hexane was added to give of (*R*)-**5a** as a white-yellow powder (3.02 g, 88% yield). A trace amount of Et_2O remained. ^{1}H NMR (400 MHz, THF-d_8) δ 3.50-5.00 (br, 4H), 7.30-7.37 (m, 4H), 7.38-7.51 (m, 12H), 7.57 (d, *J* = 8.2 Hz, 2H), 7.81 (d, *J* = 7.6 Hz, 8H), 7.88 (s, 2H), 8.00-8.04 (m, 6H), 8.23 (s, 2H). ^{13}C NMR (100 MHz, THF-d_8) δ 125.7 (2C), 126.1 (2C), 126.5 (2C), 126.7 (2C), 127.6 (2C), 128.0 (4C), 128.2 (8C), 129.0 (6C), 129.5 (8C), 132.4 (2C), 132.7 (2C), 133.6 (2C), 136.4 (2C), 140.4 (2C), 142.3 (4C), 142.4 (4C), 147.4 (2C) [Contamination of a trace amount of acetone (δ 30.2) through the ^{13}C NMR analysis.]. ^{31}P NMR (160 MHz, THF-d_8) δ –0.13. IR (KBr) 3423, 3058, 2924, 1595, 1400, 1239, 1190, 1151, 1034, 1002 cm^{-1}. $[\alpha]_D^{27}$ = +178.8 (*c* 1.00, THF). M.p. 218 °C (decomposition). HRMS (FAB–) calcd for $C_{56}H_{39}O_8P_2$ $[M–H]^-$ 901.2120, found 901.2138.

Preparation of anion exchange resin: Commercially available DOWEX Cl^- form ion-exchange resin (*ca.* 100 mL) in a column was washed with 1 *M* HCl aqueous solution (200 mL) until the yellow eluate would be colorless. The resin was washed with water (200 mL) until the acidic eluate would be neutral by monitoring with pH test paper. The resin was washed with 1 *M* NaOH aqueous solution (200 mL) and then water (500 mL) until the basic eluate would be neutral by monitoring with pH test paper.

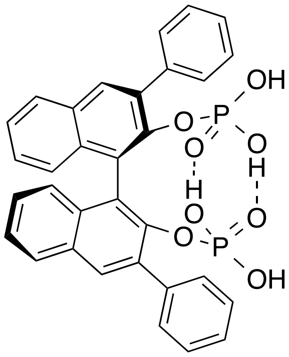

(*R*)-3,3'-Diphenyl-1,1'-binaphthyl-2,2'-bis(phosphoric acid) ((*R*)-S3): Prepared by Method 1. 83% yield for the first step and 97% yield for the second step. A trace amount of *n*-hexane remained. White-yellow soild. ^{1}H NMR (400 MHz, THF-d_8) δ 7.31 (t, *J* = 7.3 Hz, 2H), 7.37-7.41 (m, 6H), 7.47 (t, *J* = 6.9 Hz, 2H), 7.54 (d, *J* = 8.2 Hz, 2H), 7.69 (d, *J* = 6.9 Hz, 4H), 7.97 (d, *J* = 8.2 Hz, 2H), 8.02 (s, 2H) (Four P-OH moieties were not clearly observed.). ^{13}C NMR (100 MHz, THF-d_8) δ 125.5 (2C), 125.9 (2C), 126.2 (2C), 126.9 (2C), 127.5 (2C), 128.1 (4C), 128.6 (2C), 130.6 (4C), 131.6 (2C), 132.2 (2C), 133.0 (2C), 136.3 (2C), 139.1 (2C), 146.9 (2C). ^{31}P NMR (160 MHz, THF-d_8) δ –0.2. IR (KBr) 3448, 3052, 2890, 1496, 1423, 1359, 1246, 1194,

1149, 1033 cm^{-1}. M.p. 171 °C (decomposition). $[\alpha]_D^{27}$ = +174.7 (*c* 1.00, THF). HRMS (FAB–) calcd for $C_{32}H_{23}O_8P_2$ $[M–H]^-$ 597.0874, found 597.0891.

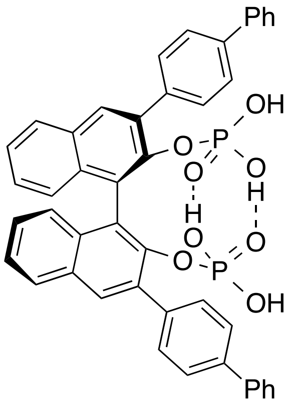

(*R*)-3,3'-Di(4-biphenyl)-1,1'-binaphthyl-2,2'-bis(phosphoric acid) ((*R*)-S4): Prepared by Method 1. 35% yield for the first step and 83% yield for the second step. A trace amount of DMF remained. White-yellow powder. ^{1}H NMR (400 MHz, THF-d_8) δ 4.50-5.02 (br, 4H), 7.31 (t, *J* = 7.3 Hz, 2H), 7.39-7.46 (m, 6H), 7.48 (t, *J* = 6.9 Hz, 2H), 7.58 (d, *J* = 8.2 Hz, 2H), 7.66-7.76 (m, 8H), 7.82 (d, *J* = 8.2 Hz, 4H), 8.00 (d, *J* = 8.2 Hz, 2H), 8.10 (s, 2H). ^{13}C NMR (100 MHz, THF-d_8) δ 125.9 (2C), 126.3 (2C), 126.6 (2C), 127.0 (4C), 127.3 (2C), 127.6 (4C), 127.9 (2C), 128.9 (2C), 129.4 (4C), 131.4 (4C), 132.0 (2C), 132.6 (2C), 133.3 (2C), 136.2 (2C), 138.5 (2C), 140.6 (2C), 141.7 (2C), 147.2 (2C). ^{31}P NMR (160 MHz, THF-d_8) δ 0.1. IR (KBr) 3411, 3029, 1489, 1194, 1034 cm^{-1}. M.p. 206 °C (decomposition). $[\alpha]_D^{27}$ = +177.1 (*c* 1.00, THF). HRMS (FAB–) calcd for $C_{44}H_{31}O_8P_2$ $[M–H]^-$ 749.1494, found 749.1488.

3. Preparation of chiral C_2-symmetric bis(phosphoric acid)s (*R*)-5 (Method 2).

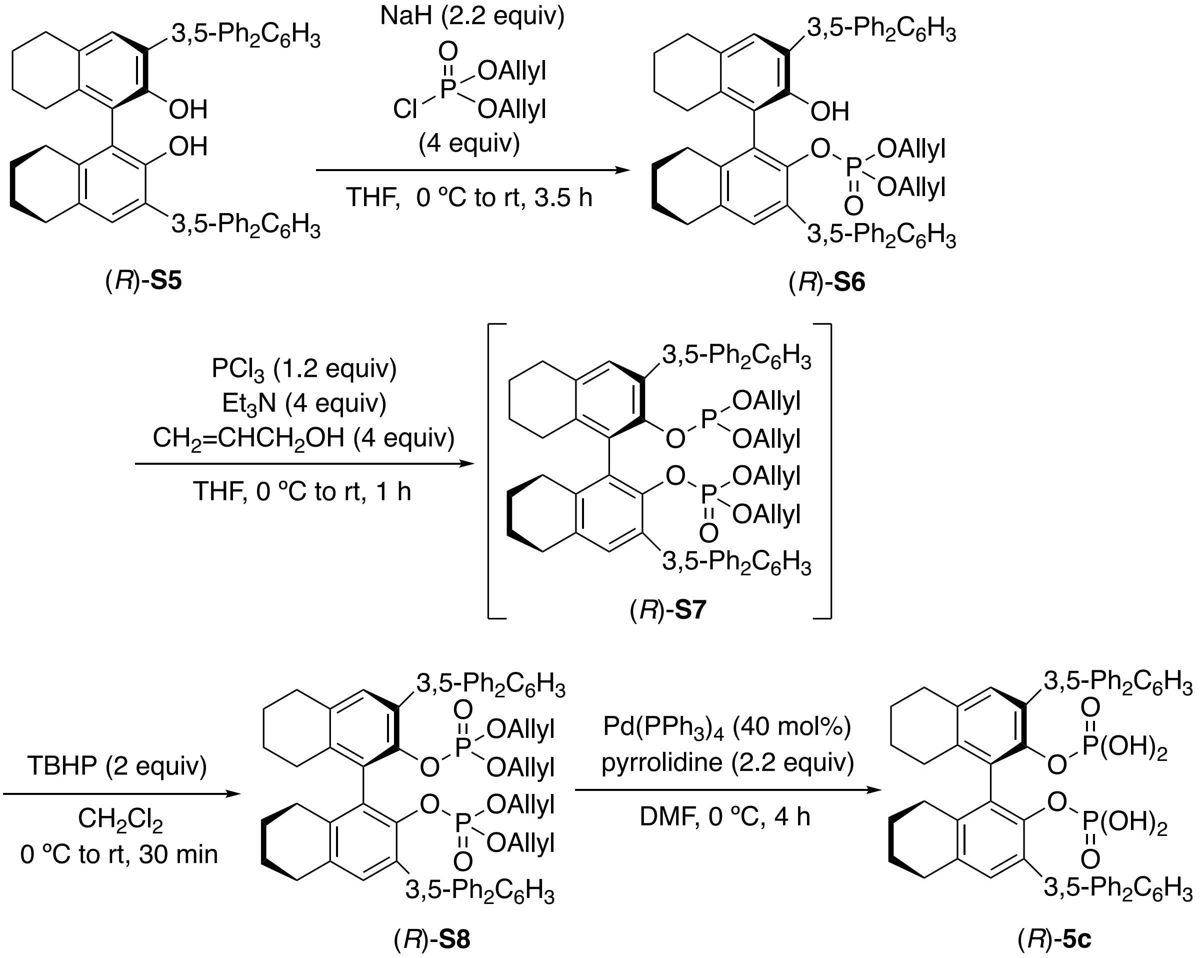

Preparation of (*R*)-S6: A solution of (*R*)-**S5** (2.27 g, 3.02 mmol) and sodium hydride (*ca.* 60%w/w oil dispersion, 266 mg, 6.65 mmol) in THF (30 mL) was stirred at 0 °C for 30 min under a nitrogen atmosphere. Diallyl chlorophosphate (1.97 mL, 12.0 mmol) was slowly added at 0 °C, and the mixture was stirred at room temperature for 4 h. The resulting mixture was then cooled in ice bath, and diluted with ethyl acetate (40 mL) and water (20 mL). The product was extracted with ethyl acetate (20 mL × 2) and washed with brine (20 mL). The combined extracts were dried over Na_2SO_4. The organic phase was concentrated under reduced pressure, and the crude product was purified by silica gel column chromatography (eluent: *n*-hexane:EtOAc = 20:1 to 10:1) to give the product (*R*)-**S6** as coloreless solid (2.39 g, 87% yield). ^{1}H NMR (400 MHz, $CDCl_3$) δ 1.62-1.92 (m, 8H), 2.09-2.23 (m, 2H), 2.51-2.66 (m, 2H), 2.73-2.95 (m, 4H), 3.50-3.59 (m, 1H), 3.64-3.73 (m, 1H), 3.74-3.87 (m, 2H), 4.83-4.90 (m, 2H), 4.99-5.07 (m, 2H), 5.27-5.39 (m, 1H), 5.48-5.59 (m, 1H), 6.61 (s, 1H), 7.22-7.26 (m, 2H), 7.32-7.51 (m, 12H), 7.65-7.72 (m, 8H), 7.72-7.77 (m, 4H), 7.82-7.85 (m, 2H). ^{13}C NMR (100 MHz, $CDCl_3$) Many peaks overlapped. δ 22.7, 23.0, 23.2, 23.4, 27.1, 27.4, 29.6, 29.8, 67.5 (d, $J_{C\text{–}P}$ = 5.7 Hz), 68.2 (d, $J_{C\text{–}P}$ = 5.7 Hz), 117.5, 117.6, 124.7, 125.2, 127.2, 127.4, 127.5, 127.6, 127.7, 128.7, 128.9, 129.8, 130.4, 130.8, 131.0, 131.6, 131.8, 131.9 (d, $J_{C\text{–}P}$ = 7.6 Hz), 132.3 (d, $J_{C\text{–}P}$ = 8.6 Hz), 135.7, 136.0, 138.0,

139.2, 139.7, 141.1, 141.6, 141.8, 144.0 (d, J_{C-P} = 8.6 Hz), 149.0. ^{31}P NMR (160 MHz, $CDCl_3$) δ –3.99. IR (KBr) 3268, 3033, 2927, 2856, 1594, 1497, 1450, 1410, 1250, 1030 cm^{-1}. M.p. 122 °C (decomposition). $[\alpha]_D^{26}$ = –40.7 (*c* 1.00, $CHCl_3$). HRMS (ESI+) calcd for $C_{62}H_{56}O_5P$ $[M+H]^+$ 911.3860, found 911.3860.

Preparation of (*R*)-S7: To a solution of (*R*)-**S6** (2.20 g, 2.41 mmol) and triethylamine (1.34 mL, 9.6 mmol) in THF (6 mL) was added phosphorus trichloride (255 µL, 2.92 mmol) at 0 °C under a nitrogen atmosphere. The mixture was stirred at room temperature for 30 min, and then allyl alcohol (670 µL, 9.85 mmol) was slowly added at 0 °C. The mixture was stirred at room temperature for 1 h. The resulting mixture was diluted with ethyl acetate (40 mL) and water (20 mL). The product was extracted with ethyl acetate (20 mL × 2) and washed with brine (20 mL). The combined extracts were dried over Na_2SO_4. The organic phase was concentrated under reduced pressure to give a coloreless solid mixture of products (*R*)-**S7** and its partially air-oxidized compound (i.e., (*R*)-**S8**), which were used for the next step without the further purification.

Preparation of (*R*)-S8: A solution of the obtained (*R*)-**S7** (and (*R*)-**S8**) (2.41 mmol based on (*R*)-**S6**) in dichloromethane (24 mL) was added *tert*-butyl hydroperoxide (TBHP, *ca.* 5.5 *M* in nonane solution, 870 µL, 4.79 mmol) at 0 °C. The mixture was stirred at room temperature for 30 min. The resulting mixture was diluted with ethyl acetate (40 mL) and saturated $Na_2S_2O_3$ aqueous solution (10 mL). The product was extracted with ethyl acetate (20 mL × 2) and washed by brine (20 mL). The combined extracts were dried over Na_2SO_4. The organic phase was concentrated under reduced pressure, and the crude product was purified by silica gel column chromatography (eluent: *n*-hexane:EtOAc = 5:1 to 2:1) to give the desired product (*R*)-**S8** as coloreless solid (1.41 g, 55% yield based on 2.41 mmol of (*R*)-**S6**). ^{1}H NMR (400 MHz, $CDCl_3$) δ 1.71-1.86 (m, 8H), 2.31-2.45 (m, 2H), 2.70-2.91 (m, 6H), 3.52-3.61 (m, 2H), 3.70-3.88 (m, 6H), 4.83-4.91 (m, 4H), 4.95-5.07 (m, 4H), 5.34-5.45 (m, 2H), 5.48-5.59 (m, 2H), 7.16 (s, 2H), 7.37 (t, *J* = 7.3 Hz, 4H), 7.46 (t, *J* = 7.3 Hz, 8H), 7.69 (d, *J* = 7.3 Hz, 8H), 7.72-7.78 (m, 6H). ^{13}C NMR (100 MHz, $CDCl_3$) δ 22.8 (2C), 23.0 (2C), 27.6 (2C), 29.6 (2C), 67.4 (d, J_{C-P} = 5.7 Hz, 2C), 67.5 (d, J_{C-P} = 5.7 Hz, 2C), 117.2 (2C), 117.5 (2C), 125.0 (2C), 127.4 (8C), 127.5 (4C), 128.0 (4C), 128.9 (8C), 129.1 (2C), 131.9 (2C), 132.1 (2C), 132.4 (d, J_{C-P} = 7.6 Hz, 2C), 132.6 (d, J_{C-P} = 8.5 Hz, 2C), 134.9 (2C), 138.5 (2C), 139.5 (2C), 141.1 (4C), 141.6 (4C), 144.1 (d, J_{C-P} = 7.6 Hz, 2C). ^{31}P NMR (160 MHz, $CDCl_3$) δ –6.12. IR (KBr) 3448, 3059, 3033, 2935, 2860, 1734, 1594, 1498, 1452, 1423, 1409, 1278, 1196, 1031 cm^{-1}. M.p. 238-240 °C. $[\alpha]_D^{27}$ = –87.2 (*c* 1.00, $CHCl_3$). HRMS (ESI+) calcd for $C_{68}H_{65}O_8P_2$ $[M+H]^+$ 1071.4149, found 1071.4138.

Preparation of (*R*)-3,3'-di(3,5-terphenyl)-1,1'-(5,5',6,6',7,7',8,8'-octahydro-binaphthyl)-2,2'-bis(phosphoric acid) ((*R*)-5c): (*R*)-**S8** was used for the next step with the same procedure described above, and (*R*)-**5c** was obtained as a white-yellow powder (0.937 g, 78% yield). A small amount (4%) of inseparable *n*-hexane remained, and the purity of (*R*)-**5c** was 95% (i.e., 74% yield equivalent). ^{1}H NMR (400 MHz, THF-d_8) δ 1.60-1.91 (m, 8H), 2.10-2.20 (m, 2H), 2.75-2.98 (m, 6H), 7.27 (s, 2H), 7.32 (t, J = 7.3 Hz, 4H), 7.44 (t, J = 7.3 Hz, 8H), 7.74-7.85 (m, 14H), 9.01 (br, 4H). ^{13}C NMR (100 MHz, THF-d_8) δ 23.8 (2C), 24.1 (2C), 28.1 (2C), 30.4 (2C), 125.2 (2C), 127.9 (4C), 128.1 (8C), 128.8 (4C), 129.4 (8C), 130.8 (2C), 132.7 (2C), 133.9 (2C), 135.1 (2C), 137.1 (2C), 140.9 (2C), 142.0 (4C), 142.5 (4C), 146.2 (2C). ^{31}P NMR (160 MHz, THF-d_8) δ 0.32. IR (KBr) 3621, 3419, 2928, 2857, 1594, 1497, 1449, 1409, 1035 cm^{-1}. M.p. 194 °C (decomposition). $[\alpha]_D^{23}$ = –99.2 (*c* 1.00, THF). HRMS (FAB–) calcd for $C_{56}H_{47}O_8P_2$ $[M–H]^-$ 909.2746, found 909.2729.

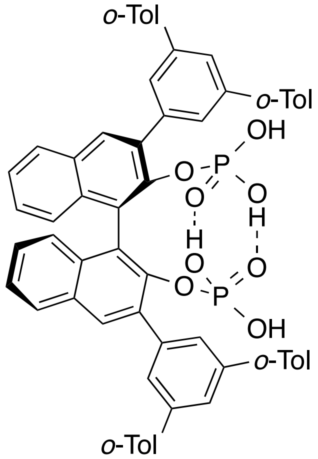

(*R*)-3,3'-Di(3,5-di(*o*-tolyl)phenyl)-1,1'-binaphthyl-2,2'-bis(phosphoric acid) ((*R*)-5b): Prepared by Method 2. A trace amount of *n*-hexane, DMF, and EtOAc remained. A white-yellow powder. ^{1}H NMR (400 MHz, THF-d_8) δ 2.39 (s, 12H), 7.18-7.64 (m, 26H), 7.56 (d, J = 8.7 Hz, 2H), 7.62 (d, J = 1.8 Hz, 4H), 7.98 (d, J = 8.2 Hz, 2H), 8.14 (s, 2H). ^{13}C NMR (100 MHz, THF-d_8) δ 20.9 (4C), 125.8 (2C), 126.4 (6C), 126.6 (2C), 127.5 (2C), 127.9 (4C), 128.9 (2C), 129.7 (2C), 130.6 (8C), 131.0 (4C), 132.0 (2C), 132.6 (2C), 133.5 (2C), 136.4 (4C), 136.8 (2C), 139.3 (2C), 142.2 (4C), 143.0 (4C), 147.4 (2C). ^{31}P NMR (160 MHz, THF-d_8) δ –0.08. IR (KBr) 3057, 2923, 2237, 1594, 1493, 1451, 1399, 1239, 1189, 1151, 1097, 1042 cm^{-1}. M.p. 223 °C (decomposition). $[\alpha]_D^{28}$ = +242.3 (*c* 1.00, $CHCl_3$). HRMS (ESI–) calcd for $C_{60}H_{47}O_8P_2$ $[M–H]^-$ 957.2752, found 952.2756.

4. X-ray analysis of (*R*)-5c•(pyridine)$_2$ (Fig. 2).

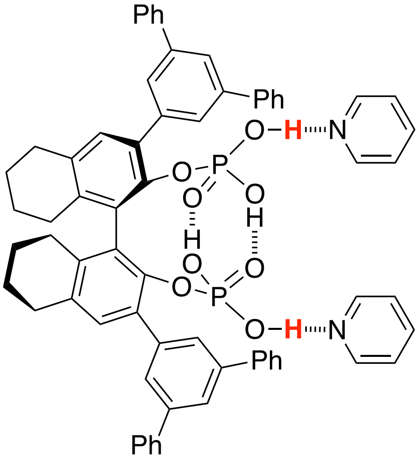

Crystal data of (*R*)-5c·(pyridine)$_2$ (Fig. S1): Compound (*R*)-**5c**·(pyridine)$_2$ was recrystallized in benzene for X-ray analysis. Formula $C_{67}H_{60}Cl_2N_2O_8P_2$, colorless, crystal dimensions 0.20 × 0.20 × 0.20 mm^3, orthorhombic, space group $P2_12_12_1$ (#19), a = 11.4778(15) Å, b = 13.6448(18) Å, c = 36.849(5) Å, α = 90.00 °, β = 90.00 °, γ = 90.00 °, V = 5771.0(13) Å^3, Z = 4, ρ_{calc} = 1.328 g cm^{-3}, F(000) = 2416, μ(MoKα) = 0.228 mm^{-1}, T = 123 K. 39877 reflections collected, 12226 independent reflections with $I > 2\sigma(I)$ ($2\theta_{max}$ = 27.53 °), and 746 parameters were used for the solution of the structure. The non-hydrogen atoms were refined anisotropically. Flack x = –0.001(17). R_1 = 0.0374 and wR_2 = 0.0884. GOF =1.017. Crystallographic data for the structure reported in this paper have been deposited with the Cambridge Crystallographic Data Centre as supplementary publication no. CCDC-1520625. Copies of the data can be obtained free of charge on application to CCDC, 12 Union Road, Cambridge CB2 1EZ, UK [Web page: http://www.ccdc.cam.ac.uk/].

(a) Side view

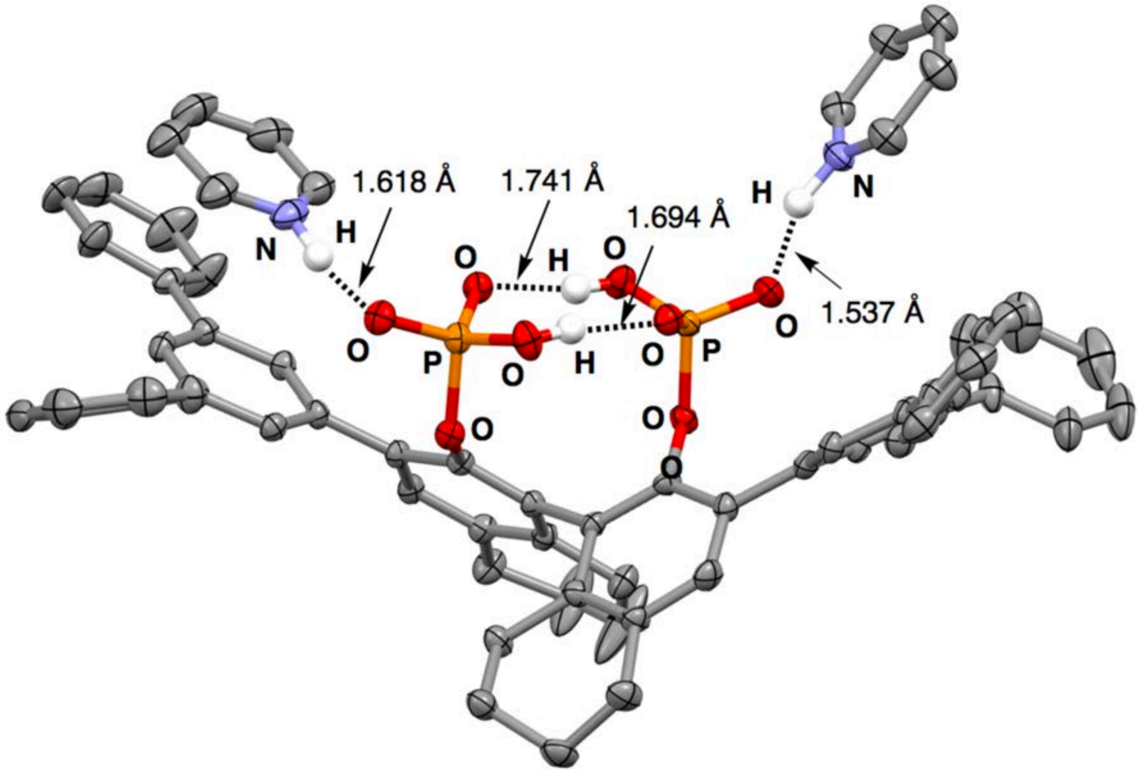

(b) Top view

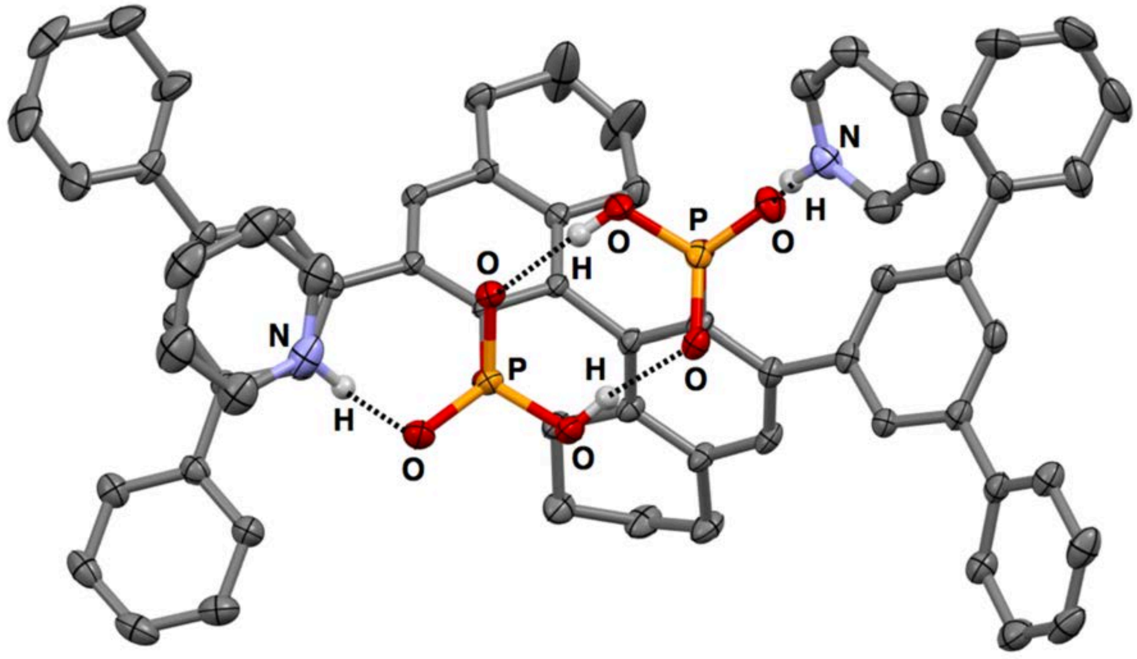

Fig. S1 ORTEP drawing of (*R*)-**5c**•(pyridine)$_2$. (a) Side view. (b) Top view. Some hydrogen atoms were omitted for clarity.

5. Preparation of chiral C_1-symmetric bis(phosphoric acid)s (*R*)-9.

Method A

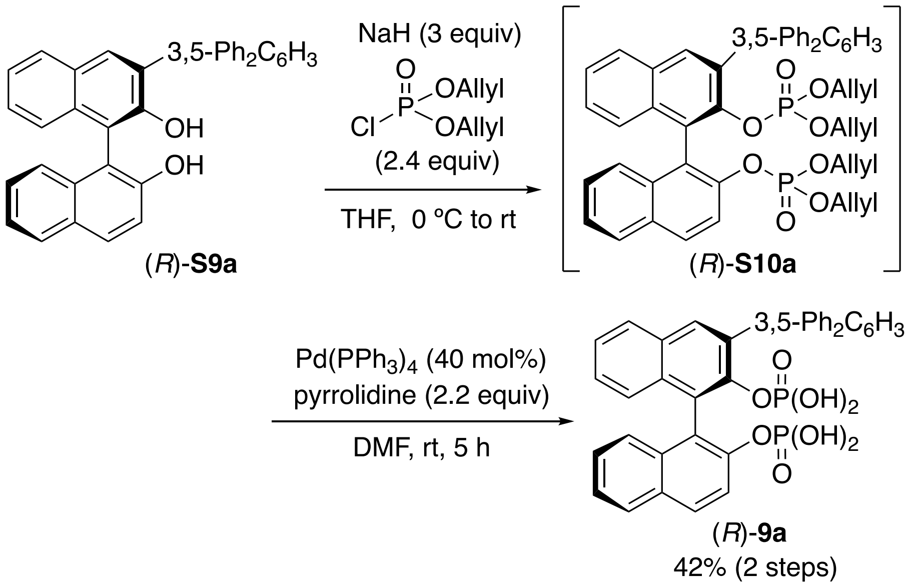

Preparation of (*R*)-S10a: A solution of chiral diol (*R*)-**S9a**[3] (1.67 g, 3.25 mmol) and sodium hydride (*ca.* 60%w/w oil dispersion, 400 mg, 9.9 mmol) in THF (33 mL) was stirred at 0 °C for 3 h under a nitrogen atmosphere. Diallyl chlorophosphate[2] (1.30 mL, 7.9 mmol) was slowly added at 0 °C, and the mixture was stirred at room temperature for 9 h. The resulting mixture was then cooled in ice bath, and diluted with ethyl acetate (20 mL) and water (10 mL). The product was extracted with ethyl acetate (10 mL × 2) and washed with brine (10 mL). The combined extracts were dried over Na_2SO_4. The organic phase was concentrated under reduced pressure, and the crude was roughly purified by short silica gel column chromatography (eluent: *n*-hexane:EtOAc = 5:1). Impure (*R*)-**S10a** was obtained in ca. 75% yield (2.0 g).

Preparation of (*R*)-9a: Pyrrolidine (430 μL, 5.3 mmol) and tetrakis(triphenylphosphine)palladium (0) (1.1 g, 0.96 mmol) was added to a solution of (*R*)-**S10a** (impure, 2.0 g, 2.4 mmol) in *N*,*N*-dimethylformamide (24 mL) at 0 °C. The reaction mixture was stirred at room temperature for 5 h. The solution was put onto a column with the anion exchange resin (DOWEX Cl^- form. See below for the preparation of the resin), which was prepared in advance. The sample-mounted resin was washed with THF (300 mL). Then THF and 12 *M* HCl aqueous solution (v/v = 10/1, 500 mL) was passed through the resin, and the filtrate was collected. The acidic layer was concentrated under reduced pressure, and extracted with dichloromethane (20 mL × 2) and washed with 1 *M* HCl aqueous solution (10 mL). The resulting organic layer was concentrated under reduced pressure. The obtained product was dissolved in toluene (20 mL), and the volatiles were thoroughly removed under reduced pressure. The product was dissolved in dichloromethane (10 mL), and the excess amount of *n*-hexane was added to give pure (*R*)-**9a** as a white-yellow powder (0.924 g, 42% yield in two steps).

(*R*)-3-(3,5-Terphenyl)-1,1'-binaphthyl-2,2'-bis(phosphoric acid) ((*R*)-9a): Light brown solid. ^{1}H NMR (400 MHz, THF-d_8) δ 7.18 (d, J = 8.7 Hz, 1H), 7.23-7.50 (m, 11H), 7.59 (d, J = 8.7 Hz, 1H), 7.80 (d, J = 7.3 Hz, 4H), 7.87 (s, 1H), 7.94 (d, J = 7.8 Hz, 1H), 7.95-8.05 (m, 4H), 8.19 (s, 1H), 9.81 (brs, 4H). ^{13}C NMR (100 MHz, THF-d_8) Peaks are overlapped. δ 122.4, 124.0 (d, J_{C-P} = 5.7 Hz), 125.6, 125.9, 126.4, 126.5, 127.4, 127.6, 128.0, 128.1, 128.9, 129.0, 129.5, 130.6, 132.2, 132.5, 133.9, 134.4, 136.2, 140.3, 142.2, 142.3, 147.2 (d, J_{C-P} = 6.7 Hz), 149.2 (d, J_{C-P} = 5.7 Hz). ^{31}P NMR (160 MHz, THF-d_8) δ –2.07, 1.27. IR (KBr) 3058, 2923, 2851, 2240, 1595, 1404, 1237, 1032, 998 cm^{-1}. M.p. was not available due to decomposition. $[\alpha]_D^{27}$ = +186.8 (*c* 1.00, $CHCl_3$). HRMS (FAB+) calcd for $C_{38}H_{29}O_8P_2$ $[M+H]^+$ 675.1338, found 675.1326.

Method B

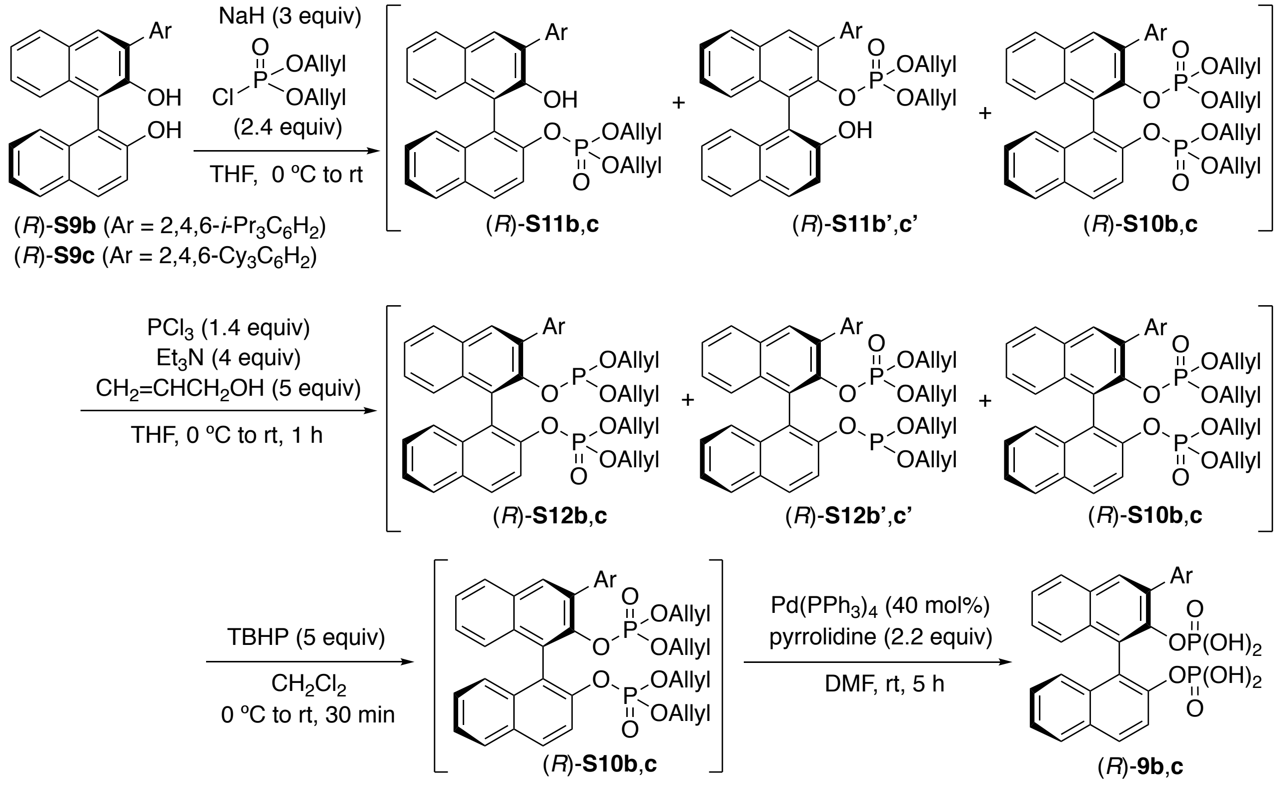

Preparation of (*R*)-S11b, (*R*)-S11b', and (*R*)-S10b (or (*R*)-S11c, (*R*)-S11c', and (*R*)-S10c): A solution of chiral diol (*R*)-**S9b** (or (*R*)-**S9c**) (3.8 mmol) and sodium hydride (*ca.* 60%w/w oil dispersion, 440 mg, 11 mmol) in THF (38 mL) was stirred at 0 °C for 3 h under a nitrogen atmosphere. Diallyl chlorophosphate (1.8 mL, 9.1 mmol) was slowly added at 0 °C, and the mixture was stirred at room temperature for 7 h. The resulting mixture was then cooled in ice bath, and diluted with ethyl acetate (20 mL) and water (10 mL). The product was extracted with ethyl acetate (10 mL × 2) and washed with brine (10 mL). The combined extracts were dried over Na_2SO_4. The organic phase was concentrated under reduced pressure, and the crude product was purified by silica gel column chromatography (eluent: *n*-hexane:EtOAc = 5:1) to give the

mixture of (*R*)-**S11b**, (*R*)-**S11b'**, and (*R*)-**S10b** (or (*R*)-**S11c**, (*R*)-**S11c'**, and (*R*)-**S10c**) which was used for the next step without the further purification.

Preparation of (*R*)-S12b, (*R*)-S12b', and (*R*)-S10b (or (*R*)-S12c, (*R*)-S12c', and (*R*)-S10c): To a solution of the mixture of (*R*)-**S11b**, (*R*)-**S11b'**, and (*R*)-**S10b** (or (*R*)-**S11c**, (*R*)-**S11c'**, and (*R*)-**S10c**) (based on 3.8 mmol of starting (*R*)-**S9b** (or (*R*)-**S9c**)) and triethylamine (2.6 mL, 19 mmol) in THF (38 mL) was added phosphorus trichloride (460 μL, 5.3 mmol) at 0 °C under a nitrogen atmosphere. The mixture was stirred at room temperature for 30 min, and then allyl alcohol (1.6 mL 19 mmol) was slowly added at 0 °C. The mixture was stirred at room temperature for 1 h. The resulting mixture was diluted with ethyl acetate (40 mL) and water (20 mL). The product was extracted with ethyl acetate (20 mL × 2) and washed with brine (20 mL). The combined extracts were dried over Na_2SO_4. The organic phase was concentrated under reduced pressure, and the crude products (*R*)-**S12b**, (*R*)-**S12b'**, and (*R*)-**S10b** (or (*R*)-**S12c**, (*R*)-**S12c'**, and (*R*)-**S10c**) were used for the next step without the further purification.

Preparation of (*R*)-S10b or (*R*)-S10c: A solution of the obtained crude products **S12b**, **S12b'**, and (*R*)-**S10b** (or (*R*)-**S12c**, (*R*)-**S12c'**, and (*R*)-**S10c**) (based on 3.8 mmol of starting (*R*)-**S9b** (or (*R*)-**S9c**)) in dichloromethane (38 mL) was added *tert*-butyl hydroperoxide (TBHP, *ca.* 5.5 *M* in nonane solution, 3.5 mL, 19 mmol) at 0 °C. The mixture was stirred at room temperature for 30 min. The resulting mixture was diluted with ethyl acetate (40 mL) and saturated $Na_2S_2O_3$ aqueous solution (10 mL). The product was extracted with ethyl acetate (20 mL × 2) and washed by brine (20 mL). The combined extracts were dried over Na_2SO_4. The organic phase was concentrated under reduced pressure, and the crude was roughly purified by short silica gel column chromatography (eluent: *n*-hexane:EtOAc = 5:1) to give (*R*)-**S10b** (or (*R*)-**S10c**) (impure).

Preparation of (*R*)-9b or (*R*)-9c: (*R*)-**9b** and (*R*)-**9c** were prepared by the similar procedure for (*R*)-**9a** described above.

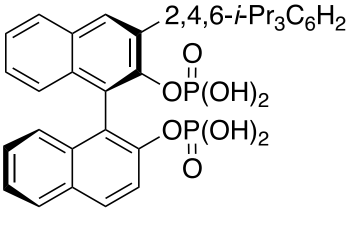

(*R*)-3-(2,4,6-Triisopropylphenyl)-1,1'-binaphthyl-2,2'-bis(phosphoric acid) ((*R*)-9b): Prepared by **Method B** (Typically 45–55% yield in four steps from (*R*)-**S9b**). Light brown solid. ^{1}H NMR (400 MHz, THF-d_8) δ 1.00 (d, *J* = 6.9 Hz, 3H), 1.23 (d, *J* = 6.9 Hz, 3H), 1.25 (d, *J* = 6.9 Hz, 3H), 1.27 (d, *J* = 6.9 Hz, 9H), 2.84 (septet, *J* = 6.9 Hz, 1H), 2.91 (septet, *J* = 6.9 Hz, 1H), 2.97 (septet, *J* = 6.9 Hz, 1H), 7.03 (d, *J* = 1.4 Hz, 1H), 7.13 (d, *J* = 1.4 Hz, 1H), 7.17 (d, *J* = 8.3 Hz, 1H), 7.20 (d, *J* = 8.7 Hz, 1H), 7.22-7.31 (m, 2H), 7.34-7.45 (m, 2H), 7.61 (d, *J* = 9.2 Hz, 1H), 7.80 (brs,

4H), 7.84-7.95 (m, 3H), 7.98 (d, J = 9.2 Hz, 1H). ^{13}C NMR (100 MHz, THF-d_8) Peaks are overlapped. δ 23.6, 23.8, 24.5, 24.6, 25.5, 27.4, 31.9, 32.0, 35.4, 120.7, 121.7, 122.6, 123.7 (d, J_{C-P} = 6.7 Hz), 125.4 (d, J_{C-P} = 3.8 Hz), 125.7, 126.3, 126.7, 127.0, 128.7, 128.8, 130.4, 132.0, 132.2, 132.5, 133.1, 133.9, 134.5, 134.7, 148.2, 148.3 (d, J_{C-P} = 6.7 Hz), 148.9, 149.0, 149.3 (d, J_{C-P} = 5.7 Hz). ^{31}P NMR (160 MHz, THF-d_8) δ –3.42, 0.78. IR (KBr) 3462, 2961, 2869, 1603, 1509, 1466, 1415, 1362, 1210, 1001 cm^{-1}. M.p. was not available due to decomposition. $[\alpha]_D^{24}$ = +257.2 (c 1.00, CHCl$_3$). HRMS (FAB+) calcd for $C_{35}H_{39}O_8P_2$ [M+H]$^+$ 649.2120, found 649.2119.

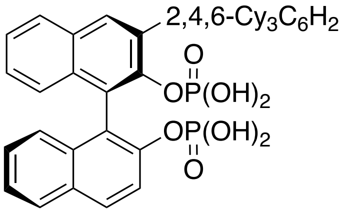

(*R*)-3-(2,4,6-Tricyclohexylphenyl)-1,1'-binaphthyl-2,2'-bis(phosphoric acid) ((*R*)-9c): Prepared by **Method B** (Typically 25–35% yield in four steps from (*R*)-**S9c**). Light brown solid. ^{1}H NMR (400 MHz, THF-d_8) δ 1.01-2.00 (m, 28H), 2.11 (d, J = 12.4 Hz, 2H), 2.38-2.61 (m, 3H), 6.90 (br, 4H), 6.99 (s, 1H), 7.07 (s, 1H), 7.15 (d, J = 8.2 Hz, 1H), 7.21-7.34 (m, 3H), 7.36-7.48 (m, 2H), 7.57 (d, J = 9.2 Hz, 1H), 7.83 (s, 1H), 7.88 (d, J = 8.2 Hz, 1H), 7.93 (d, J = 8.3 Hz, 1H), 8.00 (d, J = 9.2 Hz, 1H). ^{13}C NMR (100 MHz, THF-d_8) Peaks are overlapped. δ 27.2, 27.3, 27.5, 27.8, 28.0, 28.2, 33.4, 34.1, 35.5, 35.6, 36.5, 38.3, 42.7, 42.9, 45.9, 122.0, 122.8, 123.2, 124.0 (d, J_{C-P} = 6.7 Hz), 125.2 (d, J_{C-P} = 4.8 Hz), 125.8, 126.2, 126.5, 126.7, 127.0, 127.3, 128.7, 128.9, 130.3, 131.9, 132.2, 132.9, 133.5, 133.9, 134.5, 147.0, 147.7, 148.2, 148.3 (d, J_{C-P} = 6.7 Hz), 149.3 (d, J_{C-P} = 6.7 Hz). ^{31}P NMR (160 MHz, THF-d_8) δ –2.83, 1.64. IR (KBr) 3457, 2925, 2850, 1602, 1448, 1228, 1148, 1035, 1001 cm^{-1}. M.p. was not available due to decomposition. $[\alpha]_D^{25}$ = +213.6 (c 1.00, CHCl$_3$). HRMS (FAB+) calcd for $C_{44}H_{51}O_8P_2$ [M+H]$^+$ 769.3059, found 769.3081.

6. Preparation of chiral C_1-symmetric bis(phosphoric acid)s (*R*)-10.

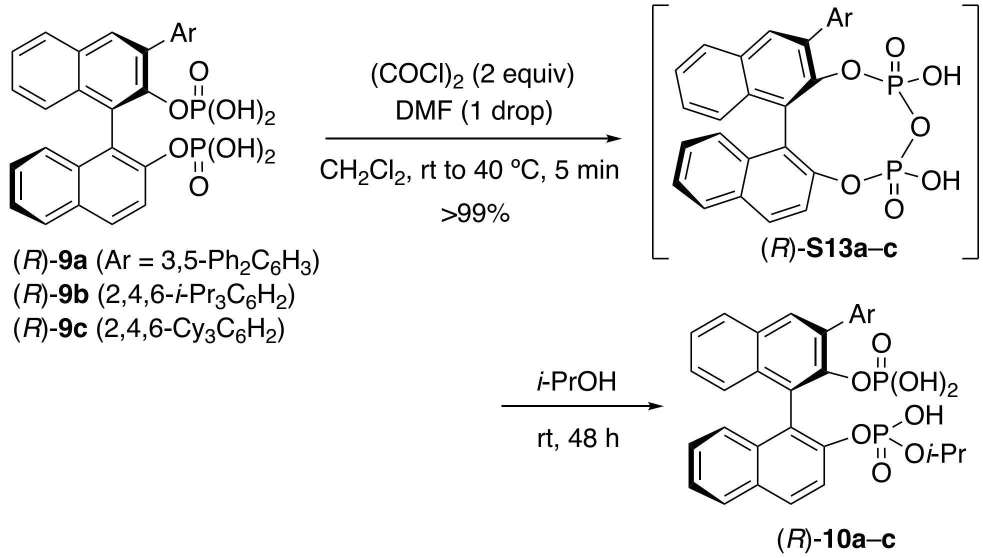

To a solution of (*R*)-**9** (0.030 mmol) in dichloromethane (2 mL), one drop of *N*,*N*-dimethylformamide was added at room temperature. Then oxalyl chloride (5.1 μL, 0.060 mmol) was added at room temperature, and the mixture was warmed to 40 °C. The mixture was stirred at 40 °C for 5 min. Toluene (2 mL) was then added to the resulting mixture, and the volatiles were removed *in vacuo* under heat conditions (ca. 40–50 °C). The obtained product (*R*)-**S13** was used in the next step without further purification. Compound (*R*)-**S13** was dissolved in 2-propanol (2 mL) and the solution was stirred at room temperature for 48 h. 2-Propanol was then removed *in vacuo*. (*R*)-**10** was separated by silica gel column chromatography (eluent: $CHCl_3$:MeOH = 9:1 to 4:1). The isolated (*R*)-**10** was dissolved in chloroform (10 mL), and washed with 1 *M* HCl aqueous solution (10 mL). The resulting organic layer was concentrated under reduced pressure. The obtained compound was dissolved in toluene (2 mL), and the volatiles were thoroughly removed under reduced pressure to give pure (*R*)-**10** as light brown solid.

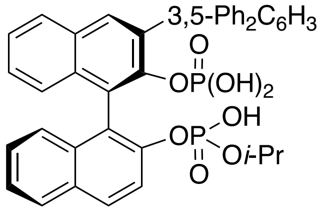

(1*R*)-3-([1,1':3',1''-Terphenyl]-5'-yl)-2'-((hydroxy(isopropoxy)phosphoryl)oxy)-[1,1'-binaphthalen]-2-yl dihydrogen phosphate ((*R*)-10a): 17% yield in 2 steps. Light brown solid. ^{1}H NMR (400 MHz, THF-d_8) δ 0.99 (d, J = 6.0 Hz, 3H), 1.13 (d, J = 6.4 Hz, 3H), 4.39 (m, 1H), 7.03 (br, 3H), 7.24 (d, J = 8.7 Hz, 1H), 7.27-7.37 (m, 4H), 7.38-7.49 (m, 7H), 7.72-7.83 (m, 5H), 7.85-7.91 (m, 1H), 7.92-8.06 (m, 5H), 8.19 (s, 1H). ^{13}C NMR (100 MHz, THF-d_8) Peaks are overlapped. δ 23.5 (d, $J_{C–P}$ = 4.8 Hz), 23.7 (d, $J_{C–P}$ = 5.7 Hz), 73.6 (d, $J_{C–P}$ = 5.7 Hz), 121.4, 123.6 (d, $J_{C–P}$ = 7.6 Hz), 125.6, 125.8, 126.5, 126.6, 127.4, 127.5, 128.0, 128.1, 128.9, 129.5, 130.8,

132.2, 132.5, 133.9, 134.5, 136.0, 140.4, 142.3, 142.4, 146.9 (d, $J_{C–P}$ = 6.7 Hz), 149.1 (d, $J_{C–P}$ = 5.7 Hz). ^{31}P NMR (160 MHz, THF-d_8) δ –3.37, –3.04. IR (KBr) 3450, 3059, 2925, 2853, 1595, 1498, 1467, 1405, 1237, 1149, 1001 cm^{-1}. M.p. was not available due to decomposition. $[\alpha]_D^{29}$ = +184.3 (*c* 1.00, $CHCl_3$). HRMS (FAB+) calcd for $C_{41}H_{35}O_8P_2$ $[M+H]^+$ 717.1807, found 717.1789.

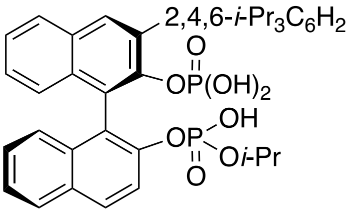

(*R*)-2'-((Hydroxy(isopropoxy)phosphoryl)oxy)-3-(2,4,6-triisopropylphenyl)-[1,1'-binaphthalen]-2-yl dihydrogen phosphate ((*R*)-10b): 50% yield in 2 steps. Light brown solid. ^{1}H NMR (400 MHz, THF-d_8) δ 0.70 (d, *J* = 6.8 Hz, 3H), 1.03 (d, *J* = 6.9 Hz, 3H), 1.08 (d, *J* = 6.9 Hz, 3H), 1.19-1.35 (m, 15H), 2.74-2.99 (m, 3H), 4.20 (m, 1H), 6.72 (br, 3H), 7.05 (d, *J* = 1.4 Hz, 1H), 7.12 (d, *J* = 1.4 Hz, 1H), 7.22-7.49 (m, 6H), 7.83-7.96 (m, 4H), 7.99 (d, *J* = 9.2 Hz, 1H). ^{13}C NMR (100 MHz, THF-d_8) Peaks are overlapped. δ 23.1 (d, $J_{C–P}$ = 4.8 Hz), 23.6, 23.8, 24.5, 27.0, 31.9, 32.0, 35.3, 72.8 (d, $J_{C–P}$ = 6.7 Hz), 120.7, 121.2, 121.6, 122.9 (d, $J_{C–P}$ = 8.6 Hz), 125.3, 125.6, 126.3, 126.4, 126.8, 127.1, 127.2, 128.7, 128.8, 130.7, 132.1, 132.2, 132.4, 133.2, 133.9, 134.4, 134.5, 147.9 (d, $J_{C–P}$ = 7.6 Hz), 148.1, 148.8, 148.9, 149.1 (d, $J_{C–P}$ = 5.7 Hz). ^{31}P NMR (160 MHz, THF-d_8) δ –4.96, –4.88. IR (KBr) 3451, 2959, 2927, 2869, 1626, 1467, 1415, 1362, 1230, 1033, 999 cm^{-1}. M.p. was not available due to decomposition. $[\alpha]_D^{28}$ = +254.3 (*c* 1.00, $CHCl_3$). HRMS (FAB+) calcd for $C_{38}H_{45}O_8P_2$ $[M+H]^+$ 691.2590, found 691.2599.

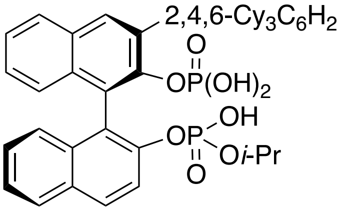

(*R*)-2'-((Hydroxy(isopropoxy)phosphoryl)oxy)-3-(2,4,6-tricyclohexylphenyl)-[1,1'-binaphthalen]-2-yl dihydrogen phosphate ((*R*)-10c): 58% yield in 2 steps. Light brown solid. ^{1}H NMR (400 MHz, THF-d_8) δ 0.97-1.80 (m, 23H), 1.03 (d, *J* = 6.4 Hz, 3H), 1.07 (d, *J* = 6.4 Hz, 3H), 1.80-1.96 (m, 5H), 1.98-2.13 (m, 2H), 2.39-2.57 (m, 3H), 4.41 (m, 1H), 7.00 (d, *J* = 1.4 Hz, 1H), 7.08 (d, *J* = 1.4 Hz, 1H), 7.22 (d, *J* = 8.2 Hz, 1H), 7.24-7.35 (m, 3H), 7.35-7.45 (m, 2H), 7.82 (d, *J* = 9.2 Hz, 1H), 7.83 (s, 1H), 7.88 (d, *J* = 8.3 Hz, 1H), 7.94 (d, *J* = 8.2 Hz, 1H), 8.01 (d, *J* = 9.2 Hz, 1H), 9.54 (br, 3H). ^{13}C NMR (100 MHz, $CDCl_3$) Peaks are overlapped. δ 23.5 (d, $J_{C–P}$ = 4.8 Hz), 23.7 (d, J_{C-P} = 4.8 Hz), 27.1, 27,2, 27.4, 27.8, 28.0, 28.1, 33.7, 33.9, 35.5, 35.6, 36.3, 38.2, 42.7, 45.9, 73.4 (d, $J_{C–P}$ = 5.7 Hz), 121.7, 122.0, 123.1, 123.3 (d, $J_{C–P}$ = 6.7 Hz), 125.1 (d, $J_{C–P}$ =

3.8 Hz), 125.7, 126.3, 126.6, 126.7, 127.2, 127.3, 128.7, 129.0, 130.6, 132.0, 132.2, 132.7, 133.5, 133.9, 134.3, 134.6, 147.0, 147.8, 147.9 (d, $J_{C\text{–}P}$ = 6.7 Hz), 148.1, 149.2 (d, $J_{C\text{–}P}$ = 5.7 Hz). ^{31}P NMR (160 MHz, DMSO-d_6) δ –4.04, –2.88. IR (KBr) 3448, 2925, 2850, 1626, 1448, 1231, 1147, 1033, 999 cm^{-1}. M.p. was not available due to decomposition. $[\alpha]_D^{26}$ = 193.6 (*c* 1.00, $CHCl_3$). HRMS (FAB+) calcd for $C_{47}H_{57}O_8P_2$ $[M+H]^+$ 811.3529, found 811.3523.

We tried to crystallize (*R*)-**10c**, but we could not obtain a suitable crystalfor X-ray analysis. Instead, we obtained a suitable crystal of *racemic*-**10c**. By X-ray analysis, we unambiguously confirmed the structure of **10c** (Fig. S3), in particular, the position of the *i*-Pr moiety. The other possible isomer (*R*)-**10c'** was not obtained in our preparation, probably due to steric constraints of the bulky aryl moiety at the 3-position of the binaphthyl of (*R*)-**S13c** (Fig. S2). *Racemic*-**10c** does not have a conjugated intramolecular double hydrogen bond network as seen for (*R*)-**5c**•(pyridine)$_2$ in Fig. S1. Indeed, *racemic*-**10c** formed a dimer due to an intermolecular hydrogen bond network as shown in Fig. S4. The dimer structure is symmetric: one is (*R*)-**10c** and the other is (*S*)-**10c**. Intramolecular hydrogen bonds are not observed among dimers. (*R*)-**10c** and (*S*)-**10c** have one intramolecular hydrogen bond (1.696 Å), respectively, and four hydrogen bonds (1.576–1.848 Å) are observed between (*R*)-**10c** and (*S*)-**10c**. This dimerization of *racemic*-**10c** might disturb the formation of a conjugated intramolecular double hydrogen bond network. To date, it is still unclear whether or not optically pure (*R*)-**10c** would form a dimer. However, by the ESI-MS analysis of (*R*)-**10c** (Fig. S7f), (*R*)-**10c** might be a monomer as a major species.

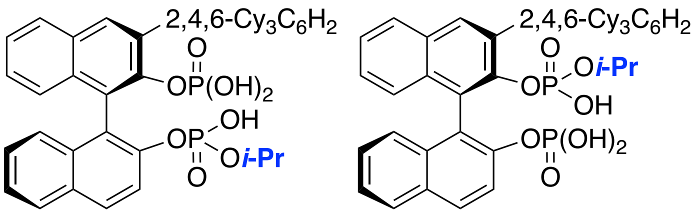

Fig. S2 (*R*)-**10c** and a possible isomer (*R*)-**10c'**. (*R*)-**10c'** was not obtained in our preparation.

Crystal data of *racemic*-10c (Figs. S3 and S4): *Racemic*-**10c** was recrystallized in chloroform–*n*-hexane for X-ray analysis. Formula $C_{47}H_{56}O_8P_2$, colorless, crystal dimensions 0.15 × 0.13 × 0.12 mm^3, monoclinic, space group *P–1* (#2), a = 12.7831(18) Å, b = 14.6459(16) Å, c = 14.800(2) Å, α = 116.231(11) °, β = 101.896(13) °, γ = 102.26(2) °, V = 2282.1(6) Å^3, Z = 2, ρ_{calc} = 1.180 g cm^{-3}, F(000) = 864, μ(MoKα) = 0.145 mm^{-1}, T = 93 K. 19527 reflections collected, 9800 independent reflections with $I > 2\sigma(I)$ ($2\theta_{max}$ = 27.55 °), and 532 parameters were used for the solution of the structure. The non-hydrogen atoms were refined anisotropically. R_1 = 0.0477 and wR_2 = 0.1472. GOF = 1.091. Crystallographic data for the structure reported in this paper have been deposited with the Cambridge Crystallographic Data Centre as supplementary publication no. CCDC- 1834632.

Copies of the data can be obtained free of charge on application to CCDC, 12 Union Road, Cambridge CB2 1EZ, UK [Web page: http://www.ccdc.cam.ac.uk/].

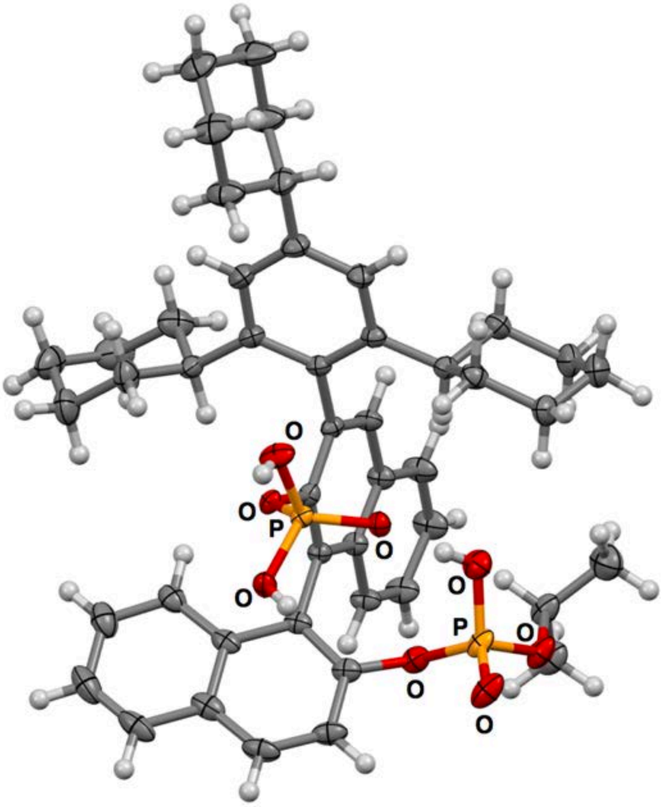

Fig. S3 ORTEP drawing of *racemic*-**10c**.

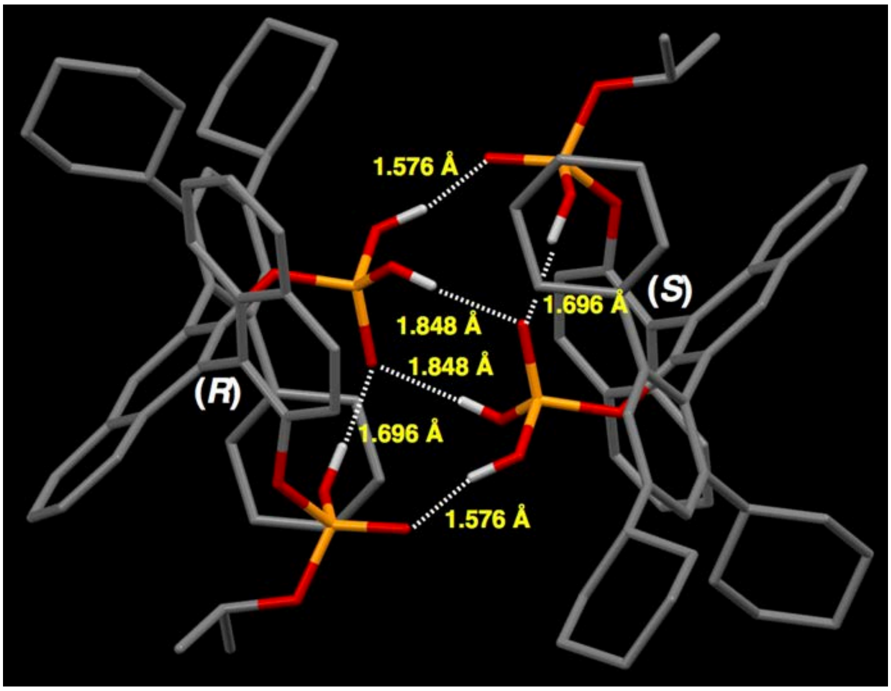

Fig. S4 Dimer structure of *racemic*-**10c** (*R*/*S*-pair).

7. Preparation of α-ketimino esters 1.

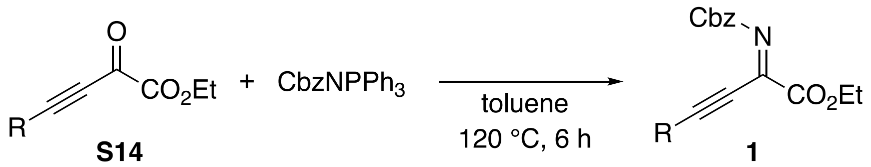

β,γ-Alkynyl-α-imino esters 1: Ketoesters **S14** were prepared based on the reported procedures.[4–6] Compounds **1** were prepared on the basis of a literature procedure.[7] To a well-dried round bottom two necks flask (50 mL) with ketoester **S14** (5.0 mmol) and *N*-Cbz-triphenyliminophosphorane[8] (2.06 g, 5.0 mmol) was added toluene (10 mL). The mixture was heated to 120 °C and stirred for 6 h. After cooling to room temperature, volatiles were removed under reduced pressure. The resultant residue was purified by MPLC (eluent: *n*-hexane:EtOAc = typically 100:0 to 80:20) to give the desired product **1**.

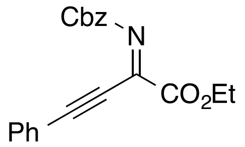

Ethyl 2-(((benzyloxy)carbonyl)imino)-4-phenylbut-3-ynoate (1a): 37% yield. Yellow-orange oil. ^{1}H NMR (400 MHz, $CDCl_3$) δ 1.41 (t, *J* = 6.9 Hz, 3H), 4.42 (q, *J* = 6.9 Hz, 2H), 5.35 (s, 2H), 7.27-7.50 (m, 10H). ^{13}C NMR (100 MHz, $CDCl_3$) δ 14.0, 63.4, 68.8, 81.0, 102.5, 119.4, 128.5 (7C) 131.1, 132.9 (2C), 134.9, 146.0, 160.7, 161.1. IR (neat) 3456, 2983, 2198, 1733, 1615, 1490, 1444, 1373, 1216, 1101, 1021 cm^{-1}. HRMS (FAB+) calcd for $C_{20}H_{18}NO_4$ $[M+H]^+$ 336.1236, found 336.1241.

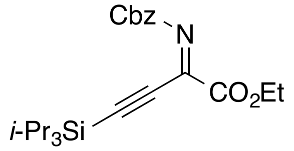

Ethyl 2-(((benzyloxy)carbonyl)imino)-4-(triisopropylsilyl)but-3-ynoate (1b): 78% yield. Light yellow oil. ^{1}H NMR (400 MHz, $CDCl_3$) δ 1.04-1.15 (m, 21H), 1.37 (t, *J* = 6.9 Hz, 3H), 4.37 (q, *J* = 6.9 Hz, 2H), 5.27 (s, 2H), 7.33-7.42 (m, 5H). ^{13}C NMR (100 MHz, $CDCl_3$) δ 10.8 (3C), 13.8, 18.3 (6C), 63.1, 68.8, 96.5, 108.5, 128.3 (2C), 128.4 (3C), 134.5, 145.4, 160.1, 160.7. IR (neat) 3461, 2944, 2866, 2152, 1748, 1618, 1462, 1370, 1220, 1132, 1021 cm^{-1}. HRMS (FAB+) calcd for $C_{23}H_{34}NO_4Si$ $[M+H]^+$ 416.2257, found 416.2265.

8. Representative procedures for the enantioselective aza-Friedel–Crafts reaction of 2 with 1 (Table 1 and Eq. 1).

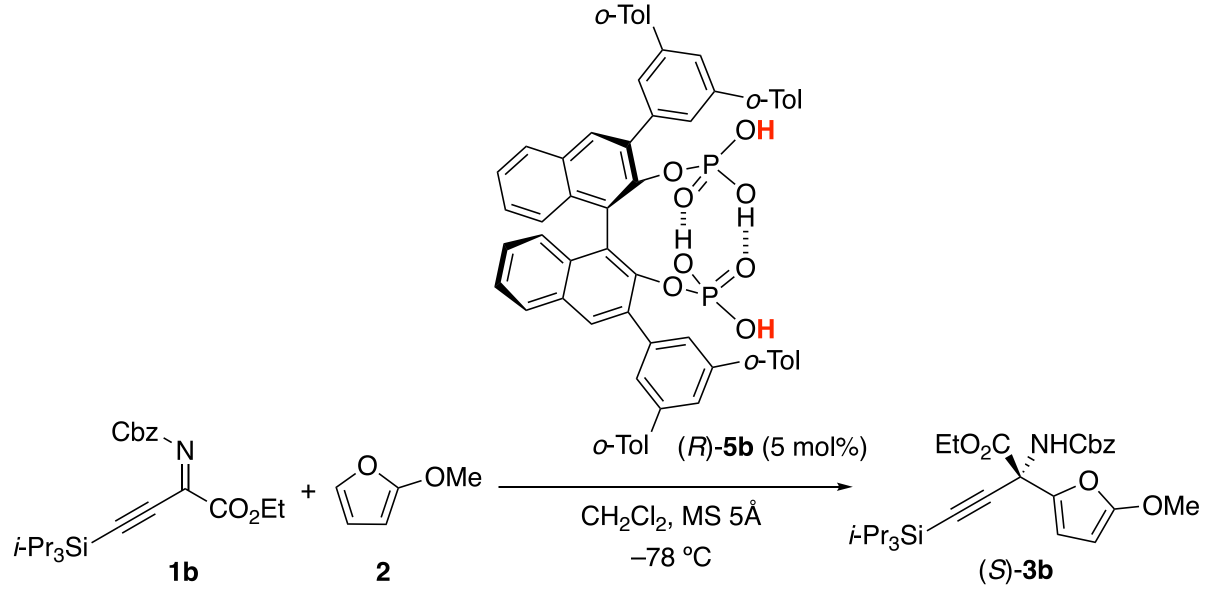

0.20 mmol scale: 85.3 mg, 83%, 91% ee (24 h)
4.80 mmol scale: 1.89 g, 77%, 91% ee (36 h)

Standard conditions (0.20 mmol scale): To a well-dried pyrex Schlenk tube charged with activated MS 5Å (50 mg) under a nitrogen atmosphere were added catalyst (*R*)-**5b** (9.6 mg, 0.010 mmol) in dichloromethane (1.0 mL) and α-ketimino ester **1b** (83 mg, 0.20 mmol). The mixture was diluted with dichloromethane (1.0 mL) and cooled to –78 °C. 2-Methoxyfuran **2** (37 μL, 0.40 mmol) was added and the mixture was stirred at –78 °C for 24 h. To quench the reaction, silica gel (4 mL) was added to the mixture at –78 °C. The resultant silica gel was thoroughly washed with *n*-hexane and ethyl acetate (3:1, 200 mL) at room temperature. The filtrate was concentrated under reduced pressure, and the resultant residue was purified by silica gel column chromatography (eluent: *n*-hexane:EtOAc = 5:1 to 3:1) to give the product **3b** (85.3 mg, 83% yield). The catalyst could be recovered as some metal (Li, Na, K, Ca, etc.) salts of (*R*)-**5b** through the same silica gel column chromatography (eluent: $CHCl_3$:MeOH = 3:1) quantitatively. When the catalyst would be reused for the catalysis, the further purification with washing by 1 *M* HCl aqueous solution is necessary (>99% recovery). The enantiomeric purity of **3b** was determined by chiral HPLC analysis (91% ee).

Gram-scale synthesis: To a well-dried pyrex Schlenk tube charged with activated MS 5Å (50 mg) under a nitrogen atmosphere were added catalyst (*R*)-**5b** (230 mg, 0.24 mmol) in dichloromethane (14 mL) and α-ketimino ester **1b** (2.00 g, 4.8 mmol). The mixture was diluted with dichloromethane (34 mL) and cooled to –78 °C. 2-Methoxyfuran **2** (880 μL, 9.6 mmol) was added and the mixture was stirred at –78 °C for 36 h. To quench the reaction, silica gel (100 mL) was added to the mixture at –78 °C. The resultant silica gel was thoroughly washed with

n-hexane and ethyl acetate (3:1, 400 mL) at room temperature. The filtrate was concentrated under reduced pressure, and the resultant residue was purified by silica gel column chromatography (eluent: *n*-hexane:EtOAc = 5:1 to 3:1) to give the product **3b** (1.89 g, 77% yield). The catalyst could be recovered as some metal salts of (*R*)-**5b** through the same silica gel column chromatography (eluent: $CHCl_3$:MeOH = 3:1) quantitatively. When the catalyst would be reused for the catalysis, the further purification with washing by 1 *M* HCl aqueous solution is necessary (>99% recovery). The enantiomeric purity of **3b** was determined by chiral HPLC analysis (91% ee).

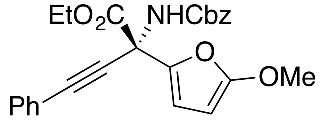

Ethyl (*S*)-2-(((benzyloxy)carbonyl)amino)-2-(5-methoxyfuran-2-yl)-4-phenylbut-3-ynoate (3a): 88% yield, 76% ee. Colorless oil. ^{1}H NMR (400 MHz, $CDCl_3$) δ 1.26 (t, *J* = 6.8 Hz, 3H), 3.82 (s, 3H), 4.22-4.38 (m, 2H), 5.09 (d, *J* = 12.1 Hz, 1H), 5.14 (d, *J* = 12.1 Hz, 1H), 5.15 (d, *J* = 3.6 Hz, 1H), 6.21 (br, 1H), 6.60 (br, 1H), 7.28-7.39 (m, 8H), 7.46 (br, 2H). ^{13}C NMR (100 MHz, $CDCl_3$) δ 14.0, 56.6, 57.8, 63.6, 67.0, 80.8, 83.6, 84.6, 111.7, 122.1, 128.1 (3C), 128.2 (2C), 128.5 (2C), 128.8, 132.1 (2C), 136.2, 138.5, 154.0, 161.6, 167.0. IR (neat) 3409, 2979, 2936, 2906, 1734, 1615, 1576, 1490, 1369, 1260, 1022 cm^{-1}. $[\alpha]_D^{26}$ = –2.8 (*c* 1.00, $CHCl_3$, 76% ee (*S*)). HPLC analysis; AD-H, *n*-hexane/*i*-PrOH = 1/1, 254 nm, 1.0 mL/min, t_R = 15.9 min (major, *S*), 32.4 min (minor, *R*). HRMS (FAB+) calcd for $C_{25}H_{23}NNaO_6$ $[M+Na]^+$ 456.1423, found 456.1418.

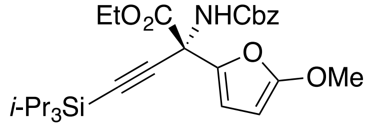

Ethyl (*S*)-2-(((benzyloxy)carbonyl)amino)-2-(5-methoxyfuran-2-yl)-4-(triisopropylsilyl)but -3-ynoate (3b): 83% yield, 91% ee. Pale yellow oil. ^{1}H NMR (400 MHz, $CDCl_3$) δ 1.07 (s, 21H), 1.23 (t, *J* = 6.9 Hz, 3H), 3.80 (s, 3H), 4.15 (br, 1H), 4.31 (br, 1H), 5.09 (d, *J* = 12.4 Hz, 1H), 5.12 (d, *J* = 2.8 Hz, 1H), 5.14 (d, *J* = 12.4 Hz, 1H), 6.11 (br, 1H), 6.54 (br, 1H), 7.25-7.38 (m, 5H). ^{13}C NMR (100 MHz, $CDCl_3$) δ 11.1 (3C), 13.9, 18.6 (6C), 56.5, 57.8, 63.4, 66.8, 80.8, 86.3, 100.7, 111.8, 128.0 (3C), 128.4 (2C), 136.4, 138.7, 153.6, 161.4, 166.9. IR (neat) 3418, 2943, 2176, 1746, 1614, 1574, 1470, 1369, 1257, 1019 cm^{-1}. $[\alpha]_D^{24}$ = –2.8 (*c* 1.00, $CHCl_3$, 91% ee (*S*)). HPLC analysis; AD-H, *n*-hexane/*i*-PrOH = 9/1, 254 nm, 0.6 mL/min, t_R = 10.3 min (major, *S*), 12.0 min (minor, *R*). HRMS (FAB+) calcd for $C_{28}H_{39}NNaO_6Si$ $[M+Na]^+$ 536.2444, found 536.2437.

9. Screening of achiral catalysts in the probe reaction of 2 with 1a.

The screening of achiral catalysts in the probe reaction of **2** with **1a** is summarized in Table S1. The pK_a values of the acid compounds used here are generally available in the literature. The desired product **3a** was obtained in better yield and chemoselectivity by catalysts, particularly some carboxylic acids such as CHF_2CO_2H and CCl_3CO_2H (entries 3 and 4), with pK_a values of 2.5–6.5 in DMSO and 0.65–1.24 in H_2O. The pK_a range was comparable to the pK_a values of phosphoric acids (entries 10 and 11).

Table S1 Screening of the achiral catalysts in the probe reaction of **2** with **1a**.

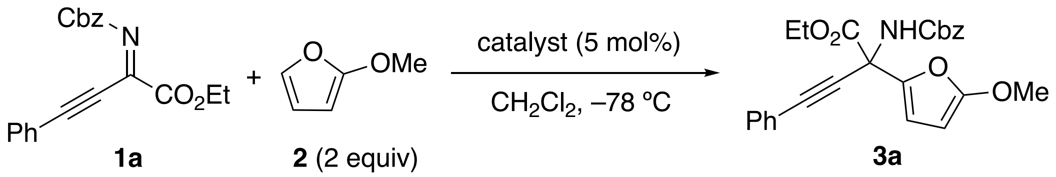

Entry	Catalyst	pK_a (calcd)[a]	pK_a in H_2O[b]	pK_a in DMSO	Reaction time (h)	Conversion (%)	Yield (%)
1	CH_3CO_2H	4.79	4.76	12.3[c]	24	0	0
2	CH_2BrCO_2H	2.73	2.86	–	24	13	13
3	CHF_2CO_2H	1.32	1.24	6.45[d]	24	56	52
4	CCl_3CO_2H	0.09	0.65	2.5[e]	24	>99	59
5	CF_3CO_2H	0.05	0.26	3.5[c]	12	>99	53
6	HCl[f]	–	–8.00	1.8[c]	3	>99	59
7	*p*-$CH_3C_6H_4SO_3H$	–0.43	–1.34	0.9[g]	12	>99	34
8	CF_3SO_3H	–3.91	–13.0	0.3[c]	6	>99	0
9	$(CF_3SO_2)_2NH$	–10.42	–	2.4[h]	3	>99	0
10	**PhOP(=O)(OH)$_2$**	1.25	1.42[i]	–	24	**51**	**49**
11	**(PhO)$_2$P(=O)OH**	1.12	0.26[j]	3.7[k]	24	**60**	**60**

[a] Data in SciFinder. Calculated using ACD/Labs Software V11.02. [b] D. Gryko, M. Zimnicka and R. Lipiński, *J. Org. Chem.*, 2007, **72**, 964. [c] F. G. Bordwell, *Acc. Chem. Res.*, 1988, **21**, 456. [d] C. D. Ritchie and S. Lu, *J. Am. Chem. Soc.*, 1990, **112**, 7748. [e] H.-s. Kim, T. D. Chung and H. Kim, *J. Electroanal. Chem.*, 2001, **498**, 209. [f] 1 *M* HCl in diethyl ether was used as HCl source. [g] H. Kim, J. Gao and D. Burgessa, *J. Int. J. Pharm.*, 2009, **377**, 105. [h] C. Yang, X.-S. Xue, X. Li and J.-P. Cheng, *J. Org. Chem.*, 2014, **79**, 4340. [i] D. Shamir, I. Zilbermann, E. Maimon, A. I. Shames, H. Cohen and D. Meyerstein, *Inorg. Chim. Acta*, 2010, **363**, 2819. [j] F. Krašovec and J. Jan, *Croat. Chem. Acta*, 1963, **35**, 183. [k] P. Christ, A. G. Lindsay, S. S. Vormittag, J.-M. Neudröfl, A. Berkessel and A. M. C. O'Donoghue, *Chem. Eur. J.*, 2011, **17**, 8524.

10. Screening of chiral catalysts in the probe reaction of 2 with 1a.

Screening of chiral catalysts in the probe reaction of **2** with **1a** is summarized in Scheme S1. The catalytic activities of conventional chiral phosphoric acids, such as (*R*)-**S15**,[9] (*R*)-**4a**,[10] and (*R*)-**4b**,[11] and (*R*)-**4c**[12] were moderate, but the enatio-induction were low. In contrast, bis(phosphoric acid)s (*R*)-**5a** and (*R*)-**5b** showed higher catalytic reactivities with high enantioselectivities, although sterically less hindered (*R*)-**S3** and (*R*)-**S4** showed low catalytic activities due to their low solubility. Less acidic (*R*)-**5c** did not provide better reactivity than (*R*)-**5a** or (*R*)-**5b**. Highly acidic chiral 3,3'-Ar$_2$-BINSA (*R*)-**S16**[13] gave a poor result, since significant amounts of byproducts were obtained due to the strong acidity of (*R*)-**S16**.

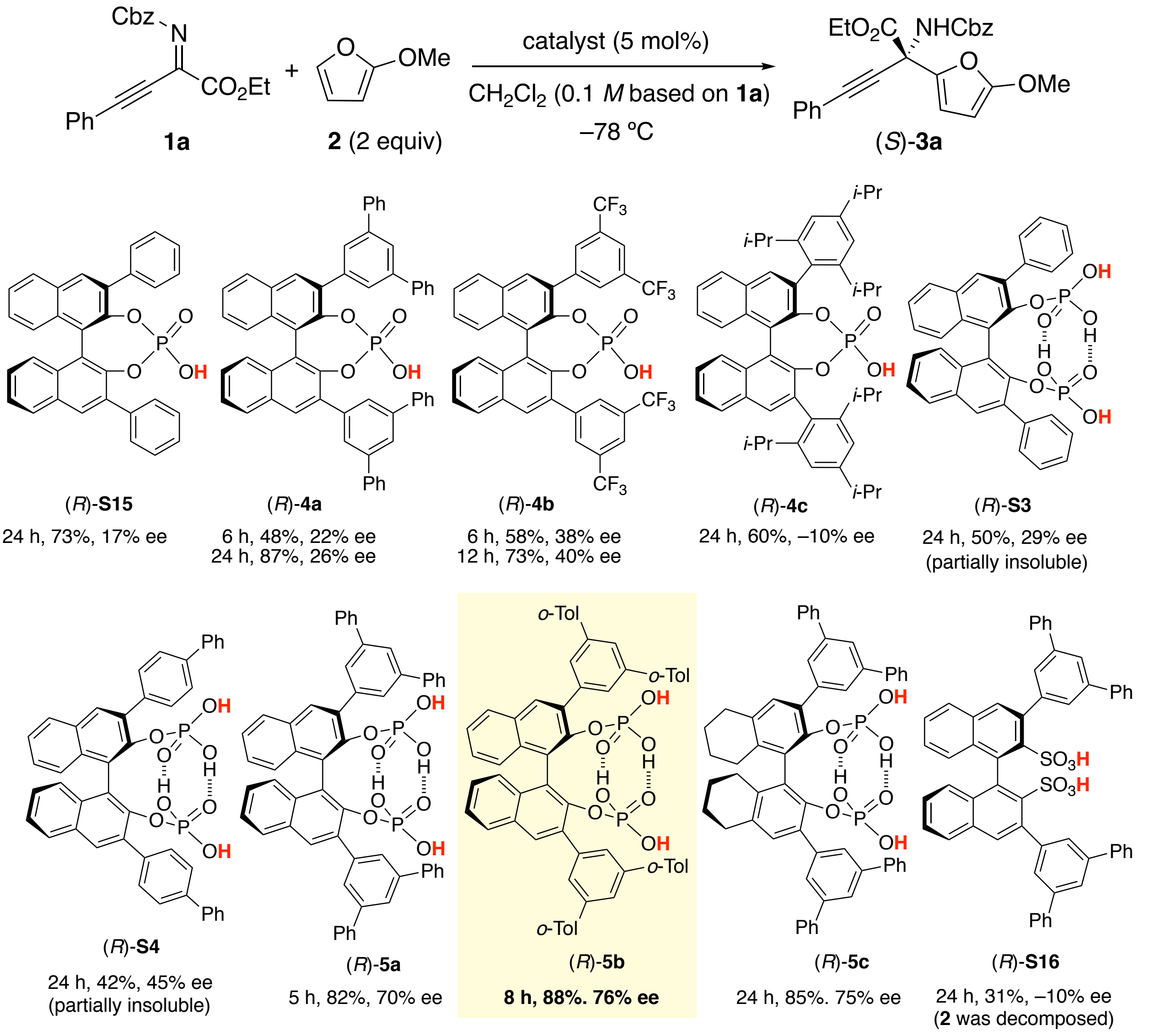

Scheme S1 Screening of the catalysts in the probe reaction of **2** with **1a**.

11. Screening of chiral catalysts in the probe reaction of 2 with 1b.

Substrate **1b** was much less reactive than **1a**. Therefore, the screening of chiral catalysts in the probe reaction of **2** with **1b** was examined again (Scheme S2). As a result, the catalytic activities of conventional chiral phosphoric acids (*R*)-**4a** and (*R*)-**4b** were low. An increase in the amount of (*R*)-**4b** from 5 mol% to 10 mol% slightly improved both the yield and the enantioselectivity. Moreover, chiral BINOL-derived phosphoric acids (*R*)-**4c** (i.e., TRIP) and (*R*)-**S17** were also quite inactive (<10% conversion). Chiral VAPOL-derived phosphoric acid (*R*)-**S18** moderately promoted the reaction but with low enantioselectivity. Chiral phosphoramide (*R*)-**S19** was also ineffective. Byproducts were obtained in the catalysis of (*R*)-**S18** (ca. 15% based on **1b**) and (*R*)-**S19** (ca. 20% based on **1b**). In contrast, bis(phosphoric acid)s (*R*)-**5a** and (*R*)-**5b** showed high yields with high enantioselectivities (up to 91% ee). In this reaction, less acidic (*R*)-**5c** gave a much lower yield and lower enantioselectivity (76% ee) than (*R*)-**5a** or (*R*)-**5b**. Based on these results, we selected (*R*)-**5b** as an optimized catalyst for this reaction.

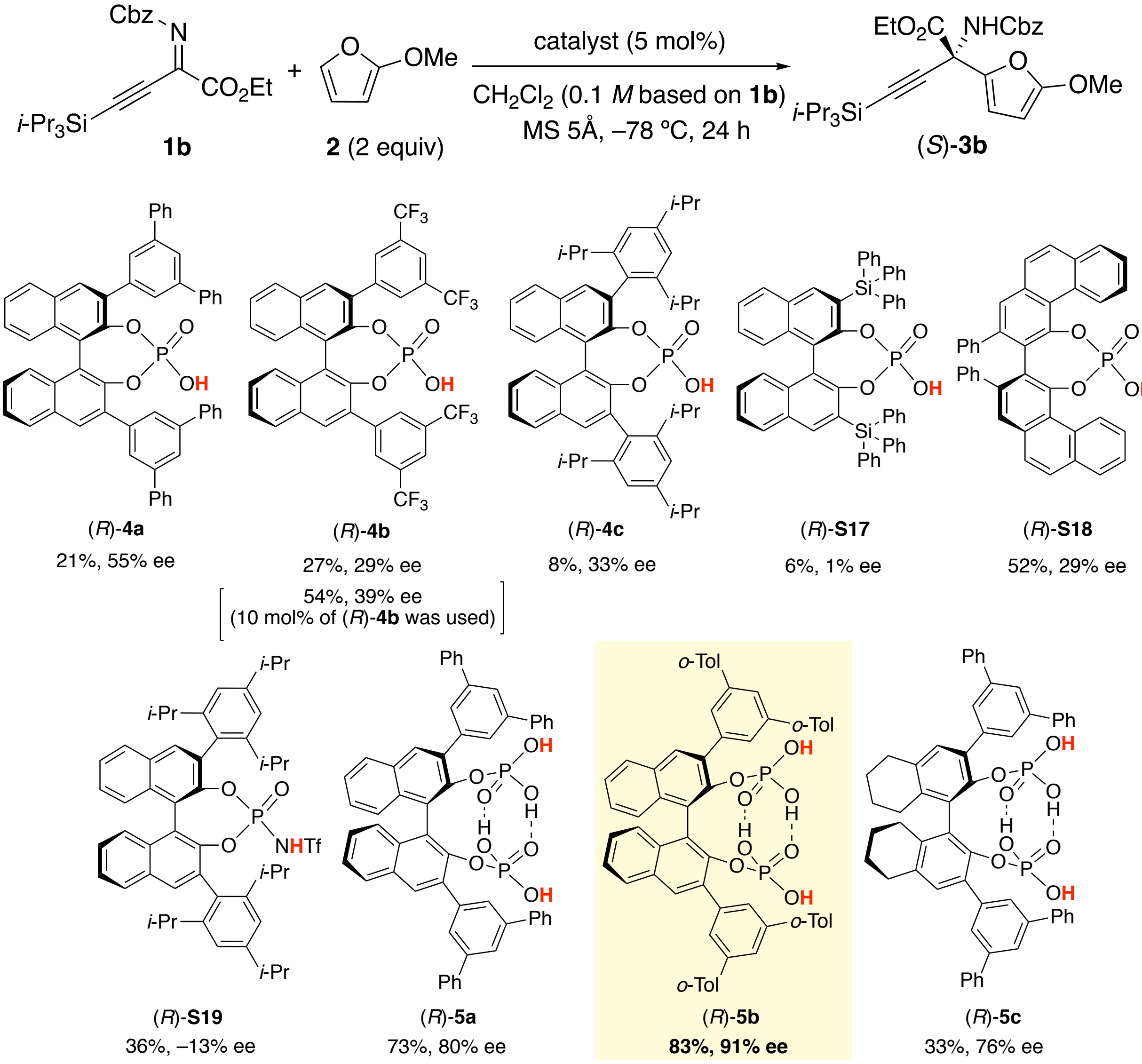

Scheme S2 Screening of the catalysts in the probe reaction of **2** with **1b**.

12. Calculation of the electrostatic potential of phosphoric acids.

An effective approach to estimating molecular pK_a values from simple density functional calculations has been developed by Liu.[14] Various compounds show a strong correlation between experimental pK_a values and molecular electrostatic potential (MEP). As a result of their research, a linear relationship between the MEP and experimental pK_a values has been established. Therefore, we performed preliminary theoretical calculations using Spartan'10 for Macintosh from Wavefunction, Inc. (Fig. S5 and Table S2). The geometries of **S20–S26** were optimized with gradient-corrected density functional theory (DFT) calculations with B3LYP using the 6-31+G* basis set, after MMFF (molecular mechanics) and HF/3-21G (*ab initio* molecular orbital method) calculations. We first investigated the MEP values of simple compounds **S20** and **S21**, which have known pK_a values (pK_a = 0.26[15] for **S20** and 1.42[16] for **S21**). As a result, a higher MEP value was observed in **S20** than in **S21**, and our preliminary calculations for these model compounds may support a relationship between pK_a and MEP values.

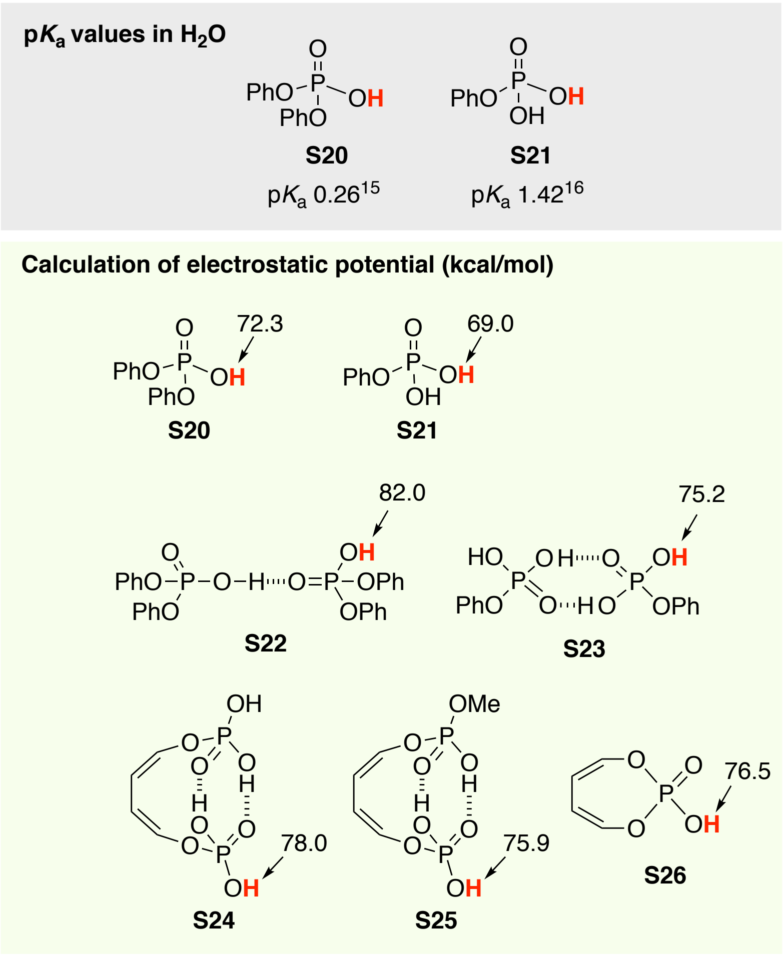

Fig. S5 Calculation of electrostatic potential (kcal/mol).

First, we investigated the effect of external hydrogen bonding in **S22** and **S23**, which involves one hydrogen bonding between two molecules of **S20** and two hydrogen bondings between two molecules of **S21**, respectively. Also, we observed a higher value of MEP (75.2 kcal/mol) for **S23** than for **S21** (69.0 kcal/mol). These results should clearly support the idea that appropriate hydrogen bonding between two molecules of phosphoric acids would increase the Brønsted acidity.

Next, we investigated the effect of two internal hydrogen bondings in **S24** as a simple model of (*R*)-**5**. As a result, we observed a higher value of MEP (78.0 kcal/mol) for cyclic **S24** than for acyclic **S23** (75.2 kcal/mol), although **S23** and **S24** have different ester moieties. Moreover, the MEP value for **S25** (75.9 kcal/mol) as a model of (*R*)-**10** was lower than that for **S24**. Moreover, the MEP value for **S26** (76.5 kcal/mol) as a model of (*R*)-**4** was lower than that for **S24** (78.0 kcal/mol). Accordingly, (*R*)-**5** might be expected to be more acidic than (*R*)-**4**, and (*R*)-**10** might be expected to be less acidic than (*R*)-**4** as shown in Fig. S6. It should be noted that the estimated order in Fig. S6 does not involve steric factors of the catalysts (also see Fig. S9).

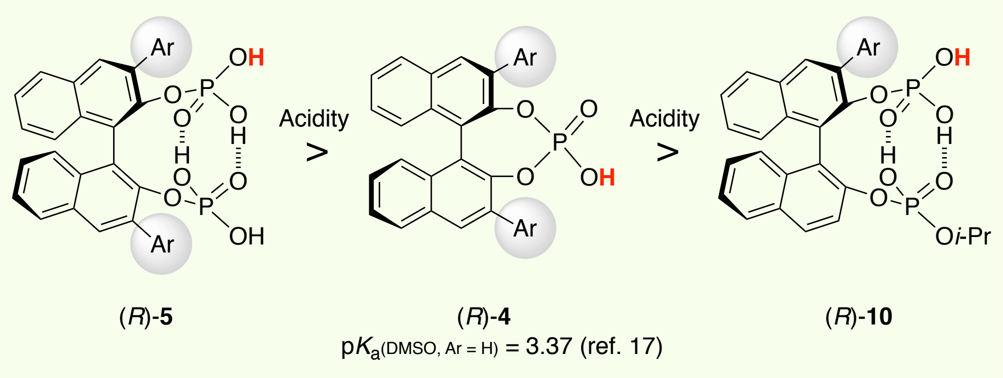

Fig. S6 Possible order of the strength of Brønsted acidity of (*R*)-**5**, (*R*)-**4**, and (*R*)-**10**.

Table S2 Summary of DFT calculation of **S20–S26** by using B3LYP/6-31+G*.

S20

```
Job type: Single point.
Method: RB3LYP
Basis set: 6-31+G*
Number of shells: 108
Number of basis functions: 349
Multiplicity: 1
Parallel Job: 4 threads
SCF model:
A restricted hybrid HF-DFT SCF calculation will be
performed using Pulay DIIS + Geometric Direct Minimization
SCF total energy:   -1106.2565201 hartrees
-------------------------------------------------------
```

Number	Atom	Charge	X(A)	Y(A)	Z(A)
1	P	0	-0.1230	-1.9290	0.0840
2	O	0	-1.0150	-2.7570	0.9210
3	O	0	0.7820	-2.7490	-0.9730
4	H	0	0.4900	-3.6740	-1.0280
5	O	0	-0.8030	-0.8630	-0.9230

6	O	0	0.9300	-1.0820	0.9590
7	C	0	-1.6920	0.1460	-0.5180
8	C	0	-3.4510	2.2030	0.1120
9	C	0	-2.7580	-0.1150	0.3430
10	C	0	-1.4910	1.4090	-1.0730
11	C	0	-2.3790	2.4380	-0.7550
12	C	0	-3.6340	0.9290	0.6570
13	H	0	-2.8950	-1.1080	0.7590
14	H	0	-0.6490	1.5710	-1.7390
15	H	0	-2.2290	3.4250	-1.1840
16	H	0	-4.4660	0.7380	1.3290
17	H	0	-4.1390	3.0070	0.3590
18	C	0	1.7690	-0.0460	0.5320
19	C	0	3.4810	2.0440	-0.1290
20	C	0	1.9280	1.0150	1.4220
21	C	0	2.4550	-0.0880	-0.6820
22	C	0	3.3100	0.9700	-1.0060
23	C	0	2.7890	2.0610	1.0860
24	H	0	1.3790	1.0090	2.3590
25	H	0	2.3310	-0.9280	-1.3560
26	H	0	3.8470	0.9450	-1.9510
27	H	0	2.9160	2.8910	1.7760
28	H	0	4.1510	2.8600	-0.3880

S21

Job type: Single point.
Method: RB3LYP
Basis set: 6-31+G*
Number of shells: 65
Number of basis functions: 208
Multiplicity: 1
Parallel Job: 4 threads
SCF model:
A restricted hybrid HF-DFT SCF calculation will be
performed using Pulay DIIS + Geometric Direct Minimization
SCF total energy: -799.9624168 hartrees

Number	Atom	Charge	X(A)	Y(A)	Z(A)
1	H	0	2.9260	-2.1460	0.0000
2	C	0	2.4020	-1.2080	0.0000
3	C	0	1.0520	1.2170	0.0000
4	C	0	1.0200	-1.1910	0.0000
5	C	0	3.1100	-0.0130	0.0000
6	C	0	2.4390	1.1960	0.0000
7	C	0	0.3400	0.0250	0.0000
8	H	0	0.4730	-2.1160	0.0000
9	H	0	4.1860	-0.0300	0.0000
10	H	0	2.9890	2.1200	0.0000
11	H	0	0.5220	2.1520	0.0000
12	P	0	-1.4480	0.1060	0.0000
13	O	0	-2.0390	1.4380	0.0000
14	O	0	-1.8790	-0.7790	-1.2560
15	H	0	-2.5920	-0.3810	-1.7420
16	O	0	-1.8790	-0.7790	1.2560
17	H	0	-2.5920	-0.3810	1.7420

S22

Job type: Single point.
Method: RB3LYP
Basis set: 6-31+G*
Number of shells: 216
Number of basis functions: 698
Multiplicity: 1
Parallel Job: 4 threads
SCF model:
A restricted hybrid HF-DFT SCF calculation will be
performed using Pulay DIIS + Geometric Direct Minimization
SCF total energy: -2212.5169304 hartrees

Number	Atom	Charge	X(A)	Y(A)	Z(A)
1	P	0	2.6350	1.0200	0.1910
2	O	0	1.2380	0.9950	-0.1950
3	O	0	3.5540	0.7700	-1.0590

4	O	0	3.0820	0.0130	1.3200
5	C	0	4.9370	0.7760	-1.0970
6	C	0	7.6870	0.7430	-1.2990
7	C	0	5.6210	1.9700	-1.0960
8	C	0	5.5960	-0.4310	-1.2100
9	C	0	6.9800	-0.4420	-1.3100
10	C	0	7.0050	1.9450	-1.1940
11	H	0	5.0790	2.8930	-1.0250
12	H	0	5.0300	-1.3390	-1.2280
13	H	0	7.5010	-1.3770	-1.4010
14	H	0	7.5470	2.8690	-1.1930
15	H	0	8.7600	0.7310	-1.3770
16	C	0	2.8490	-1.3630	1.3760
17	C	0	2.4940	-4.0830	1.5970
18	C	0	3.9450	-2.1660	1.6700
19	C	0	1.5790	-1.8820	1.2010
20	C	0	1.4110	-3.2560	1.3080
21	C	0	3.7580	-3.5340	1.7810
22	H	0	4.9150	-1.7190	1.8080
23	H	0	0.7370	-1.2560	0.9820
24	H	0	0.4240	-3.6600	1.1630
25	H	0	4.6020	-4.1680	2.0110
26	H	0	2.3550	-5.1470	1.6810
27	O	0	3.1360	2.3680	0.8330
28	H	0	3.4640	2.2820	1.7290
29	P	0	-2.1720	-0.2450	-0.3530
30	O	0	-1.5590	-1.4360	0.2070
31	O	0	-2.7930	0.6400	0.8100
32	O	0	-3.3570	-0.4720	-1.3760
33	C	0	-4.4310	-1.3140	-1.1620
34	C	0	-6.6250	-2.9570	-0.8600
35	C	0	-5.6940	-0.7540	-1.1310
36	C	0	-4.2420	-2.6820	-1.0460
37	C	0	-5.3500	-3.4990	-0.8920
38	C	0	-6.7930	-1.5840	-0.9800
39	H	0	-5.8050	0.3110	-1.2270
40	H	0	-3.2490	-3.0860	-1.0680
41	H	0	-5.2120	-4.5630	-0.8000
42	H	0	-7.7790	-1.1550	-0.9570
43	H	0	-7.4800	-3.5980	-0.7420
44	C	0	-3.4230	1.8580	0.7920
45	C	0	-4.7220	4.2930	0.9770
46	C	0	-3.8920	2.2970	2.0220
47	C	0	-3.5950	2.6230	-0.3510
48	C	0	-4.2480	3.8400	-0.2440
49	C	0	-4.5380	3.5130	2.1110
50	H	0	-3.7410	1.6780	2.8880
51	H	0	-3.2230	2.2910	-1.3010
52	H	0	-4.3800	4.4360	-1.1300
53	H	0	-4.8990	3.8510	3.0680
54	H	0	-5.2250	5.2400	1.0460
55	O	0	-1.2800	0.7000	-1.2110
56	H	0	-0.3690	0.7970	-0.9080

S23

Job type: Single point.
Method: RB3LYP
Basis set: 6-31+G*
Number of shells: 140
Number of basis functions: 454
Multiplicity: 1
Parallel Job: 4 threads
SCF model:
A restricted hybrid HF-DFT SCF calculation will be
performed using Pulay DIIS + Geometric Direct Minimization
SCF total energy: -1750.4488325 hartrees

Number	Atom	Charge	X(A)	Y(A)	Z(A)
1	P	0	1.5770	-1.9200	-0.4720
2	O	0	0.4630	-2.5520	-1.2420
3	O	0	1.8260	-0.4240	-0.9930
4	O	0	3.0190	-2.5960	-0.6880
5	H	0	3.0550	-3.4960	-0.3260
6	O	0	1.4060	-1.9070	1.1020
7	H	0	0.4680	-1.6450	1.3590

8	C	0	2.5410	0.5420	-0.2660
9	C	0	3.9180	2.5380	1.0700
10	C	0	1.8620	1.3210	0.6670
11	C	0	3.8890	0.7390	-0.5500
12	C	0	4.5770	1.7470	0.1280
13	C	0	2.5640	2.3230	1.3370
14	H	0	0.8100	1.1370	0.8580
15	H	0	4.3780	0.1110	-1.2870
16	H	0	5.6290	1.9120	-0.0840
17	H	0	2.0480	2.9370	2.0700
18	H	0	4.4570	3.3220	1.5950
19	P	0	-2.2670	-1.3620	0.5890
20	O	0	-1.0600	-1.0710	1.4150
21	O	0	-3.2200	-2.4420	1.2970
22	H	0	-3.8800	-2.8080	0.6850
23	O	0	-2.0180	-1.8620	-0.8900
24	O	0	-3.2780	-0.1140	0.4320
25	C	0	-2.8180	1.1150	-0.0700
26	C	0	-2.0290	3.5920	-1.0320
27	C	0	-2.6640	1.2900	-1.4430
28	C	0	-2.5880	2.1510	0.8320
29	C	0	-2.1920	3.3960	0.3410
30	C	0	-2.2640	2.5400	-1.9190
31	H	0	-2.8490	0.4590	-2.1140
32	H	0	-2.7220	1.9720	1.8940
33	H	0	-2.0100	4.2120	1.0340
34	H	0	-2.1350	2.6870	-2.9880
35	H	0	-1.7190	4.5630	-1.4090
36	H	0	-1.0600	-2.1530	-1.0730

S24

Job type: Single point.
Method: RB3LYP
Basis set: 6-31+G*
Number of shells: 88
Number of basis functions: 290
Multiplicity: 1
Parallel Job: 4 threads
SCF model:
A restricted hybrid HF-DFT SCF calculation will be performed using Pulay DIIS + Geometric Direct Minimization
SCF total energy: -1441.9200909 hartrees

Number	Atom	Charge	X(A)	Y(A)	Z(A)
1	C	0	-1.5540	-0.3740	1.9070
2	H	0	-2.4840	-0.9310	1.9650
3	C	0	-0.6370	-0.3640	2.8830
4	H	0	-0.8950	-0.9190	3.7830
5	C	0	0.6370	0.3640	2.8830
6	H	0	0.8950	0.9190	3.7830
7	C	0	1.5540	0.3740	1.9070
8	H	0	2.4840	0.9310	1.9650
9	O	0	1.4150	-0.4330	0.7910
10	O	0	-1.4150	0.4330	0.7910
11	P	0	1.8230	-0.0380	-0.7180
12	O	0	1.2420	1.2490	-1.2150
13	P	0	-1.8230	0.0380	-0.7180
14	O	0	-1.2420	-1.2490	-1.2150
15	O	0	-1.4370	1.3560	-1.4980
16	H	0	-0.4420	1.4850	-1.4950
17	O	0	-3.4310	0.0260	-0.6950
18	H	0	-3.7890	-0.6530	-1.2920
19	O	0	3.4310	-0.0260	-0.6950
20	H	0	3.7890	0.6530	-1.2920
21	O	0	1.4370	-1.3560	-1.4980
22	H	0	0.4420	-1.4850	-1.4950

S25

Job type: Single point.
Method: RB3LYP
Basis set: 6-31+G*
Number of shells: 97
Number of basis functions: 313

Multiplicity: 1
Parallel Job: 4 threads
SCF model:
A restricted hybrid HF-DFT SCF calculation will be performed using Pulay DIIS + Geometric Direct Minimization
SCF total energy: -1481.2250368 hartrees

Number	Atom	Charge	X(A)	Y(A)	Z(A)
1	C	0	-0.2400	2.9900	0.4210
2	H	0	-0.1970	3.8860	1.0390
3	C	0	-1.4760	2.7510	-0.3360
4	H	0	-1.9010	3.5910	-0.8840
5	C	0	0.8700	2.2430	0.3970
6	H	0	1.7550	2.4700	0.9840
7	C	0	-2.1880	1.6190	-0.3600
8	H	0	-3.1030	1.4990	-0.9320
9	O	0	1.0000	1.1770	-0.4760
10	O	0	-1.8500	0.5440	0.4450
11	P	0	1.6700	-0.2480	-0.1260
12	O	0	1.2040	-0.8530	1.1630
13	P	0	-1.9250	-1.0170	0.0580
14	O	0	-1.2860	-1.3760	-1.2480
15	O	0	-3.5000	-1.3450	0.0880
16	H	0	-3.7390	-2.0100	-0.5800
17	O	0	-1.3420	-1.6860	1.3620
18	H	0	-0.3650	-1.4740	1.4510
19	O	0	1.3850	-1.0810	-1.4430
20	H	0	0.4100	-1.2960	-1.5260
21	O	0	3.2340	0.0700	-0.1680
22	C	0	4.1870	-0.9160	0.2930
23	H	0	3.9330	-1.2420	1.3060
24	H	0	5.1550	-0.4160	0.2840
25	H	0	4.2000	-1.7690	-0.3910

S26

Job type: Single point.
Method: RB3LYP
Basis set: 6-31+G*
Number of shells: 56
Number of basis functions: 185
Multiplicity: 1
SCF model:
A restricted hybrid HF-DFT SCF calculation will be performed using Pulay DIIS + Geometric Direct Minimization
SCF total energy: -797.7340994 hartrees

Number	Atom	Charge	X(A)	Y(A)	Z(A)
1	C	0	-2.1770	0.7520	-0.1110
2	H	0	-3.0770	1.2520	-0.4610
3	C	0	-2.2210	-0.6930	0.0810
4	H	0	-3.1650	-1.1450	0.3760
5	C	0	-1.1090	1.5270	0.1290
6	H	0	-1.0900	2.6000	-0.0280
7	C	0	-1.1820	-1.5220	-0.0980
8	H	0	-1.2240	-2.5950	0.0550
9	O	0	0.0570	1.0620	0.7170
10	O	0	0.0360	-1.1110	-0.6160
11	P	0	1.0430	-0.0640	0.0970
12	O	0	2.0100	-0.5680	1.0890
13	O	0	1.6950	0.5900	-1.2250
14	H	0	2.6560	0.6850	-1.1160

13. Optimization of the concentration, drying agents, solvents, and substrates in the reaction of 2 with 1.

The effects of the concentration of substrate **1b** and drying agents were examined (Table S3). As a result, 0.1 *M* (based on **1b**) conditions showed better enantioselectivity than 0.05 *M* and 0.2 *M* (entries 1–3). The drying agent used did not significantly affect the enantioselectivity, but did affect the reactivity (i.e., the reaction time for full conversion) and chemoselectivity for unknown byproducts, which might be triggered by the reaction of water with **1b** and/or **2** (entries 4–7). As a result, MS 5Å was better than MS 3Å, MS 4Å, and $MgSO_4$. Next, the general solvent effect was examined (Table S4). As a result, polar solvents were not suitable at all, and dichloromethane was much better than the other solvents tested. The reaction temperature was also examined (entries 7–9), and a lower temperature gave higher enantioselectivity (92–94% ee), although the yields were decreased (46–52%).

Table S3 Effect of molecular sieves and concentration of substrate **1b**.

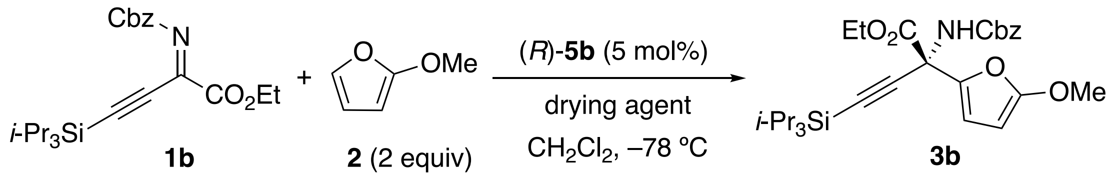

Entry	Drying agent	Concentration (*M*) based on **1b**	Reaction time (h)	Yield (%)	ee (%)
1	–	0.05	12	87	84
2	–	**0.1**	17	**72**	**90**
3	–	0.2	12	68	88
4	MS 3Å	0.1	48	85	89
5	MS 4Å	0.1	24	74	89
6	**MS 5Å**	**0.1**	24	**83**	**91**
7	$MgSO_4$	0.1	24	59	89

Table S4 Effect of solvents and temperature.

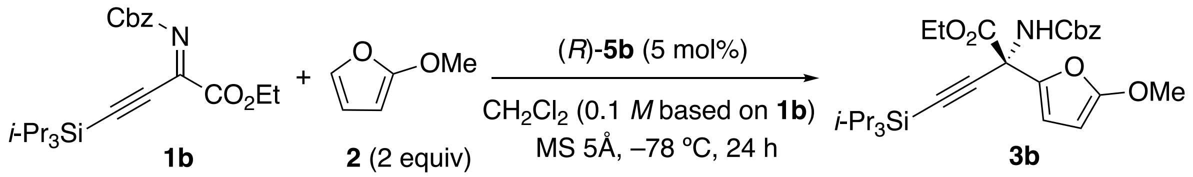

Entry	Solvent	Temperature (°C)	Reaction time (h)	Yield (%)	ee (%)
1	Et_2O	–78	24	0	–
2	EtOAc	–78	24	0	–
3	$EtNO_2$	–78	24	0	–
4	EtCN	–78	24	0	–
5	toluene	–78	24	35	9
6	**CH_2Cl_2**	**–78**	24	**83**	**91**
7	CH_2Cl_2	–60	14	78	76
8	CH_2Cl_2	–90	48	52	92
9	CH_2Cl_2	–95	48	46	94

Next, β,γ-alkynyl-α-imino esters **1** were optimized with the use of (*R*)-**5b** (Table S5). To avoid the effect of adventitious water, which might react with **1** and **2** to give undesired products, powdered MS 5Å was used as a drying agent. As a result, the reaction of **2** with **1a** (R^1 = Ph CO_2R^2 = CO_2Et, CO_2R^3 = Cbz) proceeded smoothly, and a slightly better result (88% yield and 76% ee) was observed (entry 1). Next, we changed the terminal R^1 group of the acetylene from a Ph group to sterically hindered silyl groups (entries 2–6). As a result, the enantioselectivity of the corresponding product **3** was improved according to the bulkiness of the silyl group (also see a possible transition state on page S72). Ultimately, when we used **1b** (R^1 = *i*-Pr_3Si, CO_2R^2 = CO_2Et, CO_2R^3 = Cbz) with a bulky *i*-Pr_3Si group, **3b** was obtained in 83% yield with 91% ee (entry 6). The ester groups CO_2R^2 and C=NCO_2R^3 in **1** were also optimized. However, we did not find better ester groups to replace CO_2Et for CO_2R^2 and Cbz for CO_2R^3 (entries 7–10). Based on these results, we selected **1b** (R^1 = *i*-Pr_3Si, CO_2R^2 = CO_2Et, CO_2R^3 = Cbz) as an optimized catalyst for this reaction.

Table S5 Screening of substrates.[a]

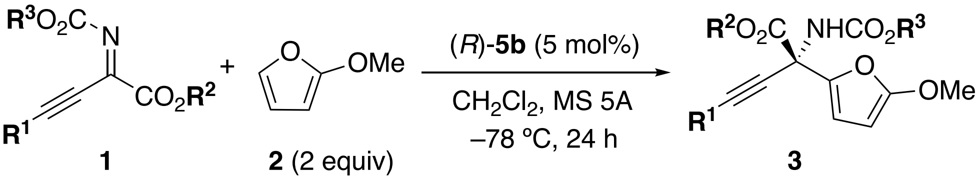

Entry	R^1	CO_2R^2	CO_2R^3	Yield (%)	ee (%)
1[b]	Ph	CO_2Et	CO_2Bn (Cbz)	88	76
2	Et_3Si	CO_2Et	Cbz	82	85
3	Ph_3Si	CO_2Et	Cbz	89	79
4	*t*-$BuMe_2Si$	CO_2Et	Cbz	88	82
5	*t*-$BuPh_2Si$	CO_2Et	Cbz	76	88
6[c]	***i*-Pr$_3$Si**	**CO_2Et**	**Cbz**	**83**	**91**
7	*i*-Pr_3Si	CO_2Et	CO_2Me	39	88
8	*i*-Pr_3Si	CO_2Et	CO_2*t*-Bu (Boc)	33	57
9	*i*-Pr_3Si	CO_2Me	Cbz	80	83
10	*i*-Pr_3Si	Cbz	Cbz	91	86

[a] The reaction was carried out with (*R*)-**5b** (5 mol%), **1** (1 equiv), and **2** (2 equiv) in dichloromethane (0.1 *M* based on **1**) at –78 °C for 24 h. [b] **1a**/**3a** system. Reaction time was 6 h. [c] **1b**/**3b** system. Reaction time was 6 h

14. Preparation of (*R*)-6 (Scheme 1).

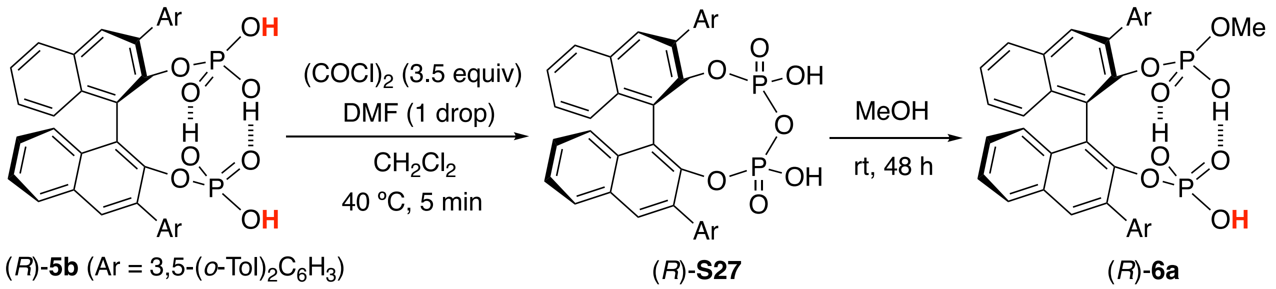

To a solution of (*R*)-**5b** (9.6 mg, 0.010 mmol) in dichloromethane (0.2 mL), one drop of *N*,*N*-dimethylformamide was added at room temperature. Then oxalyl chloride (3.0 μL, 0.035 mmol) was added at room temperature, and the mixture was warmed to 40 °C. The mixture was stirred at 40 °C for 5 min. Volatiles were removed *in vacuo* under heat conditions (ca. 40–50 °C). The obtained product (*R*)-**S27** would be used in the next step without further purification. (*R*)-**S27** was dissolved in methanol (2 mL) and the solution was stirred at room temperature for 4 h. Excess mthanol was then removed *in vacuo*. The obtained product was dissolved in toluene (2 mL), and the volatiles were thoroughly removed under reduced pressure to give pure (*R*)-**6a** as white-yellow solid (>99%, 9.7 mg). A trace amount of DMF remained.

Methyl ester (*R*)-6a: ^{1}H NMR (400 MHz, THF-d_8) 2.38 (s, 6H), 2.39 (s, 6H), 3.15 (d$_{H\text{–}P}$, J = 11.5 Hz, 3H), 4.84 (br, 3H), 7.16-7.40 (m, 20H), 7.43-7.49 (m, 2H), 7.54 (t, J = 8.7 Hz, 2H), 7.60 (t, J = 2.3 Hz, 4H), 7.98 (d, J = 8.2 Hz, 2H), 8.13 (s, 1H), 8.15 (s, 1H). ^{13}C NMR (100 MHz, THF-d_8) Many peaks were overlapped. δ 20.8 (2C), 20.9 (2C), 54.2 (d, $J_{C\text{–}P}$ = 5.7 Hz), 125.4, 125.9, 126.5-126.8 (m, 8C), 127.6 (2C), 127.9 (2C), 128.1 (2C), 129.0 (2C), 129.8, 129.9, 130.6 (8C), 131.0 (2C), 131.1 (2C), 132.3, 132.4, 132.7 (2C), 133.4, 133.6, 136.2 (2C), 136.4 (3C), 136.5, 139.3 (2C), 142.2 (2C), 142.4 (2C), 142.6 (2C), 143.0 (2C), 146.7 (d, $J_{C\text{–}P}$ = 7.6 Hz), 147.2 (d, $J_{C\text{–}P}$ = 5.7 Hz) [Contamination of a trace amount of acetone (δ 30.2) through the ^{13}C NMR analysis.]. ^{31}P NMR (160 MHz, THF-d_8) δ –0.27, 0.75. IR (KBr) 3448, 2924, 2854, 1593, 1492, 1450, 1398, 1239, 1188, 1058 cm^{-1}. M.p. 142 °C (decomposition). $[\alpha]_D^{23}$ = +189.6 (*c* 1.00, $CHCl_3$). HRMS (ESI–) calcd for $C_{61}H_{49}O_8P_2$ $[M–H]^-$ 971.2908, found 971.2911.

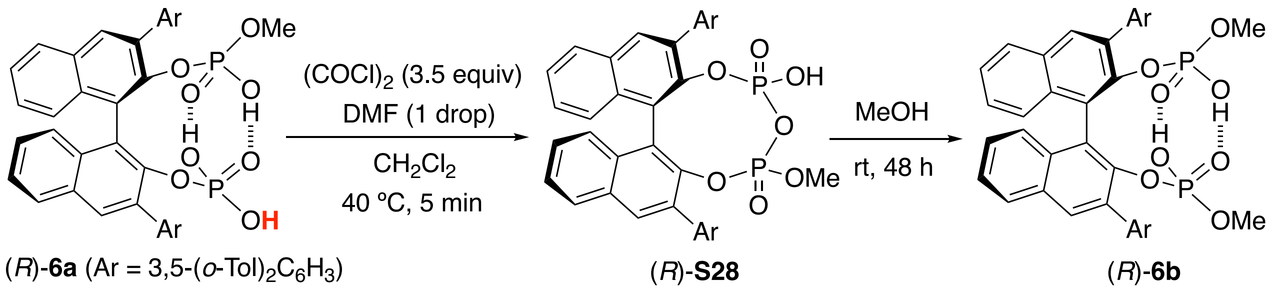

To a solution of (*R*)-**6a** (97.3 mg, 0.10 mmol) in dichloromethane (2 mL), three drops of *N*,*N*-dimethylformamide was added at room temperature. Then oxalyl chloride (15 μL, 0.175 mmol) was added at room temperature, and the mixture was warmed to 40 °C. The mixture was

stirred at 40 °C for 5 min. Then oxalyl chloride (15 μL, 0.175 mmol) was added at room temperature, and the mixture was warmed to 40 °C. The mixture was stirred at 40 °C for 5 min. Volatiles were removed *in vacuo* under heat conditions (ca. 40–50 °C). The obtained product (*R*)-**S28** would be used in the next step without further purification. (*R*)-**S28** was dissolved in mixed solvent of dichloromethane (2 mL) and methanol (4 ml) the solution was stirred at room temperature for 2 h. The solution was concentrated under reduced pressure, and the crude product was purified by silica gel column chromatography (eluent: $CHCl_3$:MeOH = 8:1 to 1:1) to give (*R*)-**6b**, which would be contaminated with alkali and alkali earth metal ions. The obtained (*R*)-**6b** was dissolved in dichloromethane, and throughly washed with 1 *M* HCl aqueous solution, and the organic phase was separated. After the removal of volatiles under reduced pressure, the residue was dissolved in toluene, and the volatiles were thoroughly removed under reduced pressure to give pure (*R*)-**6b** as white-yellow solid (87%, 86.1 mg).

Dimethyl ester (*R*)-6b: ^{1}H NMR (400 MHz, THF-d_8) δ 2.40 (s, 12H), 3.10 (d_{H-P}, J = 11.5 Hz, 6H), 4.19 (br, 2H), 7.19-7.30 (m, 12H), 7.32-7.43 (m, 8H), 7.47 (t, J = 7.8 Hz, 2H). 7.57 (d, J = 8.7 Hz, 2H), 7.61 (s, 4H), 7.98 (d, J = 7.8 Hz, 2H), 8.15 (s, 2H). ^{13}C NMR (100 MHz, THF-d_8) δ 20.9 (4C), 54.3 (d, J_{C-P} = 4.8 Hz, 2C), 125.6 (2C), 126.6 (4C), 126.7 (2C), 126.9 (2C), 127.7 (2C), 128.1 (4C), 129.0 (2C), 130.0 (2C), 130.5 (4C), 130.6 (4C), 131.1 (4C), 132.6 (2C), 132.7 (2C), 133.5 (2C), 136.2 (6C), 139.3 (2C), 142.4 (4C), 142.7 (4C), 146.5 (d, J_{C-P} = 6.7 Hz, 2C) [Contamination of a trace amount of acetone (δ 30.2) through the ^{13}C NMR analysis.]. ^{31}P NMR (160 MHz, THF-d_8) δ –1.4. IR (KBr) 3433, 2953, 2927, 2853, 1593, 1492, 1450, 1400, 1241, 1187, 1151, 1060 cm^{-1}. M.p. 140 °C (decomposition). $[\alpha]_D^{22}$ = +212.4 (*c* 1.00, $CHCl_3$). HRMS (ESI–) calcd for $C_{62}H_{51}O_8P_2$ $[M–H]^-$ 985.3065, found 985.3065.

Summary of the reaction with the use of (*R*)-**5b**, (*R*)-**6a**, and (*R*)-**6b** is shown in Scheme S3.

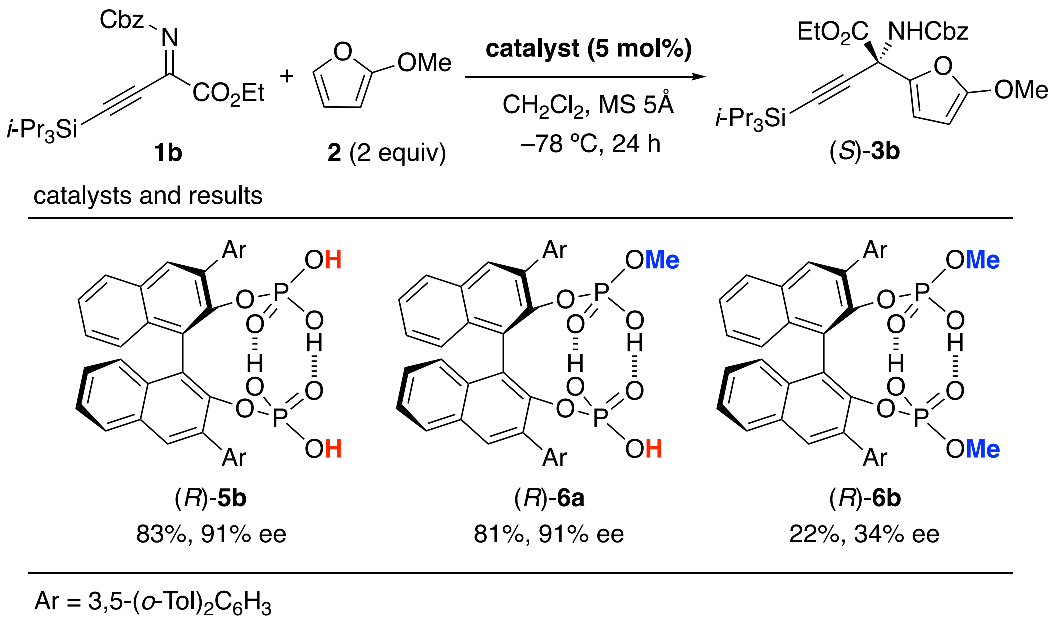

Scheme S3 Role of Brønsted acid in the catalysts.

15. Non-linear effect in the reaction of 2 with 1a (Fig. 3).

The presence of a non-linear effect was examined in the reaction of 2-methoxyfuran **2** (0.80 mmol) with α-ketimino ester **1a** (0.40 mmol) in the presence of (*R*)-**5b** (5 mol%, 0% ee to 100% ee) in dichloromethane (0.1 *M* based on **1a**) at –78 °C for 6 h. As shown in Scheme S4, a non-linear effect was not observed. Moreover, the yields of (*S*)-**3a** were independent of the enantiopurity of (*R*)-**5b**, and (*S*)-**3a** was obtained in a consistent yield of 71–75%. Therefore, a possible active species might be the monomeric structure of (*R*)-**5b**.

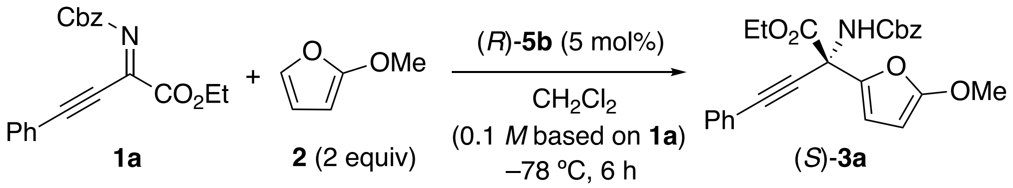

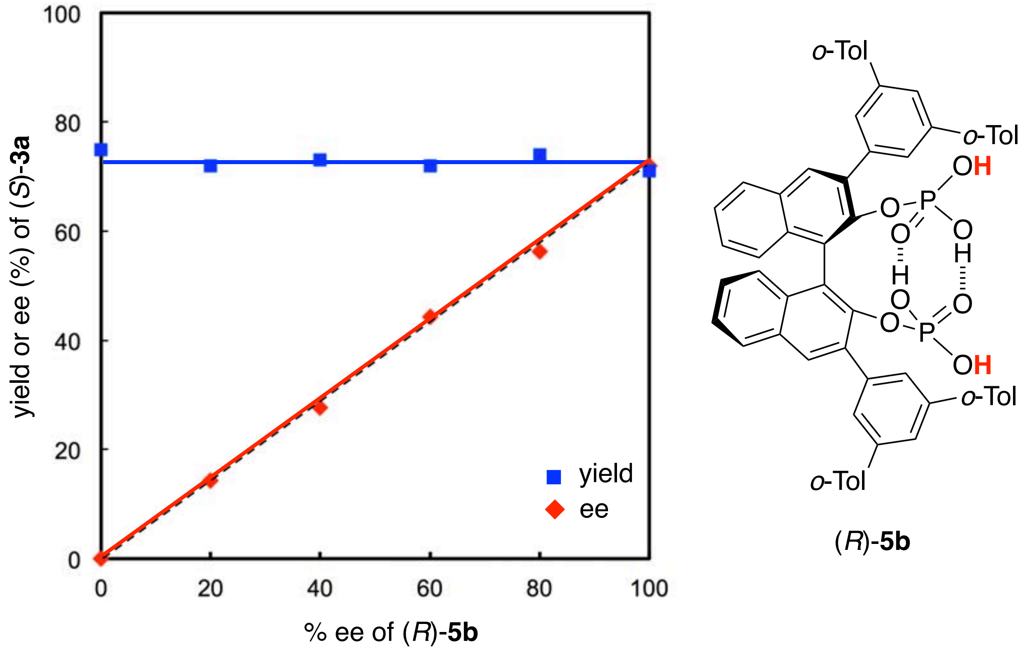

Scheme S4 Plot of the yield (%) and ee of (*S*)-**3a** vs. ee of (*R*)-**5b**.

We also examined the reaction of **2** with **1a** in the presence of (*R*)-**4a** or (*R*)-**4b** (5 mol%, 0% ee to 100% ee) in dichloromethane (0.1 *M* based on **1a**) at –78 °C (Schemes S5 and S6). The reaction time was 12 h for (*R*)-**4a** catalysis, and 6 h for (*R*)-**4b** catalysis. As shown in Scheme S5, a positive non-linear effect was observed for (*R*)-**4a**-catalysis. This result strongly suggests that (*R*)-**4a**-catalysis might involve the dimeric structure of (*R*)-**4a**. In contrast, as shown in Scheme S6, a non-linear effect was not observed for (*R*)-**4b**-catalysis. This result strongly suggests again that a possible active species might be the monomeric structure of (*R*)-**4b**. The P=O moiety of (*R*)-**4b** is much less basic than (*R*)-**4a**, and therefore, the dimeric structure might not be involved in (*R*)-**4b** catalysis.

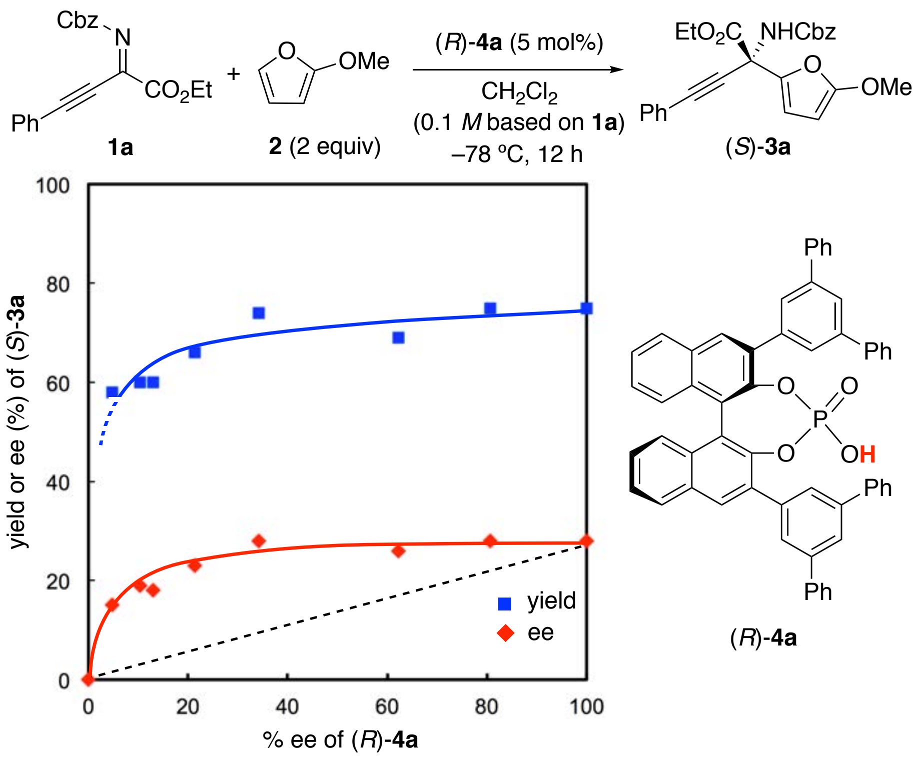

Scheme S5 Plot of the yield (%) and ee of (*S*)-**3a** vs. ee of (*R*)-**4a**.

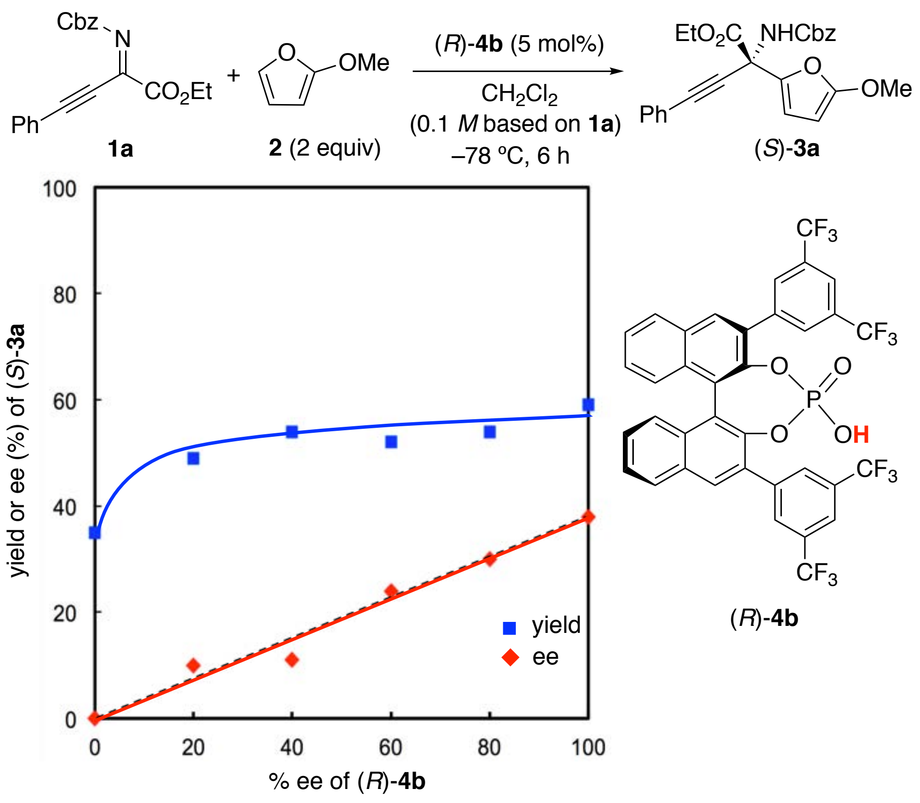

Scheme S6 Plot of the yield (%) and ee of (*S*)-**3a** vs. ee of (*R*)-**4b**.

16. ESI-MS analysis of catalysts.

[Preparation of the samples for S20 (Fig. S7a), (*R*)-S15 (Fig. S7b), and (*R*)-S3 (Fig. S7c)] Acid (0.01 mmol) was dissolved in dichloromethane (200 μL) in a test tube at room temperature. After 30 min, 20 μL of the resulting solution was diluted with mixed solvent of dichloromethane (80 μL), methanol (50 μL) and acetonitrile (50 μL) in a test tube (final concentration: 5.0 m*M*), and passed through a membrane filter (200 mm mesh) just before injection. The spectra are shown in Figs. S5a–c.

[Preparation of the samples for (*R*)-S3 (Fig. S7d), (*R*)-5b (Fig. S7e), (*R*)-10c (Fig. S7f)] Acid (0.01 mmol) was dissolved in dichloromethane (200 μL) in a test tube at room temperature. After 30 min, 20 μL of the resulting solution was diluted with dichloromethane (180 μL) in a test tube (final concentration: 5.0 m*M*), and passed through a membrane filter (200 mm mesh) just before injection. The spectra are shown in Figs. S5d–f.

Since **S20** and (*R*)-**S15** could not be detected in less polar solvents such as dichloromethane probably due to the inherent ionization problem with **S20** and (*R*)-**S15**, we first used polar solvents. As a result, the ESI-MS (negative) analysis of **S20** in $CH_2Cl_2/MeOH/CH_3CN$ = 2/1/1, as shown in Fig. S7a, clearly suggests that **S20** would not be a monomer. Instead, a dimer, trimer, tetramer, 5-mer, and 6-mer were observed under polar solvent conditions. (*R*)-**S15** as shown in Figs. S5b would be a monomer, but a dimer, trimer, and tetramer were also observed as major species. In sharp contrast, the population of dimer, trimer, and tetramer of (*R*)-**S3** in $CH_2Cl_2/MeOH/CH_3CN$ = 2/1/1 was reduced, as shown in Fig. S7c. Next, we used CH_2Cl_2 alone for highly soluble (*R*)-**S3**, as shown in Fig. S7d. As a result, a trimer and tetramer were not observed and the population of dimer was greatly decreased. Less polar solvents might be favored for hydrogen bonding, and the intramolecular double hydrogen bond network might be maintained under CH_2Cl_2 solvent conditions. Moreover, much more sterically hindered (*R*)-**5b** and (*R*)-**10c** in CH_2Cl_2 gave spectra (Figs.S5e and S5f) that were quite similar to that in Fig. S7d. Overall, this ESI-MS analysis suggests that bis(phosphoric acid)s, such as (*R*)-**S3**, (*R*)-**5b**, (*R*)-**10c**, would remain mostly as a monomer, whereas phosphoric acids, such as **S20** and (*R*)-**S15**, would easily exhibit dimer, trimer, and tetramer forms. Overall, Fig. S8 summarizes the possible aggregation of the catalysts **S20**, (*R*)-**S15**, (*R*)-**5b**, and (*R*)-**10c**.

The correlation between the possible acidity (see Table S1, and Figs. S5 and S6), aggregation (see Figs. S7 and S8), and catalytic activity (see Table S1 and Scheme S1) of the catalysts is shown in Fig. S9. Catalyst (*R*)-**5b** might have much better Brønsted acidity than the others, and might avoid aggregation due to neutralization of the highly Brønsted basic P=O moiety through the conjugated double hydrogen bond network. As a result, catalyst (*R*)-**5b** would show better results than the others.

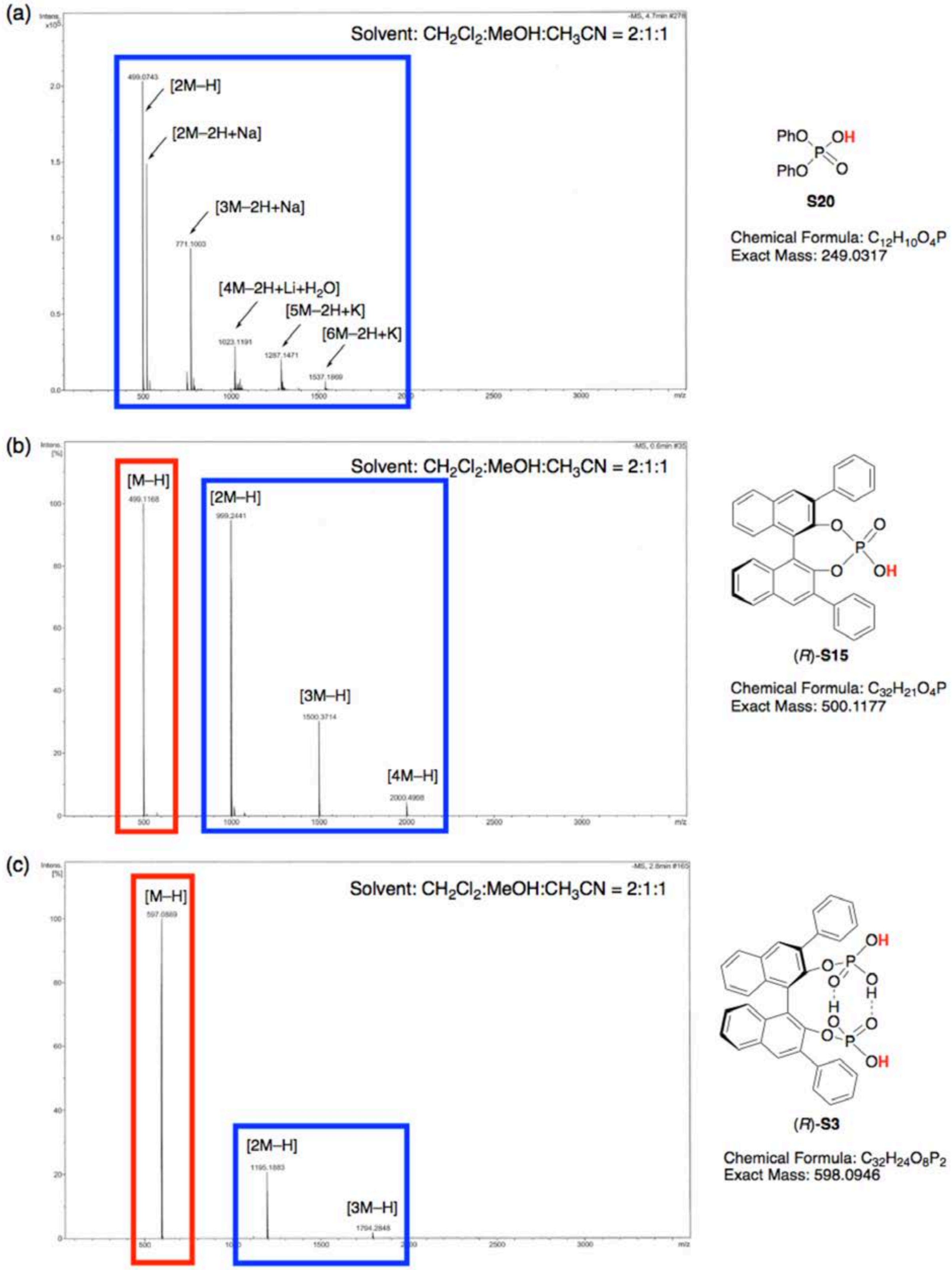

Fig. S7 ESI-MS (negative) spectrum of catalysts. (a) **S20** in CH_2Cl_2/MeOH/CH_3CN. (b) (*R*)-**S15** in CH_2Cl_2/MeOH/CH_3CN. (c) (*R*)-**S3** in CH_2Cl_2/MeOH/CH_3CN. (d) (*R*)-**S3** in CH_2Cl_2. (e) (*R*)-**5b** in CH_2Cl_2. (f) (*R*)-**10c** in CH_2Cl_2

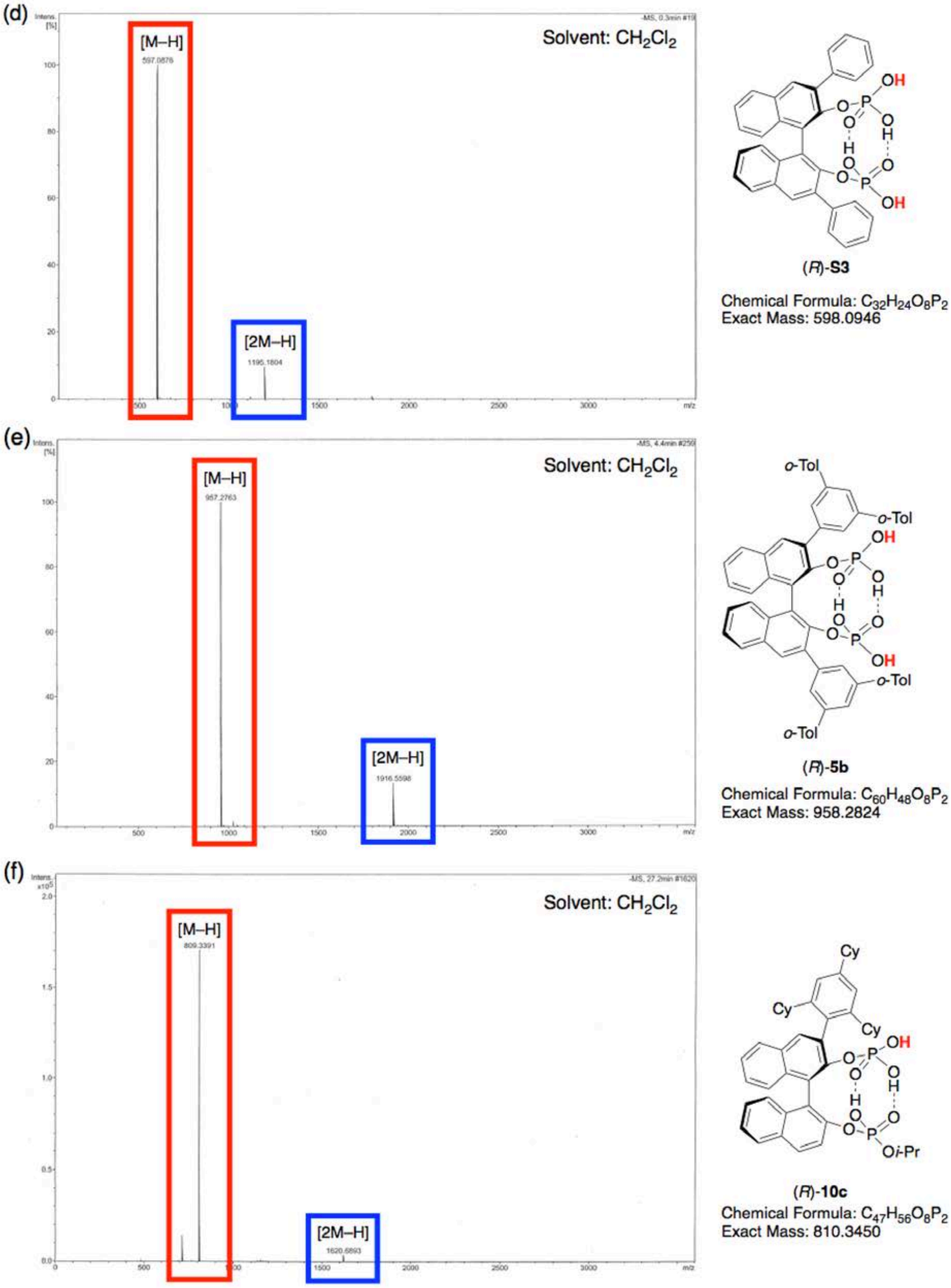

Fig. S7 *(continued)*

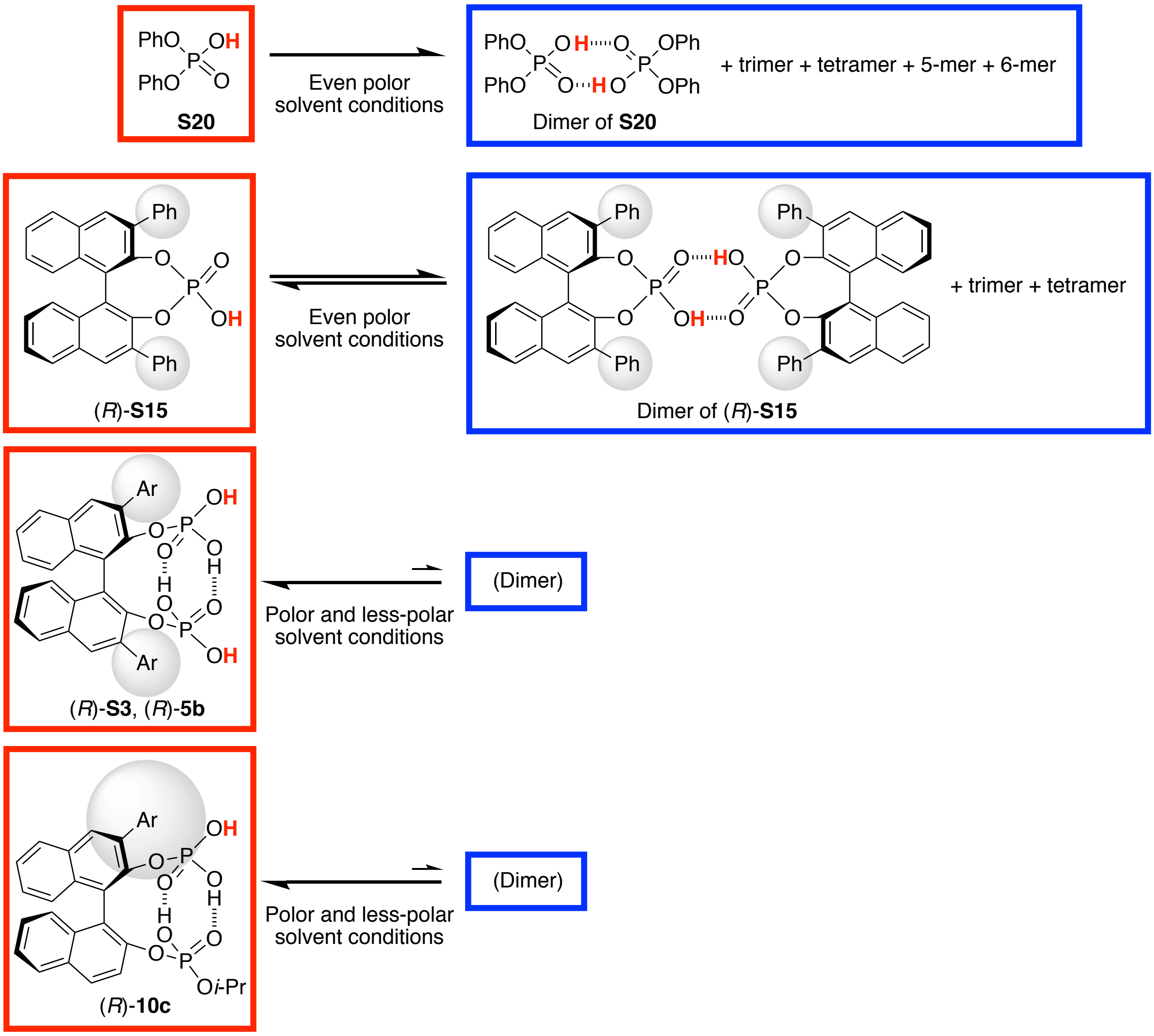

Fig. S8 Possible equilibriums among monomer, dimer, and trimer of catalysts.

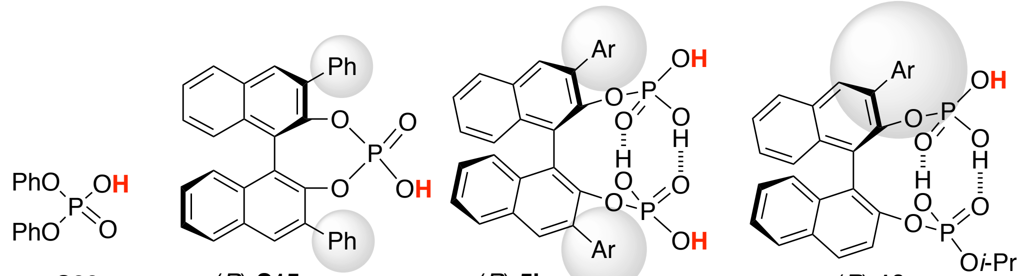

	**S20**	(*R*)-**S15**	(*R*)-**5b**	(*R*)-**10c**
Brønsted acidity:	+	+	+++	+
Aggregetion:	+++	++	–	–
**Catalytic activity:**	+	++	+++	++

Fig. S9 Correlations among the possible acidity, aggregation, and catalytic activity of the catalysts.

17. Preparation of α-ketimino esters 7.

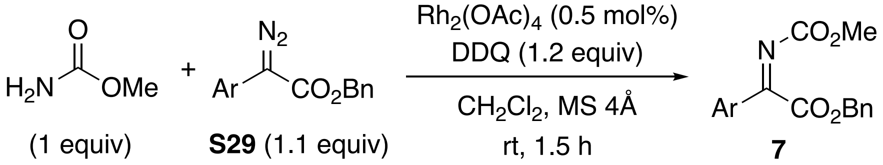

α-Ketimino esters **7** were prepared based on the literature procedure.[18] A suspension of rhodium(II) acetate dimer ($Rh_2(OAc)_4$, 6.6 mg, 0.5 mol%), methyl carbamate (225 mg, 3.0 mmol), 2,3-dichloro-5,6-dicyano-1,4-benzoquinone (DDQ, 817 mg, 3.6 mmol), and well-dried MS 4Å (2 g) in dichloromethane (15 mL) was stirred at room temperature. Then the diazo compound **S29**[19] (3.3 mmol) in dichloromethane (10 mL) was added to the suspension over 1 h *via* a syringe pump. After completion of the addition, the reaction mixture was stirred for another 0.5 h. The resulting mixture was filtered through a pad of Celite, and the filtrate was condensed under reduced pressure. The residue was purified by silica gel column chromatography (eluent: dichloromethane) to give the desired product **7**.

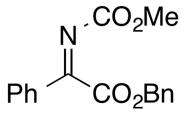

Benzyl 2-((methoxycarbonyl)imino)-2-phenylacetate (7a): Light yellow oil. ^{1}H NMR (400 MHz, $CDCl_3$) δ 3.65 (s, 3H), 5.37 (s, 2H), 7.36-7.46 (m, 7H), 7.56 (t, J = 7.3 Hz, 1H), 7.86 (d, J = 7.8 Hz, 2H). ^{13}C NMR (100 MHz, $CDCl_3$) δ 53.7, 68.4, 128.7 (2C), 128.8 (2C), 128.9, 129.0 (2C), 129.4 (2C), 132.0, 133.3, 134.2, 162.1, 162.4, 162.9. IR (neat) 2953, 1739, 1635, 1450, 1316, 1232, 1004 cm^{-1}. HRMS (FAB+) calcd for $C_{17}H_{16}NO_4$ $[M+H]^+$ 298.1079, found 298.1069.

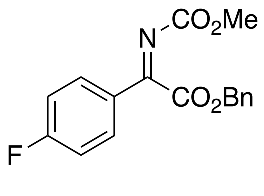

Benzyl 2-(4-fluorophenyl)-2-((methoxycarbonyl)imino)acetate (7b): Light yellow oil. ^{1}H NMR (400 MHz, $CDCl_3$) δ 3.64 (s, 3H), 5.36 (s, 2H), 7.08-7.15 (m, 2H), 7.35-7.45 (m, 5H), 7.86-7.95 (m, 2H). ^{13}C NMR (100 MHz, $CDCl_3$) δ 53.7, 68.5, 116.1 (d, J_{C-F} = 21.9 Hz, 2C), 128.2 (d, J_{C-F} = 2.9 Hz), 128.8 (2C), 129.0 (3C), 131.9 (d, J_{C-F} = 8.6 Hz, 2C), 134.0, 161.4, 161.9, 162.1, 165.8 (d, J_{C-F} = 254.6 Hz). ^{19}F NMR (376 MHz, $CDCl_3$) δ –104.2. IR (neat) 2954, 2846, 1739, 1636, 1593, 1509, 1437, 1415, 1324, 1229, 1158, 1037, 1002 cm^{-1}. HRMS (FAB+) calcd for $C_{17}H_{15}FNO_4$ $[M+H]^+$ 316,0985, found 316.0994.

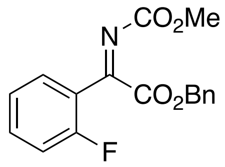

Benzyl 2-(2-fluorophenyl)-2-((methoxycarbonyl)imino)acetate (7c): Colorless oil. ^{1}H NMR (400 MHz, $CDCl_3$, 50 °C) δ 3.70 (s, 3H), 5.31 (s, 2H), 7.08 (t, J = 8.7 Hz, 1H), 7.20 (t, J = 7.3 Hz, 1H), 7.30-7.40 (m, 5H), 7.49 (q, J = 6.9 Hz, 1H), 7.75 (br, 1H). ^{13}C NMR (100 MHz, $CDCl_3$, 50 °C) δ 53.7, 68.6, 116.2 (d, $J_{C–F}$ = 21.0 Hz,), 121.9 (d, $J_{C–F}$ = 10.5 Hz), 124.7 (d, $J_{C–F}$ = 2.9 Hz), 128.7 (2C), 128.8 (3C), 130.5, 134.4, 134.5 (d, $J_{C–F}$ = 8.6 Hz,), 158.9, 161.5, 161.6, 161.7 (d, $J_{C–F}$ = 253.6 Hz). ^{19}F NMR (376 MHz, $CDCl_3$,) δ –111.5. IR (neat) 3035, 2955, 1745, 1639, 1613, 1486, 1457, 1266, 1229, 1000 cm^{-1}. HRMS (ESI+) calcd for $C_{17}H_{15}FNO_4$ $[M+H]^+$ 316,0980, found 316.0986.

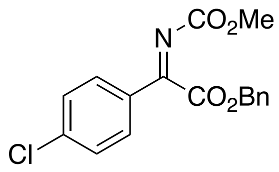

Benzyl 2-(4-chlorophenyl)-2-((methoxycarbonyl)imino)acetate (7d): Light yellow oil. ^{1}H NMR (400 MHz, $CDCl_3$) δ 3.64 (s, 3H), 5.36 (s, 2H), 7.35-7.44 (m, 7H), 7.82 (d, J = 8.2 Hz, 2H). ^{13}C NMR (100 MHz, $CDCl_3$) δ 53.8, 68.6, 128.8 (2C), 129.1 (5C), 130.5, 130.7 (2C), 134.0, 139.8, 161.4, 161.9 (2C). IR (neat) 2953, 1739, 1633, 1592, 1568, 1492, 1436, 1405, 1377, 1321, 1284, 1232, 1176, 1092, 1037, 1002 cm^{-1}. HRMS (FAB+) calcd for $C_{17}H_{15}ClNO_4$ $[M+H]^+$ 332.0690, found 332.0681.

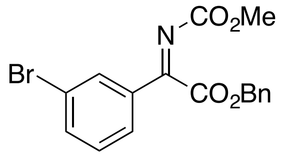

Benzyl 2-(3-bromophenyl)-2-((methoxycarbonyl)imino)acetate (7e): Light yellow oil. ^{1}H NMR (400 MHz, $CDCl_3$) δ 3.64 (s, 3H), 5.36 (s, 2H), 7.30 (t, J = 7.8 Hz, 1H), 7.35-7.45 (m, 5H), 7.67 (dm, J = 7.8 Hz, 1H), 7.78 (d, J = 7.8 Hz, 1H), 8.03 (s, 1H). ^{13}C NMR (100 MHz, $CDCl_3$) δ 53.8, 68.7, 122.9, 128.0, 128.9 (2C), 129.1 (3C), 130.2, 132.2, 134.0 (2C), 136.1, 160.9, 161.6, 161.7. IR (neat) 2953, 1739, 1638, 1231, 1198 cm^{-1}. HRMS (FAB+) calcd for $C_{17}H_{15}BrNO_4$ $[M+H]^+$ 376.0184, found 376.0174.

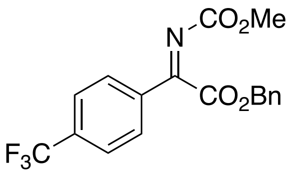

Benzyl 2-((methoxycarbonyl)imino)-2-(4-(trifluoromethyl)phenyl)acetate (7f): Light yellow oil. ^{1}H NMR (400 MHz, $CDCl_3$) δ 3.65 (s, 3H), 5.37 (s, 2H), 7.37-7.45 (m, 5H), 7.69 (d, J = 8.7 Hz, 2H), 7.98 (d, J = 7.8 Hz, 2H). ^{13}C NMR (100 MHz, $CDCl_3$) δ 53.9, 68.8, 123.6 (q, $J_{C–F}$ = 271.3 Hz), 125.7 (q, $J_{C–F}$ = 3.8 Hz, 2C), 128.9 (2C), 129.2 (3C), 129.8 (2C), 133.9, 134.4 (q, $J_{C–F}$ = 32.4 Hz), 135.4, 160.6, 161.3, 161.6. ^{19}F NMR (376 MHz, $CDCl_3$) δ –63.1. IR (neat) 2956, 1740, 1643, 1438, 1413, 1328, 1233, 1129, 1068, 1001 cm^{-1}. HRMS (FAB+) calcd for $C_{18}H_{15}F_3NO_4$ $[M+H]^+$ 366.0953, found 366.0957.

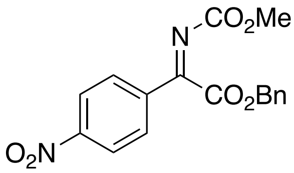

Benzyl 2-((methoxycarbonyl)imino)-2-(4-nitrophenyl)acetate (7g): Yellow powder. ^{1}H NMR (400 MHz, $CDCl_3$) δ 3.65 (s, 3H), 5.38 (s, 2H), 7.39-7.44 (m, 5H), 8.05 (d, J = 8.3 Hz, 2H), 8.27 (d, J = 8.7 Hz, 2H). ^{13}C NMR (100 MHz, $CDCl_3$) δ 53.9, 69.1, 123.7 (2C), 128.9 (2C), 129.2 (2C), 129.3, 130.5 (2C), 133.7, 137.7, 150.2, 159.4, 160.7, 161.3. IR (KBr) 3115, 2962, 1742, 1724, 1644, 1522, 1350, 1318, 1246, 1199, 1004 cm^{-1}. M.p. 72-74 °C (decomposition). HRMS (FAB+) calcd for $C_{17}H_{15}N_2O_6$ $[M+H]^+$ 343.0930, found 343.0932.

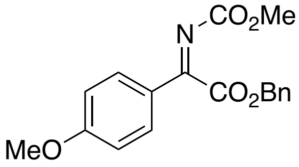

Benzyl 2-((methoxycarbonyl)imino)-2-(4-methoxyphenyl)acetate (7h): Light yellow oil. ^{1}H NMR (400 MHz, $CDCl_3$) δ 3.65 (s, 3H), 3.87 (s, 3H), 5.36 (s, 2H), 6.92 (d, J = 8.7 Hz, 2H), 7.34-7.47 (m, 5H), 7.85 (d, J = 8.2 Hz, 2H). ^{13}C NMR (100 MHz, $CDCl_3$) δ 53.7, 55.6, 68.2, 114.3 (2C), 124.5, 128.8 (2C), 128.9, 129.0 (2C), 131.7 (2C), 134.4, 162.4, 163.1, 163.2, 164.0. IR (neat) 2953, 1739, 1598, 1571, 1514, 1310, 1228, 1168 cm^{-1}. HRMS (FAB+) calcd for $C_{18}H_{17}NNaO_5$ $[M+Na]^+$ 350.1004, found 350.1016.

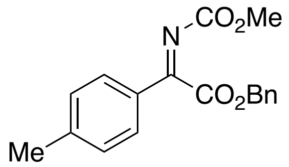

Benzyl 2-((methoxycarbonyl)imino)-2-(*p*-tolyl)acetate (7i): Light yellow oil. ^{1}H NMR (400 MHz, $CDCl_3$) δ 2.41 (s, 3H), 3.65 (s, 3H), 5.36 (s, 2H), 7.23 (d, J = 8.2 Hz, 2H), 7.33-7.49 (m, 5H), 7.76 (d, J = 8.2 Hz, 2H). ^{13}C NMR (100 MHz, $CDCl_3$) δ 21.8, 53.7, 68.3, 128.8 (2C), 128.9, 129.0 (2C), 129.3, 129.5 (2C), 129.6 (2C), 134.3, 144.5, 162.3, 162.8, 163.2. IR (neat) 3033, 2953, 1740, 1633, 1605, 1436, 1323, 1228, 1177, 1038, 1003 cm^{-1}. HRMS (FAB+) calcd for $C_{18}H_{17}NNaO_4$ [M+Na]$^+$ 334.1055, found 334.1047.

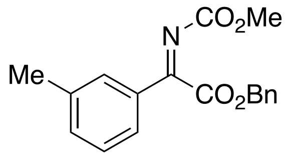

Benzyl 2-((methoxycarbonyl)imino)-2-(*m*-tolyl)acetate (7j): Colorless oil. ^{1}H NMR (400 MHz, $CDCl_3$) δ 2.36 (s, 3H), 3.66 (s, 3H), 5.37 (s, 2H), 7.30-7.44 (m, 7H), 7.65 (s, 2H). ^{13}C NMR (100 MHz, $CDCl_3$) δ 21.4, 53.8, 68.3, 126.7, 128.7, 128.8 (2C), 129.0, 129.1 (2C), 129.8, 132.0, 134.30, 134.34, 138.7, 162.2, 162.7, 163.5. IR (neat) 3034, 2953, 1739, 1634, 1436, 1323, 1229, 1163, 1025 cm^{-1}. HRMS (ESI+) calcd for $C_{18}H_{18}NO_4$ [M+H]$^+$ 312.1230, found 312.1237.

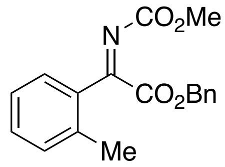

Benzyl 2-((methoxycarbonyl)imino)-2-(*m*-tolyl)acetate (7k): Colorless oil. ^{1}H NMR (400 MHz, $CDCl_3$) δ 2.32 (br, 3H), 3.68 (s, 3H), 5.31 (s, 2H), 7.23 (t, J = 7.8 Hz, 3H), 7.34-7.42 (m, 6H). ^{13}C NMR (100 MHz, DMSO-d_6) δ 19.8, 53.6, 68.4, 126.1, 128.6 (3C), 128.8 (2C), 129.9, 131.3, 131.6, 132.4, 134.4, 137.4, 161.0, 161.4, 162.4. IR (neat) 3065, 3033, 2954, 1740, 1643, 1456, 1436, 1379, 1320, 1213, 1029, 1000 cm^{-1}. HRMS (FAB+) calcd for $C_{18}H_{17}NNaO_4$ [M+Na]$^+$ 334.1055, found 334.1062.

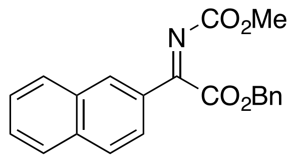

Benzyl 2-((methoxycarbonyl)imino)-2-(naphthalen-2-yl)acetate (7l): Light yellow oil. ^{1}H NMR (400 MHz, $CDCl_3$) δ 3.69 (s, 3H), 5.43 (s, 2H), 7.38-7.50 (m, 5H), 7.52 (d, J = 6.9 Hz, 1H),

7.60 (d, J = 6.9 Hz, 1H), 7.81 (d, J = 8.2 Hz, 1H), 7.85 (d, J = 7.8 Hz, 1H), 7.86 (d, J = 8.7 Hz, 1H), 8.01 (d, J = 8.7 Hz, 1H), 8.25 (s, 1H). ^{13}C NMR (100 MHz, $CDCl_3$) δ 53.8, 68.4, 124.1, 127.0, 127.9, 128.8 (3C), 128.9, 129.0, 129.2 (2C), 129.4, 129.5, 132.3, 132.5, 134.3, 135.7, 162.2, 162.7, 163.3. IR (neat) 3065, 3031, 2957, 2894, 2838, 1739, 1638, 1562, 1435, 1231, 1010 cm^{-1}. HRMS (FAB+) calcd for $C_{21}H_{18}NO_4$ $[M+H]^+$ 348.1236, found 348.1243.

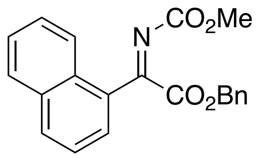

Benzyl 2-((methoxycarbonyl)imino)-2-(naphthalen-1-yl)acetate (7m): Light yellow oil. ^{1}H NMR (400 MHz, $CDCl_3$, 50 °C) δ 3.68 (s, 3H), 5.28 (s, 2H), 7.20-7.32 (m, 5H), 7.40-7.41 (m, 3H), 7.61 (d, J = 6.4 Hz, 1H), 7.83 (d, J = 7.8 Hz, 1H), 7.92 (d, J = 8.2 Hz, 1H), 8.11 (br, 1H). ^{13}C NMR (100 MHz, $CDCl_3$, 60 °C) δ 53.6, 68.5, 124.7, 124.9, 126.6, 127.6, 128.66 (6C), 128.71, 128.76, 130.7, 132.1, 133.7, 134.5, 161.6, 162.5, 163.1. IR (neat) 3035, 2953, 1738, 1642, 1436, 1313, 1227, 1177, 1101, 1026 cm^{-1}. HRMS (ESI+) calcd for $C_{21}H_{18}NO_4$ $[M+H]^+$ 348.1230, found 348.1230.

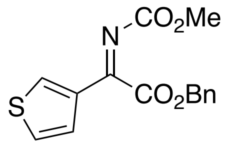

Benzyl 2-((methoxycarbonyl)imino)-2-(thiophen-3-yl)acetate (7n): Light yellow oil. ^{1}H NMR (400 MHz, $CDCl_3$) δ 3.62 (s, 3H), 5.35 (s, 2H), 7.34 (dd, J = 5.3, 3.0 Hz, 1H), 7.35-7.46 (m, 5H), 7.60 (d, J = 4.6 Hz, 1H), 8.12 (d, J = 1.8 Hz, 1H). ^{13}C NMR (100 MHz, $CDCl_3$) δ 53.6, 68.6, 126.7, 127.3, 128.8 (2C), 129.0 (3C), 134.1, 134.3, 135.4, 155.8, 161.5, 162.1. IR (neat) 3112, 2952, 1739, 1628, 1517, 1434, 1303, 1227, 1171, 1080, 1018 cm^{-1}. HRMS (FAB+) calcd for $C_{15}H_{13}NNaO_4S$ $[M+H]^+$ 326.0463, found 326.0470.

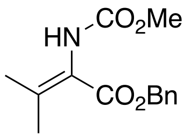

Benzyl 2-((methoxycarbonyl)amino)-3-methylbut-2-enoate (7o): Light yellow solid. ^{1}H NMR (400 MHz, $CDCl_3$) δ 1.89 (s, 3H), 2.17 (s, 3H), 3.48-3.85 (br, 3H), 5.19 (s, 2H), 5.62-6.00 (br, 1H), 7.29-7.39 (m, 5H). ^{13}C NMR (100 MHz, $CDCl_3$) δ 21.5, 22.6, 52.6, 66.7, 121.1, 128.2 (3C), 128.6 (2C), 135.9, 147.0, 155.4, 164.8. IR (KBr) 3297, 3035, 2954, 1719, 1697, 1517, 1380, 1304, 1271, 1225, 1094, 1068 cm^{-1}. M.p. 54 °C. HRMS (FAB+) calcd for $C_{14}H_{17}NNaO_4$ $[M+Na]^+$ 286.1055, found 286.1053.

18. Representative procedures for the enantioselective aza-Friedel–Crafts reaction of 2 with 7 (Table 2 and Scheme 2).

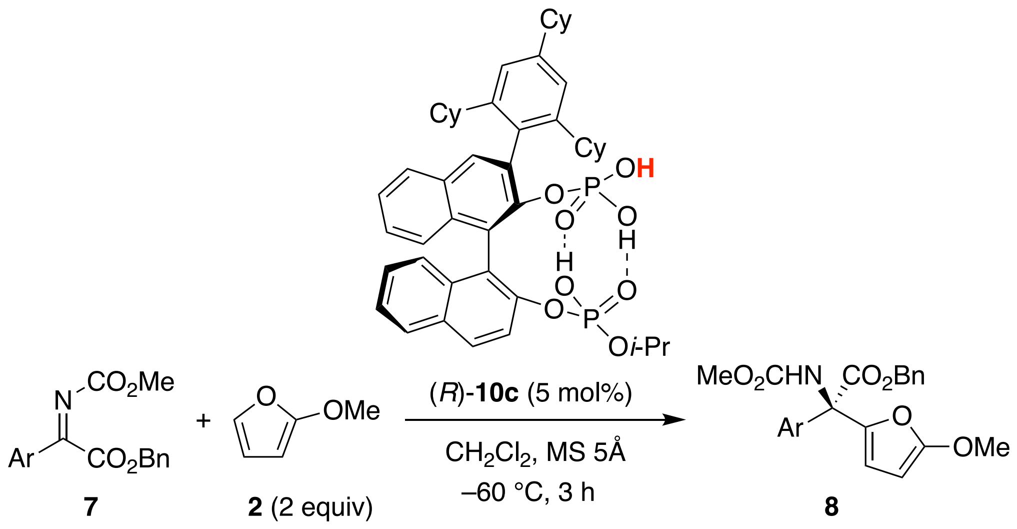

To a well-dried pyrex Schlenk tube charged with activated MS 5Å (50 mg) under a nitrogen atmosphere were added (*R*)-**10c** (8.1 mg, 0.010 mmol) in dichloromethane (1 mL) at –78 °C. After 5 min, α-ketimino ester **7** (0.20 mmol) in dichloromethane (1 mL), and 2-methoxyfuran **2** (37 μL, 0.40 mmol) were added at –78 °C. The reaction mixture was allowed to warm to –60 °C and was then stirred at that temperature for 3 h. To quench the reaction, triethylamine (0.1 mL) was added to the mixture at –60 °C, and the mixture was concentrated under reduced pressure at room temperature. The residue was purified by the silica gel column chromatography (eluent: *n*-hexane/EtOAc = 3/1 to 1/1) to give the desired product **8**. The enantiomeric purity was determined by chiral HPLC analysis.

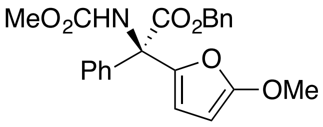

Benzyl (*R*)-2-((methoxycarbonyl)amino)-2-(5-methoxyfuran-2-yl)-2-phenylacetate (8a): –60 °C, 3 h, 93% yield, 95% ee. Coloreless oil. ^{1}H NMR (400 MHz, $CDCl_3$) δ 3.60 (s, 3H), 3.78 (s, 3H), 5.12 (d, J = 3.2 Hz, 1H), 5.16 (d, J = 12.4 Hz, 1H), 5.21 (d, J = 12.4 Hz, 1H), 6.22 (s, 1H), 6.38 (s, 1H), 7.12-7.18 (m, 2H), 7.24-7.29 (3H), 7.29-7.35 (m, 3H), 7.44-7.50 (m, 2H). ^{13}C NMR (100 MHz, $CDCl_3$) δ 52.1, 57.6, 65.3, 68.0, 80.2, 112.4, 127.3 (2C), 127.8 (2C), 128.2, 128.3 (2C), 128.41 (2C), 128.43, 135.1, 137.2, 140.6, 154.8, 161.1, 169.3. ^{19}F NMR (376 MHz, $CDCl_3$) δ –113.8. IR (neat) 3399, 2952, 1731, 1574, 1495, 1450. 1367, 1262, 1024 cm^{-1}. $[\alpha]_D^{27}$ = –2.4 (c 1.00, $CHCl_3$, 95% ee). HPLC analysis; AD-H, *n*-hexane/*i*-PrOH = 1/1, 254 nm, 0.6 mL/min, t_R = 13.6 min (minor, *S*), 17.5 min (major, *R*). HRMS (FAB+) calcd for $C_{22}H_{21}NNaO_6$ $[M+Na]^+$ 418.1267, found 418.1252.

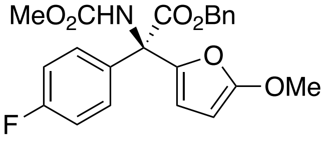

Benzyl (*R*)-2-(4-fluorophenyl)-2-((methoxycarbonyl)amino)-2-(5-methoxyfuran-2-yl)acetate (8b): –60 °C, 3 h, 96% yield, 96% ee. Coloreless oil. ^{1}H NMR (400 MHz, $CDCl_3$) δ 3.60 (s, 3H), 3.80 (s, 3H), 5.12 (d, J = 3.2 Hz, 1H), 5.17 (d, J = 12.4 Hz, 1H), 5.21 (d, J = 12.4 Hz, 1H), 6.24 (s, 1H), 6.32 (d, J = 3.2 Hz, 1H), 6.96-7.03 (m, 2H), 7.13-7.19 (m, 2H), 7.26-7.31 (m, 3H), 7.43-7.49 (m, 2H). ^{13}C NMR (100 MHz, $CDCl_3$) δ 52.2, 57.7, 64.8, 68.2, 80.2, 112.5, 115.1 (d, $J_{C–F}$ = 21.9 Hz, 2C), 127.9 (2C), 128.3, 128.5 (2C), 129.3 (d, $J_{C–F}$ = 7.6 Hz, 2C), 132.9, 134.9, 140.4, 154.8, 161.2, 162.6 (d, $J_{C–F}$ = 246.9 Hz), 169.2. ^{19}F NMR (367 MHz, $CDCl_3$) δ –113.8. IR (neat) 3409, 2952, 1734, 1615, 1576, 1507, 1456, 1367, 1262, 1024 cm^{-1}. $[\alpha]_D^{26}$ = –8.4 (*c* 1.00, $CHCl_3$, 96% ee). HPLC analysis; AD-H, *n*-hexane/*i*-PrOH = 1/1, 240 nm, 0.6 mL/min, t_R = 12.6 min (minor, *S*), 15.5 min (major, *R*). HRMS (FAB+) calcd for $C_{22}H_{21}FNO_6$ $[M+H]^+$ 414.1353, found 414.1364.

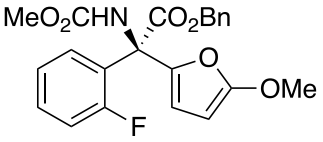

Benzyl (*R*)-2-(2-fluorophenyl)-2-((methoxycarbonyl)amino)-2-(5-methoxyfuran-2-yl)acetate (8c): –60 °C, 3 h, 90%, 97% ee. Colorless oil. ^{1}H NMR (400 MHz, $CDCl_3$) δ 3.58 (br, 3H), 3.80 (s, 3H), 5.17 (d, J = 3.2 Hz, 1H), 5.18 (d, J = 12.4 Hz, 1H), 5.28 (d, J = 12.4 Hz, 1H), 6.32 (d, J = 3.2 Hz, 1H), 6.53 (br, 1H), 7.00 (dd, J = 11.4, 8.3 Hz, 1H), 7.11 (t, J = 7.3 Hz, 1H), 7.16-7.21 (m, 2H), 7.25-7.34 (m, 4H), 7.44 (t, J = 7.8 Hz, 1H). ^{13}C NMR (100 MHz, $CDCl_3$) δ 52.2, 57.9, 62.6, 68.5, 81.1, 112.3, 115.5 (d, $J_{C–F}$ = 21.9 Hz), 123.5 (d, $J_{C–F}$ = 3.8 Hz), 128.0 (2C), 128.4, 128.5 (3C), 130.4 (d, $J_{C–F}$ = 8.6 Hz), 131.7, 135.1, 138.1, 154.6, 160.2 (d, $J_{C–F}$ = 246.0 Hz), 161.7, 169.3. ^{19}F NMR (367 MHz, $CDCl_3$) δ –114.1. IR (neat) 3410, 2953, 1734, 1614, 1573, 1489, 1456, 1369, 1262, 1038 cm^{-1}. $[\alpha]_D^{26}$ = –30.0 (*c* 1.00, $CHCl_3$, 97% ee). HPLC analysis; OD-3, *n*-hexane/*i*-PrOH = 1/1, 254 nm, 1.0 mL/min, t_R = 10.0 min (major, *R*), 16.2 min (minor, *S*). HRMS (FAB+) calcd for $C_{22}H_{20}NNaO_6$ $[M+Na]^+$ 436.1172, found 436.1185.

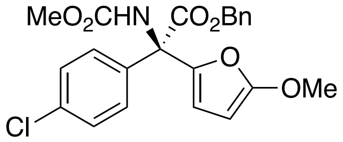

Benzyl (*R*)-2-(4-chlorophenyl)-2-((methoxycarbonyl)amino)-2-(5-methoxyfuran-2-yl)acetate (8d): –60 °C, 3 h, 96% yield, 96% ee. Coloreless oil. ^{1}H NMR (400 MHz, $CDCl_3$) δ 3.60 (s, 3H), 3.80 (s, 3H), 5.12 (d, J = 3.7 Hz, 1H), 5.17 (d, J = 12.4 Hz, 1H), 5.21 (d, J = 12.4 Hz, 1H), 6.25 (s, 1H), 6.30 (d, J = 3.2 Hz, 1H), 7.13-7.19 (m, 2H), 7.26-7.32 (m, 5H), 7.40-7.45 (m, 2H).

^{13}C NMR (100 MHz, $CDCl_3$) δ 52.2, 57.7, 64.9, 68.3, 80.3, 112.5, 127.9 (2C), 128.4 (3C), 128.5 (2C), 128.9 (2C), 134.4, 134.9, 135.7, 140.2, 154.8, 161.3, 169.0. IR (neat) 3407, 2952, 1733, 1615, 1577, 1492, 1367, 1262, 1024 cm^{-1}. $[\alpha]_D^{27}$ = –8.8 (*c* 1.00, $CHCl_3$ 96% ee). HPLC analysis; AD-H, *n*-hexane/*i*-PrOH = 1/1, 254 nm, 0.6 mL/min, t_R = 13.2 min (minor, *S*), 18.9 min (major, *R*). HRMS (FAB+) calcd for $C_{22}H_{21}ClNO_6$ $[M+H]^+$ 430.1057, found 430.1063.

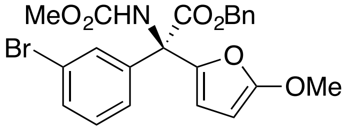

Benzyl (*R*)-2-(3-bromophenyl)-2-((methoxycarbonyl)amino)-2-(5-methoxyfuran-2-yl)acetate (8e): –60 °C, 3 h, 98% yield, 97% ee. Coloreless oil. ^{1}H NMR (400 MHz, $CDCl_3$) δ 3.60 (s, 3H), 3.79 (s, 3H), 5.12 (d, *J* = 3.2 Hz, 1H), 5.19 (s, 2H), 6.26 (br, 1H), 6.33 (br, 1H), 7.13-7.22 (m, 3H), 7.25-7.33 (m, 3H), 7.43 (t, *J* = 7.8 Hz, 2H), 7.66 (t, *J* = 1.8 Hz, 1H). ^{13}C NMR (100 MHz, $CDCl_3$) δ 52.3, 57.7, 64.9, 68.4, 80.3, 112.7, 122.4, 126.1, 127.9 (2C), 128.4, 128.5 (2C), 129.8, 130.6, 131.6, 134.8, 139.4, 140.0, 154.7, 161.3, 168.8. IR (neat) 3400, 2952, 2842, 1732, 1615, 1576, 1496, 1261, 1058, 1024 cm^{-1}. $[\alpha]_D^{29}$ = –8.4 (*c* 1.00, $CHCl_3$, 97% ee). HPLC analysis; AD-H, *n*-hexane/*i*-PrOH = 1/1, 254 nm, 0.6 mL/min, t_R = 10.6 min (minor, *S*), 12.5 min (major, *R*). HRMS (FAB+) calcd for $C_{22}H_{20}BrNNaO_6$ $[M+Na]^+$ 496.0372, found 496.0382.

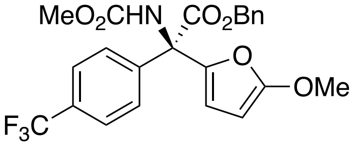

Benzyl (*R*)-2-((methoxycarbonyl)amino)-2-(5-methoxyfuran-2-yl)-2-(4-(trifluoromethyl) phenyl)acetate (8f): –60 °C, 3 h, 92% yield, 97% ee. Colorless oil. ^{1}H NMR (400 MHz, $CDCl_3$) δ 3.61 (s, 3H), 3.81 (s, 3H), 5.13 (d, *J* = 3.2 Hz, 1H), 5.18 (d, *J* = 12.4 Hz, 1H), 5.22 (d, *J* = 12.4 Hz, 1H), 6.29 (d, *J* = 3.2 Hz, 1H), 6.33 (s, 1H), 7.10-7.16 (m, 2H), 7.24-7.31 (m, 3H), 7.57 (d, *J* = 8.2 Hz, 2H), 7.63 (d, *J* = 8.7 Hz, 2H). ^{13}C NMR (100 MHz, $CDCl_3$) δ 52.3, 57.8, 65.2, 68.5, 80.4, 112.6, 124.0 (q, J_{C-F} = 270.8 Hz), 125.2 (q, J_{C-F} = 2.9 Hz, 2C), 127.9 (2C), 128.0 (2C), 128.5 (3C), 130.5 (q, J_{C-F} = 32.1 Hz), 134.8, 140.0, 141.1 154.8, 161.4, 168.7. ^{19}F NMR (376 MHz, $CDCl_3$) δ –62.6. IR (neat) 3410, 2953, 1734, 1615, 1575, 1497, 1328, 1263, 1168, 1125, 1070, 1020 cm^{-1}. $[\alpha]_D^{27}$ = –16.4 (*c* 1.00, $CHCl_3$, 97% ee). HPLC analysis; IC-3, *n*-hexane/*i*-PrOH = 1/1, 254 nm, 1.0 mL/min, t_R = 18.1 min (major, *R*), 29.5 min (minor, *S*). HRMS (FAB+) calcd for $C_{23}H_{21}F_3NO_6$ $[M+H]^+$ 464.1321, found 464.1319.

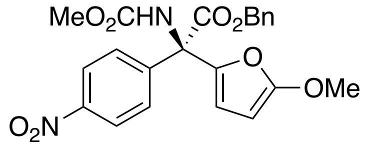

Benzyl (*R*)-2-((methoxycarbonyl)amino)-2-(5-methoxyfuran-2-yl)-2-(4-nitrophenyl)acetate (8g): –60 °C, 3 h, 95% yield, 96% ee. Yellow oil. ^{1}H NMR (400 MHz, $CDCl_3$) δ 3.61 (br, 3H), 3.81 (s, 3H), 5.14 (d, J = 3.7 Hz, 1H), 5.19 (d, J = 12.4 Hz, 1H), 5.23 (d, J = 12.4 Hz, 1H), 6.25 (d, J = 3.2 Hz, 1H), 6.37 (s, 1H), 7.13-7.21 (m, 2H), 7.27-7.32 (m, 3H), 7.69 (dt, J = 9.6, 2.3 Hz, 2H), 8.16 (dt, J = 9.6, 2.3 Hz, 2H). ^{13}C NMR (100 MHz, $CDCl_3$) δ 52.4, 57.8, 65.1, 68.8, 80.5, 112.6, 123.2 (2C), 128.1 (2C), 128.5 (2C), 128.6, 128.8 (2C), 134.5, 139.4, 144.3, 147.6, 154.8, 161.5, 168.3. IR (neat) 3400, 3031, 2953, 2841, 1732, 1615, 1574, 1521, 1496, 1350, 1262, 1112, 1024 cm^{-1}. $[\alpha]_D^{27}$ = –22.0 (c 1.00, $CHCl_3$, 96% ee). HPLC analysis; AD-H, n-hexane/i-PrOH = 1/1, 254 nm, 0.6 mL/min, t_R = 16.7 min (minor, *S*), 17.8 min (major, *R*). HRMS (FAB+) calcd for $C_{22}H_{20}N_2NaO_8$ $[M+Na]^+$ 463.1117, found 463.1120.

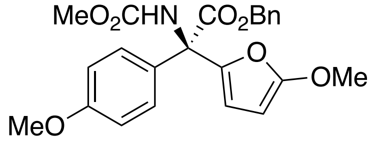

Benzyl (*R*)-2-((methoxycarbonyl)amino)-2-(5-methoxyfuran-2-yl)-2-(4-methoxyphenyl) acetate (8h): –60 °C, 6 h, 83% yield, 82% ee. Colorless oil. ^{1}H NMR (400 MHz, $CDCl_3$) δ 3.60 (br, 3H), 3.79 (s, 6H), 5.12 (d, J = 3.2 Hz, 1H), 5.16 (d, J = 12.4 Hz, 1H), 5.21 (d, J = 12.4 Hz, 1H), 6.18 (br, 1H), 6.37 (d, J = 3.2 Hz, 1H), 6.83 (d, J = 10.1 Hz, 2H), 7.14-7.20 (m, 2H), 7.25-7.32 (m, 3H), 7.38 (d, J = 10.1 Hz, 2H). ^{13}C NMR (100 MHz, $CDCl_3$) δ 52.1, 55.3, 57.6, 64.8, 68.0, 80.1, 112.3, 113.7 (2C), 127.8 (2C), 128.2, 128.4 (2C), 128.6 (2C), 129.2, 135.2, 140.8, 154.9, 159.6, 161.1, 169.5. IR (neat) 3398, 2953, 1733, 1614, 1576, 1509, 1457, 1367, 1258, 1181, 1026 cm^{-1}. $[\alpha]_D^{28}$ = –0.4 (c 1.00, $CHCl_3$, 82% ee). HPLC analysis; AD-H, n-hexane/i-PrOH = 1/1, 254 nm, 0.6 mL/min, t_R = 22.0 min (minor, *S*), 34.1 min (major, *R*). HRMS (FAB+) calcd for $C_{23}H_{23}NNaO_7$ $[M+Na]^+$ 448.1372, found 448.1367.

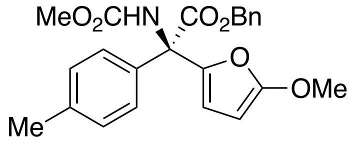

Benzyl (*R*)-2-((methoxycarbonyl)amino)-2-(5-methoxyfuran-2-yl)-2-(*p*-tolyl)acetate (8i): –60 °C, 6 h, 91% yield, 94% ee. Colorless oil. ^{1}H NMR (400 MHz, $CDCl_3$) δ 2.33 (s, 3H), 3.60 (br, 3H), 3.79 (s, 3H), 5.11 (d, J = 3.2 Hz, 1H), 5.15 (d, J = 12.4 Hz, 1H), 5.22 (d, J = 12.4 Hz, 1H), 6.20 (br, 1H), 6.38 (d, J = 2.8 Hz, 1H), 7.12 (d, J = 8.2 Hz, 2H), 7.14-7.19 (m, 2H), 7.25-7.31 (m,

3H), 7.35 (d, J = 8.2 Hz, 2H). ^{13}C NMR (100 MHz, $CDCl_3$) δ 21.1, 52.1, 57.6, 65.1, 68.0, 80.1, 112.3, 127.1 (2C), 127.8 (2C), 128.2, 128.4 (2C), 129.1 (2C), 134.3, 135.2, 138.3, 140.8, 154.8, 161.1, 169.5. IR (neat) 3412, 2952, 2840, 1733, 1615, 1576, 1496, 1456, 1367, 1262, 1060, 1024 cm^{-1}. $[\alpha]_D^{26}$ = –1.6 (*c* 1.00, $CHCl_3$, 94% ee). HPLC analysis; AD-H, *n*-hexane/*i*-PrOH = 1/1, 254 nm, 0.6 mL/min, t_R = 16.4 min (minor, *S*), 22.8 min (major, *R*). HRMS (FAB+) calcd for $C_{23}H_{23}NNaO_6$ $[M+Na]^+$ 432.1423, found 432.1421.

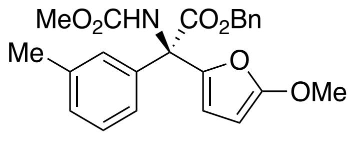

Benzyl (*R*)-2-((methoxycarbonyl)amino)-2-(5-methoxyfuran-2-yl)-2-(*m*-tolyl)acetate (8j): –60 °C, 3 h, 94% yield, 95% ee. Coloreless oil. ^{1}H NMR (400 MHz, $CDCl_3$) δ 2.30 (s, 3H), 3.61 (br, 3H), 3.80 (s, 3H), 5.12 (d, J = 3.2 Hz, 1H), 5.20 (s, 2H), 6.16 (br, 1H), 6.40 (d, J = 2.8 Hz, 1H), 7.11 (d, J = 7.3 Hz, 1H), 7.14-7.29 (m, 8H). ^{13}C NMR (100 MHz, $CDCl_3$) δ 21.6, 52.2, 57.7, 65.2, 68.0, 80.1, 112.4, 124.4, 127.8, 129.9 (2C), 128.2, 128.3, 128.4 (2C), 129.3, 135.3, 137.2, 138.1, 140.7, 154,9, 161.1, 169.4. IR (neat) 3402, 2951, 1731, 1615, 1575, 1495, 1367, 1262, 1213, 1024 cm^{-1}. $[\alpha]_D^{26}$ = –2.0 (*c* 1.00, $CHCl_3$, 95% ee). HPLC analysis; AD-H, *n*-hexane/*i*-PrOH = 1/1, 254 nm, 0.6 mL/min, t_R = 11.5 min (minor, *S*), 15.5 min (major, *R*). HRMS (FAB+) calcd for $C_{23}H_{23}NNaO_6$ $[M+Na]^+$ 432.1423, found 432.1422.

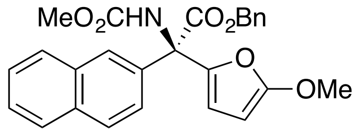

Benzyl (*R*)-2-((methoxycarbonyl)amino)-2-(5-methoxyfuran-2-yl)-2-(naphthalen-2-yl)acetate (8l): –60 °C, 12 h, 96% yield, 95% ee. Coloreless oil. ^{1}H NMR (400 MHz, $CDCl_3$) δ 3.61 (s, 3H), 3.79 (s, 3H), 5.15 (d, J = 3.2 Hz, 1H), 5.17 (d, J = 12.4 Hz, 1H), 5.22 (d, J = 12.4 Hz, 1H), 6.33 (s, 1H), 6.42 (s, 1H), 7.12-7,17 (m, 2H), 7.19-7.27 (m, 3H), 7.43-7.51 (m, 2H), 7.58 (dd, J = 8.7, 2.3 Hz, 1H), 7.73-7.83 (m, 3H), 7.92 (d, J = 1.4 Hz, 1H). ^{13}C NMR (100 MHz, $CDCl_3$) δ 52.3, 57.7, 65.5, 68.2, 80.3, 112.6, 124.9, 126.3, 126.6, 126.8, 127.5, 127.9 (2C), 128.1, 128.3, 128.4 (2C), 128.6, 132.9, 133.1, 134.7, 135.1, 140.6, 155.0, 161.2, 169.3. IR (neat) 3406, 2952, 1731, 1614, 1575, 1496, 1368, 1262, 1024 cm^{-1}. $[\alpha]_D^{25}$ = –1.6 (*c* 1.00, $CHCl_3$, 95% ee). HPLC analysis; AD-H, *n*-hexane/*i*-PrOH = 1/1, 254 nm, 0.6 mL/min, t_R = 18.4 min (minor, *S*), 28.3 min (major, *R*). HRMS (FAB+) calcd for $C_{26}H_{23}NNaO_6$ $[M+Na]^+$ 468.1423, found 468.1429.

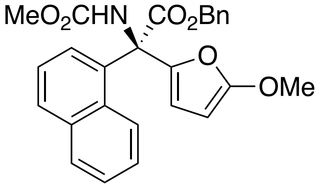

Benzyl (*R*)-2-((methoxycarbonyl)amino)-2-(5-methoxyfuran-2-yl)-2-(naphthalen-1-yl)acetate (8m): –60 °C, 12 h, 85% yield, 90% ee. White solid. ^{1}H NMR (400 MHz, $CDCl_3$) δ 3.52 (br, 3H), 3.81 (s, 3H), 5.13 (d, *J* = 12.4 Hz, 1H), 5.19 (d, *J* = 12.4 Hz, 1H), 5.20 (d, *J* = 3.2 Hz, 1H), 6.36 (d, *J* = 3.2 Hz, 1H), 6.42 (s, 1H), 6.96 (d, *J* = 7.4 Hz, 2H), 7.15 (t, *J* = 7.3 Hz, 2H), 7.21 (t, *J* = 7.3 Hz, 1H), 7.30 (td, *J* = 7.8, 1.4 Hz, 1H), 7.35-7.43 (m, 3H), 7.82 (d, *J* = 8.2 Hz, 1H), 7.85 (d, *J* = 8.2 Hz, 1H), 7.94 (d, *J* = 8.2 Hz, 1H). ^{13}C NMR (100 MHz, $CDCl_3$) δ 52.2, 57.8, 66.3, 68.4, 80.9, 112.2, 123.6, 124.9, 125.4, 126.5, 128.0 (2C), 128.2, 128.3 (2C), 128.4, 129.4, 129.7, 130.5, 133.8, 134.3, 134.7, 139.9, 154.7, 161.6, 170.5. IR (KBr) 3400, 2942, 1728, 1614, 1570, 1496, 1367, 1262, 1037 cm^{-1}. M.p. 38-50 °C (decomposition). $[\alpha]_D^{25}$ = –50.0 (*c* 1.00, $CHCl_3$, 90% ee). HPLC analysis; OD-3, *n*-hexane/*i*-PrOH = 1/1, 254 nm, 1.0 mL/min, t_R = 9.0 min (major, *R*), 22.9 min (minor, *S*). HRMS (FAB+) calcd for $C_{26}H_{23}NNaO_6$ $[M+Na]^+$ 468.1423, found 468.1429.

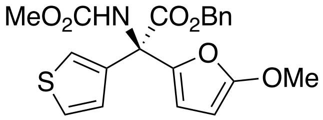

Benzyl (*R*)-2-((methoxycarbonyl)amino)-2-(5-methoxyfuran-2-yl)-2-(thiophen-3-yl)acetate (8n): –60 °C, 3 h, 96% yield, 87% ee. Coloreless oil. ^{1}H NMR (400 MHz, $CDCl_3$) δ 3.61 (s, 3H), 3.80 (s, 3H), 5.11 (d, *J* = 3.2 Hz, 1H), 5.20 (d, *J* = 12.8 Hz, 1H), 5.24 (d, *J* = 12.4 Hz, 1H), 6.26 (s, 1H), 6.29 (d, *J* = 3.2 Hz, 1H), 7.14 (dd, *J* = 5.0, 1.4 Hz, 1H), 7.17-7.21 (m, 2H), 7.25 (dd, *J* = 5.0, 3.2 Hz, 1H), 7.27-7.32 (m, 3H), 7.36 (dd, *J* = 3.2, 1.4 Hz, 1H). ^{13}C NMR (100 MHz, $CDCl_3$) δ 52.2, 57.7, 62.7, 68.1, 80.3, 111.9, 124.3, 125.5, 127.3, 127.8 (2C), 128.3, 128.5 (2C), 135.1, 137.9, 140.6, 154.8, 161.1, 169.0. IR (neat) 3400, 2952, 2839, 1732, 1615, 1577, 1497, 1365, 1262, 1059, 1024 cm^{-1}. $[\alpha]_D^{27}$ = –7.6 (*c* 1.00, $CHCl_3$, 87% ee). HPLC analysis; AD-H, *n*-hexane/*i*-PrOH = 1/1, 254 nm, 0.6 mL/min, t_R = 14.3 min (minor, *S*), 16.2 min (major, *R*). HRMS (FAB+) calcd for $C_{20}H_{19}NNaO_6S$ $[M+Na]^+$ 424.0831, found 424.0828.

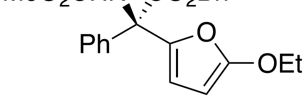

Benzyl (*R*)-2-(5-ethoxyfuran-2-yl)-2-((methoxycarbonyl)amino)-2-phenylacetate (8p): –60 °C, 12 h, 94% yield, 94% ee. Colorless oil. ^{1}H NMR (400 MHz, $CDCl_3$) δ 1.37 (t, *J* = 7.1 Hz, 3H), 3.60 (br, 3H), 4.02 (q, *J* = 7.2 Hz, 2H), 5.12 (d, *J* = 3.2 Hz, 1H), 5.16 (d, *J* = 12.4 Hz, 1H), 5.22 (d, *J* = 12.4 Hz, 1H), 6.22 (s, 1H), 6.37 (d, *J* = 2.8 Hz, 1H), 7.12-7.18 (m, 2H), 7.25-7.36 (m, 6H), 7.45-7.51 (m, 2H). ^{13}C NMR (100 MHz, $CDCl_3$) δ 14.6, 52.2, 65.3, 66.8, 68.0, 81.1, 112.4, 127.3

(2C), 127.8 (2C), 128.2, 128.3 (2C), 128.4 (3C), 135.1, 137.2, 140.4, 154.8, 160.2, 169.4. IR (neat) 3410, 2982, 2953, 1734, 1612, 1572, 1496, 1450, 1261, 1026 cm^{-1}. $[\alpha]_D^{26}$ = –7.6 (*c* 1.00, $CHCl_3$, 94% ee). HPLC analysis; AD-H, *n*-hexane/*i*-PrOH = 1/1, 230 nm, 0.6 mL/min, t_R = 11.7 min (minor, *S*), 12.8 min (major, *R*). HRMS (FAB+) calcd for $C_{23}H_{23}NNaO_6$ $[M+Na]^+$ 432.1423, found 432.1422.

Determination of absolute stereochemistry of 8e:

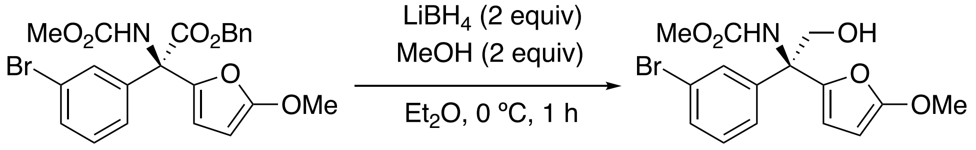

8e **S30**, 83%

To a solution of **8e** (76.0 mg, 0.16 mmol, 97% ee) in diethyl ether (1.6 mL) was added methanol (13 μL, 0.32 mmol) followed by lithium borohydride (6.9 mg, 0.32 mmol) under nitrogen atmosphere at –78 °C. The mixture was stirred at 0 °C for 1 h. Saturated NH_4Cl aqueous solution (2 mL) was then added to the mixture. The mixture was extracted with diethyl ether (5 mL × 2), and the combined organic layer was dried over Na_2SO_4. The organic phase was concentrated under reduced pressure, and purified by silica gel column chromatography (eluent: *n*-hexane:EtOAc = 2:1 to 1:1), to give the desired product **S30** in 83% yield (49.1 mg). White soild. ^{1}H NMR (400 MHz, $CDCl_3$) δ 3.68 (s, 4H), 3.83 (s, 3H), 4.14 (m, 1H), 4.25 (dd, *J* = 12.1, 6.6 Hz, 1H) 5.12 (d, *J* = 3.2 Hz, 1H), 5.72 (s, 1H), 6.19 (d, *J* = 3.2 Hz, 1H), 7.18-7.28 (m, 2H), 7.41-7.47 (m, 2H). ^{13}C NMR (100 MHz, $CDCl_3$) δ 52.6, 57.8, 63.3, 68.3, 80.0, 110.8, 122.6, 125.4, 129.8, 130.0, 131.1, 142.8 (2C), 156.5, 161.4. IR (KBr) 3232, 3073, 2953, 1690, 1615, 1567, 1439, 1370, 1283, 1261, 1098, 1047, 1021 cm^{-1}. M.p. was not available due to decomposition. $[\alpha]_D^{27}$ = +4.0 (*c* 1.00, $CHCl_3$, 97% ee). HRMS (FAB+) calcd for $C_{15}H_{16}BrNNaO_5$ $[M+Na]^+$ 392.0110, found 392.0101.

Crystal data of S30 (Fig. S10): Compound **S30** was recrystallized in diethyl ether for X-ray analysis. Formula $C_{15}H_{16}BrNO_5$, colorless, crystal dimensions 0.35 × 0.30 × 0.25 mm^3, monoclinic, space group *P2₁* (#4), *a* = 5.8577(16) Å, *b* = 14.389(4) Å, *c* = 9.621(3) Å, α = 90.00 °, β = 94.654(6) °, γ = 90.00 °, *V* = 808.2(4) Å^3, *Z* = 2, ρ_{calc} = 1.521 g cm^{-3}, F(000) = 376, μ(MoKα)= 2.565 mm^{-1}, *T* = 103 K. 6877 reflections collected, 3178 independent reflections with $I > 2\sigma(I)$ ($2\theta_{max}$ = 27.492 °), and 217 parameters were used for the solution of the structure. The non-hydrogen atoms were refined anisotropically. Flack x = 0.002(5). R_1 = 0.0248 and wR_2 = 0.0504. GOF = 0.826. Crystallographic data for the structure reported in this paper have been deposited with the Cambridge Crystallographic Data Centre as supplementary publication no. CCDC-1834631. Copies of the data can be obtained free of charge on application to CCDC, 12 Union Road, Cambridge CB2 1EZ, UK [Web page: http://www.ccdc.cam.ac.uk/].

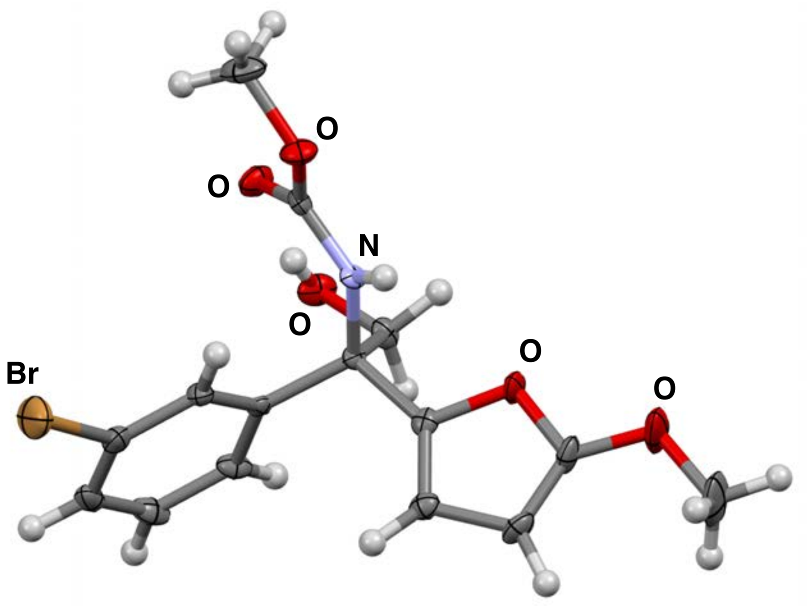

Fig. S10 ORTEP drawing of (*R*)-**S30**.

19. Optimization of catalysts, protecting groups on substrates, and reaction temperature in the reaction of 2 with 7a.

Screening of the chiral catalysts in the probe reaction of **2** with **7a** is summarized in Scheme S7. The catalytic activities of chiral BINOL-derived phosphoric acids, such as (*R*)-**4b** and (*R*)-**4c** (i.e., TRIP), had no effect on either the yield or the enantioselectivity, and a prolonged reaction time (24 h) was needed. Chiral phosphoramide (*R*)-**S19** was also ineffective. Our chiral C_2-symmetric bis(phosphoric acid)s (*R*)-**5a** and (*R*)-**5b** showed better catalytic activities (12 h) than (*R*)-**4b**, (*R*)-**4c**, and (*R*)-**S19**, although the enantioselectivity was still low. Mono-methyl ester catalyst (*R*)-**6a** showed a similar result to (*R*)-**5a** and (*R*)-**5b**. In contrast, chiral C_1-symmetric bis(phosphoric acid)s (*R*)-**10b** and (*R*)-**10c** showed much better catalytic activity, and the reactions were finished within 3 h. In particular, **8a** was obtained in 93% yield with 95% ee within 3h when we used (*R*)-**10c**. Based on these results, we selected (*R*)-**10c** as an optimized catalyst for this reaction.

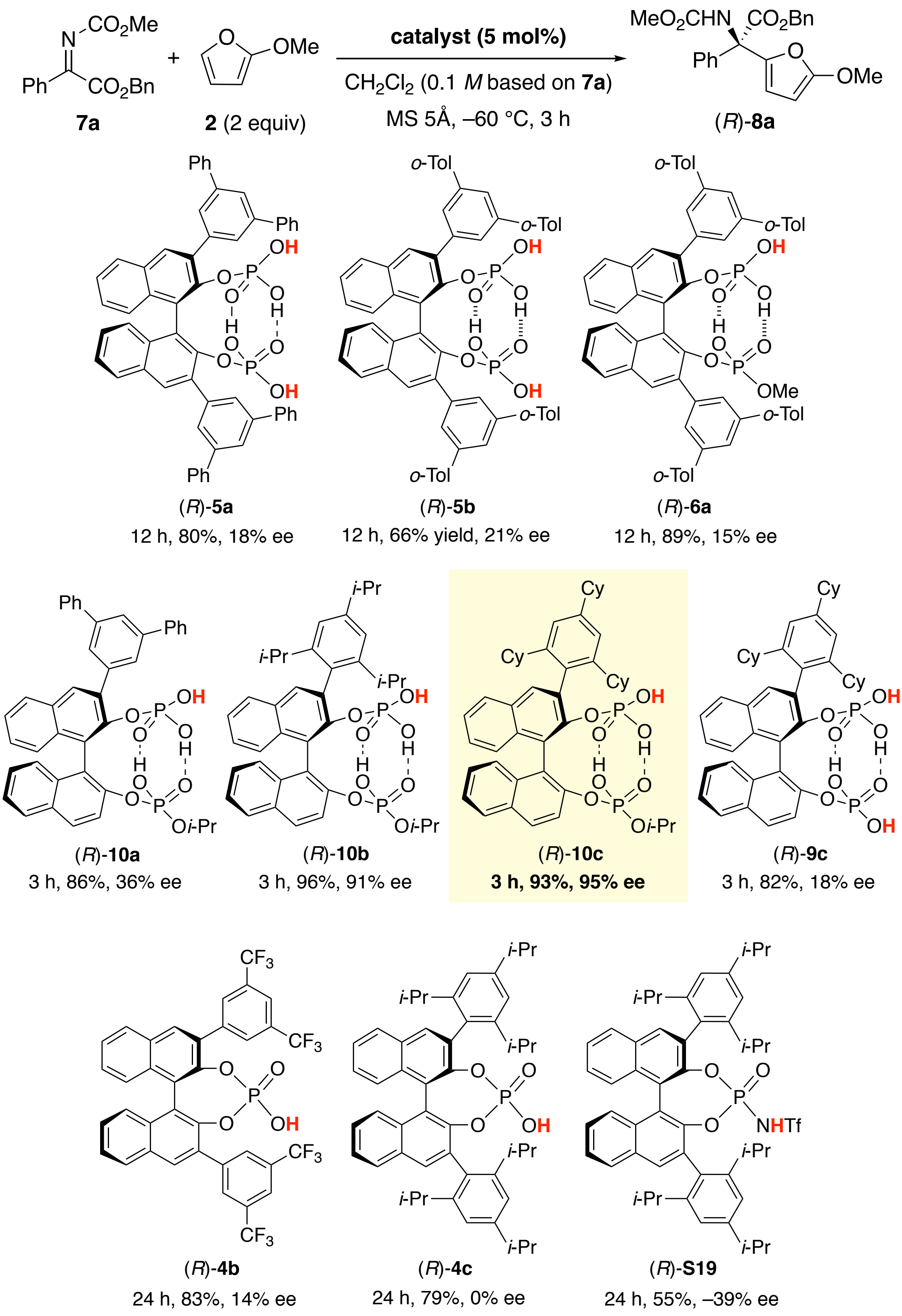

Scheme S7 Screening of the catalysts in the probe reaction of **2** with **7a**.

Next, the protecting groups of the substrates **7** were optimized (Table S6). Here, we used unoptimized chiral bis(phosphoric acid) catalyst with *n*-Pr protection. As a result, for *N*-CO_2Me-substrates, CO_2Bn (Cbz) (entry 4) was much better than CO_2Et (entry 1), CO_2*i*-Pr (entry 2), and CO_2*t*-Bu (Boc) (entry 3). Moreover, for Cbz-substrates, *N*-CO_2Me (entry 4) was much better than *N*-Cbz (entry 5) and *N*-Boc (entry 6).

Table S6 Optimization of the protecting groups of the substrates **7**.

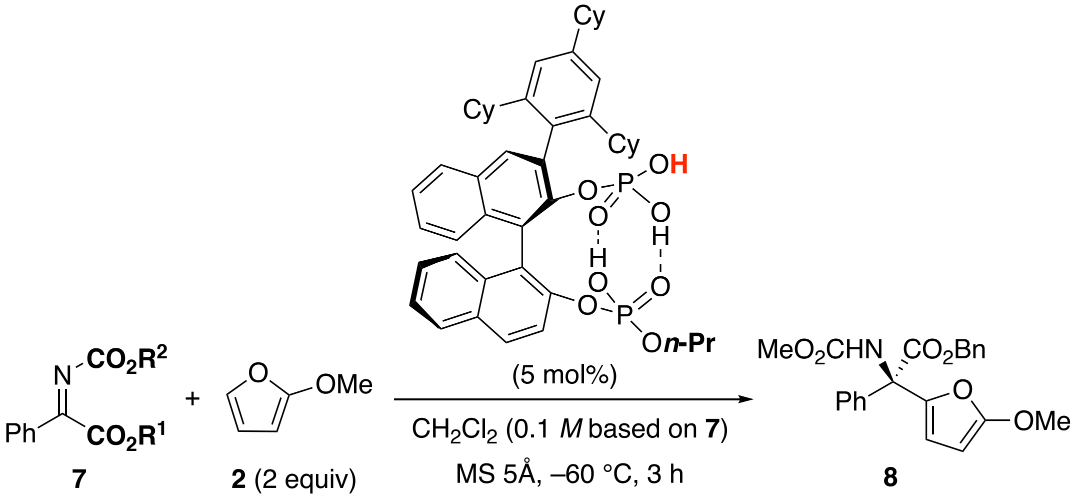

Entry	CO_2R^1	*N*-CO_2R^2	Yield (%)	ee (%)
1	CO_2Et	*N*-CO_2Me	83	67
2	CO_2*i*-Pr	*N*-CO_2Me	89	69
3	CO_2*t*-Bu (Boc)	*N*-CO_2Me	78	72
4	**CO_2Bn (Cbz)**	***N*-CO_2Me**	**89**	**92**
5	Cbz	*N*-Cbz	76	19
6	Cbz	*N*-Boc	83	0

Next, the ester moiety of the catalysts was optimized in a probe reaction of **2** with **7a** (Table S7). Without protection, the enantioselectivity of **8a** was low (entry 1). In contrast, either catalyst with Me (entry 2), *n*-Pr (entry 3), or *i*-Pr (entry 4) protection was effective, and **8a** was obtained in high yields with high enantioselectivities (92–95% ee). In particular, the catalyst with the *i*-Pr moiety (i.e., (*R*)-**10c**, entry 4) slightly more effective (95% ee) than the others.

Table S7 Optimization of the ester moiety of the catalysts.

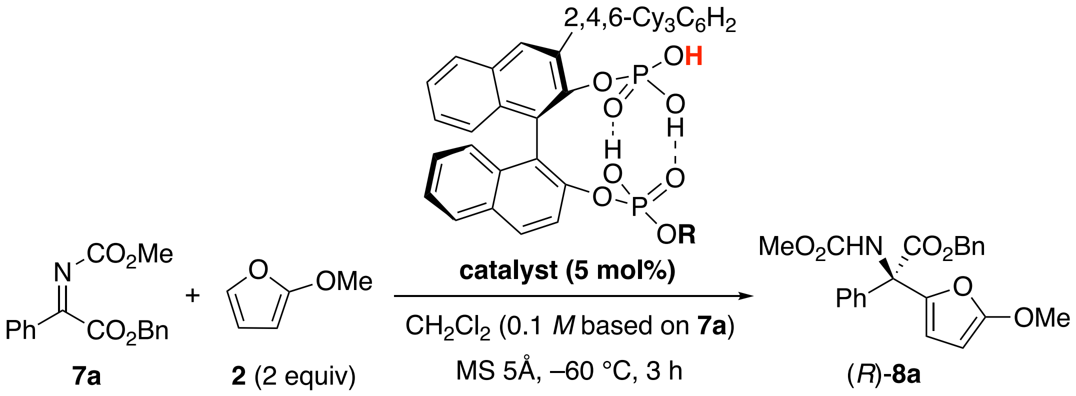

Entry	R	Yield (%)	ee (%)
1	H [(*R*)-**9c**]	82	18
2	Me	92	92
3	*n*-Pr	89	92
4	***i*-Pr** [(*R*)-**10c**]	**93**	**95**

Next, the reaction temperature was examined in an unoptimized probe reaction (Table S8). At –78 °C, the reaction proceeded sluggishly, and the product was obtained in 79% yield with 70% ee (entry 1). At –40 °C, the reaction proceeded very smoothly, although the enantioselectivity was slightly reduced (64% ee) (entry 3). In contrast, at –60 °C, the reaction proceeded smoothly, and the product was obtained in 78% yield with 72% ee (entry 2). Based on these results, we set the temperature at –60 °C.

Table S8 Optimization of the reaction temperature.

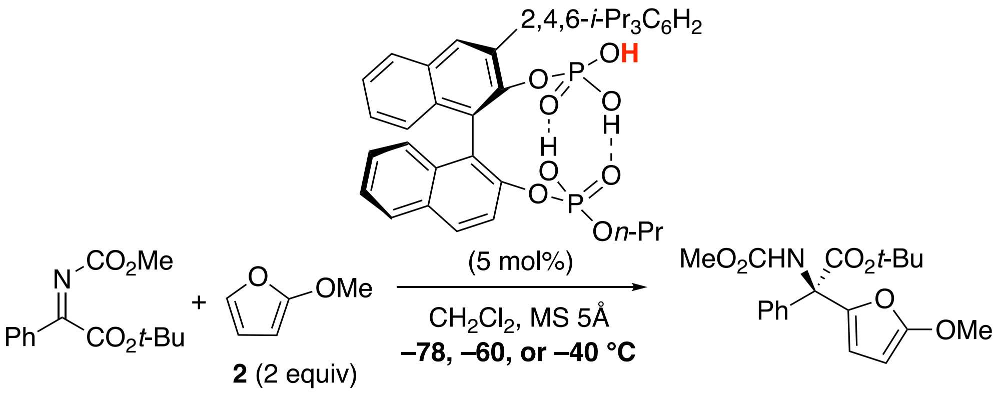

Entry	Temperature (°C)	Reaction time [h]	Yield (%)	ee (%)
1	–78	24	79	70
2	**–60**	**12**	**78**	**72**
3	–40	1	87	64

20. Preparation of (*R*)-S32 and the control experiments.

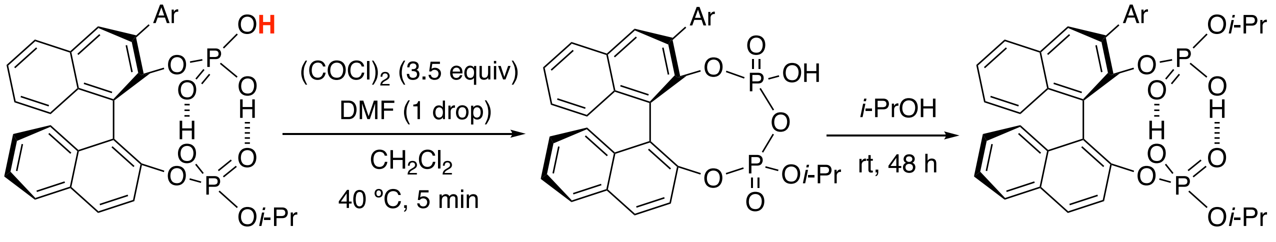

(*R*)-**10c** (Ar = 2,4,6-$Cy_3C_6H_2$) (*R*)-**S31** (*R*)-**S32**

To a solution of (*R*)-**10c** (8.1 mg, 0.010 mmol) in dichloromethane (0.2 mL), one drop of *N*,*N*-dimethylformamide was added at room temperature. Then oxalyl chloride (3.0 μL, 0.035 mmol) was added at room temperature, and the mixture was warmed to 40 °C. The mixture was stirred at 40 °C for 5 min. Volatiles were removed *in vacuo* under heat conditions (ca. 40–50 °C). The obtained (*R*)-**S31** was used in the next step without further purification. (*R*)-**S31** was dissolved in methanol (2 mL) and the solution was stirred at room temperature for 4 h. Excess mthanol was then removed *in vacuo*. The obtained product was dissolved in toluene (2 mL), and the volatiles were thoroughly removed under reduced pressure to give (*R*)-**S32** as light brown solid. (80% yield (ca. 90% purity, (*R*)-**10c** was involved), 6.8 mg).

Diisopropyl ((*R*)-3-(2,4,6-tricyclohexylphenyl)-[1,1'-binaphthalene]-2,2'-diyl) bis(hydrogen phosphate) ((*R*)-S32): ca. 90% purity (Impurity is inseparable (*R*)-**10c**, which might be generated by the reaction of (*R*)-**S31** with adventitious water.). Light brown solid. ^{1}H NMR (400 MHz, THF-d_8) δ 0.69 (d, *J* = 6.4 Hz, 3H), 0.73 (d, *J* = 6.4 Hz, 3H), 0.85-1.80 (m, 23H), 0.96 (d, *J* = 6.0 Hz, 3H), 0.99 (d, *J* = 6.0 Hz, 3H), 1.81-1.98 (m, 5H), 2.05-2.08 (m, 2H), 2.43-2.61 (m, 3H), 3.27 (m, 1H), 4.31 (m, 1H), 6.52 (br, 2H), 7.05 (s, 1H), 7.12 (s, 1H), 7.23-7.45 (m, 6H), 7.83 (s, 1H), 7.88 (d, *J* = 8.2 Hz, 1H), 7.90-7.94 (m, 1H), 7.93 (d, *J* = 8.2 Hz, 1H), 8.02 (d, *J* = 9.2 Hz, 1H). ^{13}C NMR (100 MHz, THF-d_8) Many peaks overlapped and are splited. δ 23.2, 23.3, 23.4, 23.5, 23.7, 23.8, 26.9, 27.0, 27.1, 27.6, 27.7, 27.8, 27.9, 30.5, 33.6, 33.7, 35.4, 35.5, 36.0, 37.8, 42.4, 42.6, 45.8, 72.3, 73.0, 73.1, 121.3, 122.3, 123.3, 125.1, 125.2, 125.5, 126.2, 126.3, 126.7, 127.1, 127.2, 128.5, 128.8, 130.6, 131.8, 132.0, 132.7, 133.6, 133.7, 134.3, 146.7, 147.0, 147.1, 147.9, 148.0, 149.1, 149.2. ^{31}P NMR (160 MHz, THF-d_8) δ –7.01, –4.83. IR (KBr) 3644, 3313, 2926, 2851, 1729, 1602, 1509, 1468, 1448, 1235, 1031, 997 cm^{-1}. M.p. 229-253 °C (decomposition). $[\alpha]_D^{23}$ = +186.0 (*c* 1.00, $CHCl_3$). HRMS (FAB+) calcd for $C_{50}H_{63}O_8P_2$ $[M+H]^+$ 853.3998, found 853.4006.

Summary of the reaction with the use of (*R*)-**9c**, (*R*)-**10c**, and (*R*)-**S32** is shown in Scheme S8.

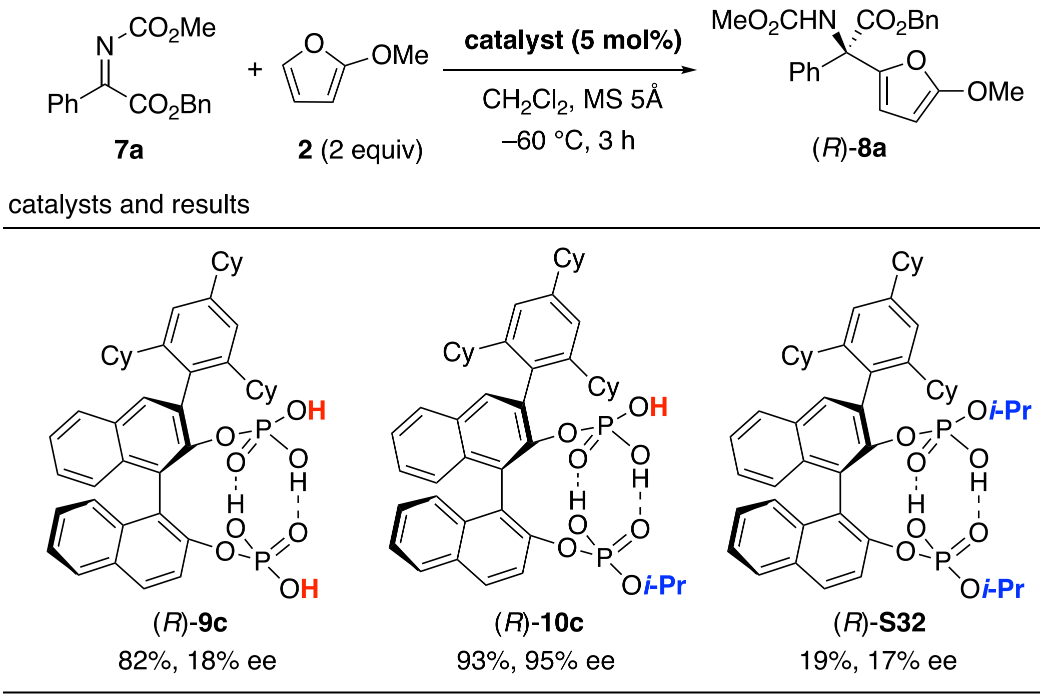

Scheme S8 Role of Brønsted acid in the catalysts.

21. Transformation of 3b to 12–14 by selective reduction (Scheme 3a).

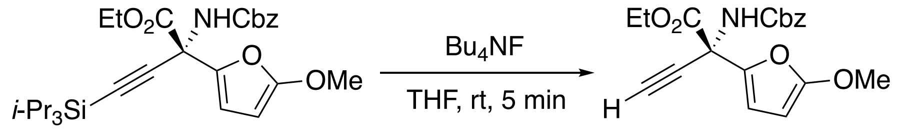

Ethyl (*S*)-2-(((benzyloxy)carbonyl)amino)-2-(5-methoxyfuran-2-yl)but-3-ynoate (11): THF (24 mL) was added to a two-necked round bottom flask with (*S*)-**3b** (1.04 g, 2.02 mmol, 91% ee), and the solution was stirred at room temperature. Tetrabuthylammonium fluoride (1.0 *M* in THF, 2.4 mL, 2.4 mmol) was added to the solution, and the mixture was stirred at room temperature for 5 min. The reaction mixture was passed through short silica gel with *n*-hexane and ethyl acetate (2:1). The filtrate was concentrated under reduced pressure, and the resultant residue was purified by silica gel column chromatography (eluent: *n*-hexane:EtOAc = 5:1 to 2:1) to give the desired product **11** as yellow oil (724 mg, >99% yield). A trace amount of EtOAc and Et_2O remained. ^{1}H NMR (400 MHz, $CDCl_3$) δ 1.24 (t, *J* = 7.3 Hz, 3H), 2.62 (s, 1H), 3.82 (s, 3H), 4.29 (br, 2H), 5.09 (d, *J* = 12.4 Hz, 1H), 5.14 (d, *J* = 4.1 Hz, 1H), 5.15 (d, *J* = 12.4 Hz, 1H), 6.13 (br, 1H), 6.56 (br, 1H) 7.29-7.36 (m, 5H). ^{13}C NMR (100 MHz, $CDCl_3$) δ 13.7, 55.8, 57.6, 63.5, 66.8, 73.1, 77.9, 80.7, 111.7, 128.0 (3C), 128.3 (2C), 135.9, 137.6, 153.8, 161.5, 166.3. IR (neat) 3413, 3292, 2979, 2898, 2124, 1758, 1614, 1574, 1504, 1369, 1020 cm^{-1}. $[\alpha]_D^{23}$ = +14.4 (*c* 1.00, $CHCl_3$, 91% ee). HRMS (FAB+) calcd for $C_{19}H_{19}NNaO_6$ $[M+Na]^+$ 380.1110, found 380.1103.

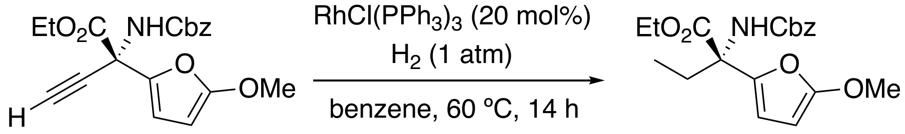

Ethyl (*S*)-2-(((benzyloxy)carbonyl)amino)-2-(5-methoxyfuran-2-yl)butanoate (12): To a round bottom flask with **11** (129.7 mg, 0.363 mmol) and chlorotris(triphenylphosphine)rhodium(I) (Wilkinson's catalyst) (37.0 mg, 0.040 mmol) was added benzene (10 mL) under a nitrogen atmosphere. The flask was purged with hydrogen with a balloon (1 atm). The mixture was stirred at 60 °C for 14 h. The reaction mixture was passed through short silica gel with *n*-hexane and ethyl acetate (3:1). The solution was concentrated under reduced pressure, and the resultant residue was purified by silica gel column chromatography (eluent: *n*-hexane:EtOAc = 9:1 to 3:1) to give the desired product **12** as colorless oil (131.1 mg, >99% yield). ^{1}H NMR (400 MHz, $CDCl_3$) δ 0.84 (t, *J* = 7.3 Hz, 3H), 1.21 (t, *J* = 7.3 Hz, 3H), 2.28 (m, 1H), 2.65 (br, 1H), 3.79 (s, 3H), 4.11-4.30 (m, 2H), 5.01 (d, *J* = 12.0 Hz, 1H), 5.08 (d, *J* = 12.0 Hz, 1H), 5.09 (d, *J* = 2.7 Hz, 1H), 6.18 (br, 1H), 6.25 (br, 1H), 7.27-7.38 (m, 5H). ^{13}C NMR (100 MHz, $CDCl_3$) δ 7.8, 14.0, 25.5, 57.6, 62.3, 62.4, 66.4, 80.3, 108.9, 127.9 (2C), 128.0, 128.5 (2C), 136.5, 141.7, 153.9, 160.9, 170.6. IR (neat) 3420, 2978, 1726, 1616, 1578, 1496, 1369, 1304, 1251, 1069, 1022 cm^{-1}. $[\alpha]_D^{24}$ =

+46.4 (*c* 1.00, $CHCl_3$, 91% ee). HRMS (FAB+) calcd for $C_{19}H_{23}NNaO_6$ $[M+Na]^+$ 384.1423, found 384.1423.

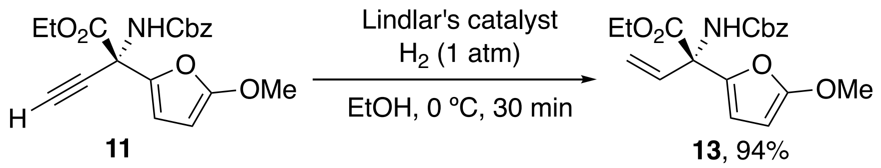

Ethyl (*S*)-2-(((benzyloxy)carbonyl)amino)-2-(5-methoxyfuran-2-yl)but-3-enoate (13): To a round bottom flask with **11** (79.8 mg, 0.223 mmol) and Lindlar's catalyst (120 mg, 150 w/w%) was added ethanol (2 mL). The flask was purged with hydrogen with a balloon (1 atm). The mixture was stirred at 0 °C for 30 min. The reaction mixture was passed through a pad of Celite with diethyl ether (20 mL). The solution was concentrated under reduced pressure, and the resultant residue was purified by silica gel column chromatography (eluent: *n*-hexane:EtOAc = 6:1 to 2:1) to give the desired product **13** as colorless oil (75.3 mg, 94% yield). ^1H NMR (400 MHz, $CDCl_3$) δ 1.21 (t, J = 6.9 Hz, 3H), 3.81 (s, 3H), 4.22 (br, 2H), 5.03 (d, J = 12.4 Hz, 1H), 5.10 (d, J = 2.7 Hz, 1H), 5.11 (d, J = 12.4 Hz, 1H), 5.38 (d, J = 10.5 Hz, 1H), 5.39 (d, J = 17.4 Hz, 1H), 6.14 (br, 1H), 6.26 (br, 1H), 6.40 (dd, J = 17.2, 10.5 Hz, 1H), 7.30-7.40 (m, 5H). ^{13}C NMR (100 MHz, $CDCl_3$) δ 13.9, 57.7, 62.7, 62.9, 66.7, 80.4, 110.6, 117.1, 128.1 (3C), 128.5 (2C), 133.2, 136.4, 140.2, 154.0, 161.3, 169.2. IR (neat) 3414, 2940, 1731, 1614, 1576, 1496, 1386, 1259, 1023 cm^{-1}. $[\alpha]_D^{26}$ = +12.8 (*c* 1.00, $CHCl_3$, 91% ee). HRMS (FAB+) calcd for $C_{19}H_{21}NNaO_6$ $[M+Na]^+$ 382.1267, found 382.1283.

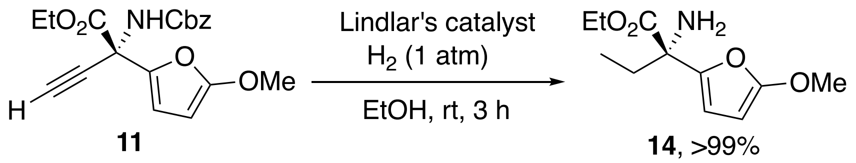

Ethyl (*S*)-2-amino-2-(5-methoxyfuran-2-yl)butanoate (14): To a round bottom flask with **11** (198.9 mg, 0.557 mmol) and Lindlar's catalyst (300 mg, 150 w/w%) was added ethanol (5.6 mL). The flask was purged with hydrogen with a balloon (1 atm). The mixture was stirred at room tempurature for 2 h. The reaction mixture was passed through a pad of Celite with diethyl ether (80 mL). The solution was concentrated under reduced pressure, and the resultant residue was purified by silica gel column chromatography (eluent: *n*-hexane:EtOAc = 7:3 to 1:1) to give the desired product **14** as colorless oil (126.5 mg, >99% yield). ^1H NMR (400 MHz, $CDCl_3$) δ 0.92 (t, J = 7.3 Hz, 3H), 1.25 (t, J = 7.3 Hz, 3H), 1.89 (br, 2H), 1.96 (m, 1H), 2.11 (m, 1H), 3.81 (s, 3H), 4.12-4.26 (m, 2H), 5.07 (d, J = 3.2 Hz, 1H), 6.14 (d, J = 3.2 Hz, 1H). ^{13}C NMR (100 MHz, $CDCl_3$) δ 7.9, 14.1, 30.3, 57.6, 60.7, 61.5, 79.9, 106.9, 145.7, 160.9, 173.6. IR (neat) 3391, 2977, 2941, 1733, 1615, 1578, 1458, 1367, 1261, 1230, 1056, 1021 cm^{-1}. $[\alpha]_D^{27}$ = +29.2 (*c* 1.00, $CHCl_3$, 91% ee). HRMS (FAB+) calcd for $C_{11}H_{17}NNaO_4$ $[M+Na]^+$ 250.1055, found 250.1061.

22. Transformation of 14 to γ-butenolide 16 (Scheme 3a).

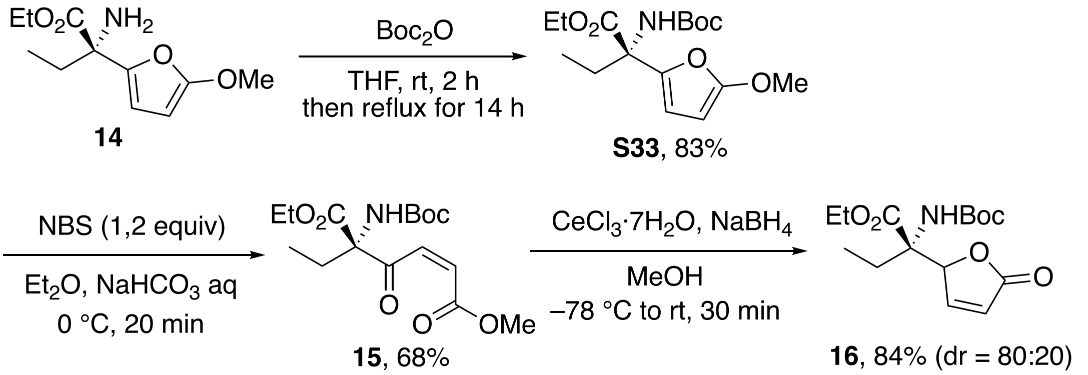

Ethyl (*S*)-2-((*tert*-butoxycarbonyl)amino)-2-(5-methoxyfuran-2-yl)butanoate (S33): S33 was synthesized based on the literature procedure.[20] THF (0.14 mL) was added to a pyrex Schlenk tube with **14** (61.1 mg, 0.268 mmol, 91% ee), and the solution was stirred at room temperature. Di-*tert*-butyl dicarbonate (61 μL, 0.282 mmol) was added to the solution, and the mixture was stirred at reflux temperature for 14 h. Volatiles were removed at room temperature under reduced pressure, and the resultant residue was purified by silica gel column chromatography (eluent: *n*-hexane:EtOAc = 9:1 to 7:3) to give the desired product **S33** as colorless oil (72.8 mg, 83% yield). ^{1}H NMR (400 MHz, $CDCl_3$) δ 0.84 (t, J = 7.3 Hz, 3H), 1.23 (t, J = 7.3 Hz, 3H), 1.40 (s, 9H), 2.27 (m, 1H), 2.55 (br, 1H), 3.81 (s, 3H), 4.13-4.27 (m, 2H), 5.10 (d, J = 3.2 Hz, 1H), 5.82 (br, 1H) 6.21 (d, J = 3.2 Hz, 1H). ^{13}C NMR (100 MHz, $CDCl_3$) δ 7.9, 14.1, 25.8, 28.3 (3C), 57.7, 62.1 (2C), 79.4, 80.4, 108.6, 142.1, 153.6, 160.8, 170.9. IR (neat) 3429, 3134, 2978, 1724, 1615, 1578, 1487, 1367, 1307, 1261, 1168, 1135, 1068, 1022 cm^{-1}. $[\alpha]_D^{22}$ = +42.0 (*c* 1.00, $CHCl_3$, 91% ee). HRMS (FAB+) calcd for $C_{16}H_{25}NNaO_6$ $[M+Na]^+$ 350.1580, found 350.1581.

6-Ethyl 1-methyl (*S*,*Z*)-5-((*tert*-butoxycarbonyl)amino)-5-ethyl-4-oxohex-2-enedioate (15): 15 was synthesized based on the literature procedure.[21] Diethyl ether (3 mL) and saturated aqueous $NaHCO_3$ (3 mL) were added to a round bottom flask with **S33** (106.3 mg, 0.325 mmol, 91% ee), and the solution was stirred at 0 °C. *N*-bromosuccinimide (69.0 mg, 0.387 mmol) was added to the solution, and the mixture was stirred at 0 °C for 20 min. The resulting mixture was extracted with diethyl ether (10 mL × 3), and washed with brine (10 mL). The combined extracts were dried over Na_2SO_4. The organic phase was concentrated under reduced pressure, and the resultant residue was purified by silica gel column chromatography (eluent: *n*-hexane:EtOAc = 4:1 to 3:2) to give the desired product **15** as colorless oil (75.6 mg, 68% yield). ^{1}H NMR (400 MHz, $CDCl_3$) δ 0.79 (t, J = 7.3 Hz, 3H), 1.26 (t, J = 7.3 Hz, 3H), 1.44 (s, 9H), 2.33 (m, 1H), 2.49 (m, 1H), 3.77 (s, 3H), 4.16-4.30 (m, 2H), 6.06 (s, 1H), 6.23 (d, J = 12.4 Hz, 1H) 6.63 (d, J = 11.9 Hz, 1H). ^{13}C NMR (100 MHz, $CDCl_3$) δ 7.5, 14.0, 25.5, 28.3 (3C), 52.3, 62.7, 72.3, 80.3, 131.3, 131.8, 154.1, 166.1, 168.3, 193.0. IR (neat) 3423, 2979, 1717, 1486, 1368, 1247, 1168, 1054 cm^{-1}. $[\alpha]_D^{23}$ = –20.8 (*c* 1.00, $CHCl_3$, 91% ee). HRMS (FAB+) calcd for $C_{16}H_{25}NNaO_7$ $[M+Na]^+$

366.1529, found 366.1526.

Ethyl (2*S*)-2-((*tert*-butoxycarbonyl)amino)-2-(5-oxo-2,5-dihydrofuran-2-yl)butanoate (16): 16 was synthesized based on the literature procedure.[22] Methanol (4 mL) was added to a round bottom flask with **15** (75.6 mg, 0.220 mmol, 91% ee), and the solution was stirred at room temperature. Cerium(III) chloride heptahydrate (82.0 mg, 0.220 mmol) was added to the solution, and the mixture was stirred at room temperature for 10 min. The mixture was cooled to –78 °C, and sodium borohydride (8.3 mg, 0.219 mmol) was added. Then the mixture was warmed to room temperature and stirred at that temperature for 30 min. The resulting mixture was quenched with saturated aqueous NH_4Cl (5 mL), extracted with diethyl ether (10 mL × 3), and washed with brine (10 mL). The combined extracts were dried over Na_2SO_4. The organic phase was concentrated under reduced pressure, and the resultant residue was purified by silica gel column chromatography (eluent: *n*-hexane:EtOAc = 4:1 to 3:2) to give the desired product **16** as colorless oil (57.9 mg, 84% yield, dr = 80:20). The absolute configuration has not been determined. Two diastereomers could not be separated from each other. IR (neat, mixture of **isomer I** and **isomer II**) 3422, 2979, 1762, 1497, 1369, 1315, 1252, 1164, 1092 cm^{-1}. HRMS (FAB+, mixture of **I** and **II**) calcd for $C_{15}H_{23}NNaO_6$ $[M+Na]^+$ 336.1423, found 336.1425. **Major isomer I:** ^1H NMR (400 MHz, $CDCl_3$) δ 0.82 (t, *J* = 7.3 Hz, 3H), 1.31 (t, *J* = 7.3 Hz, 3H), 1.40 (s, 9H), 1.95 (m, 1H), 2.64 (m, 1H), 4.28 (q, *J* = 7.3 Hz, 2H), 5.53 (m, 2H), 6.09 (dd, *J* = 5.5, 1.8 Hz, 1H), 7.71 (d, *J* = 5.0 Hz, 1H). ^{13}C NMR (100 MHz, $CDCl_3$) δ 7.9, 14.1, 23.8, 28.2 (3C), 62.8, 65.6, 80.0, 85.3, 121.6, 153.7, 156.0, 170.4, 172.6. **Minor isomer II:** ^1H NMR (400 MHz, $CDCl_3$) δ 0.98 (t, *J* = 7.3 Hz, 3H), 1.28 (t, *J* = 7.3 Hz, 3H), 1.36 (s, 9H), 1.89-2.10 (m, 2H), 4.23 (q, *J* = 7.3 Hz, 2H), 4.84 (br, 1H), 5.57 (br, 1H), 5.99 (dd, *J* = 5.5, 1.8 Hz, 1H), 7.77 (d, *J* = 5.0 Hz, 1H). ^{13}C NMR (100 MHz, $CDCl_3$) δ 8.2, 14.2, 28.1 (3C), 29.0, 61.7, 63.9, 80.4, 82.9, 120.1, 154.1, 154.3, 169.8, 173.3.

23. Transformation of 11 to 17 (Scheme 3b).

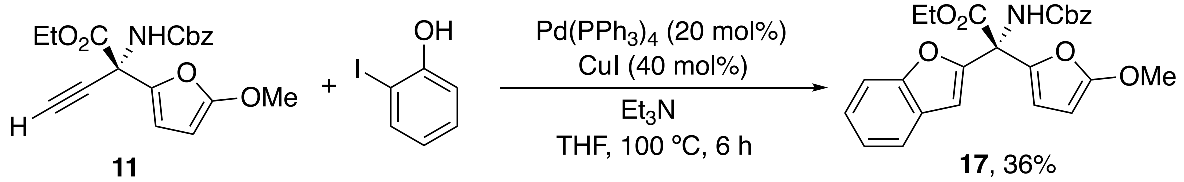

Ethyl (*S*)-2-(benzofuran-2-yl)-2-(((benzyloxy)carbonyl)amino)-2-(5-methoxyfuran-2-yl) acetate (17): 17 was synthesized based on the literature procedure.[22] To a two-necked round bottom flask with **11** (233 mg, 0.651 mmol, 91% ee), 2-iodophenol (404 mg, 1.83 mmol), tetrakis(triphenylphosphine) palladium(0) (80 mg, 0.069 mmol), and copper(I) iodide (27 mg, 0.14 mmol) were added THF (3 mL) under a nitrogen atmosphere. Triethylamine (7 mL) was added to the solution at 0 °C, and the mixture was stirred at 70 °C for 6 h. The resulting mixture was then

cooled in ice bath, and diluted with diethyl ether (20 mL) and 1 *M* HCl aqueous solution (10 mL). The mixture was extracted with diethyl ether (20 mL × 2) and washed with brine (10 mL). The combined extracts were dried over Na_2SO_4. The organic phase was concentrated under reduced pressure, and the resultant residue was purified by silica gel column chromatography (eluent: *n*-hexane:EtOAc = 5:1 to 3:2) to give the product **17** as orange oil (104 mg, 36% yield). ^{1}H NMR (400 MHz, $CDCl_3$) δ 1.19 (t, *J* = 6.9 Hz, 3H), 3.83 (s, 3H), 4.26 (q, *J* = 6.9 Hz, 2H), 5.05 (s, 2H), 5.17 (d, *J* = 3.2 Hz, 1H), 6.44 (br, 1H), 6.49 (br, 1H), 6.94 (br, 1H), 7.20-7.40 (m, 7H), 7.44 (d, *J* = 8.2 Hz, 1H), 7.55 (d, *J* = 7.8 Hz, 1H) ^{13}C NMR (100 MHz, $CDCl_3$) δ 13.9, 57.8, 61.5, 63.4, 66.9, 80.8, 107.0, 111.4, 111.9, 121.5, 123.0, 124.6, 128.0 (4C), 128.4 (2C), 136.2, 138.3, 152.0, 154.0, 154.7, 161.4, 167.5. IR (neat) 3412, 2975, 2938, 1734, 1615, 1574, 1496, 1454, 1369, 1256, 1121, 1024 cm^{-1}. $[\alpha]_D^{29}$ = +4.4 (*c* 1.00, $CHCl_3$, 91% ee). HRMS (FAB+) calcd for $C_{25}H_{23}NNaO_7$ $[M+Na]^+$ 472.1372, found 472.1382.

24. Transformation of 11 to 18 (Scheme 3c).

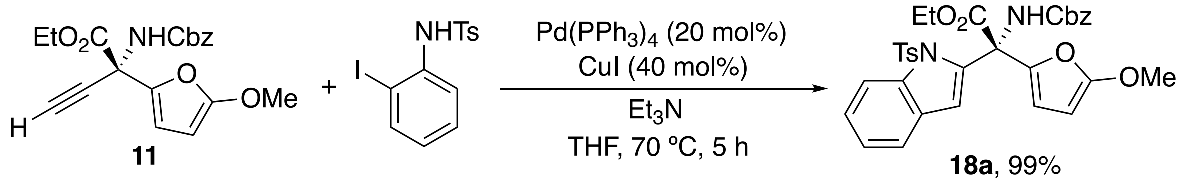

Ethyl (*S*)-2-(((benzyloxy)carbonyl)amino)-2-(5-methoxyfuran-2-yl)-2-(1-tosyl-1*H*-indol-2-yl) acetate (18a): 18a was synthesized based on the literature procedure.[22] To a two-necked round bottom flask with **11** (154 mg, 0.432 mmol, 91% ee), *N*-(2-iodophenyl)-4-toluenesulfonamide (323 mg, 0.865 mmol), tetrakis(triphenylphosphine) palladium(0) (104 mg, 0.090 mmol), and copper(I) iodide (34 mg, 0.18 mmol) was added THF (1 mL) under a nitrogen atmosphere. Triethylamine (1 mL, 7.2 mmol) was added to the solution at 0 °C, and the mixture was stirred at 70 °C for 5 h. The resulting mixture was then cooled in ice bath, and diluted with diethyl ether (5 mL) and 1 *M* HCl aqueous solution (10 mL). The mixture was extracted with diethyl ether (20 mL × 2) and washed with brine (10 mL). The combined extracts were dried over Na_2SO_4. The organic phase was concentrated under reduced pressure, and the resultant residue was purified by silica gel column chromatography (eluent: *n*-hexane:EtOAc = 5:1 to 3:1) to give the product **18a** as orange oil (257 mg, 99% yield). A trace amount of Et_2O and EtOAc remained. ^{1}H NMR (400 MHz, $CDCl_3$) δ 1.28 (t, *J* = 7.3 Hz, 3H), 2.25 (s, 3H), 3.82 (s, 3H), 4.32 (q, *J* = 7.3 Hz, 2H), 4.70 (d, *J* = 12.4 Hz, 1H), 4.99 (d, *J* = 12.4 Hz, 1H), 5.19 (d, *J* = 3.2 Hz, 1H), 6.32 (br, 1H), 6.62 (br, 1H), 6.82 (br, 1H), 7.02 (d, *J* = 7.8 Hz, 2H), 7.17-7.37 (m, 7H), 7.48 (d, *J* = 6.4 Hz, 1H), 7.57 (d, *J* = 7.3 Hz, 2H), 7.88 (d, *J* = 7.3 Hz, 1H). ^{13}C NMR (100 MHz, $CDCl_3$) δ 13.7, 22.2, 57.5, 62.2, 63.1, 66.1, 80.9, 111.7, 114.3, 116.7, 121.5, 123.2, 124.9, 126.3 (2C), 127.5 (2C), 127.8, 127.9, 128.3 (2C), 129.4 (2C), 136.1, 136.2, 136.8, 137.2, 138.8, 144.3, 153.5, 161.1, 167.6. IR (neat) 3415, 2978,

1736, 1613, 1572, 1495, 1452, 1366, 1250, 1175, 1036 cm^{-1}. $[\alpha]_D^{26}$ = –79.2 (*c* 1.00, $CHCl_3$, 91% ee). HRMS (FAB+) calcd for $C_{32}H_{30}N_2NaO_8S$ $[M+Na]^+$ 625.1621, found 625.1635.

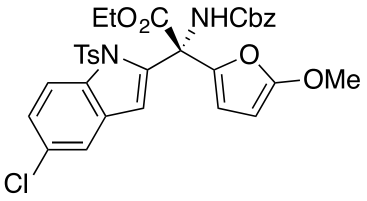

Ethyl (*S*)-2-(((benzyloxy)carbonyl)amino)-2-(5-chloro-1-tosyl-1*H*-indol-2-yl)-2-(5-methoxy furan-2-yl)acetate (18b): A trace amount of Et_2O remained. Orange oil. ^{1}H NMR (400 MHz, $CDCl_3$) δ 1.28 (t, *J* = 7.3 Hz, 3H) 2.27 (s, 3H), 3.82 (s, 3H), 4.32 (q, *J* = 7.3 Hz, 2H), 4.69 (d, *J* = 12.4 Hz, 1H), 4.99 (d, *J* = 12.4 Hz, 1H), 5.19 (d, *J* = 3.7 Hz, 1H), 6.30 (d, *J* = 3.2 Hz, 1H), 6.60 (br, 1H), 6.74 (br, 1H), 7.04 (d, *J* = 7.3 Hz, 2H), 7.20 (d, *J* = 9.2 Hz, 1H), 7.26-7.37 (m, 5H), 7.45 (s, 1H), 7.56 (d, *J* = 7.3 Hz, 2H), 7.81 (d, *J* = 8.2 Hz, 1H). ^{13}C NMR (100 MHz, $CDCl_3$) δ 13.9, 21.5, 57.8, 62.3, 63.5, 66.4, 81.3, 112.0, 115.7, 116.1, 121.2, 125.3, 126.6 (2C), 127.9 (2C), 128.1, 128.6 (2C), 129.1, 129.4, 129.7 (2C), 135.8, 136.3 (2C), 138.6, 138.8, 144.7, 153.7, 161.3, 167.6. IR (neat) 3411, 2926, 1738, 1613, 1496, 1448, 1368, 1248, 1172, 1036 cm^{-1}. $[\alpha]_D^{25}$ = –111.6 (*c* 1.00, $CHCl_3$, 91% ee). HRMS (FAB+) calcd for $C_{32}H_{29}ClN_2NaO_8S$ $[M+Na]^+$ 659.1231, found 659.1231.

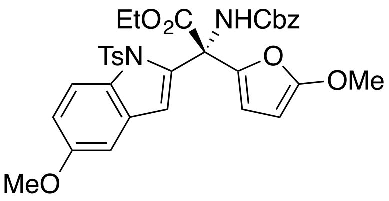

Ethyl (*S*)-2-(((benzyloxy)carbonyl)amino)-2-(5-methoxy-1-tosyl-1*H*-indol-2-yl)-2-(5-methoxy furan-2-yl)acetate (18c): Orange oil. ^{1}H NMR (400 MHz, $CDCl_3$) δ 1.27 (t, *J* = 7.3 Hz, 3H), 2.24 (s, 3H), 3.78 (s, 3H), 3.81 (s, 3H), 4.32 (q, *J* = 7.3 Hz, 2H), 4.69 (d, *J* = 12.4 Hz, 1H), 4.99 (d, *J* = 12.8 Hz, 1H), 5.18 (d, *J* = 3.2 Hz, 1H), 6.30 (d, *J* = 2.8 Hz, 1H), 6.62 (s, 1H), 6.76 (s, 1H), 6.86 (d, *J* = 8.7 Hz, 1H), 6.92 (s, 1H), 7.01 (d, *J* = 7.8 Hz, 2H), 7.25-7.39 (m, 5H), 7.54 (d, *J* = 7.3 Hz, 2H), 7.78 (d, *J* = 8.7 Hz, 1H). ^{13}C NMR (100 MHz, $CDCl_3$) δ 13.8, 21.3, 55.4, 57.6, 62.2, 63.1, 66.1, 81.0, 103.5, 111.7, 114.2, 115.3, 117.0, 126.3 (2C), 127.6 (2C), 127.9, 128.3 (2C), 128.9, 129.4 (2C), 131.9, 136.2, 136.3, 137.4, 138.9, 144.2, 153.5, 156.2, 161.1, 167.7. IR (neat) 3414, 2983, 1737, 1613, 1495, 1472, 1365, 1258, 1213, 1174, 1035 cm^{-1}. $[\alpha]_D^{26}$ = –124.8 (*c* 1.00, $CHCl_3$, 91% ee). HRMS (FAB+) calcd for $C_{33}H_{32}N_2NaO_9S$ $[M+Na]^+$ 655.1726, found 655.1730.

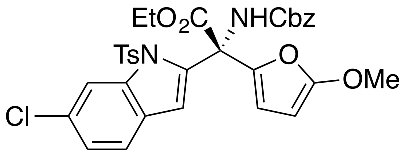

Ethyl (*S*)-2-(((benzyloxy)carbonyl)amino)-2-(6-chloro-1-tosyl-1*H*-indol-2-yl)-2-(5-methoxy furan-2-yl)acetate (18d): A trace amount of Et_2O remained. Orange oil. ^{1}H NMR (400 MHz, $CDCl_3$) δ 1.28 (t, *J* = 7.3 Hz, 3H), 2.27 (s, 3H), 3.82 (s, 3H), 4.32 (q, *J* = 7.3 Hz, 2H), 4.67 (d, *J* = 12.4 Hz, 1H), 4.98 (d, *J* = 11.9 Hz, 1H), 5.18 (d, *J* = 3.2 Hz, 1H), 6.29 (d, *J* = 3.2 Hz, 1H), 6.58 (br, 1H), 6.76 (br, 1H), 7.06 (d, *J* = 7.8 Hz, 2H), 7.18 (dd, *J* = 8.2, 1.8 Hz, 1H), 7.25-7.42 (m, 6H), 7.58 (d, *J* = 7.8 Hz, 2H), 7.93 (s, 1H). ^{13}C NMR (100 MHz, $CDCl_3$) δ 13.9, 21.5, 57.7, 62.3, 63.4, 66.3, 81.2, 111.9, 114.7, 116.3, 122.4, 124.1, 126.5 (2C), 126.6, 127.7 (2C), 128.1, 128.5 (2C), 129.7 (2C), 131.0, 136.2 (2C), 137.8 (2C), 138.9, 144.7, 153.7, 161.3, 167.6. IR (neat) 3411, 2936, 1737, 1613, 1495, 1448, 1368, 1250, 1174, 1035 cm^{-1}. $[\alpha]_D^{26}$ = –173.2 (*c* 1.00, $CHCl_3$, 91% ee). HRMS (FAB+) calcd for $C_{32}H_{29}ClMN_2NaO_8S$ $[M+Na]^+$ 659.1231, found 659.1209.

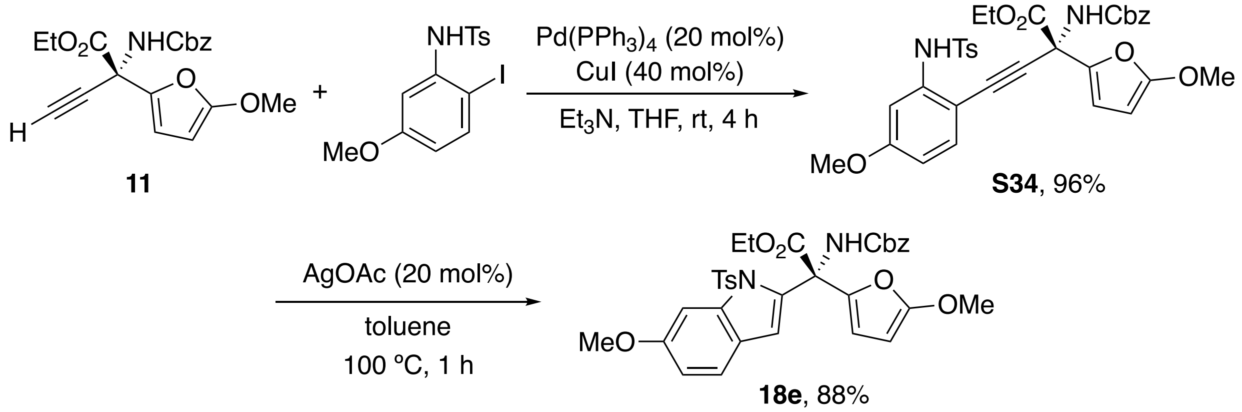

Ethyl (*S*)-2-(((benzyloxy)carbonyl)amino)-4-(4-methoxy-2-((4-methylphenyl)sulfonamido) phenyl)-2-(5-methoxyfuran-2-yl)but-3-ynoate (S34): S34 was synthesized based on the literature procedure.[23] To a two-necked roundbottom flask with **11** (261 mg, 0.730 mmol, 91% ee), *N*-(2-iodophenyl)-4-toluenesulfonamide (590 mg, 1.46 mmol), tetrakis(triphenylphosphine) palladium(0) (42 mg, 0.0363 mmol), and copper(I) iodide (14 mg, 0.0735 mmol) was added THF (6 mL) under a nitrogen atmosphere. Triethylamine (2 mL, 14.3 mmol) was added to the solution at 0 °C, and the mixture was stirred at room temperature for 4 h. The resulting mixture was then cooled in ice bath, and diluted with diethyl ether (5 mL) and 1 *M* HCl aqueous solution (10 mL). The mixture was extracted with diethyl ether (20 mL × 2) and washed with brine (10 mL). The combined extracts were dried over Na_2SO_4. The organic phase was concentrated under reduced pressure, and the resultant residue was purified by silica gel column chromatography (eluent: *n*-hexane:EtOAc = 4:1 to 3:2) to give the product **S34** as orange oil (442.9 mg, 96% yield). ^{1}H NMR (400 MHz, $CDCl_3$) δ 1.28 (t, *J* = 7.3 Hz, 3H), 2.35 (s, 3H), 3.80 (s, 3H), 3.84 (s, 3H), 4.35 (q, *J* = 7.3 Hz, 2H), 5.15 (d, *J* = 3.2 Hz, 1H), 5.20 (d, *J* = 11.9 Hz, 1H), 5.29 (d, *J* = 11.9 Hz, 1H), 6.25

(br, 1H), 6.45 (d, J = 3.2 Hz, 1H), 6.52 (dd, J = 8.7, 2.8 Hz, 1H), 7.13-7.43 (m, 9H), 7.77 (d, J = 8.2 Hz, 2H), 8.19 (br, 1H). ^{13}C NMR (100 MHz, $CDCl_3$) δ 14.0, 21.6, 55.5, 56.7, 57.9, 64.0, 67.5, 80.7, 80.8, 88.6, 104.3, 104.5, 110.1, 111.7, 127.4 (2C), 128.2, 128.4 (2C), 128.5 (2C), 129.6 (2C), 132.6, 136.0, 136.6, 138.0, 141.2, 143.6, 154.4, 161.0, 161.8, 166.7. IR (neat) 3405, 1717, 1613, 1574, 1507, 1407, 1340, 1261, 1159, 1091 cm^{-1}. $[\alpha]_D^{26}$ = –11.6 (c 1.00, $CHCl_3$, 91% ee). HRMS (FAB+) calcd for $C_{33}H_{32}N_2NaO_9S$ $[M+Na]^+$ 655.1726, found 655.1717.

Ethyl (*S*)-2-(((benzyloxy)carbonyl)amino)-2-(6-methoxy-1-tosyl-1*H*-indol-2-yl)-2-(5-methoxy furan-2-yl)acetate (18e): 18e was synthesized based on the literature procedure.[24] Toluene (7 mL) was added to a round bottom flask with **S34** (443 mg, 0.70 mmol), and the solution was stirred at room tempurature. Silver(I) acetate (23.0 mg, 0.138 mmol) was added to the solution, and the mixture was stirred at 100 °C for 1 h. The reaction mixture was passed through short silica gel (eluent: n-hexane:EtOAc = 2:1). The organic phase was concentrated under reduced pressure, and the resultant residue was purified by silica gel column chromatography (eluent: n-hexane:EtOAc = 4:1 to 3:2) to give the desired product **18e** as orange oil (390 mg, 88% yield). ^{1}H NMR (400 MHz, $CDCl_3$) δ .28 (t, J = 7.3 Hz, 3H), 2.26 (s, 3H), 3.79 (br, 3H), 3.82 (s, 3H), 4.31 (q, J = 7.3 Hz, 2H), 4.70 (d, J = 12.4 Hz, 1H), 5.00 (d, J = 12.4 Hz, 1H), 5.18 (d, J = 3.7 Hz, 1H), 6.30 (br, 1H), 6.60 (br, 1H), 6.75 (br, 1H), 6.83 (dd, J = 8.7, 2.3 Hz, 1H), 7.03 (d, J = 8.2 Hz, 2H), 7.26-7.42 (m, 7H), 7.56 (d, J = 7.8 Hz, 2H). ^{13}C NMR (100 MHz, $CDCl_3$) δ 14.0, 21.5, 55.7, 57.8, 62.5, 63.3, 66.3, 81.2, 99.0, 111.8, 112.7, 117.0, 122.0, 122.3, 126.5 (2C), 127.8 (2C), 128.0, 128.5 (2C), 129.6 (2C), 135.7, 136.5 (2C), 138.6, 139.2, 144.4, 153.7, 158.2, 161.2, 168.0. IR (neat) 3416, 2975, 1737, 1613, 1492, 1364, 1260, 1172, 1028 cm^{-1}. $[\alpha]_D^{25}$ = –204.0 (c 1.00, $CHCl_3$, 91% ee). HRMS (FAB+) calcd for $C_{33}H_{32}N_2NaO_9S$ $[M+Na]^+$ 655.1726, found 655.1701.

25. Transformation of 18a to 19 (Scheme 3d).

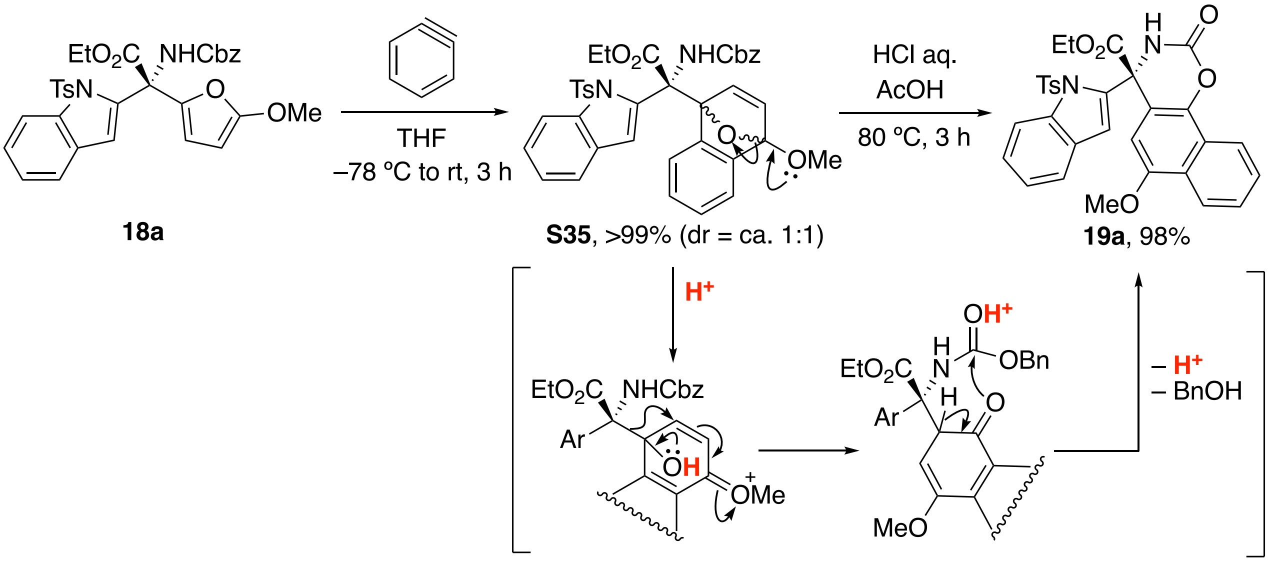

Ethyl (2*S*)-2-(((Benzyloxy)carbonyl)amino)-2-(4-methoxy-1,4-epoxynaphthalen-1(4*H*)-yl)-2-(1-tosyl-1*H*-indol-2-yl)acetate (S35): **S35** was synthesized based on the literature procedure.[25] To a well-dried pyrex Schlenk tube with cesium fluoride (261 mg, 1.72 mmol) in THF (4 mL) was added 18-crown-6 (680 mg, 2.58 mmol) in THF (4 mL). 2-(Trimethylsilyl)phenyl trifluoromethanesulfonate (210 μL, 0.86 mmol) was added, and the mixture was stirred at room temperature for 30 min. The mixture was cooled to –78 °C, and **18a** (257 mg, 0.426 mmol) in THF (2 mL) was added. Then the mixture was warmed to room temperature and stirred at that temperature for 2 h. The resulting mixture was diluted with water (5 mL), extracted with diethyl ether (20 mL × 2), and washed with brine (10 mL). The combined extracts were dried over Na_2SO_4. The organic phase was concentrated under reduced pressure, and the resultant residue was roughly purified by short silica gel column chromatography (eluent: *n*-hexane:EtOAc = 5:1 to 3:1) to give the product **S35** as light blue oil (294 mg, >99% yield, dr = ca. 1:1), which was used in the next step as soon as possible. ^{1}H NMR (400 MHz, $CDCl_3$) Many peaks of the diastereomers (dr = ca. 1:1) overlapped. δ 1.27 (t, *J* = 7.3 Hz, 6H), 2.29 (s, 6H), 3.65 (s, 6H), 4.19-4.43 (m, 4H), 4.79 (d, *J* = 11.9 Hz, 2H), 5.12 (d, *J* = 10.5 Hz, 2H), 6.66-8.87 (m, 38H), 8.15 (d, *J* = 7.4 Hz, 2H), 8.28 (br, 2H). ^{13}C NMR (100 MHz, $CDCl_3$) Many peaks of the diastereomers (dr = ca. 1:1) overlapped. δ 13.8, 14.0, 15.1, 21.6, 29.8, 55.4, 59.1, 63.1, 64.2, 65.6, 66.5, 66.8, 83.5, 102.5, 114.5, 114.7, 119.5, 121.4, 121.9, 122.2, 123.2, 123.5, 123.6, 125.0, 125.6, 126.1, 126.4, 126.7, 126.9, 127.4, 127.8, 127.9, 128.1, 128.3, 128.5, 129.7, 136.3, 137.7, 144.4, 144.7, 149.3, 153.8, 166.9, 169.7. HRMS (FAB+) calcd for $C_{38}H_{34}N_2NaO_8S$ $[M+Na]^+$ 701.1934, found 701.1909.

Ethyl (*R*)-6-methoxy-2-oxo-4-(1-tosyl-1*H*-indol-2-yl)-3,4-dihydro-2*H*-naphtho[2,1-e][1,3]oxazine-4-carboxylate (19a): To a two-necked round bottom flask with **S35** (294 mg, 0.43 mmol) were added acetic acid (9 mL) and 12 *M* HCl aqueous solution (1 mL). The solution was stirred at 80 °C for 3 h. Then the resulting mixture was diluted with diethyl ether (20 mL) and $NaHCO_3$ aqueous solution (200 mL). The mixture was extracted with diethyl ether (20 mL × 2) and washed with brine (10 mL). The combined extracts were dried over Na_2SO_4. The organic phase was concentrated under reduced pressure, and the resultant residue was purified by silica gel column chromatography (eluent: *n*-hexane:EtOAc = 5:1 to 3:1) to give the product **19a** as colorless solid (241 mg, 98% yield). The enantiomeric purity of **19a** was determined by chiral HPLC analysis (91% ee). ^{1}H NMR (400 MHz, $CDCl_3$) δ 1.33 (t, *J* = 6.9 Hz, 3H), 2.37 (s, 3H), 3.69 (s, 3H), 4.31 (m, 1H), 4.44 (m, 1H), 6.09 (s, 1H), 6.81 (s, 1H), 7.13 (t, *J* = 8.0 Hz, 1H), 7.20 (t, *J* = 8.2 Hz, 1H), 7.24-7.34 (m, 4H), 7.61-7.70 (m, 3H), 7.80 (d, *J* = 8.2 Hz, 2H), 8.31 (d, *J* = 9.1 Hz, 1H), 8.37 (d, *J* = 8.7 Hz, 1H). ^{13}C NMR (100 MHz, $CDCl_3$) δ 14.1, 21.7, 56.0, 63.5, 64.5, 99.4, 111.5, 114.2, 114.7, 121.7, 121.9, 122.1, 123.8, 124.3, 125.8, 126.4, 127.2 (2C), 127.6 (2C), 127.9, 130.2 (2C), 135.3, 136.8, 139.8, 140.9, 145.5, 149.0, 152.3, 168.9. IR (KBr) 3359, 2925, 1731, 1593, 1460, 1354, 1243, 1170, 1106, 1089, 1057 cm^{-1}. M.p. 142 °C (decomposition). $[\alpha]_D^{30}$ = –204.3 (*c* 1.00, $CHCl_3$, 91% ee). HRMS (FAB+) calcd for $C_{31}H_{26}N_2NaO_7S$ $[M+Na]^+$ 593.1358, found

593.1340. HPLC analysis; OD-3 × 2, *n*-hexane/*i*-PrOH = 4/1, 254 nm, 0.6 mL/min, t_R = 25.2 min (major, *R*), 29.9 min (minor, *S*).

Crystal data of 19a (Fig. S11): Compound **19a** was recrystallized in benzene for X-ray analysis. Formula $C_{32}H_{27}Cl_3N_2O_7S$, colorless, crystal dimensions 0.75 × 0.12 × 0.10 mm^3, monoclinic, space group $P2_1$ (#4), a = 10.664(3) Å, b = 10.372(2) Å, c = 14.789(4) Å, α = 90.00 °, β = 104.778(6) °, γ = 90.00 °, V = 1581.7(7) $Å^3$, Z = 2, ρ_{calc} = 1.449 g cm^{-3}, F(000) = 712, μ(MoKα) = 0.407 mm^{-1}, T = 123 K. 13598 reflections collected, 6894 independent reflections with $I > 2\sigma(I)$ ($2\theta_{max}$ = 27.48 °), and 469 parameters were used for the solution of the structure. The non-hydrogen atoms were refined anisotropically. Flack x = 0.009(16). R_1 = 0.0318 and wR_2 = 0.0794. GOF = 1.041. Crystallographic data for the structure reported in this paper have been deposited with the Cambridge Crystallographic Data Centre as supplementary publication no. CCDC-1520624. Copies of the data can be obtained free of charge on application to CCDC, 12 Union Road, Cambridge CB2 1EZ, UK [Web page: http://www.ccdc.cam.ac.uk/].

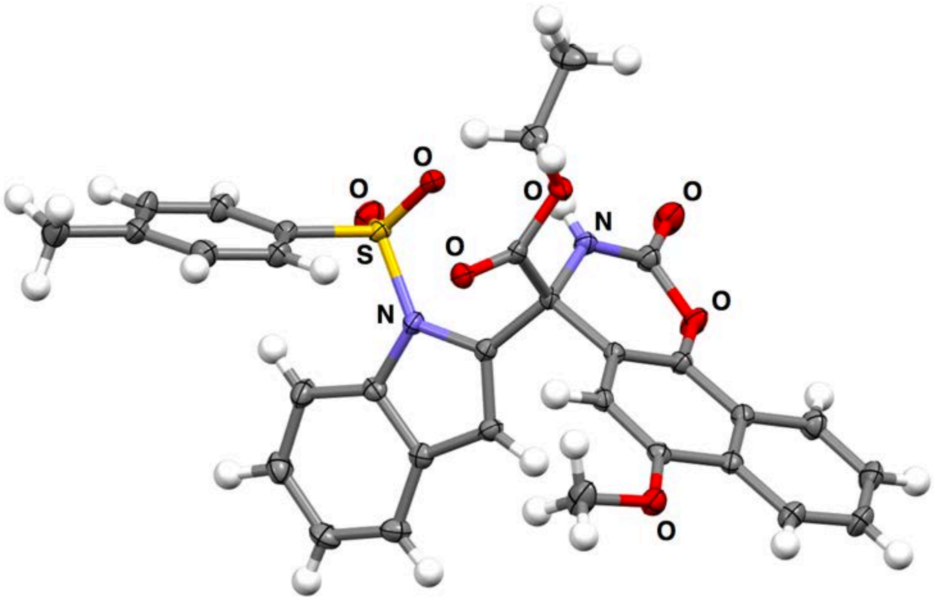

Fig. S11 ORTEP drawing of **19a**.

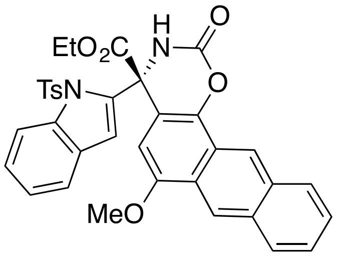

Ethyl (*R*)-6-methoxy-2-oxo-4-(1-tosyl-1*H*-indol-2-yl)-3,4-dihydro-2*H*-anthra[2,1-e][1,3] oxazine-4-carboxylate (19b): 3-(Trimethylsilyl)-2-naphthyl trifluoromethanesulfonate was used

in place of 2-(trimethylsilyl)phenyl trifluoromethanesulfonate in the procedure above. A trace amount of Et_2O remained. Yellow viscous oil. ^{1}H NMR (400 MHz, $CDCl_3$) δ 1.34 (t, J = 6.9 Hz, 3H), 2.38 (s, 3H), 4.03 (s, 3H), 4.33 (m, 1H), 4.45 (m, 1H), 6.19 (s, 1H), 6.69 (s, 1H), 7.13 (t, J = 7.3 Hz, 1H), 7.21 (t, J = 7.3 Hz, 1H), 7.29-7.31 (m, 3H), 7.36 (s, 1H), 7.56-7.59 (m, 2H), 7.64 (d, J = 8.2 Hz, 1H), 7.81 (d, J = 8.2 Hz, 2H), 8.06-8.13 (m, 2H), 8.87 (s, 1H), 8.96 (s, 1H). ^{13}C NMR (100 MHz, $CDCl_3$) δ 14.2, 21.7, 56.0, 63.5, 64.6, 97.1, 109.7, 114.3, 114.7, 121.5, 121.6, 121.7, 122.7, 123.8, 125.1, 125.8, 126.8 (2C), 127.3 (2C), 127.7, 128.7, 128.8, 130.2 (2C), 132.4, 132.5, 135.4, 136.9, 139.9, 140.8, 145.5, 149.0, 152.5, 169.0. IR (neat) 3416, 2975, 2927, 1741, 1459, 1358, 1308, 1239, 1173, 1089, 1041 cm^{-1}. $[\alpha]_D^{31}$ = –92.0 (c 1.00, $CHCl_3$, 91% ee). HRMS (FAB+) calcd for $C_{35}H_{28}N_2NaO_7S$ $[M+Na]^+$ 643.1515, found 643.1504.

26. Gram-scale reaction and transformation to amino acid 21 (Scheme 4).

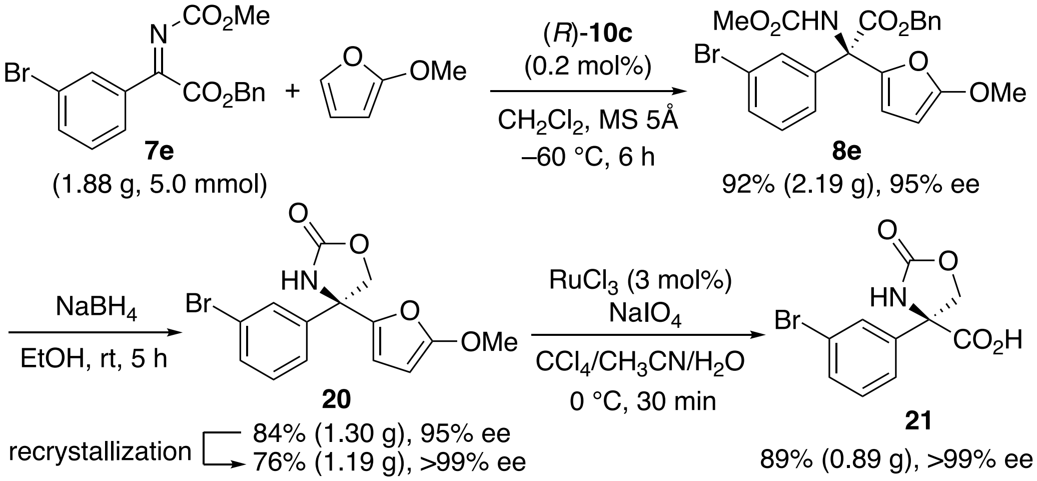

5 mmol scale reaction of 7e: To a well-dried two-necked flask charged with activated MS 5Å (1.25 g) under a nitrogen atmosphere were added (R)-**10c** (8.1 mg, 0.010 mmol) in dichloromethane (40 mL) at –78 °C. After 5 min, α-ketimino ester **7e** (1.881 g, 5.00 mmol) in dichloromethane (10 mL), and 2-methoxyfuran (930 μL, 10.0 mmol) were added at –78 °C. The reaction mixture was allowed to warm to –60 °C and was then stirred at that temperature for 6 h. The resulting mixture was quenched with triethylamine (0.1 mL) at –60 °C, and concentrated under reduced pressure at room temperature. The residue was purified by silica gel column chromatography (eluent: n-hexane:EtOAc = 3:1 to 1:1) to give the product **8e** (2.187 g, 92% yield). The enantiomeric purity was determined by chiral HPLC analysis (95% ee).

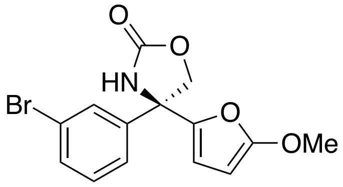

(*S*)-4-(3-Bromophenyl)-4-(5-methoxyfuran-2-yl)oxazolidin-2-one (20): To a solution of **8e** (2.187 g, 4.61 mmol, 95% ee) in ethanol (15 mL) was added sodium borohydride (0.698 g, 18.4 mmol). The mixture was stirred at room temperature for 5 h, and concentrated under reduced pressure. The resultant residue was dissolved in dichloromethane (30 mL). Saturated NH_4Cl aqueous solution (30 mL) was added, and the mixture was extracted with dichloromethane (10 mL × 3), and the combined organic layer was dried over Na_2SO_4. The organic phase was concentrated under reduced pressure, and purified by silica gel column chromatography (eluent: *n*-hexane:EtOAc = 2:1 to 1:1) to give the desired product **20** (1.302 g, 84% yield, 95% ee). Compound **20** was recrystallized from dichloromethane/diethyl ether/*n*-hexane (1:10:5) at room temperature (1.190 g, 76% yield, >99% ee). Coloreless oil. ^{1}H NMR (400 MHz, $CDCl_3$) δ 3.84 (s, 3H), 4.41 (d, *J* = 8.7 Hz, 1H), 4.91 (d, *J* = 8.7 Hz, 1H), 5.11 (d, *J* = 3.2 Hz, 1H), 6.09 (d, *J* = 3.7 Hz, 1H), 6.34 (s, 1H), 7.24-7.32 (m, 2H), 7.46-7.53 (m, 2H). ^{13}C NMR (100 MHz, $CDCl_3$) δ 57.9, 62.5, 75.1, 80.2, 110.4, 122.9, 124.4, 128.9, 130.4, 131.6, 142.6, 142.7, 158.8, 162.1. IR (KBr) 3317, 3071, 1761, 1736, 1615, 1575, 1372, 1262, 1038 cm^{-1}. M.p. 122 °C. $[\alpha]_D^{26}$ = +119.6 (*c* 1.00, $CHCl_3$, >99% ee). HPLC analysis; IA-3, CH_2Cl_2, 254 nm, 0.9 mL/min, t_R = 14.0 min (major, *S*), 33.7 min (minor, *R*). HRMS (FAB+) calcd for $C_{14}H_{12}BrNO_4$ $[M]^+$ 336.9950, found 336.9940.

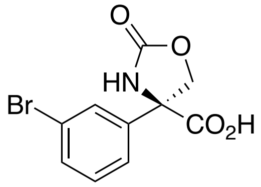

(*S*)-4-(3-Bromophenyl)-2-oxooxazolidine-4-carboxylic acid (21): **21** was synthesized based on the literature procedure.[26] To a solution of **20** (1.190 g, 3.52 mmol) in acetonitrile (18 mL) and carbon tetrachloride (18 mL), water (35 mL) and sodium periodate (11.29 g, 52.8 mmol) were added at 0 °C. After stirred for 5 min, ruthenium(III) chloride (21.9 mg, 0.11 mmol) was added, and the mixture was stirred at 0 °C for 30 min. The mixture was filtered and the filtrate was extracted with ethyl acetate (15 mL × 3). Diethyl ether (10 mL) was added to the combined organic layer, and the solution was stirred at room temperature for 2 h. Ruthenium(IV) oxide was gradually precipitated. The suspension was dried over Na_2SO_4, and filtered through a pad of Celite. The filtrate was concentrated under reduced pressure. The residue was dissolved in chloroform (20 mL), and extracted with $NaHCO_3$ aqueous solution (20 mL × 2). The combined aqueous layer was acidified with 3 *M* HCl aqueous solution (20 mL), and extracted with chloroform (15 mL × 3). The combined organic layer was dried over Na_2SO_4, and concentrated

under reduced pressure to give the desired product **21** (0.893 g, 89% yield). Light brown oil. ^{1}H NMR (400 MHz, $CDCl_3$) δ 4.43 (d, *J* = 9.2 Hz, 1H), 5.22 (d, *J* = 9.2 Hz, 1H), 7.28 (t, *J* = 7.8 Hz, 1H), 7.37 (d, *J* = 8.2 Hz, 1H), 7.52 (d, *J* = 7.8 Hz, 1H), 7.58 (t, *J* = 1.8 Hz, 1H), 7.78 (s, 1H), 8.87 (brs, 1H). ^{13}C NMR (100 MHz, $CDCl_3$) δ 66.7, 74.2, 123.4, 123.5, 128.0, 130.9, 132.4, 139.6, 160.2, 172.9. IR (neat) 3291, 1739, 1475, 1421, 1261, 1052 cm^{-1}. $[\alpha]_D^{27}$ = +106.0 (*c* 1.00, $CHCl_3$, >99% ee). HRMS (FAB+) calcd for $C_{10}H_9BrNO_4$ $[M+H]^+$ 285.9715, found 285.9709.

27. Theoretical study on the *E*/*Z*-geometry of substrates 1b and 7a.

With regard to Eqs. 2 and 3 in the main text, we considered the *E*/*Z*-geometry of the substrates. According to the literature, **1b** would have an *E*-geometry,[6] whereas **7a** would have a Z-geometry.[27] Indeed, a preliminary theoretical study was preliminary performed by a molecular mechanics method (MM2, Chem3D for Windows) (Fig. S12). As a result, (*E*)-**1b** was more stable than (*Z*)-**1b** by 8.12 kcal/mol (Fig. S12a). On the other hand, (*Z*)-**7a** was more stable than (*E*)-**7a** by 2.97 kcal/mol (Fig. S12b). Moreover, ^{1}H and ^{13}C NMR analyses of either **1b** or **7a** showed a single geometric isomer in $CDCl_3$ at room temperature, and thus the observed geometry (i.e., (*E*)-**1b** and (*Z*)-**7a**) should be quite stable.

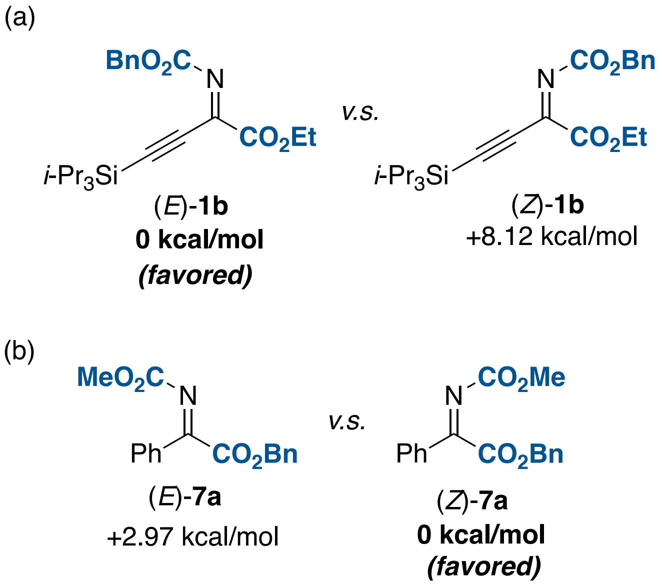

Fig. S12 Theoretical study for the *E*/*Z*-geometry of substrates **1b** and **7a**.

28. Possible transition states for the reactions.

Fig. S13a shows a possible transition state with the use of (*R*)-**5b**/**1b**/**2**. The imino nitrogen atom of **1b** might coordinate to the C_2-symmetric chiral Brønsted acid center of (*R*)-**5b**. Under these conditions, the sterically hindered *i*-Pr_3Si moiety of **1b** might be far from the 3,5-(*o*-Tol)C_6H_3 moiety and turned outward to avoid steric constraints. Nucleophile **2** would then selectively attack the activated **1b** from the *si*-face. As a result, enantioenriched (*S*)-**3b** might be provided (up to 91% ee). The steric effect of the silyl moiety might play an important role in the orientation of the substrates, and the sterically more hindered silyl moiety could induce high enantioselectivity: Ph (76% ee) < Ph_3Si (79% ee) < *t*-$BuMe_2Si$ (82% ee) < *t*-$BuPh_2Si$ (88% ee) < *i*-Pr_3Si (91% ee) (see Table S5).

Fig. S13b shows a possible transition state with the use of (*R*)-**10c**/**7a**/**2**. The imino nitrogen atom of **7a** might coordinate to the C_1-symmetric chiral Brønsted acid center of (*R*)-**10c**. Under these conditions, the sterically hindered phenyl moiety of **7a** might avoid steric constraints from the outstandingly bulky 2,4,6-$Cy_3C_6H_2$ moiety of catalyst (*R*)-**10c**. Nucleophile **2** would then selectively attack the activated **7a** from the *re*-face. As a result, enantioenriched (*R*)-**8a** might be provided (up to 95% ee).

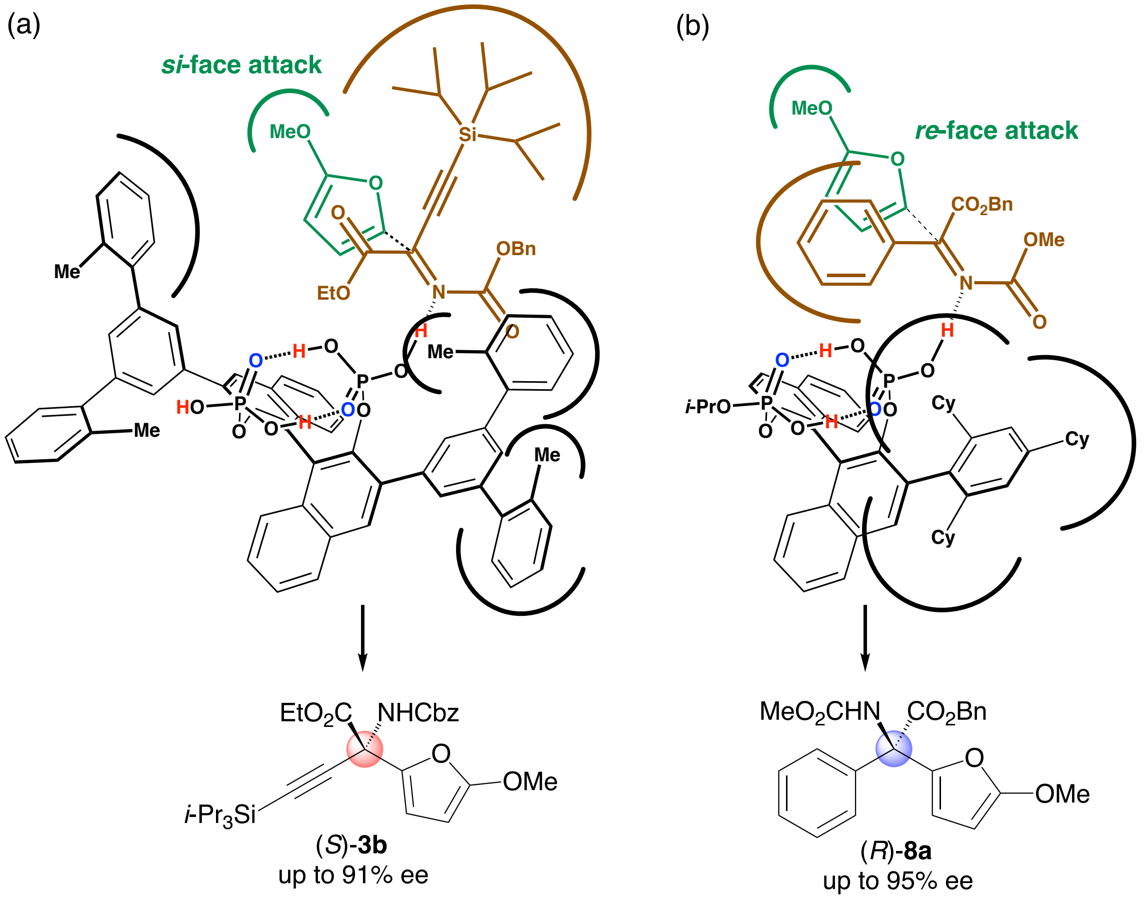

Fig. S13 Possible transition states for the reactions with the use of (*R*)-**5b** and (*R*)-**10c**.

29. References.

1 D. G. Manly and E. D. Amstutz, *J. Org. Chem.*, 1956, **21**, 516.

2 B. Wang, C. M. Yu, Z. W. Chen and W. K. Su, *Chinese Chem. Lett.*, 2008, **19**, 904.

3 T. Harada and K. Kanda, *Org. Lett.*, 2006, **8**, 3817.

4 I. Mizota, Y. Matsuda, S. Kamimura, H. Tanaka and M. Shimizu, *Org. Lett.*, 2013, **15**, 4206.

5 M. Guo, D. Li and Z. Zhang, *J. Org. Chem.*, 2003, **68**, 10172.

6 M. Hatano, K. Yamashita, M. Mizuno, O. Ito and K. Ishihara, *Angew. Chem., Int. Ed.*, 2015, **54**, 2707.

7 W. Yan, D. Wang, J. Feng, P. Li, D. Zhao and R. Wang, *Org. Lett.*, 2012, **14**, 2512.

8 P. Calí and M. Begtrup, *Synthesis*, 2002, **2002**, 2515.

9 D. Uraguchi and M. Terada, *J. Am. Chem. Soc.*, 2004, **126**, 5356.

10 M. Terada, K. Machioka and K. Sorimachi, *Angew. Chem., Int. Ed.*, 2009, **48**, 2553.

11 T. Akiyama, H. Morita, J. Itoh and K. Fuchibe, *Org. Lett.*, 2005, **7**, 2583.

12 (*a*) S. Hoffmann, A. M. Seayad, B. List, *Angew. Chem., Int. Ed.* 2005, **44**, 7424; (*b*) M. Klussmann, L. Ratjen, S. Hoffmann, V. Wakchaure, R. Goddard, B. List, *Synlett*, 2010, **2010**, 2189.

13 M. Hatano, T. Ozaki, K. Nishikawa and K. Ishihara, *J. Org. Chem.*, 2013, **78**, 10405.

14 S. Liu and L. G. Pedersen, *J. Phys. Chem. A*, 2009, **113**, 3648.

15 F. Krasovec and J. Jan, *Croat. Chem. Acta*, 1963, **35**, 183.

16 D. Shamir, I. Zilbermann, E. Maimon, A. I. Shames, H. Cohen and D. Meyerstein, *Inorg. Chim. Acta,* 2010, **363**, 2819.

17 P. Christ, A. G. Lindsay, S. S. Vormittag, J.-M. Neudröfl, A. Berkessel and A. M. C. O'Donoghue, *Chem. Eur. J.*, 2011, **17**, 8524.

18 Y. Qian, C. Jing, C. Zhai and W.-h. Hua, *Adv. Synth. Catal.*, 2012, **354**, 301.

19 H. M. L. Davies, T. Hansen and M. R. Churchill, *J. Am. Chem. Soc.*, 2000, **122**, 3063.

20 K. Dolbeare, G. F. Pontoriero, S. K. Gupta, R. K. Mishra and R. L. Johnson, *J. Med. Chem.*, 2003, **46**, 727.

21 D. Uraguchi, K. Sorimachi and M. Terada, *J. Am. Chem. Soc.*, 2004, **126**, 11804.

22 B. Prasad, R. Adepu, S. Sandra, D. Rambabu, G. R. Krishna, C. M. Reddy, G. S. Deora, P. Misra and M. Pal, *Chem. Commun.*, 2012, **48**, 10434.

23 E. Chong and S. A. Blum, *J. Am. Chem. Soc.*, 2015, **137**, 10144.

24 (*a*) T. Kurisaki, T. Naniwa, H. Yamamoto, H. Imagawa and M. Nishizawa, *Tetrahedron Lett.*, 2007, **48**, 1871; (*b*) B. C. J. van Esseveldt, F. L. van Delft, J. M. M. Smits, R. de Gelder, H. E. Schoemaker and F. P. J. T. Rutjes, *Adv. Synth. Catal.*, 2004, **346**, 823.

25 G. E. Collis and A. K. Burrell, *Tetrahedron Lett.*, 2005, **46**, 3653.

26 (*a*) F. Köhler, H.-J. Gais and G. Raabe, *Org. Lett.*, 2007, **9**, 1231; (*b*) H. Liu, J. Xu and D.-M. Du, *Org. Lett.*, 2007, **9**, 4725.

27 T. Hashimoto, K. Yamamoto and K. Maruoka, *Chem. Lett.*, 2011, **40**, 326.

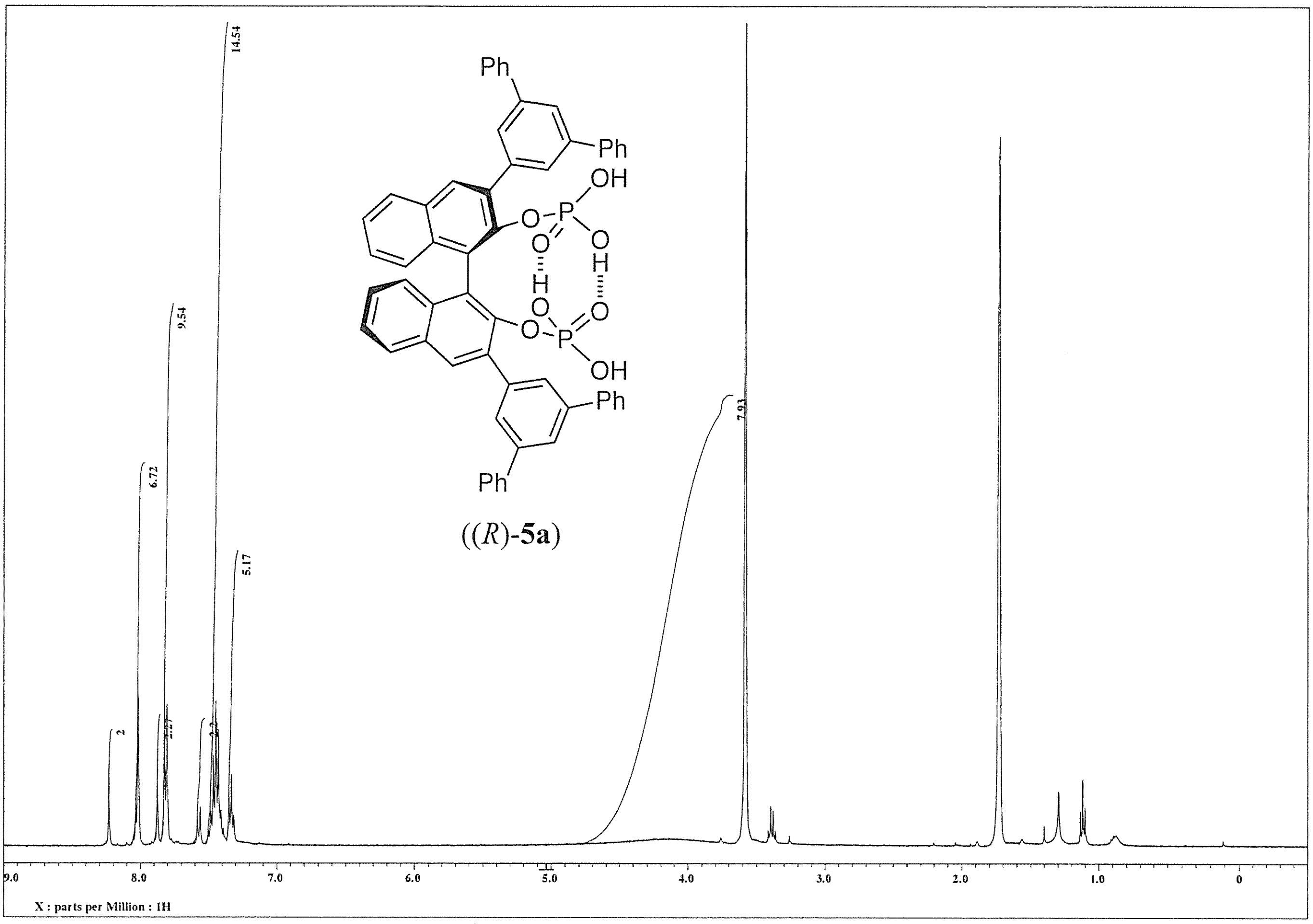

^{1}H NMR, 400 MHz, THF-d_8

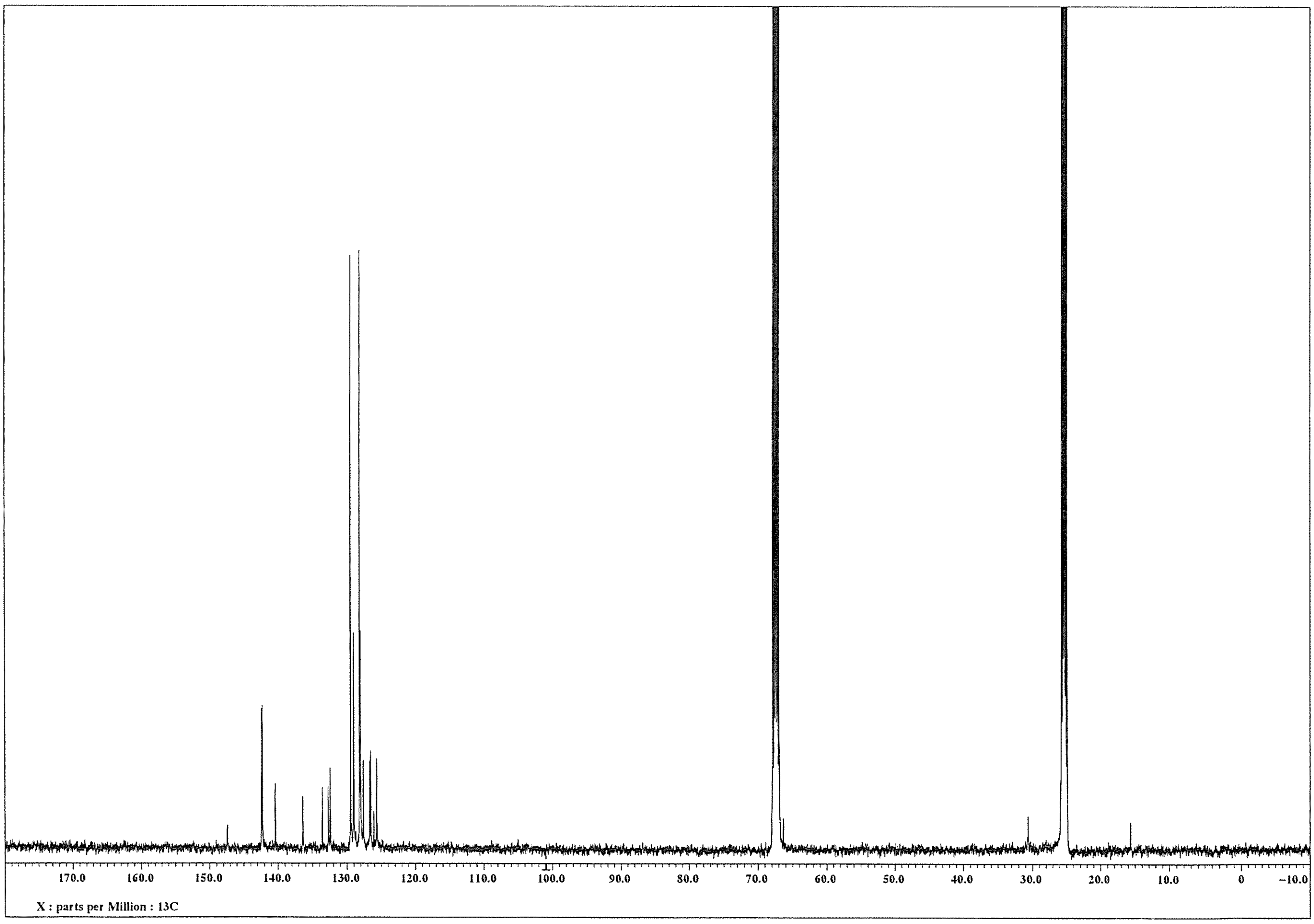

^{13}C NMR, 100 MHz, THF-d_8

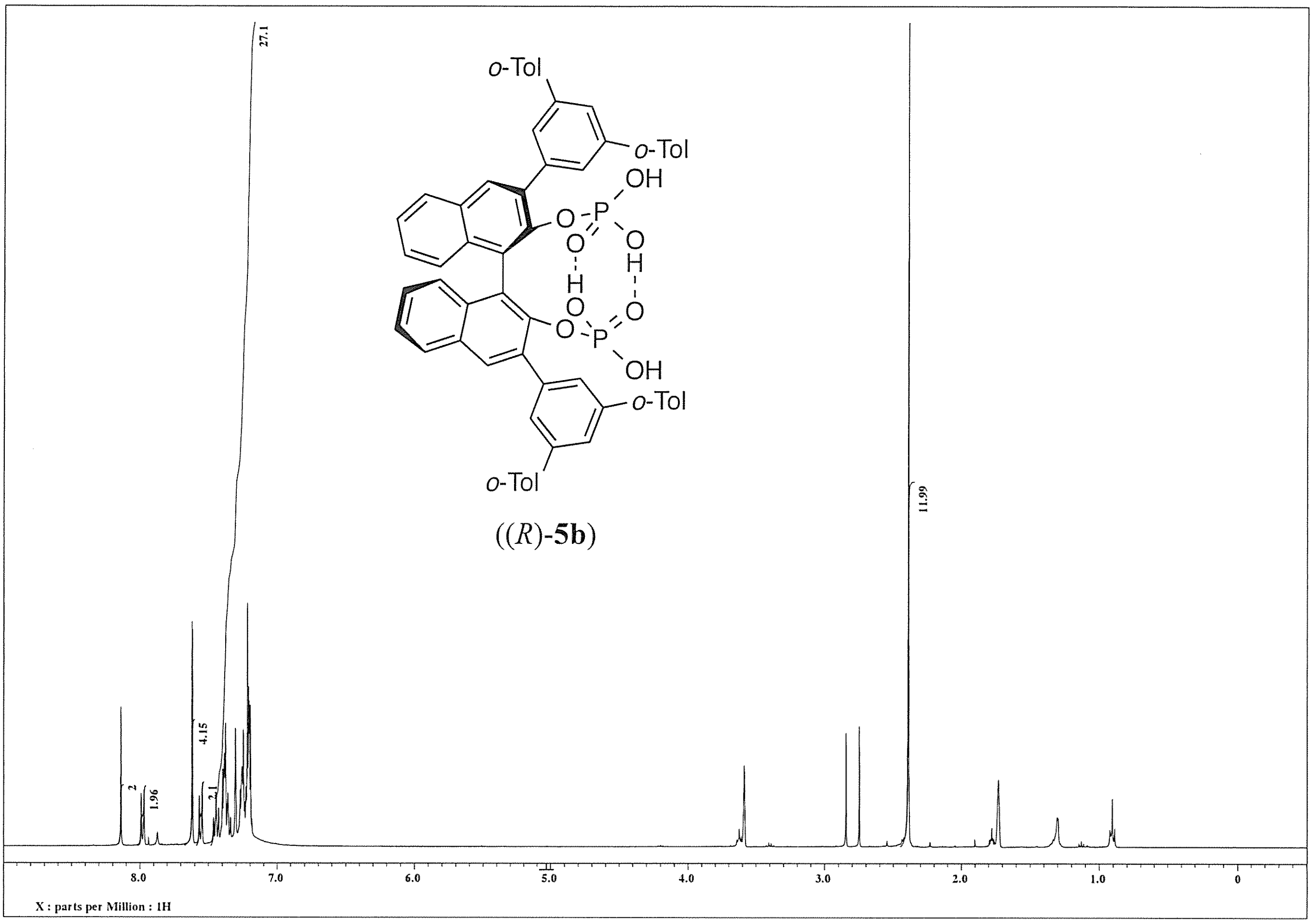

^{1}H NMR, 400 MHz, THF-d_8

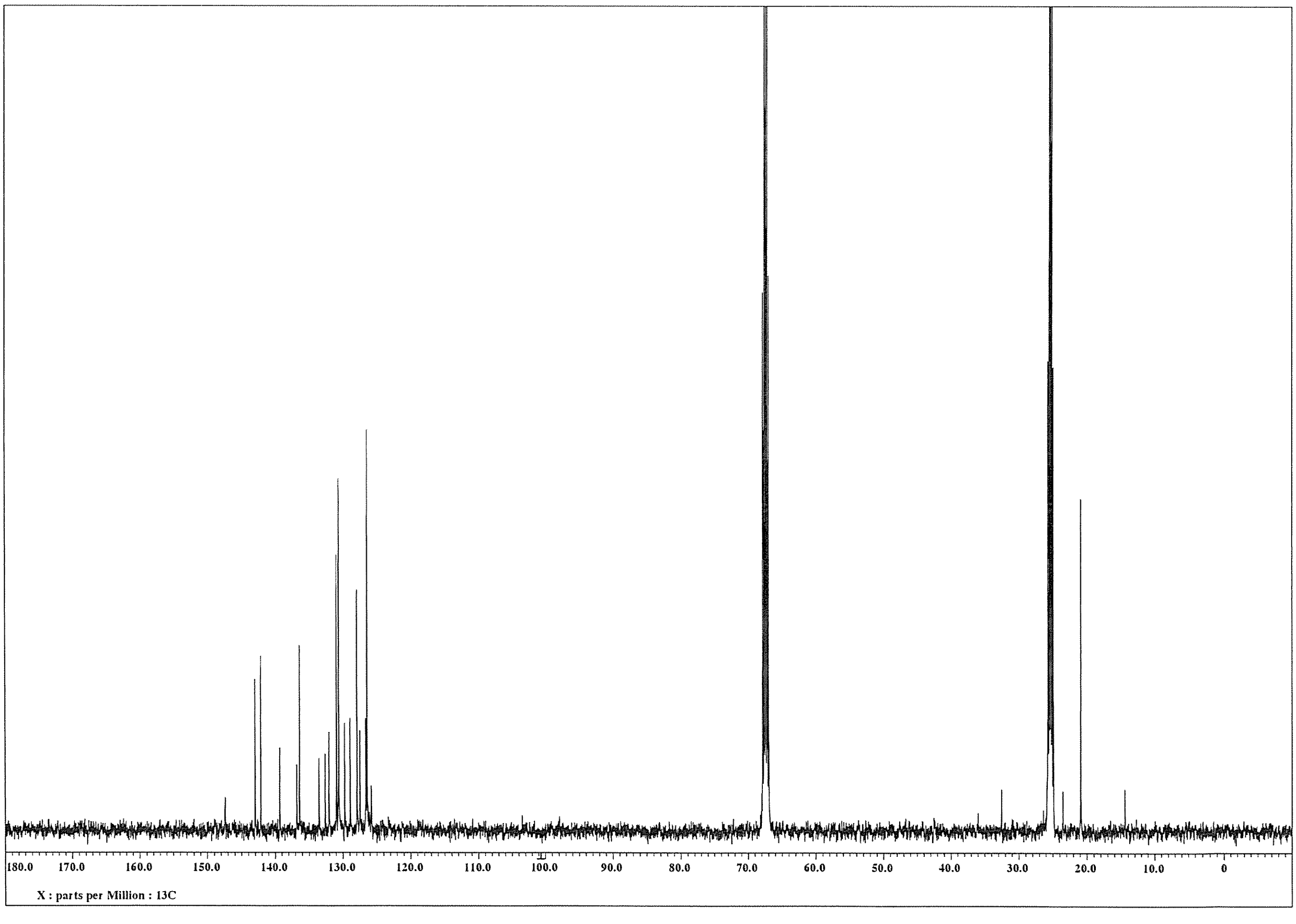

^{13}C NMR, 100 MHz, THF-d_8

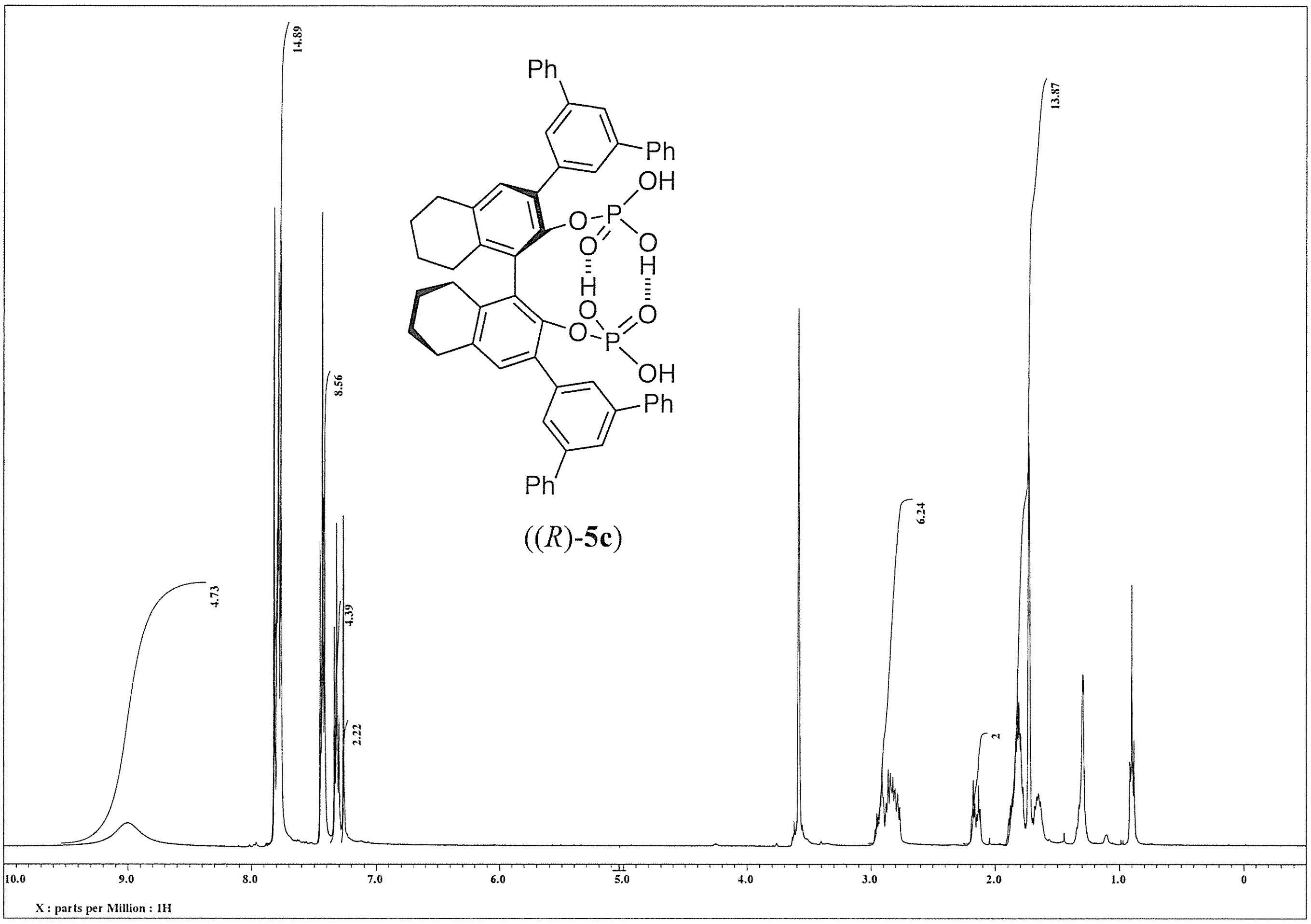

^{1}H NMR, 400 MHz, THF-d_8

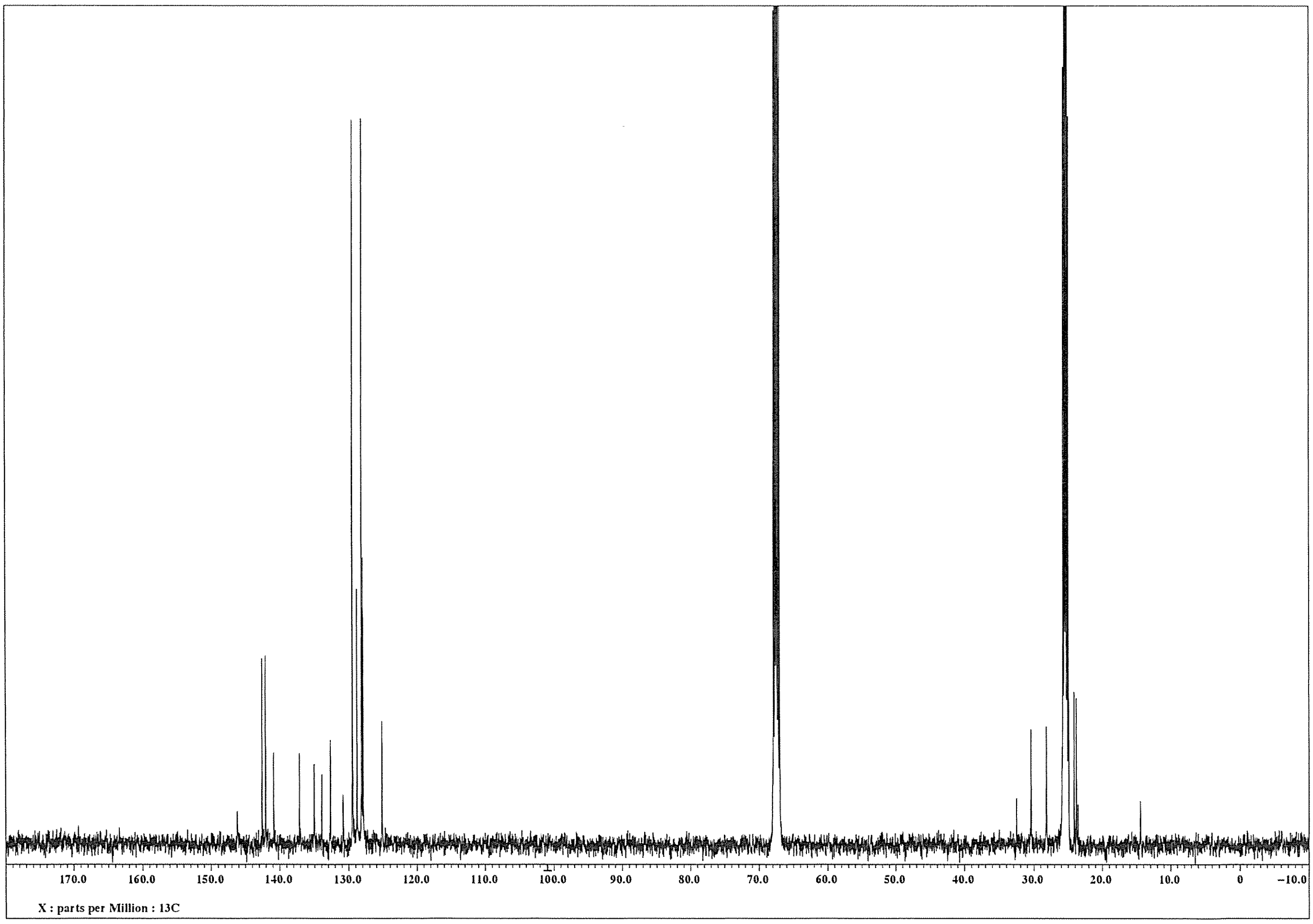

^{13}C NMR, 100 MHz, THF-d_8

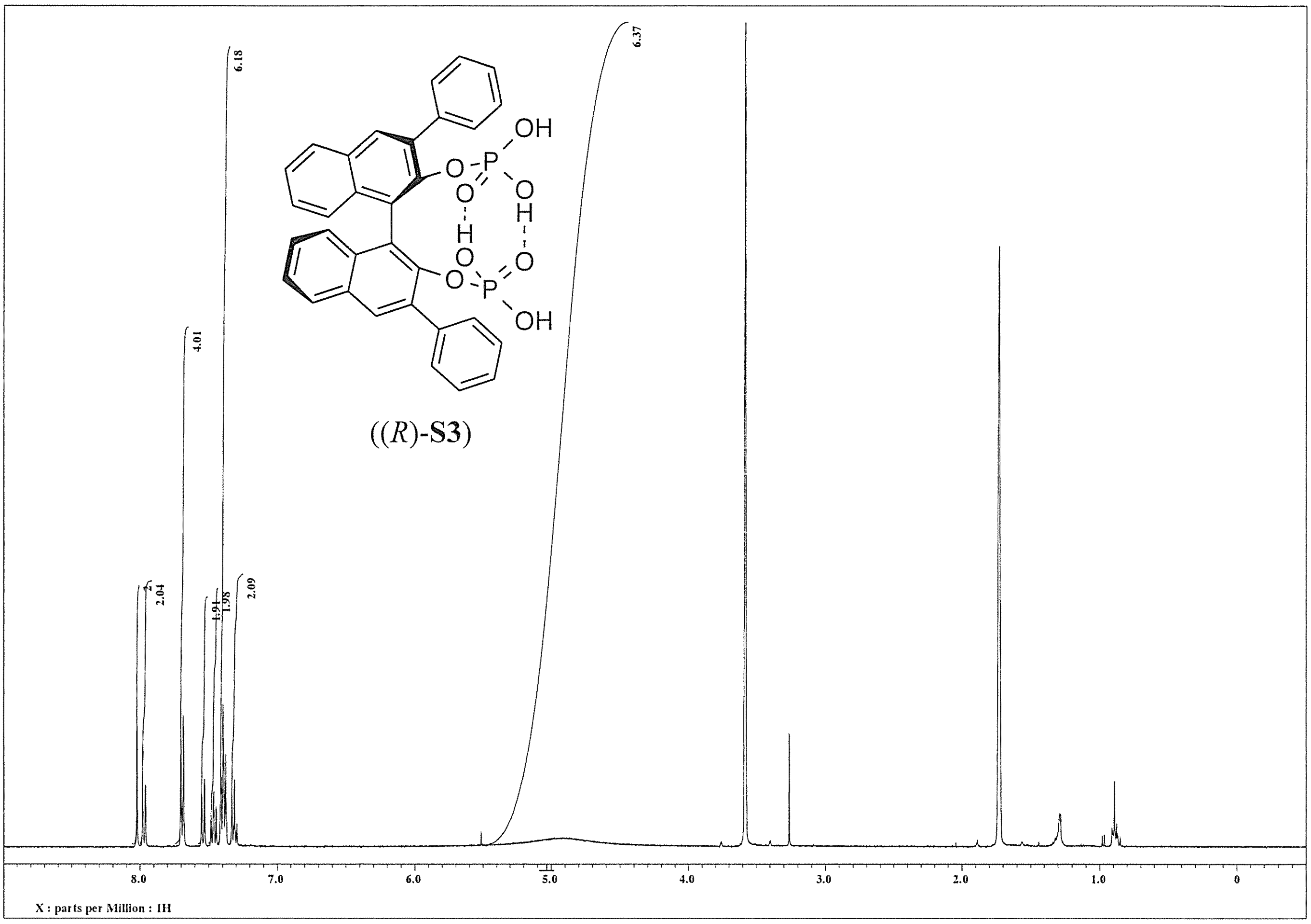

^{1}H NMR, 400 MHz, THF-d_8

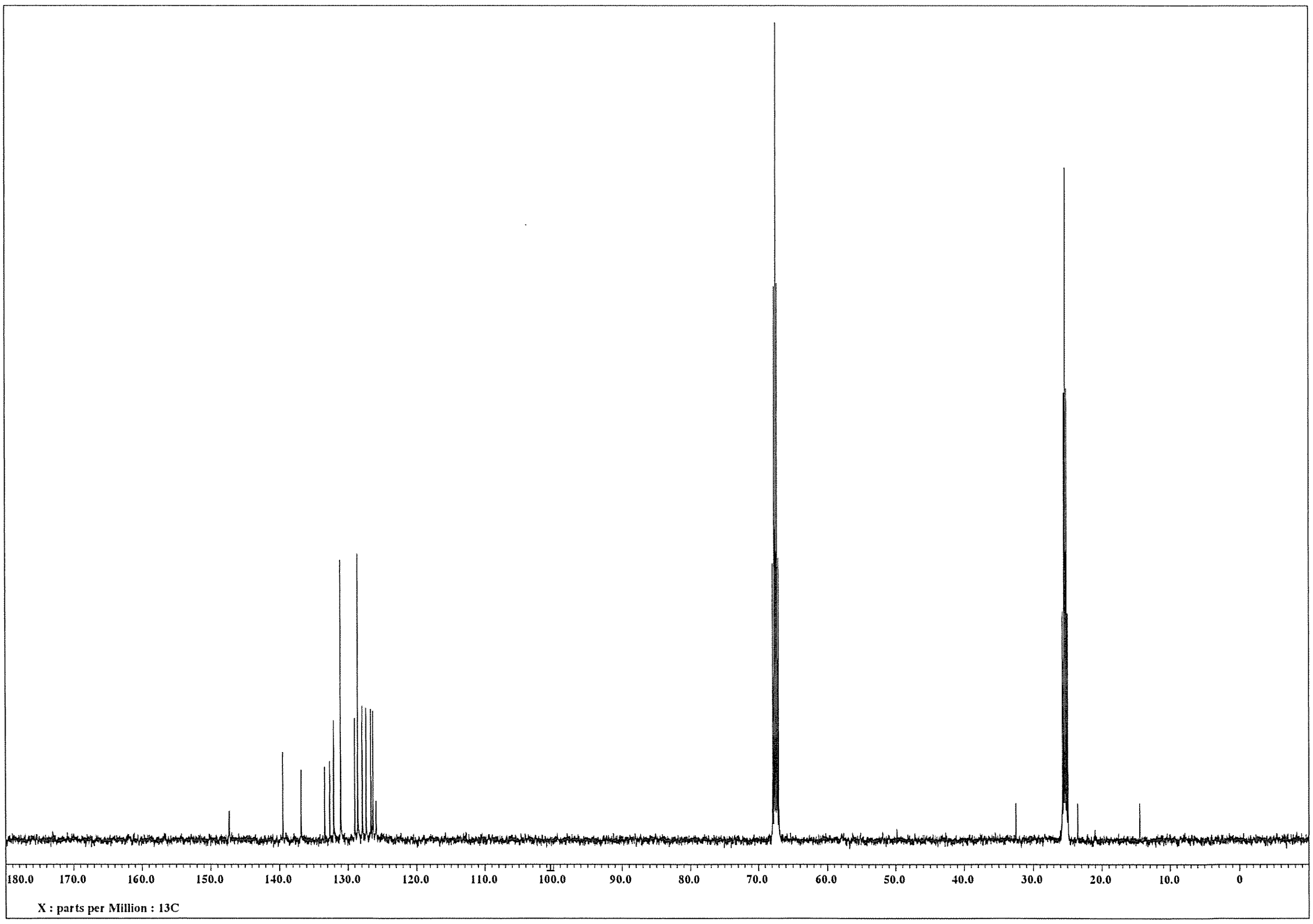

^{13}C NMR, 100 MHz, THF-d_8

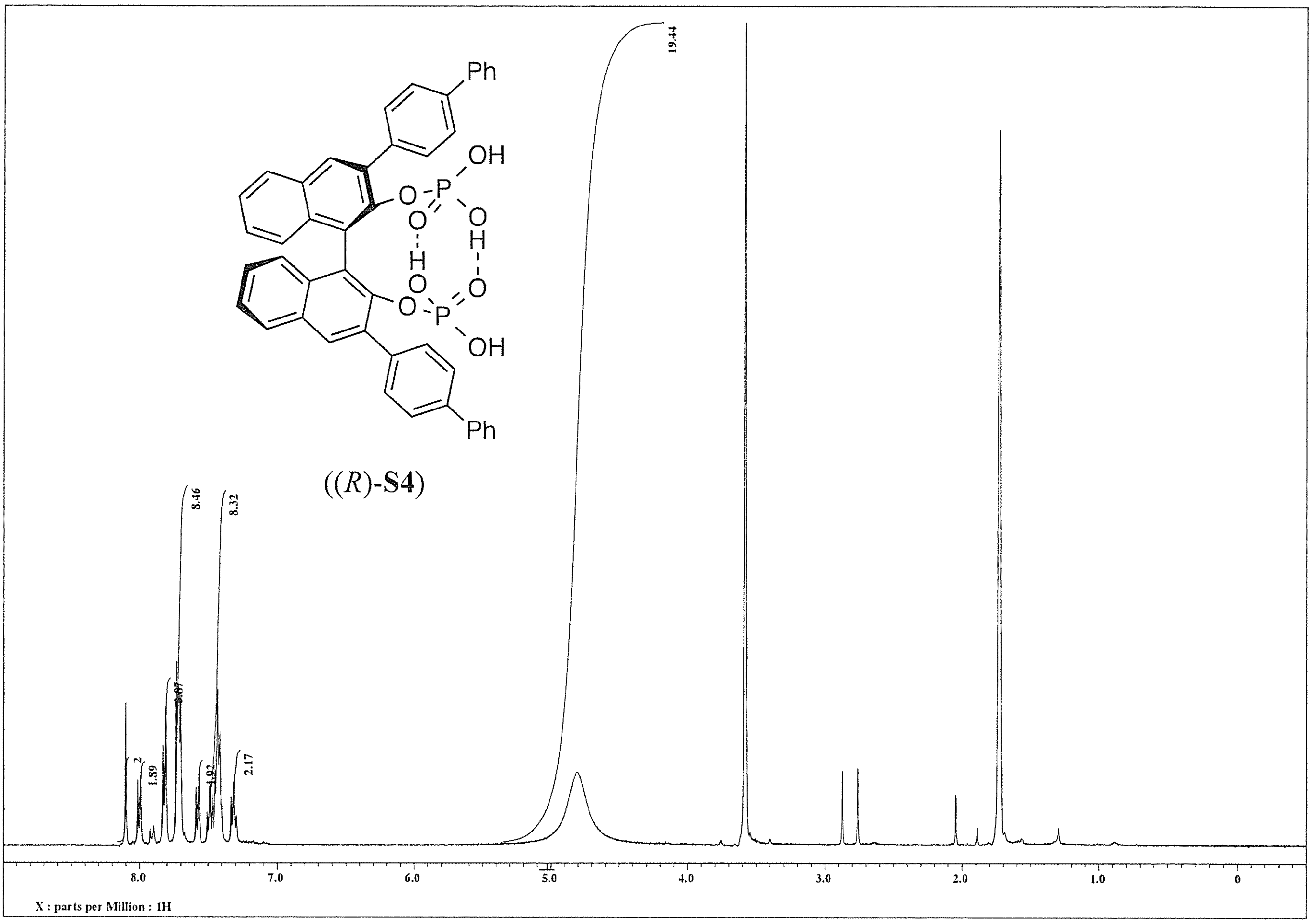

^{1}H NMR, 400 MHz, THF-d_8

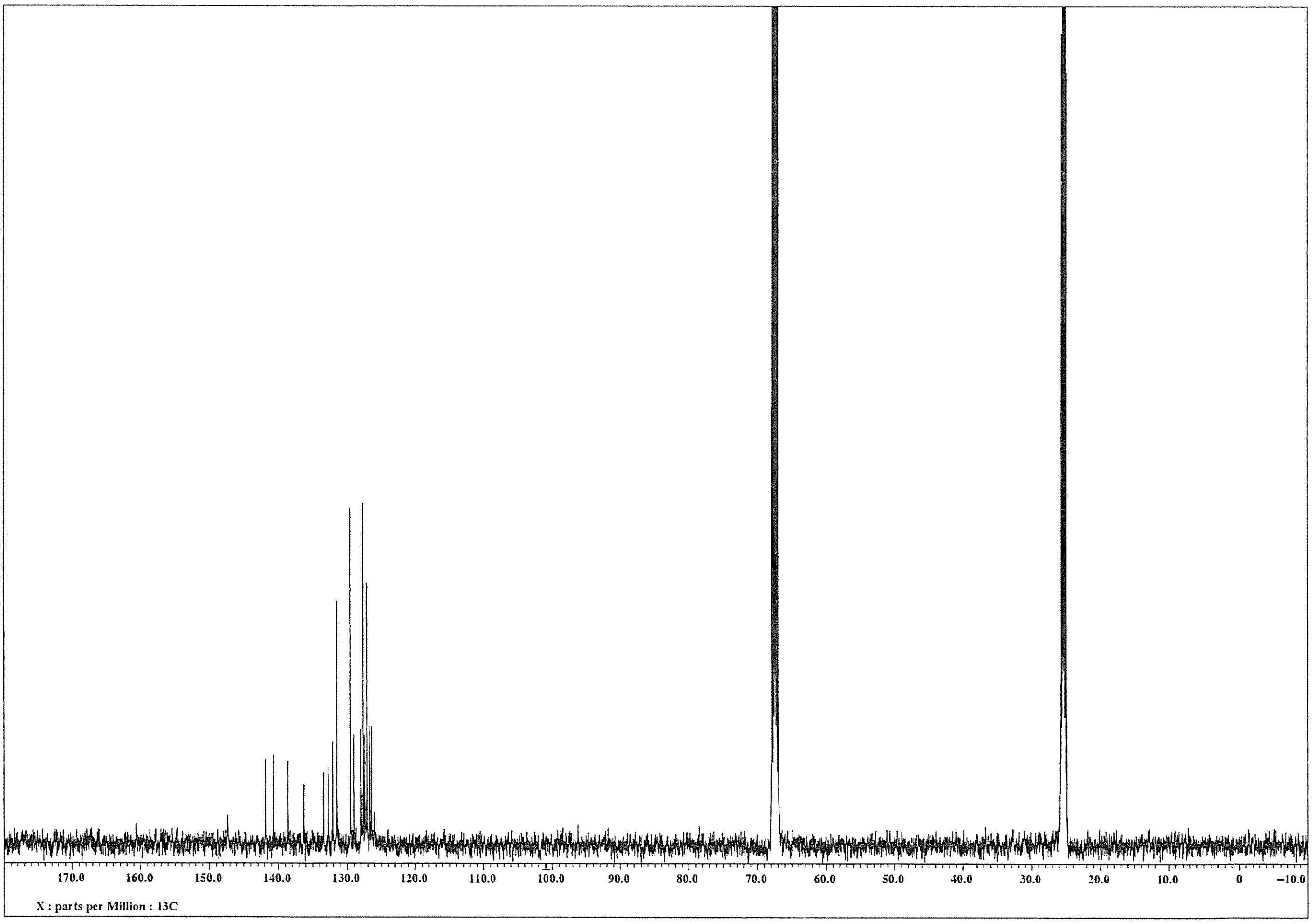

^{13}C NMR, 100 MHz, THF-d_8

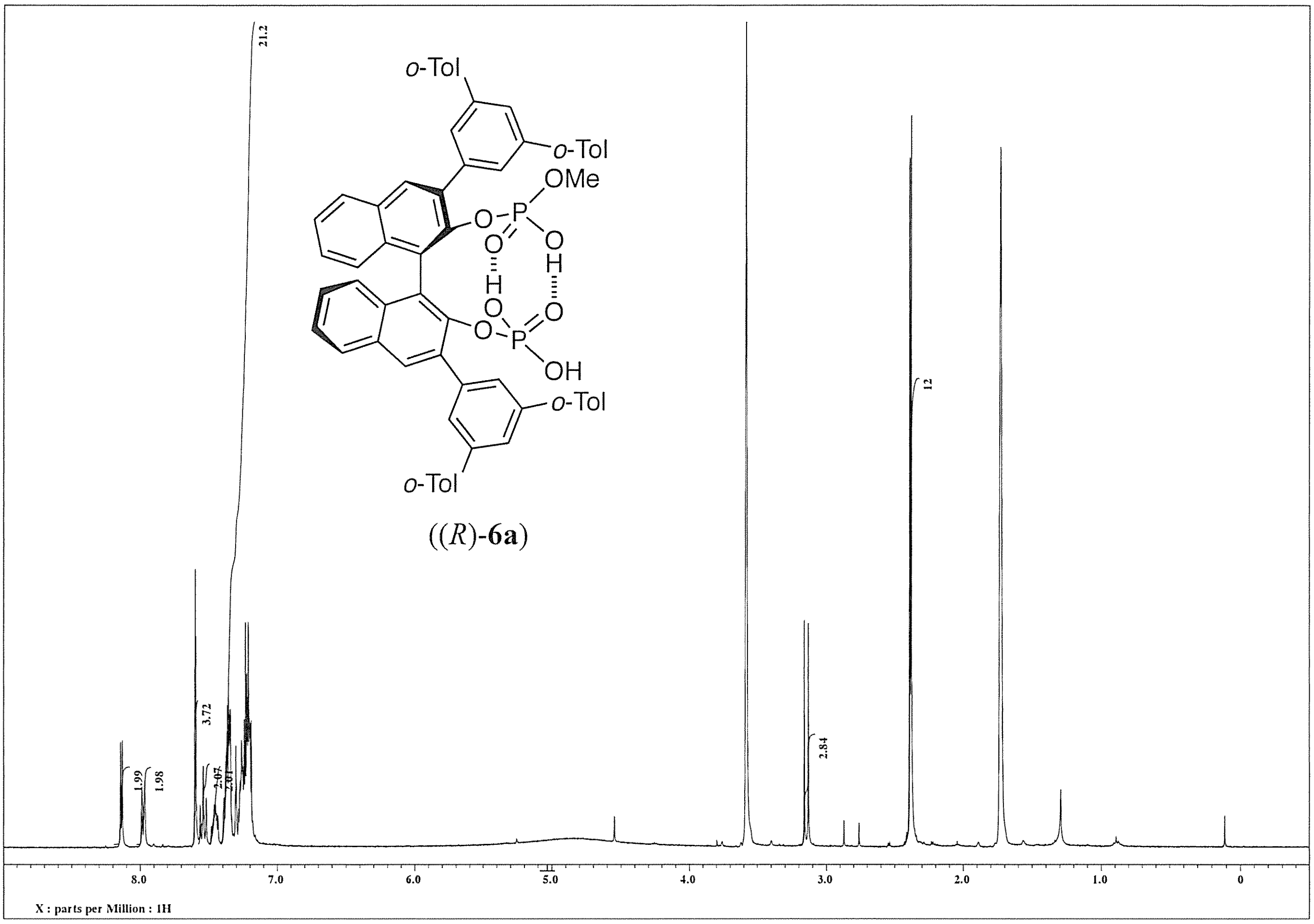

^{1}H NMR, 400 MHz, THF-d_8

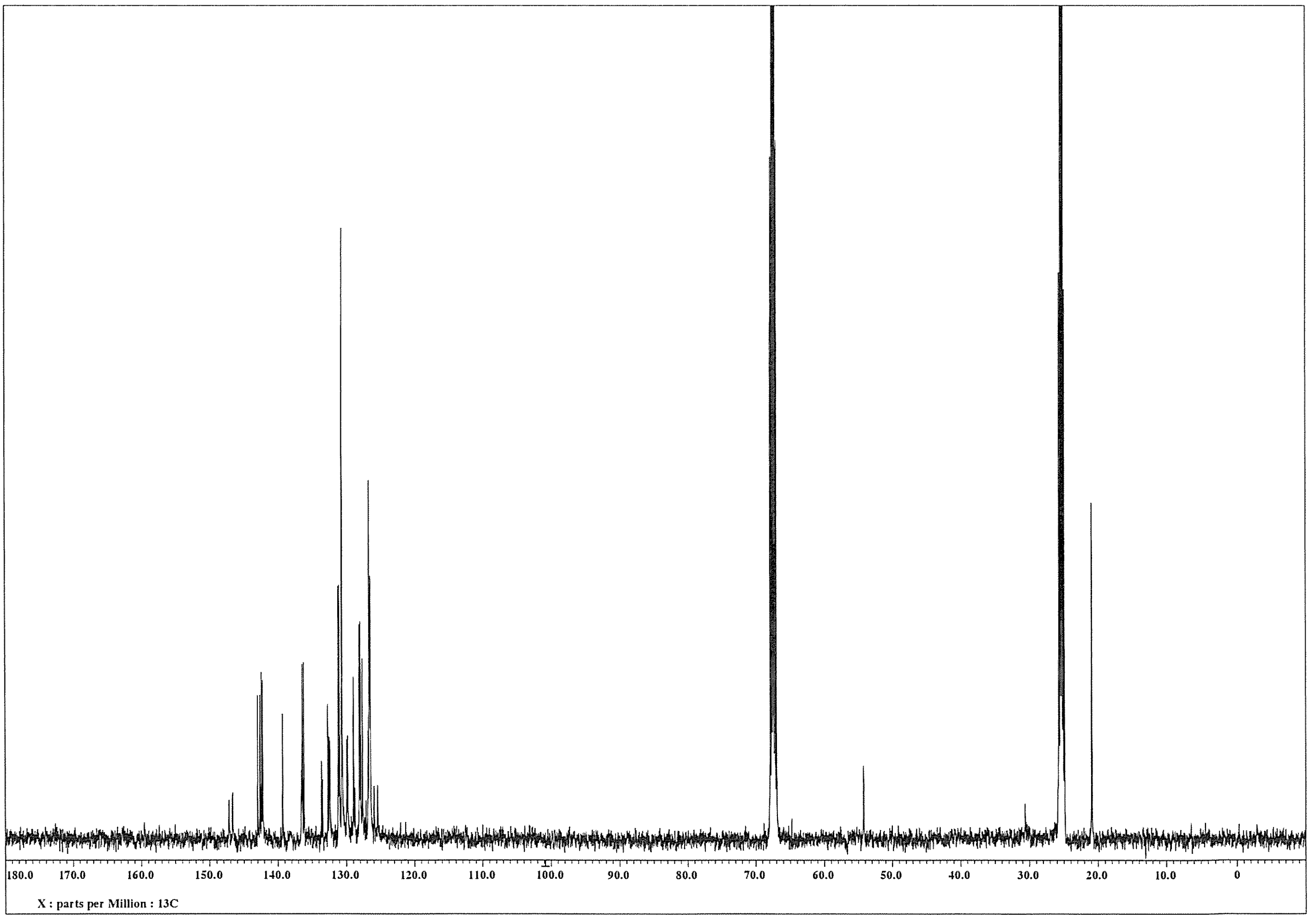

^{13}C NMR, 100 MHz, THF-d_8

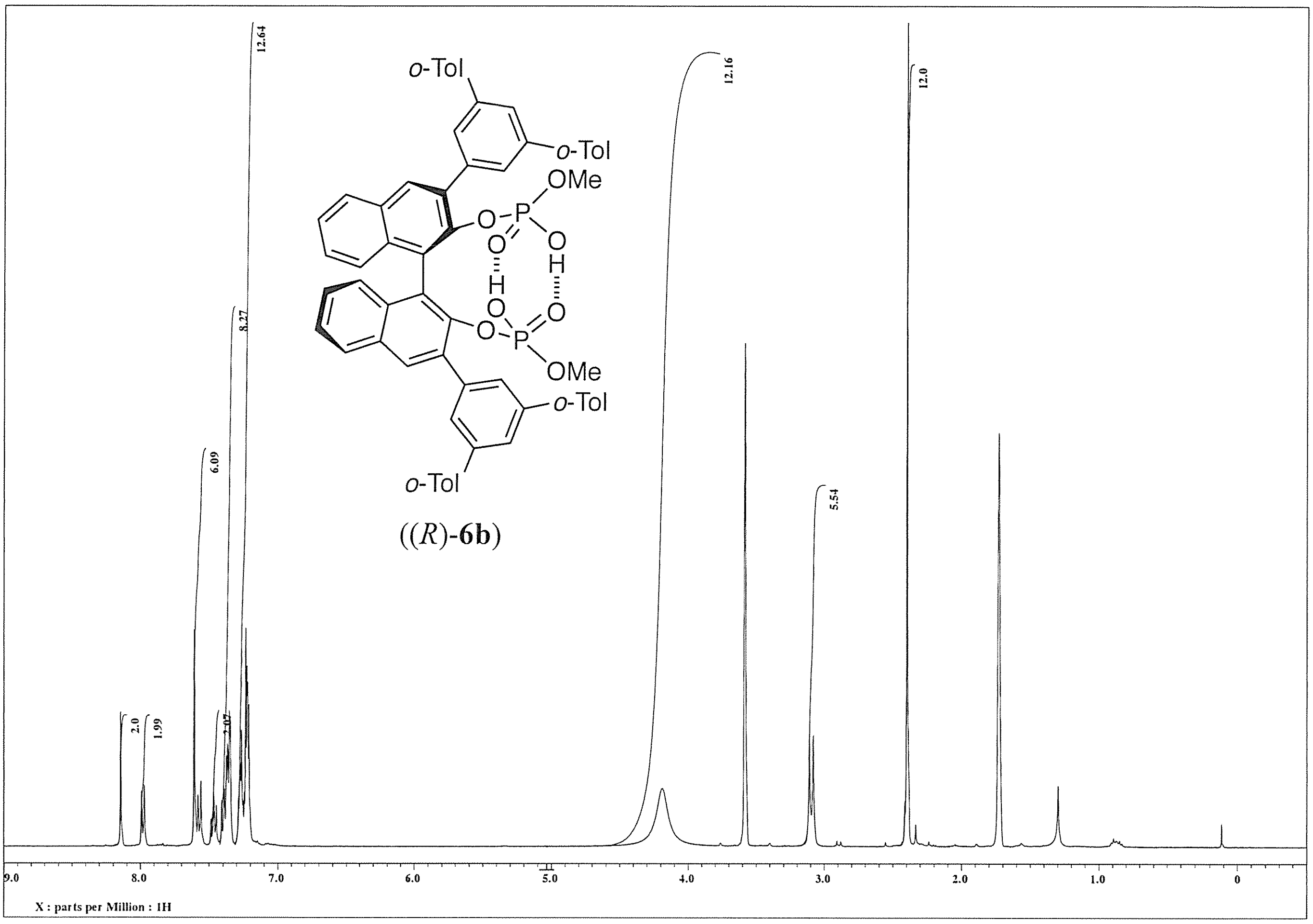

^{1}H NMR, 400 MHz, THF-d_8

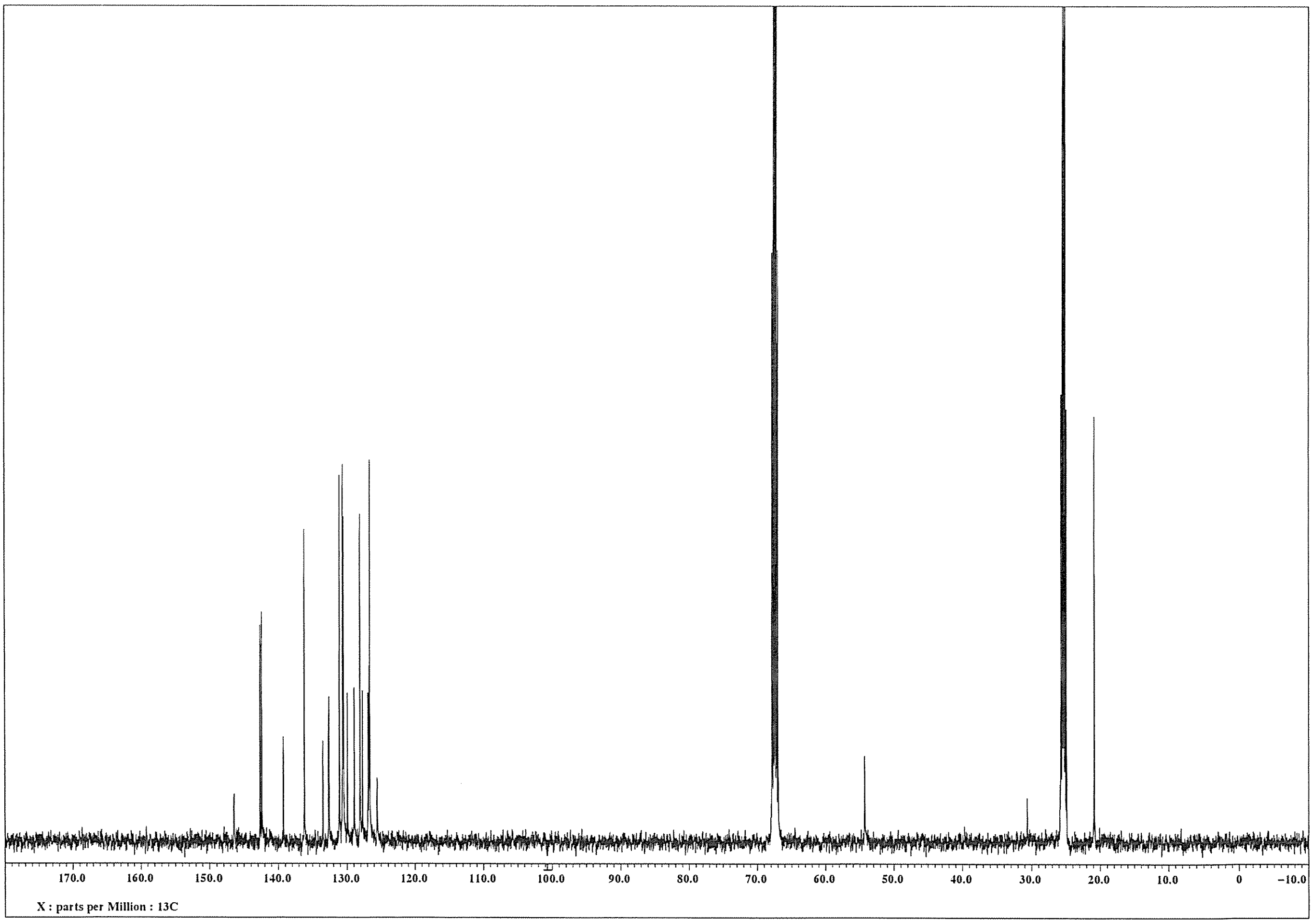

^{13}C NMR, 100 MHz, THF-d_8

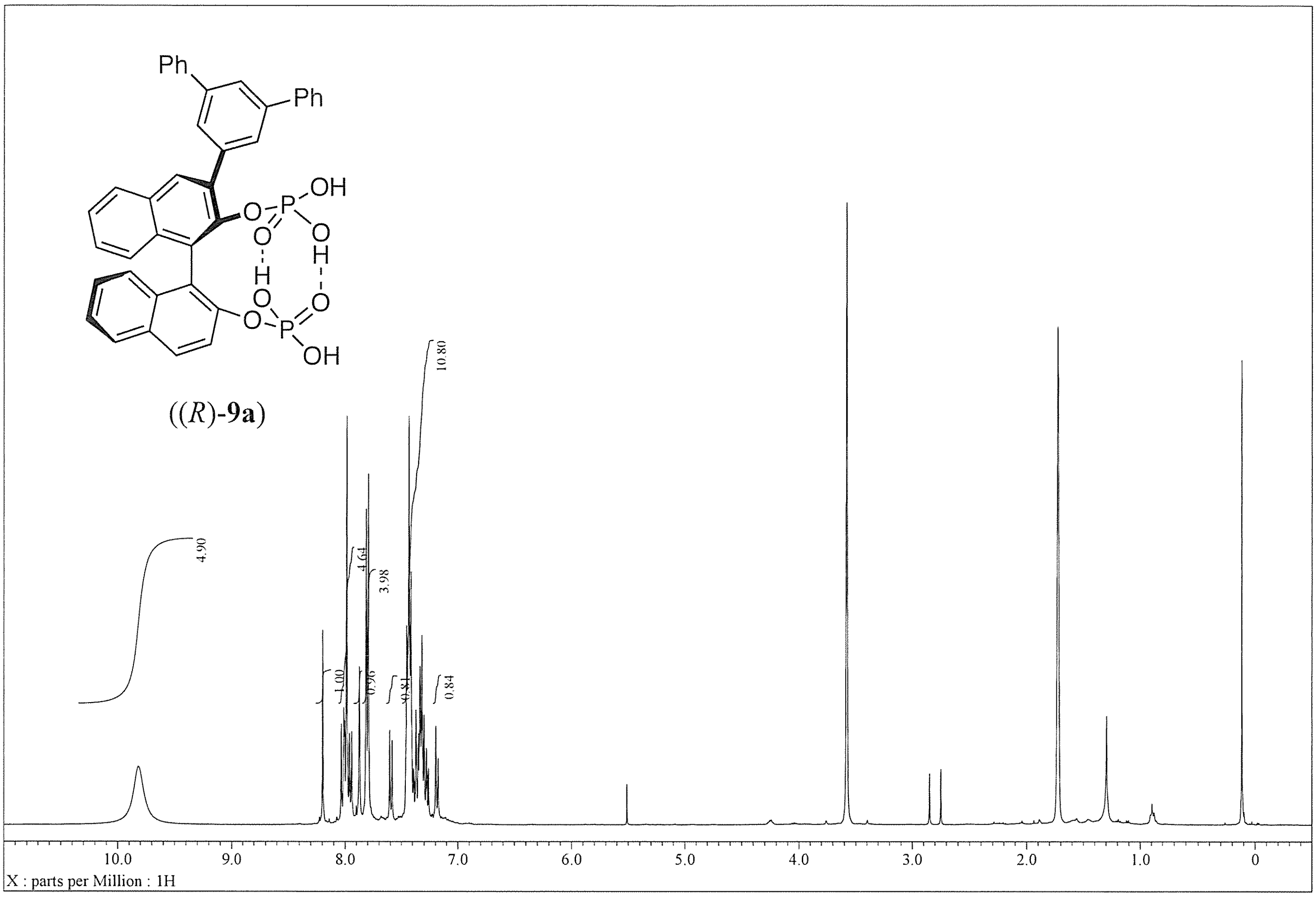

^{1}H NMR, 400 MHz, THF-d_8

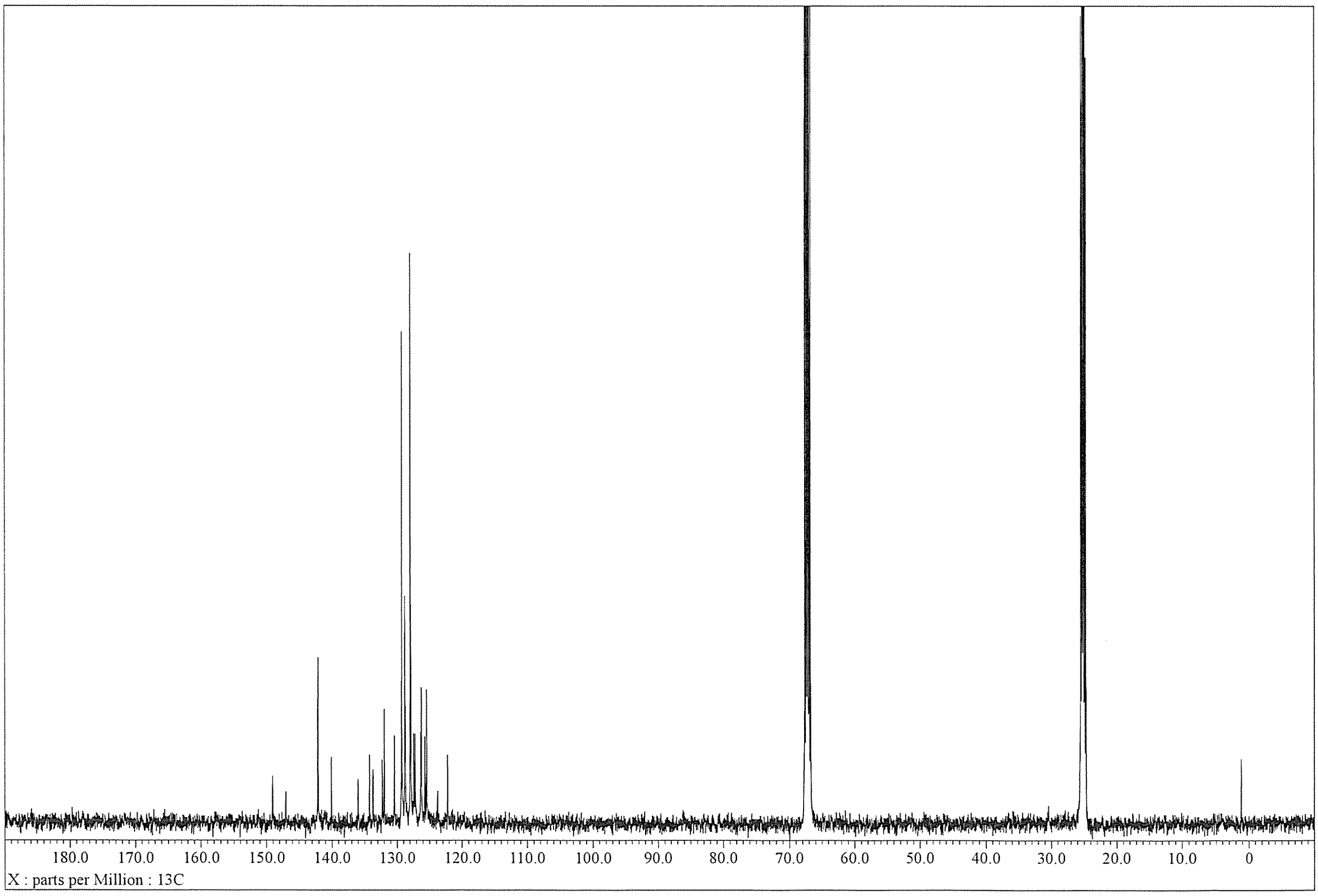

^{13}C NMR, 100 MHz, THF-d_8

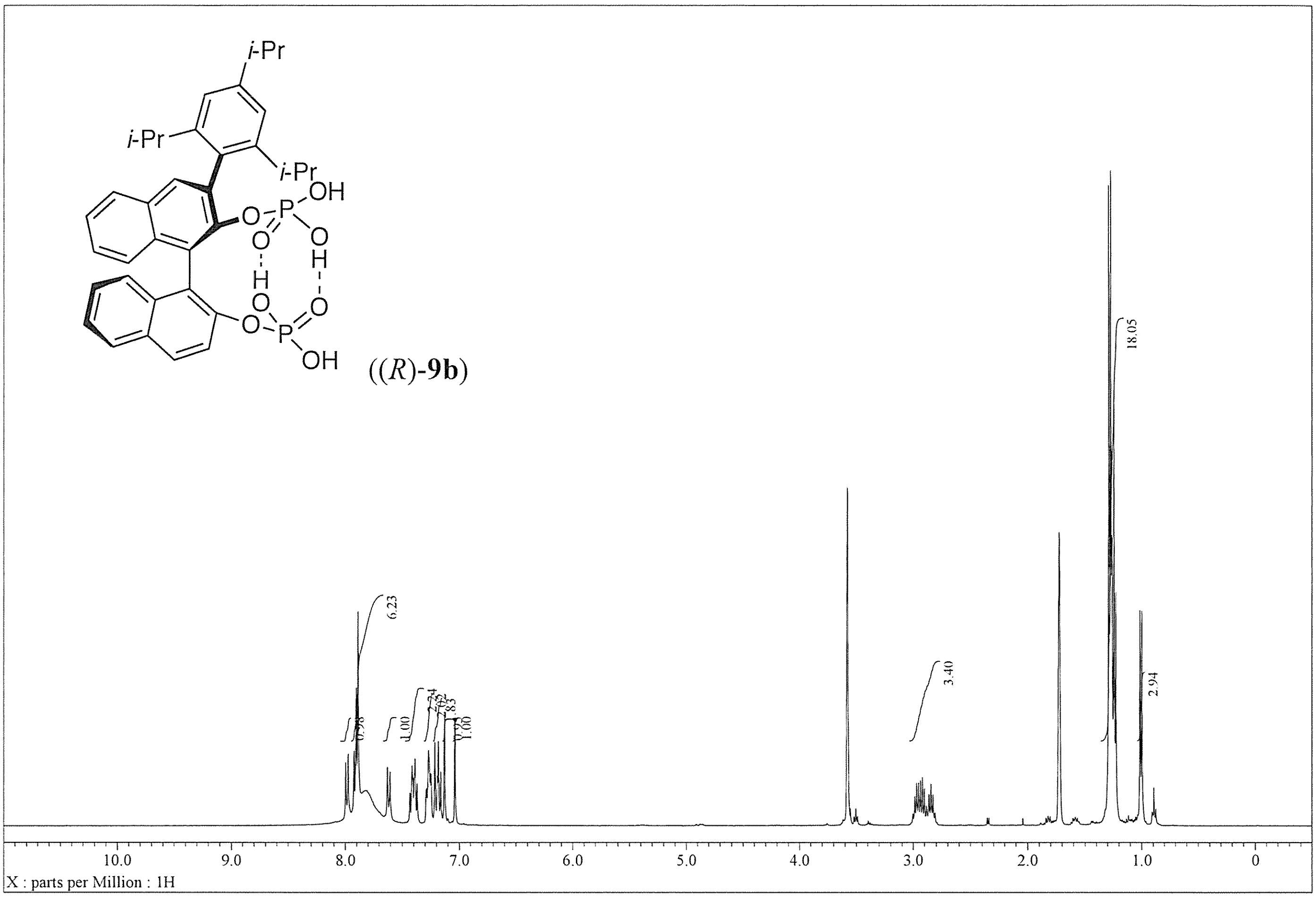

^{1}H NMR, 400 MHz, THF-d_8

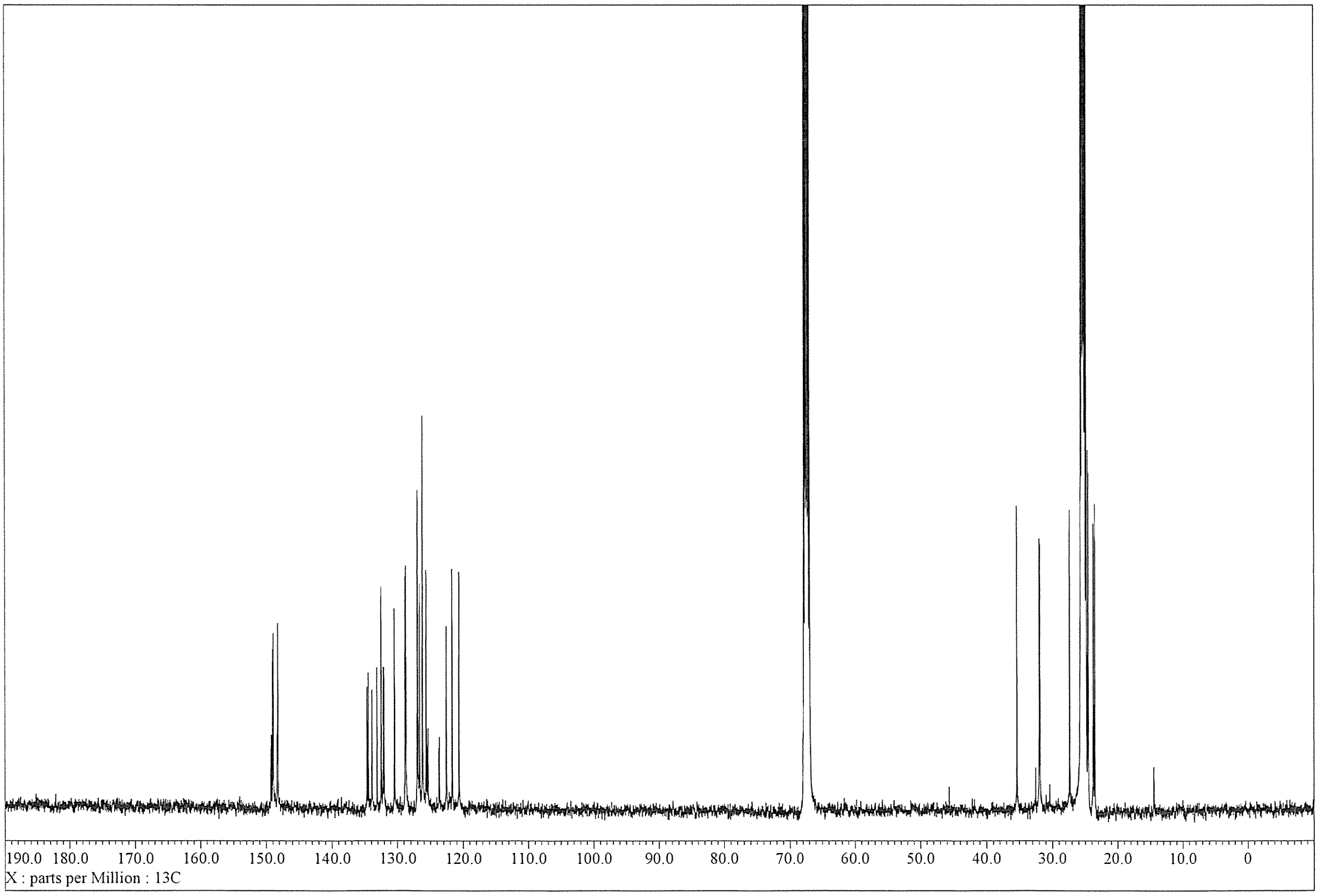

^{13}C NMR, 100 MHz, THF-d_8

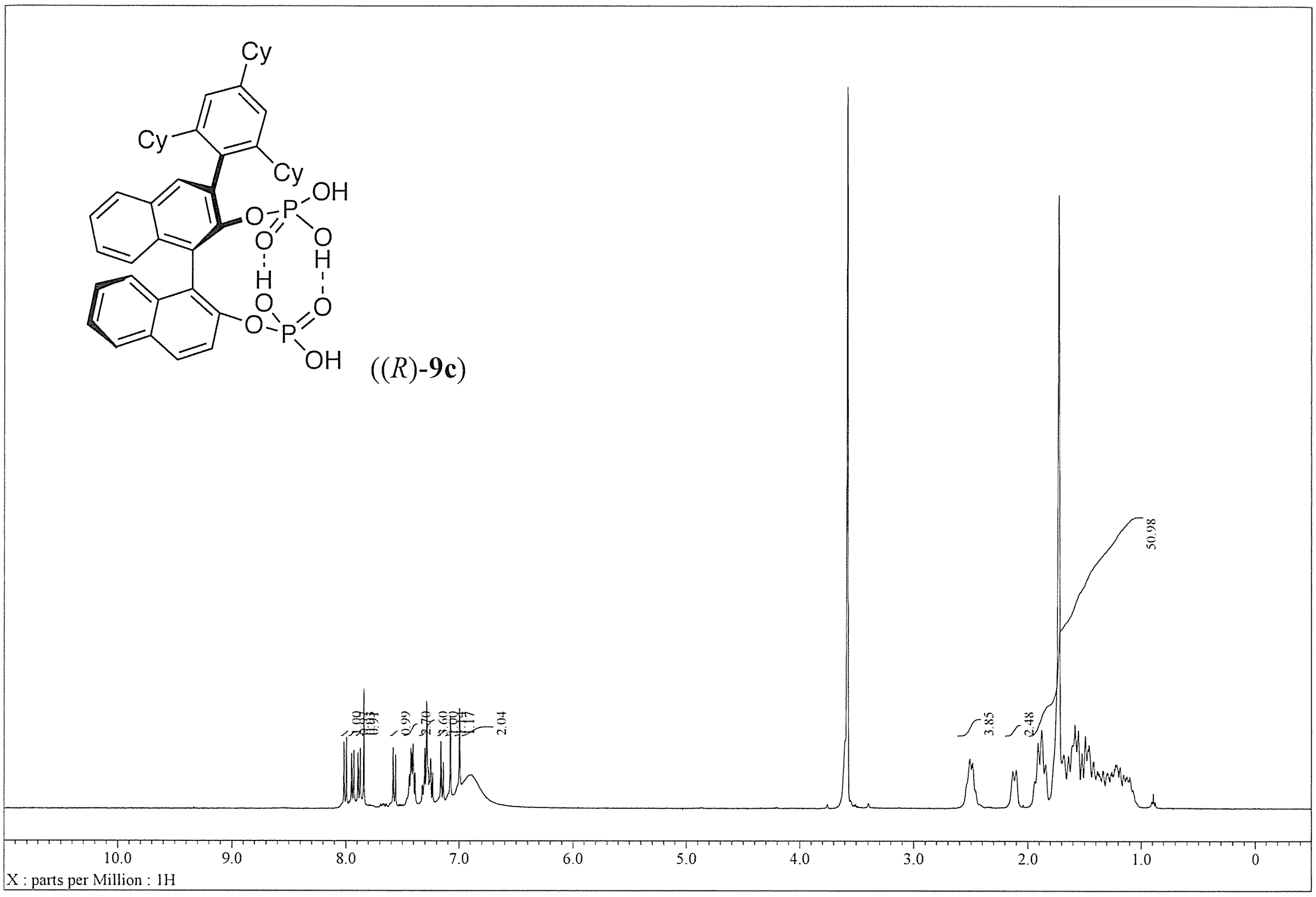

^{1}H NMR, 400 MHz, THF-d_8

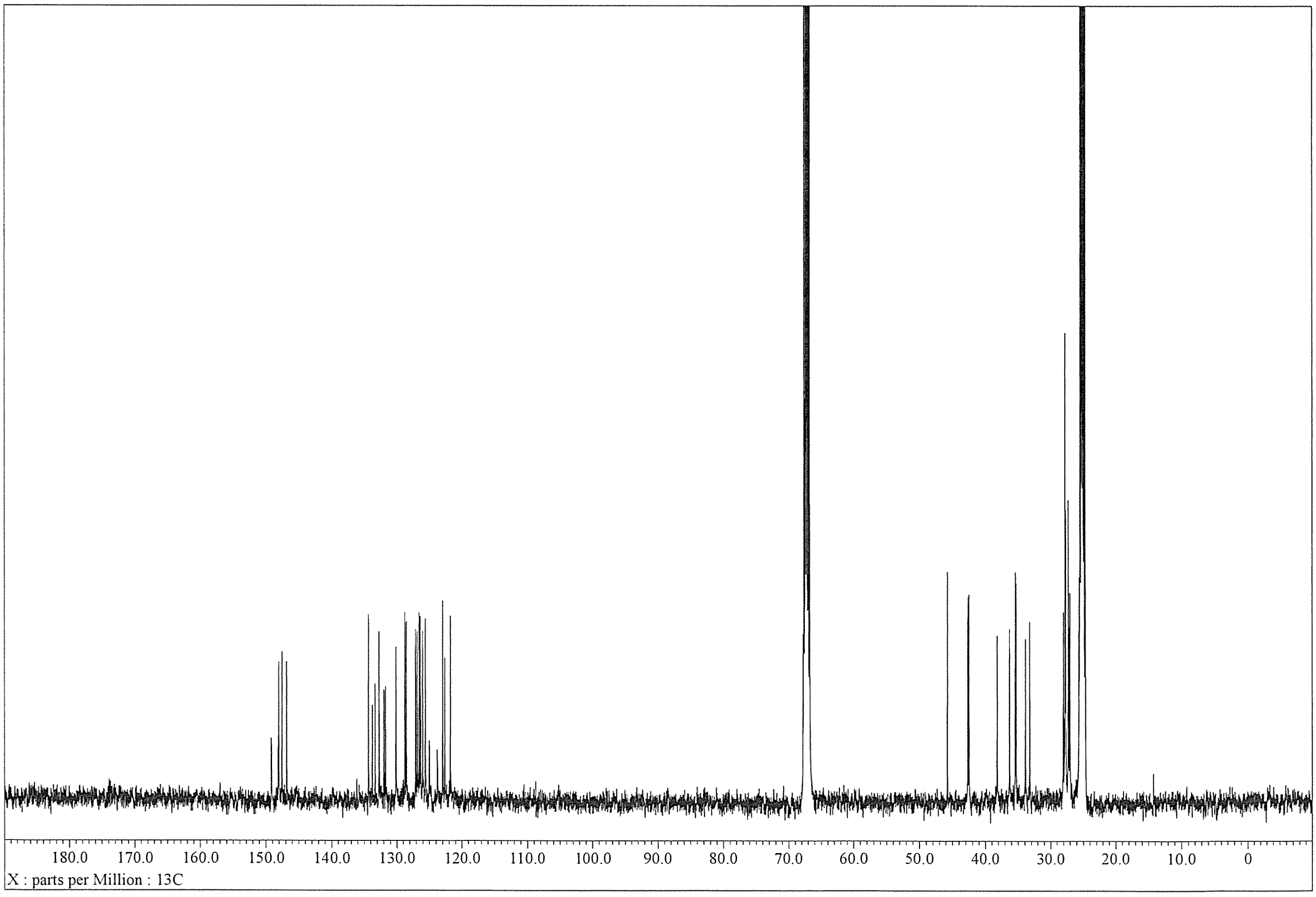

^{13}C NMR, 100 MHz, THF-d_8

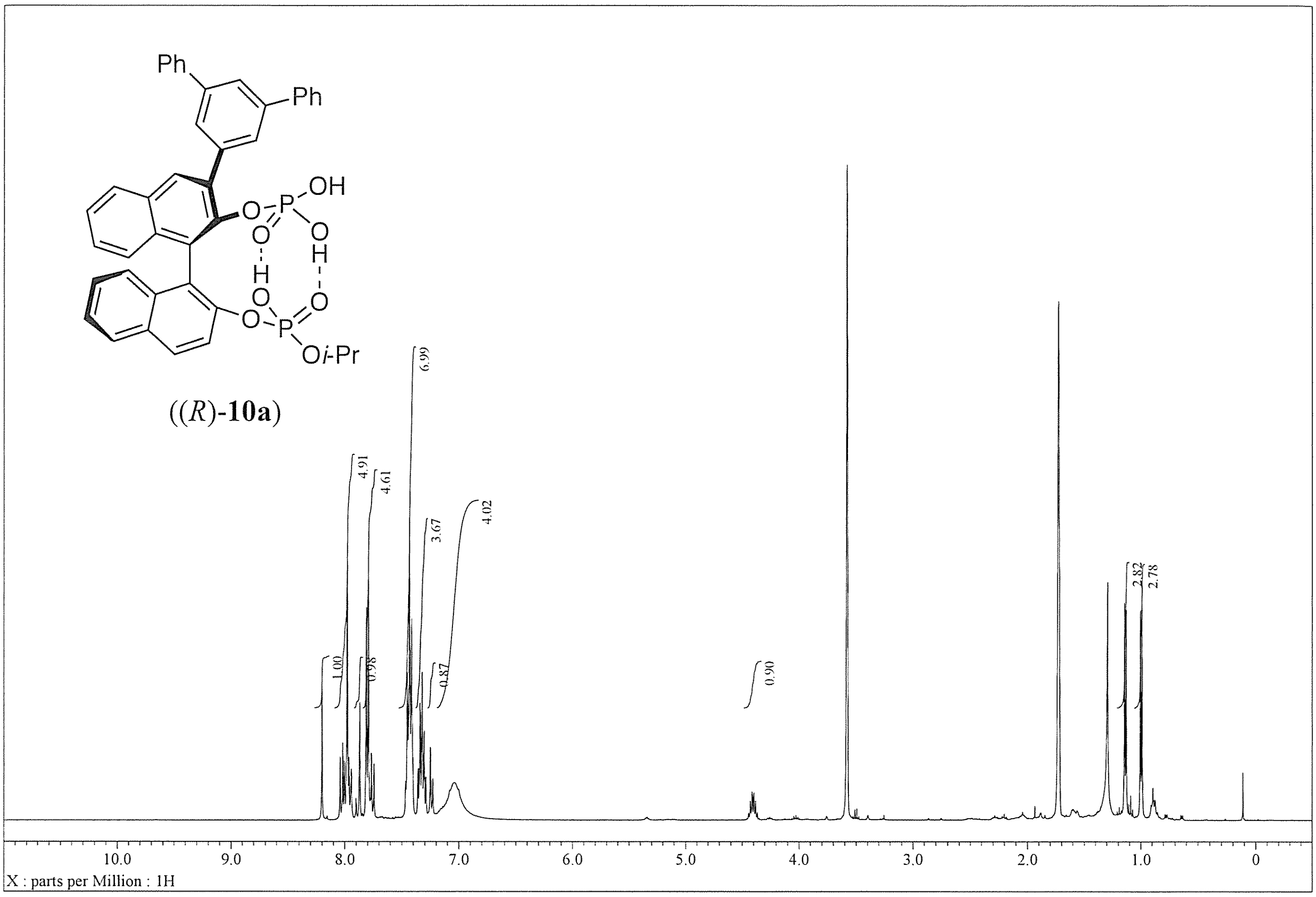

^{1}H NMR, 400 MHz, THF-d_8

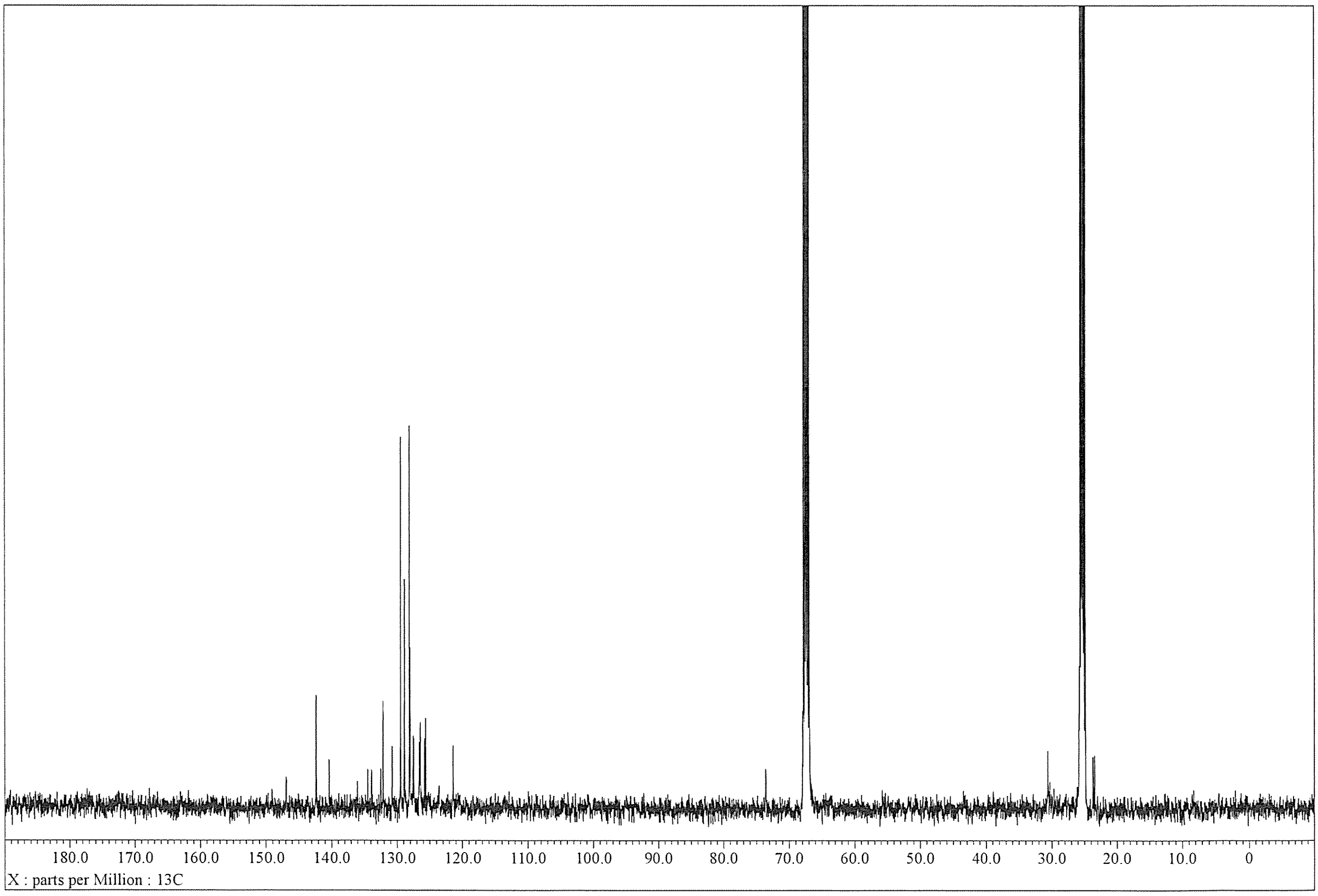

^{13}C NMR, 100 MHz, THF-d_8

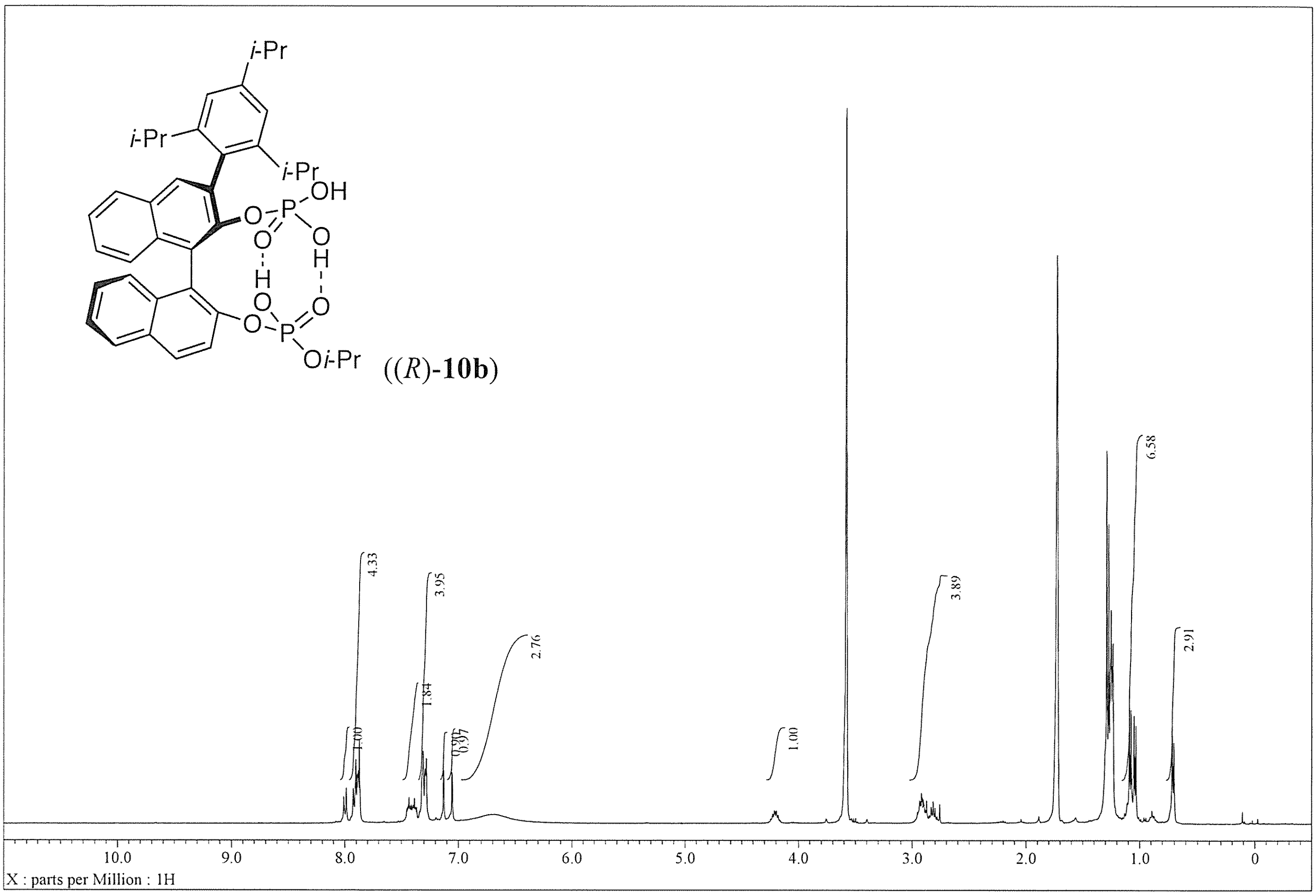

^{1}H NMR, 400 MHz, THF-d_8

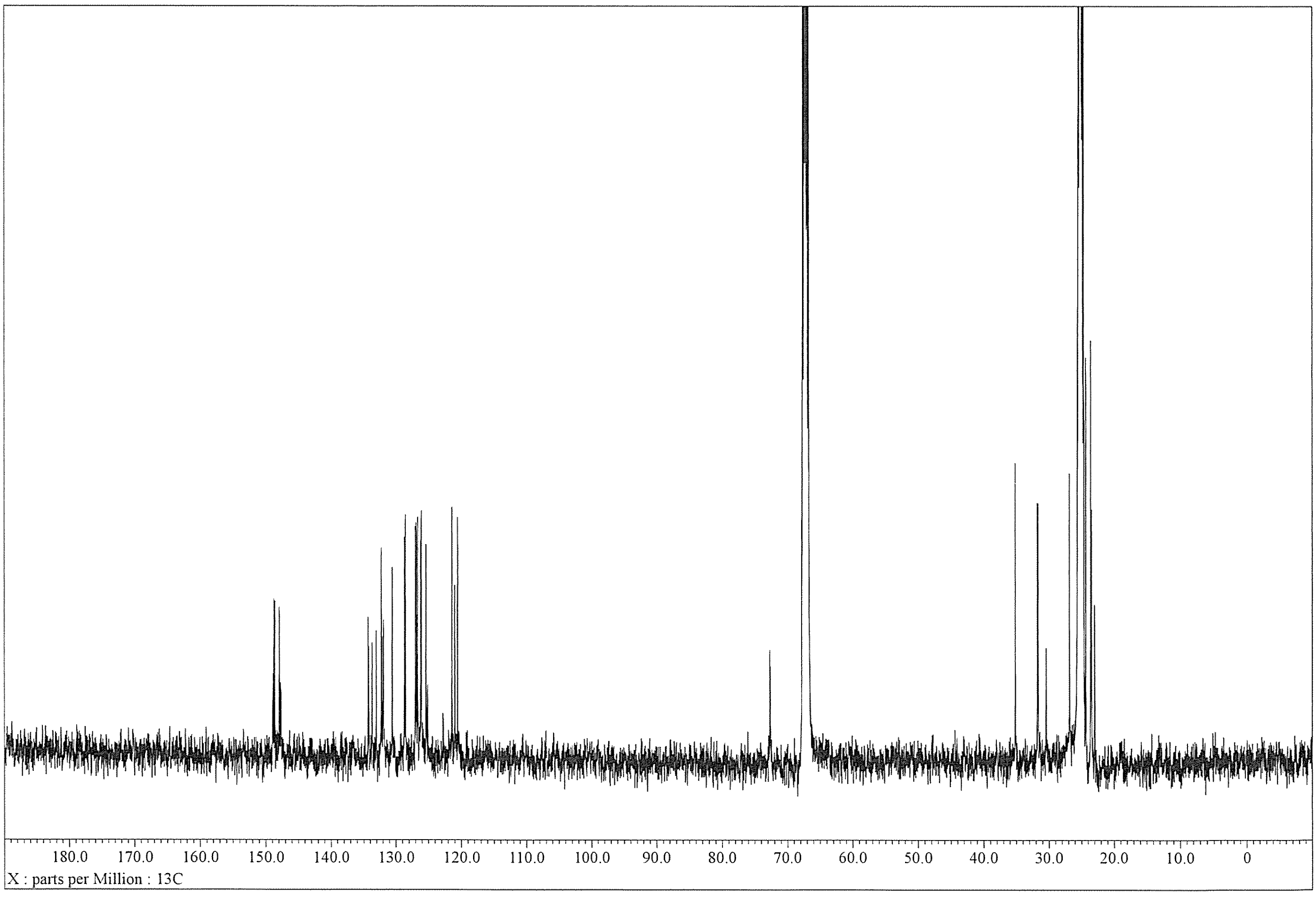

^{13}C NMR, 100 MHz, THF-d_8

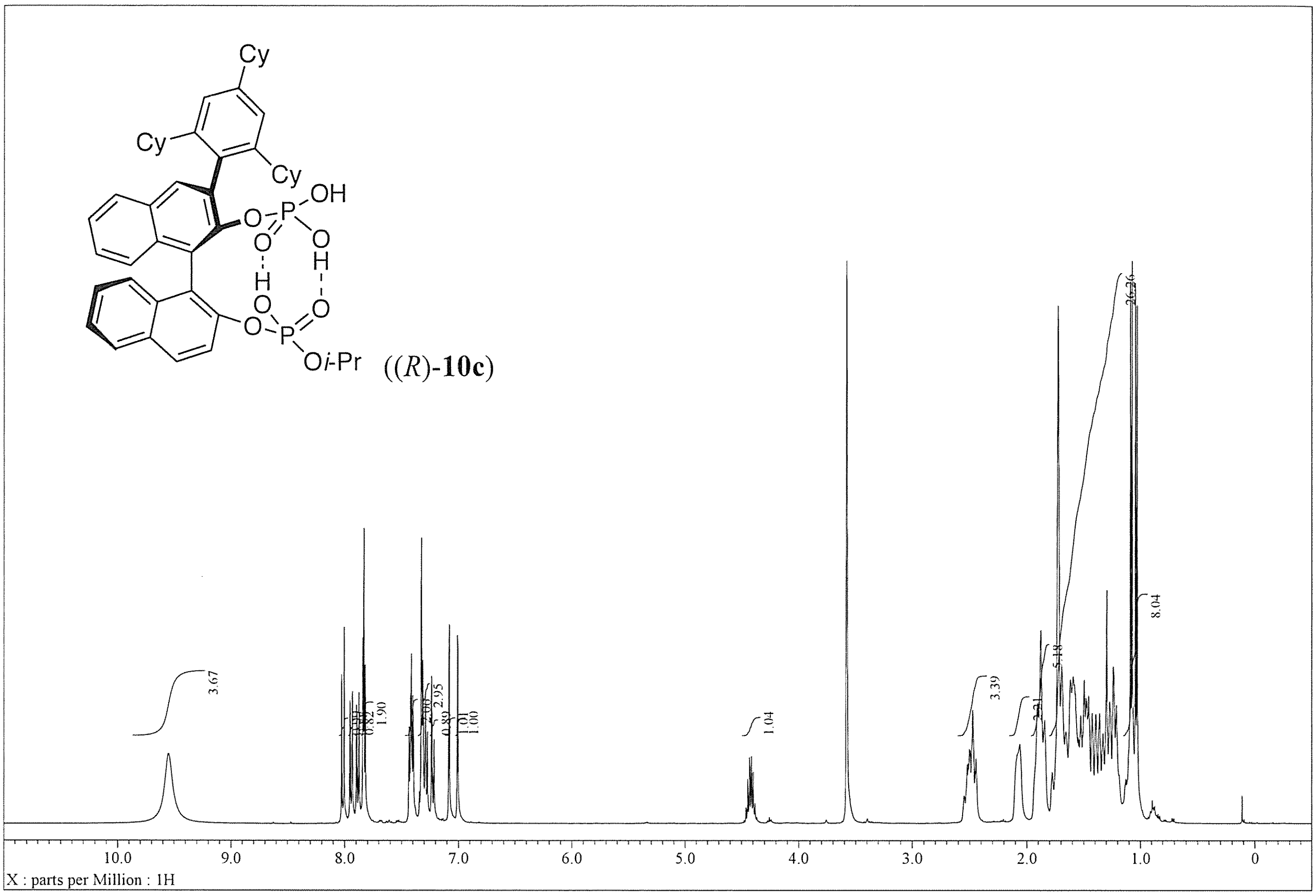

^{1}H NMR, 400 MHz, THF-d_8

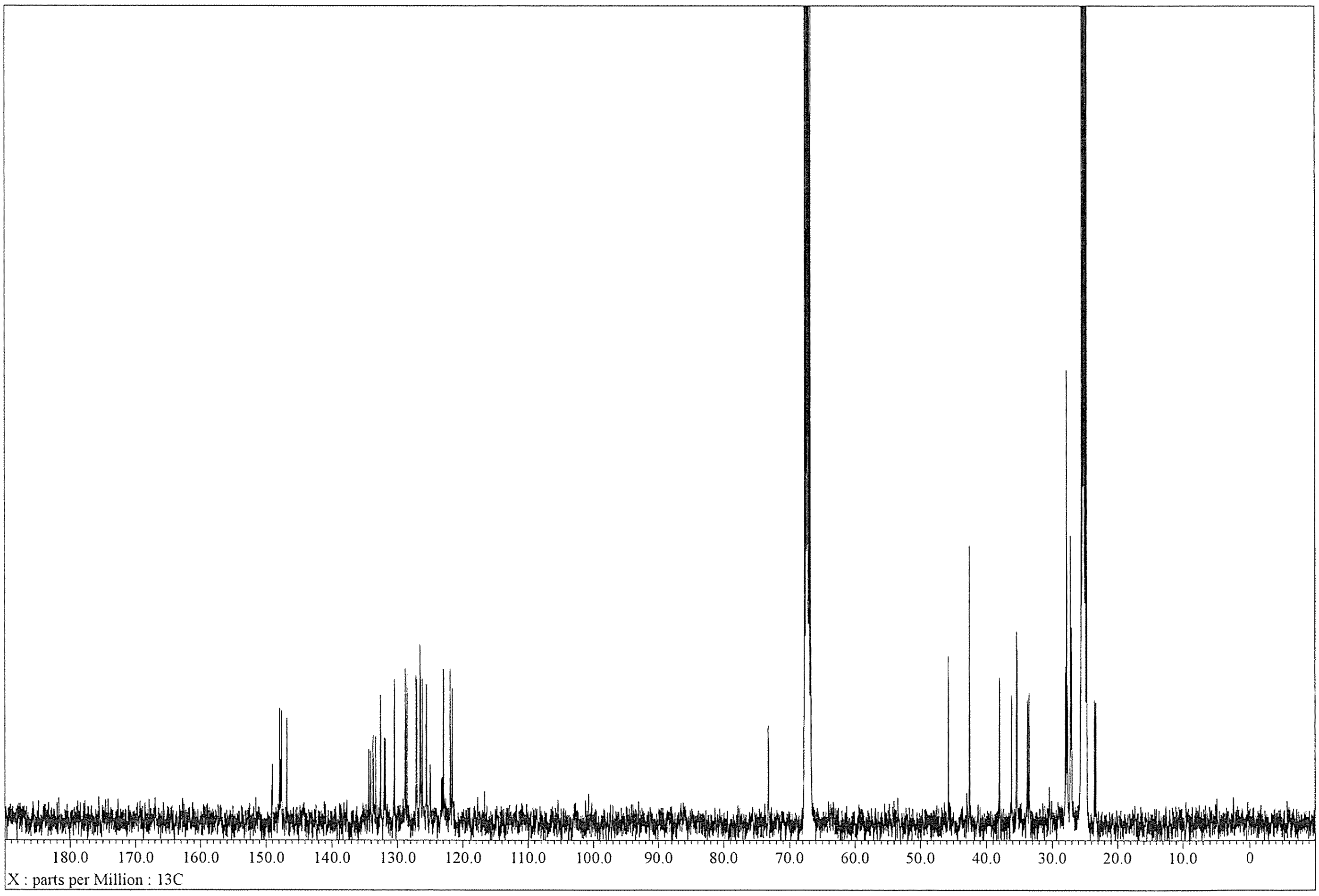

^{13}C NMR, 100 MHz, $CDCl_3$

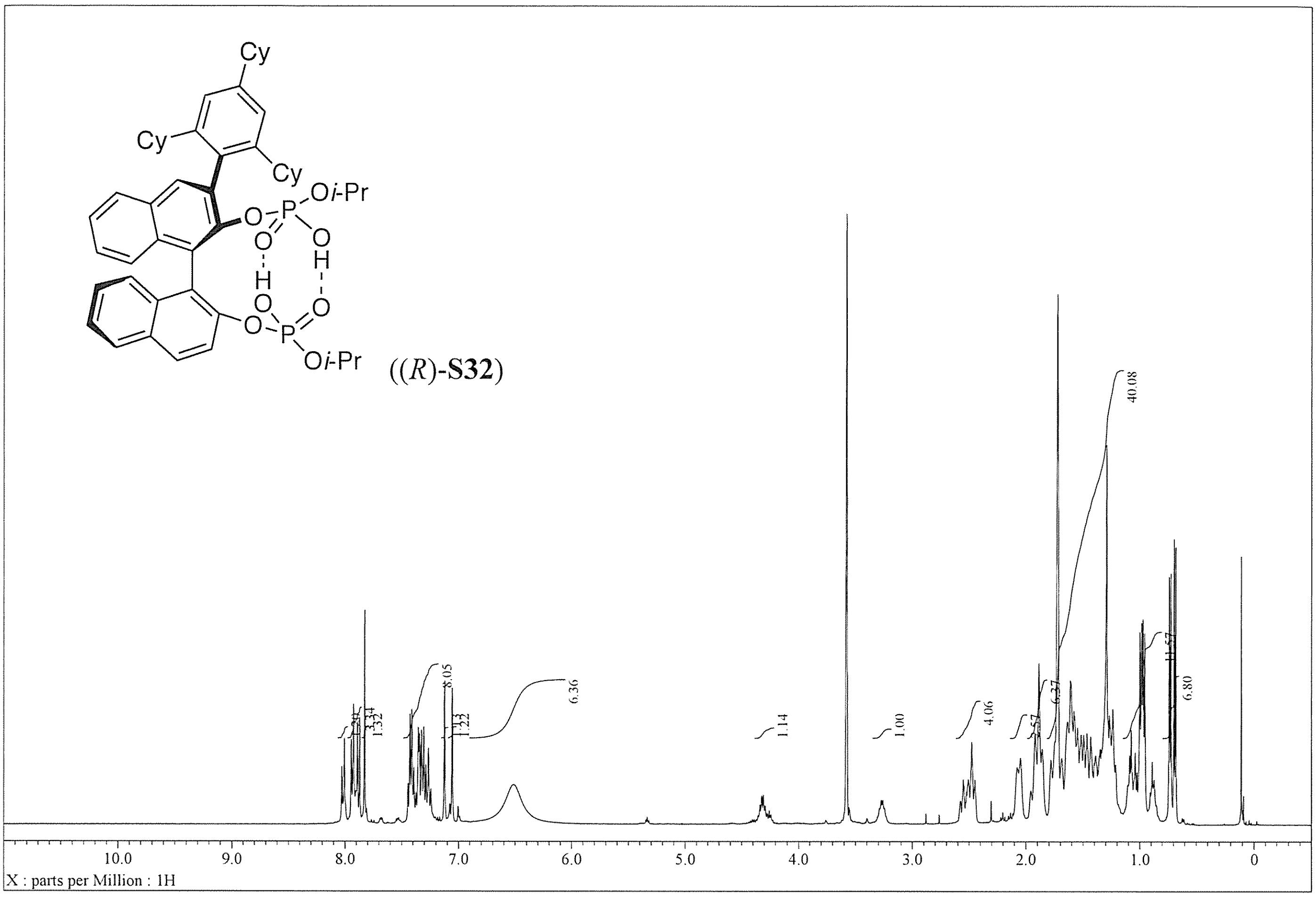

^{1}H NMR, 400 MHz, THF-d_8

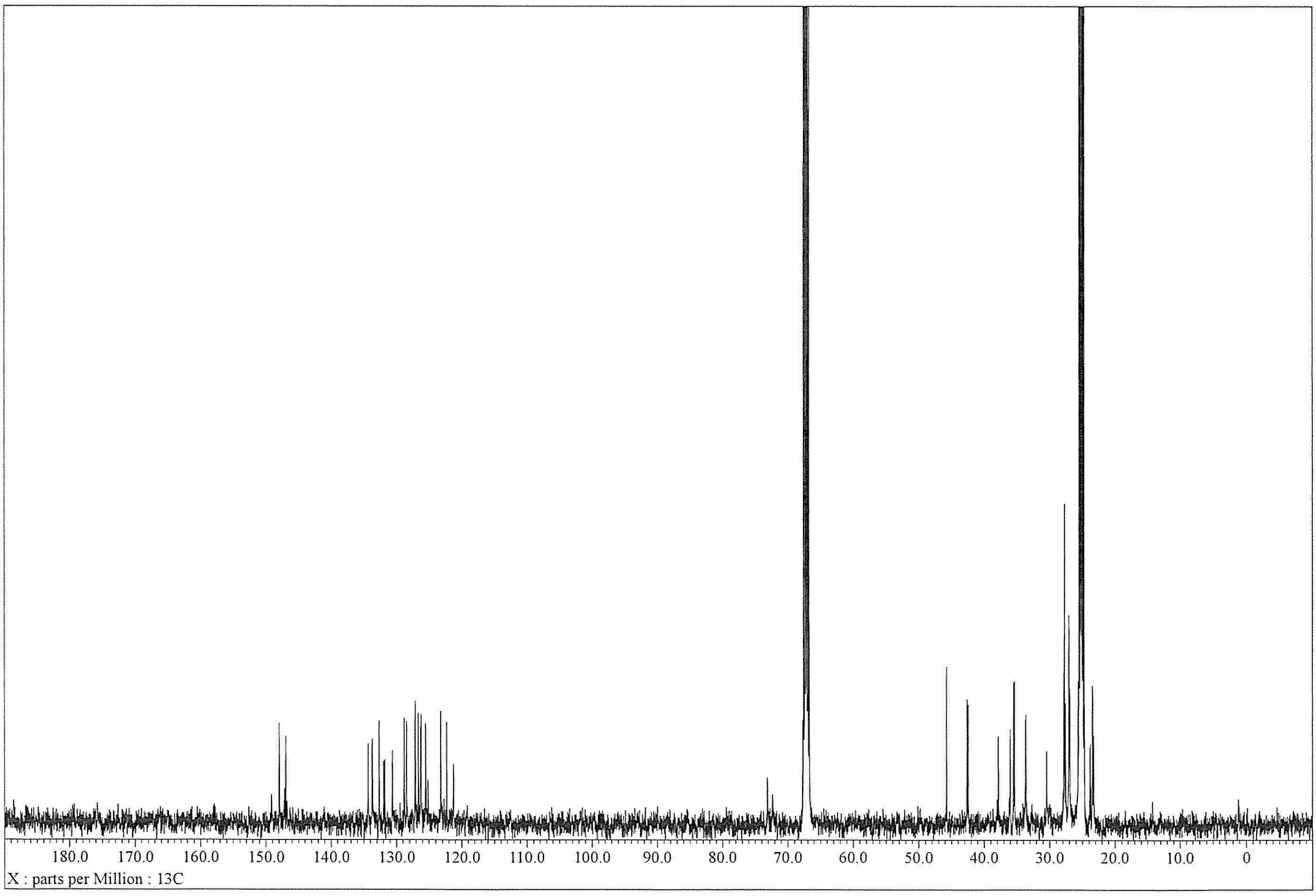

^{13}C NMR, 100 MHz, THF-d_8

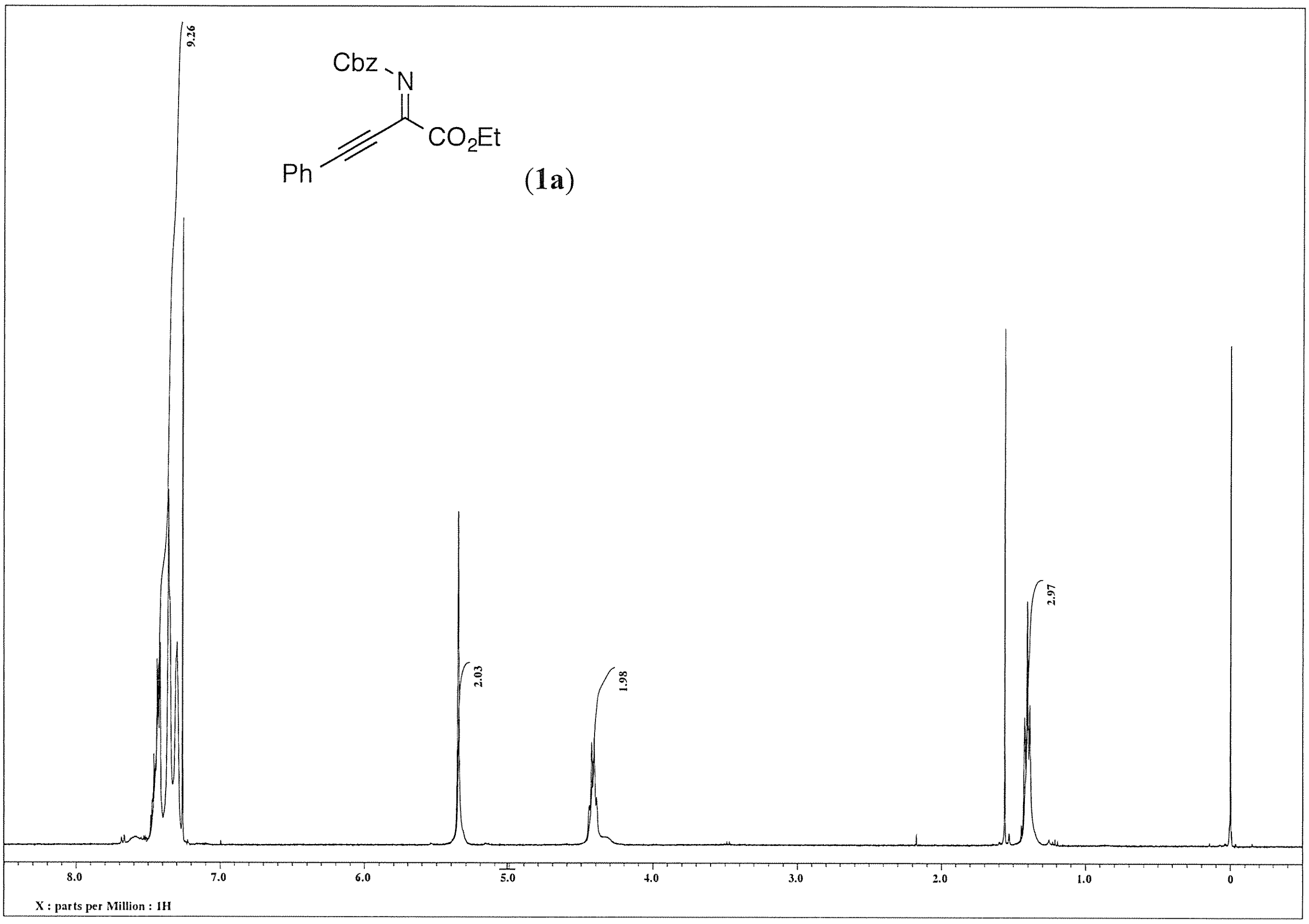

^{1}H NMR, 400 MHz, $CDCl_3$

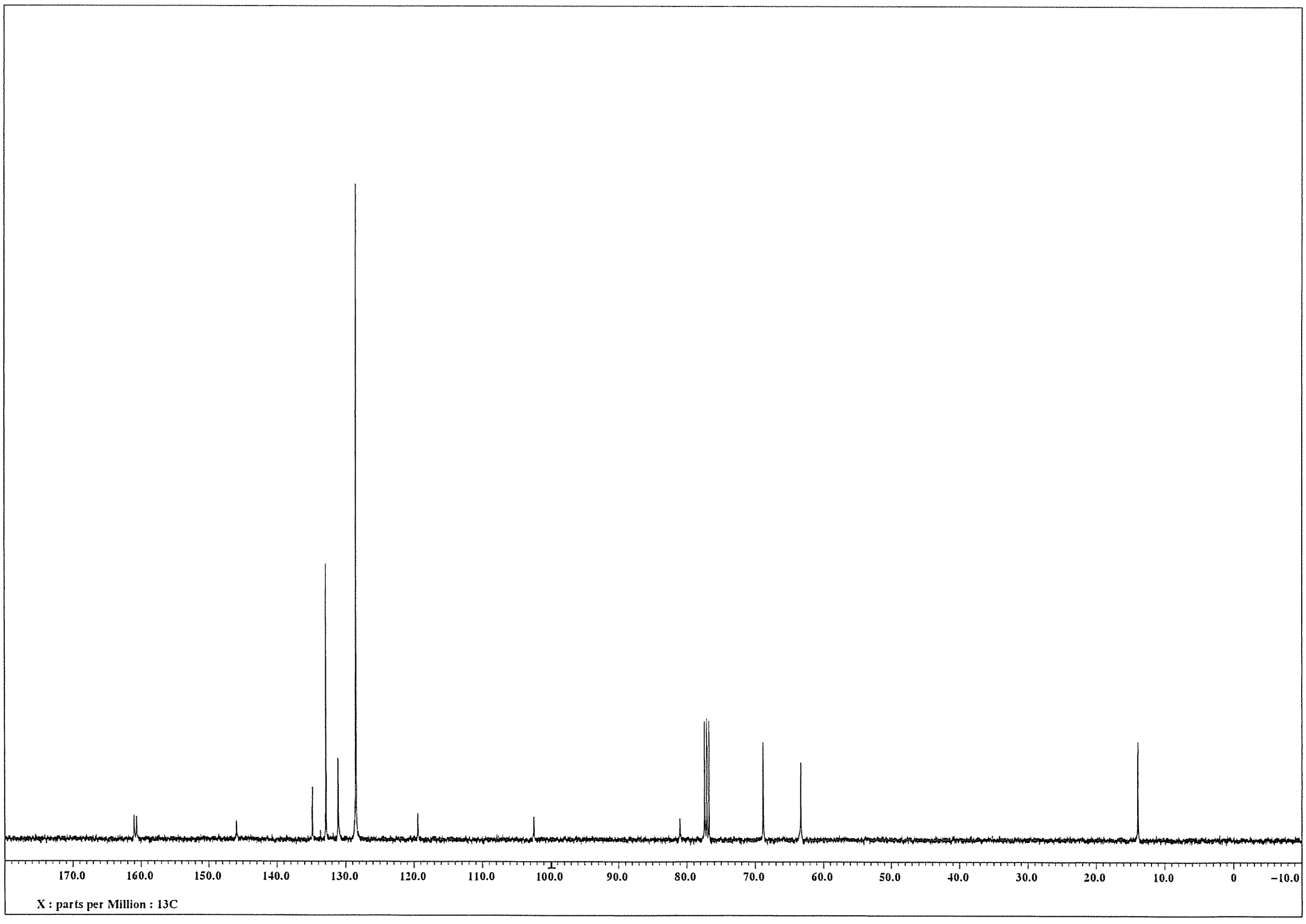

^{13}C NMR, 100 MHz, $CDCl_3$

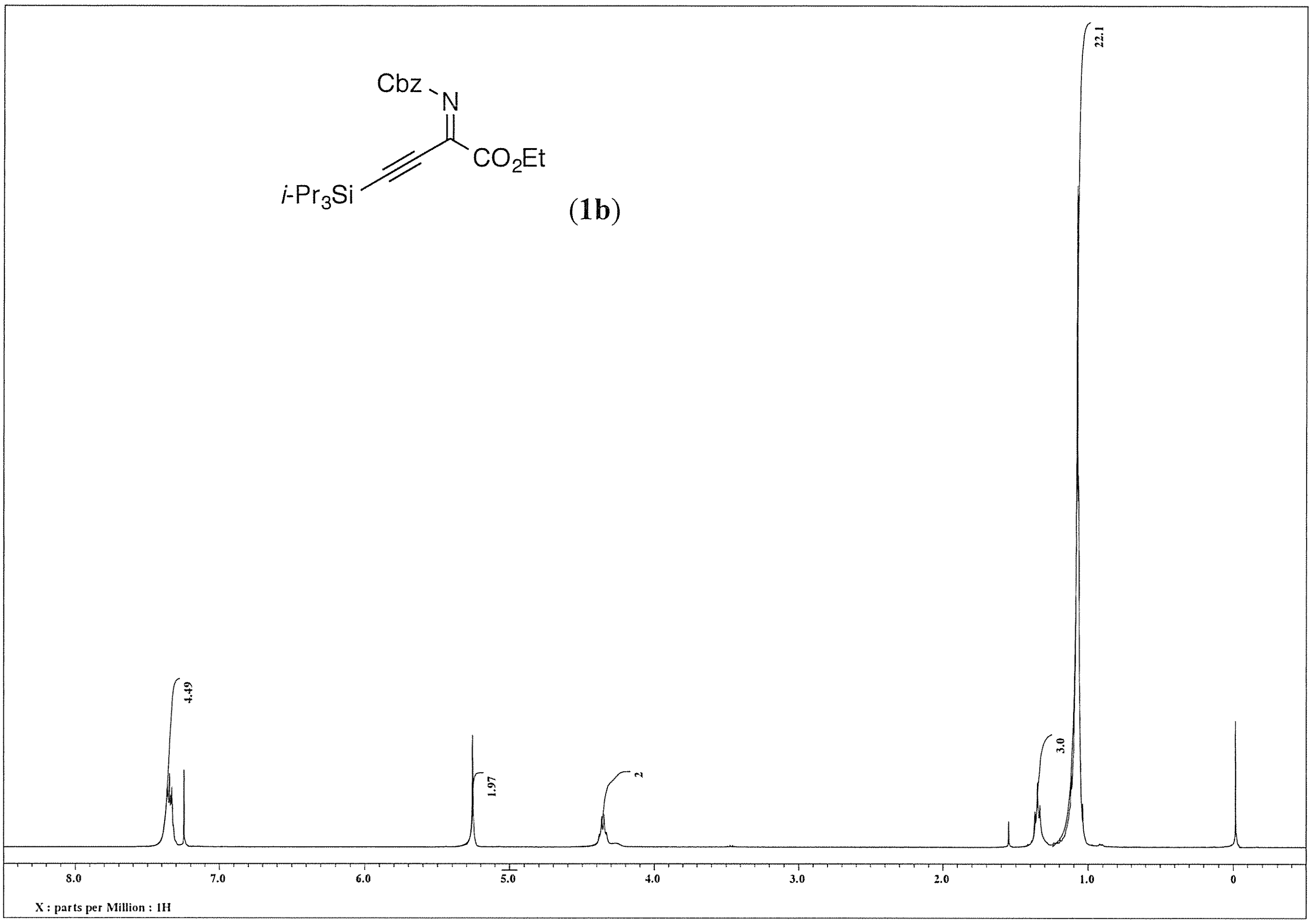

^{1}H NMR, 400 MHz, $CDCl_3$

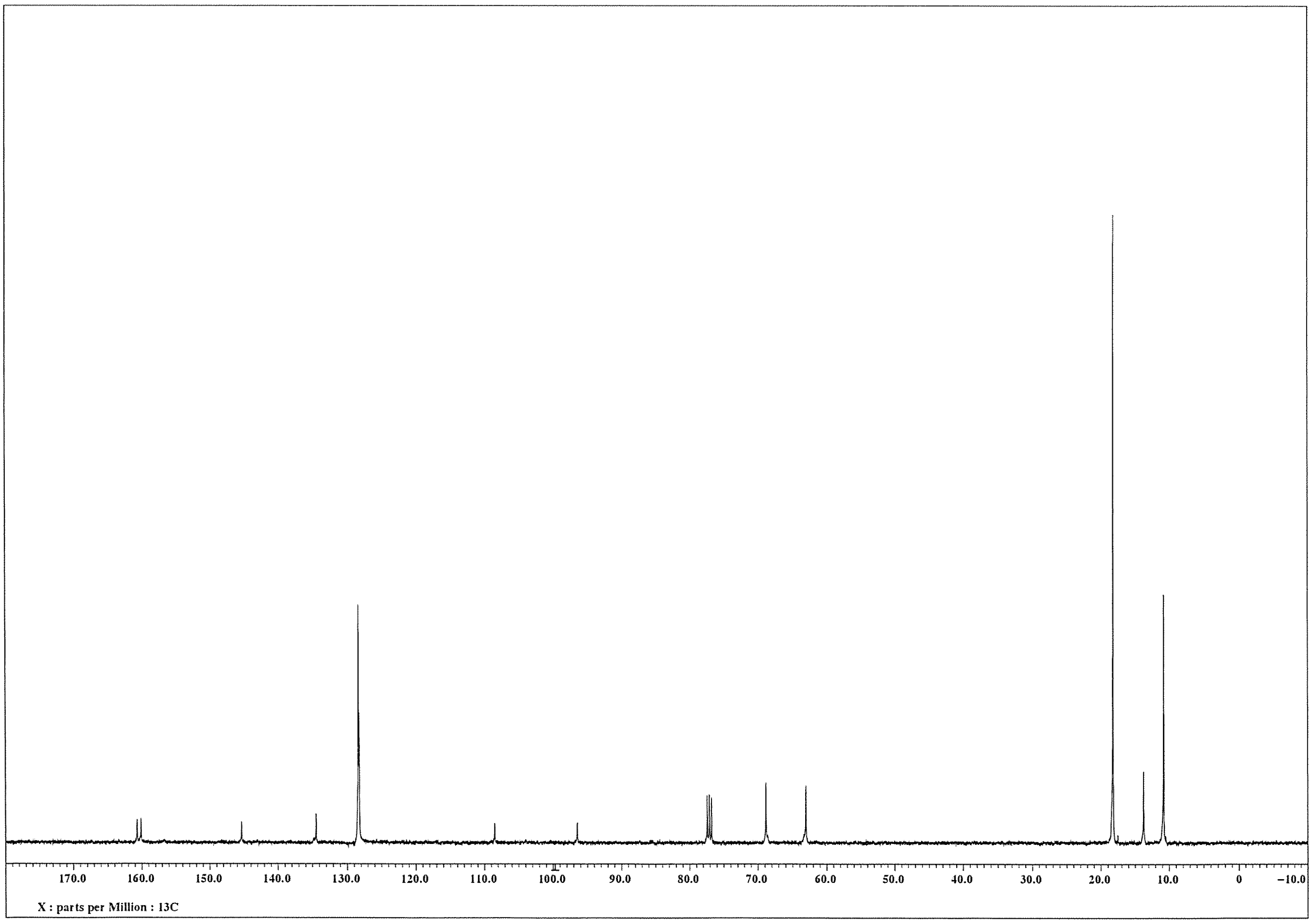

^{13}C NMR, 100 MHz, $CDCl_3$

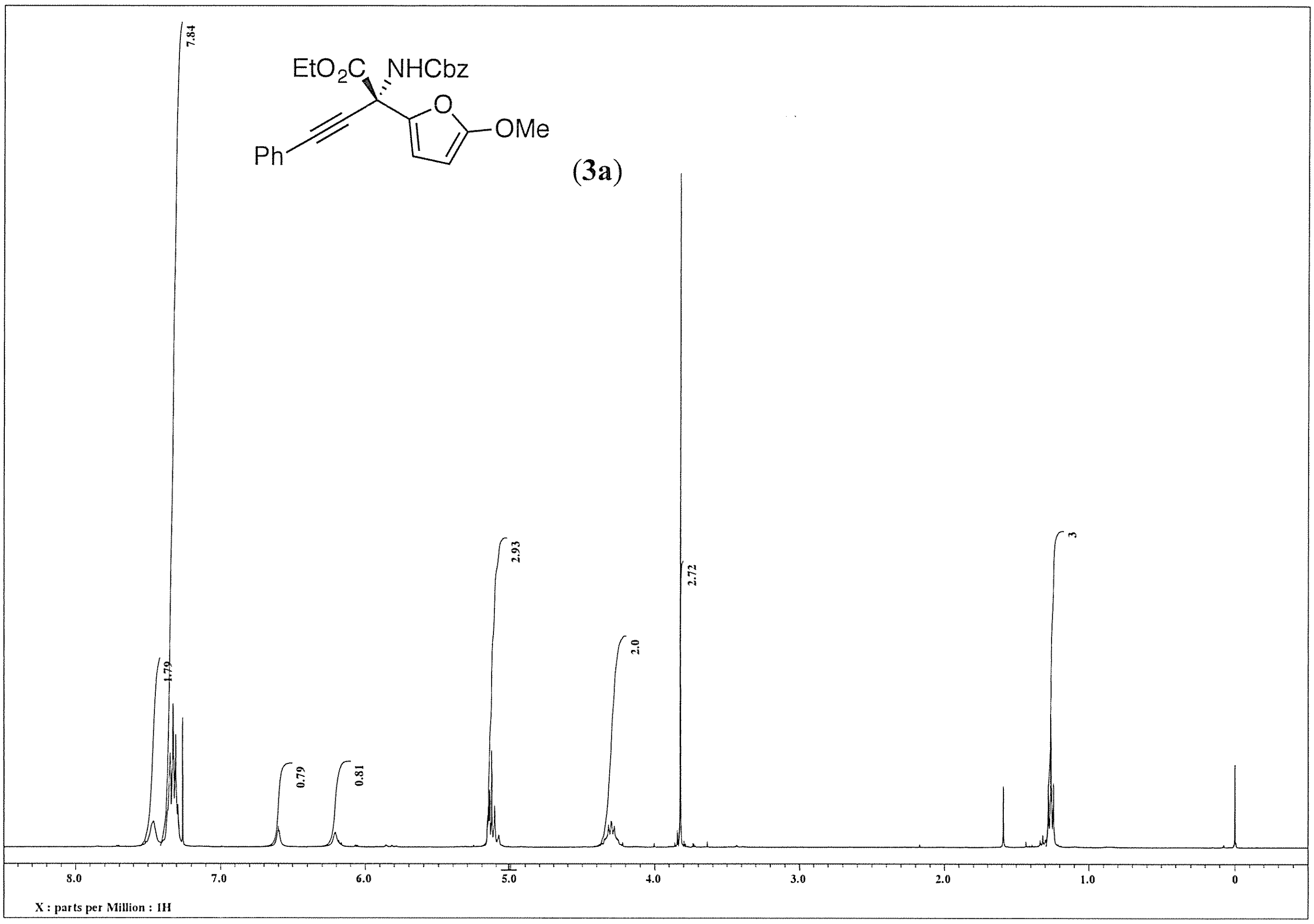

^{1}H NMR, 400 MHz, $CDCl_3$

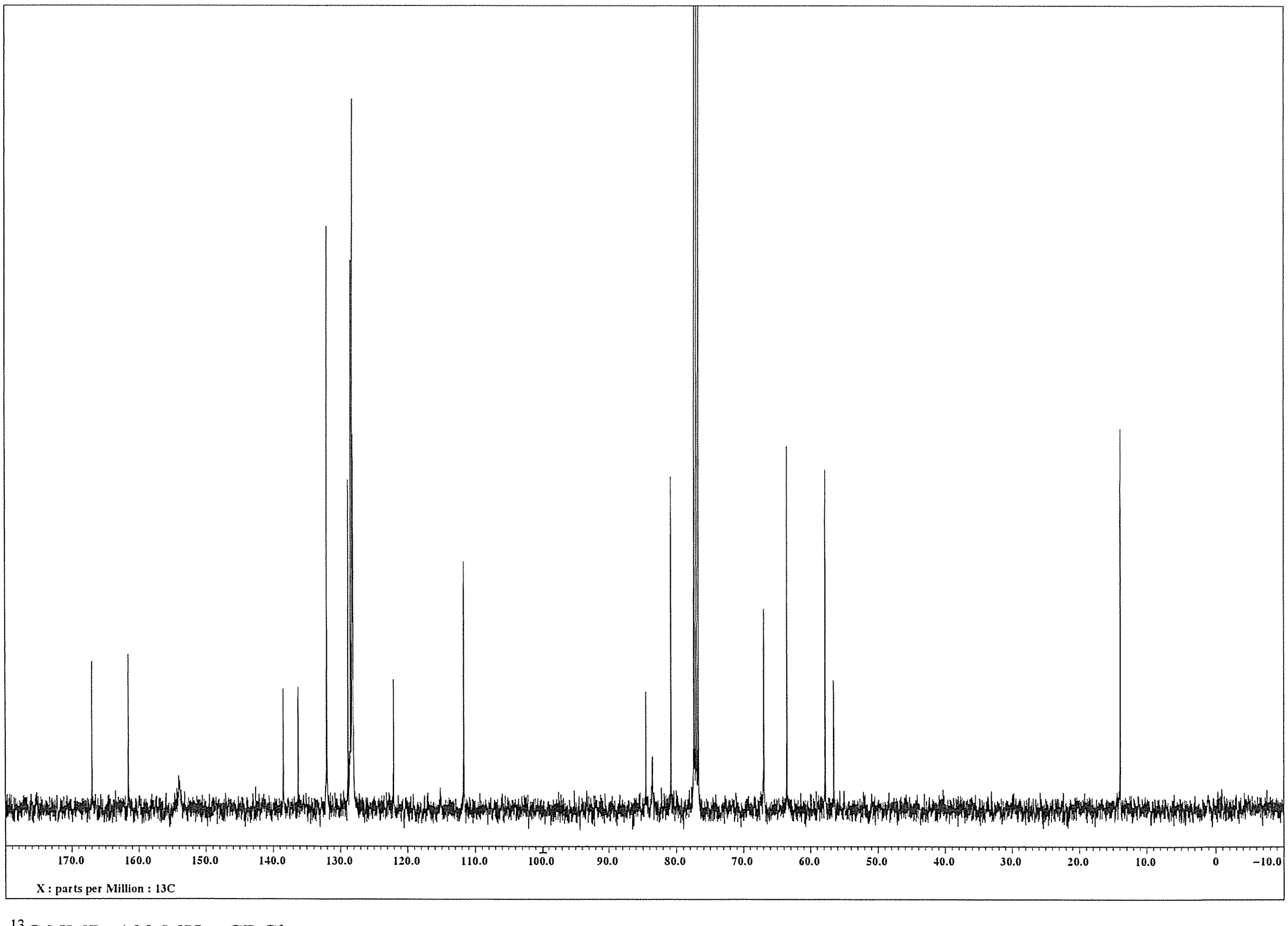

^{13}C NMR, 100 MHz, $CDCl_3$

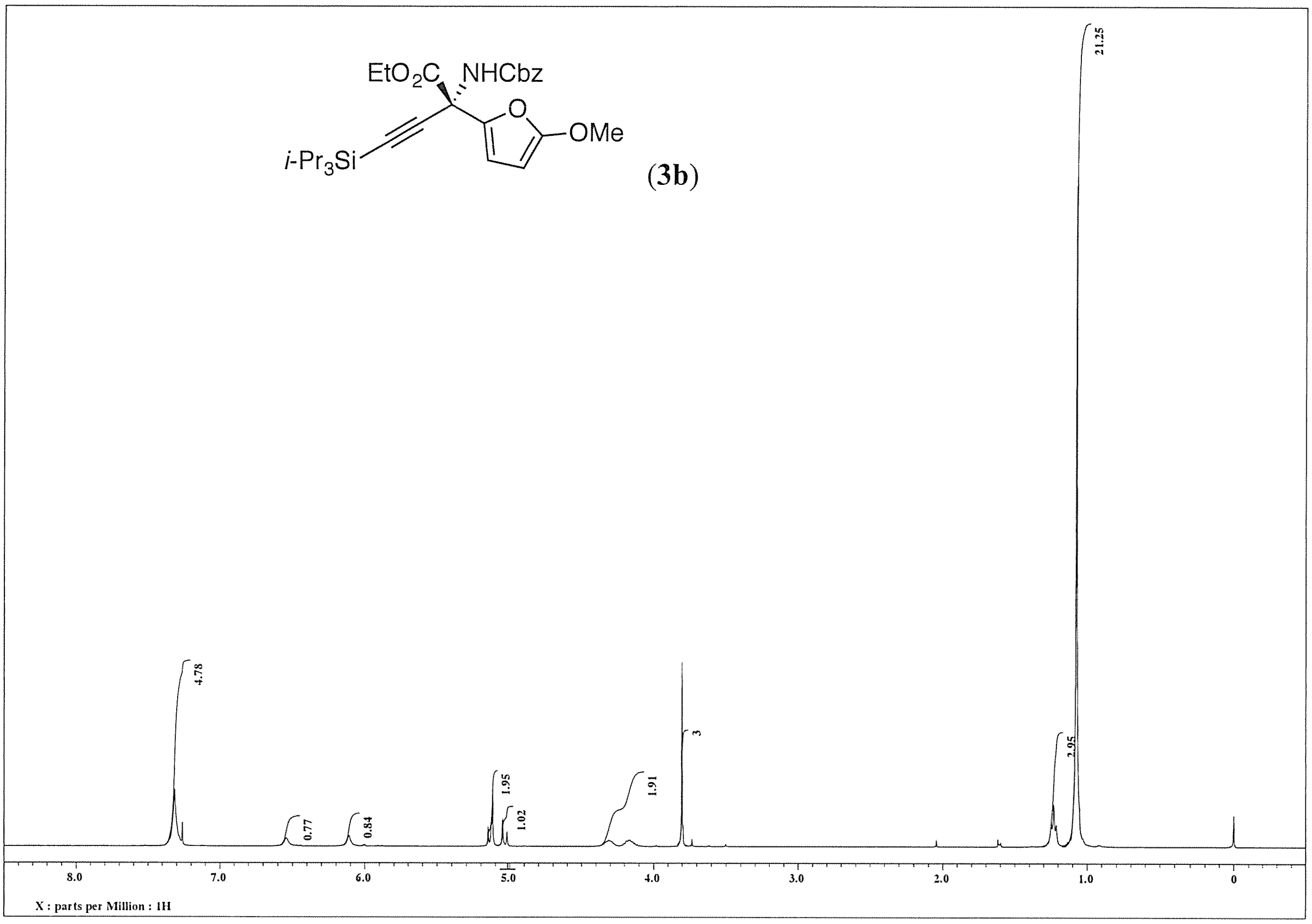

^{1}H NMR, 400 MHz, $CDCl_3$

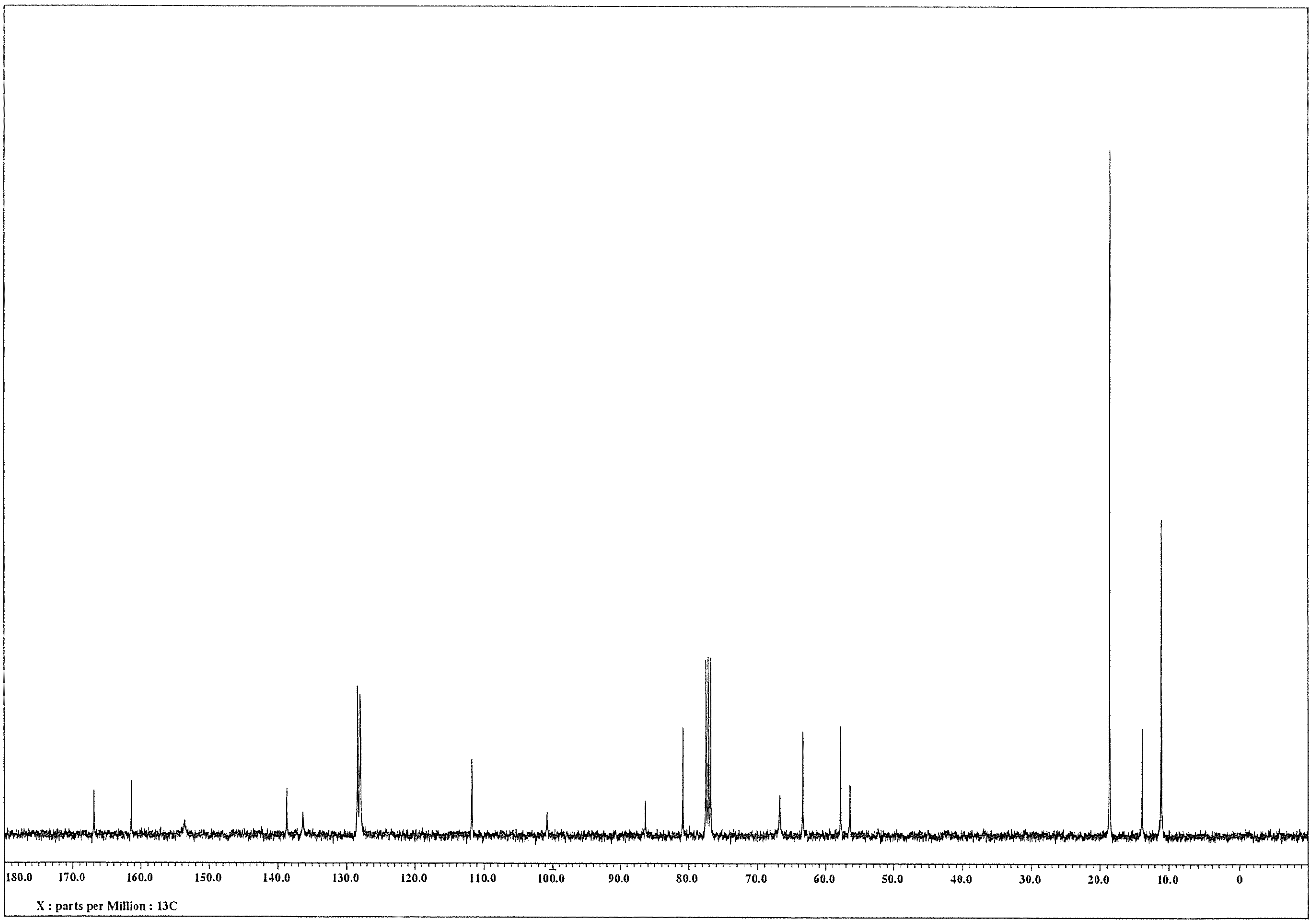

^{13}C NMR, 100 MHz, $CDCl_3$

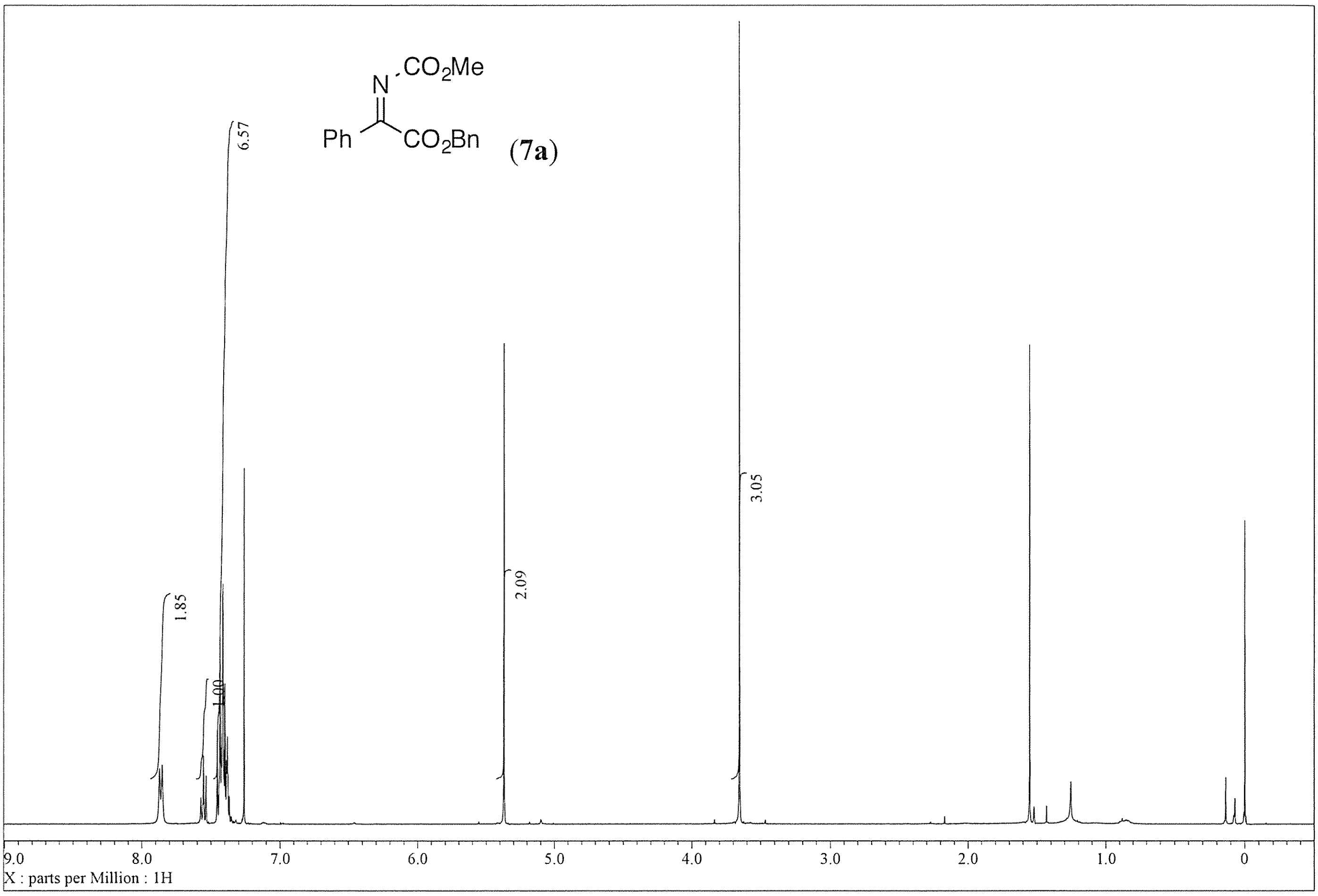

^{1}H NMR, 400 MHz, $CDCl_3$

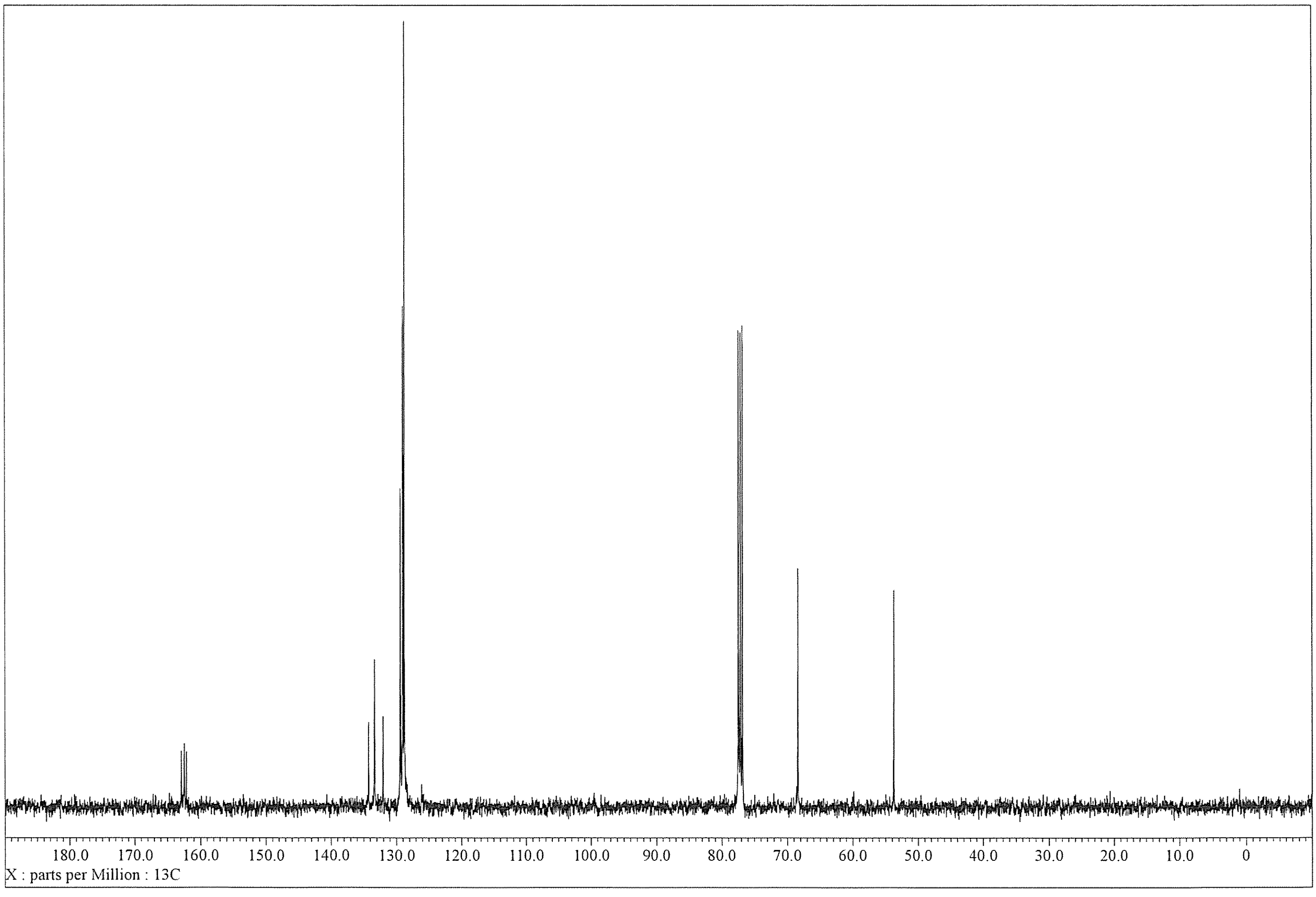

^{13}C NMR, 100 MHz, $CDCl_3$

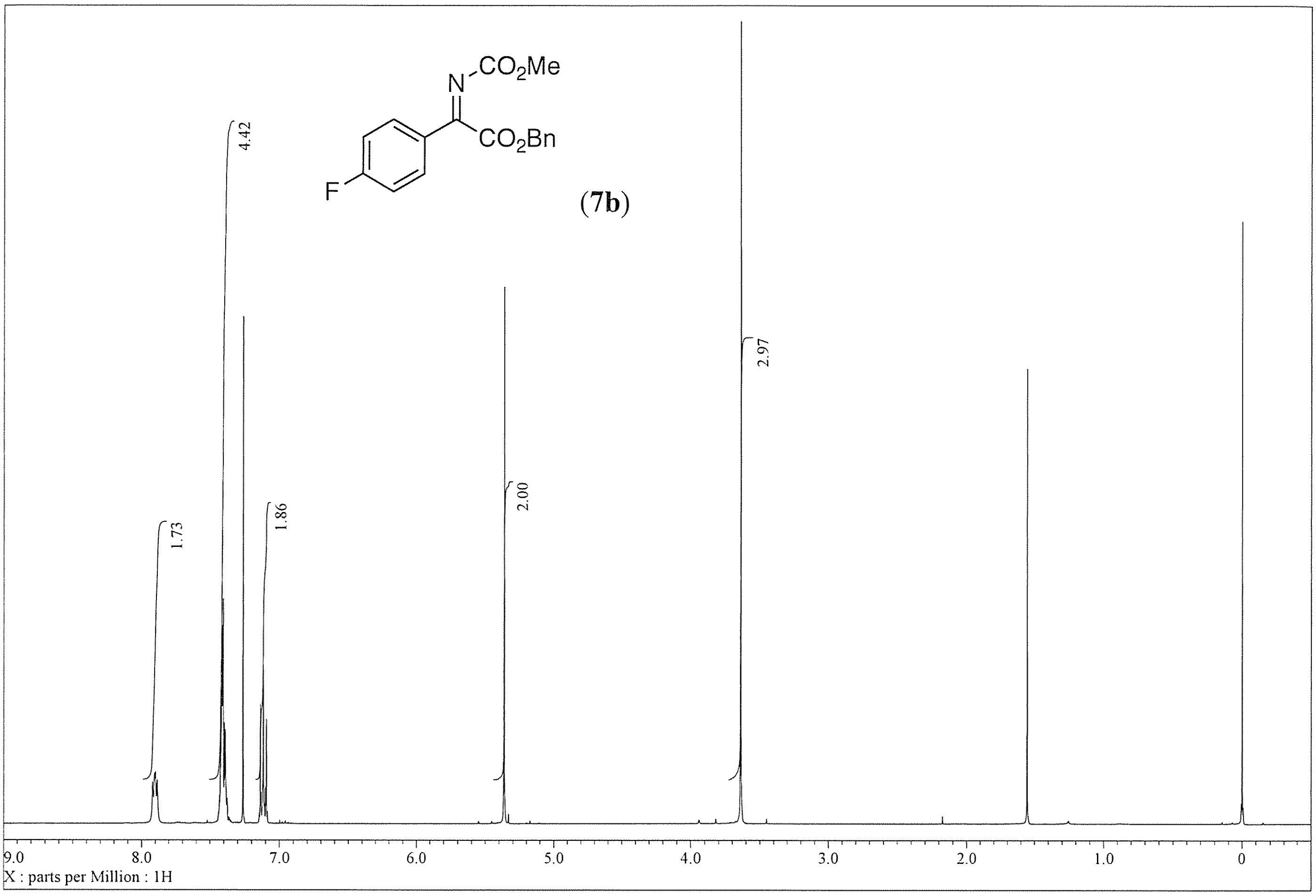

^{1}H NMR, 400 MHz, $CDCl_3$

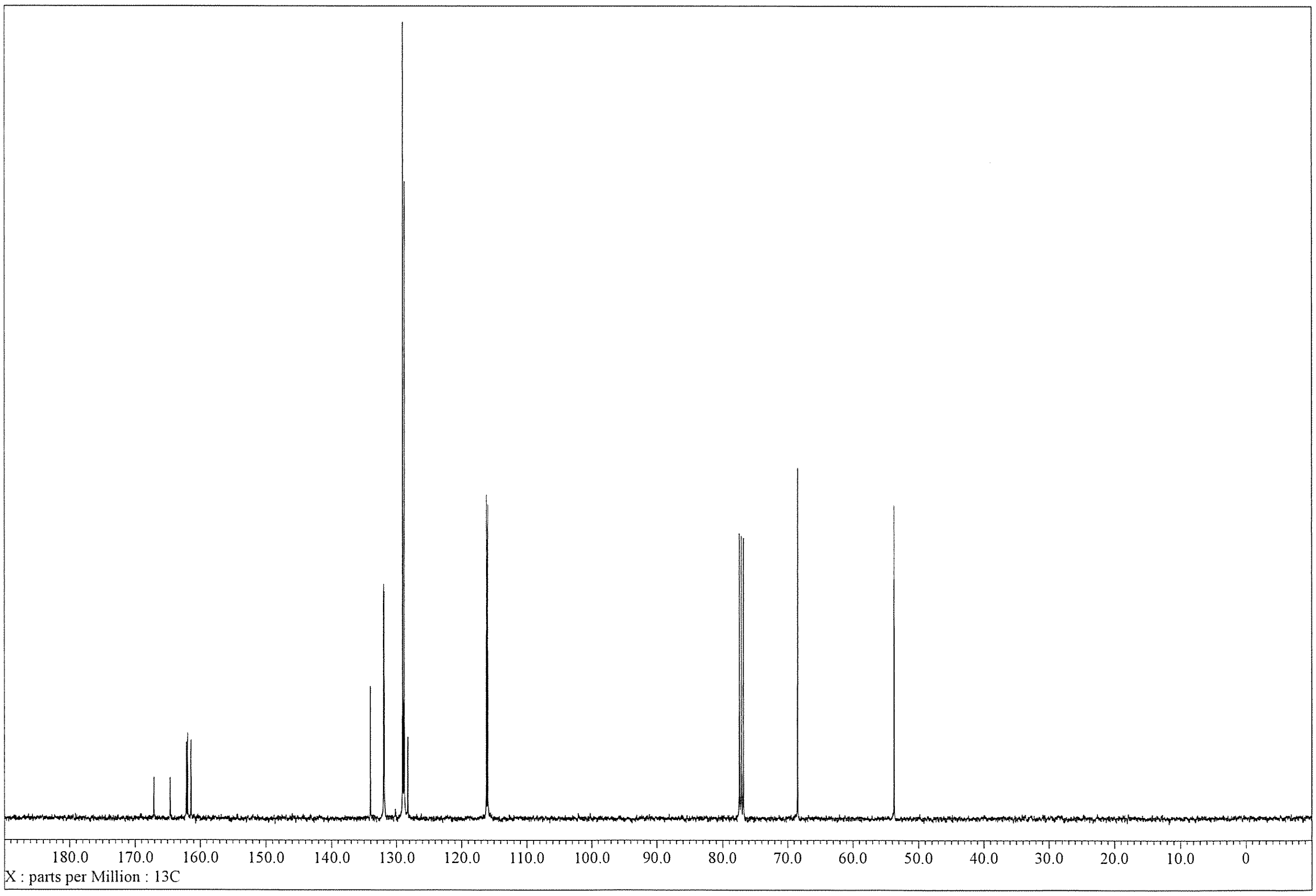

^{13}C NMR, 100 MHz, $CDCl_3$

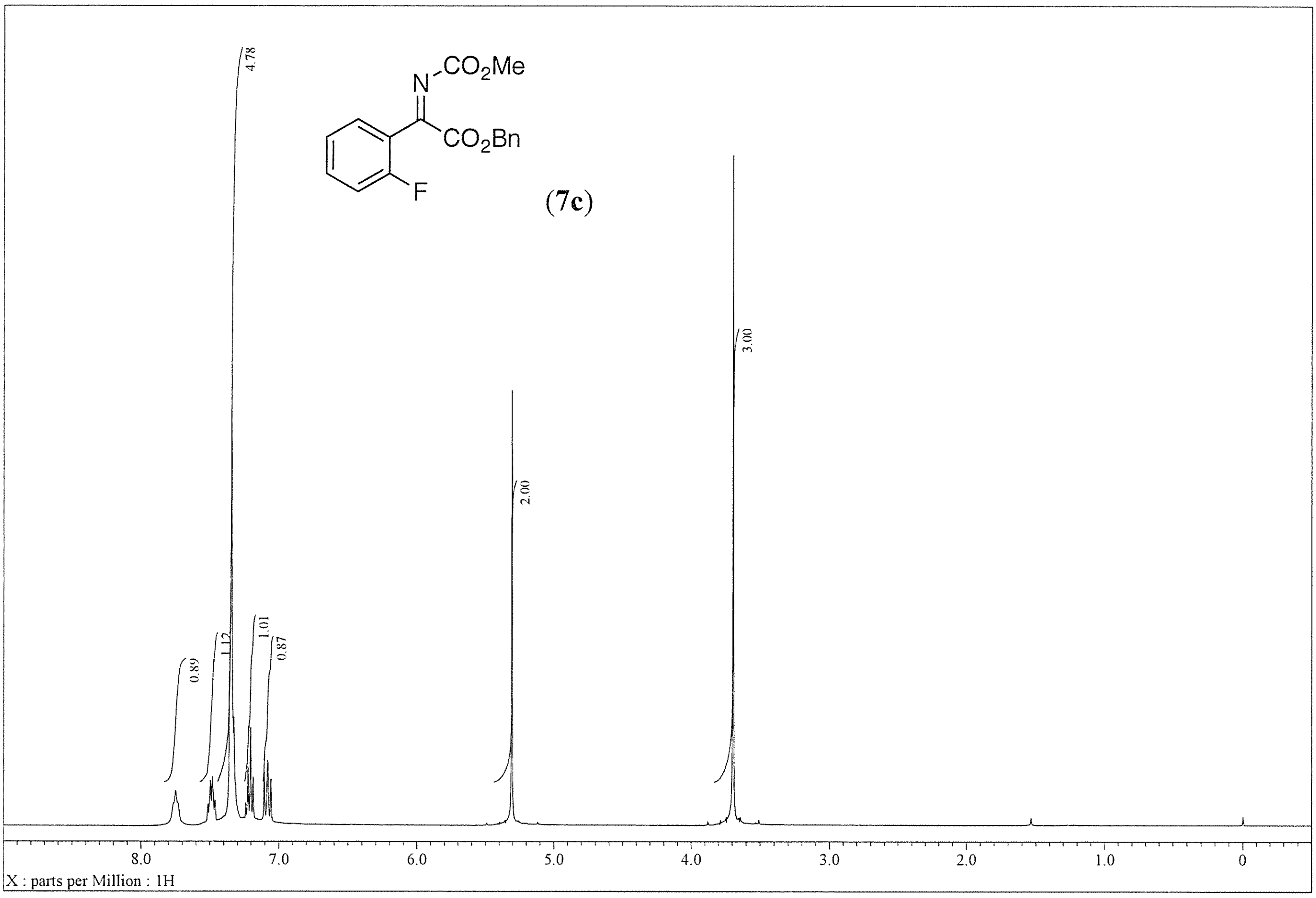

^{1}H NMR, 400 MHz, $CDCl_3$

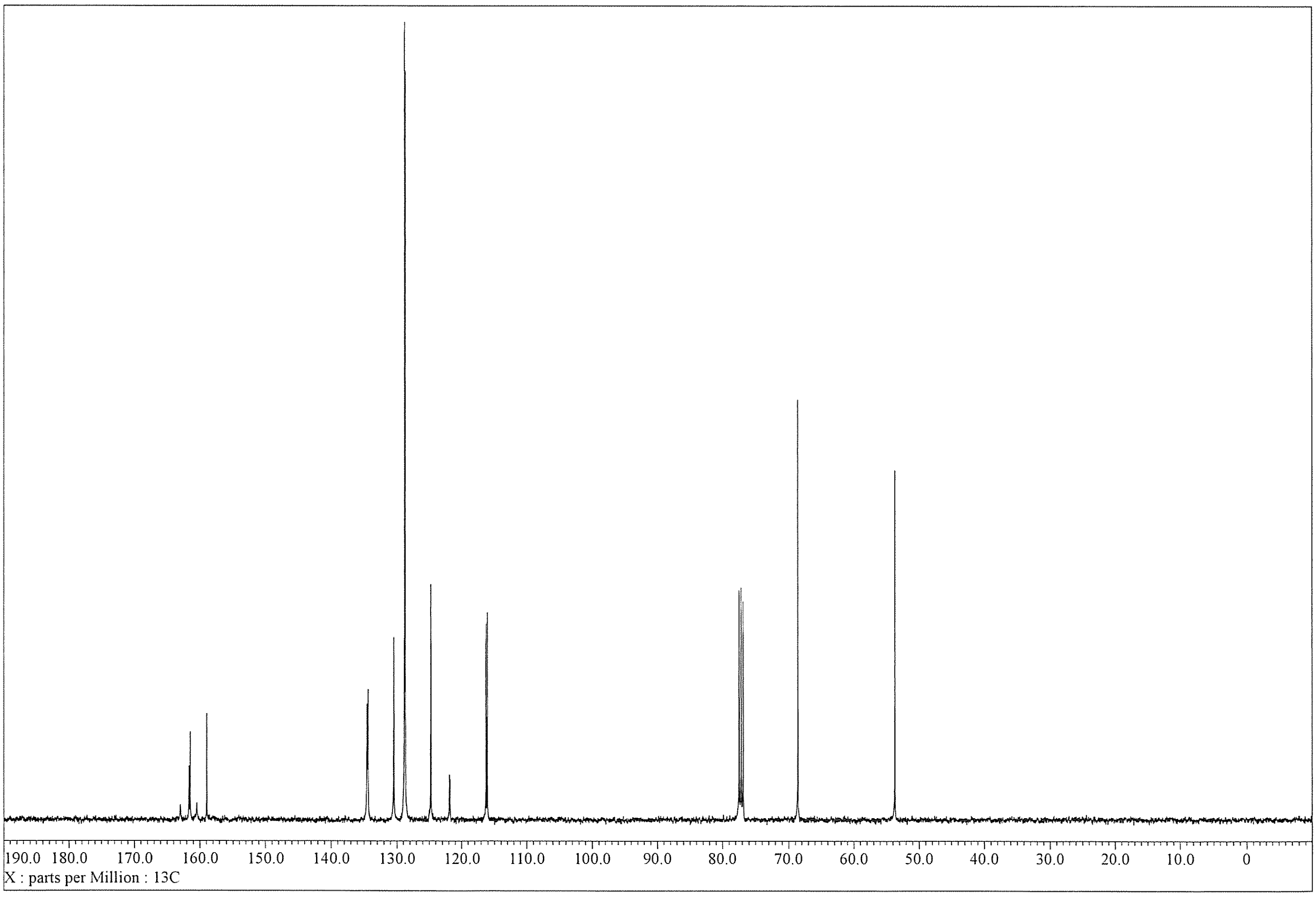

^{13}C NMR, 100 MHz, $CDCl_3$

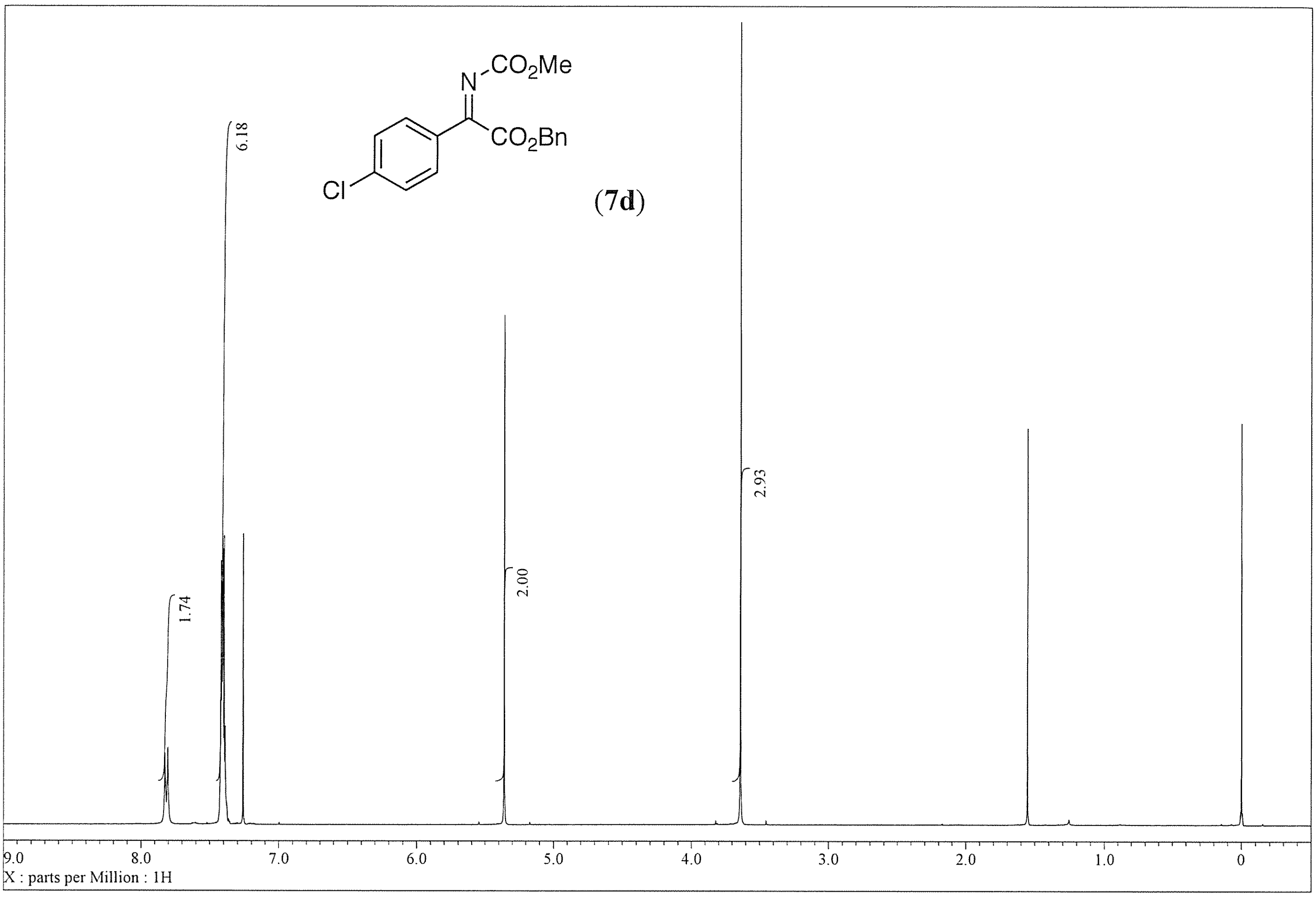

^{1}H NMR, 400 MHz, $CDCl_3$

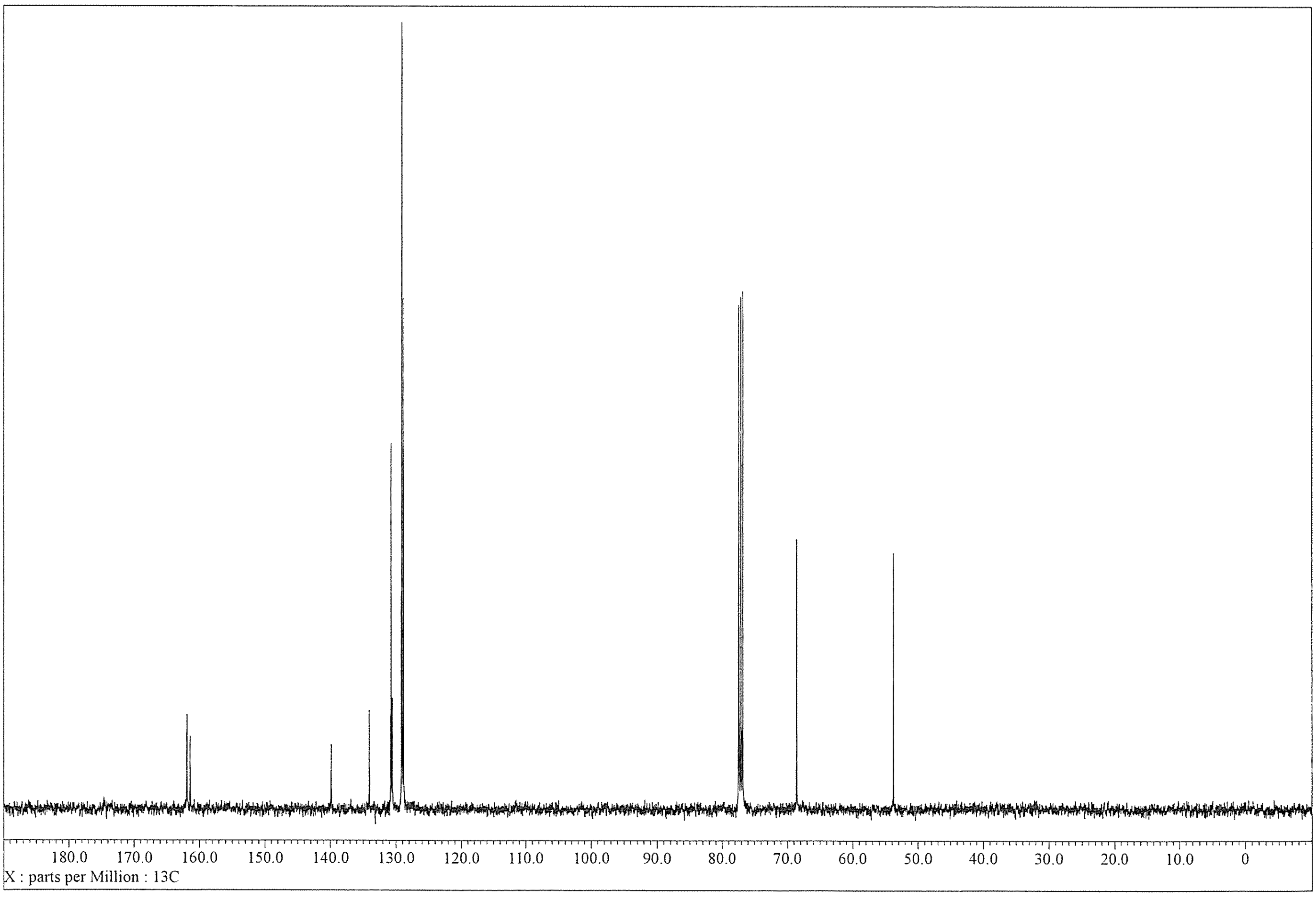

^{13}C NMR, 100 MHz, $CDCl_3$

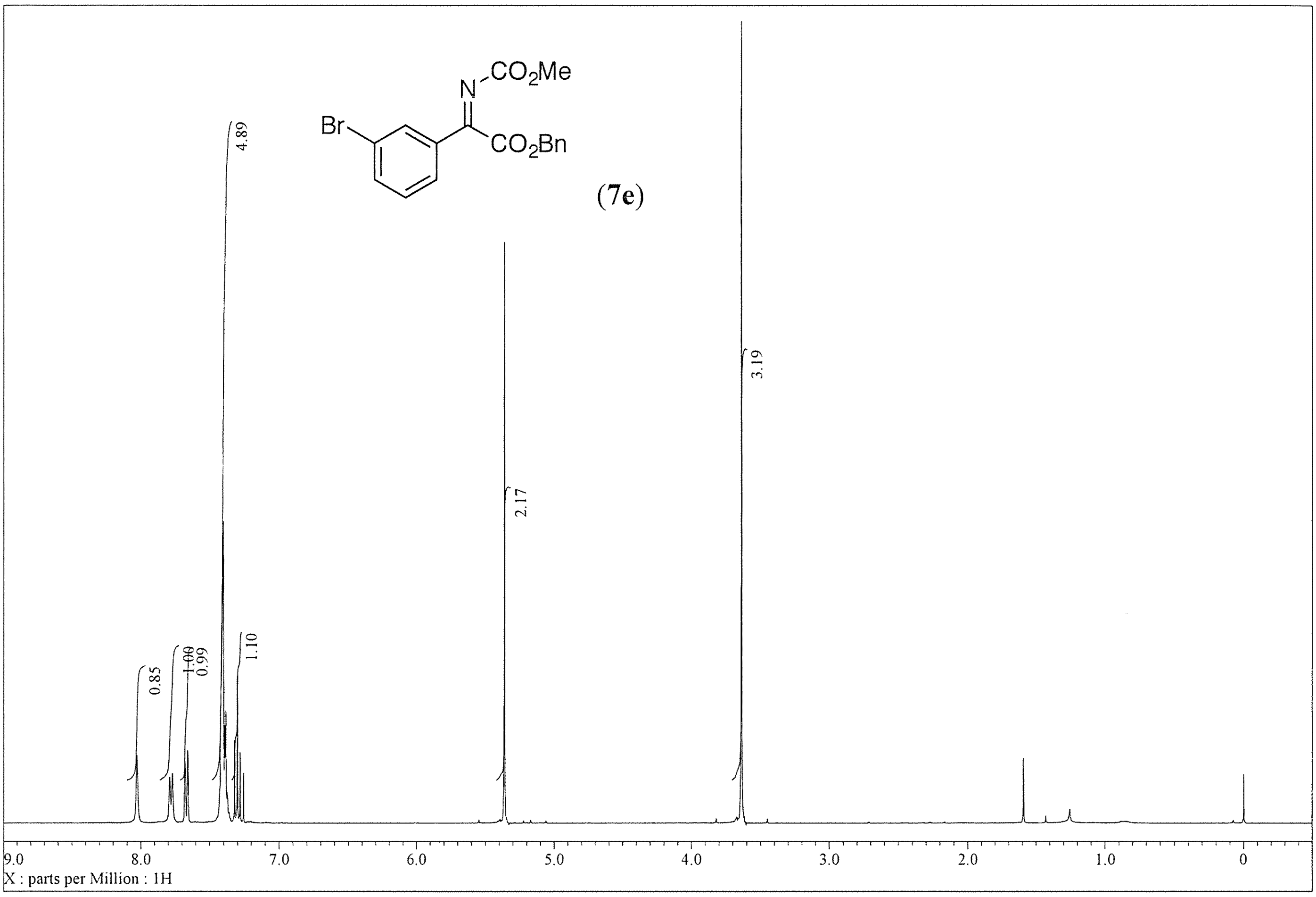

^{1}H NMR, 400 MHz, $CDCl_3$

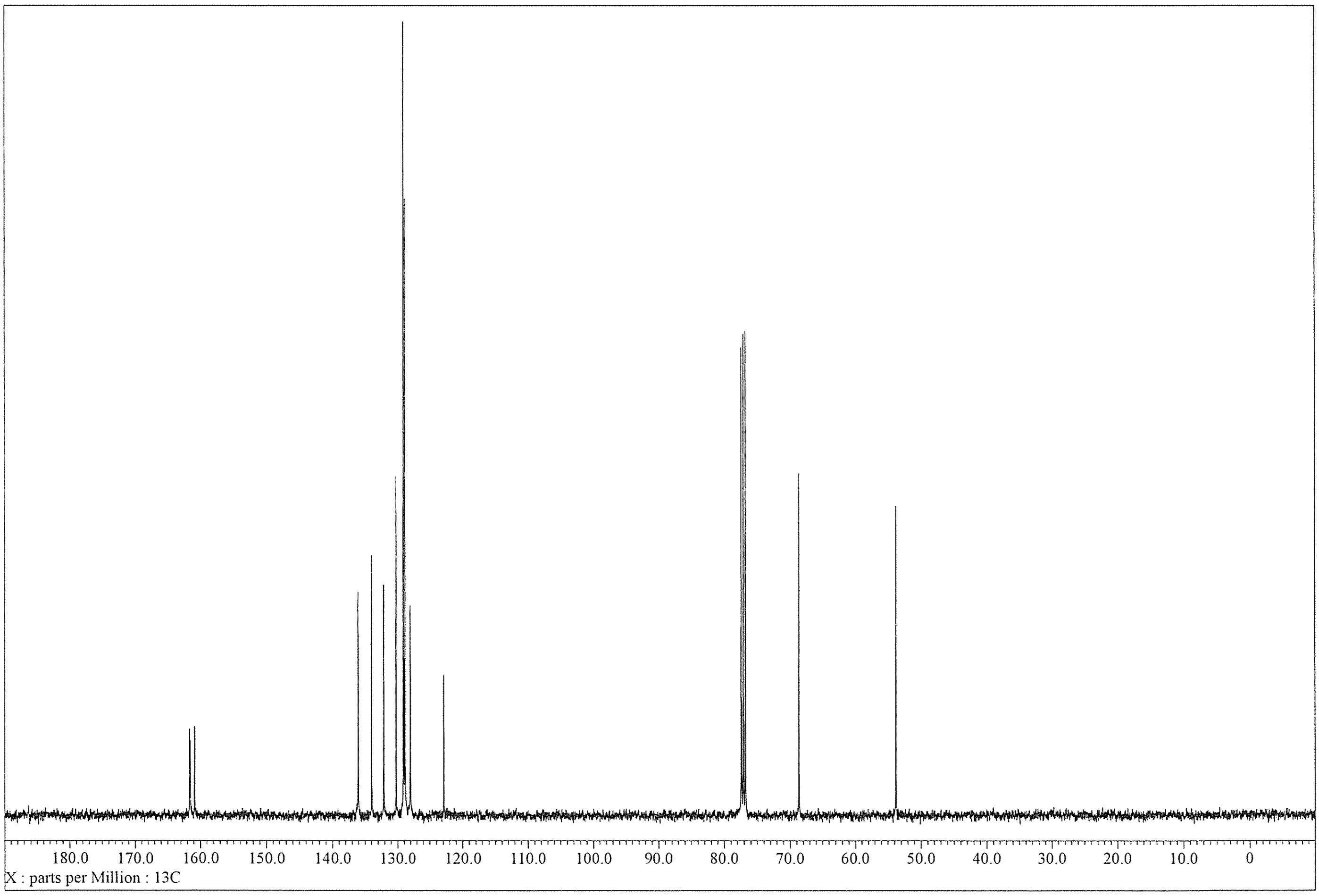

^{13}C NMR, 100 MHz, $CDCl_3$

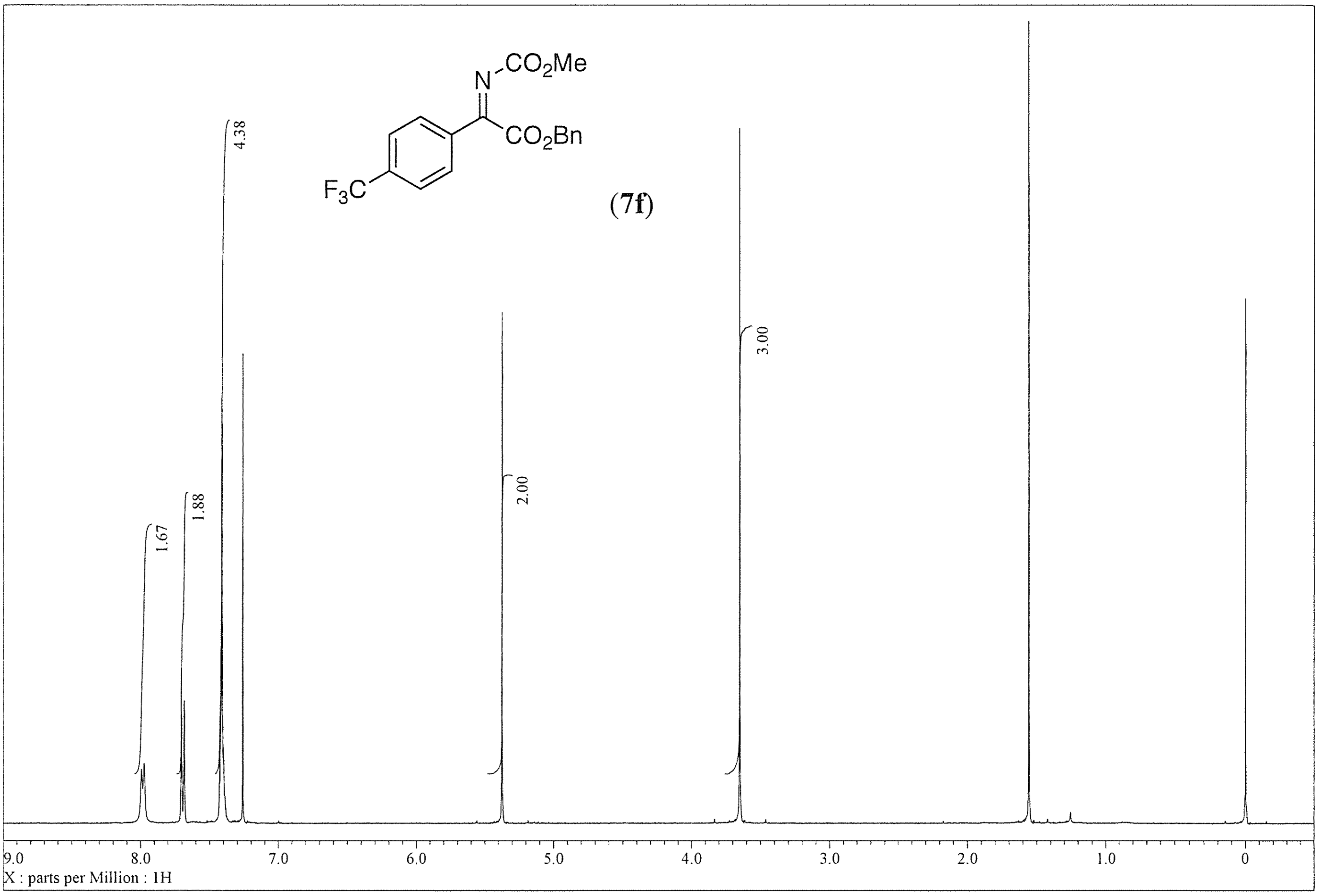

^{1}H NMR, 400 MHz, $CDCl_3$

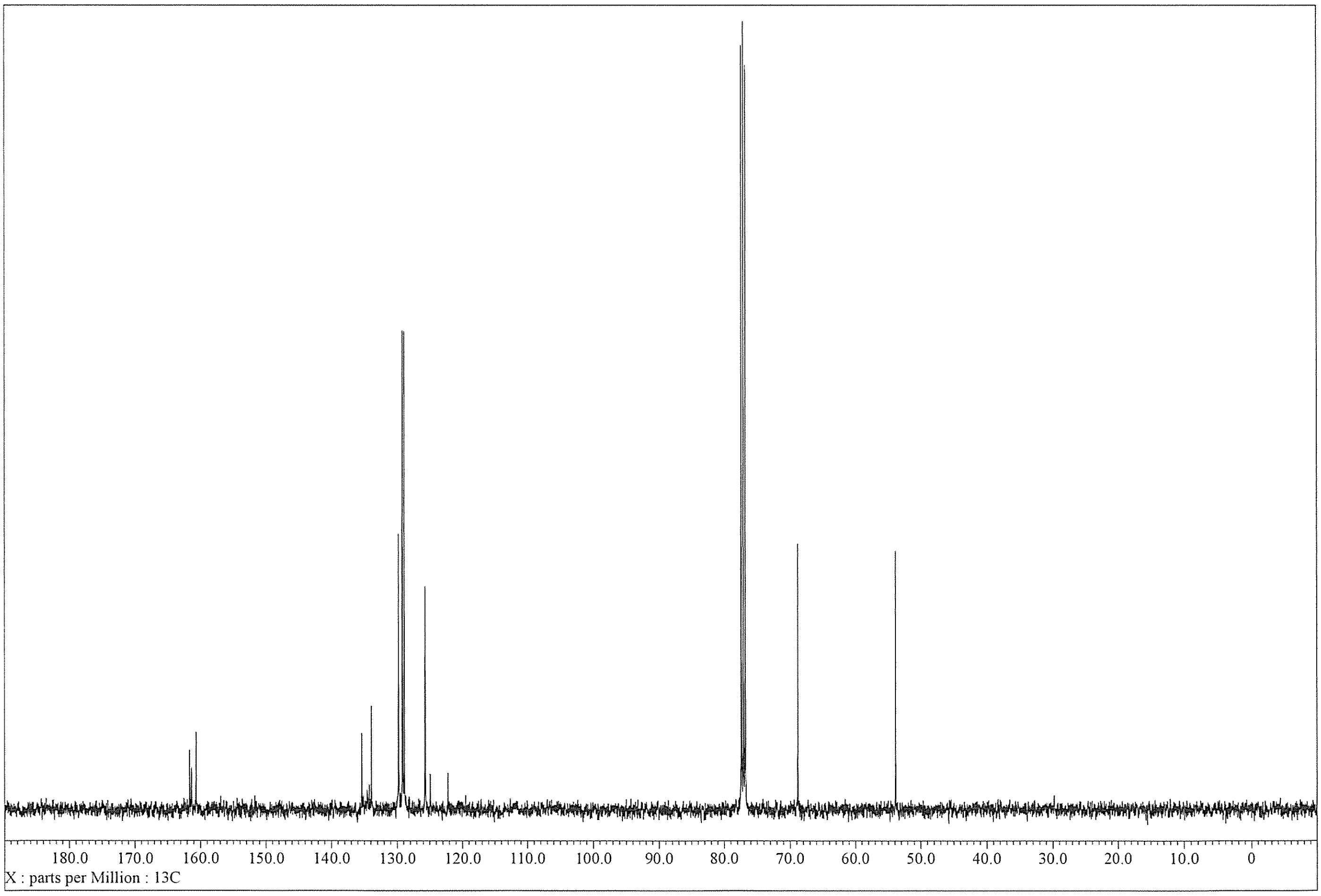

^{13}C NMR, 100 MHz, $CDCl_3$

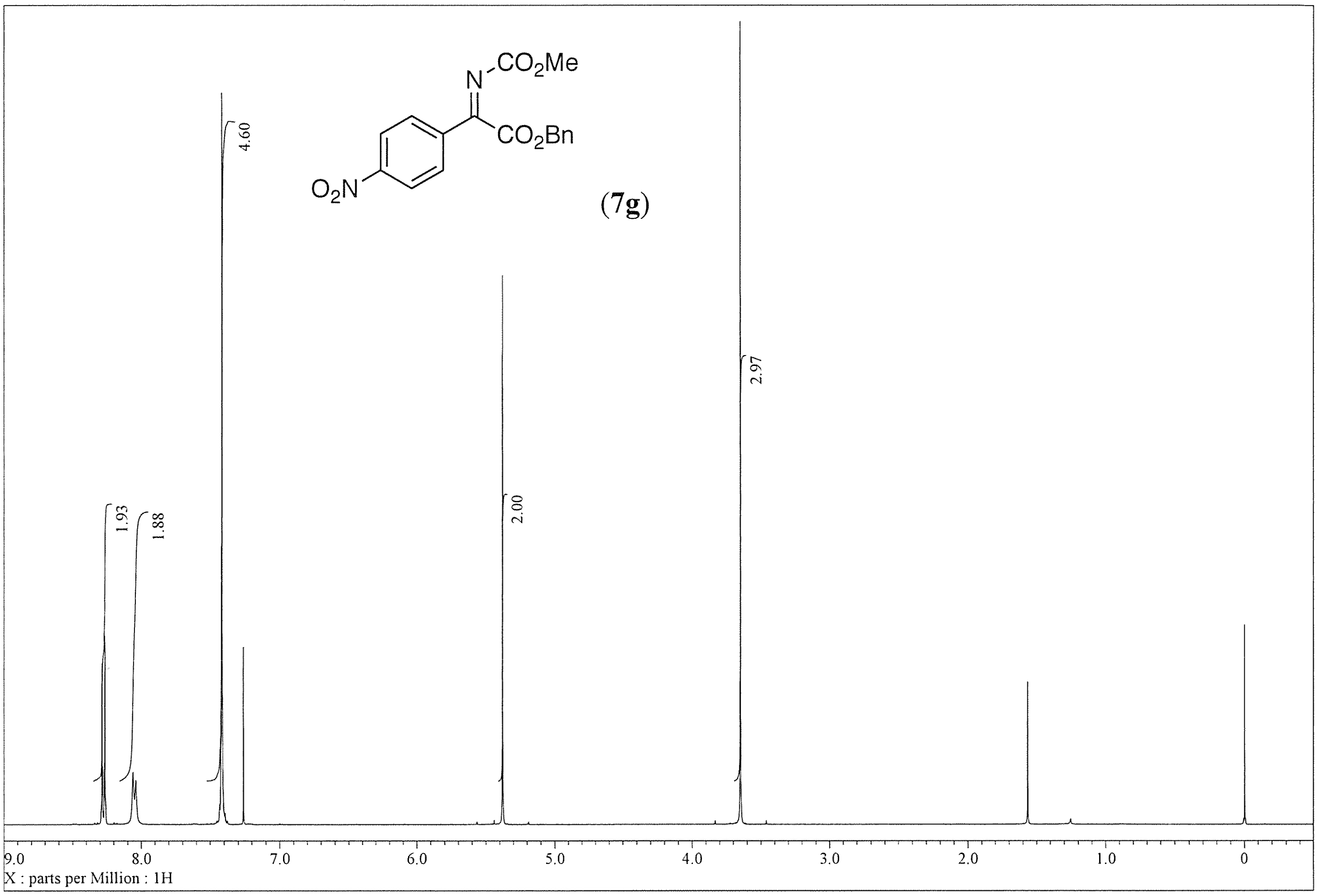

^{1}H NMR, 400 MHz, $CDCl_3$

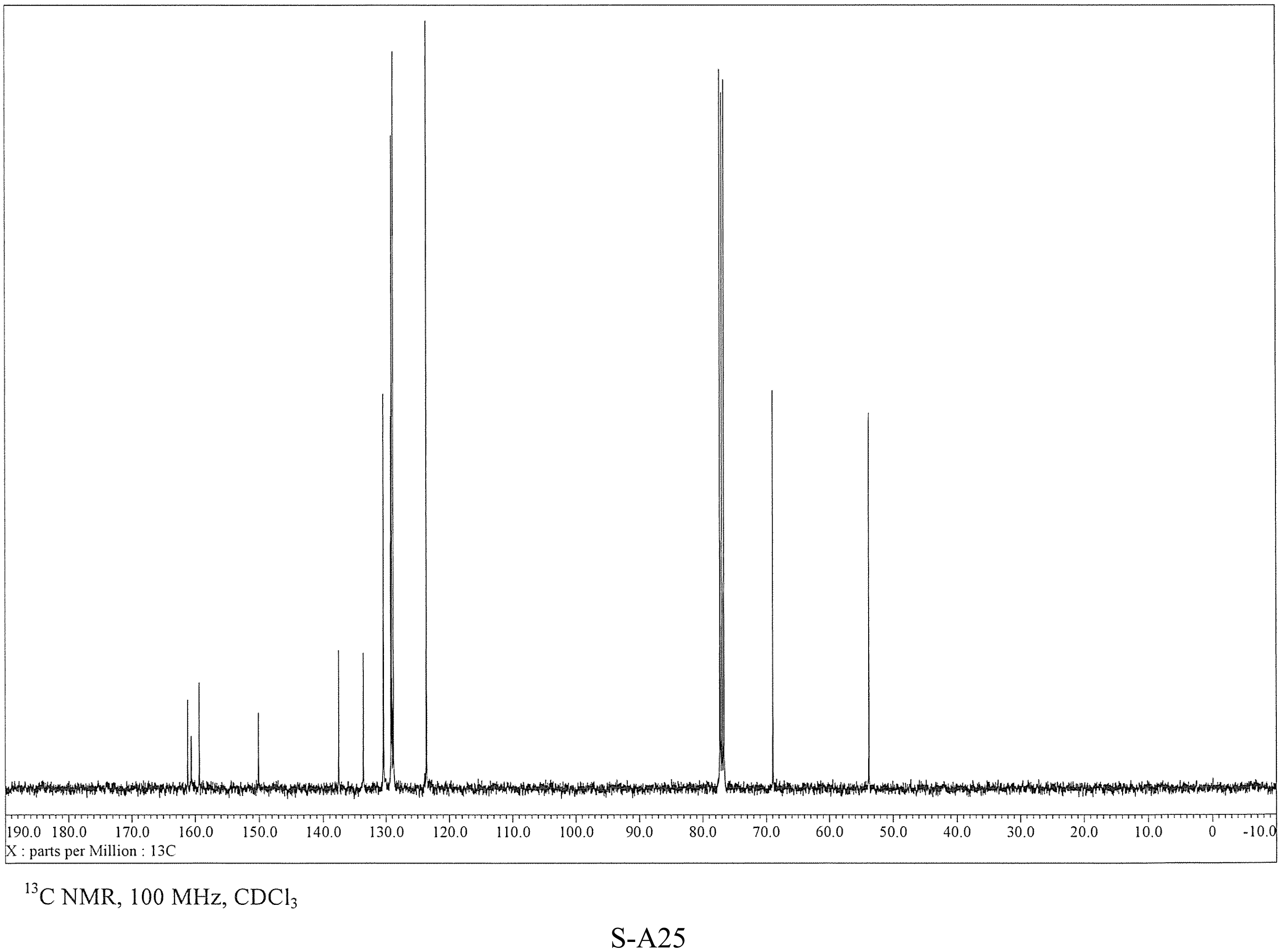

^{13}C NMR, 100 MHz, $CDCl_3$

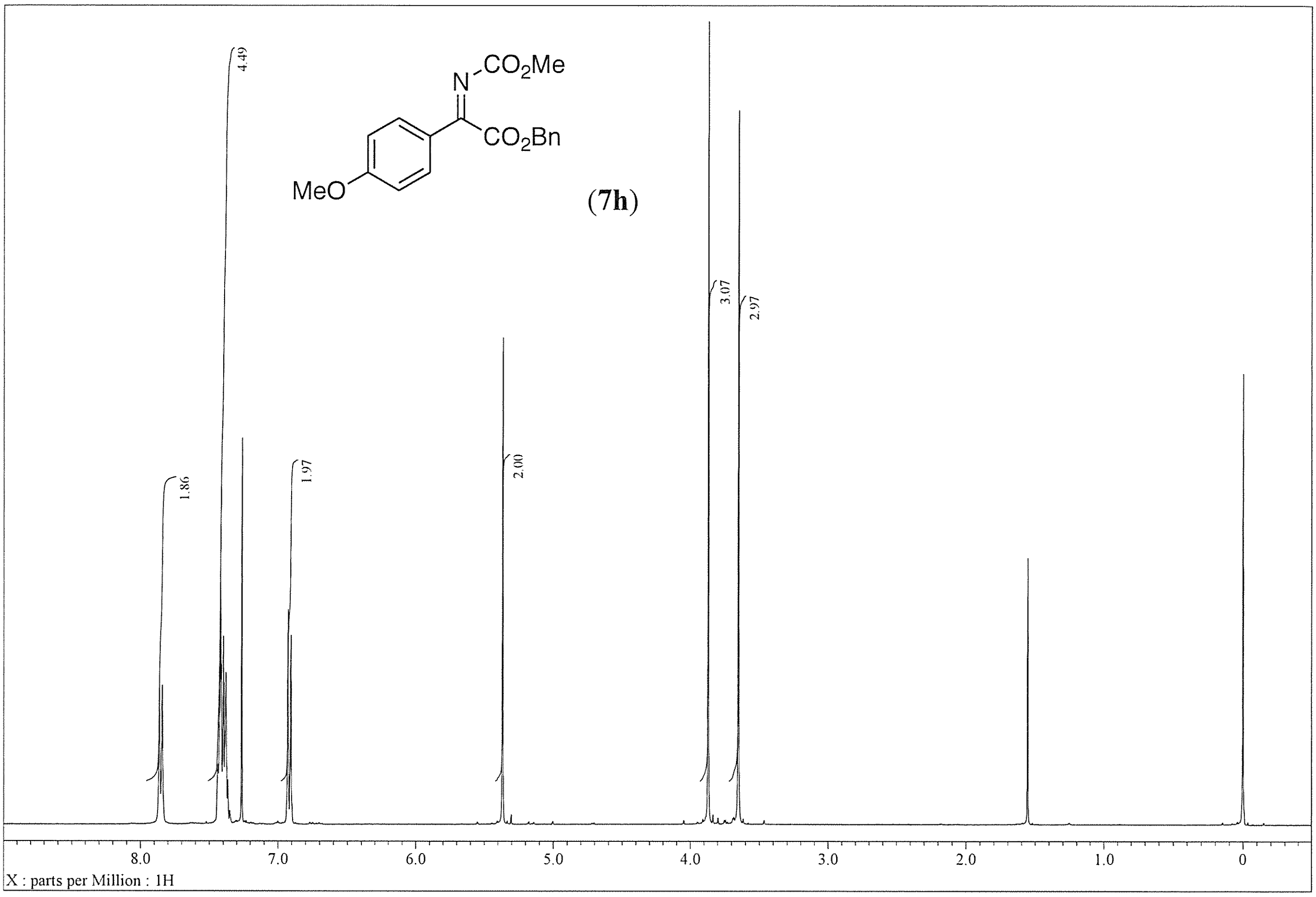

^{1}H NMR, 400 MHz, $CDCl_3$

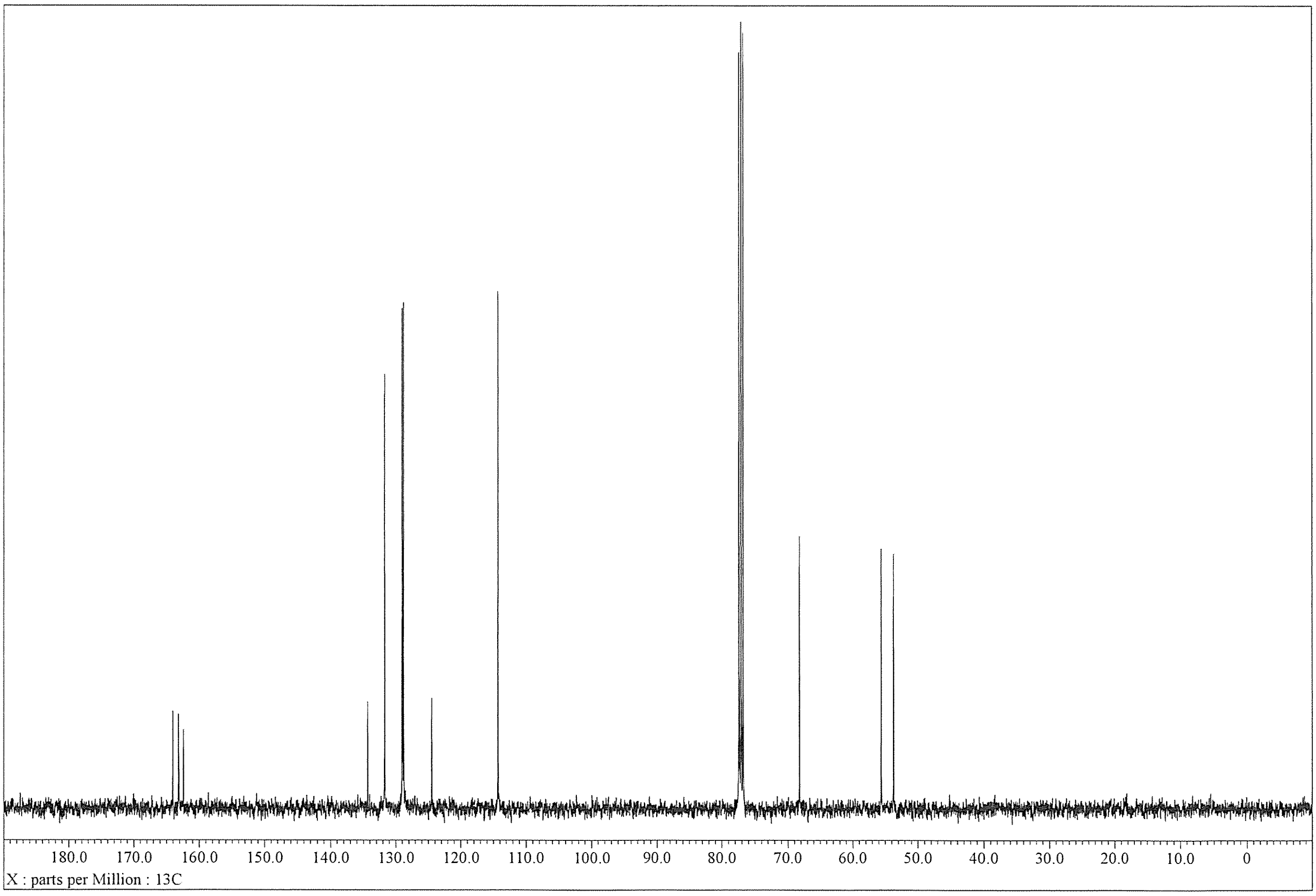

^{13}C NMR, 100 MHz, $CDCl_3$

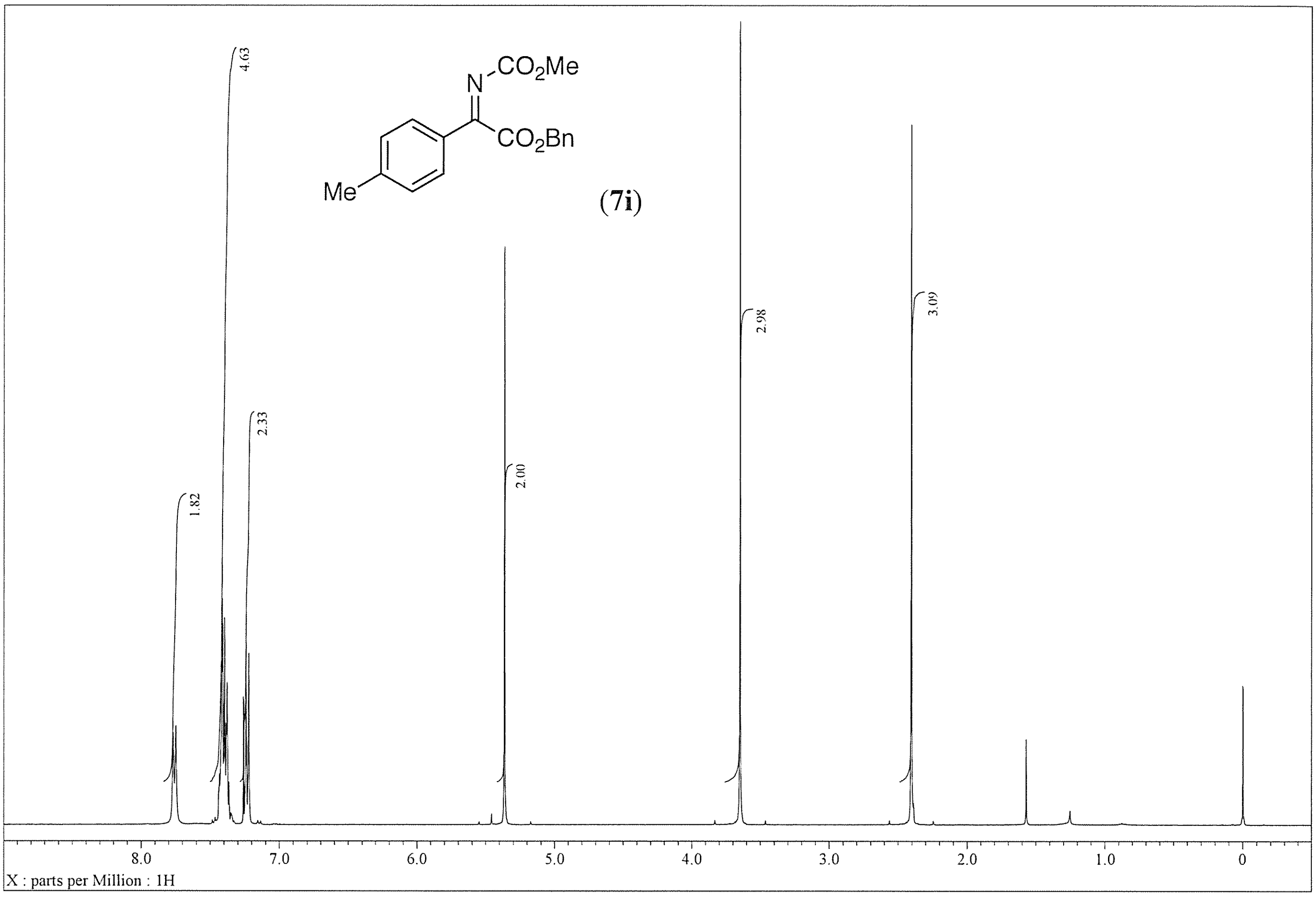

^{1}H NMR, 400 MHz, CDCl$_3$

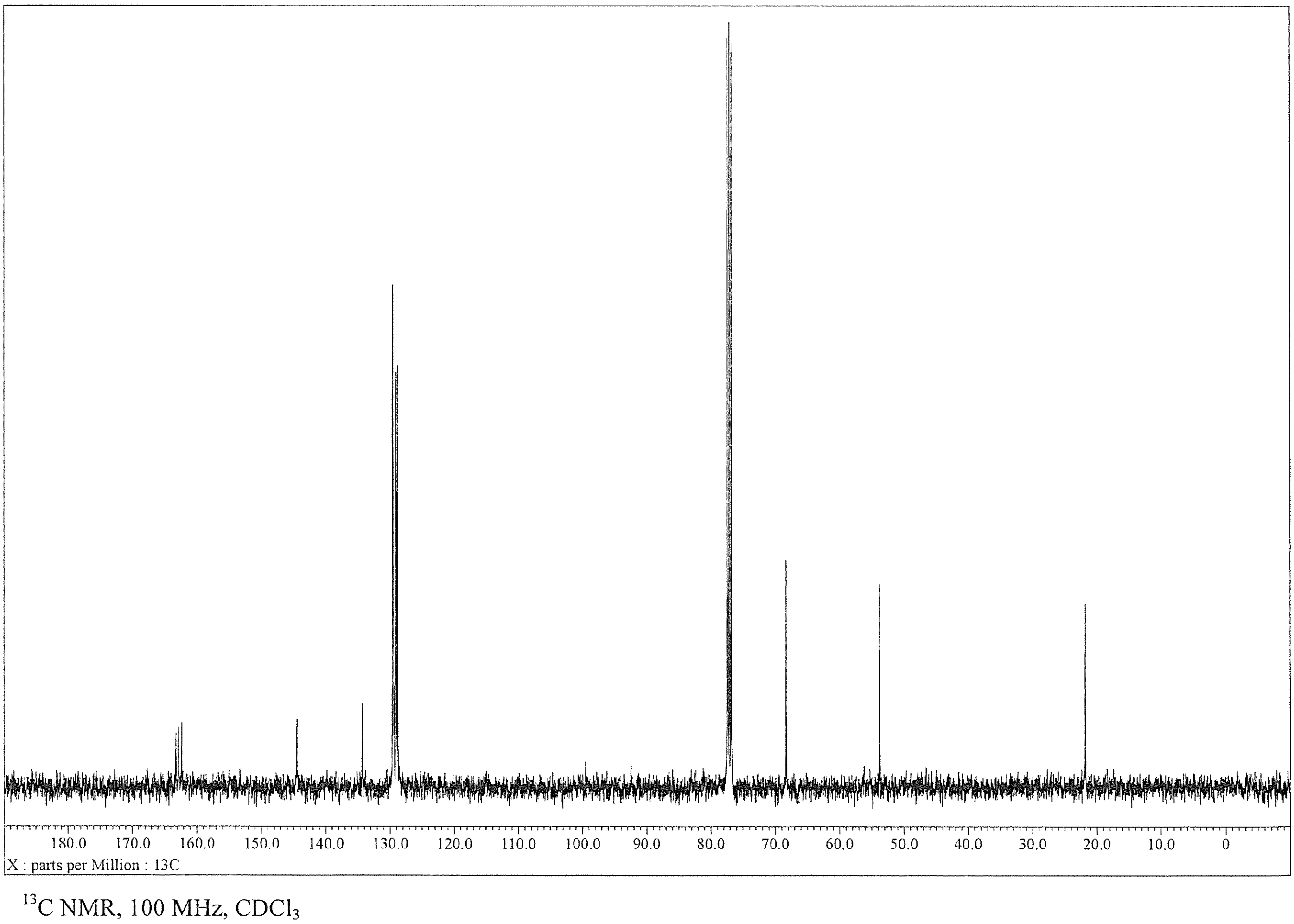

^{13}C NMR, 100 MHz, CDCl$_3$

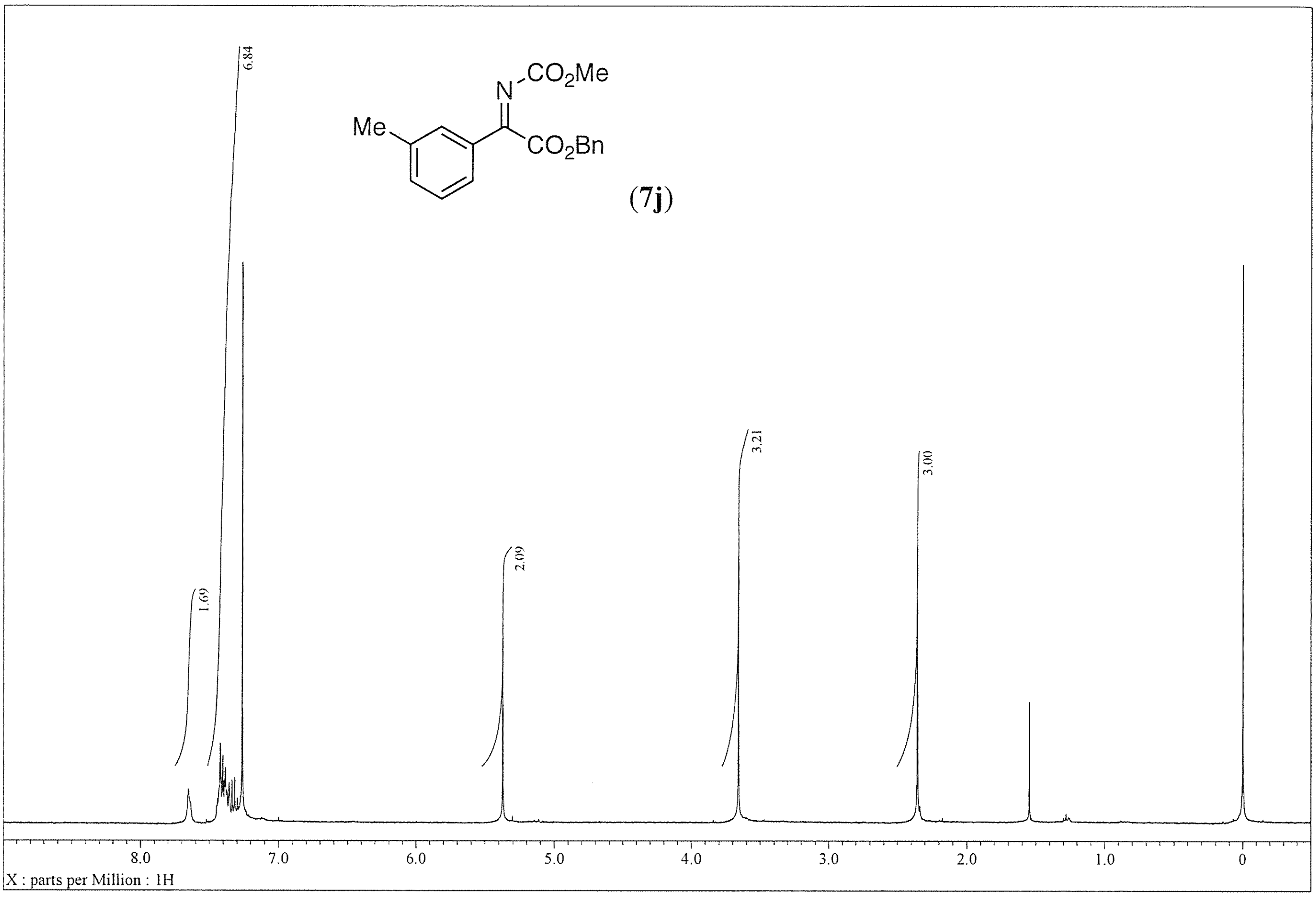

^{1}H NMR, 400 MHz, $CDCl_3$

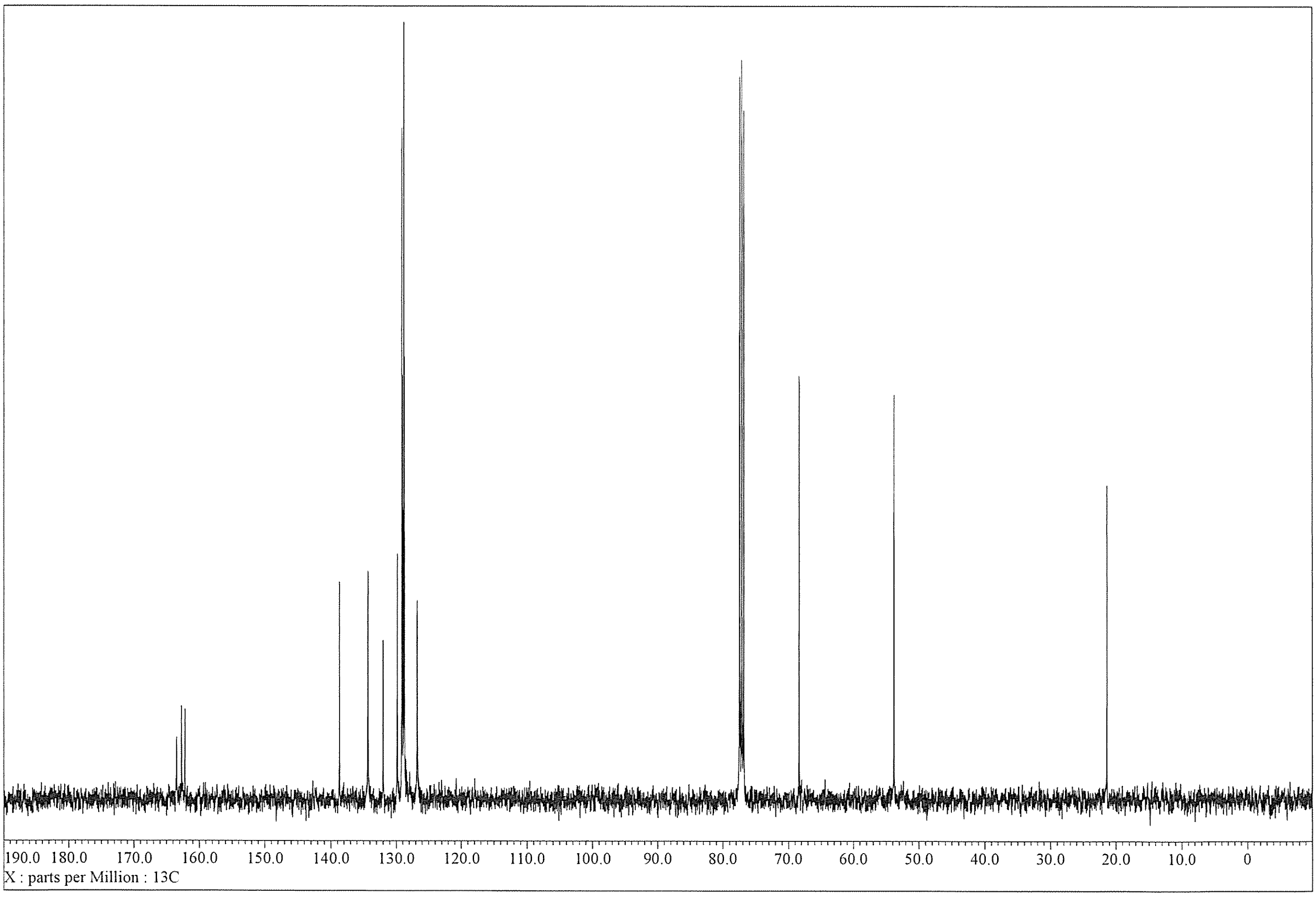

^{13}C NMR, 100 MHz, $CDCl_3$

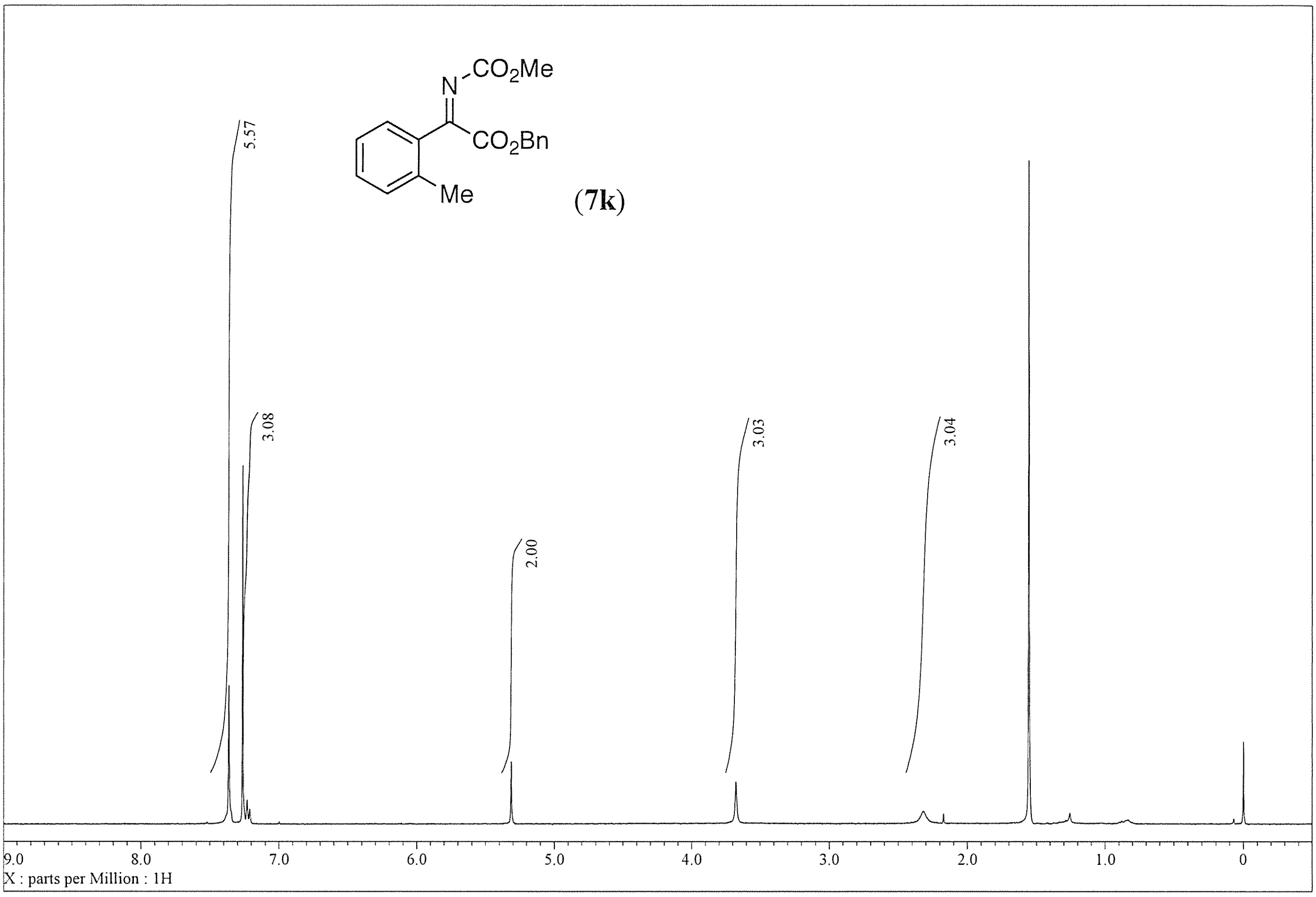

^{1}H NMR, 400 MHz, $CDCl_3$

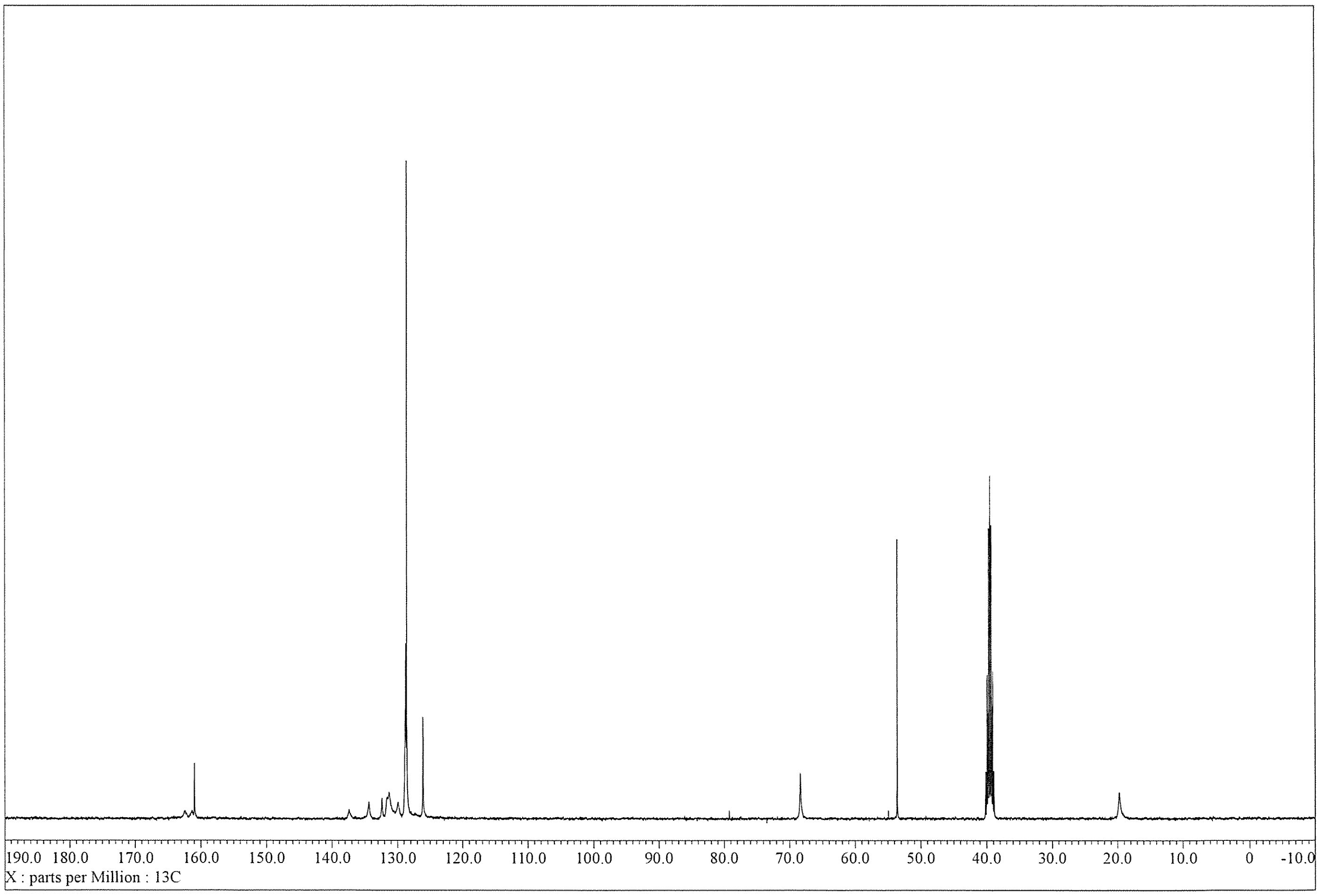

^{13}C NMR, 100 MHz, DMSO-d_6

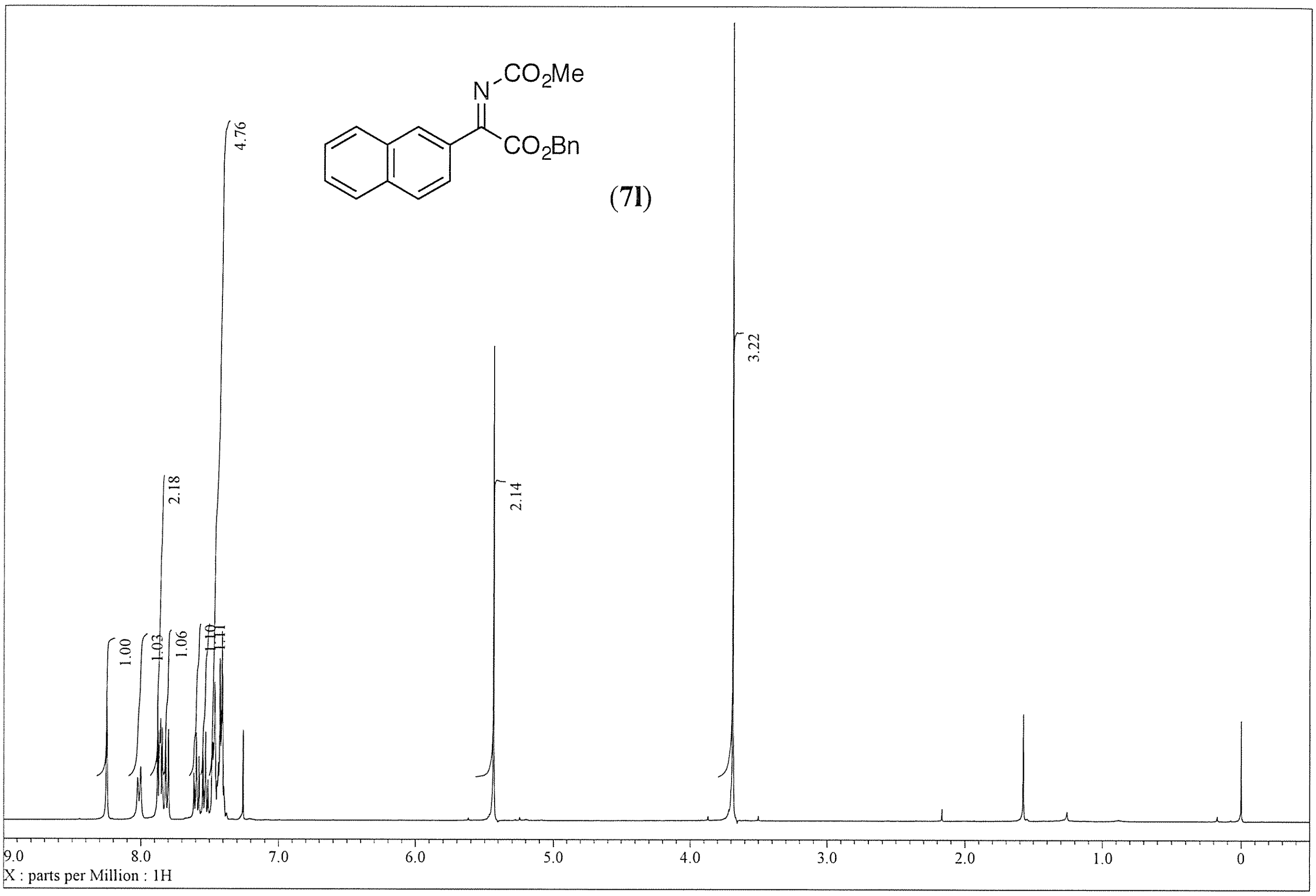

^{1}H NMR, 400 MHz, $CDCl_3$

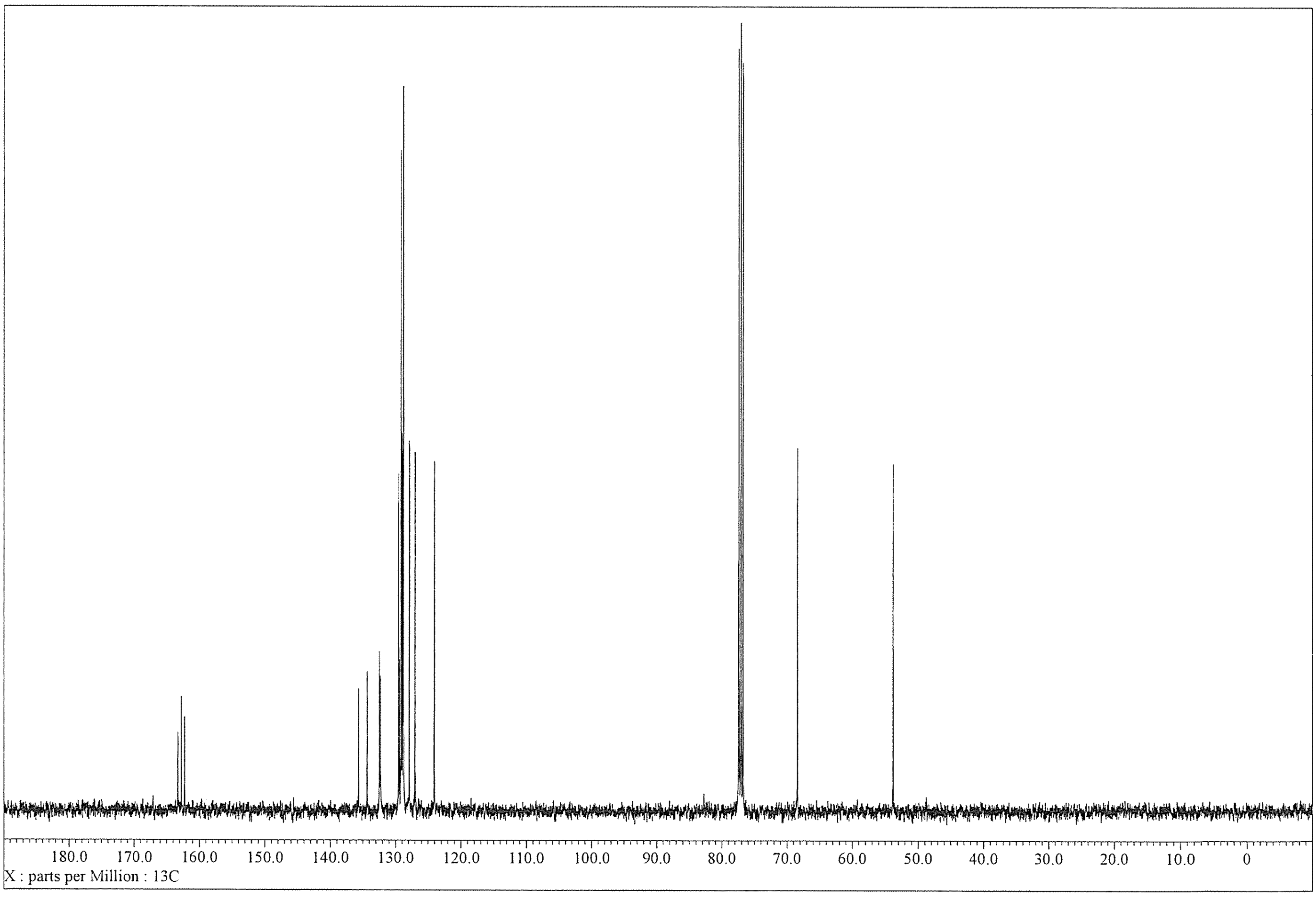

^{13}C NMR, 100 MHz, $CDCl_3$

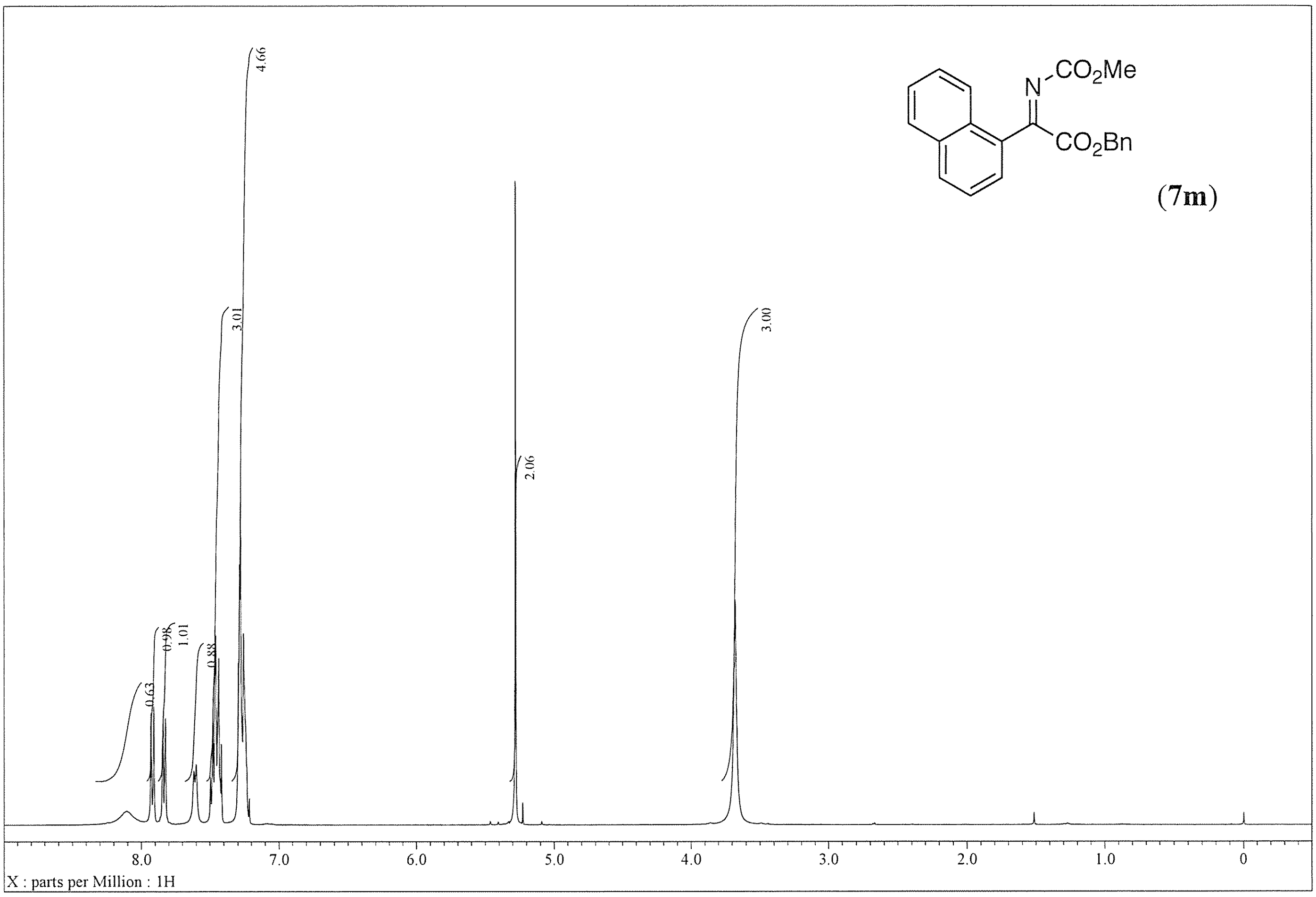

^{1}H NMR, 400 MHz, $CDCl_3$

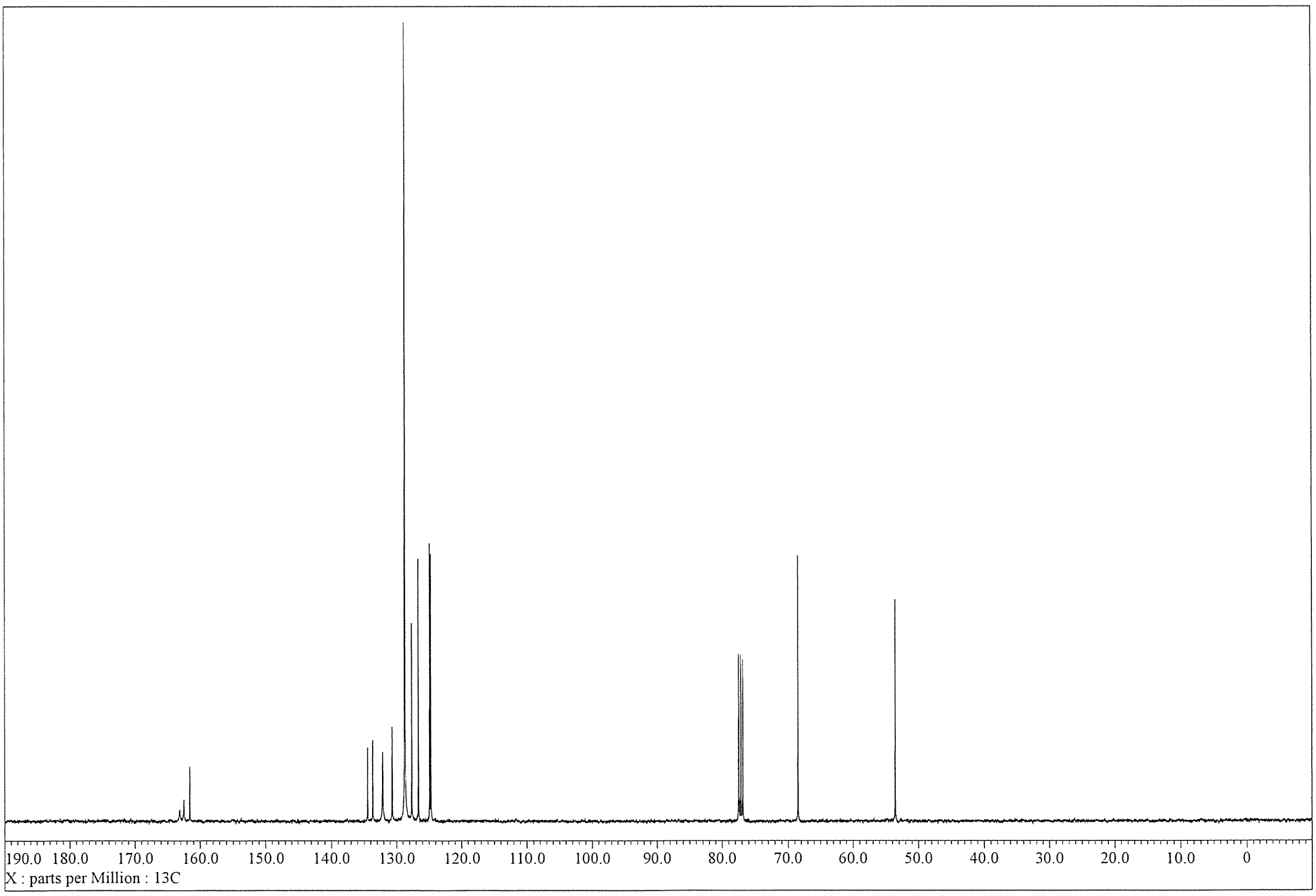

^{13}C NMR, 100 MHz, $CDCl_3$

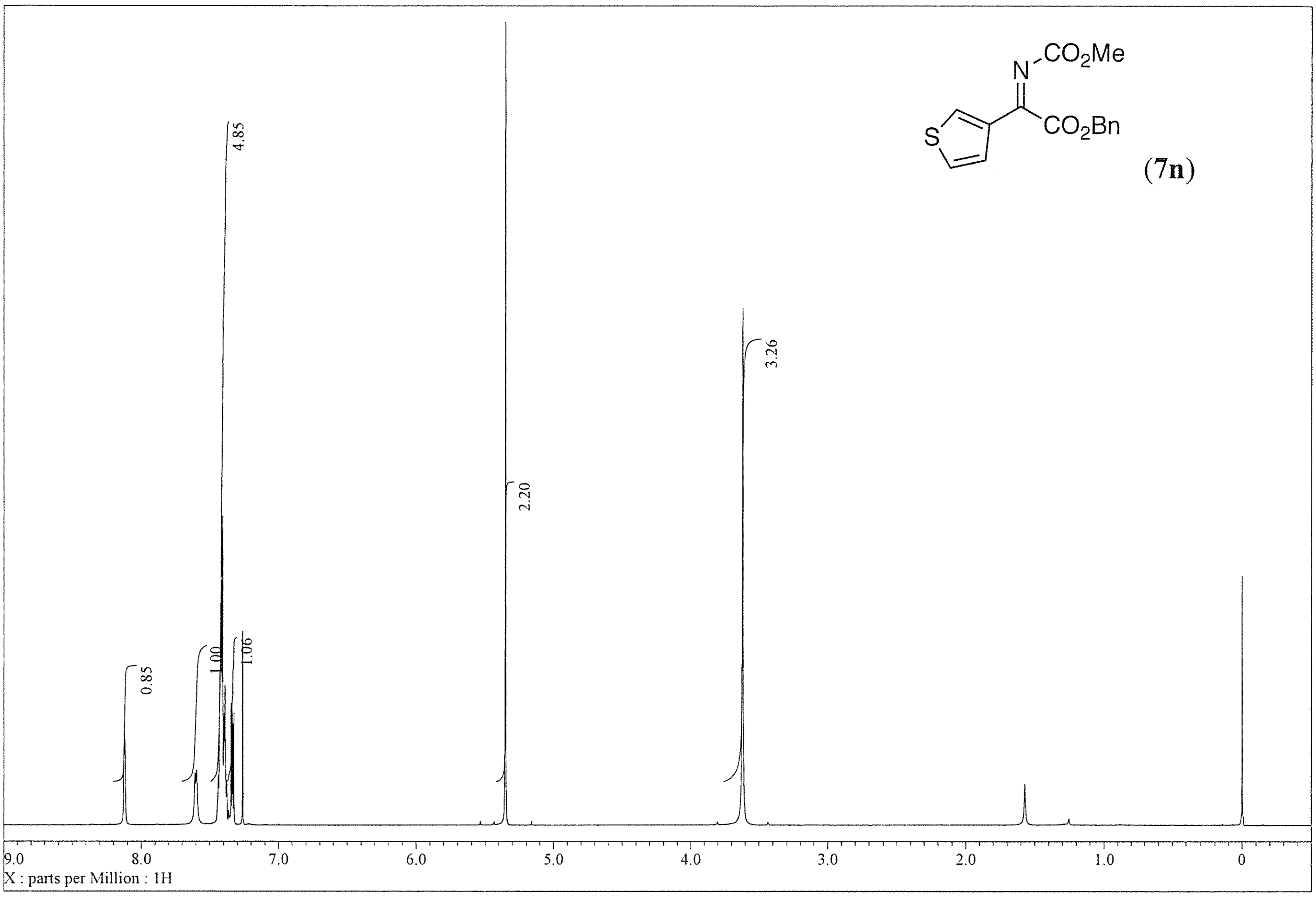

^{1}H NMR, 400 MHz, $CDCl_3$

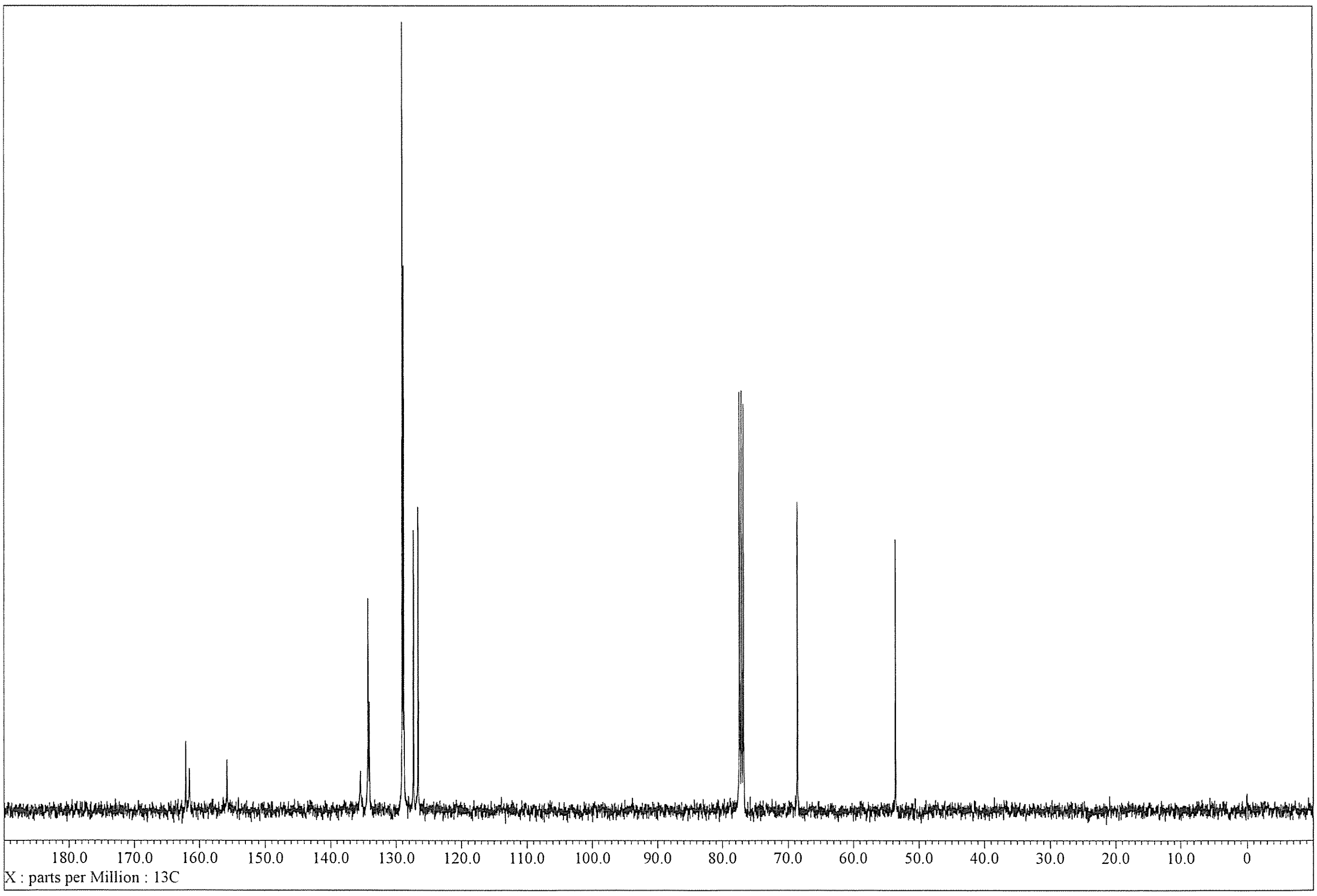

^{13}C NMR, 100 MHz, $CDCl_3$

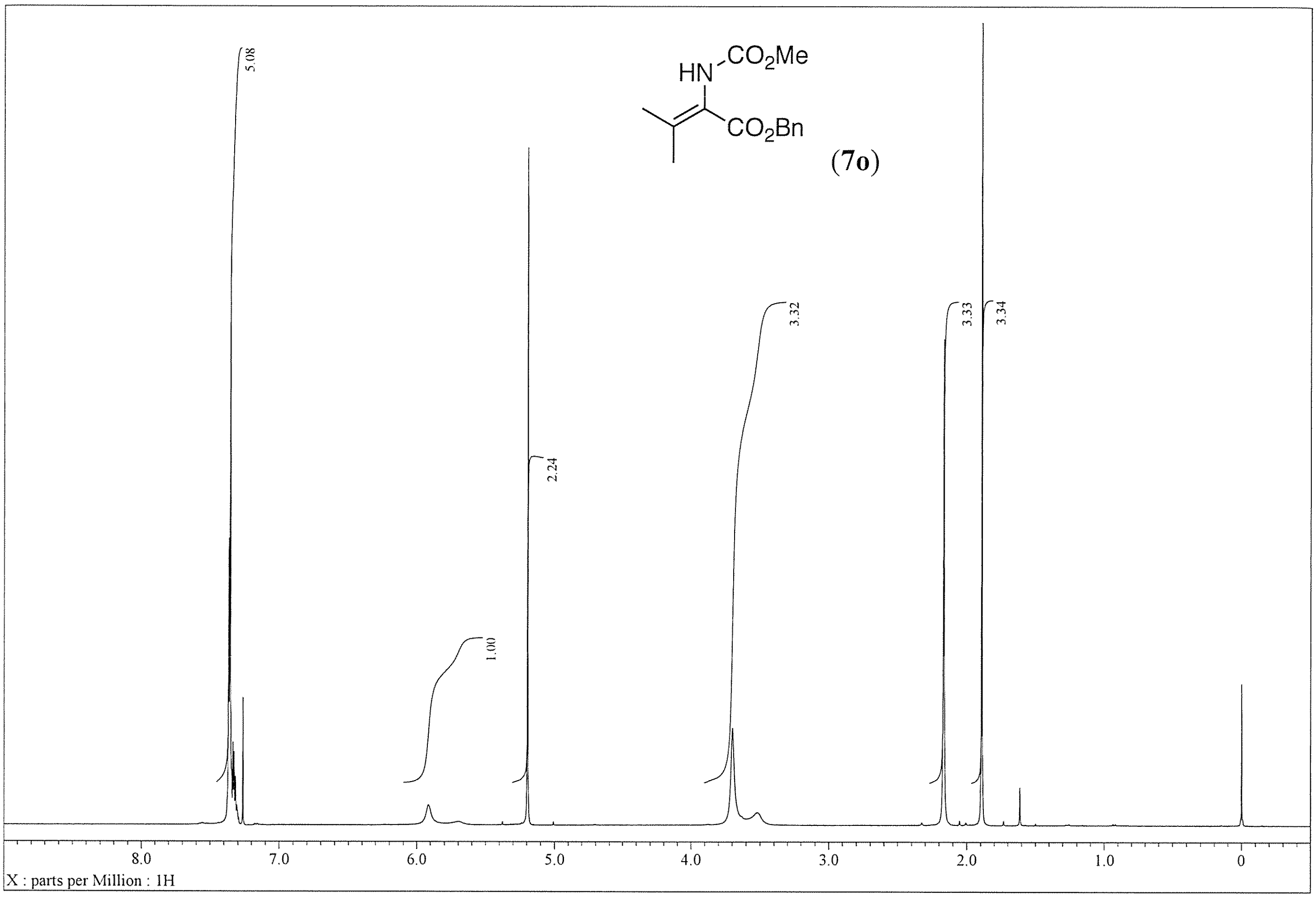

^{1}H NMR, 400 MHz, $CDCl_3$

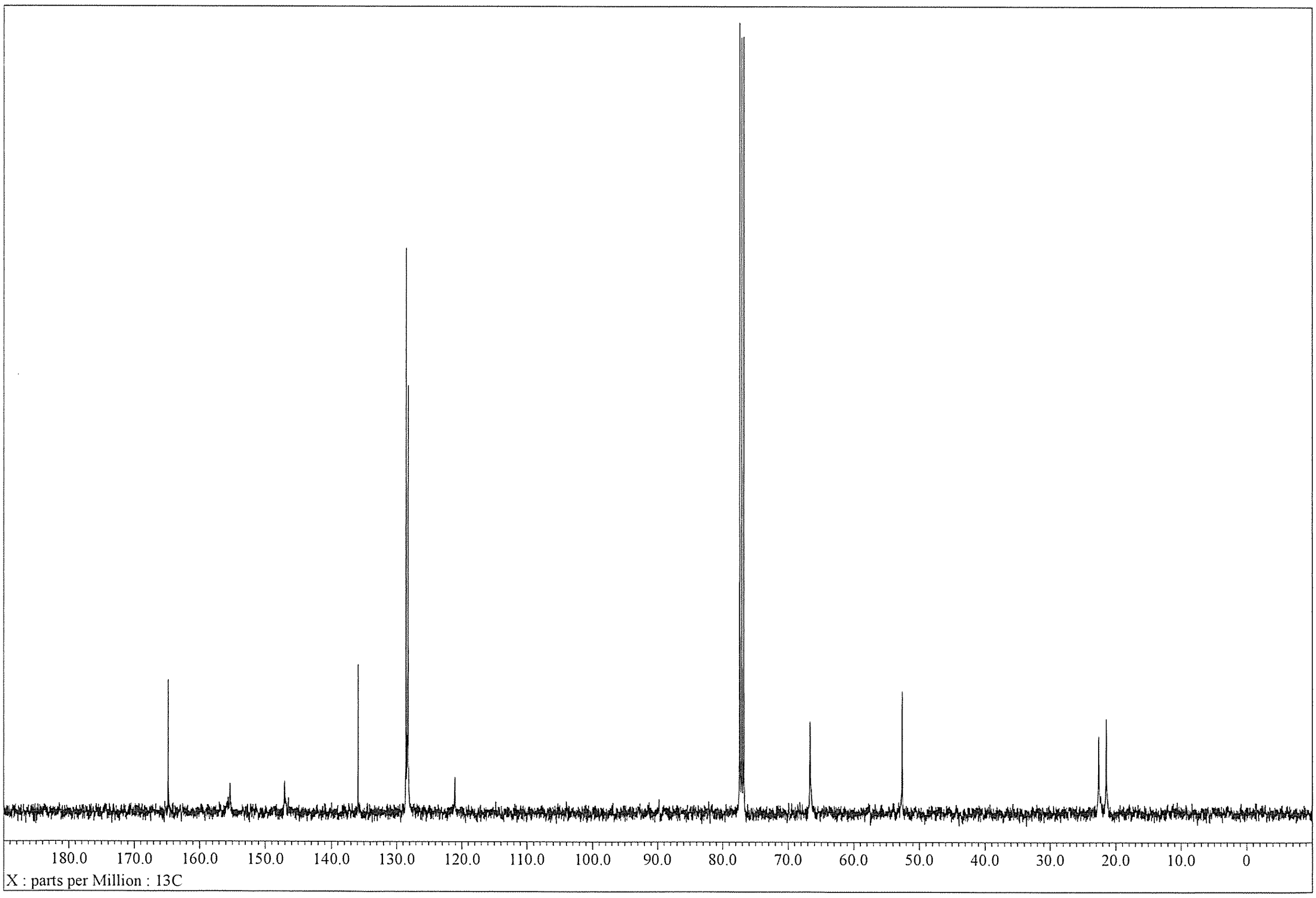

^{13}C NMR, 100 MHz, $CDCl_3$

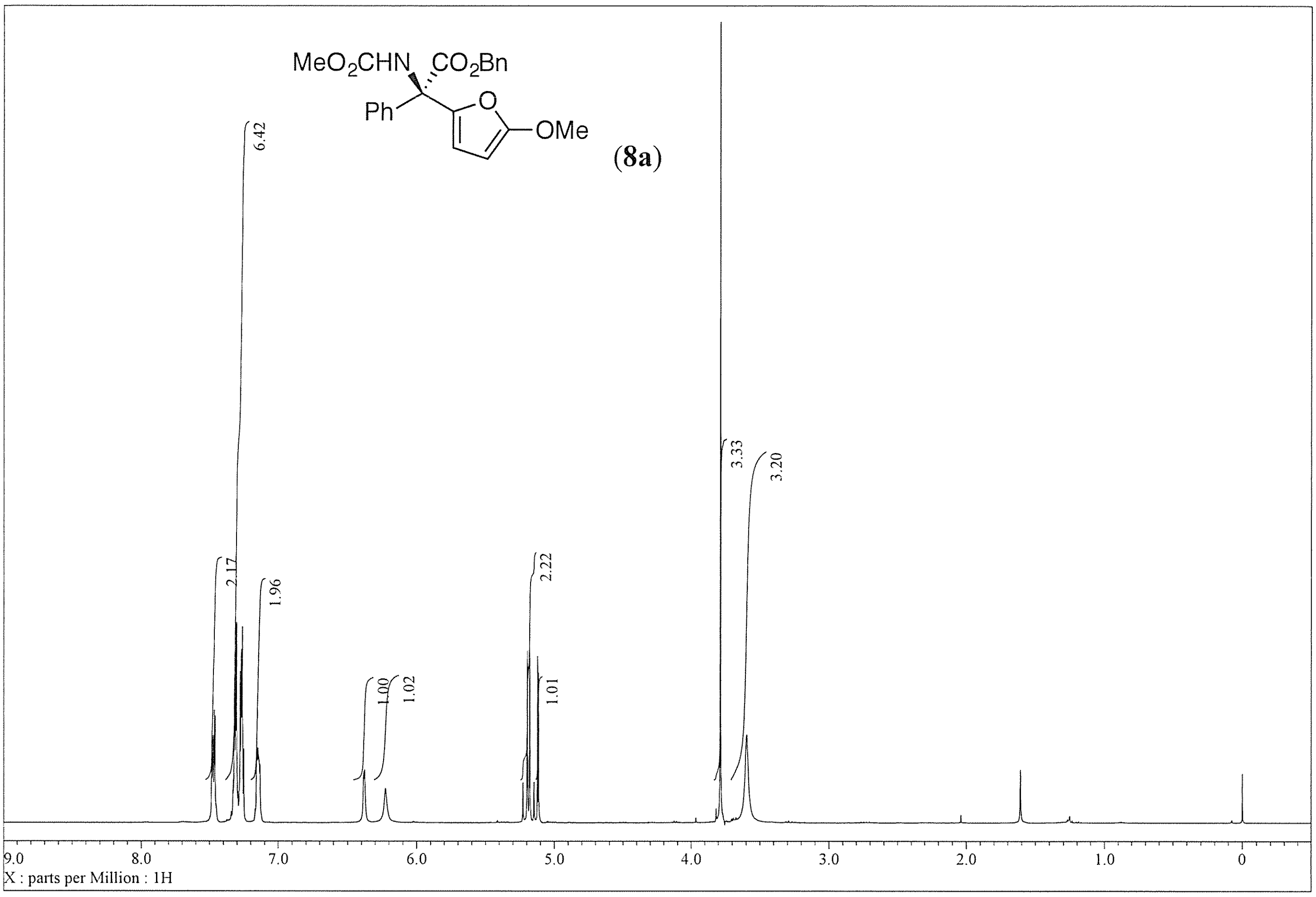

^{1}H NMR, 400 MHz, $CDCl_3$

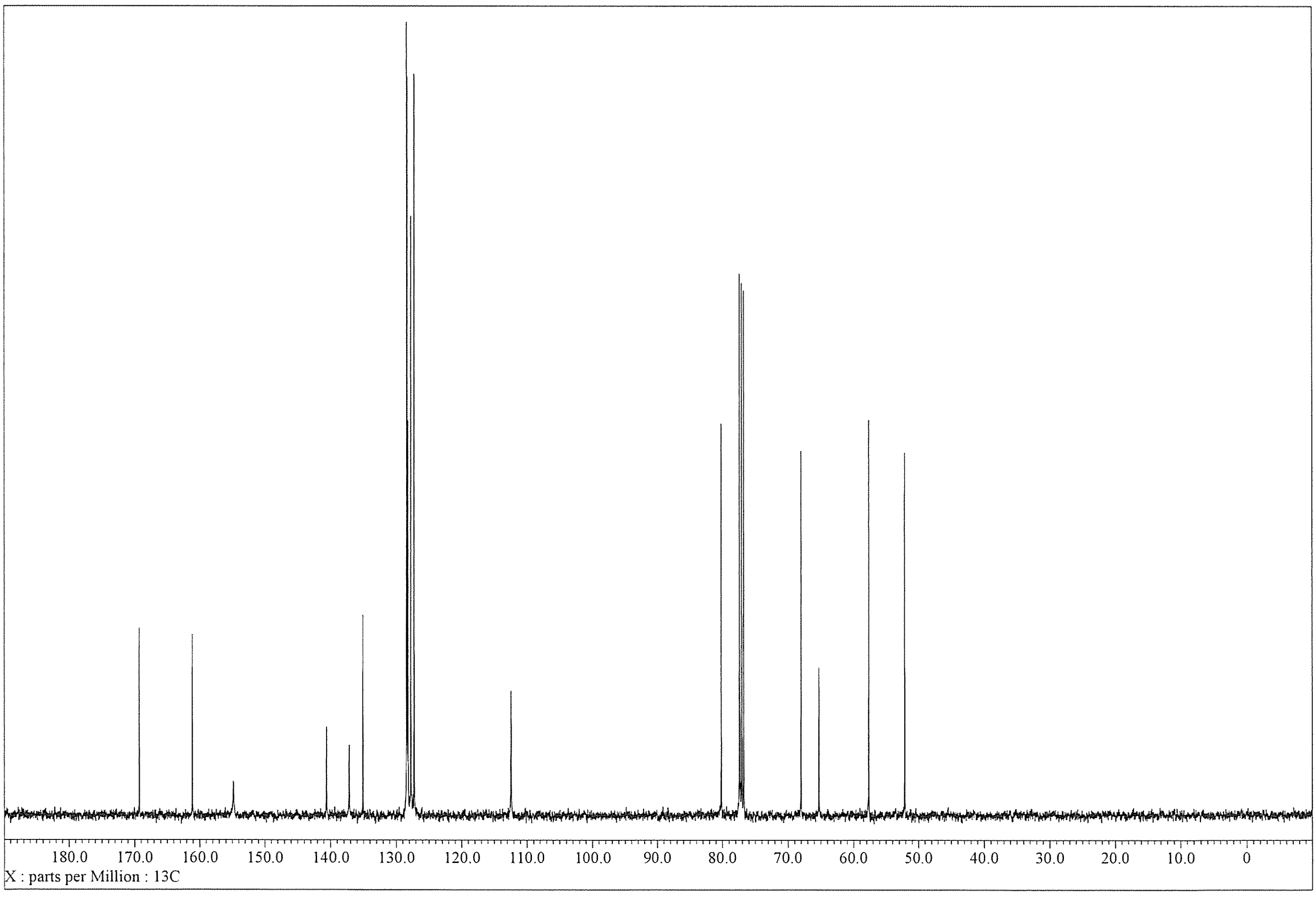

^{13}C NMR, 100 MHz, $CDCl_3$

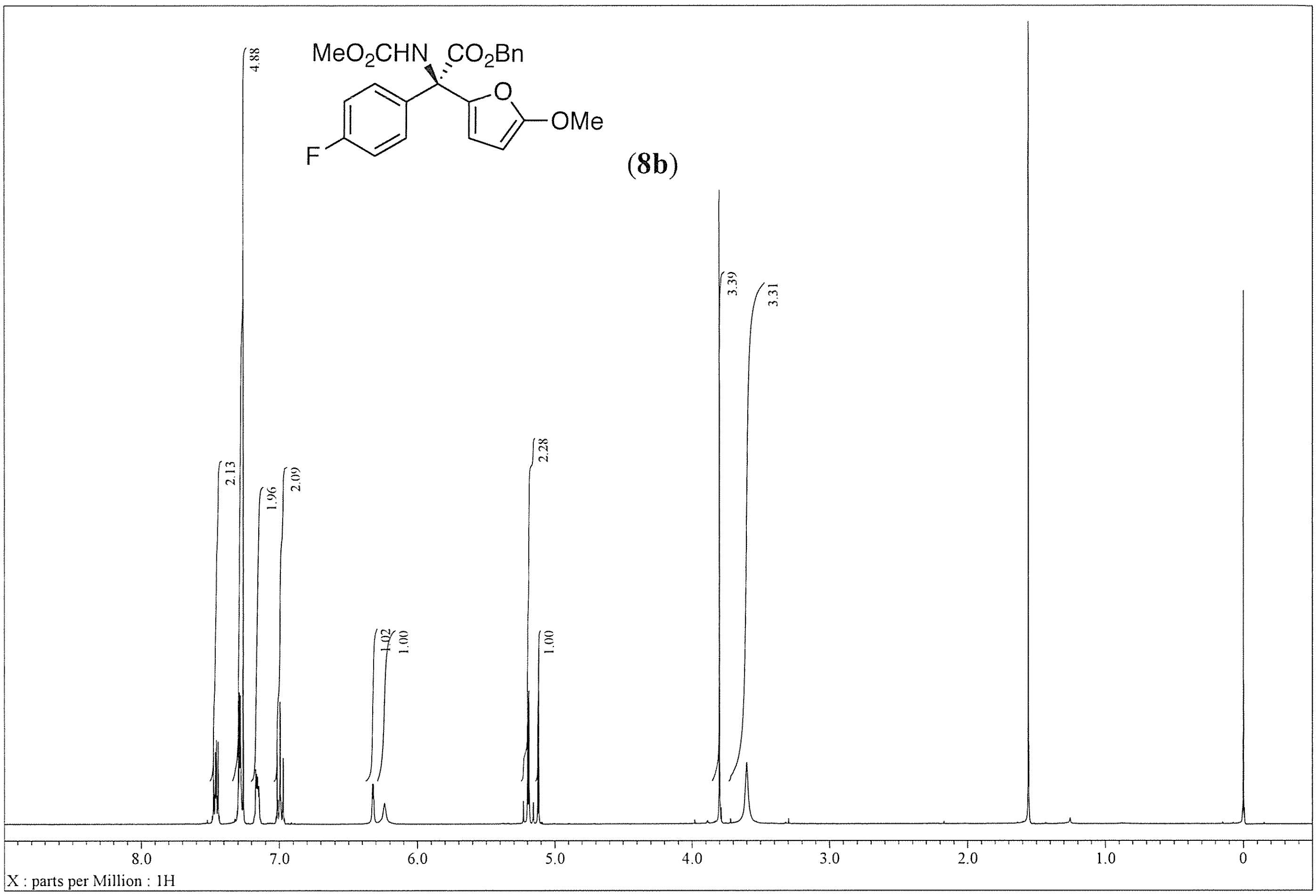

^{1}H NMR, 400 MHz, $CDCl_3$

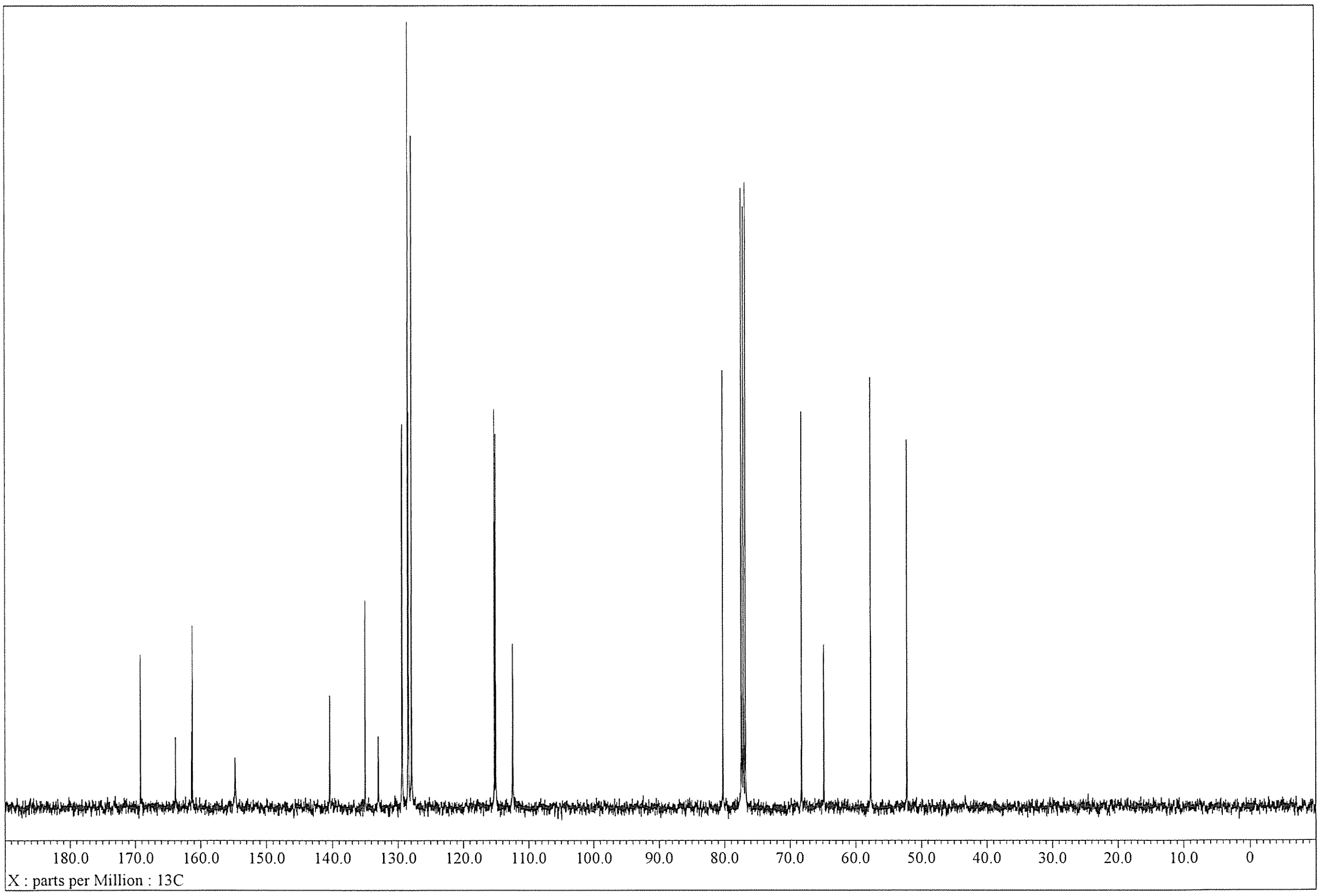

^{13}C NMR, 100 MHz, $CDCl_3$

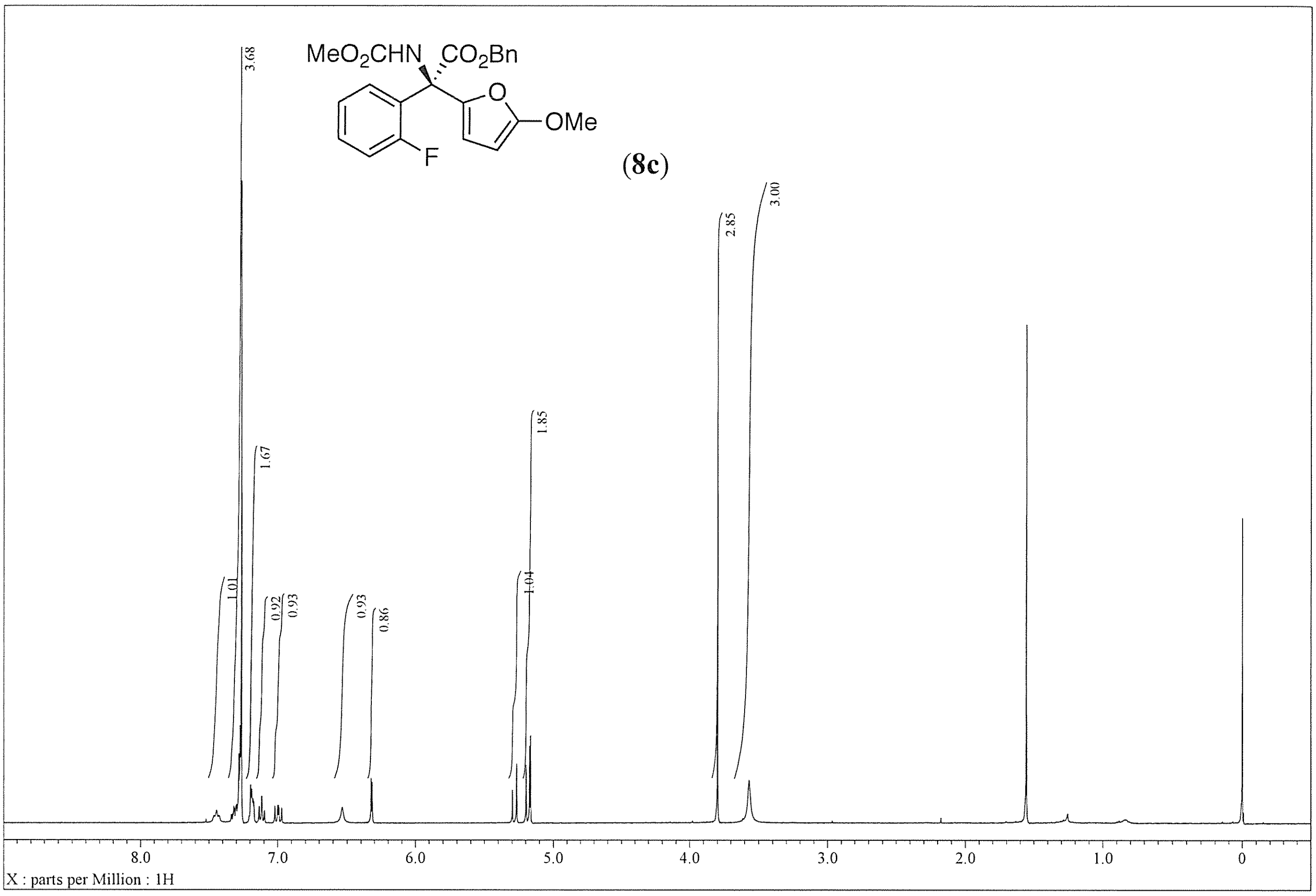

^{1}H NMR, 400 MHz, $CDCl_3$

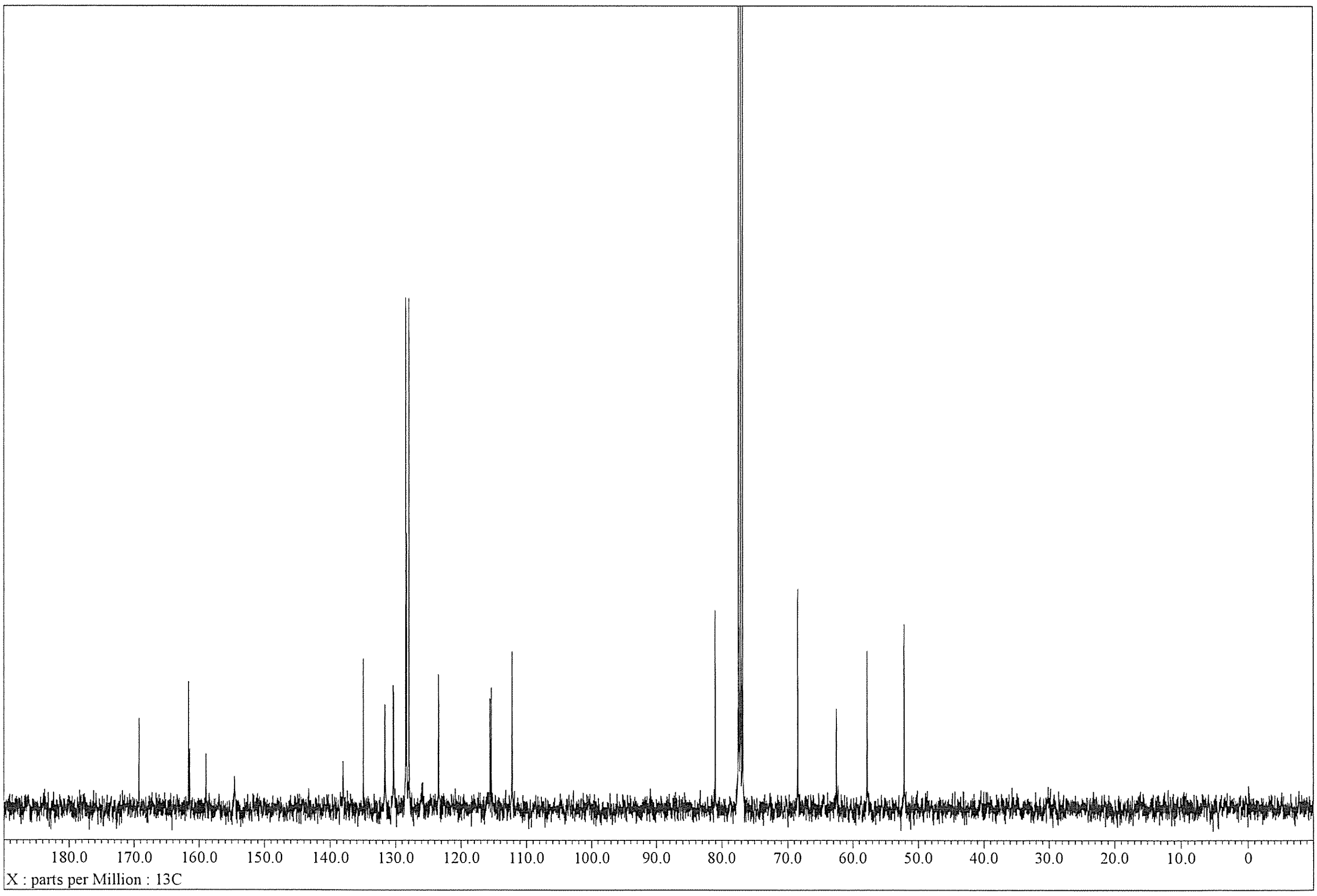

^{13}C NMR, 100 MHz, $CDCl_3$

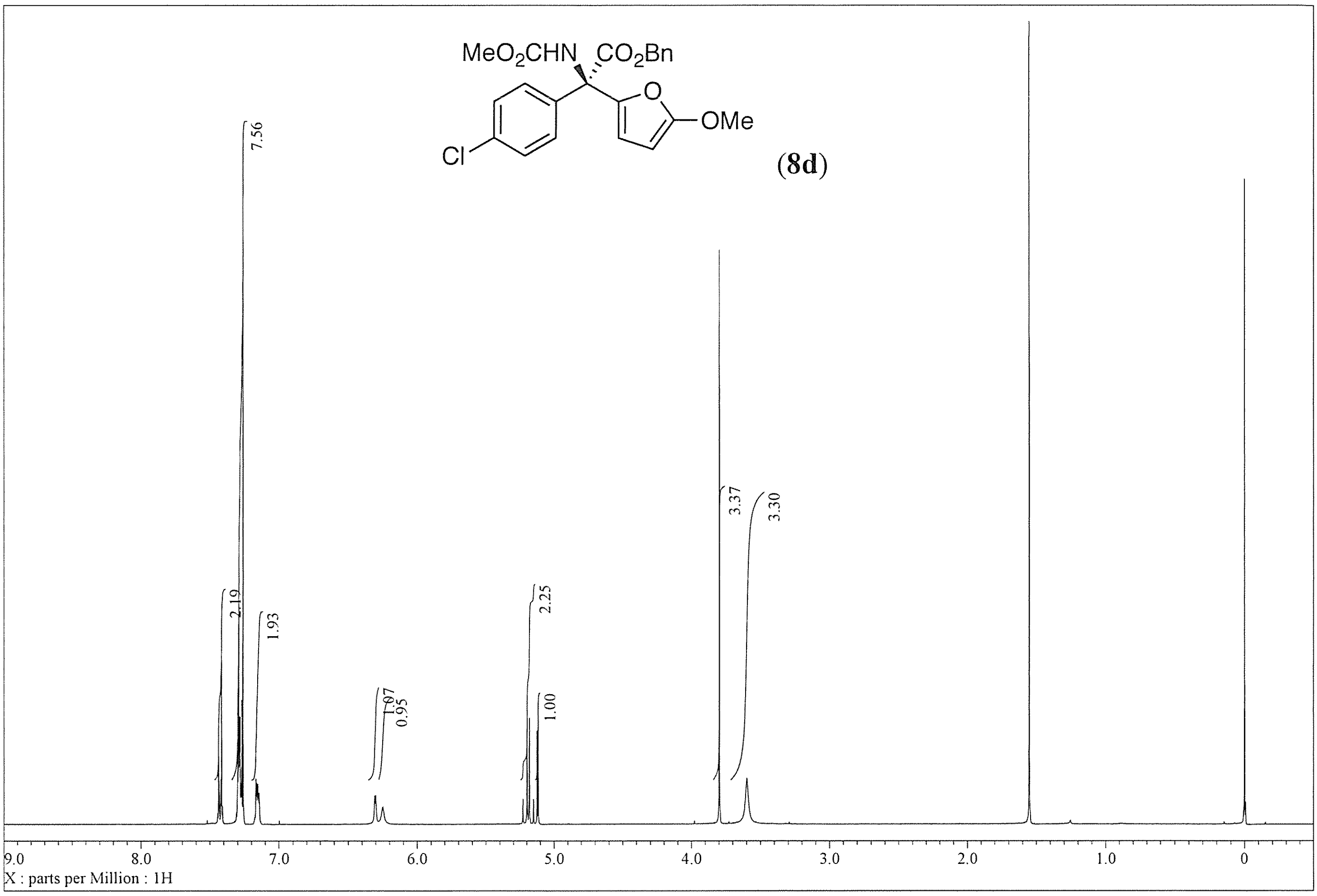

^{1}H NMR, 400 MHz, $CDCl_3$

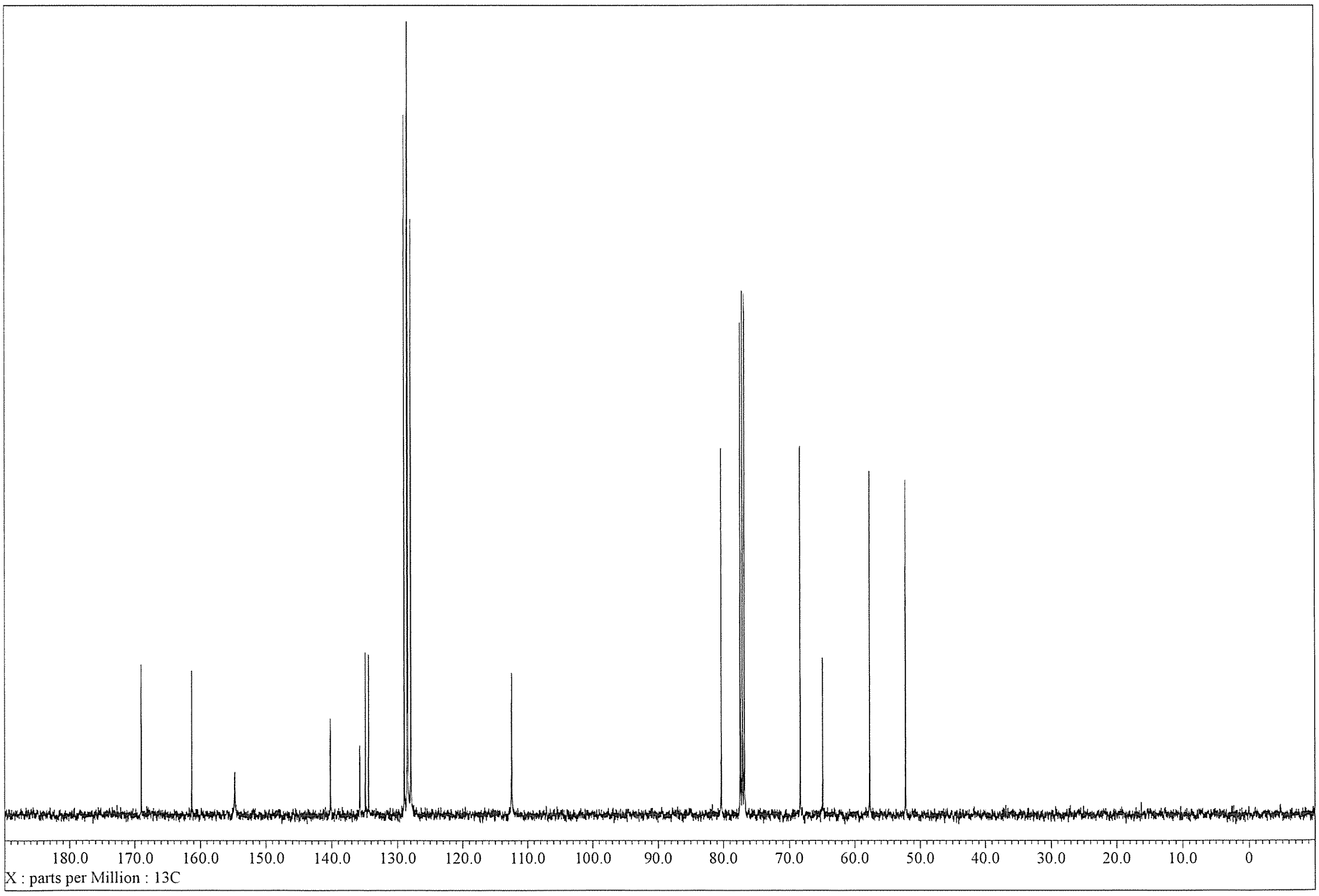

^{13}C NMR, 100 MHz, $CDCl_3$

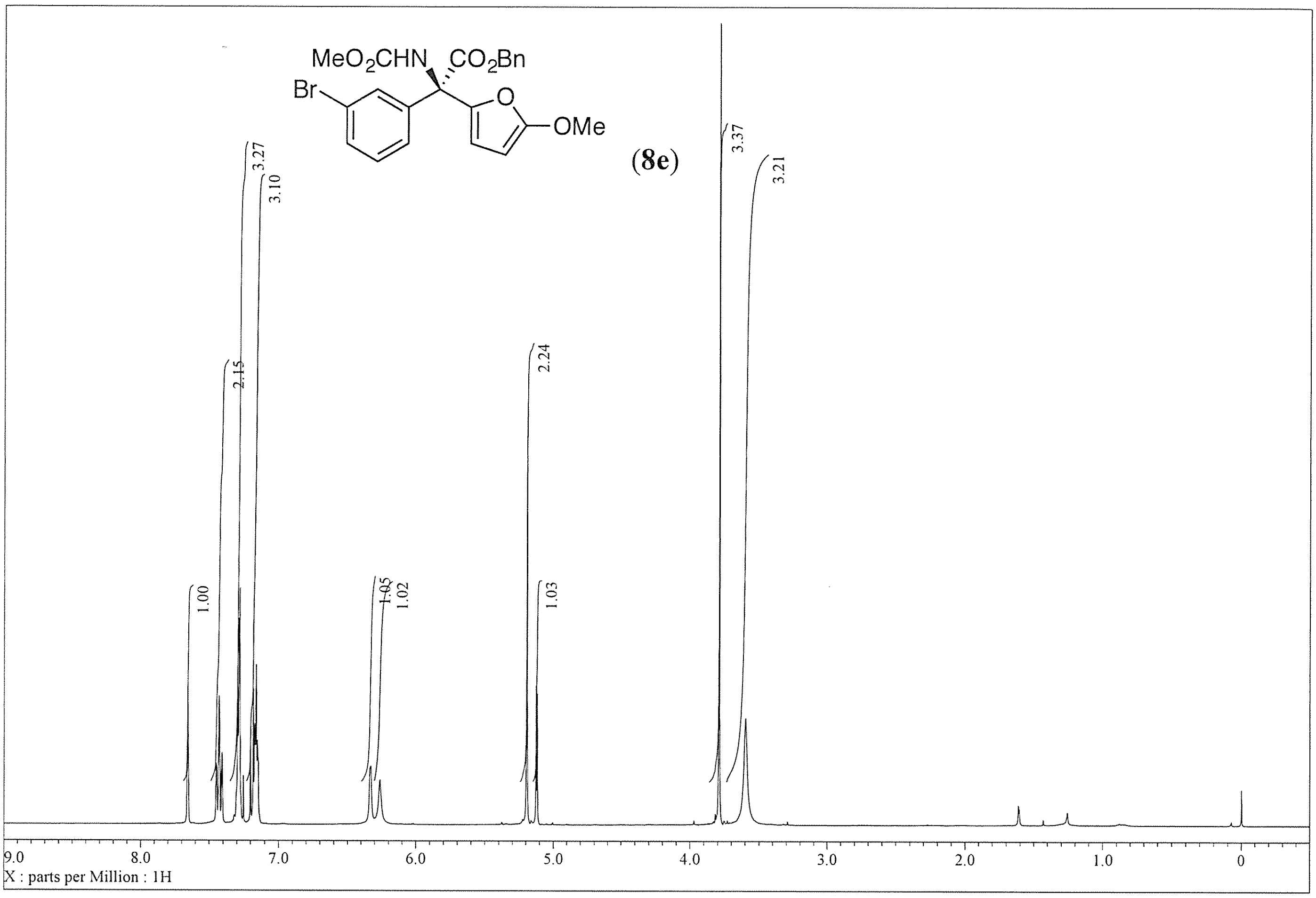

^{1}H NMR, 400 MHz, $CDCl_3$

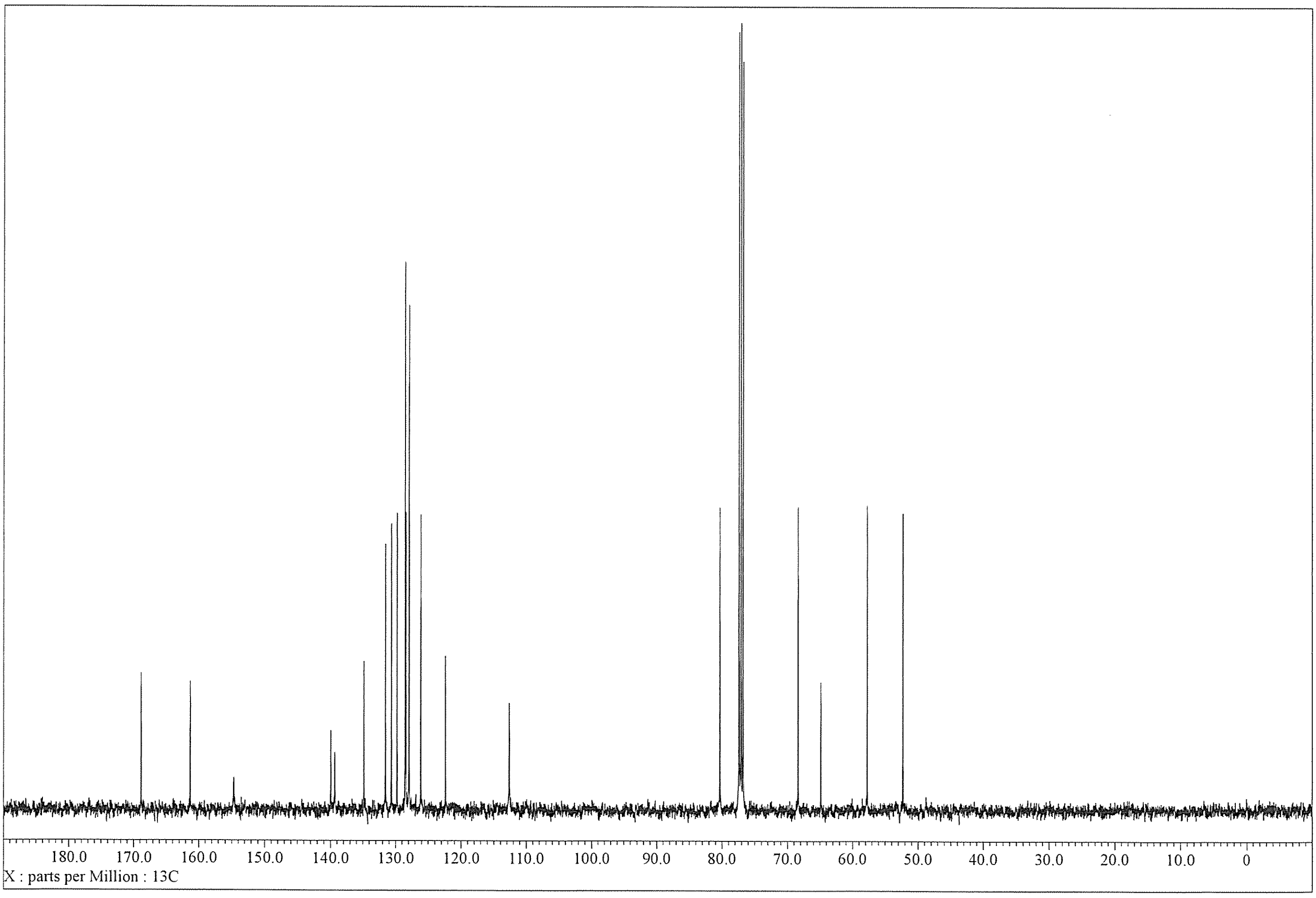

^{13}C NMR, 100 MHz, $CDCl_3$

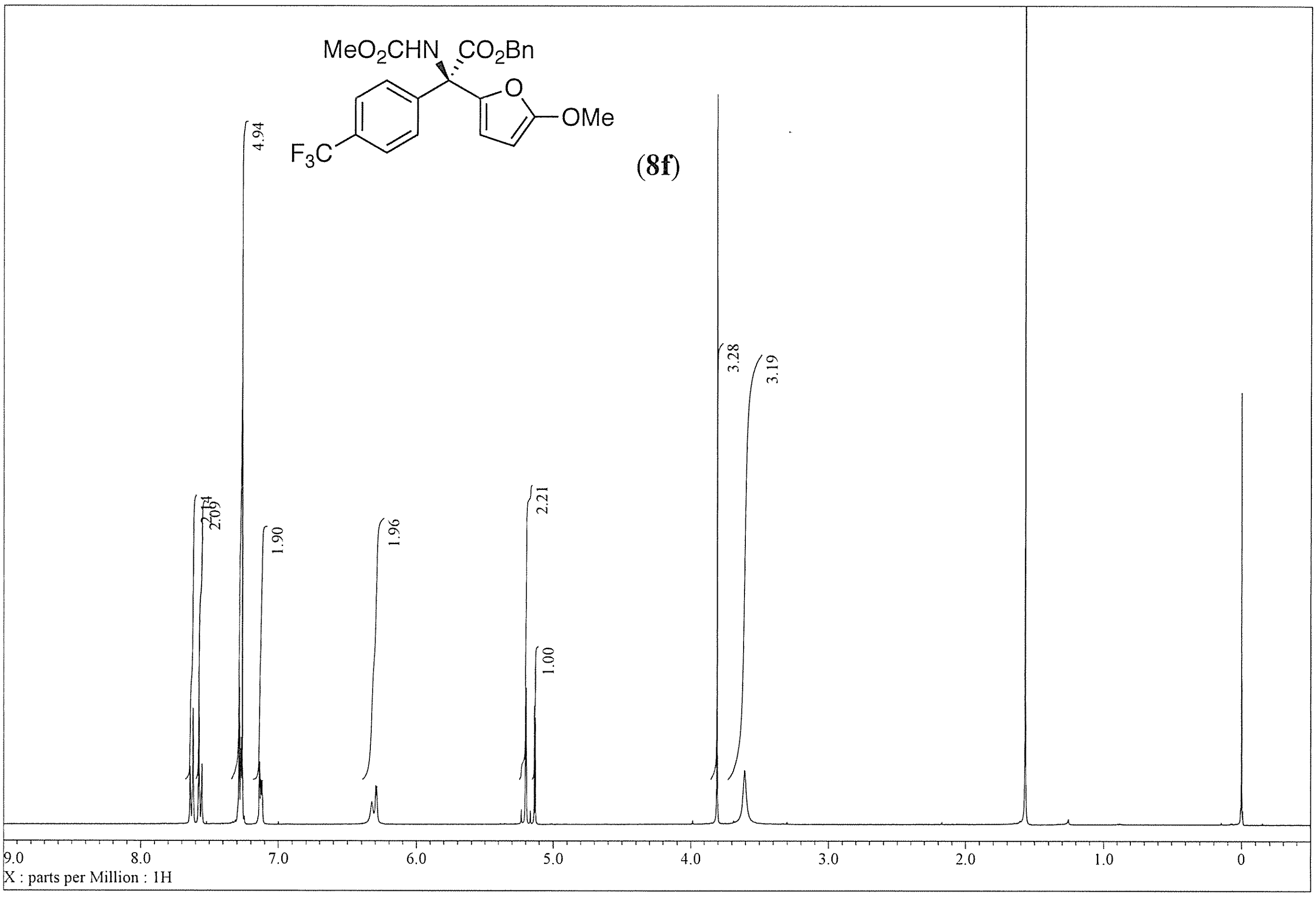

^{1}H NMR, 400 MHz, $CDCl_3$

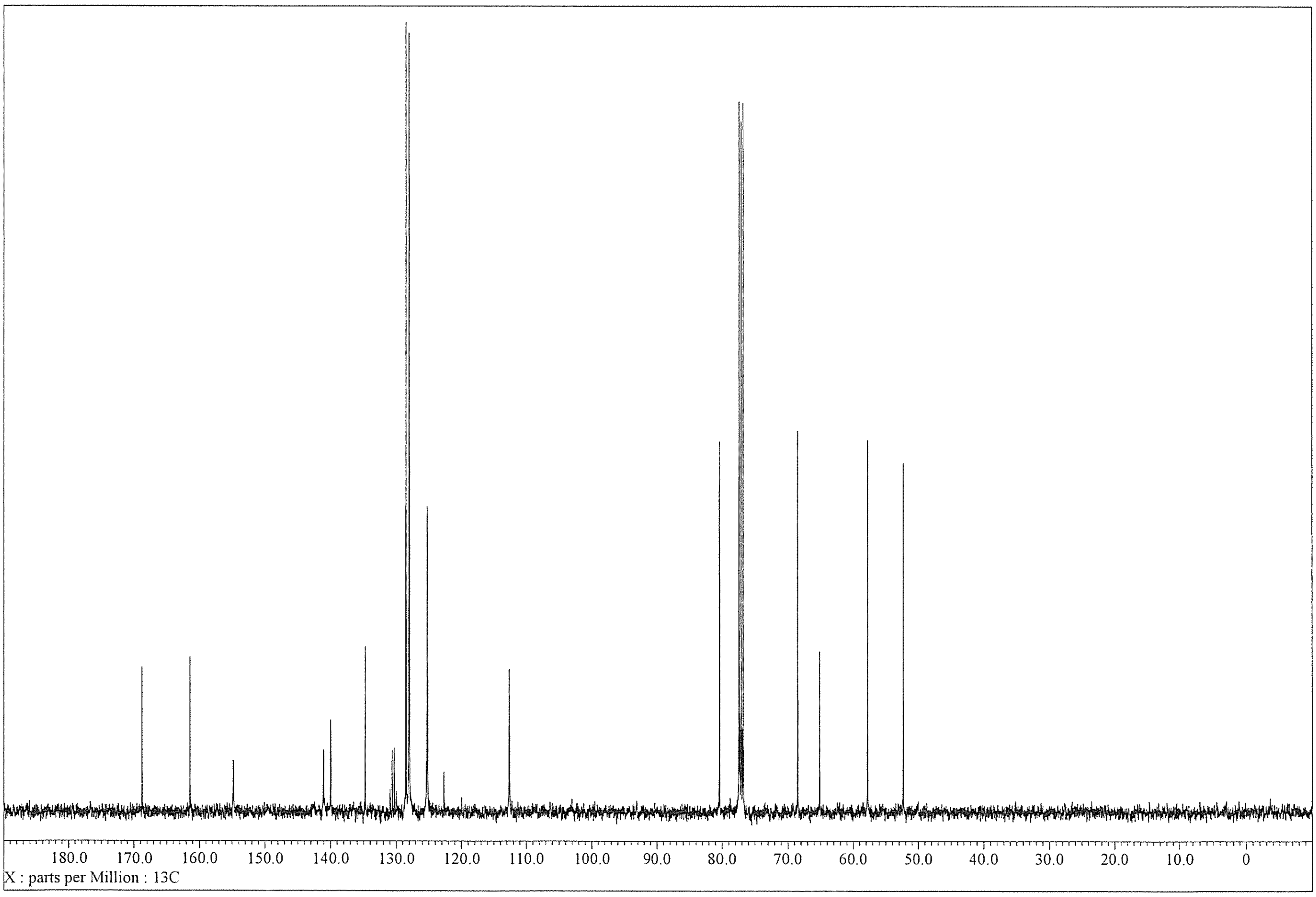

^{13}C NMR, 100 MHz, $CDCl_3$

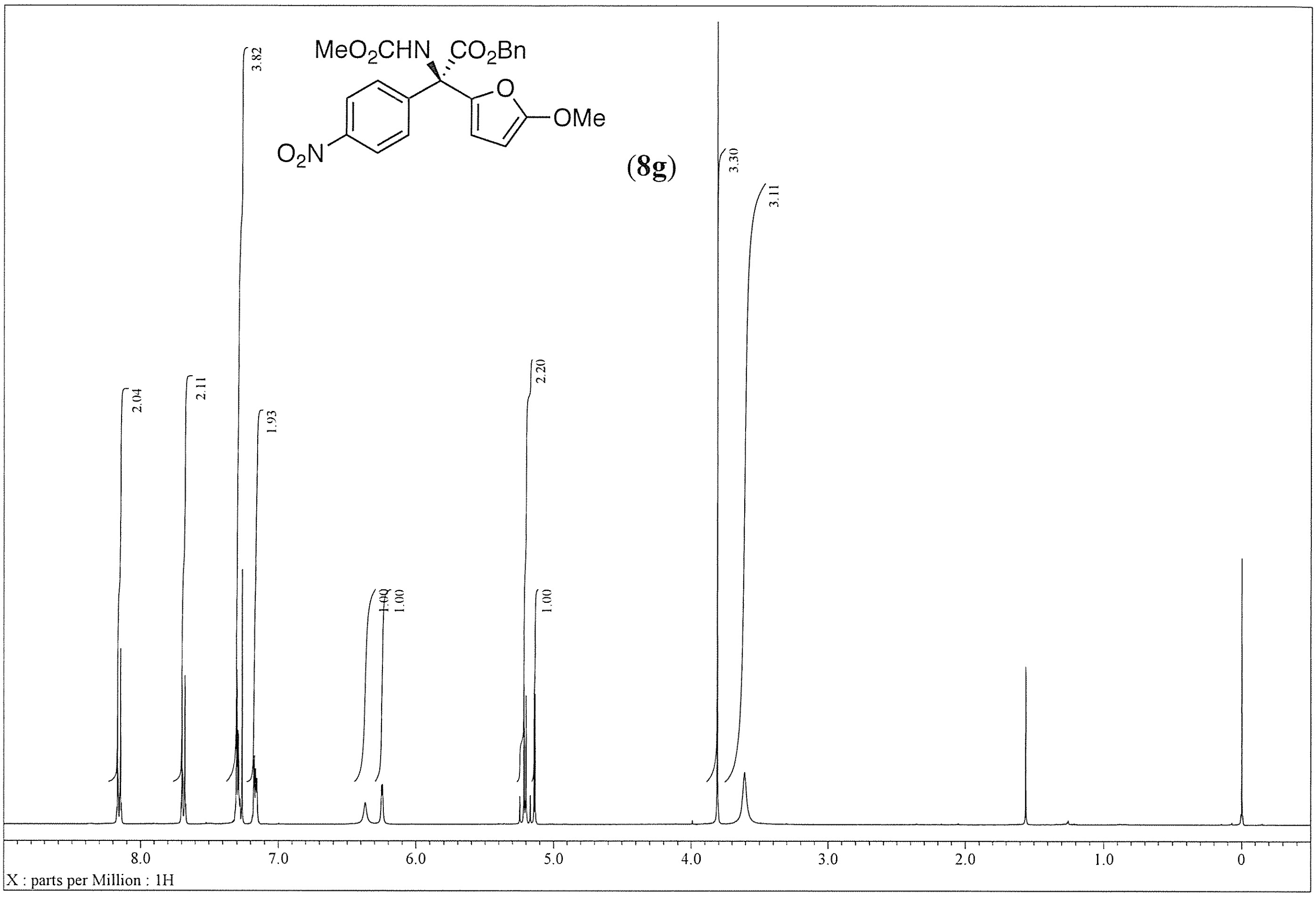

^{1}H NMR, 400 MHz, $CDCl_3$

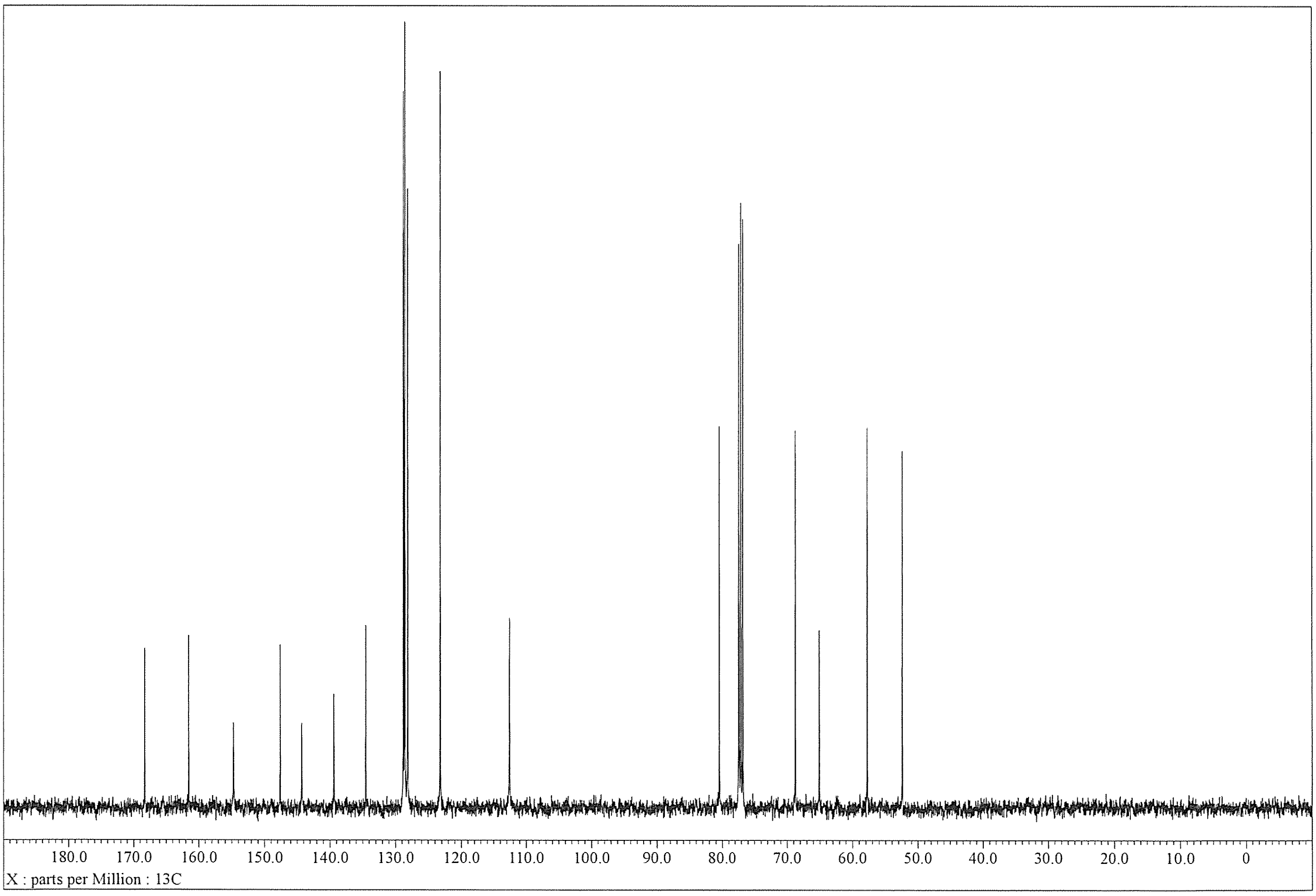

^{13}C NMR, 100 MHz, $CDCl_3$

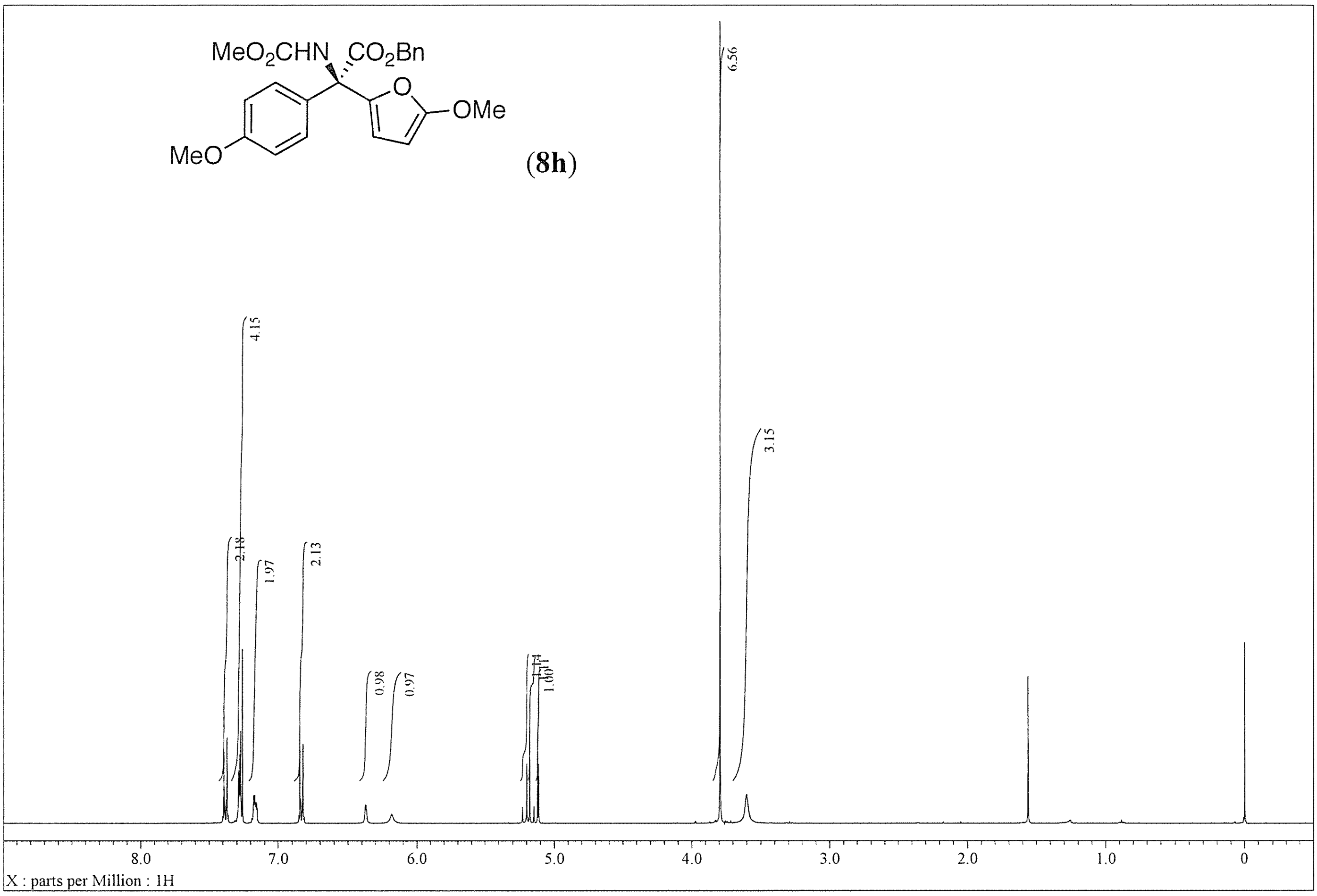

^{1}H NMR, 400 MHz, $CDCl_3$

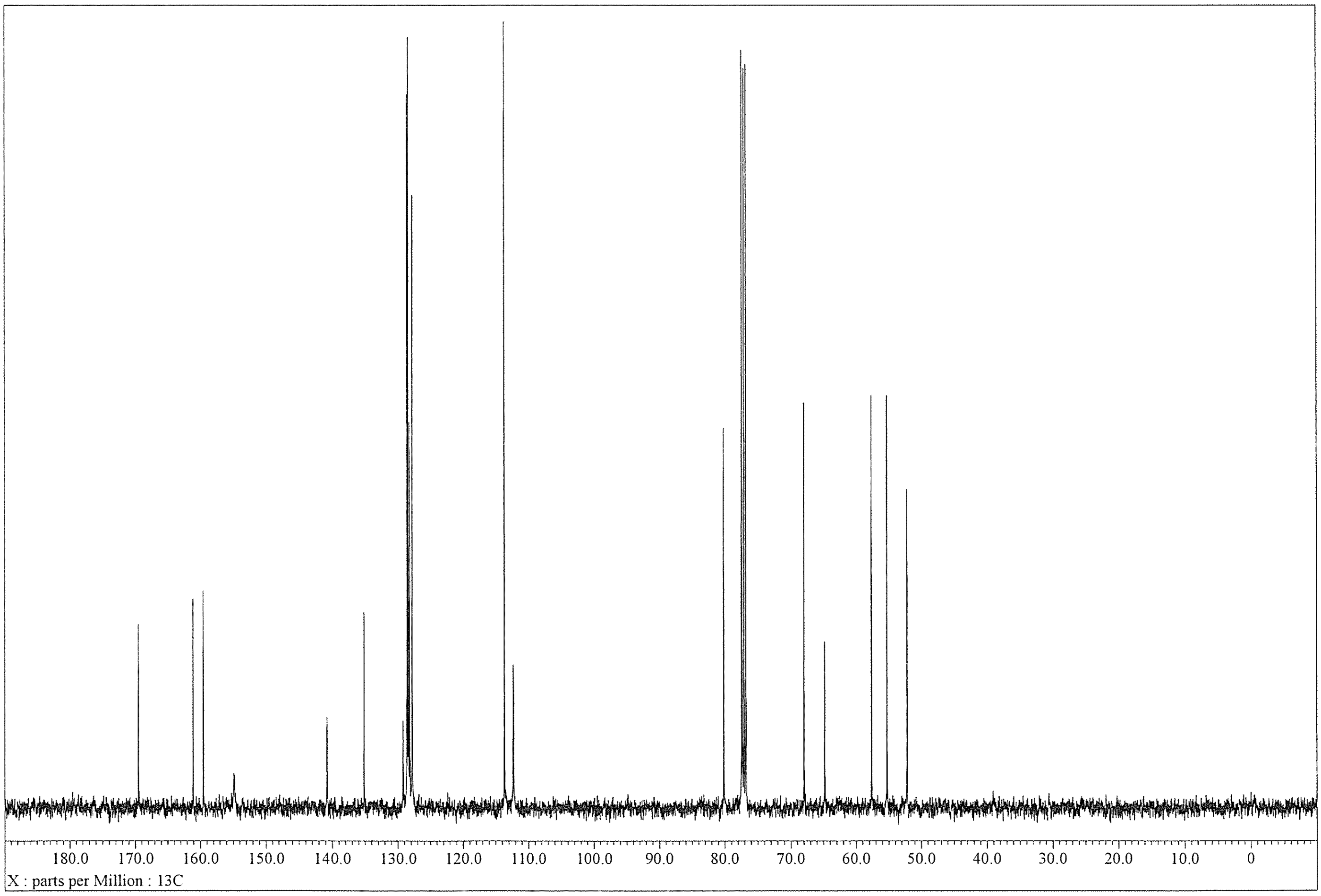

^{13}C NMR, 100 MHz, $CDCl_3$

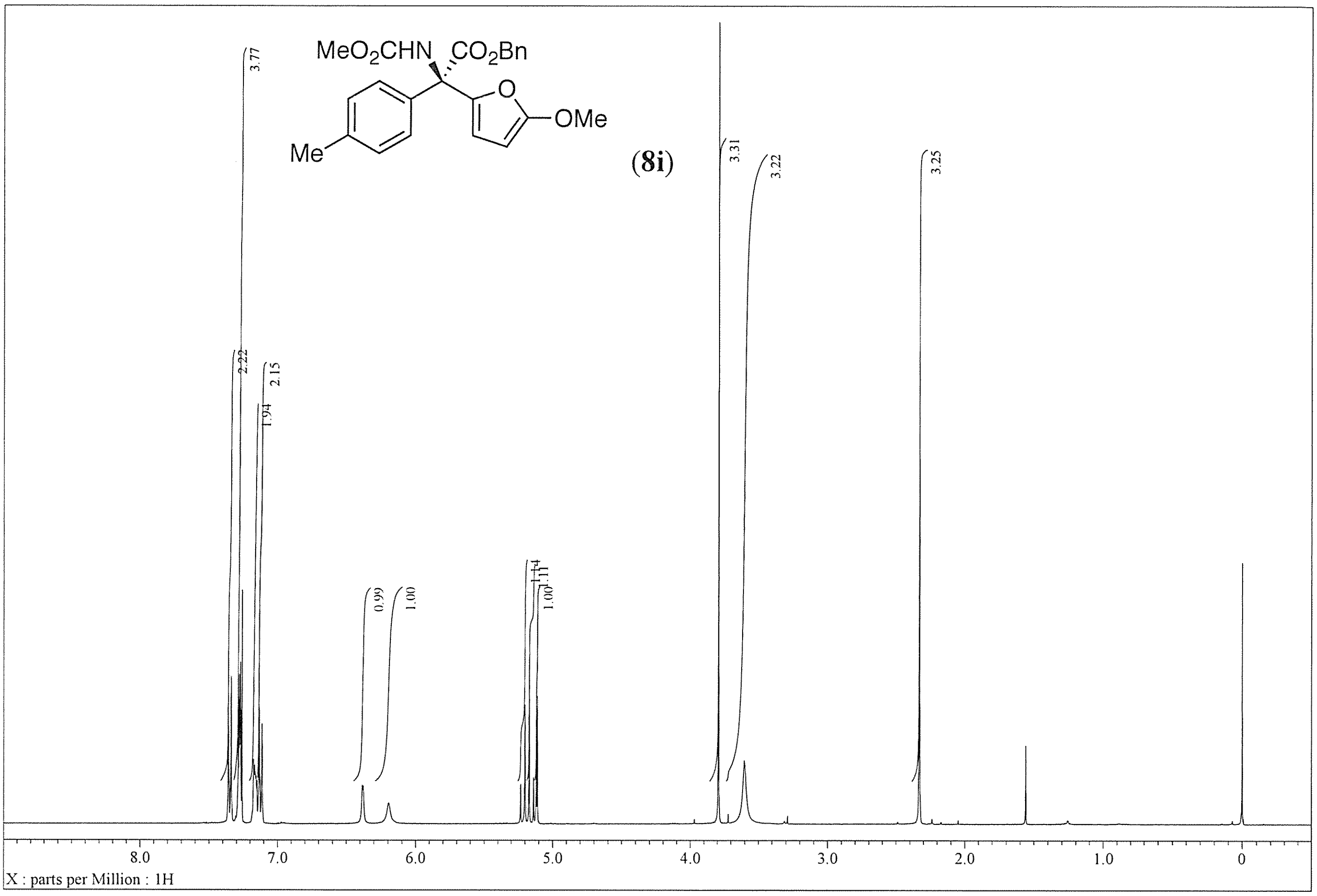

^{1}H NMR, 400 MHz, $CDCl_3$

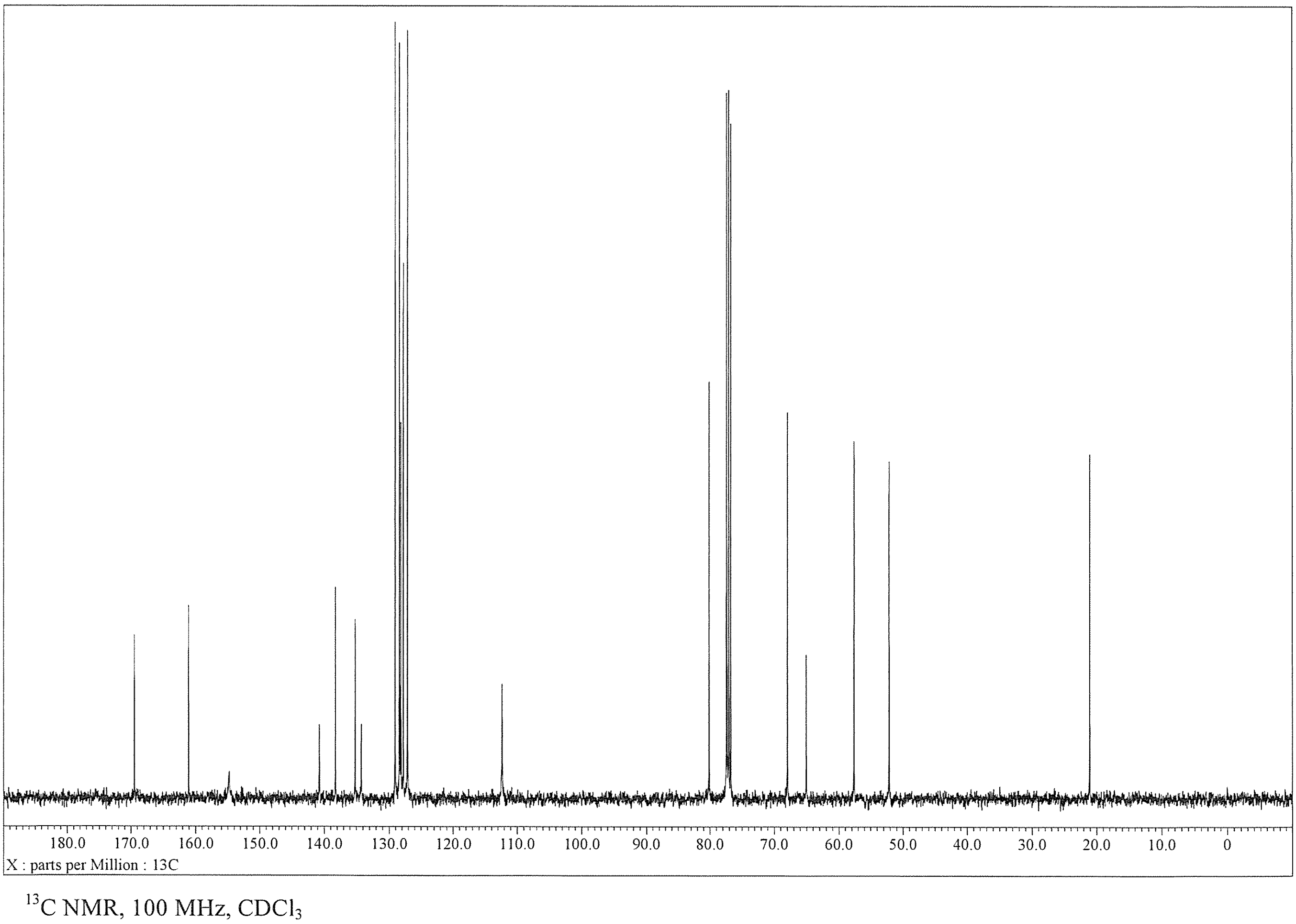

^{13}C NMR, 100 MHz, $CDCl_3$

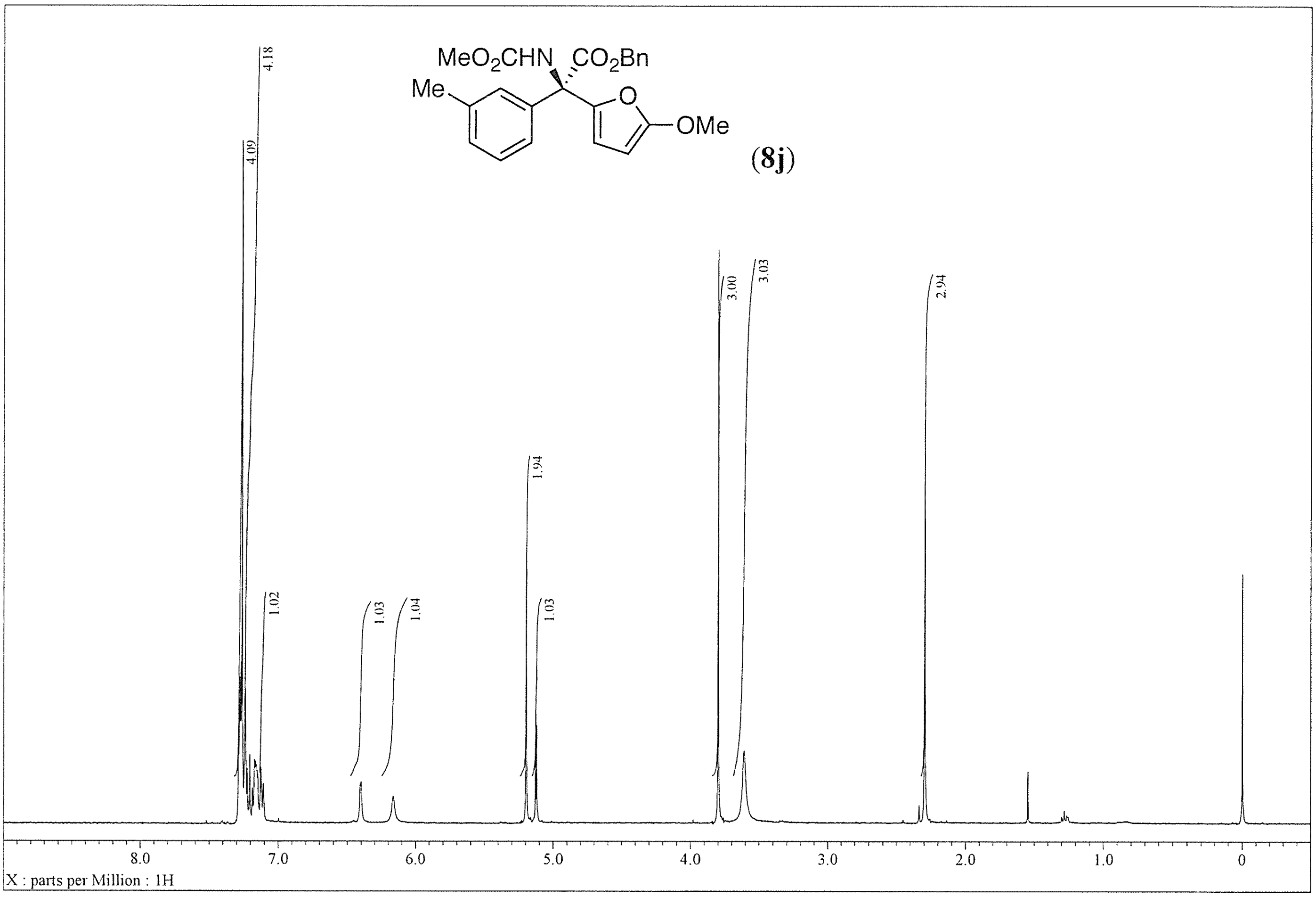

^{1}H NMR, 400 MHz, $CDCl_3$

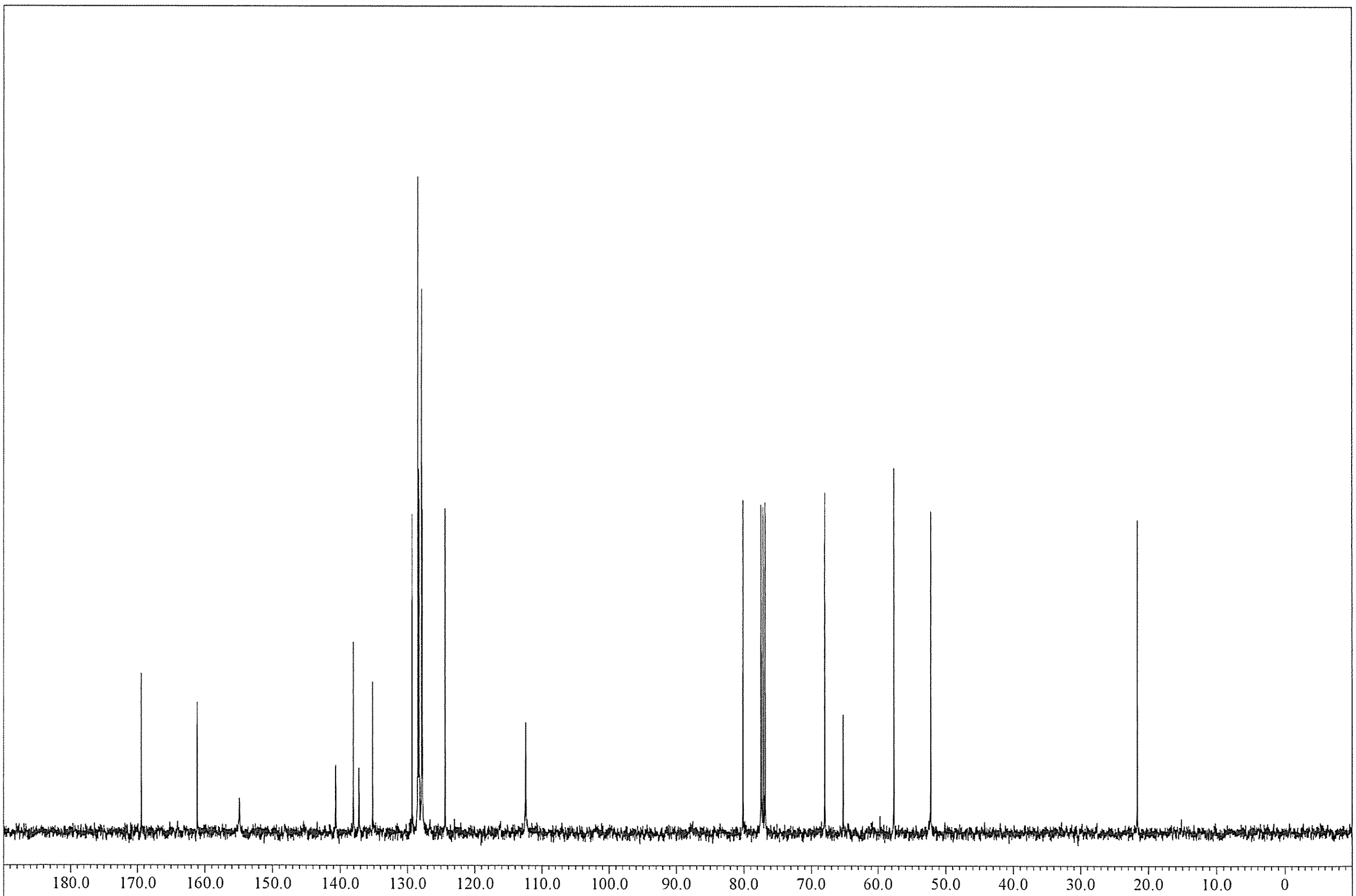

^{13}C NMR, 100 MHz, $CDCl_3$

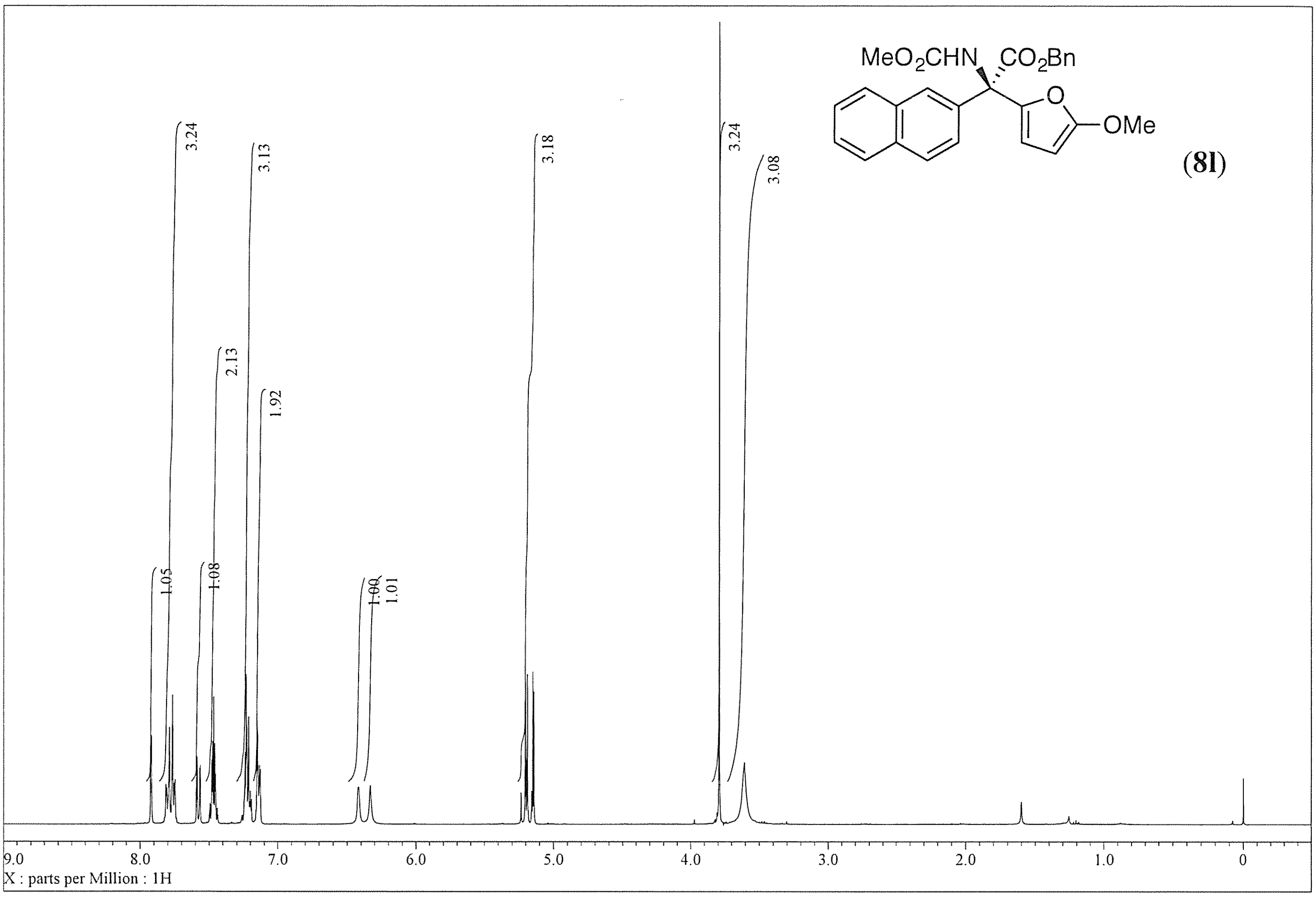

^{1}H NMR, 400 MHz, $CDCl_3$

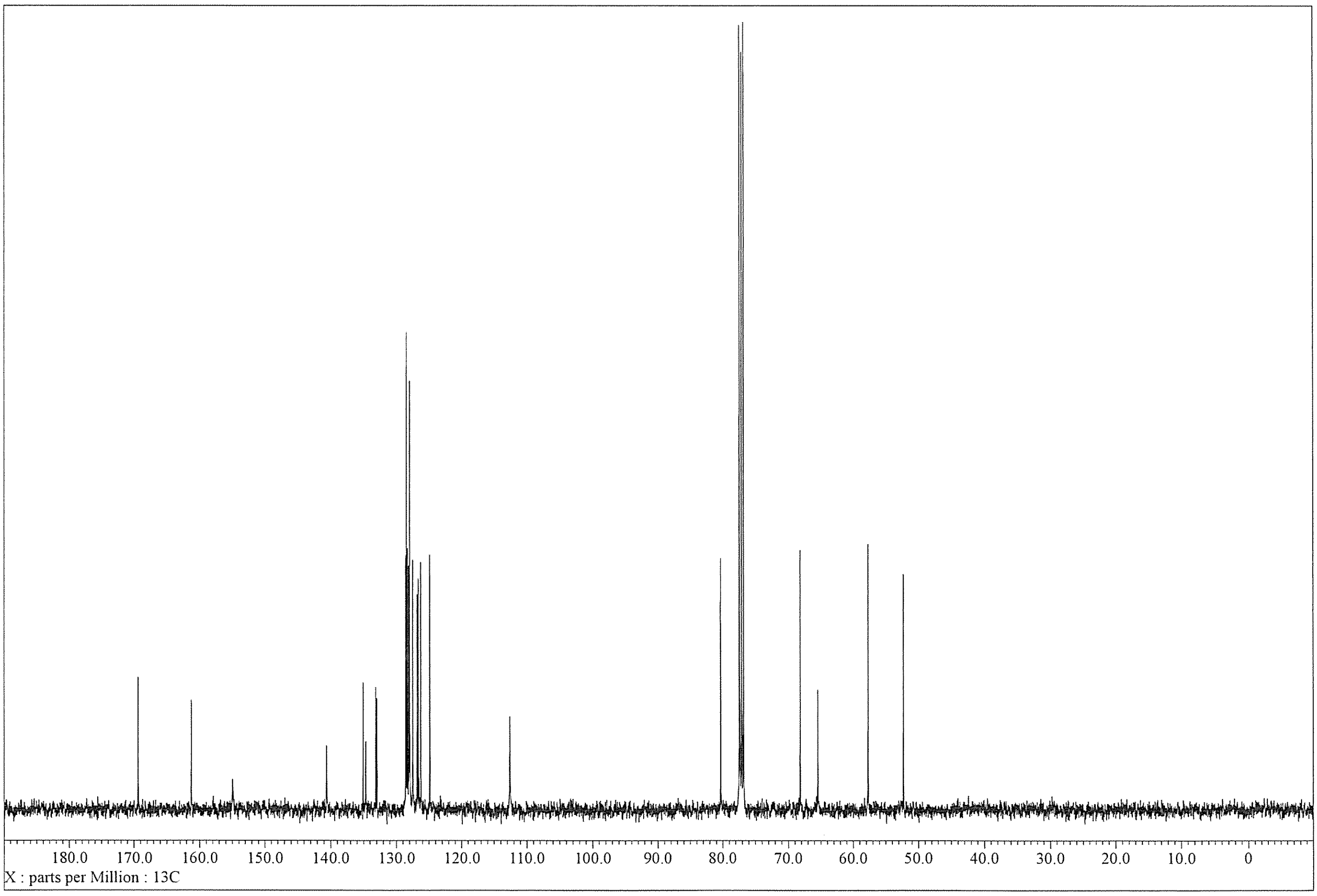

^{13}C NMR, 100 MHz, $CDCl_3$

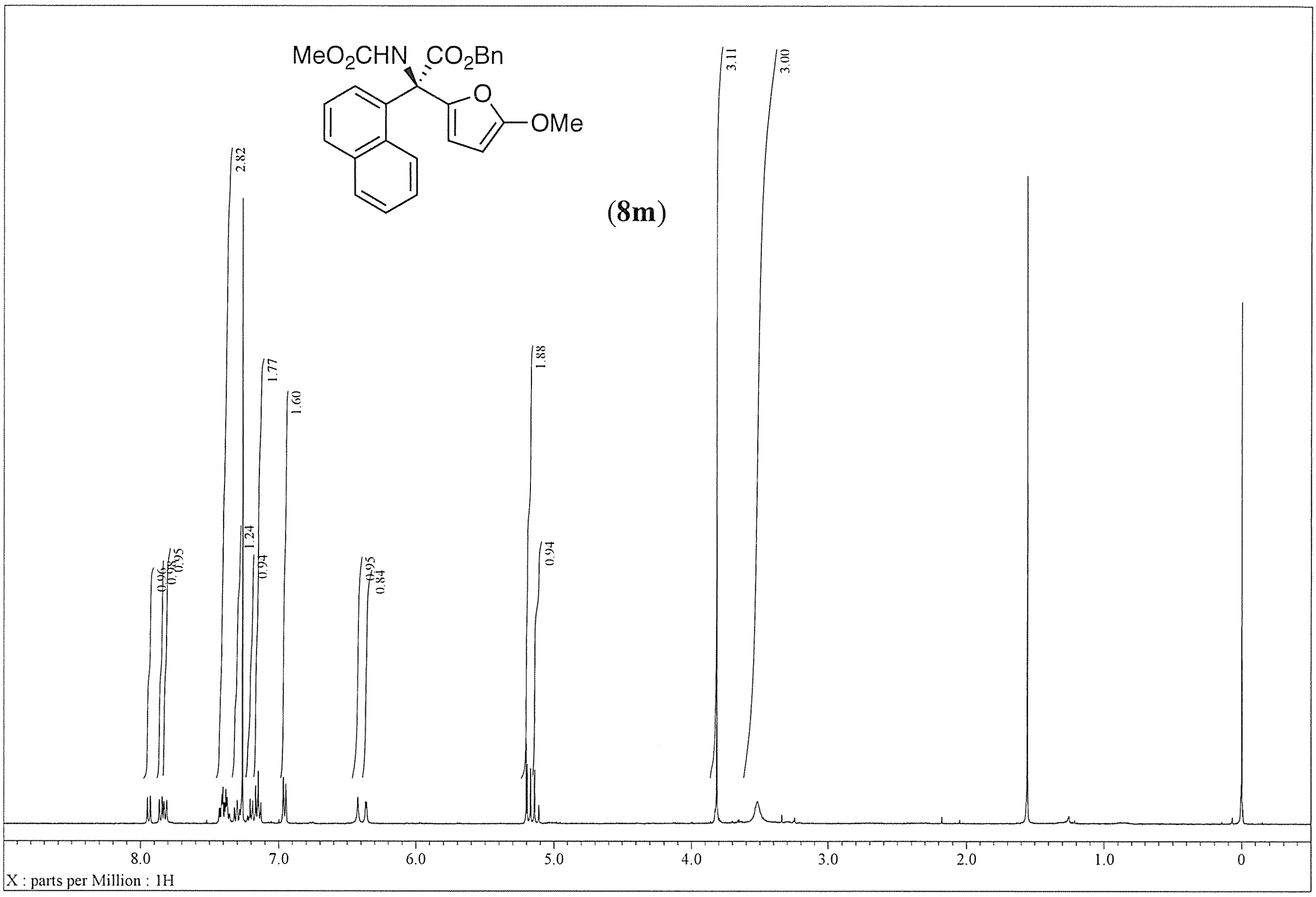

^{1}H NMR, 400 MHz, $CDCl_3$

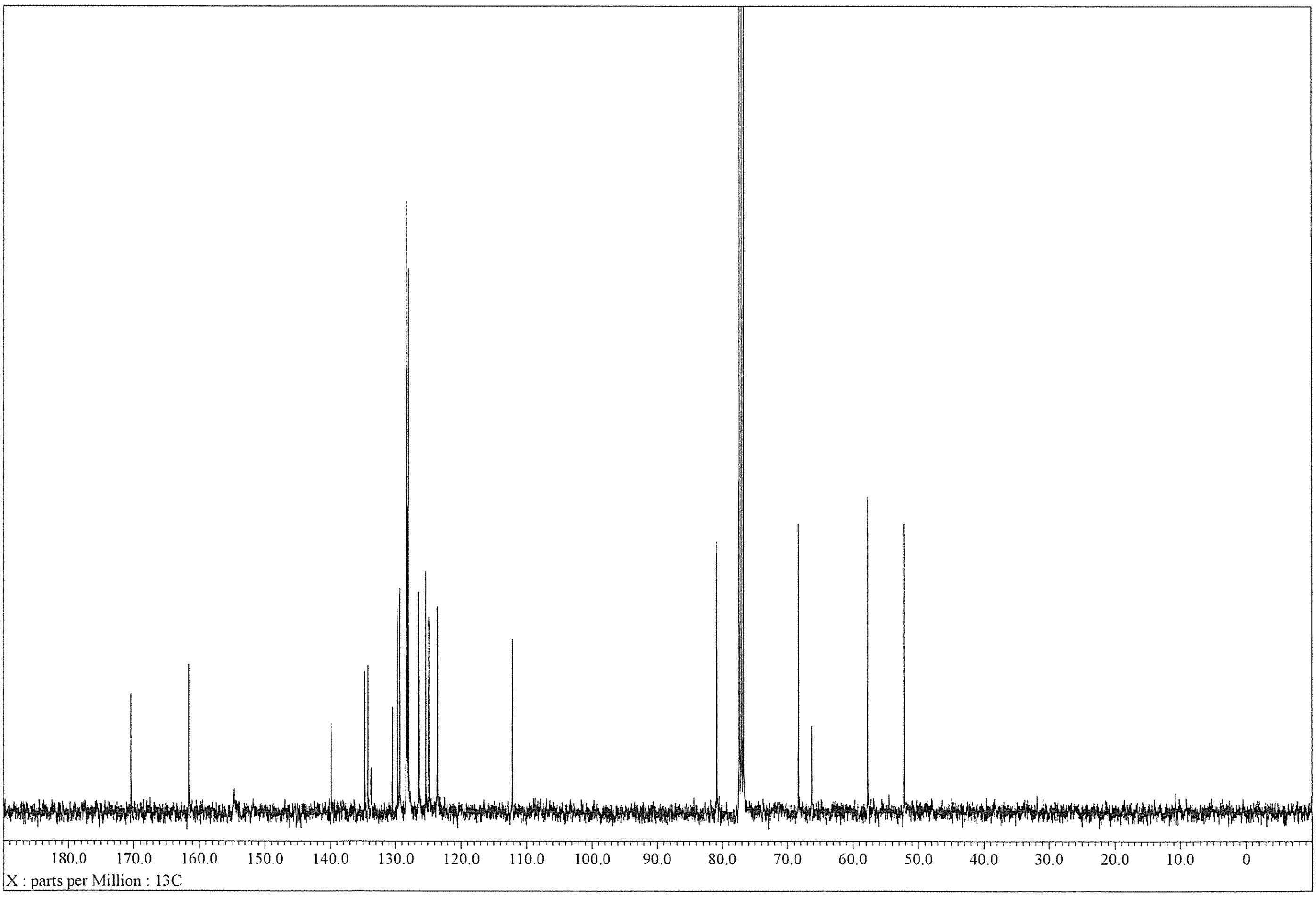

^{13}C NMR, 100 MHz, $CDCl_3$

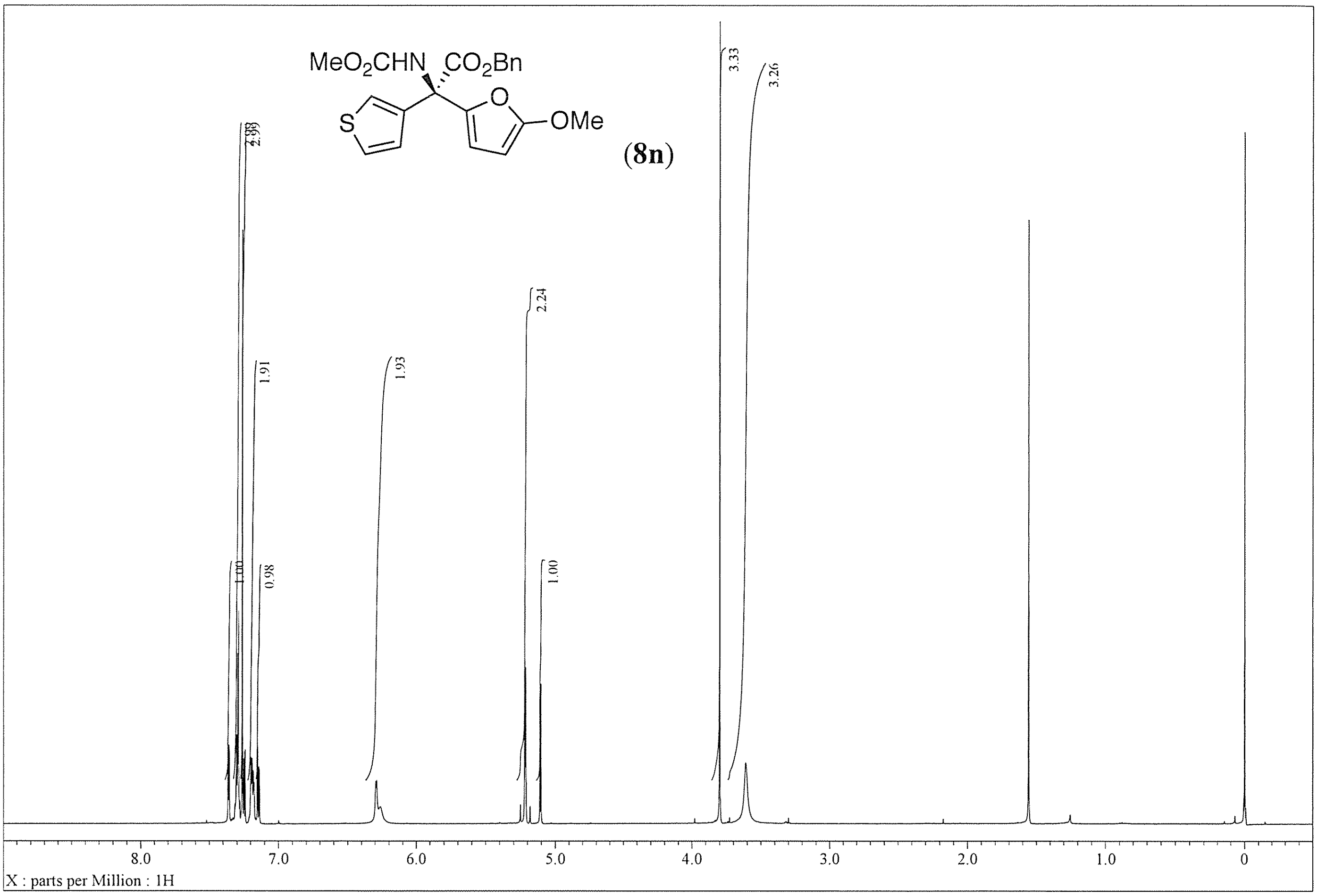

^{1}H NMR, 400 MHz, $CDCl_3$

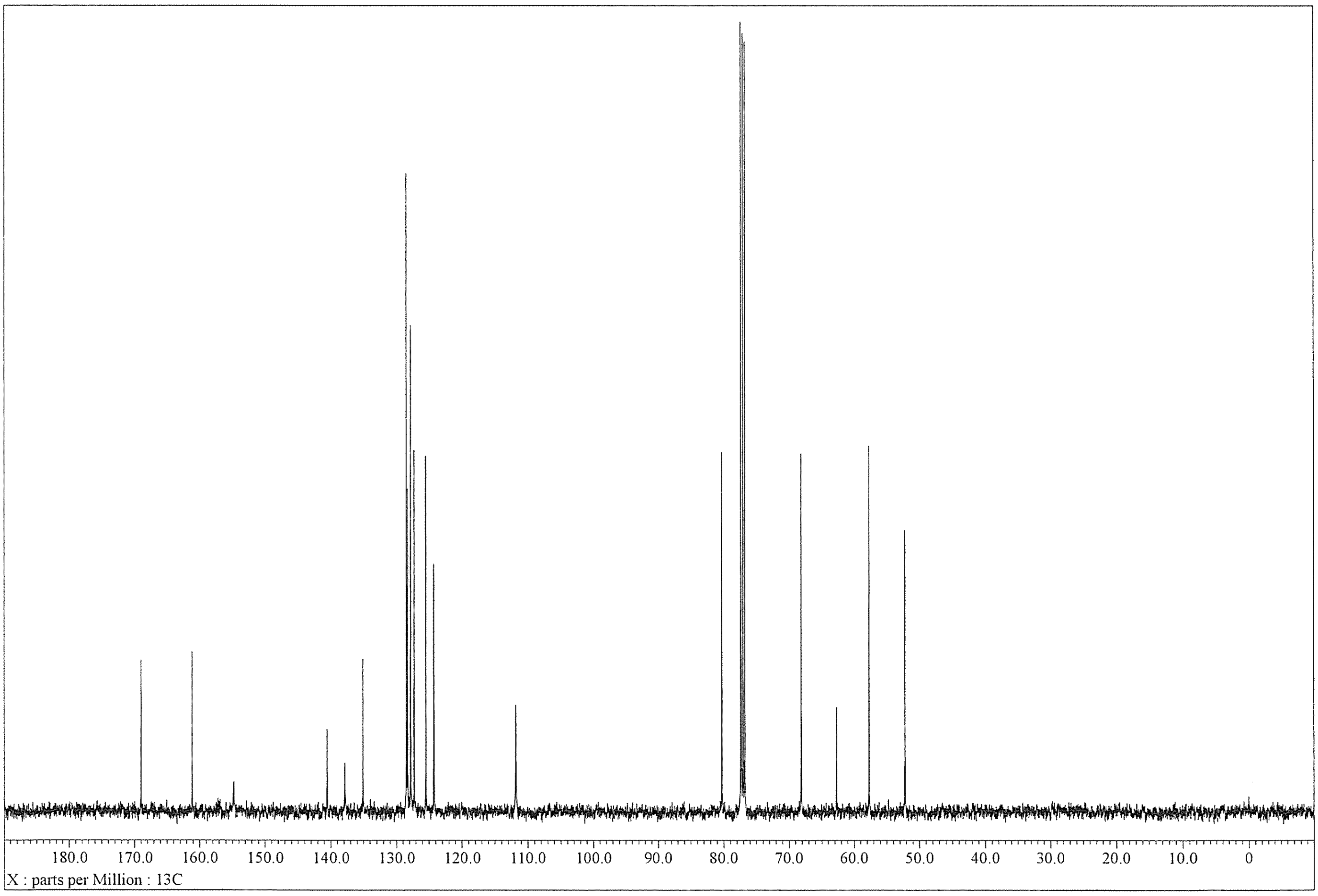

^{13}C NMR, 100 MHz, $CDCl_3$

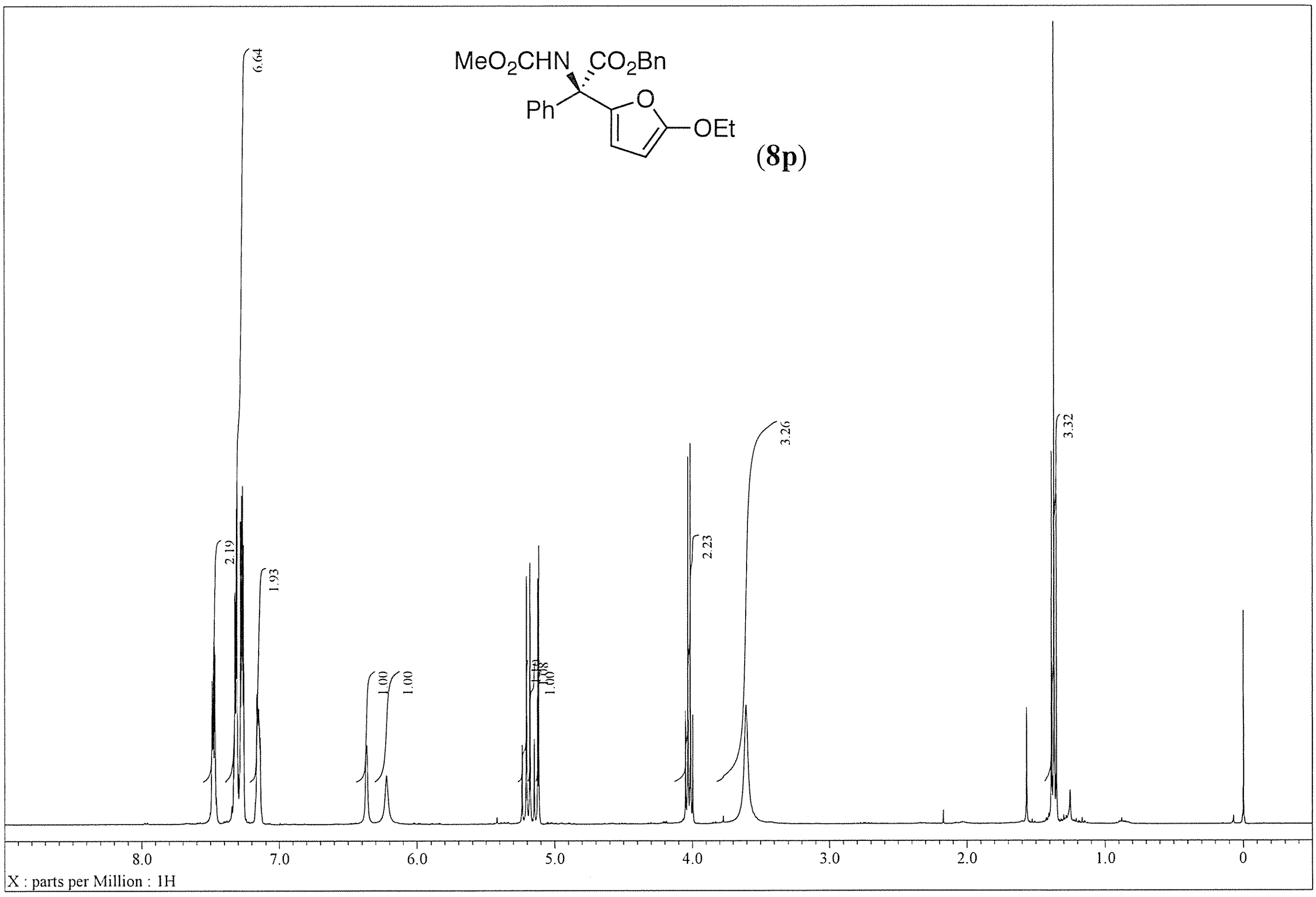

^{1}H NMR, 400 MHz, $CDCl_3$

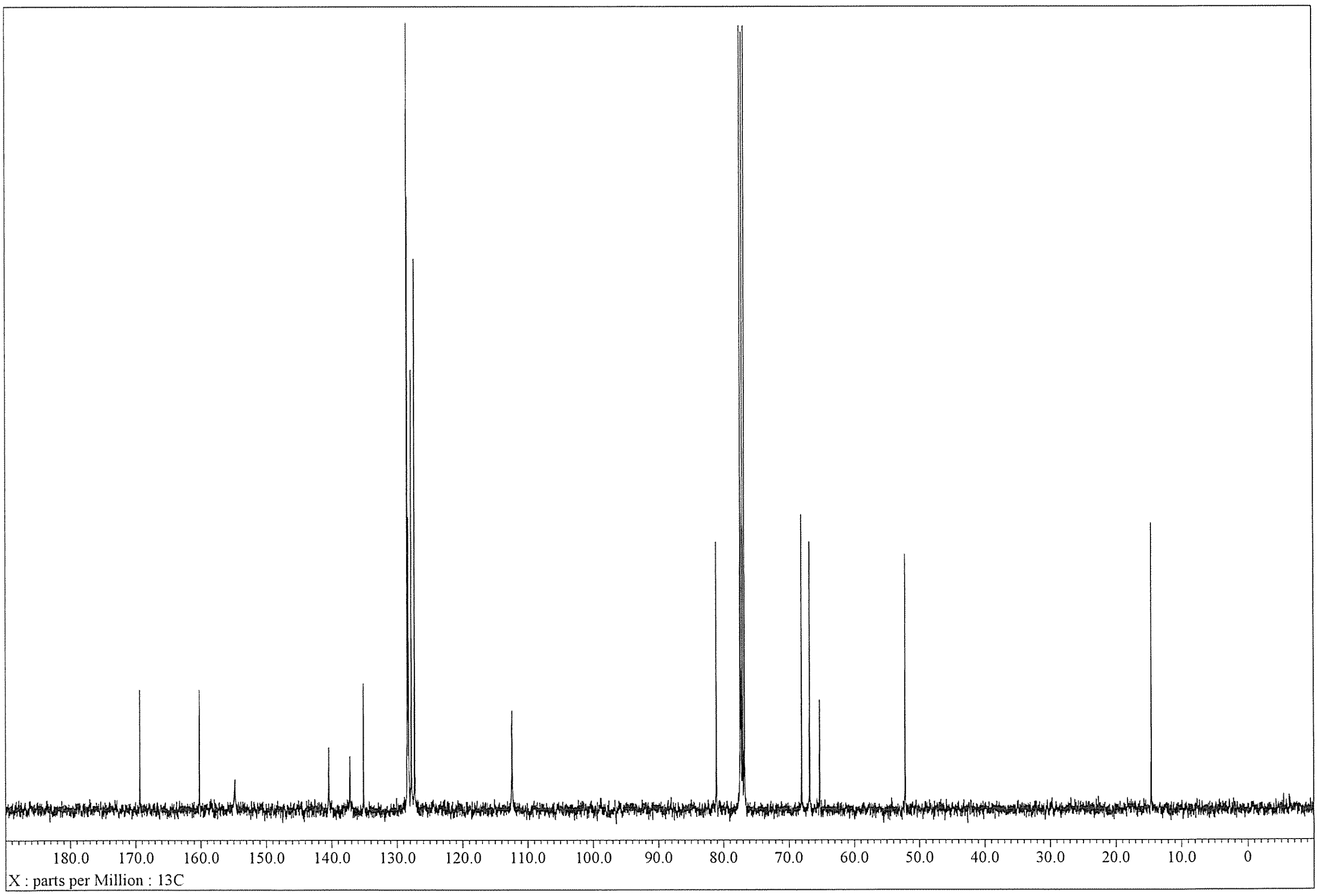

^{13}C NMR, 100 MHz, $CDCl_3$

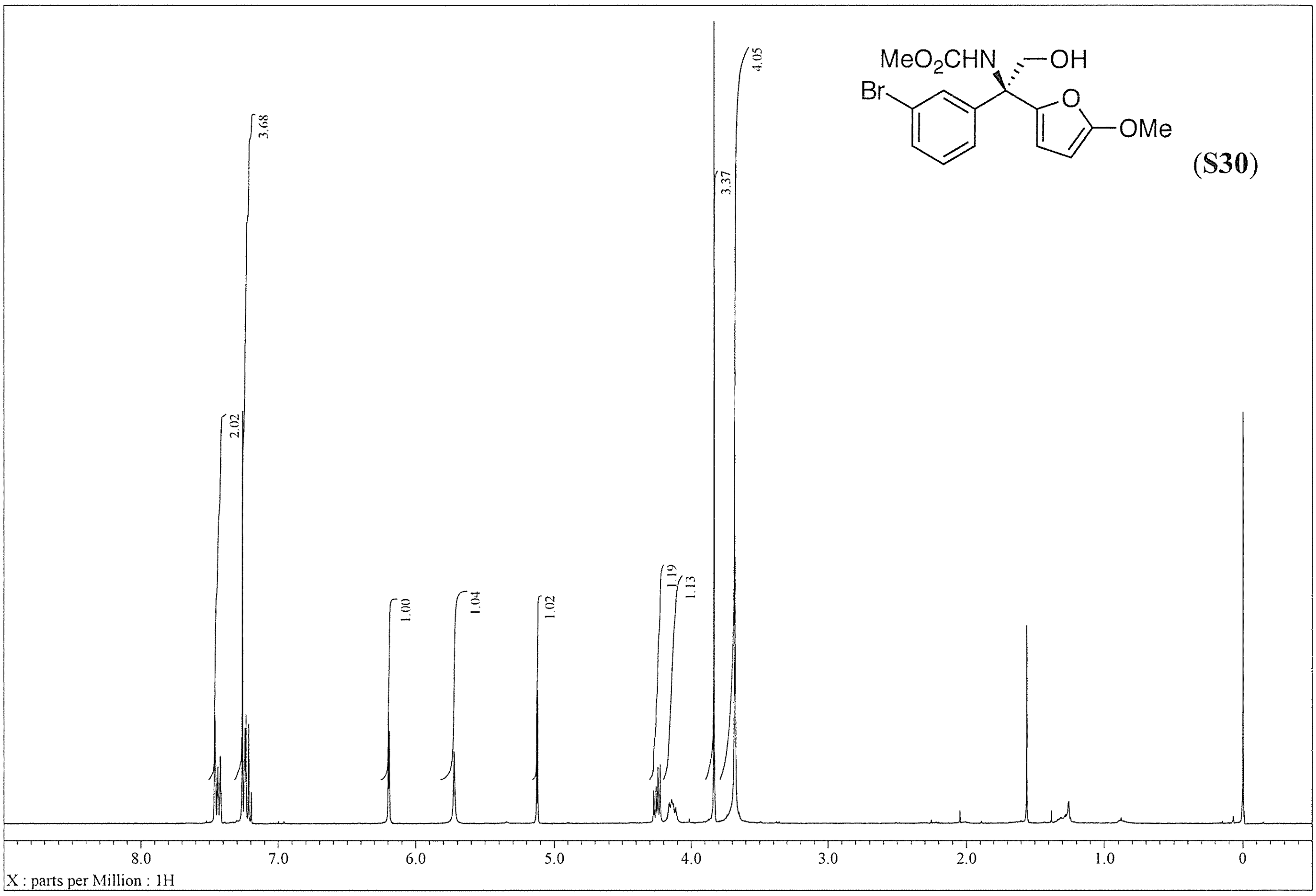

^{1}H NMR, 400 MHz, $CDCl_3$

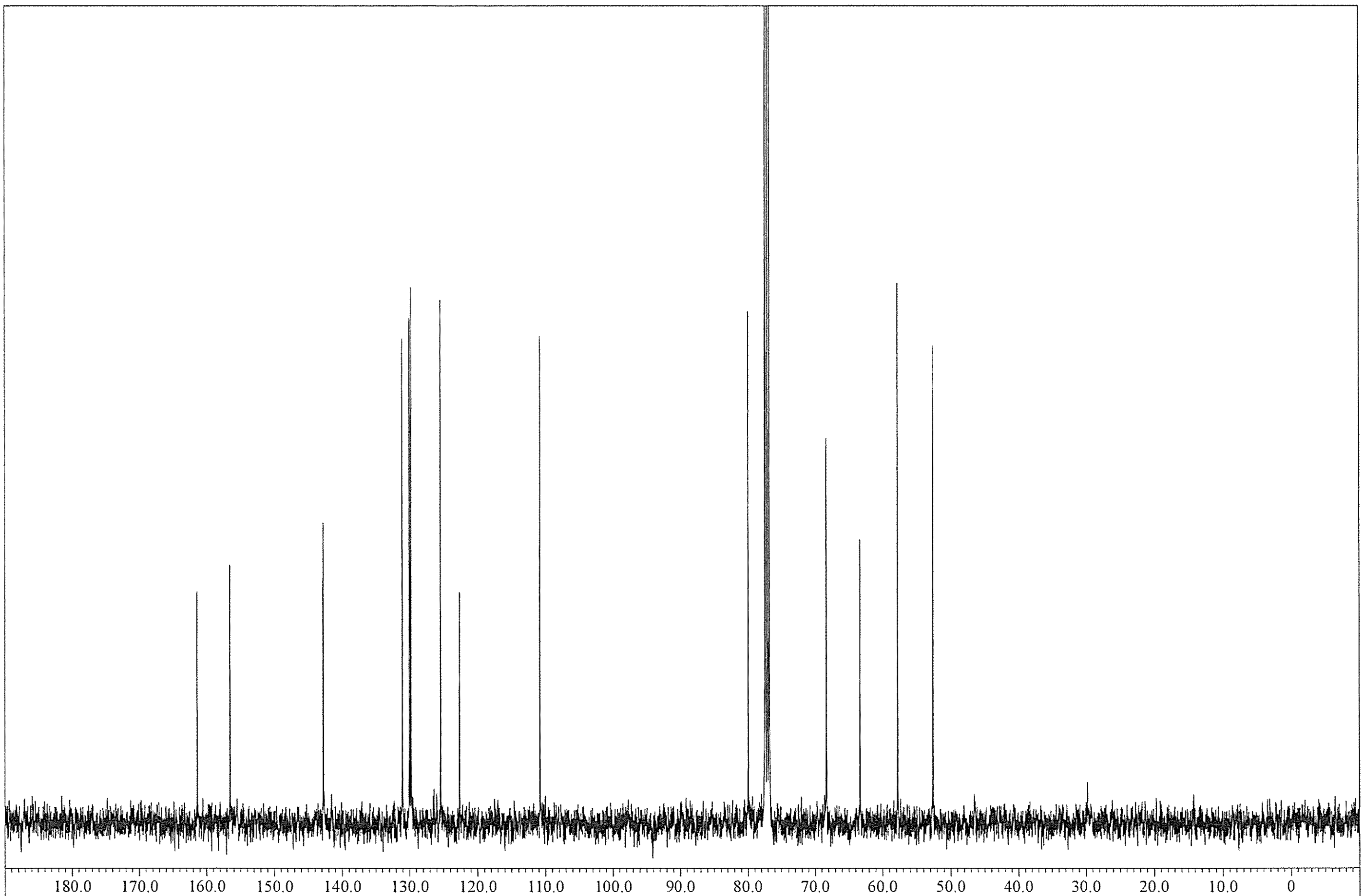

^{13}C NMR, 100 MHz, $CDCl_3$

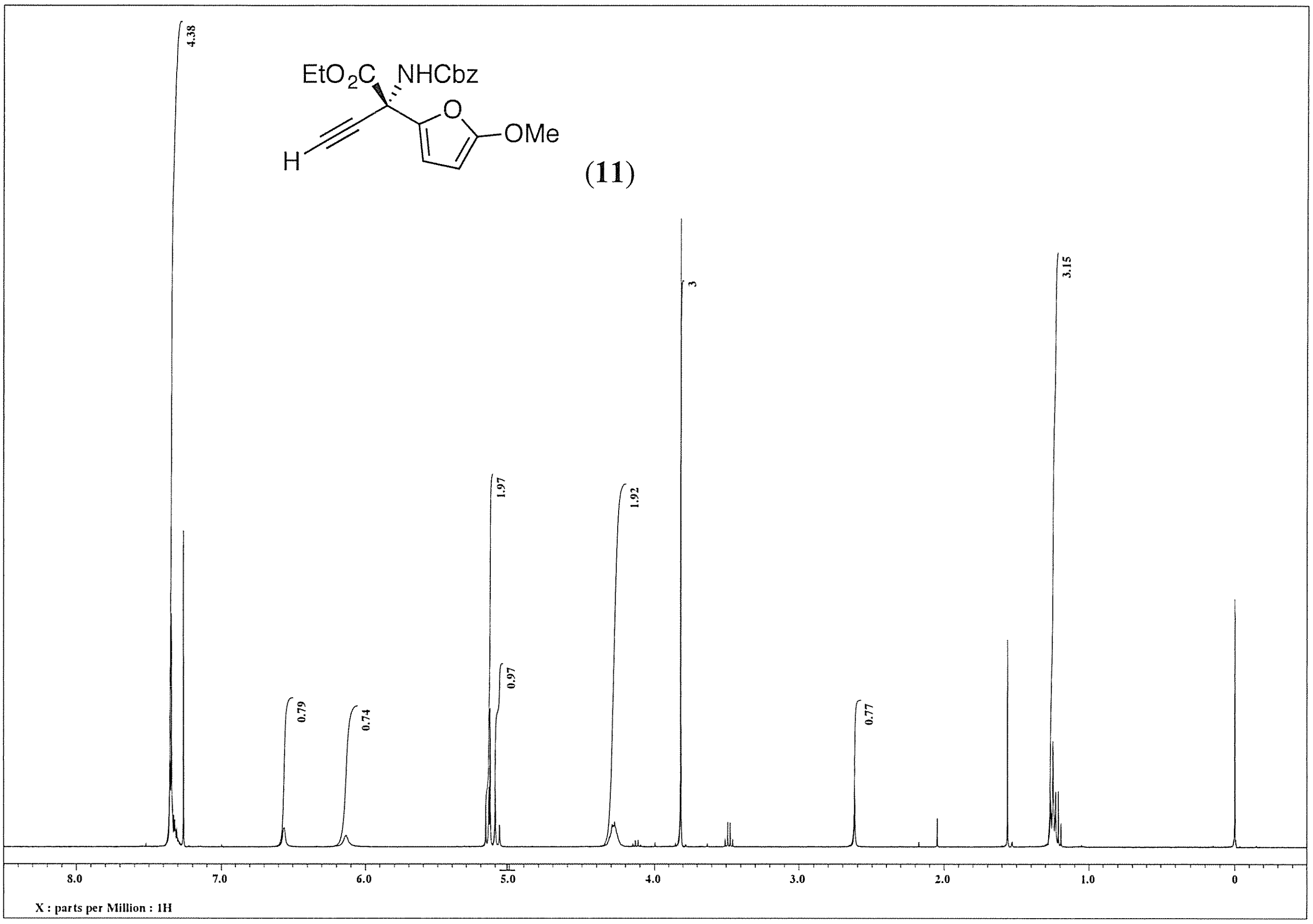

^{1}H NMR, 400 MHz, $CDCl_3$

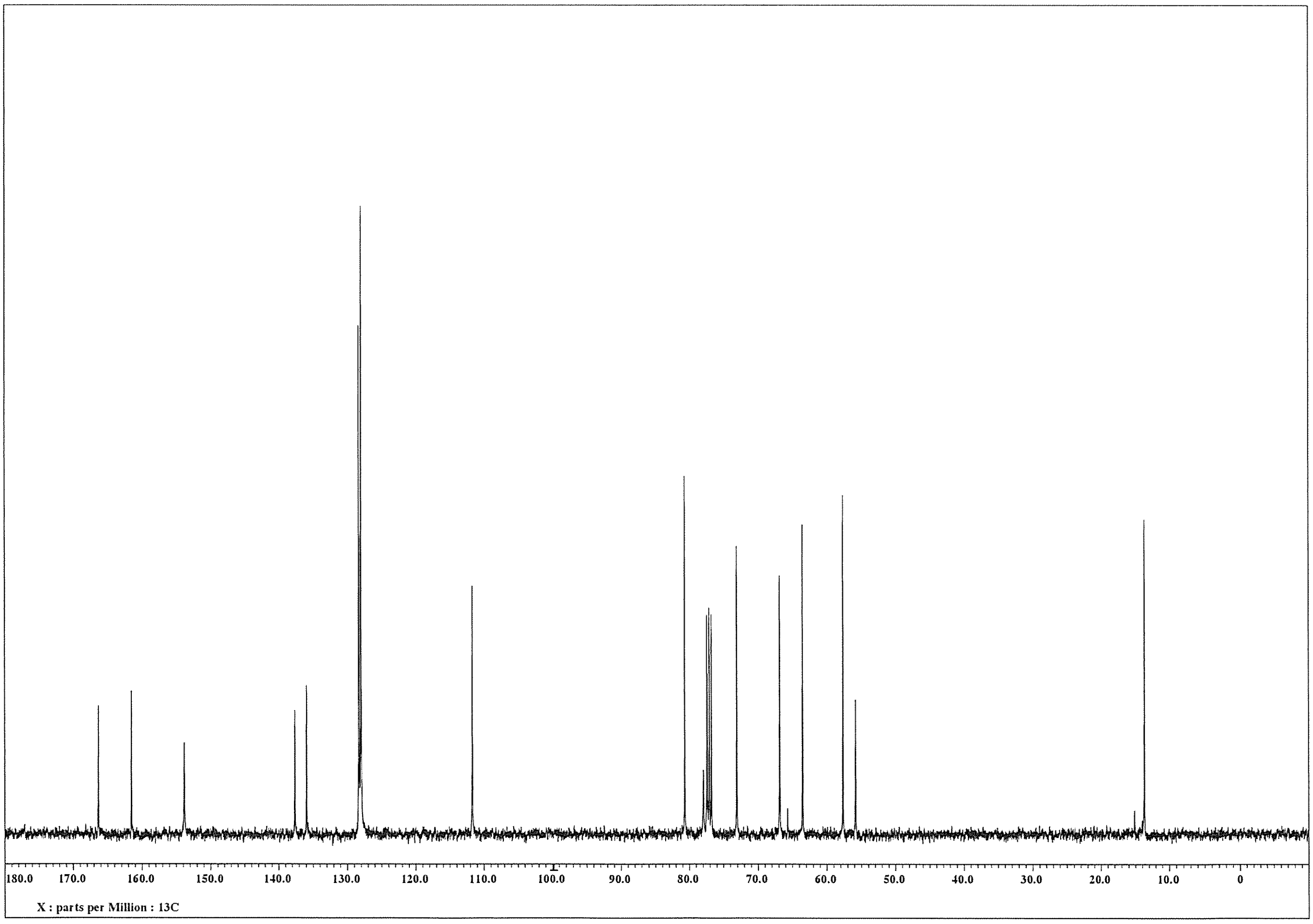

^{13}C NMR, 100 MHz, $CDCl_3$

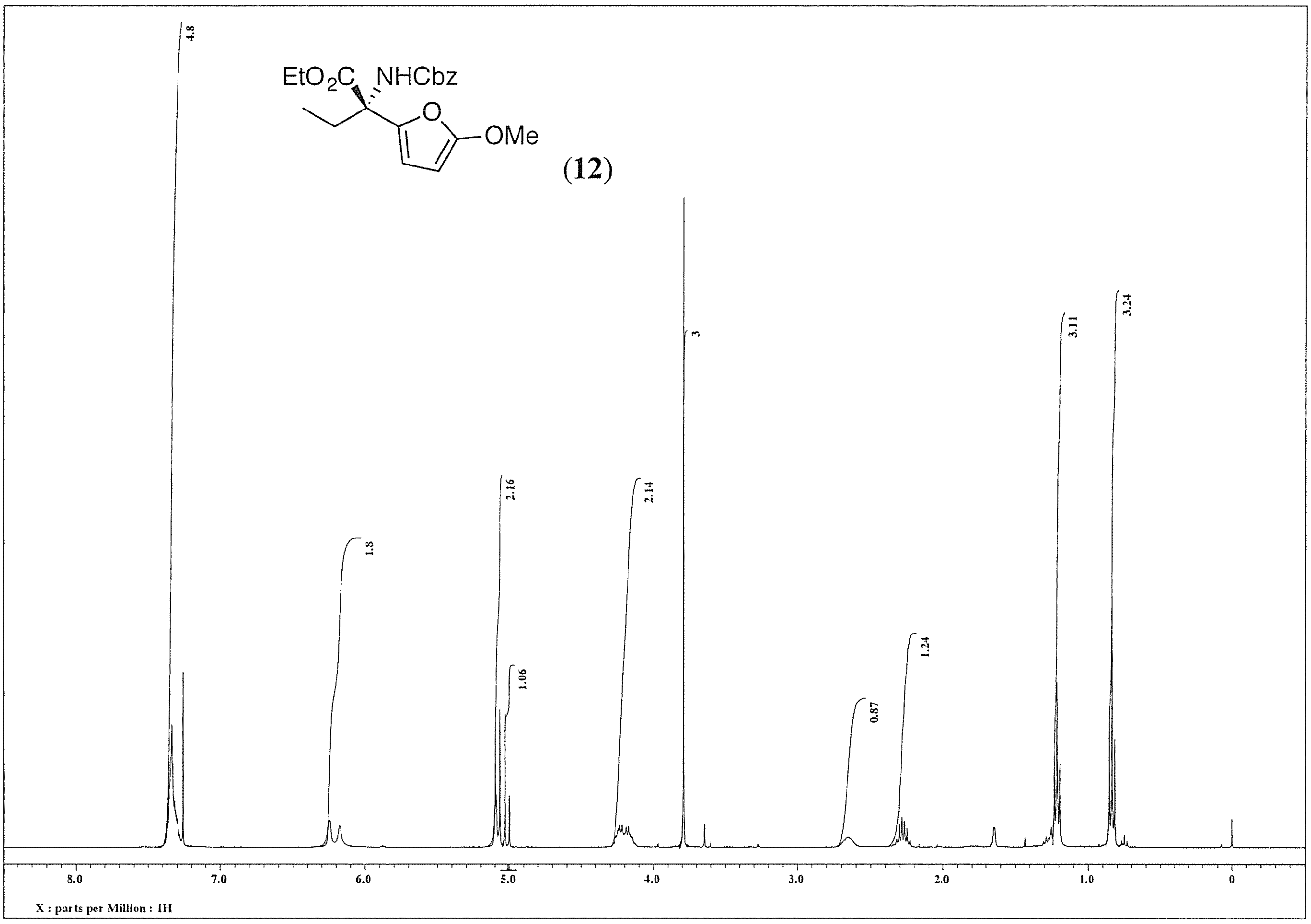

^{1}H NMR, 400 MHz, $CDCl_3$

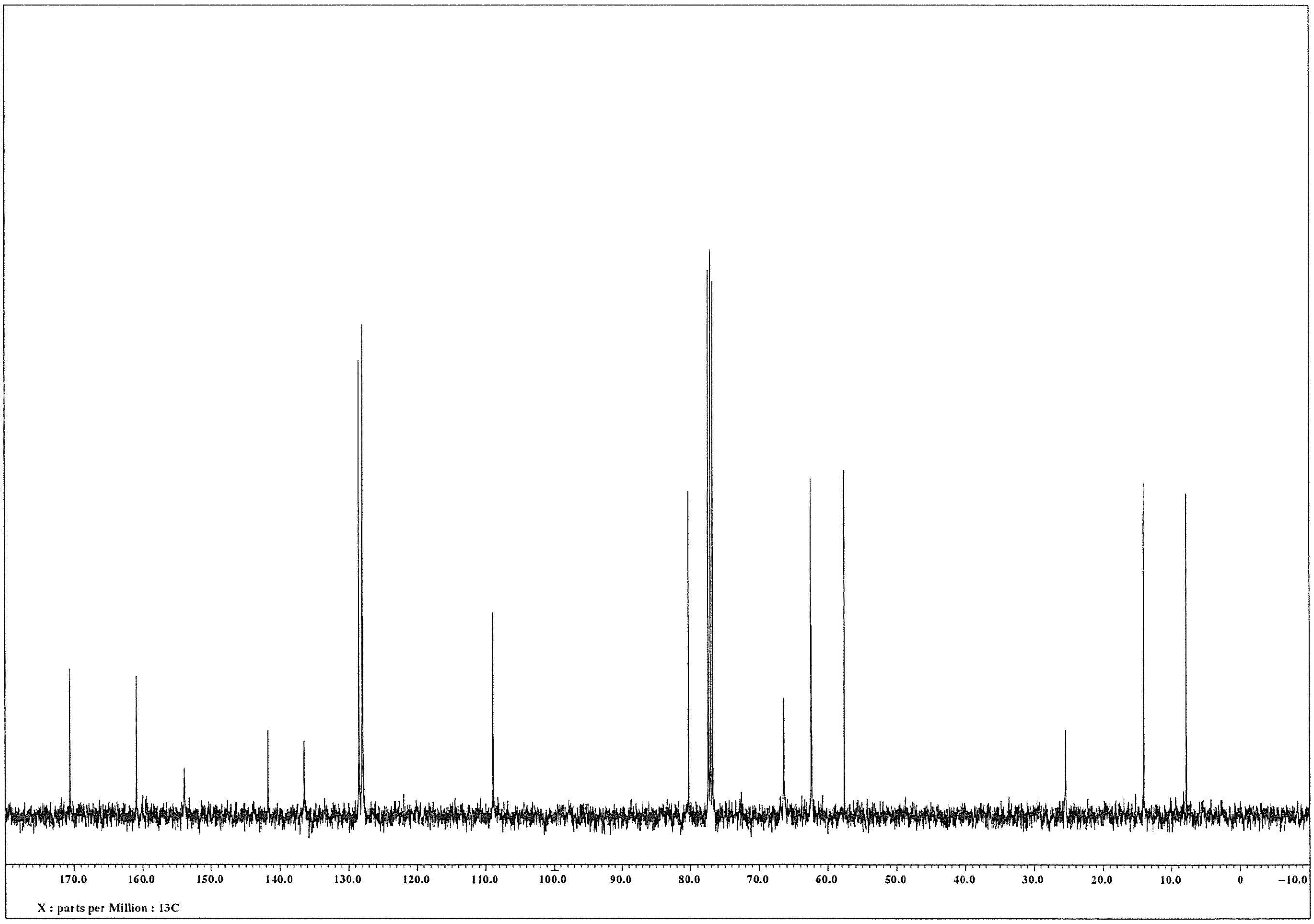

^{13}C NMR, 100 MHz, $CDCl_3$

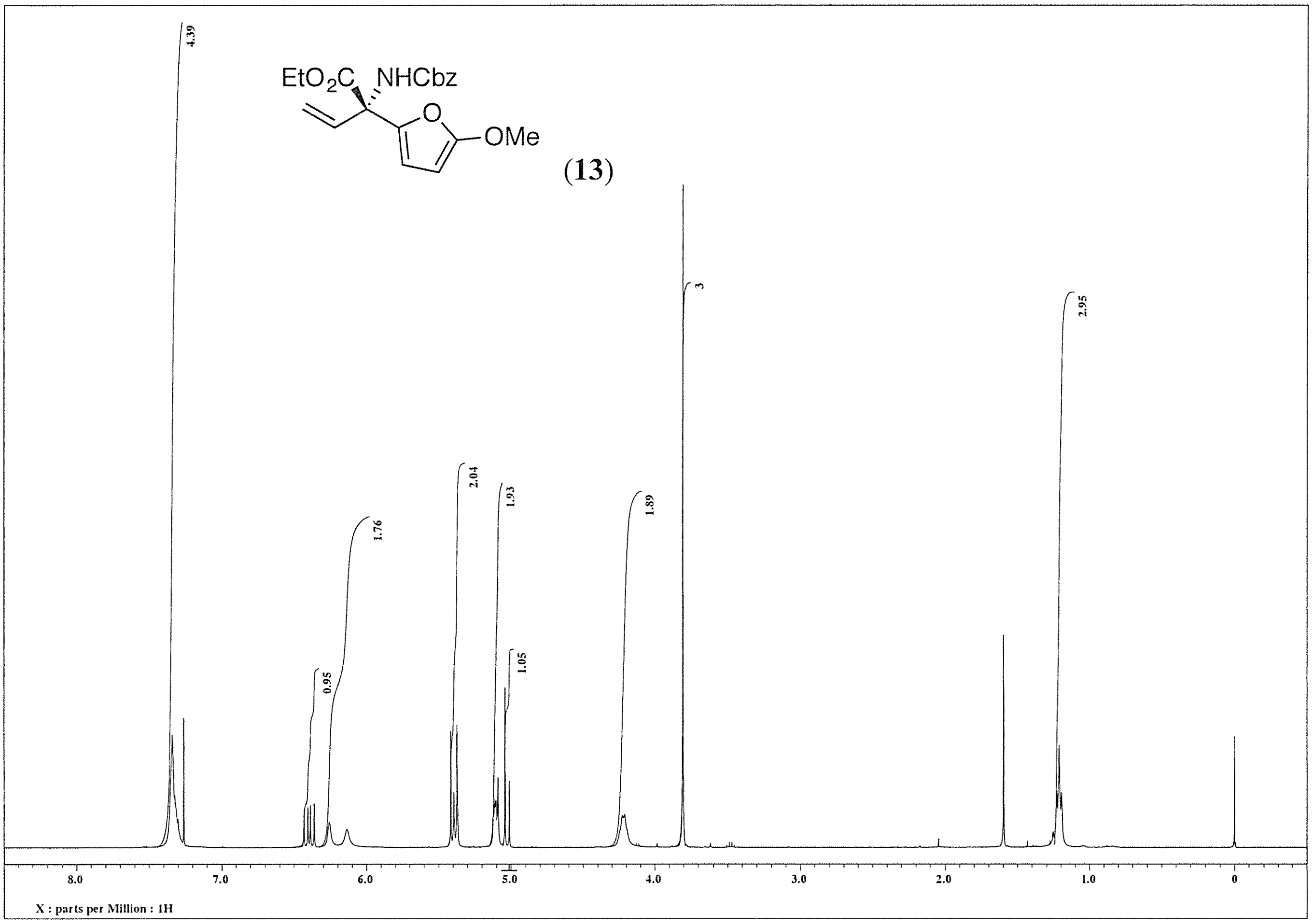

^{1}H NMR, 400 MHz, $CDCl_3$

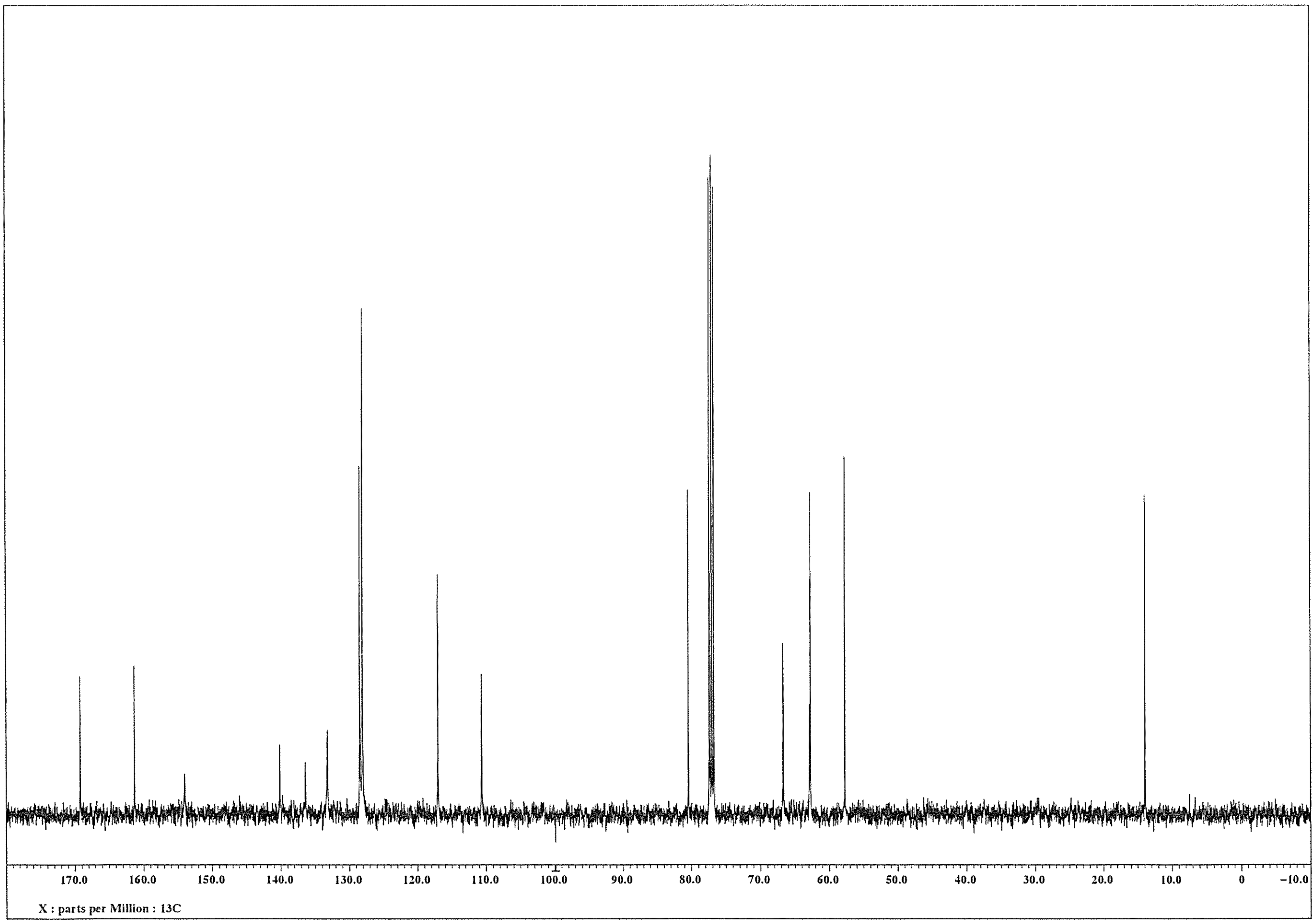

^{13}C NMR, 100 MHz, $CDCl_3$

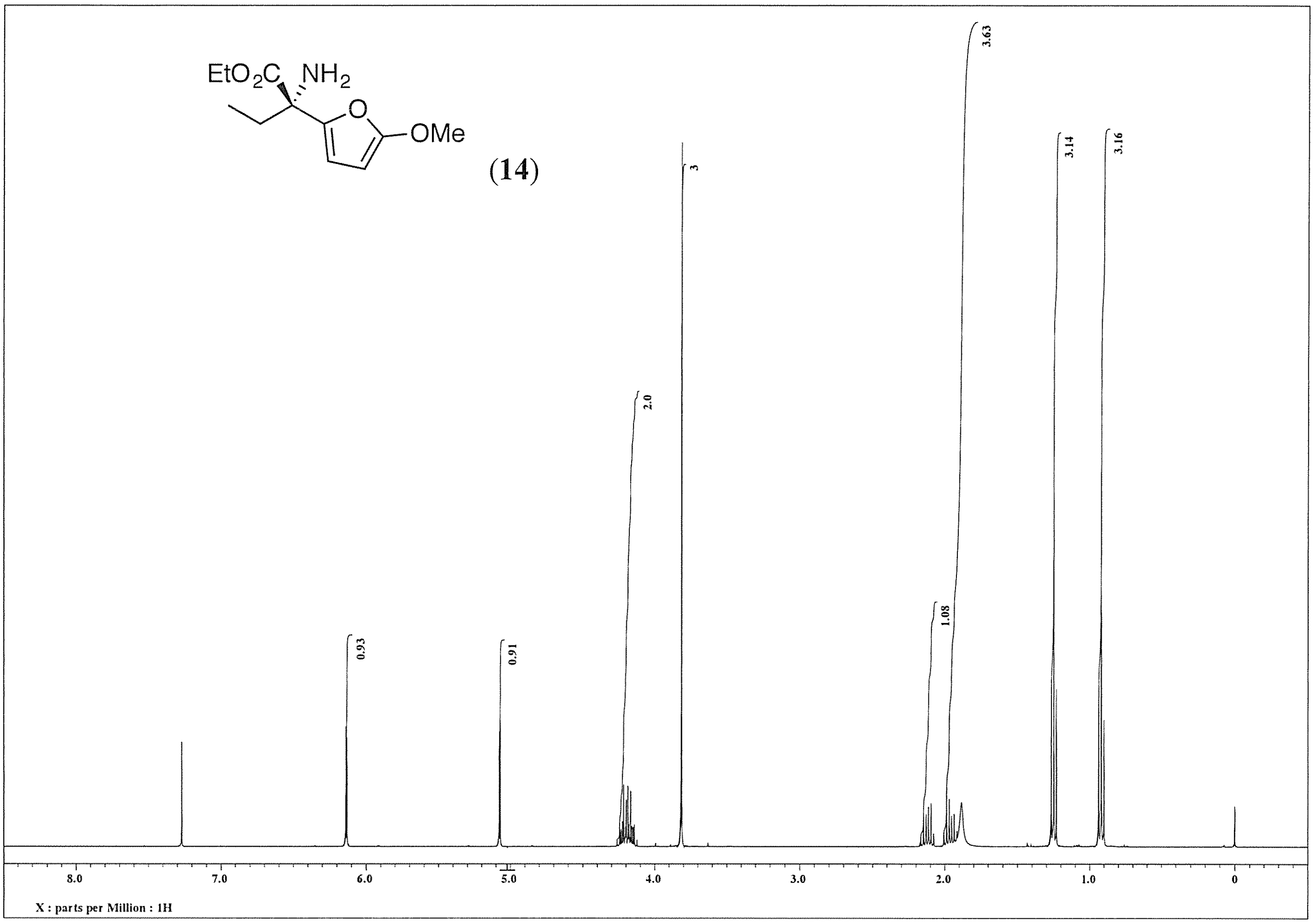

^{1}H NMR, 400 MHz, $CDCl_3$

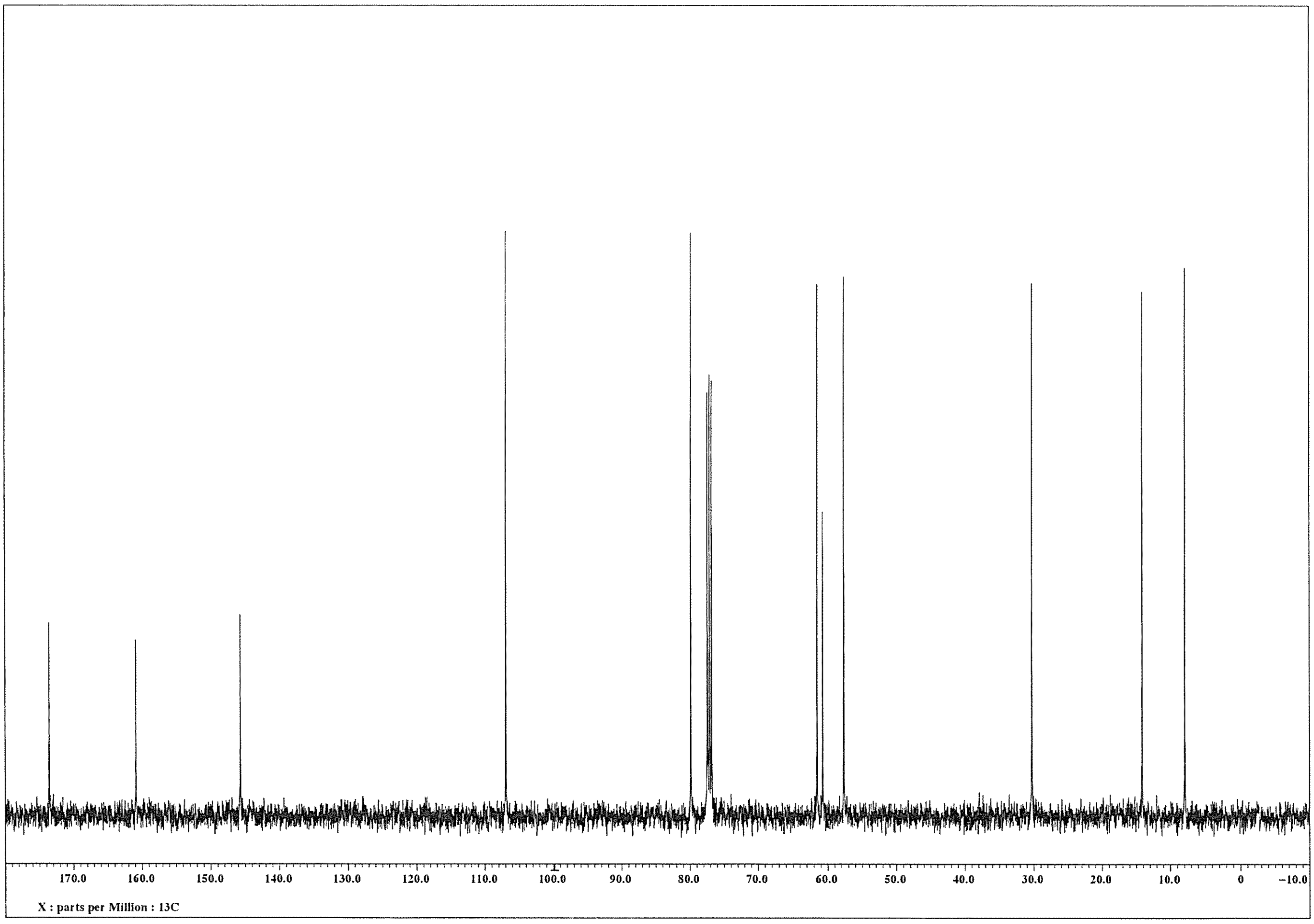

^{13}C NMR, 100 MHz, $CDCl_3$

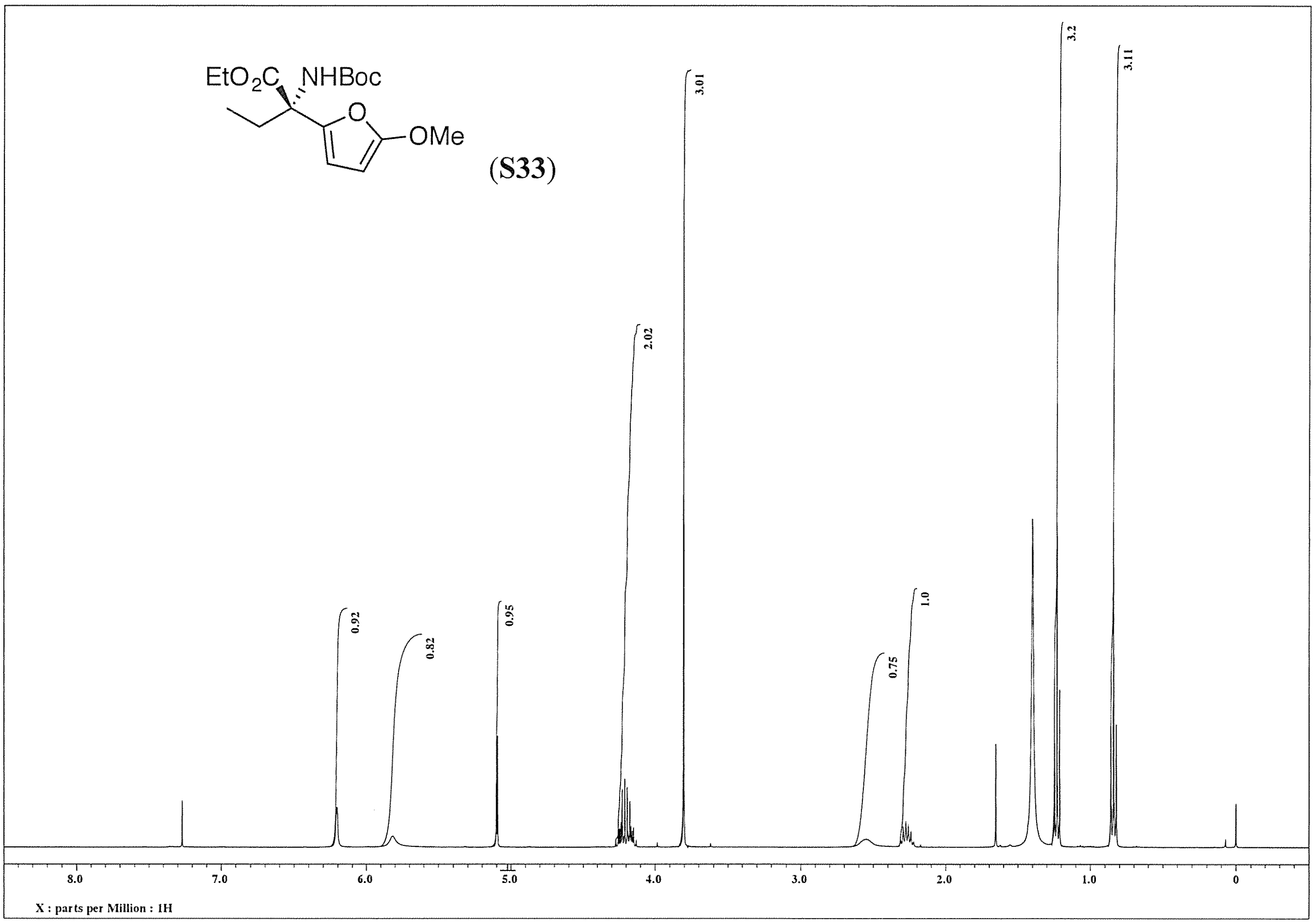

^{1}H NMR, 400 MHz, $CDCl_3$

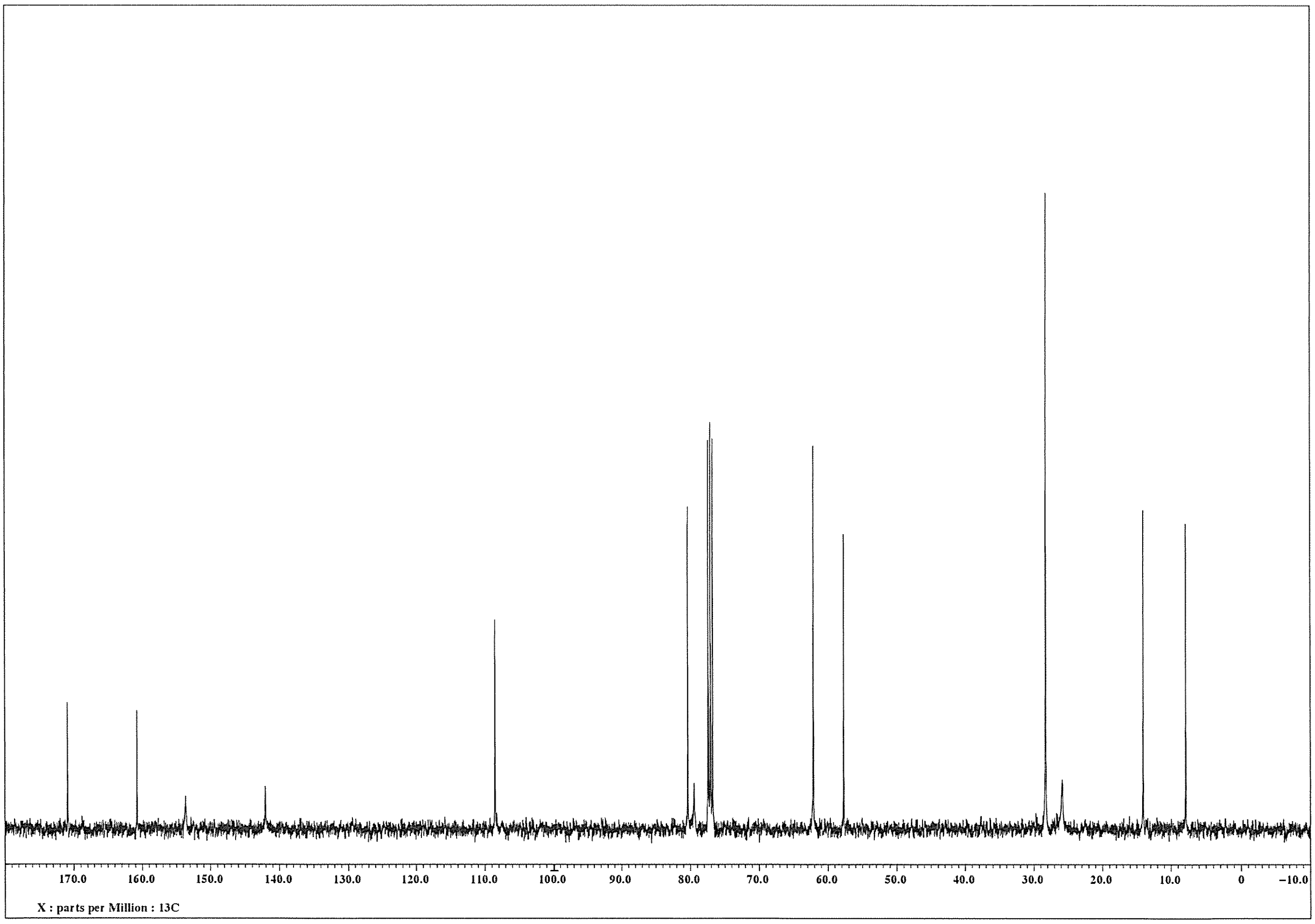

^{13}C NMR, 100 MHz, $CDCl_3$

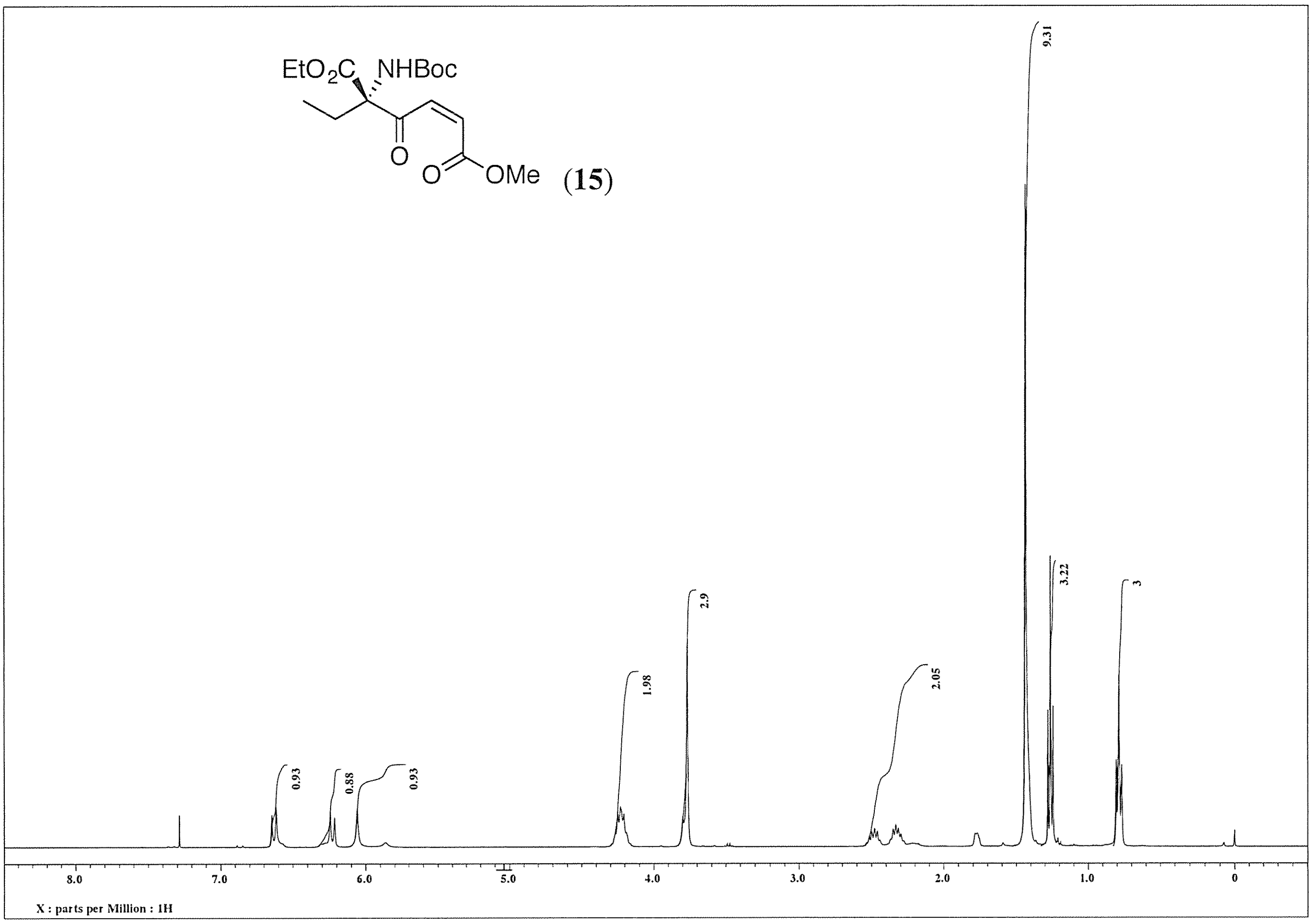

^{1}H NMR, 400 MHz, $CDCl_3$

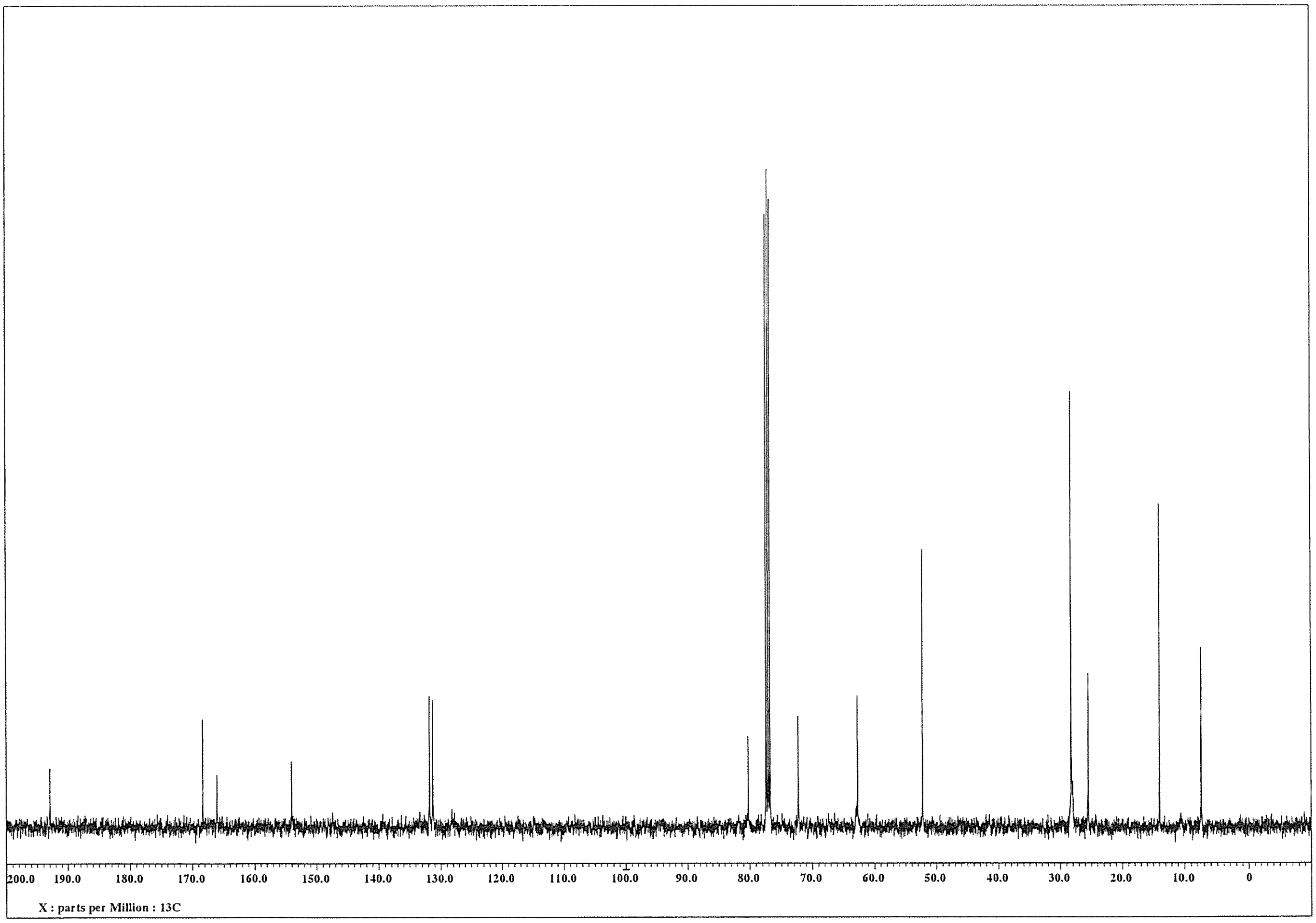

^{13}C NMR, 100 MHz, $CDCl_3$

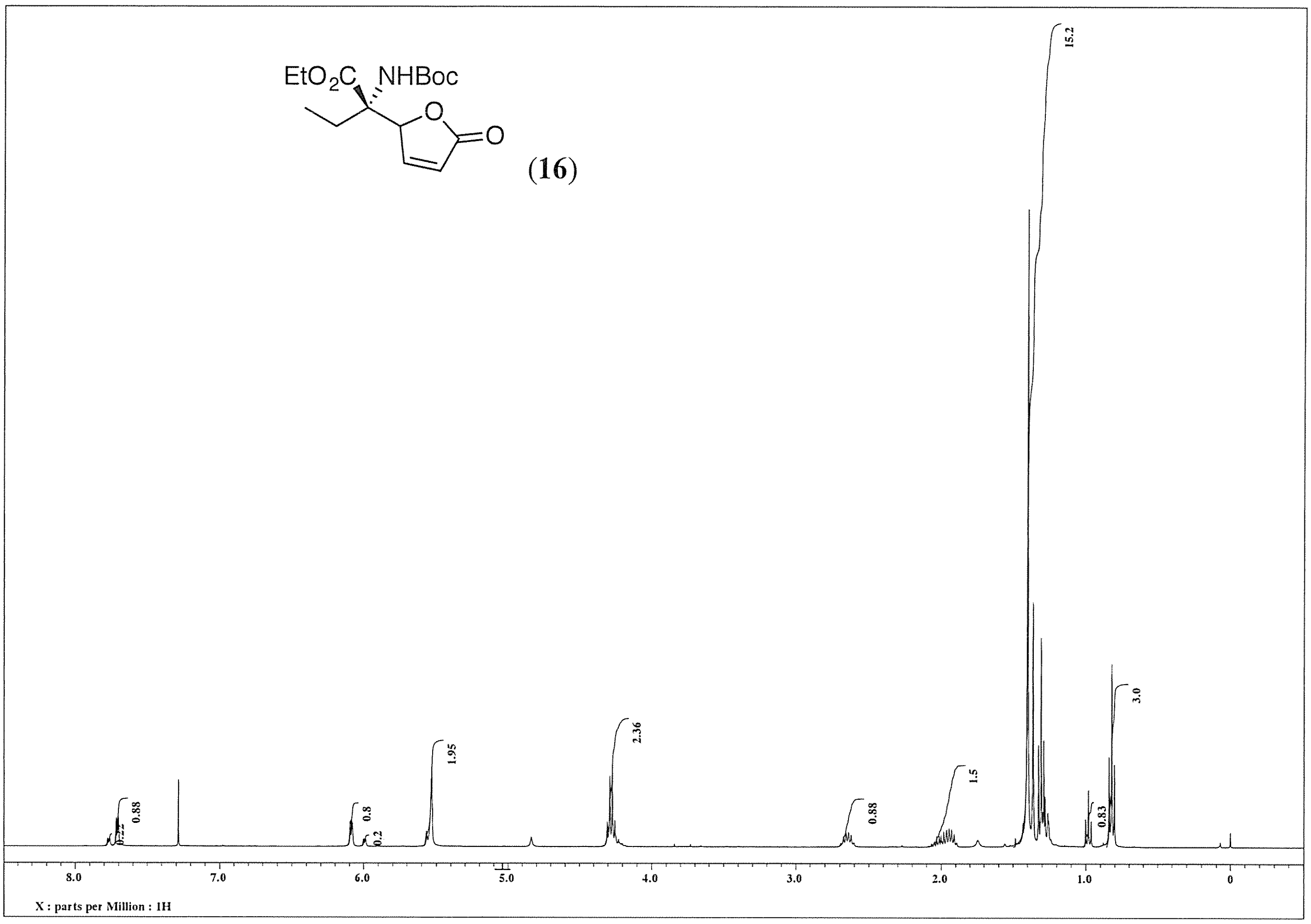

^{1}H NMR, 400 MHz, $CDCl_3$

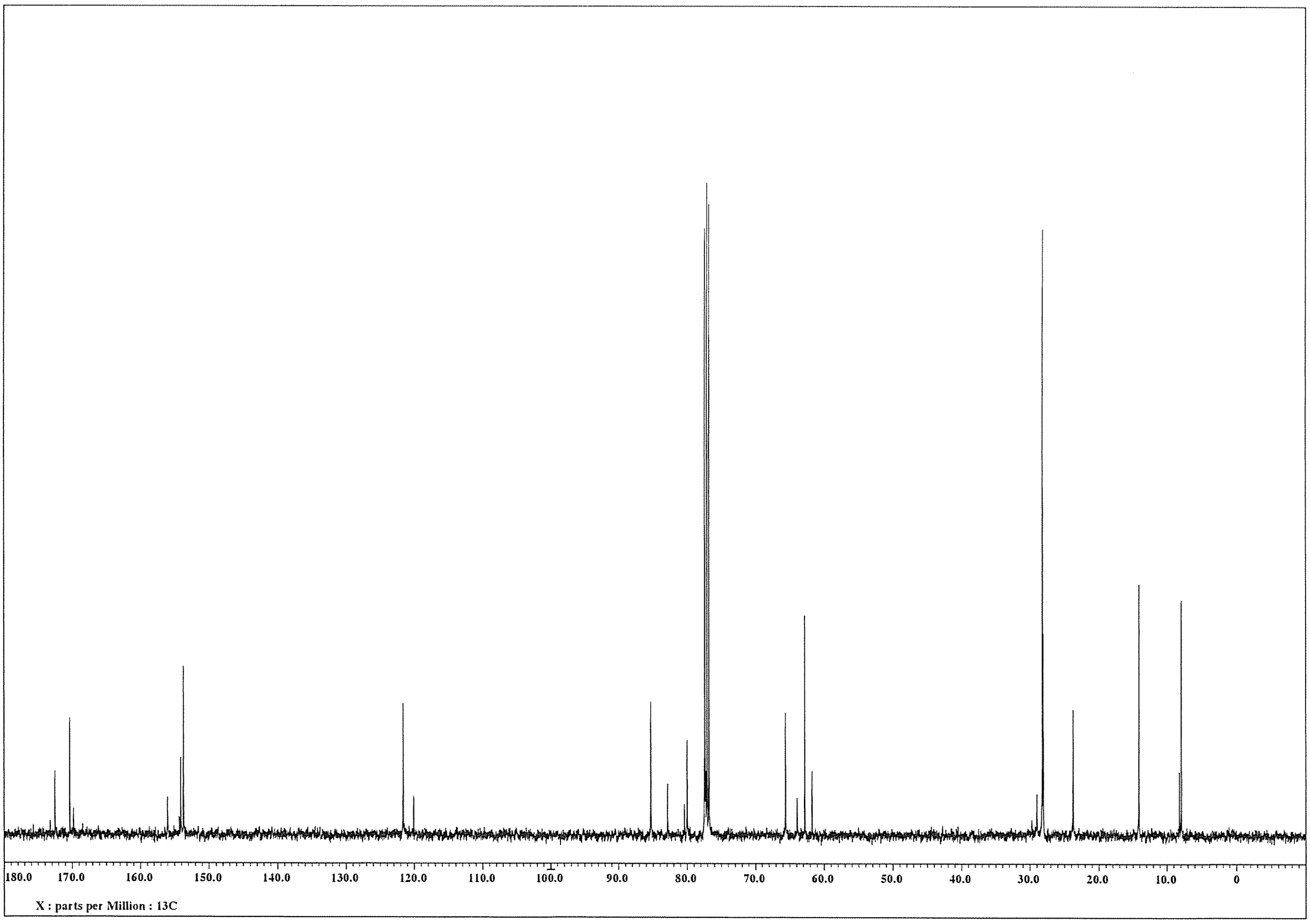

^{13}C NMR, 100 MHz, $CDCl_3$

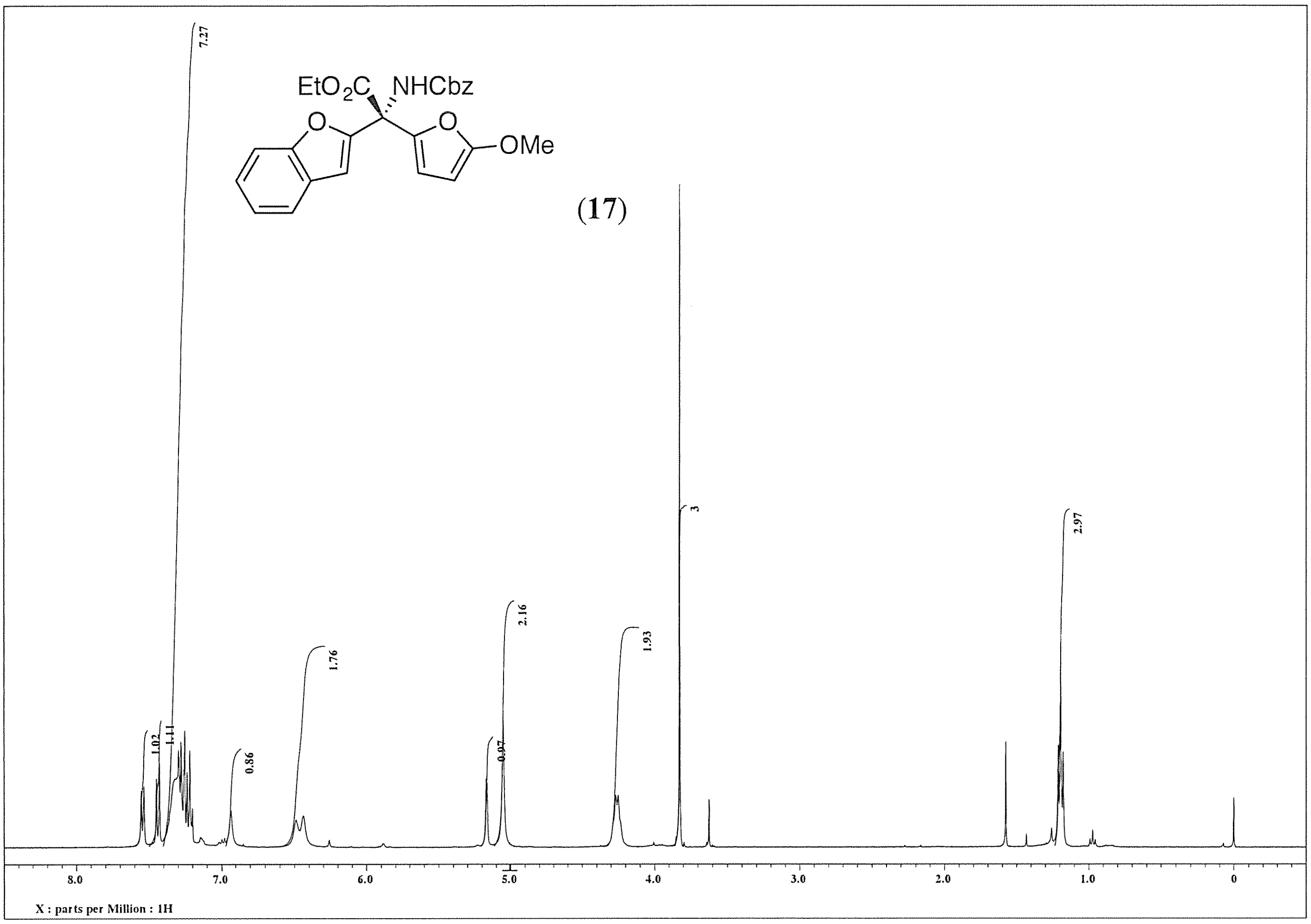

^{1}H NMR, 400 MHz, $CDCl_3$

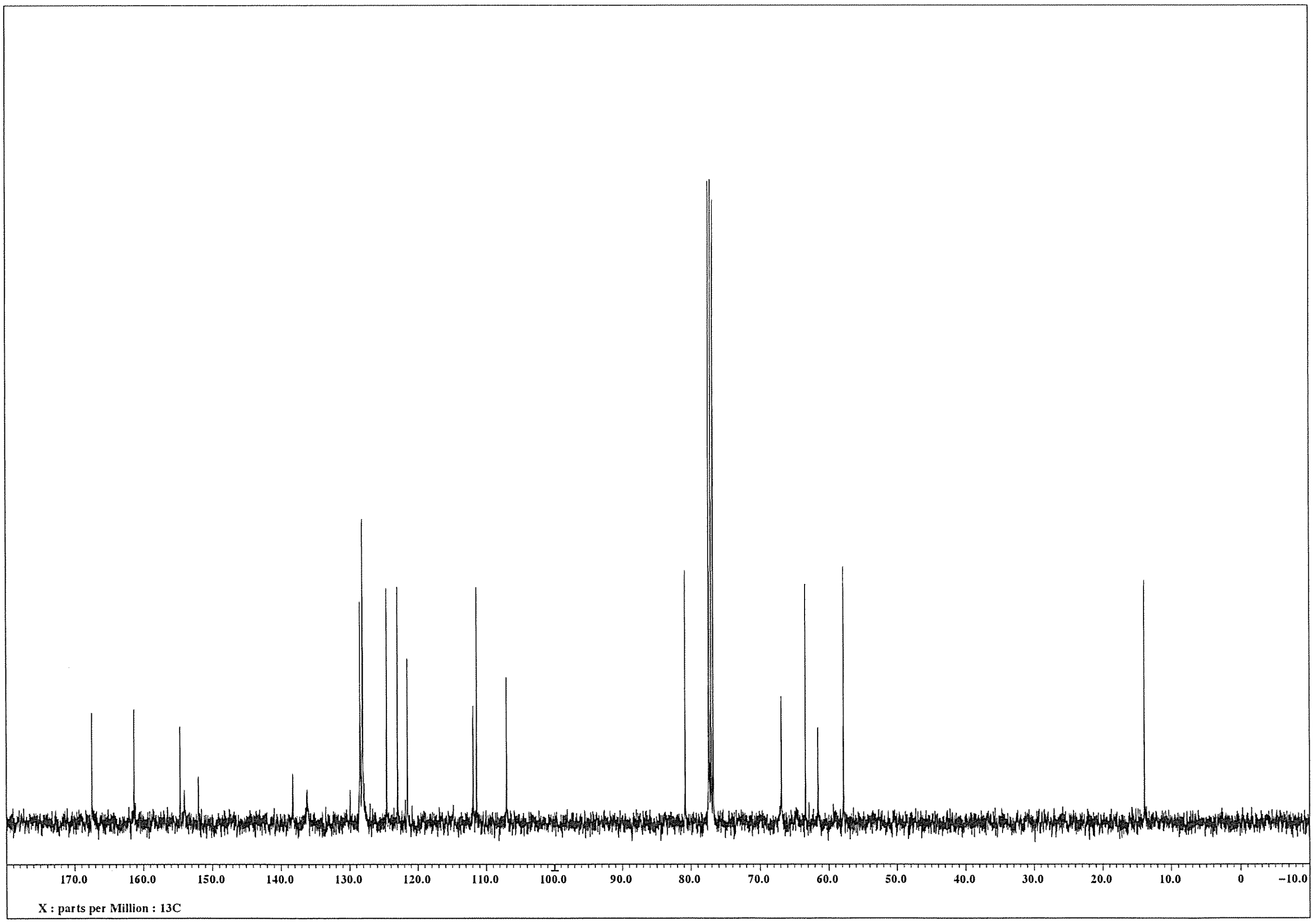

^{13}C NMR, 100 MHz, $CDCl_3$

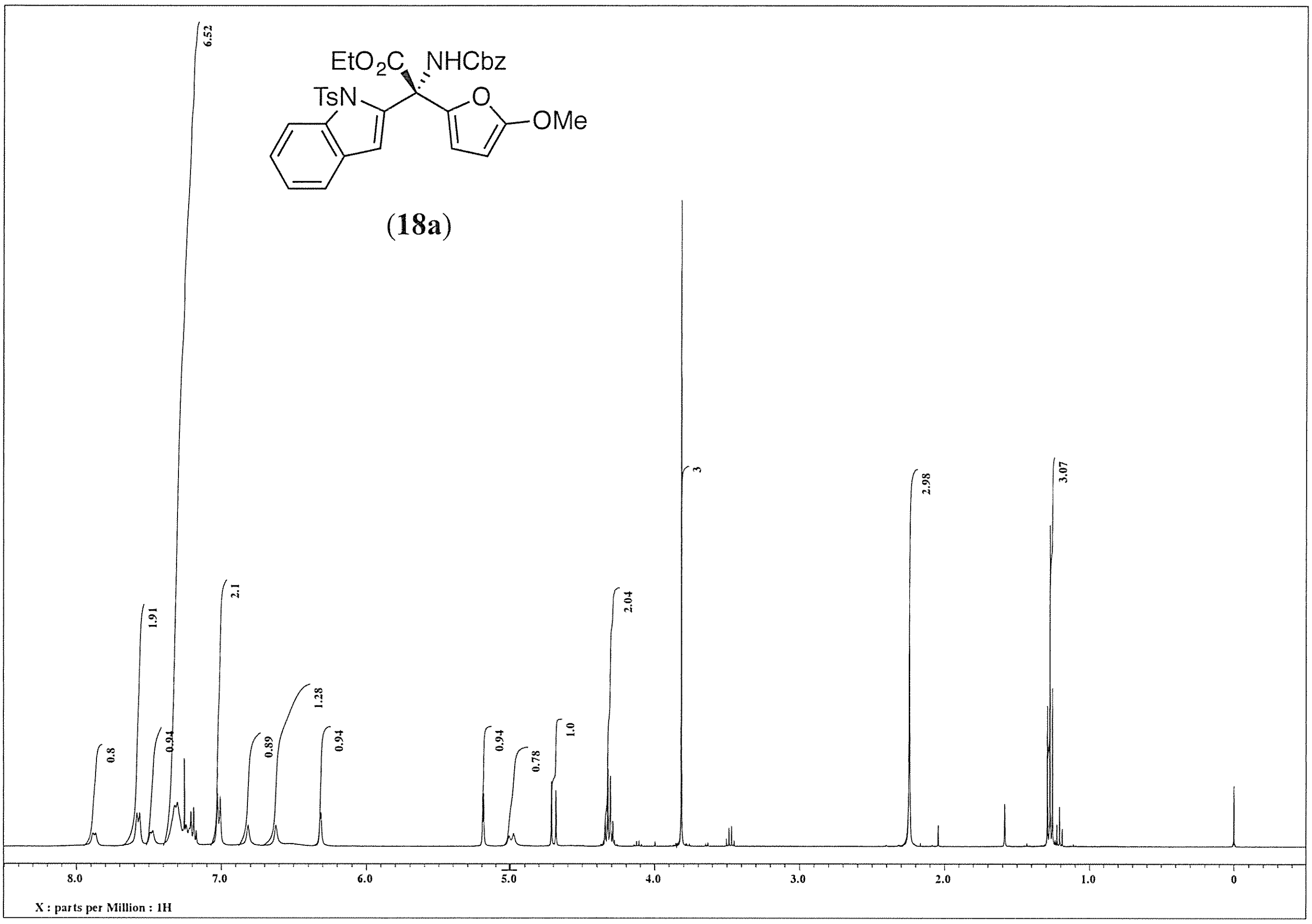

^{1}H NMR, 400 MHz, $CDCl_3$

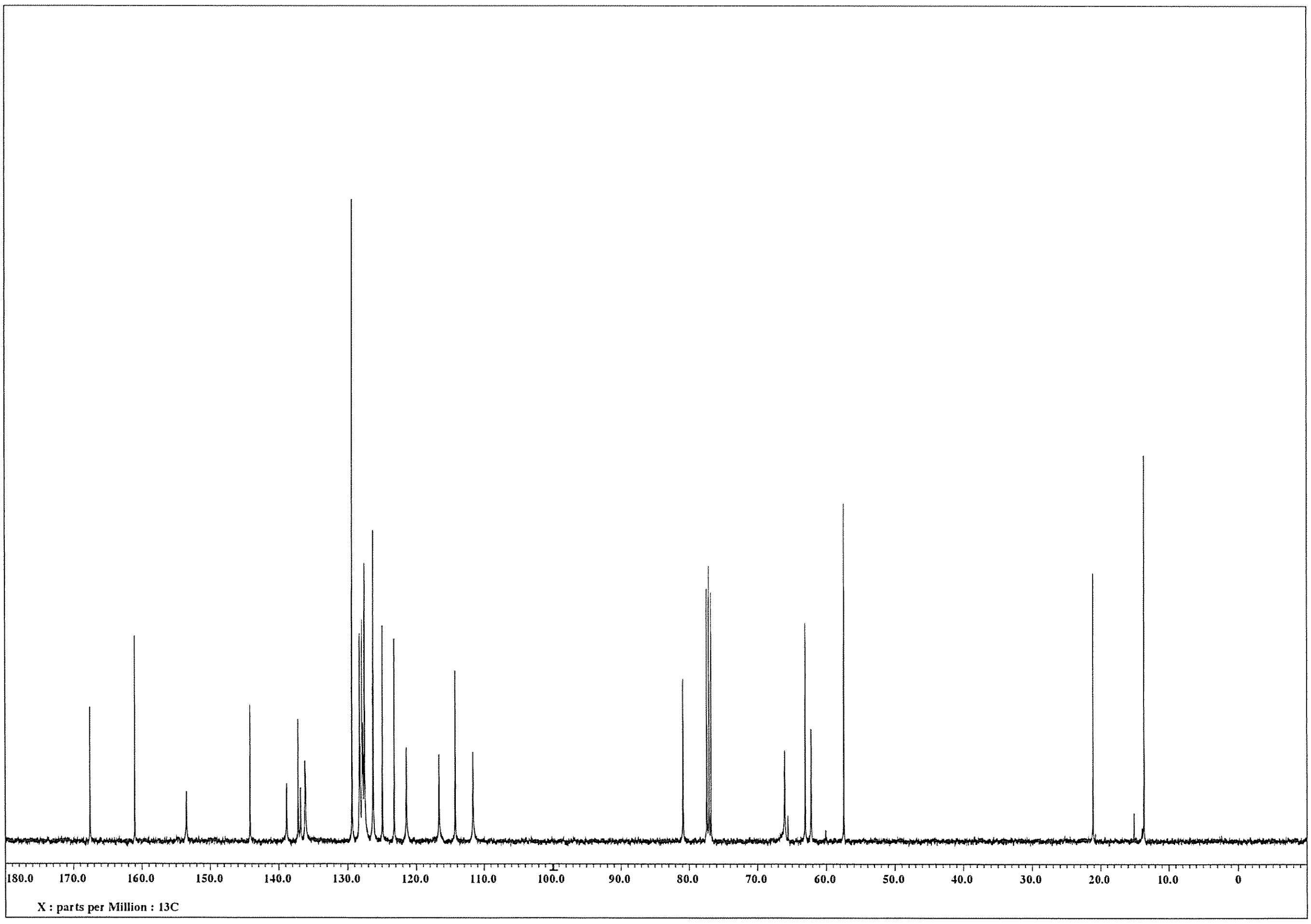

^{13}C NMR, 100 MHz, $CDCl_3$

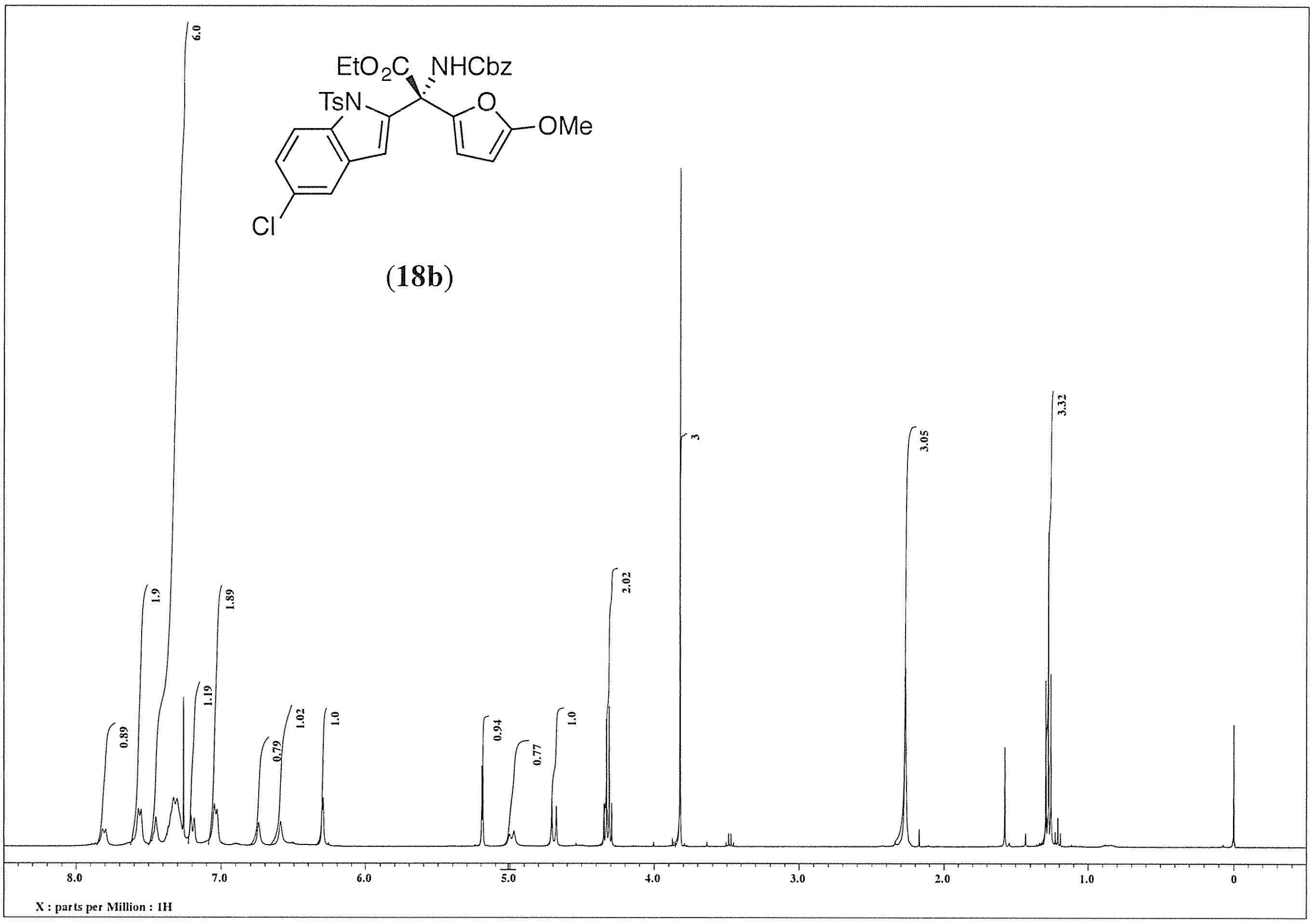

^{1}H NMR, 400 MHz, $CDCl_3$

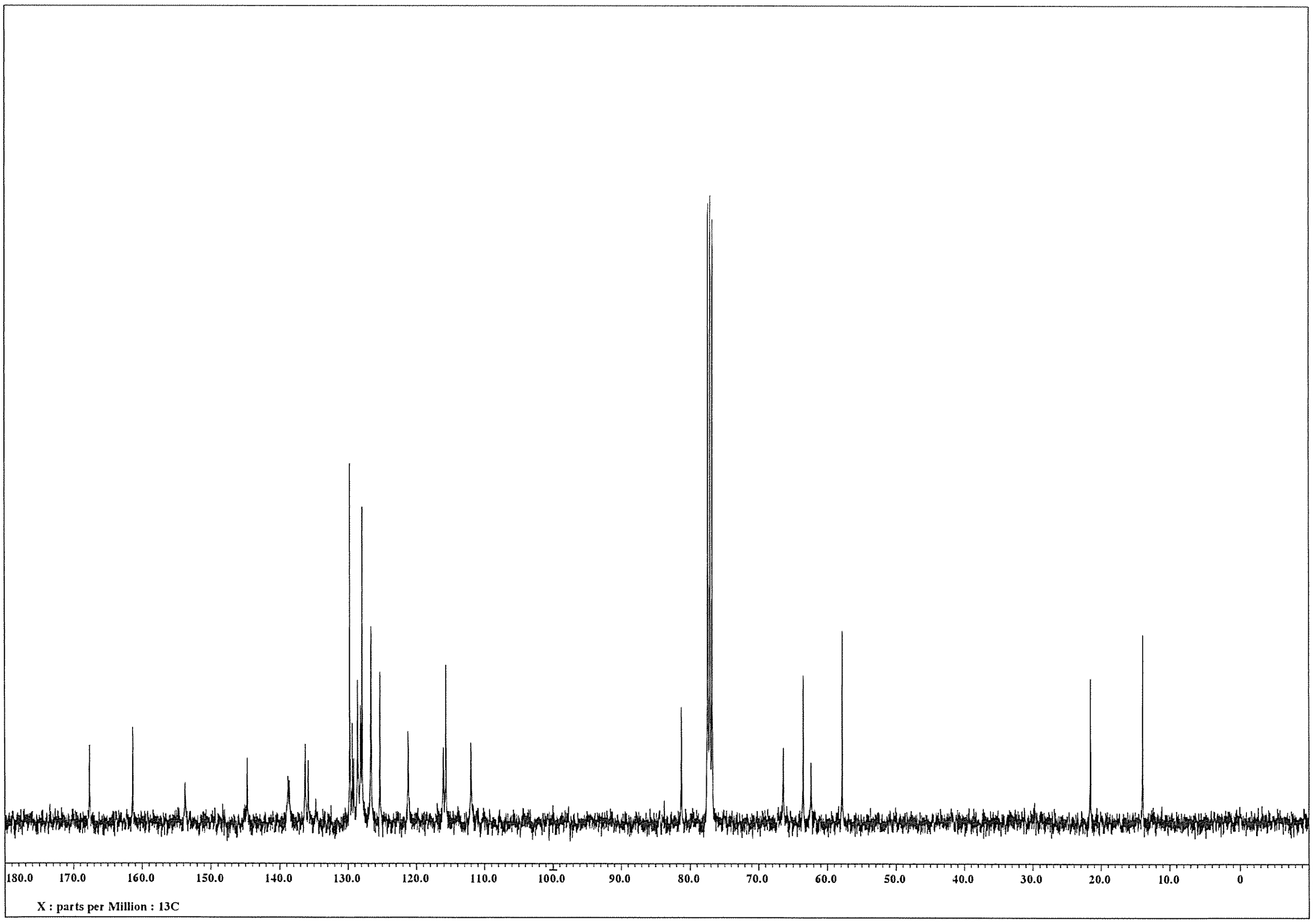

^{13}C NMR, 100 MHz, $CDCl_3$

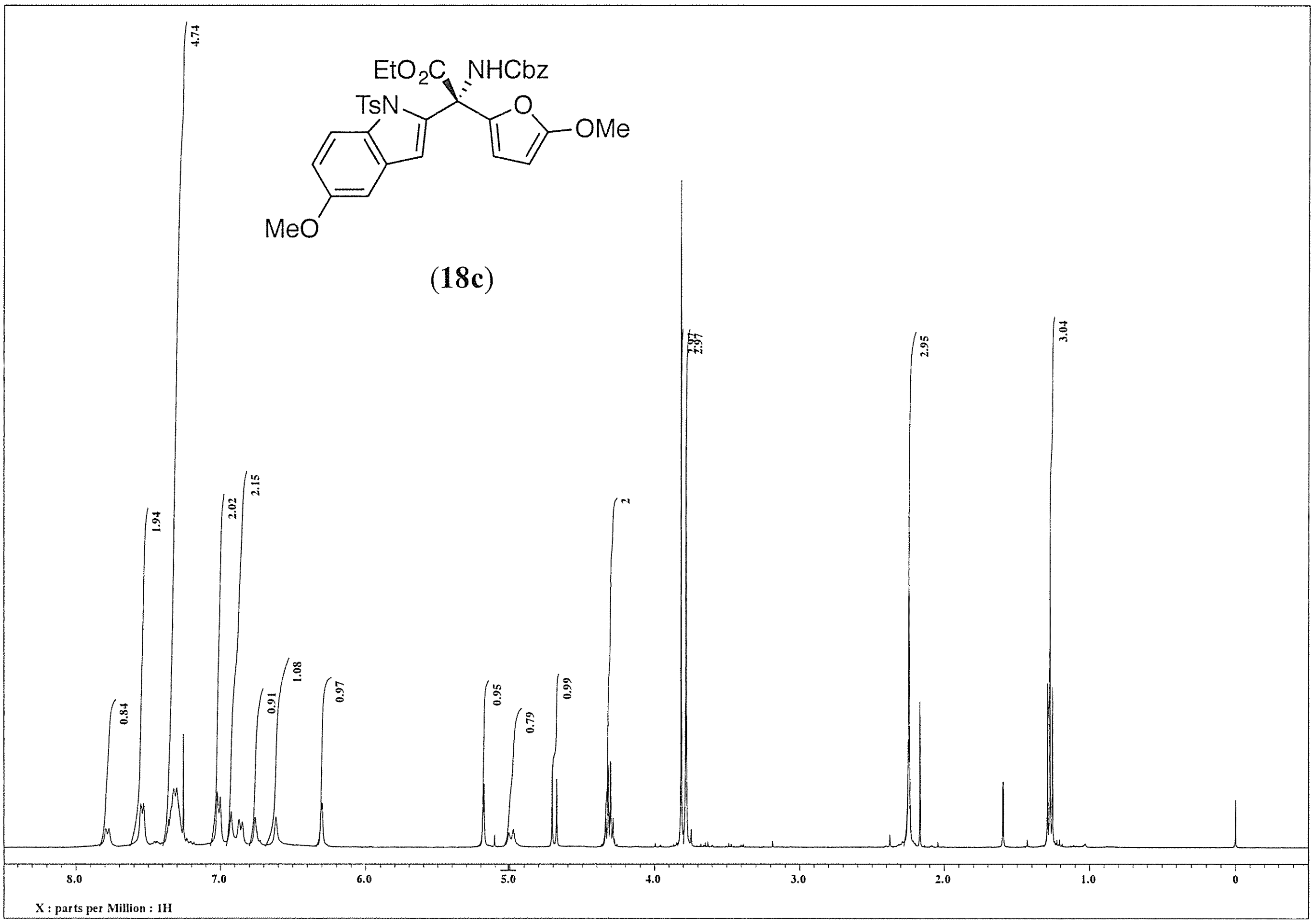

^{1}H NMR, 400 MHz, $CDCl_3$

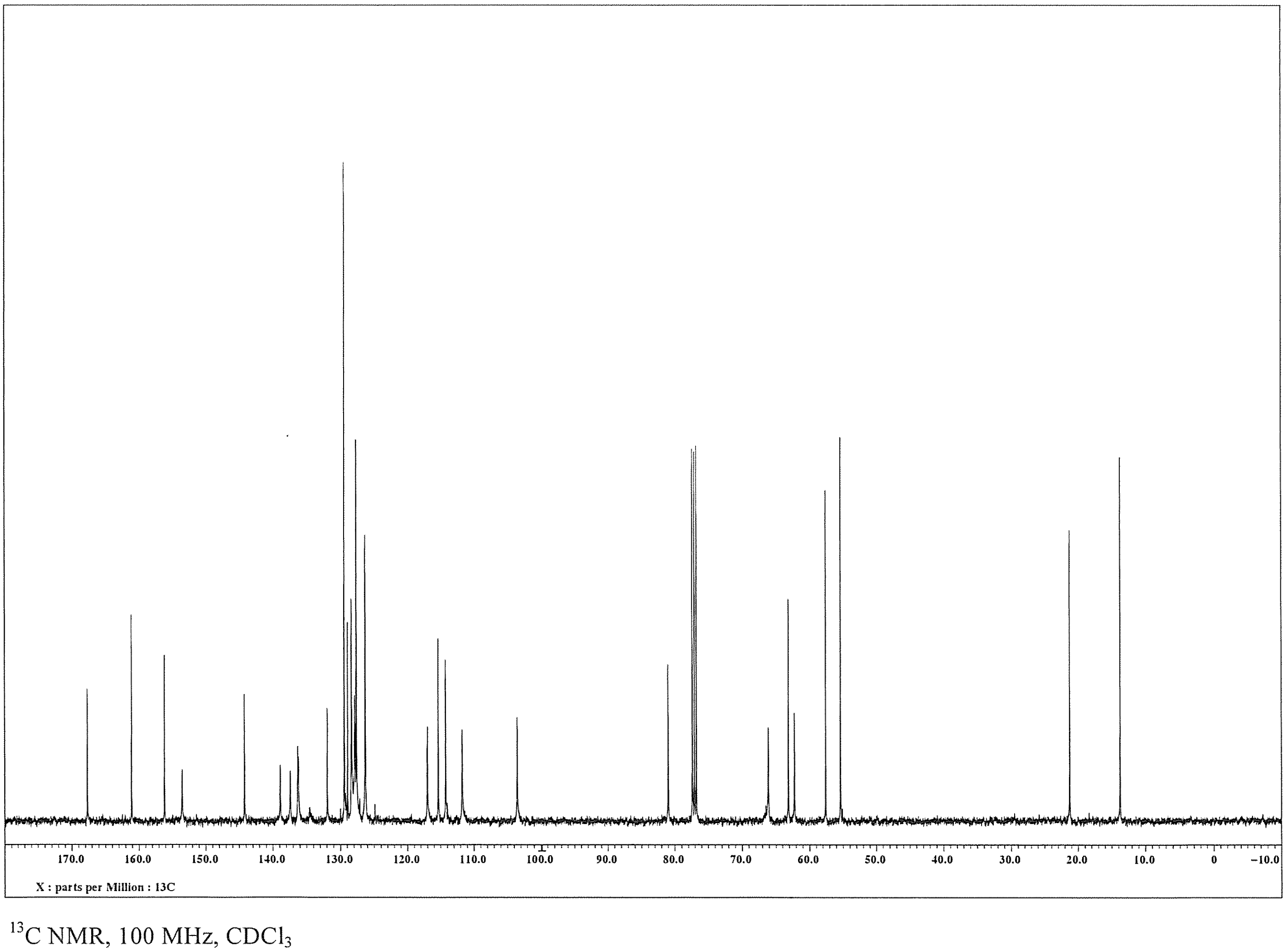

^{13}C NMR, 100 MHz, $CDCl_3$

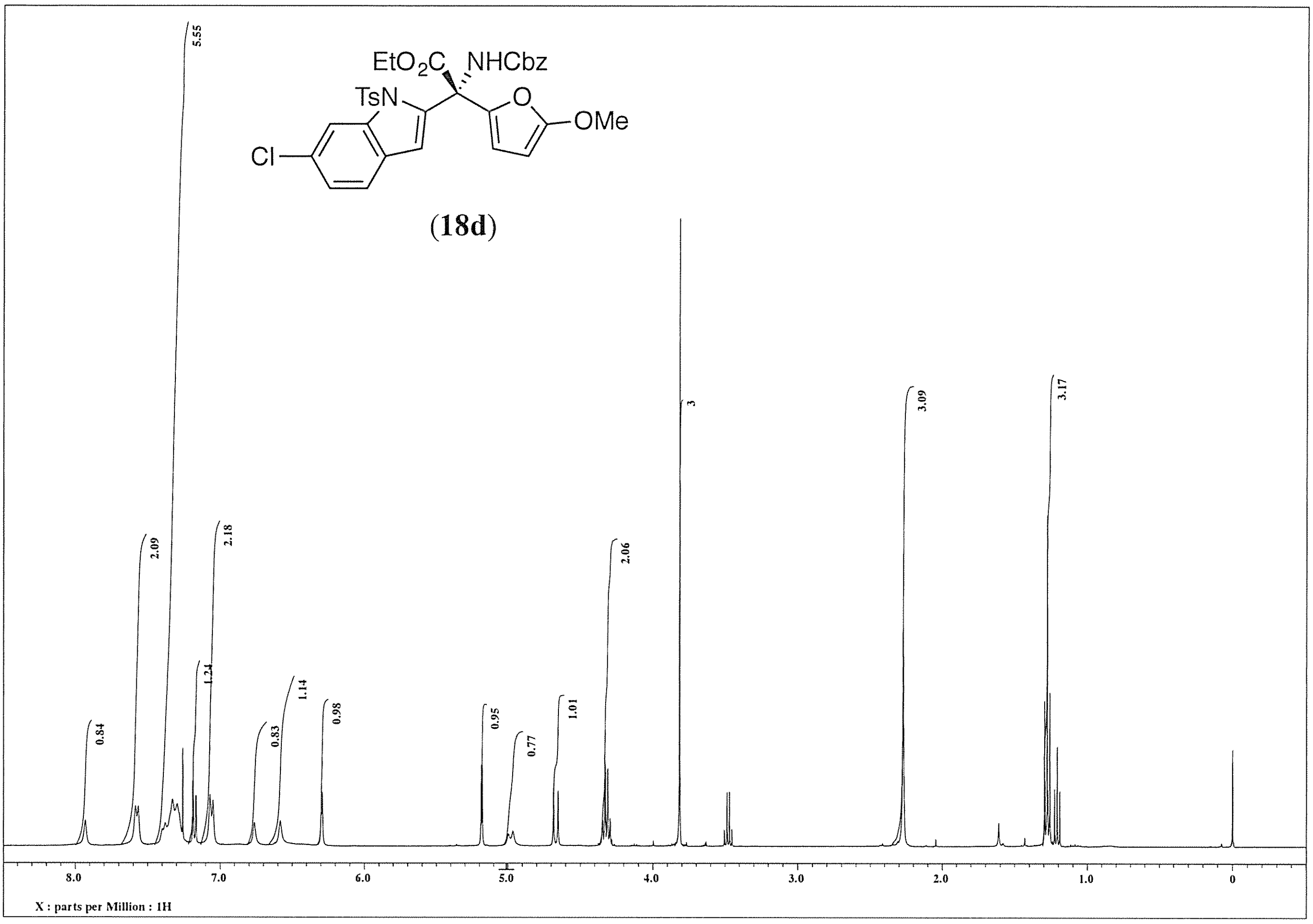

^{1}H NMR, 400 MHz, $CDCl_3$

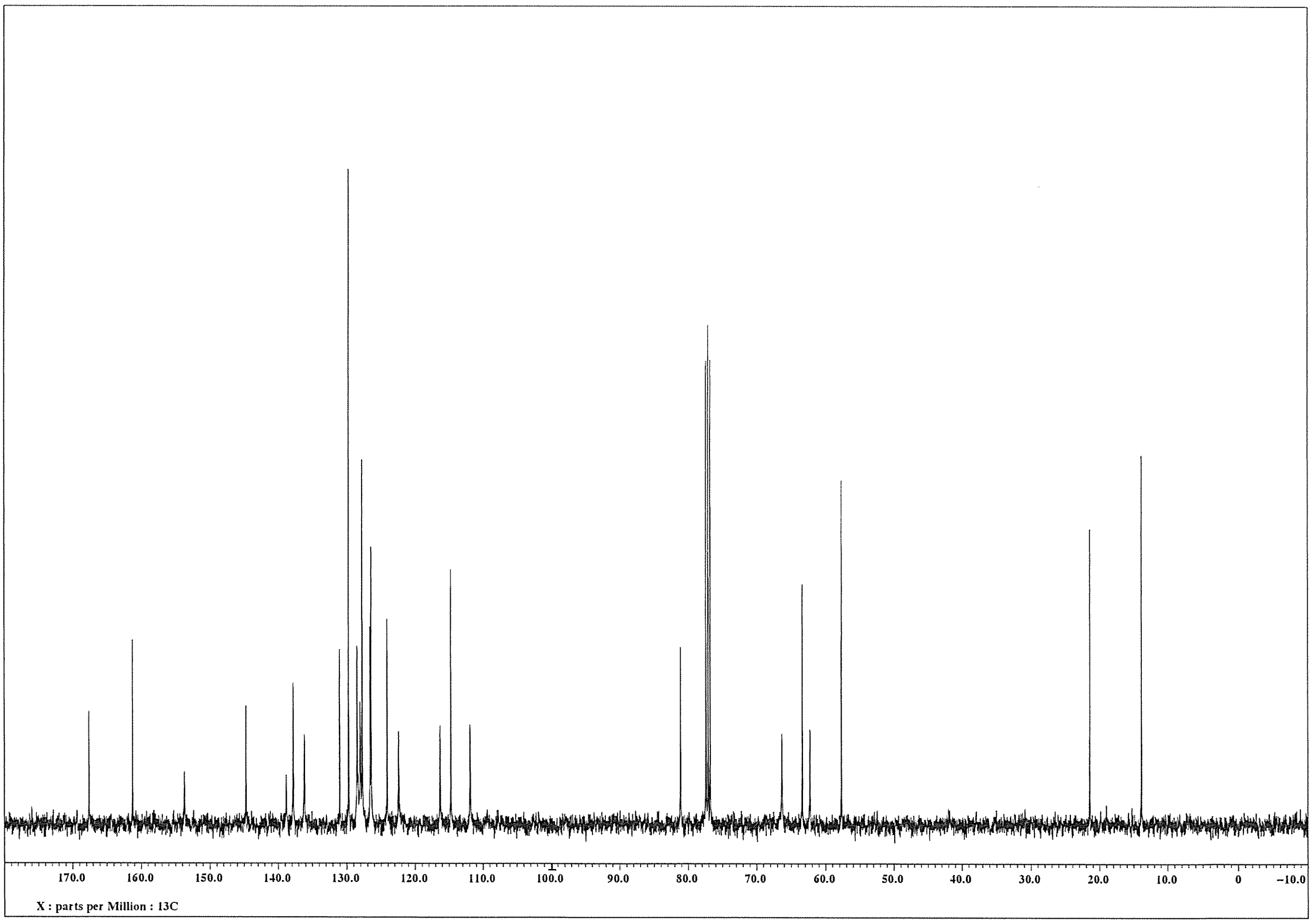

^{13}C NMR, 100 MHz, $CDCl_3$

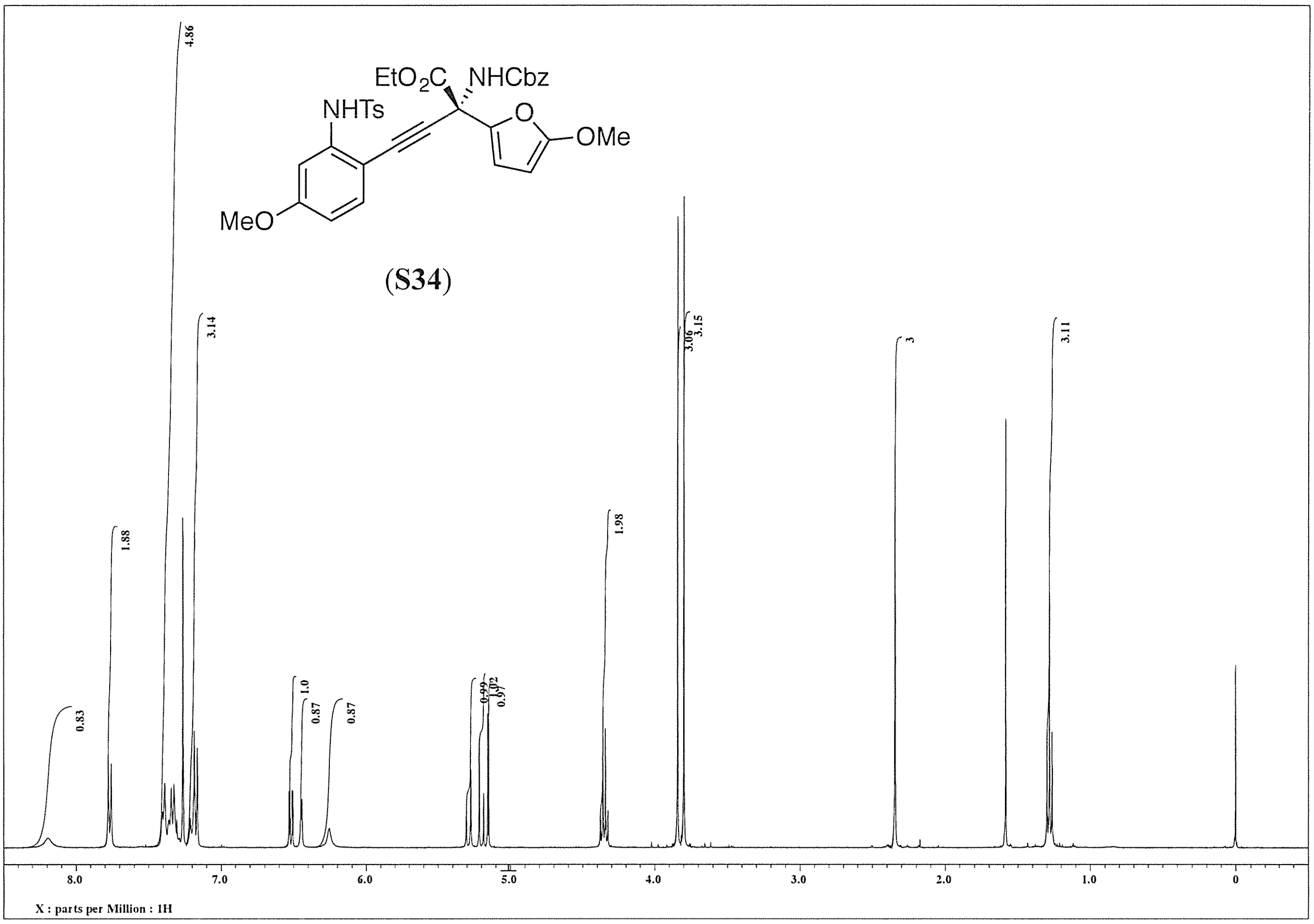

^{1}H NMR, 400 MHz, $CDCl_3$

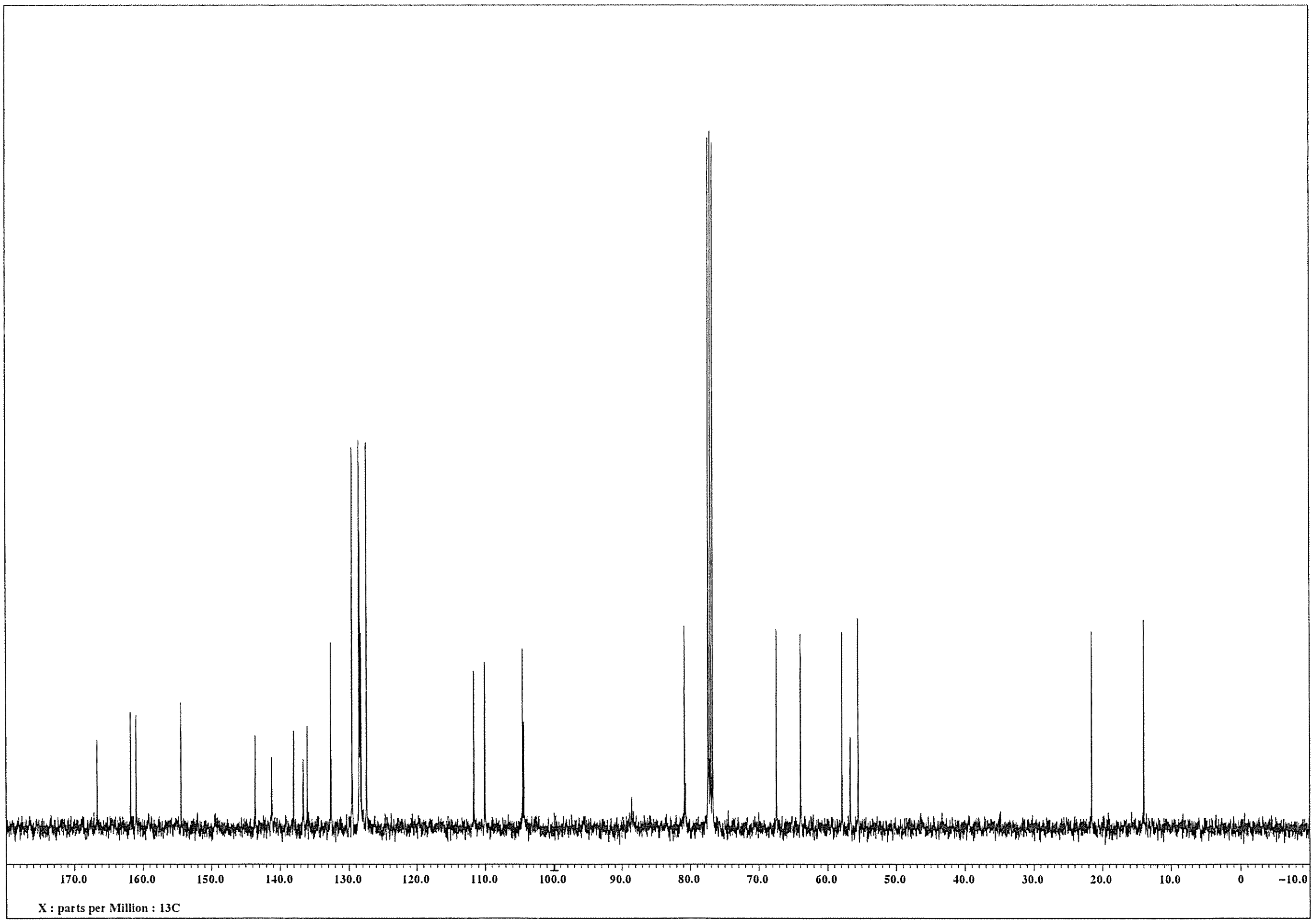

^{13}C NMR, 100 MHz, $CDCl_3$

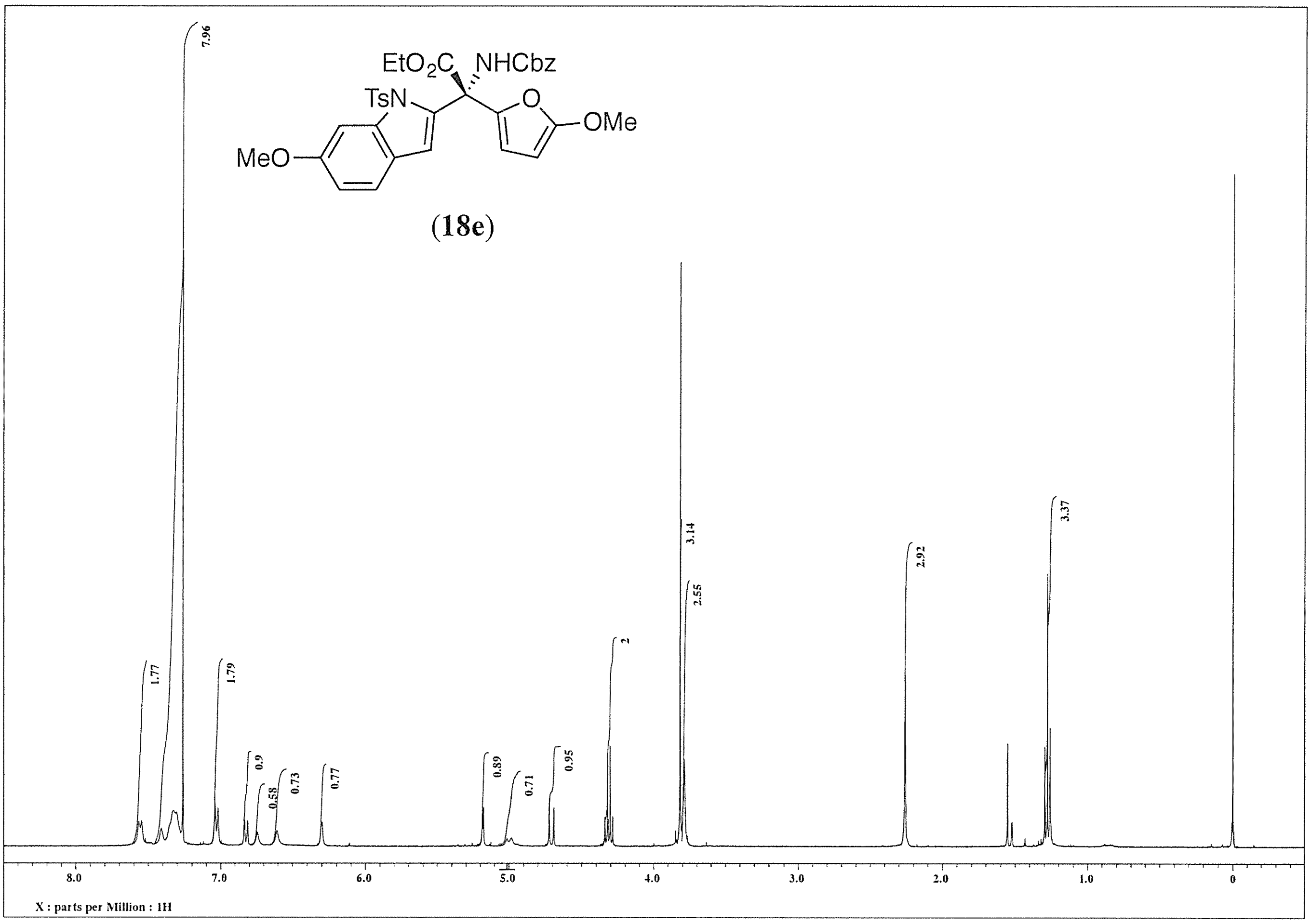

^{1}H NMR, 400 MHz, $CDCl_3$

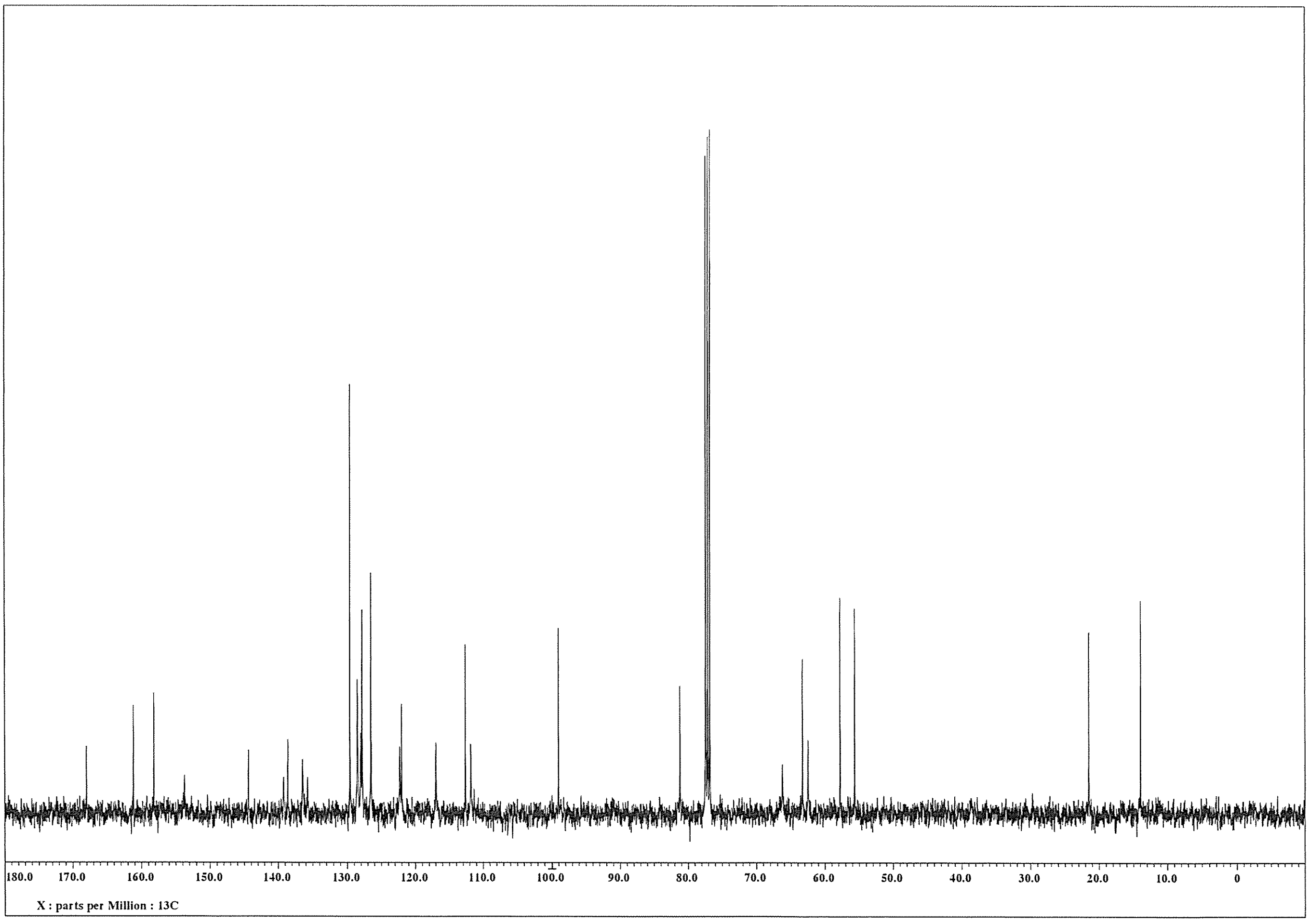

^{13}C NMR, 100 MHz, $CDCl_3$

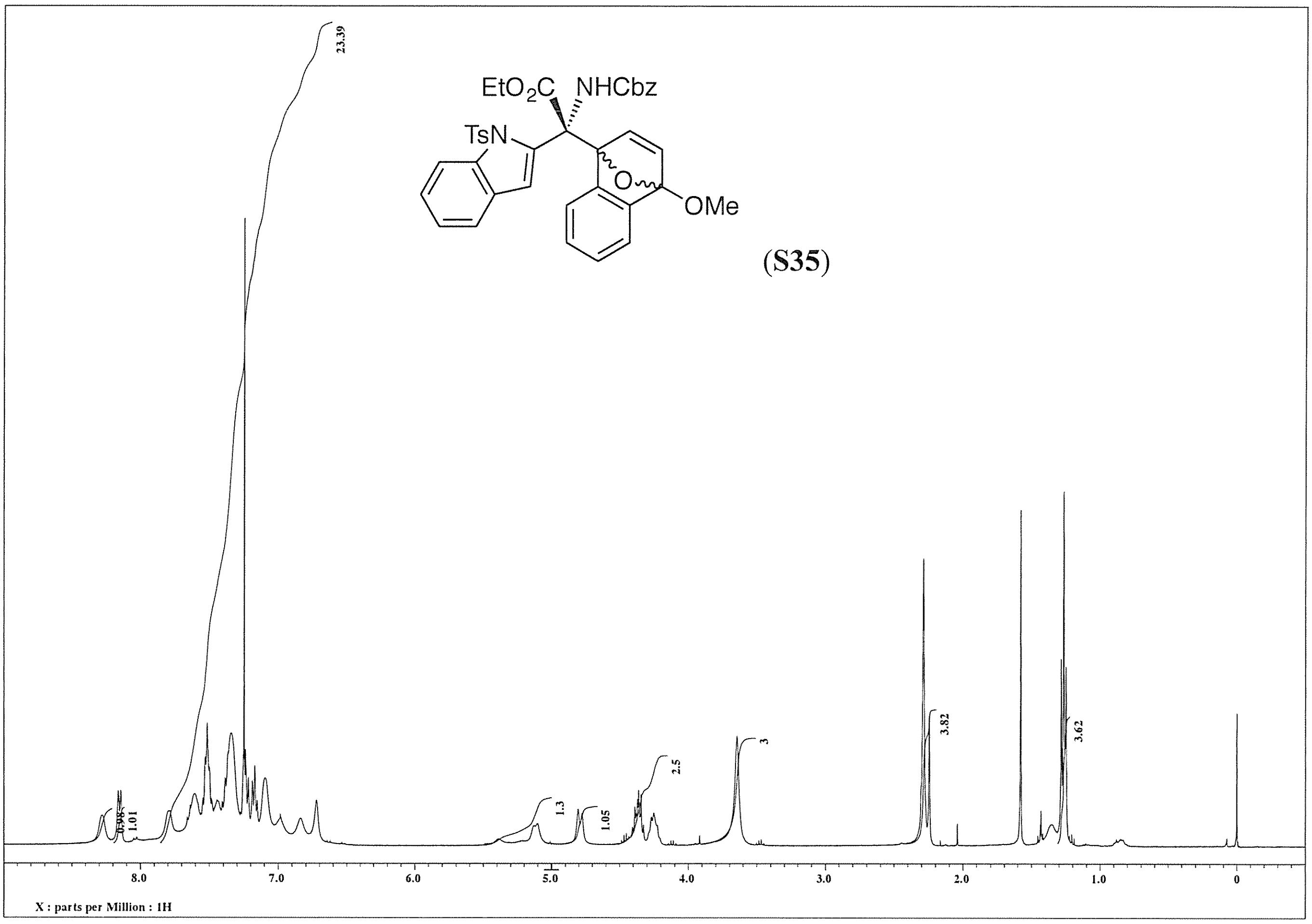

^{1}H NMR, 400 MHz, $CDCl_3$

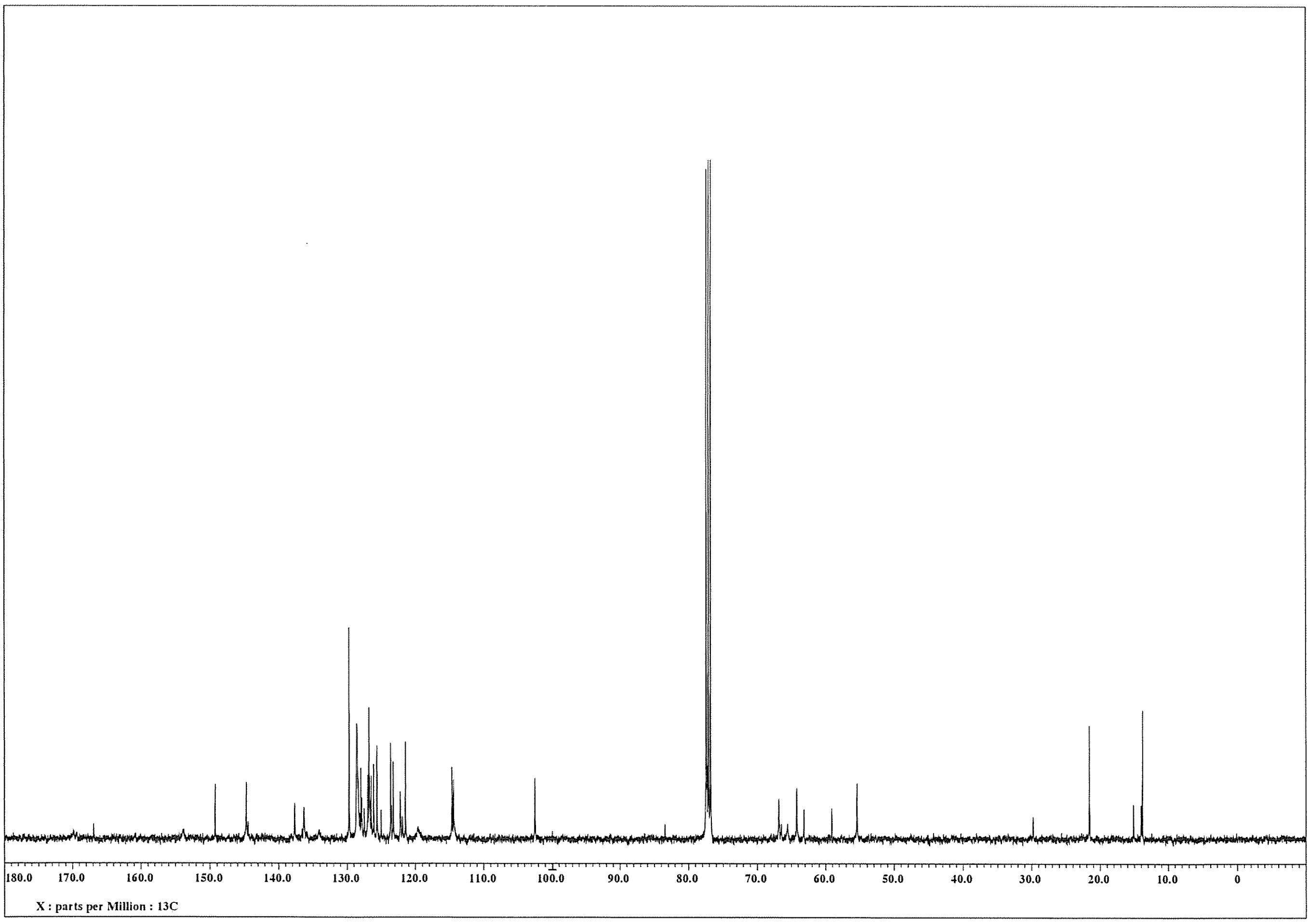

^{13}C NMR, 100 MHz, $CDCl_3$

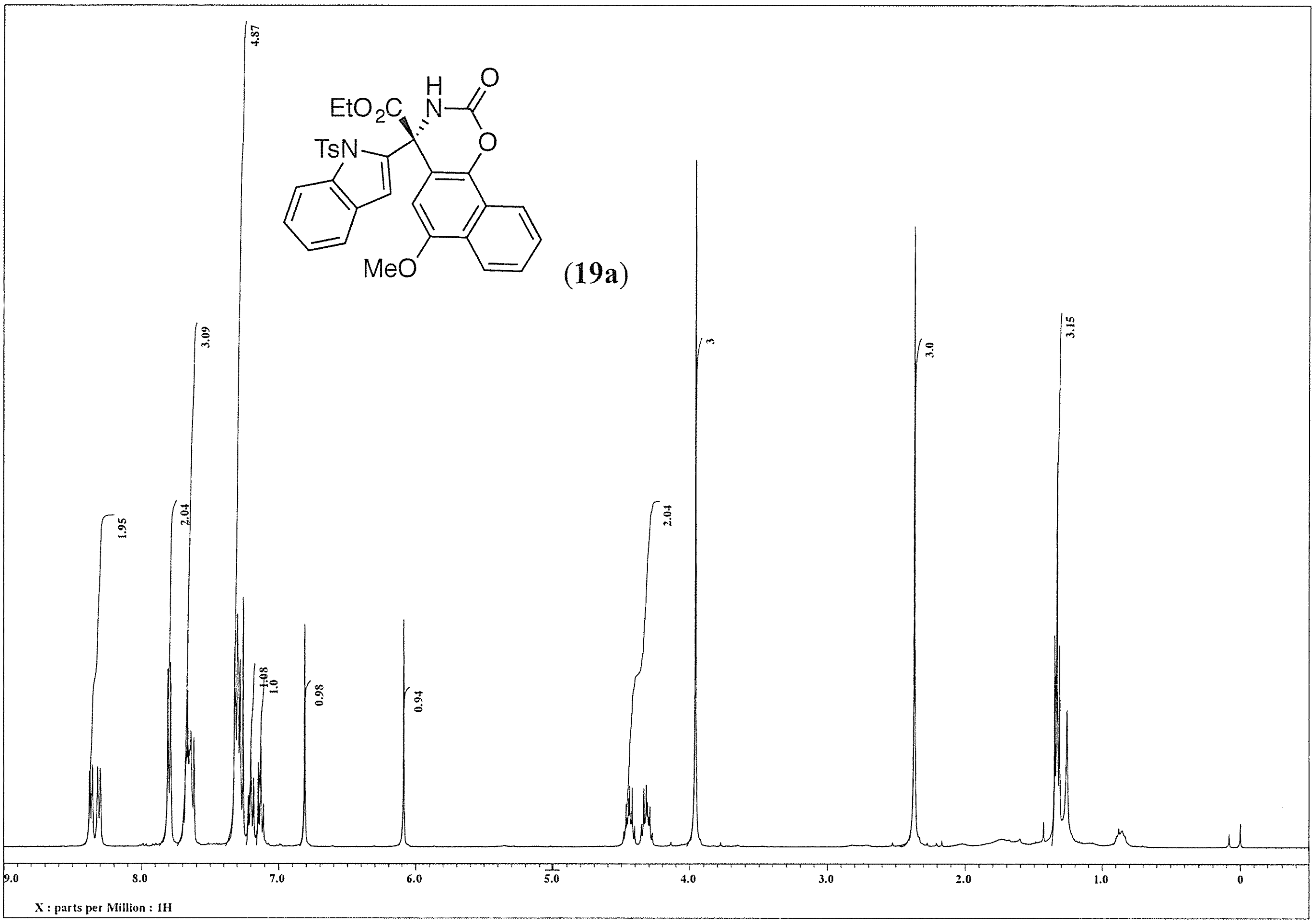

^{1}H NMR, 400 MHz, $CDCl_3$

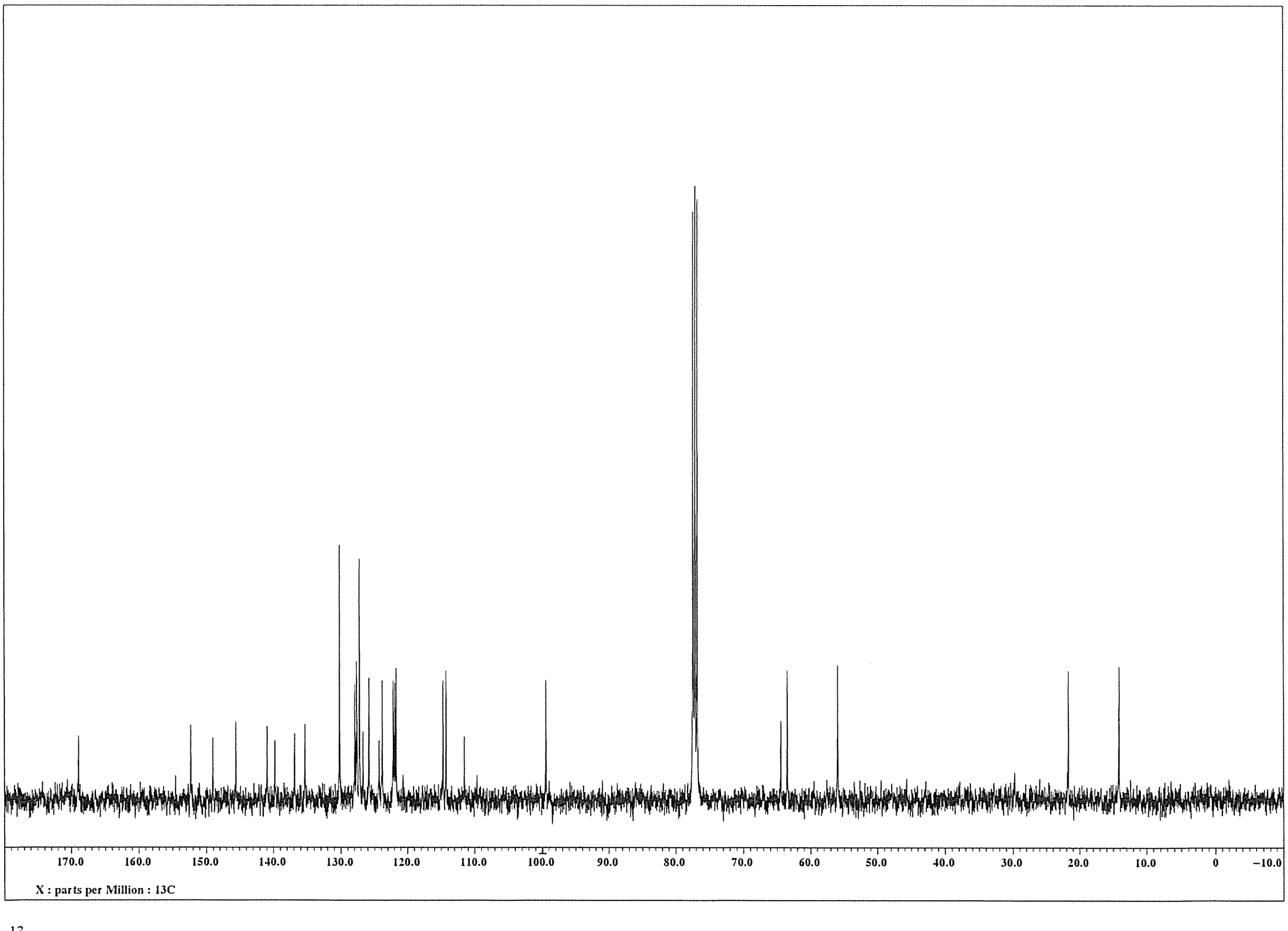

^{13}C NMR, 100 MHz, $CDCl_3$

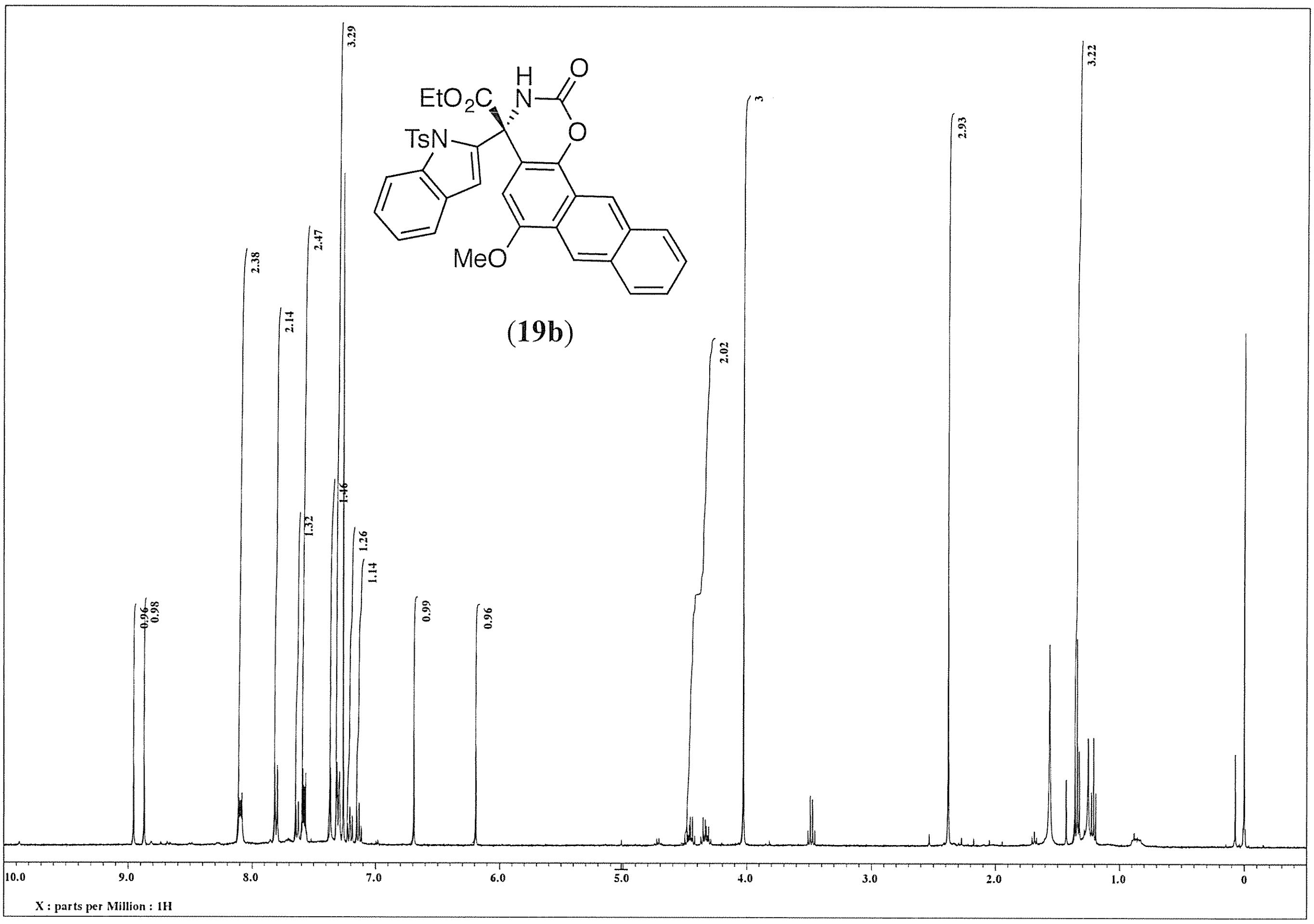

^{1}H NMR, 400 MHz, $CDCl_3$

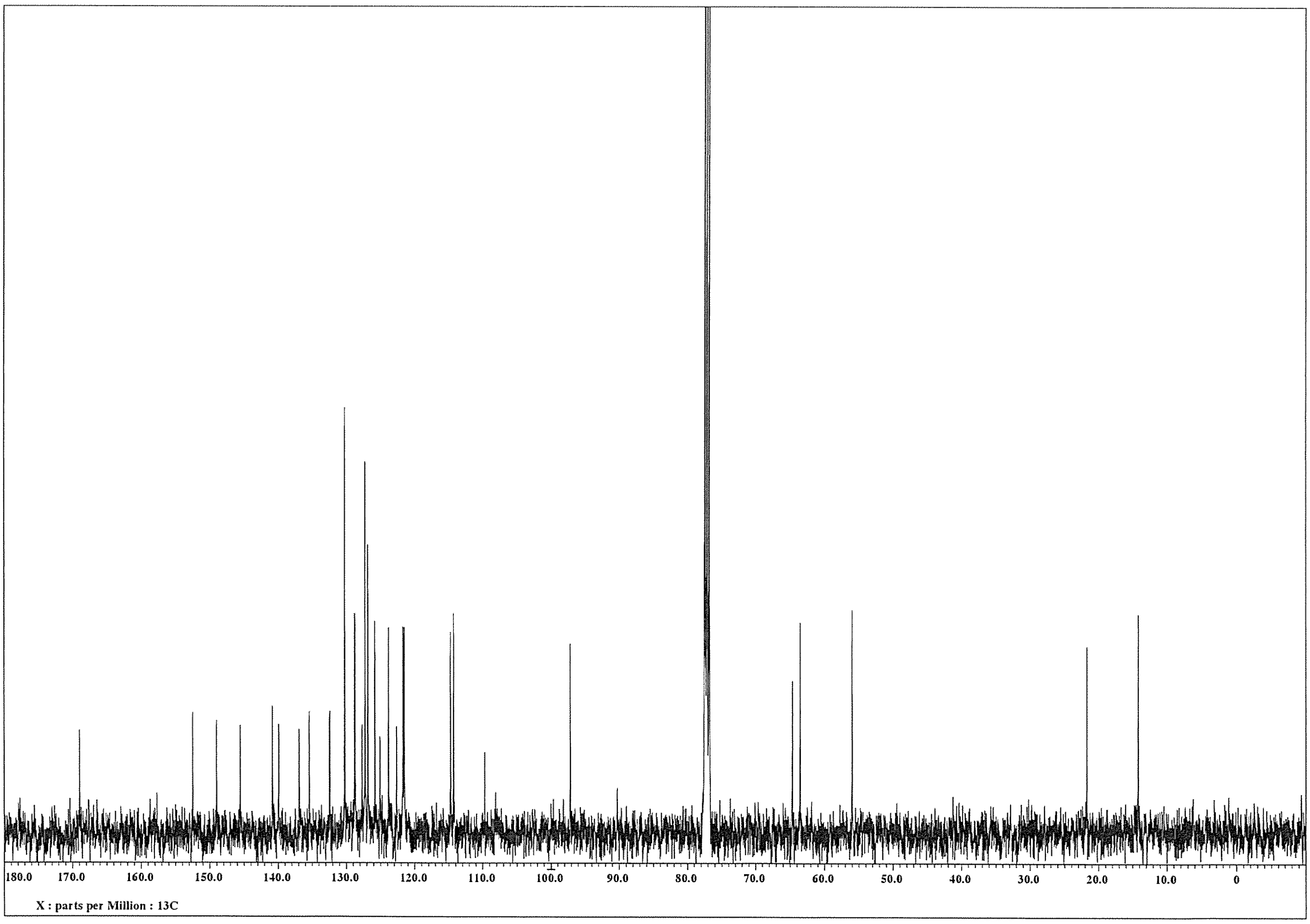

^{13}C NMR, 100 MHz, $CDCl_3$

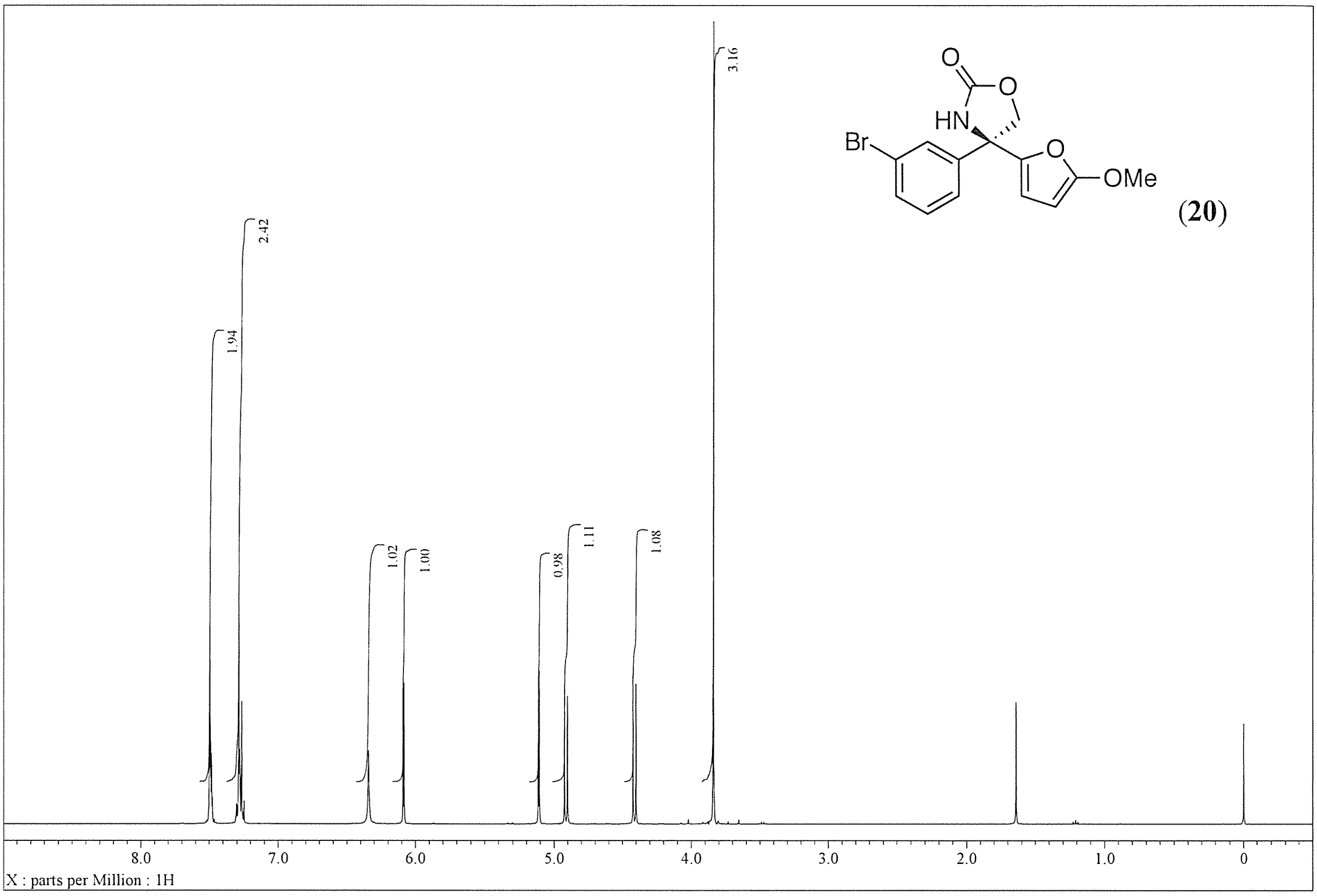

^{1}H NMR, 400 MHz, $CDCl_3$

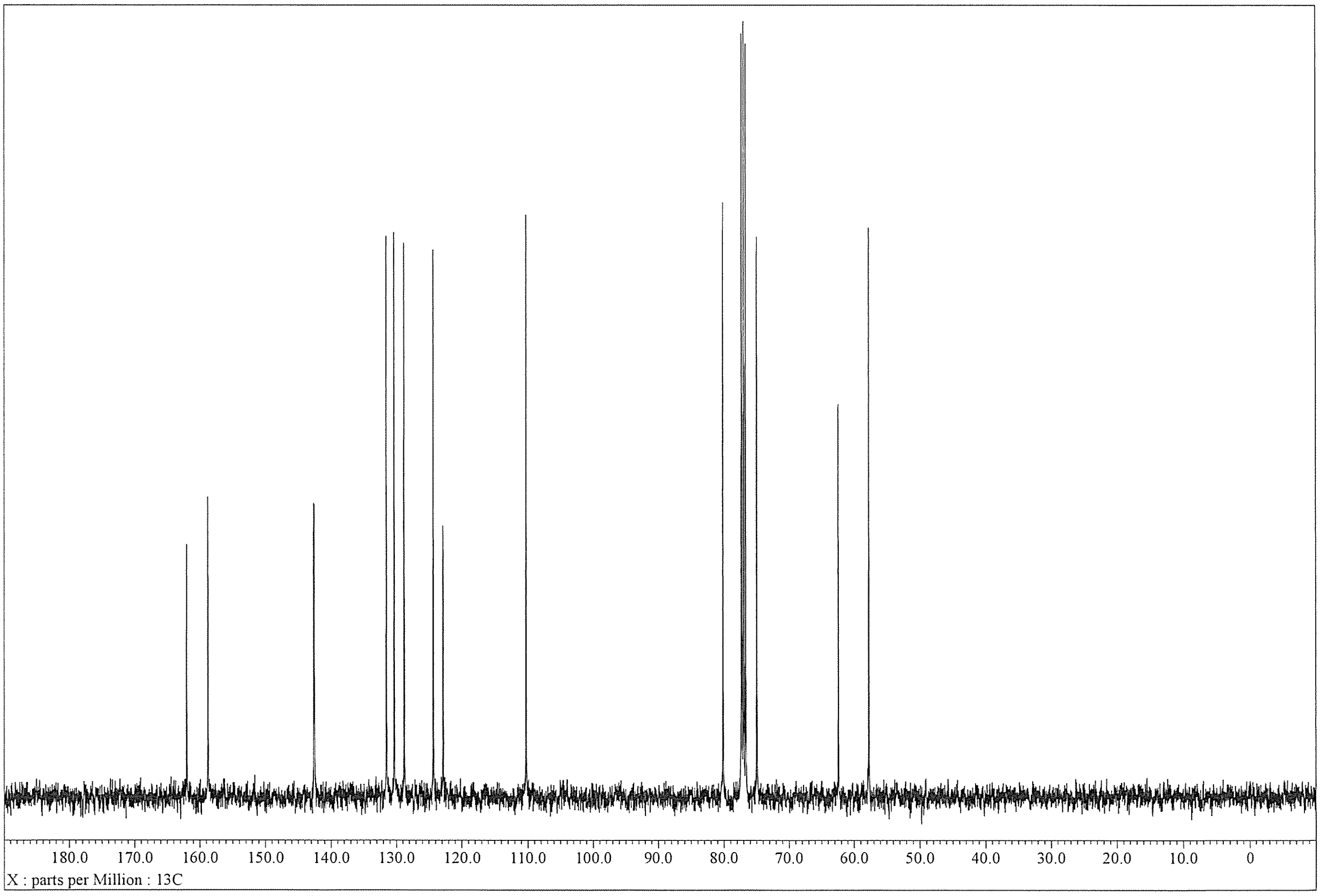

^{13}C NMR, 100 MHz, $CDCl_3$

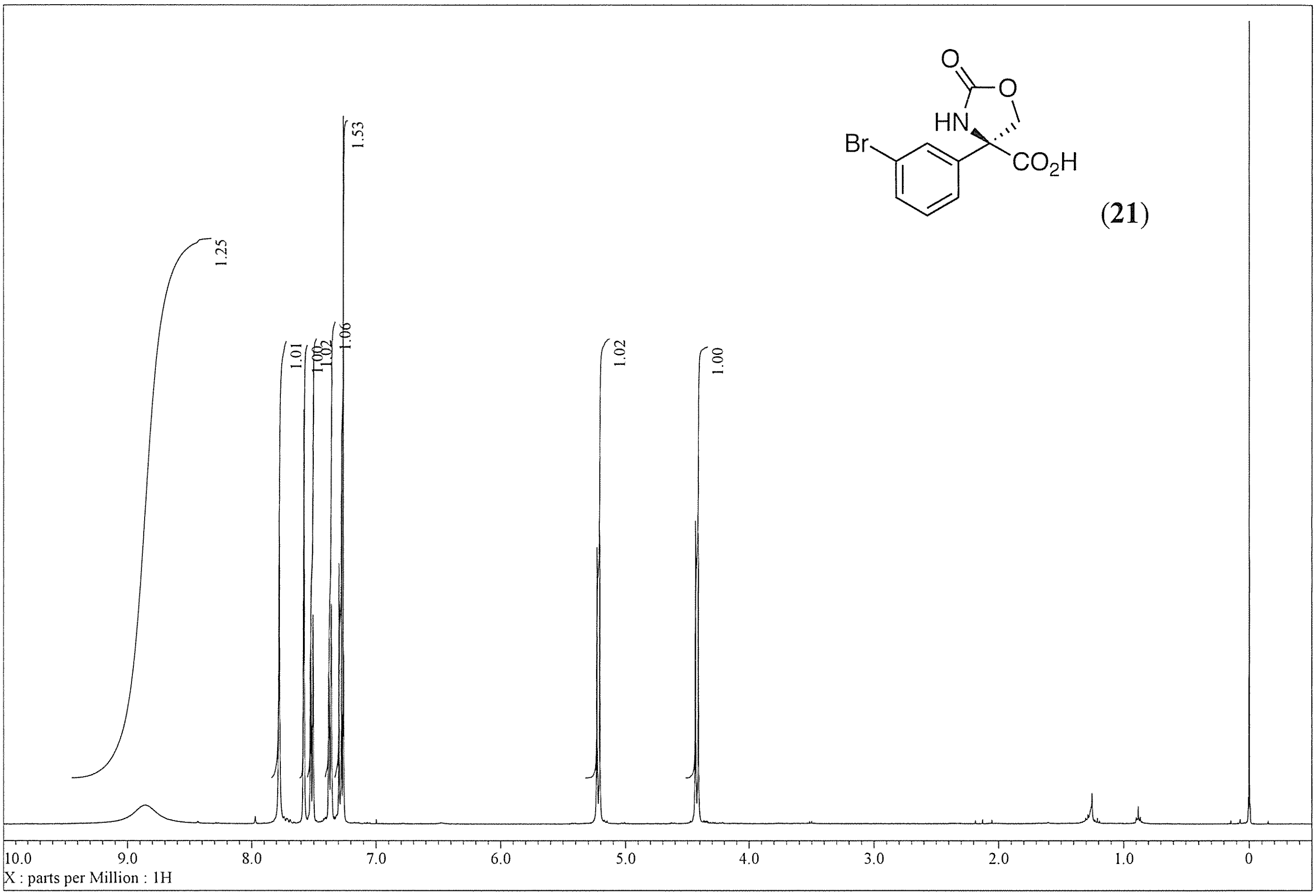

^{1}H NMR, 400 MHz, $CDCl_3$

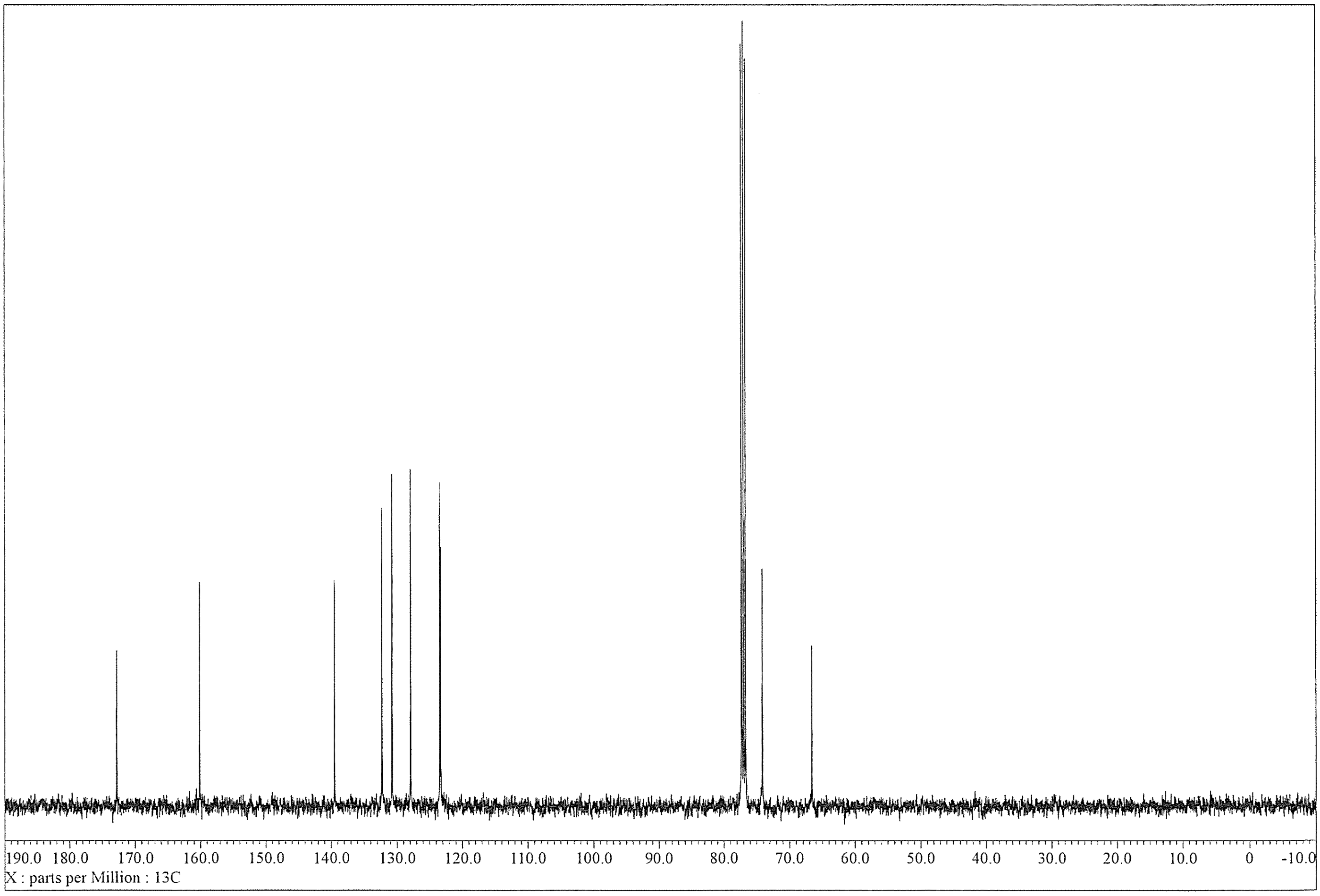

^{13}C NMR, 100 MHz, $CDCl_3$

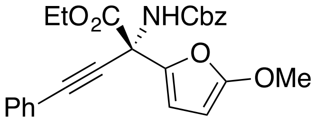

(**3a**)

AD-H,
n-hexane/*i*-PrOH = 1/1,
1.0 mL/min, 254 nm

Racemic **3a**

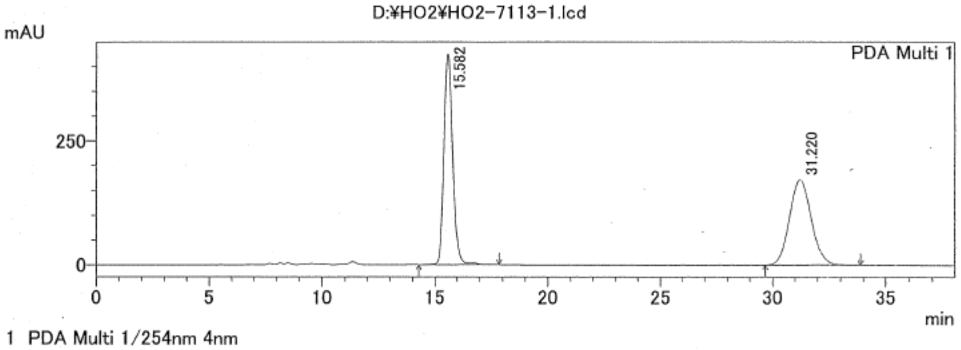

3a by the catalysis

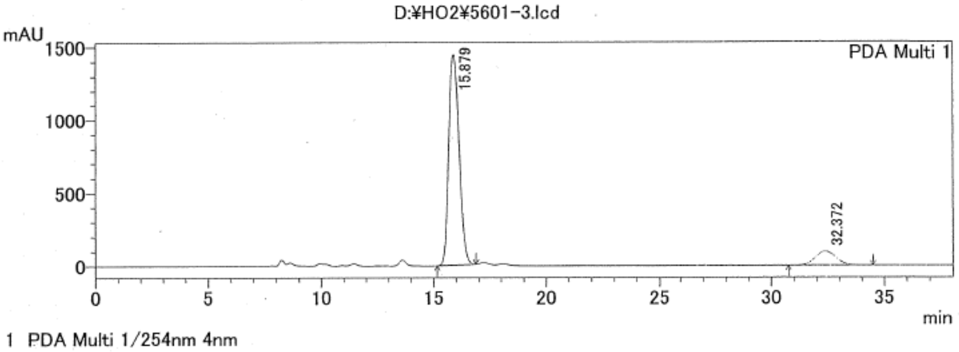

Peak Table

SPD-M20A Ch1 254nm 4nm

Peak No	RT (min)	Area	Height (mV)	% Area
1	15.879	46139871	1443911	88.011
2	32.372	6285531	98331	11.989
Total		52425402	1542242	100.000

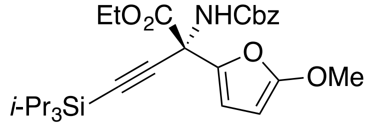

(**3b**)

AD-H,
n-hexane/*i*-PrOH = 9/1,
0.6 mL/min, 254 nm

Racemic **3b**

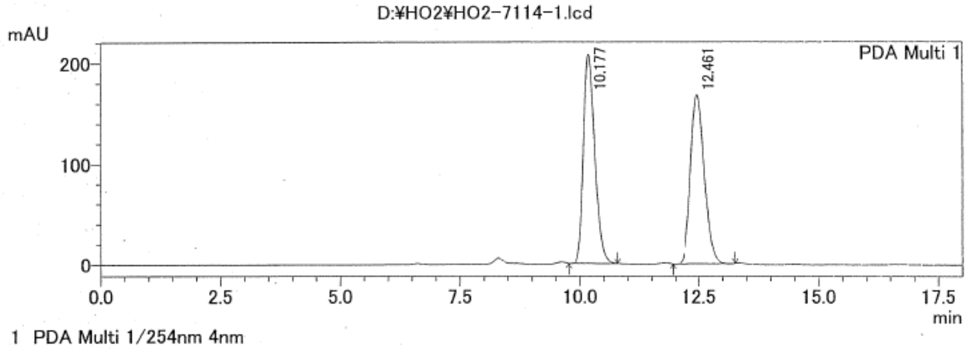

3b by the catalysis

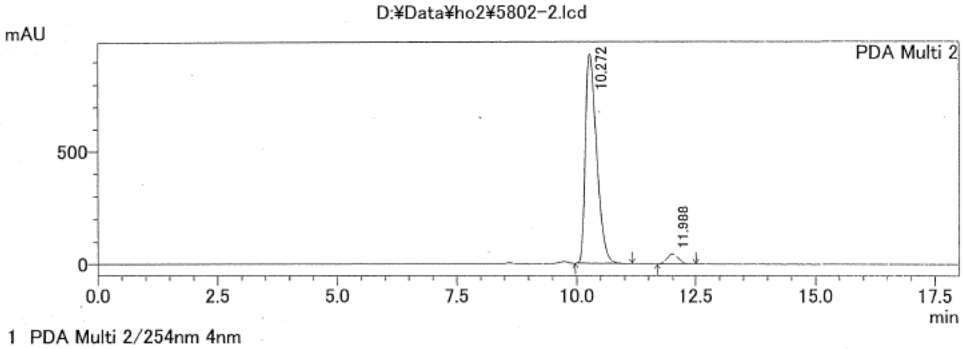

Peak Table

PDA Ch2 254nm 4nm

Peak No	RT (min)	Area	Height (mV)	% Area
1	10.272	15153756	929106	95.343
2	11.988	740249	42893	4.657
合計		15894005	971999	100.000

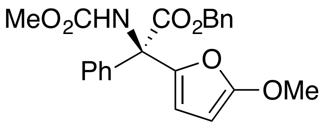

(**8a**)

AD-H,
n-hexane/*i*-PrOH = 1/1,
0.6 mL/min, 254 nm

Racemic **8a**

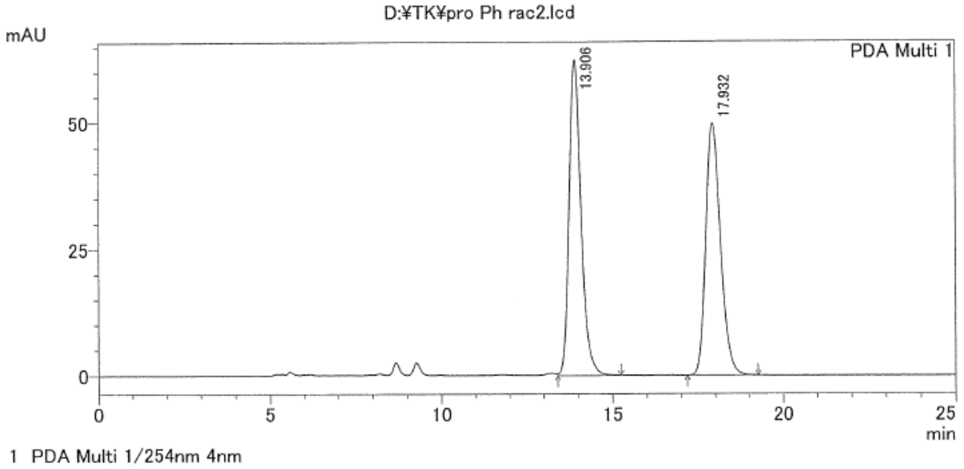

8a by the catalysis

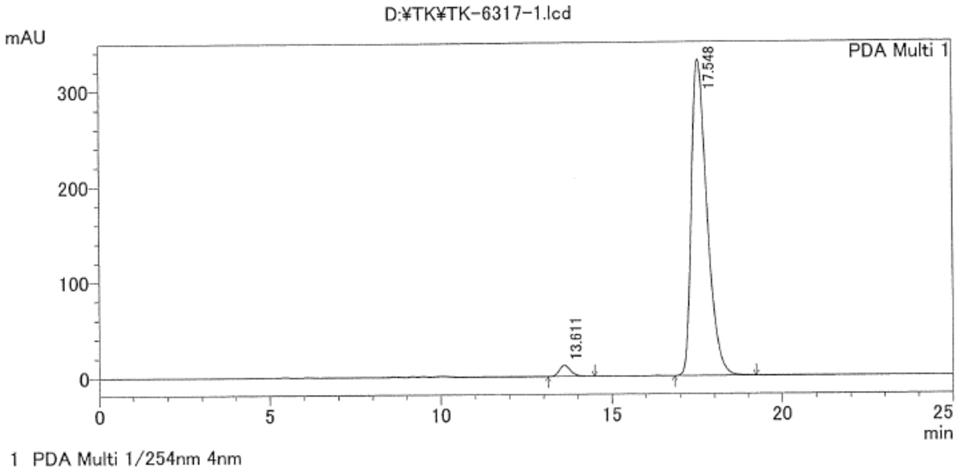

Peak Table

SPD-M20A Ch1 254nm 4nm

Peak No	RT (min)	Area	Height (mV)	% Area
1	13.611	275847	11579	2.532
2	17.548	10619806	329836	97.468
Total		10895653	341415	100.000

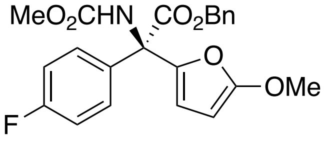

(**8b**)

AD-H,
n-hexane/*i*-PrOH = 1/1,
0.6 mL/min, 240 nm

Racemic **8b**

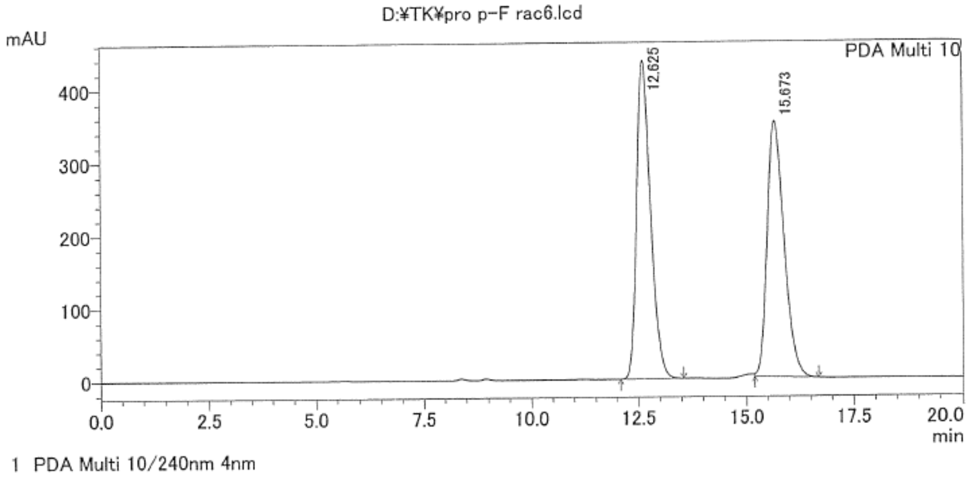

8b by the catalysis

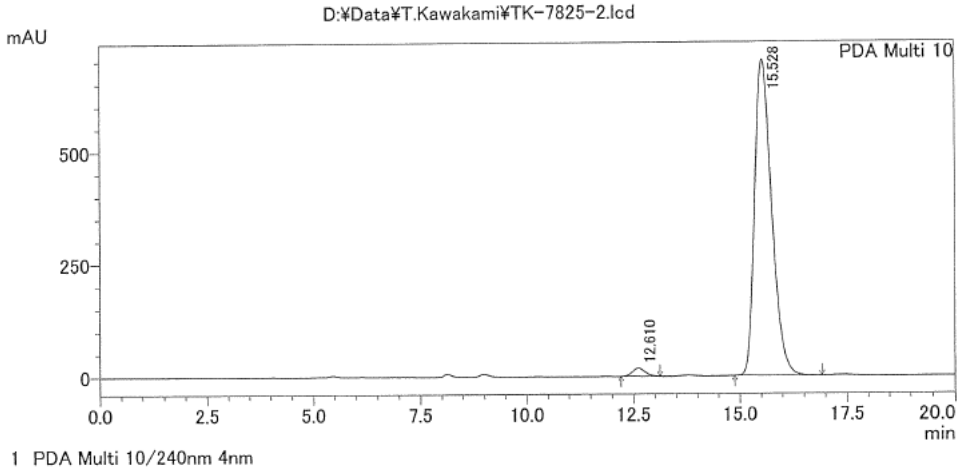

PDA Ch10 240nm 4nm

Peak No	RT (min)	Area	Height (mV)	% Area
1	12.610	366490	17820	1.848
2	15.528	19466932	700562	98.152
Total		19833422	718381	100.000

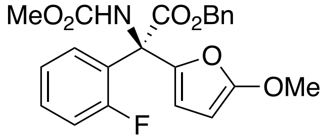

(**8c**)

OD-3,
n-hexane/*i*-PrOH = 1/1,
1.0 mL/min, 254 nm

Racemic **8c**

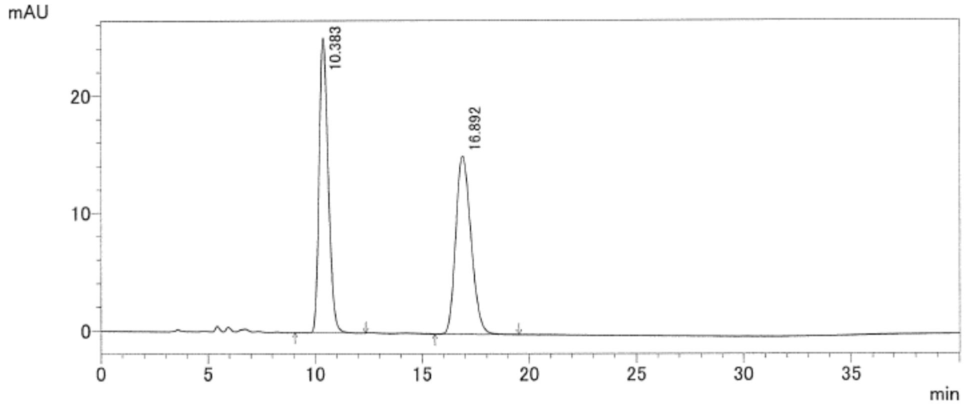

8c by the catalysis

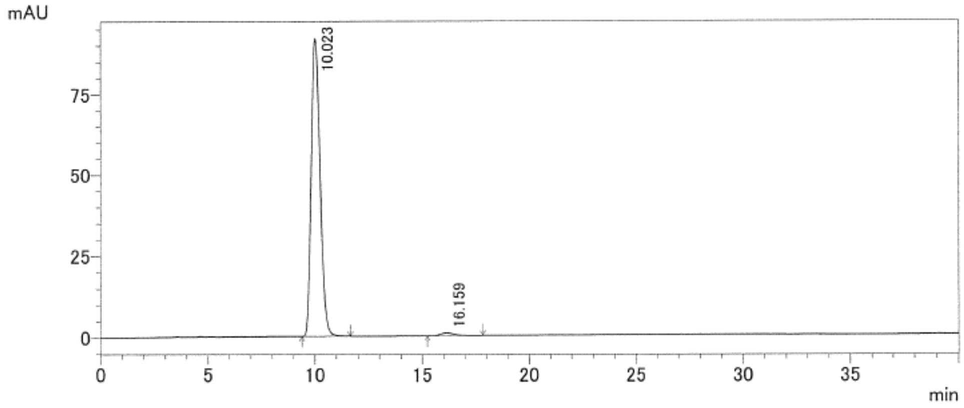

PDA Ch4 254nm

Peak No.	RT (min)	Area	Height	% Area
1	10.023	2625634	91803	98.506
2	16.159	39816	891	1.494
Total		2665450	92694	100.000

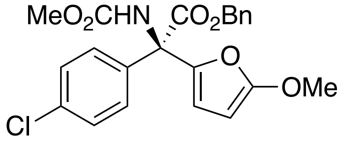

(**8d**)

AD-H,
n-hexane/*i*-PrOH = 1/1,
0.6 mL/min, 254 nm

Racemic **8d**

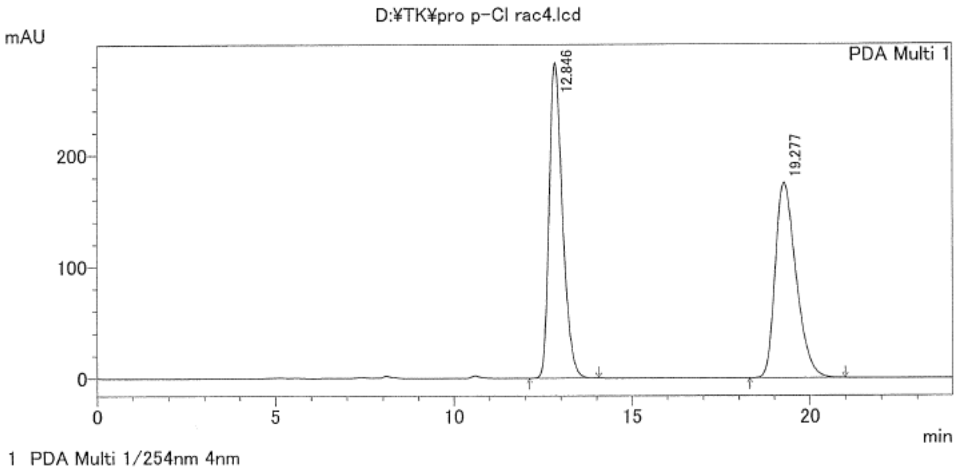

8d by the catalysis

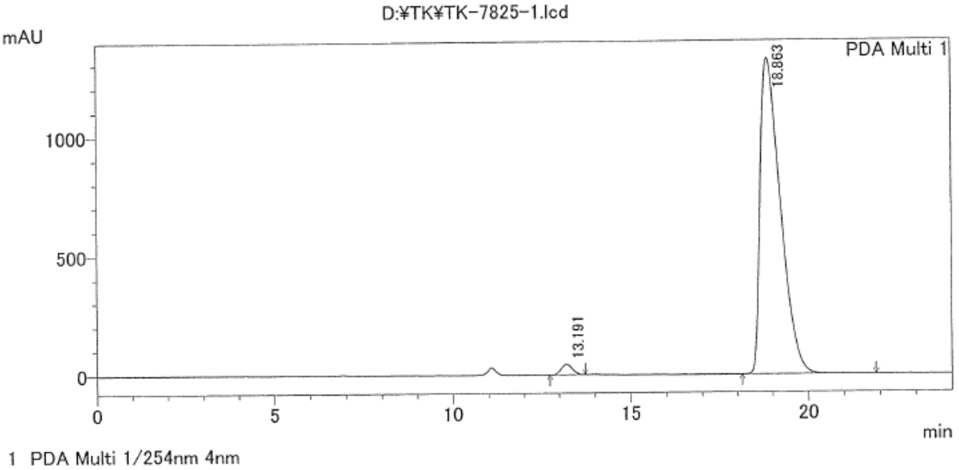

Peak Table

SPD-M20A Ch1 254nm 4nm

Peak No	RT (min)	Area	Height (mV)	% Area
1	13.191	965076	44652	1.797
2	18.863	52733762	1322733	98.203
Total		53698838	1367385	100.000

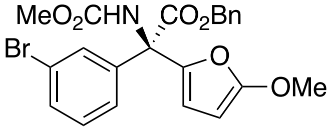

(**8e**)

AD-H,
n-hexane/*i*-PrOH = 1/1,
0.6 mL/min, 254 nm

Racemic **8e**

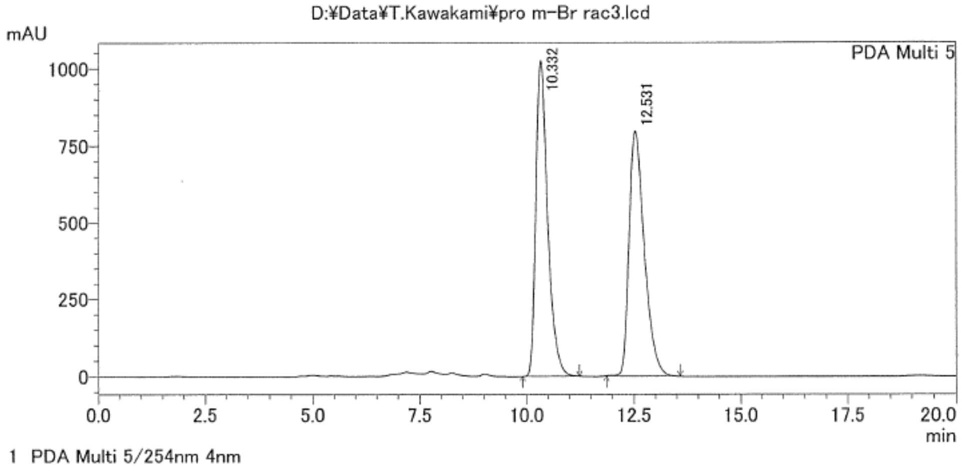

8e by the catalysis

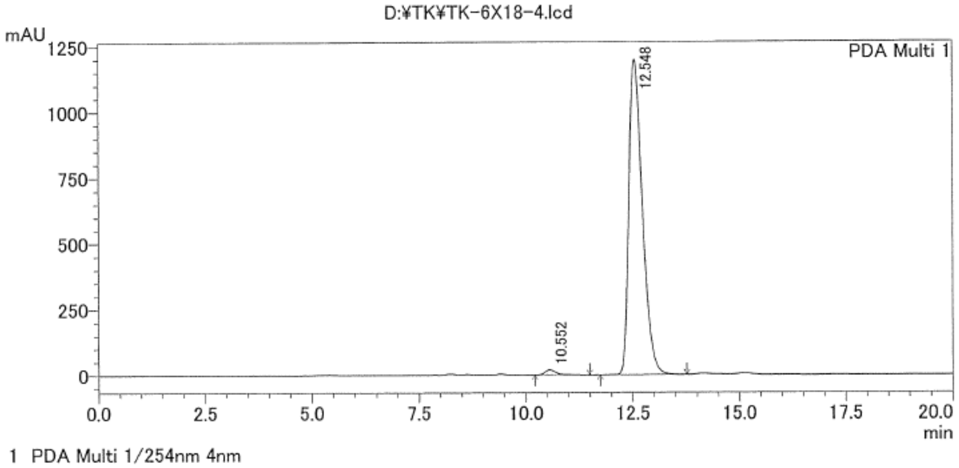

Peak Table

SPD-M20A Ch1 254nm 4nm

Peak No	RT (min)	Area	Height (mV)	% Area
1	10.552	352914	19075	1.311
2	12.548	26569332	1197815	98.689
Total		26922246	1216890	100.000

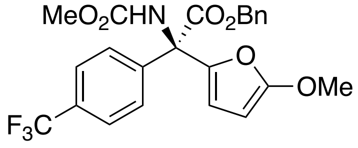

(8f)

IC-3,
n-hexane/*i*-PrOH = 1/1,
0.6 mL/min, 254 nm

Racemic **8f**

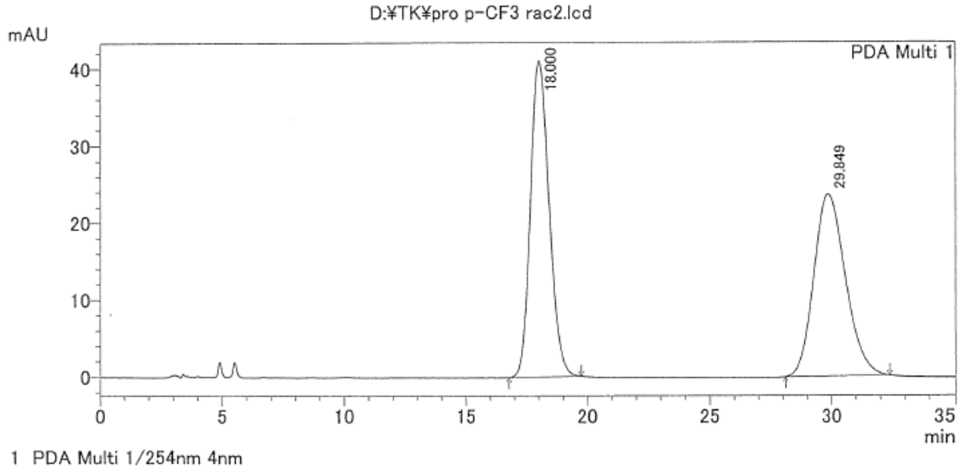

8f by the catalysis

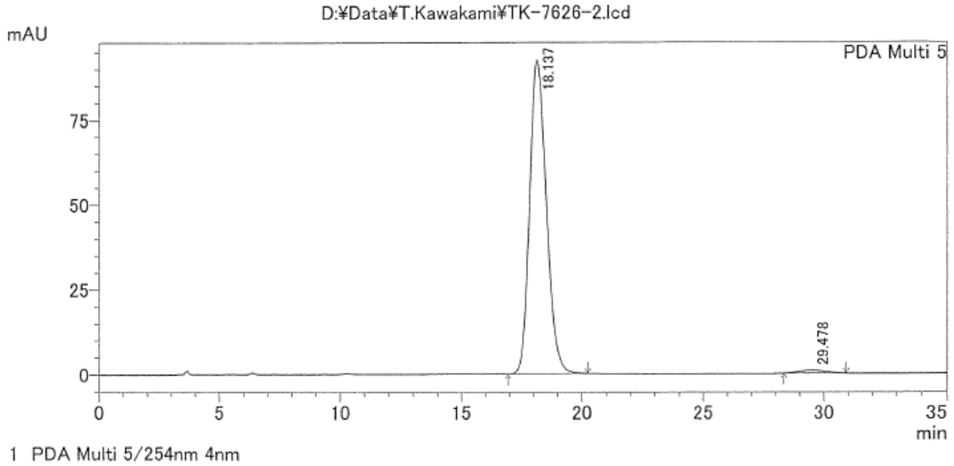

PDA Ch5 254nm 4nm

Peak No	RT (min)	Area	Height (mV)	% Area
1	18.137	4463637	92292	98.544
2	29.478	65972	894	1.456
Total		4529609	93186	100.000

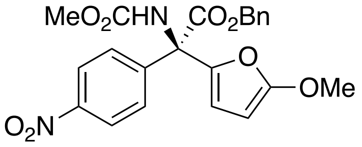

(**8g**)

AD-H,
n-hexane/*i*-PrOH = 1/1,
0.6 mL/min, 254 nm

Racemic **8g**

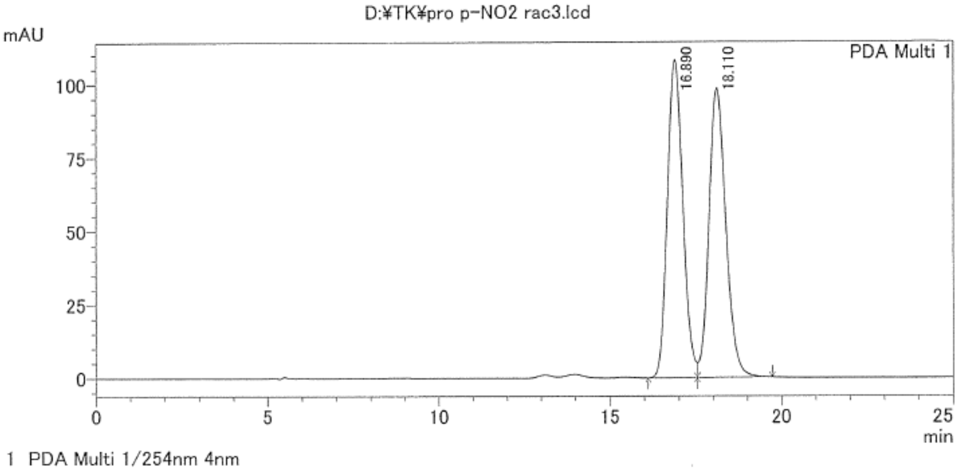

8g by the catalysis

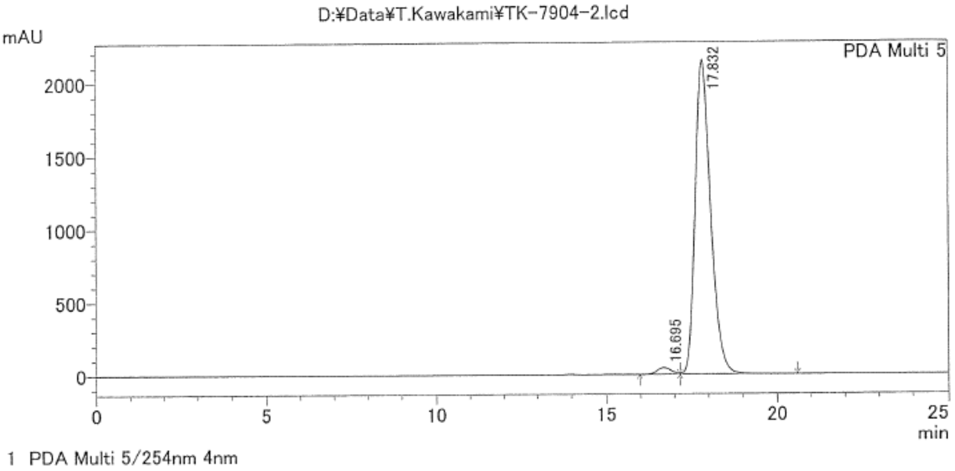

PDA Ch5 254nm 4nm

Peak No	RT (min)	Area	Height (mV)	% Area
1	16.695	1329395	45703	1.917
2	17.832	68014809	2147014	98.083
Total		69344204	2192716	100.000

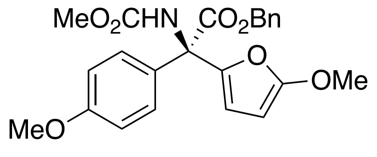

(**8h**)

AD-H,
n-hexane/*i*-PrOH = 1/1,
0.6 mL/min, 254 nm

Racemic **8h**

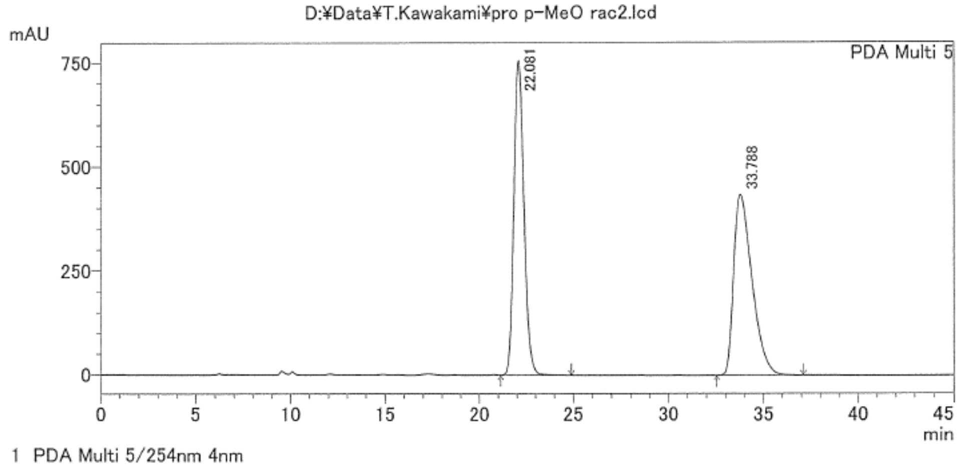

8h by the catalysis

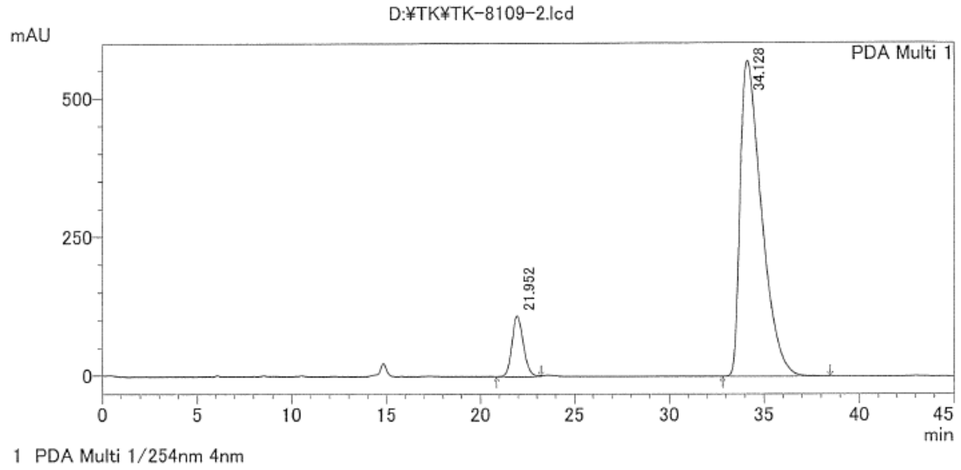

Peak Table

SPD-M20A Ch1 254nm 4nm

Peak No	RT (min)	Area	Height (mV)	% Area
1	21.952	4678468	109942	9.392
2	34.128	45134465	569126	90.608
Total		49812933	679068	100.000

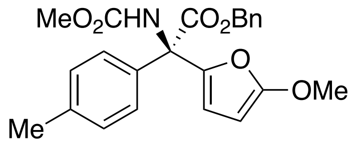

(**8i**)

AD-H,
n-hexane/*i*-PrOH = 1/1,
0.6 mL/min, 254 nm

Racemic **8i**

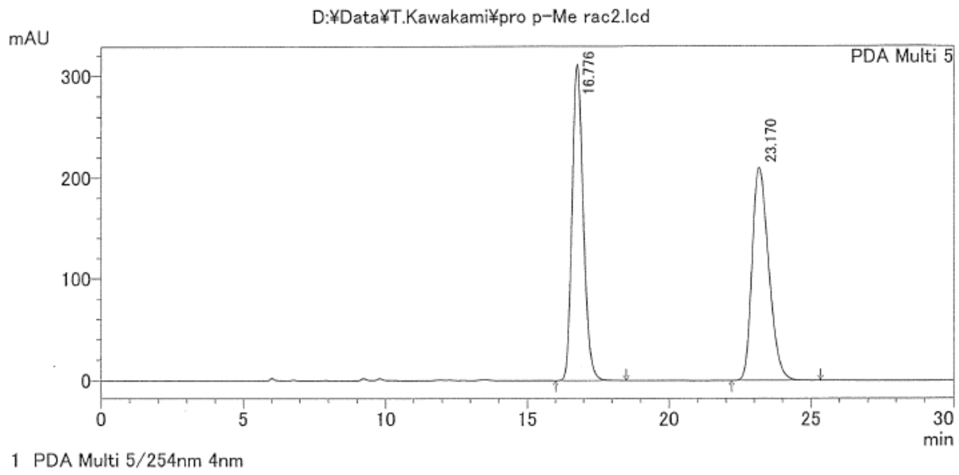

8i by the catalysis

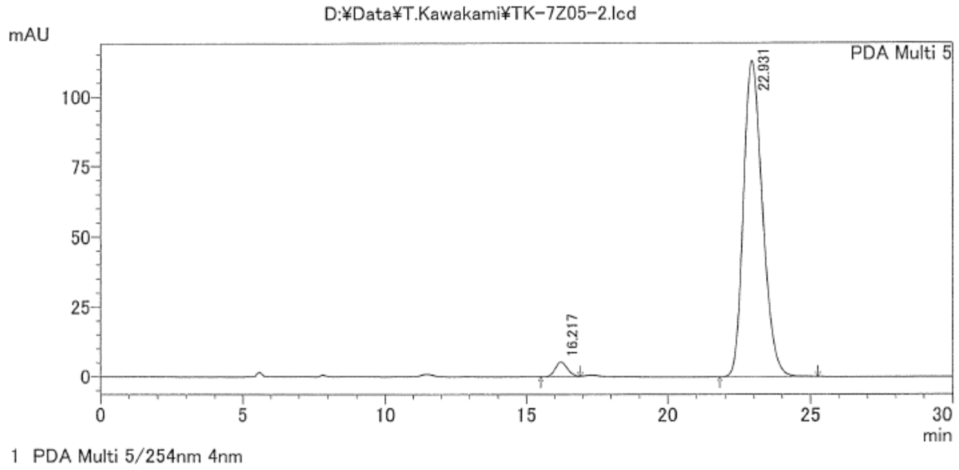

PDA Ch5 254nm 4nm

Peak No	RT (min)	Area	Height (mV)	% Area
1	16.217	162988	5275	3.000
2	22.931	5269158	112710	97.000
Total		5432147	117986	100.000

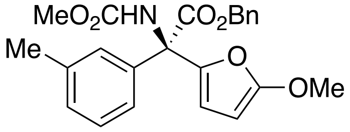

(**8j**)

AD-H,
n-hexane/*i*-PrOH = 1/1,
0.6 mL/min, 254 nm

Racemic **8j**

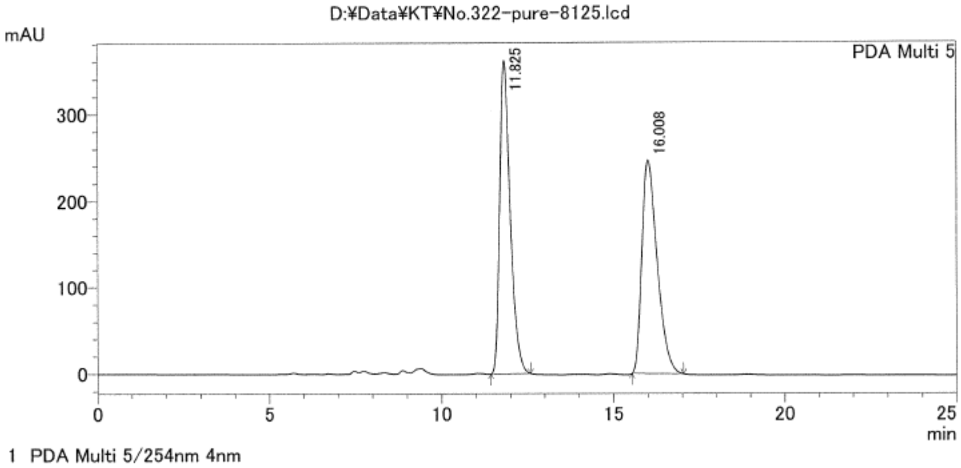

8j by the catalysis

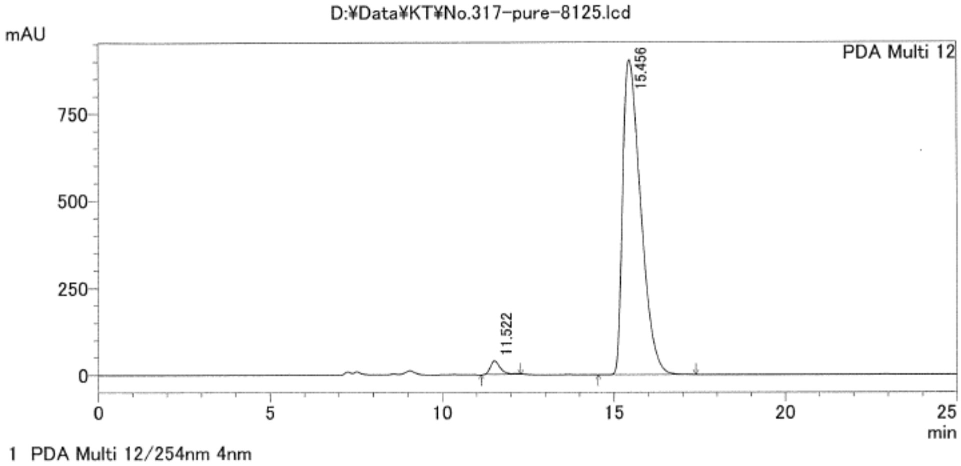

PDA Ch12 254nm 4nm

Peak No	RT (min)	Area	Height (mV)	% Area
1	11.522	778537	38657	2.309
2	15.456	32939379	903043	97.691
Total		33717916	941700	100.000

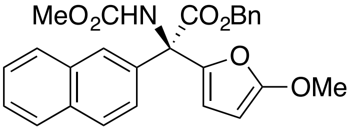

(**8l**)

AD-H,
n-hexane/*i*-PrOH = 1/1,
0.6 mL/min, 254 nm

Racemic **8l**

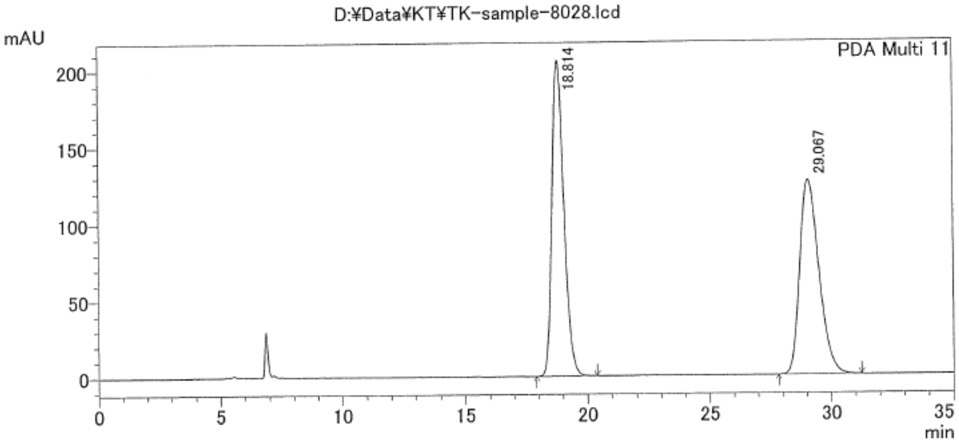

8l by the catalysis

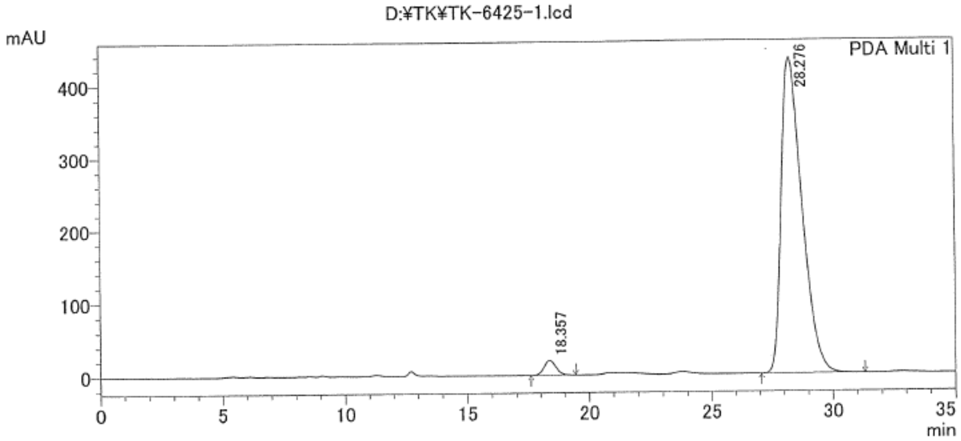

1 PDA Multi 1/254nm 4nm

Peak Table

SPD-M20A Ch1 254nm 4nm

Peak No	RT (min)	Area	Height (mV)	% Area
1	18.357	685474	20183	2.619
2	28.276	25490989	432946	97.381
Total		26176463	453129	100.000

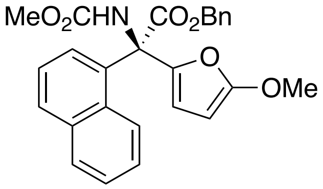

(**8m**)

OD-3,
n-hexane/*i*-PrOH = 1/1,
1.0 mL/min, 254 nm

Racemic **8m**

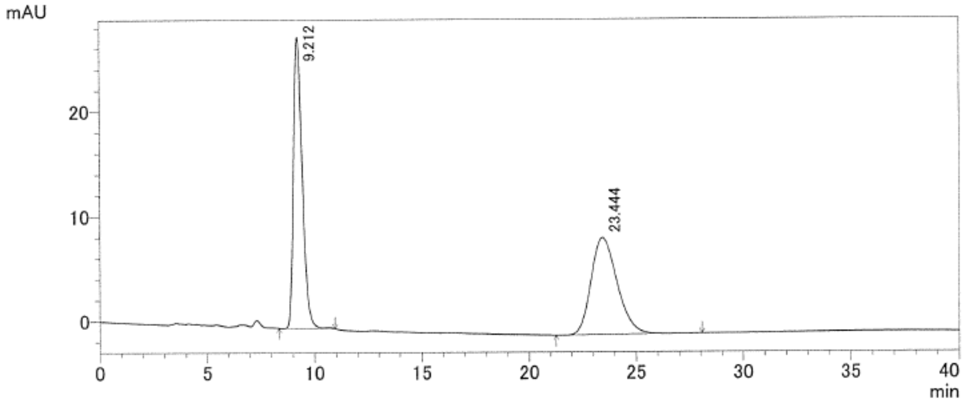

8m by the catalysis

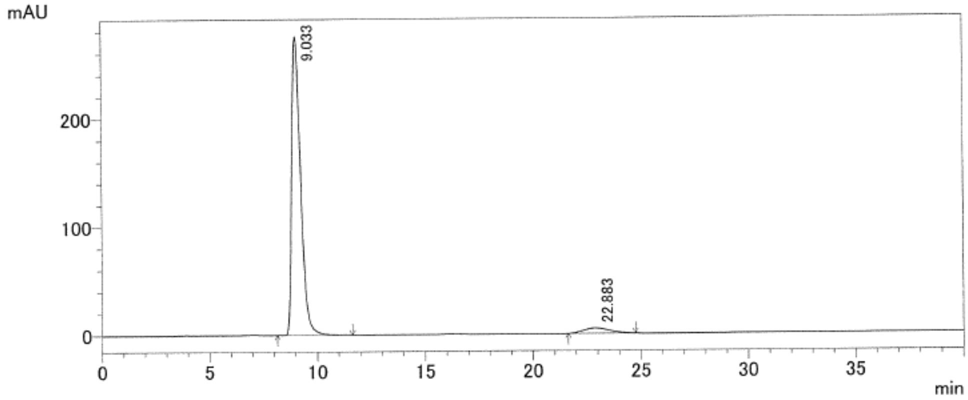

PDA Ch4 254nm

Peak No.	RT (min)	Area	Height	% Area
1	9.033	7927118	275428	95.057
2	22.883	412194	4901	4.943
Total		8339312	280329	100.000

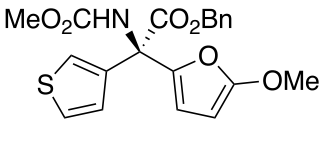

(**8n**)

AD-H,
n-hexane/*i*-PrOH = 1/1,
0.6 mL/min, 254 nm

Racemic **8n**

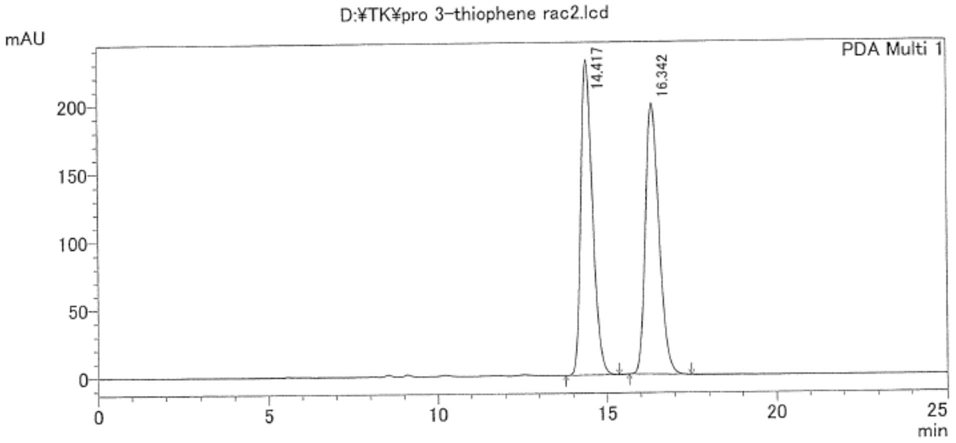

8n by the catalysis

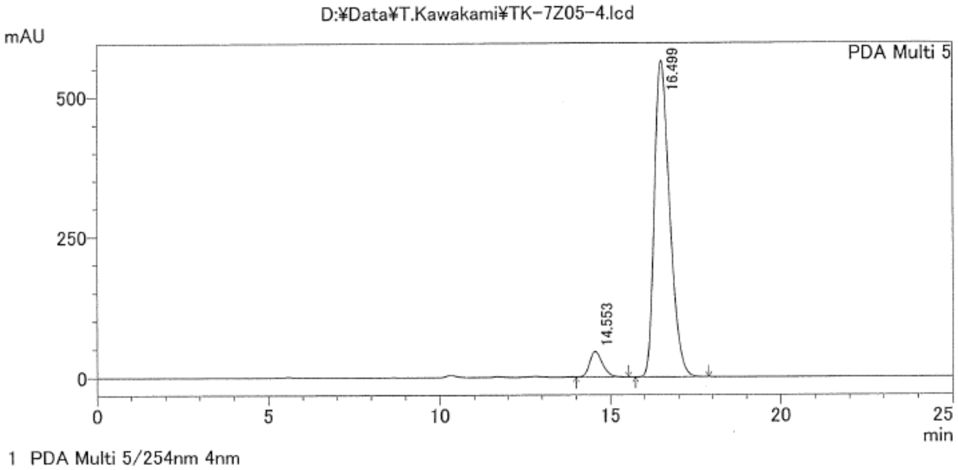

1 PDA Multi 5/254nm 4nm

PDA Ch5 254nm 4nm

Peak No	RT (min)	Area	Height (mV)	% Area
1	14.553	1213484	45498	6.415
2	16.499	17702452	562753	93.585
Total		18915936	608251	100.000

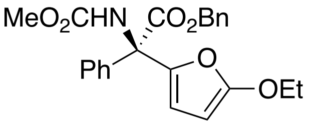

(**8p**)

AD-H,
n-hexane/*i*-PrOH = 1/1,
0.6 mL/min, 230 nm

Racemic **8p**

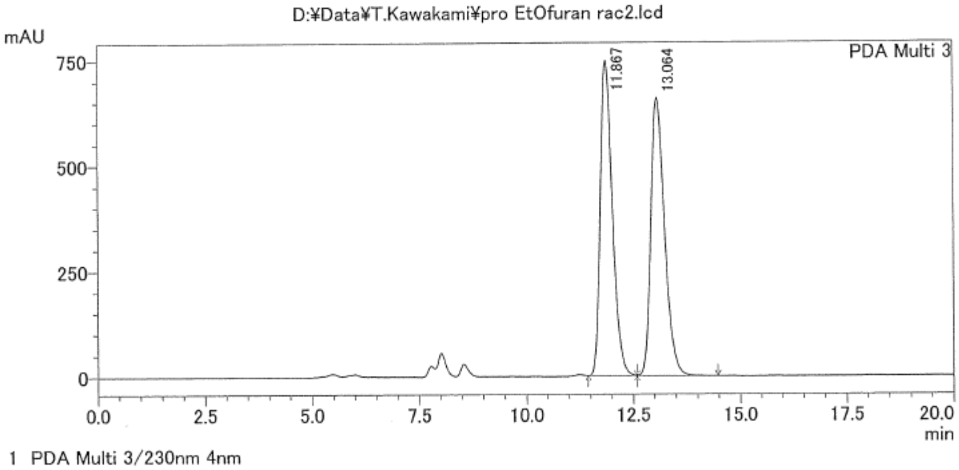

8p by the catalysis

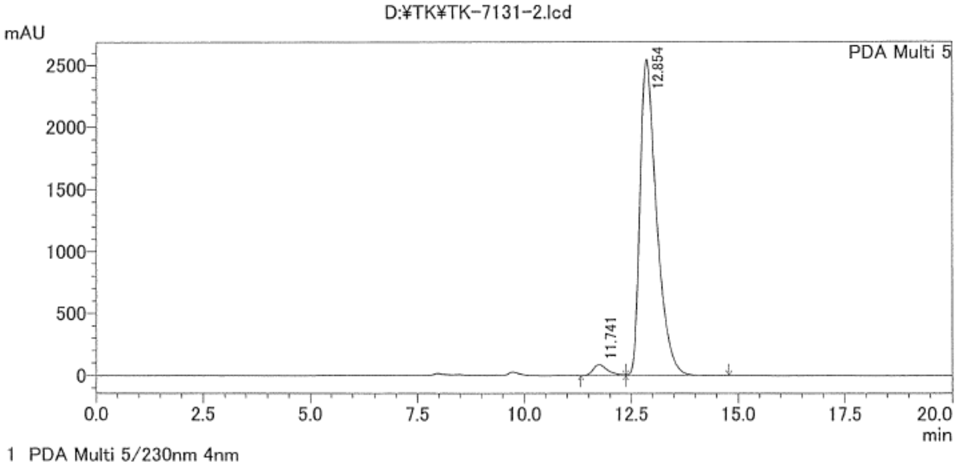

Peak Table

SPD-M20A Ch5 230nm 4nm

Peak No	RT (min)	Area	Height (mV)	% Area
1	11.741	2039458	86386	2.880
2	12.854	68767049	2544629	97.120
Total		70806508	2631015	100.000
